# Endothelial FOXC1 and FOXC2 promote intestinal regeneration after ischemia–reperfusion injury

**DOI:** 10.15252/embr.202256030

**Published:** 2023-05-08

**Authors:** Can Tan, Pieter R Norden, Wei Yu, Ting Liu, Naoto Ujiie, Sun Kyong Lee, Xiaocai Yan, Yaryna Dyakiv, Kazushi Aoto, Sagrario Ortega, Isabelle G De Plaen, Venkatesh Sampath, Tsutomu Kume

**Affiliations:** ^1^ Department of Medicine, Feinberg Cardiovascular and Renal Research Institute, Feinberg School of Medicine Northwestern University Chicago IL USA; ^2^ Division of Neonatology, Department of Pediatrics Children's Mercy Hospital Kansas City MO USA; ^3^ Department of Pediatrics, Feinberg School of Medicine Northwestern University Chicago IL USA; ^4^ Department of Biochemistry Hamamatsu University School of Medicine Hamamatsu Japan; ^5^ Mouse Genome Editing Unit, Biotechnology Program Spanish National Cancer Research Centre Madrid Spain

**Keywords:** CXCL12, intestinal regeneration, ischemia, paracrine signaling, Wnt/R‐spondin, Molecular Biology of Disease, Vascular Biology & Angiogenesis

## Abstract

Intestinal ischemia underlies several clinical conditions and can result in the loss of the intestinal mucosal barrier. Ischemia‐induced damage to the intestinal epithelium is repaired by stimulation of intestinal stem cells (ISCs), and paracrine signaling from the vascular niche regulates intestinal regeneration. Here, we identify FOXC1 and FOXC2 as essential regulators of paracrine signaling in intestinal regeneration after ischemia–reperfusion (I/R) injury. Vascular endothelial cell (EC)‐ and lymphatic EC (LEC)‐specific deletions of *Foxc1*, *Foxc2*, or both in mice worsen I/R‐induced intestinal damage by causing defects in vascular regrowth, expression of chemokine CXCL12 and Wnt activator R‐spondin 3 (RSPO3) in blood ECs (BECs) and LECs, respectively, and activation of Wnt signaling in ISCs. Both FOXC1 and FOXC2 directly bind to regulatory elements of the *CXCL12* and *RSPO3* loci in BECs and LECs, respectively. Treatment with CXCL12 and RSPO3 rescues the I/R‐induced intestinal damage in EC‐ and LEC‐*Foxc* mutant mice, respectively. This study provides evidence that FOXC1 and FOXC2 are required for intestinal regeneration by stimulating paracrine CXCL12 and Wnt signaling.

## Graphical abstract

The transcriptional activity of FOXC1 and FOXC2 in lymphatic and blood endothelial cells in the small intestine contributes to vascular repair and intestinal regeneration after ischemia‐reperfusion injury by regulating CXCL12 and R‐spondin3 signalling, respectively.

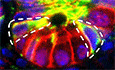

## Introduction

Tissue regeneration and repair are essential for maintaining physiological homeostasis and rely on the precise control of molecular networks that regulate, or are regulated by, the vasculature. Endothelial cells (ECs) present in the blood and lymphatic vessels are crucial participants in the vascular‐dependent processes that restore damaged tissue because they control the secretion of paracrine factors from both the vessels themselves and nearby cells (Rafii *et al*, 2016). Although significant progress has recently been made toward elucidation of the mechanisms by which the vascular system regulates tissue regeneration and repair, an adequate understanding of the biological processes that contribute to EC‐dependent tissue repair, as well as their roles in the pathogenesis and potential treatment of vascular disease or injury, is crucially dependent on a thorough characterization of how blood/lymphatic vessels control tissue regeneration.

Intestinal ischemia underlies several clinical conditions including ischemic bowel disease and can result in villus dysfunction, bacterial translocation, local and systemic inflammation, and intestinal necrosis in severe cases (Gonzalez *et al*, 2015; Ahmed, 2021). Intestinal ischemia can be caused by thrombus formation in the mesenteric vasculature, embolisms that arise as a consequence of cardiopulmonary disease, and disease‐ or shock‐induced declines in perfusion, as well as when blood flow is interrupted by intestinal transplantation (Gonzalez *et al*, 2015; Bertoni *et al*, 2018). Moreover, impairments in intestinal microvasculature development contribute to the pathogenesis of neonatal necrotizing enterocolitis (NEC), which is the most common devastating gastrointestinal emergency in neonatal patients (Bowker *et al*, 2018). Both homeostasis and repair of the small intestine are mediated via intestinal stem cells (ISCs; Barker *et al*, 2007; Barker, 2014). Active ISCs express the specific marker leucine‐rich repeat‐containing G protein‐coupled receptor 5 (Lgr5) and are located at the base of the crypts of the small intestine where they vigorously proliferate to continuously regenerate the intestinal epithelium. Ischemia/reperfusion (I/R) injury, as well as radiation injury and stresses such as acute inflammation, induce apoptosis in proliferating Lgr5^+^ ISCs (Richmond *et al*, 2018; Gonzalez *et al*, 2019), while ISC regeneration after injury is restored largely by dedifferentiation of crypt cells (Murata *et al*, 2020). However, the mechanisms that coordinate the role of intestinal ECs in intestinal regeneration and repair, and whether blood and lymphatic ECs (BECs and LECs) in the ISC niche regulate the regenerative activity of ISCs, as well as the preservation of the ISC niche after injury, have yet to be fully elucidated.

In adult mice, the proliferation of active ISCs is controlled, in part, by Wnt/β‐catenin signaling (Santos *et al*, 2018; Hageman *et al*, 2020), and canonical Wnt/β‐catenin signaling is promoted by the cooperative activity of Wnt proteins and R‐spondins (RSPO1‐RSPO4). Lgr5 functions (with Lgr4 and Lgr6) as a cognate receptor for R‐spondins and R‐spondins are expressed in mesenchymal stromal cells of the ISC niche (Shoshkes‐Carmel *et al*, 2018; McCarthy *et al*, 2020a), including Pdgfra^lo^Grem1^+^ trophocytes as an essential source of R‐spondins (RSPO1‐RSPO3, especially RSPO3; McCarthy *et al*, 2020b). Foxl1^+^ mesenchymal cells (Telocytes) and other nonepithelial stromal cells express Wnt ligands in the ISC niche (Farin *et al*, 2012; Valenta *et al*, 2016; Shoshkes‐Carmel *et al*, 2018). Both the number of Lgr5^+^ ISCs and the regenerative response to intestinal radiation injury are reduced by cotreatment with RSPO2‐ and RSPO3‐neutralizing antibodies (Storm *et al*, 2016), and the mucosal damage induced by intestinal I/R injury can be rescued by treatment with RSPO3 (Kannan *et al*, 2013). Most importantly, intestinal RSPO3 (Ogasawara *et al*, 2018; McCarthy *et al*, 2020a, 2020b) and Wnt2 (McCarthy *et al*, 2020b) are highly produced by LECs, and RSPO3 produced by intestinal LECs is required for maintaining ISCs in homeostasis and regeneration (Goto *et al*, 2022; Niec *et al*, 2022; Palikuqi *et al*, 2022).

The CXC chemokine CXCL12, also called stromal cell‐derived factor 1 (SDF‐1), is a homeostatic chemokine expressed in many cell types such as stromal cells, ECs, and fibroblasts in various tissues (García‐Cuesta *et al*, 2019). CXCL12 is an essential factor for angiogenesis that involves EC proliferation and migration to form neo‐vessel networks (Herbert & Stainier, 2011). CXCL12 can be induced by hypoxic stress (Hitchon *et al*, 2002; Santiago *et al*, 2011) and regulates angiogenesis in an autocrine/paracrine manner by interacting with the CXCR4 and CXCR7 receptors (Santagata *et al*, 2021). In addition, there is evidence that CXCL12/CXCR4 signaling promotes lymphangiogenesis (Zhuo *et al*, 2012).

FOXC1 and FOXC2 are closely related members of the FOX transcription factor family and have numerous essential roles in cardiovascular development, health, and disease (Golson & Kaestner, 2016). Mutations or changes in the copy number of human *FOXC1* are associated with autosomal‐dominant Axenfeld‐Rieger syndrome, which is characterized by abnormalities in the eye and extraocular defects (Seifi & Walter, 2018), while inactivating mutations of human *FOXC2* are responsible for the autosomal‐dominant syndrome Lymphedema‐distichiasis, which is characterized by obstructed lymph drainage in the limbs and the growth of extra eyelashes (Fang *et al*, 2000). There is also some evidence that *Foxc2* haploinsufficiency in mice increases their susceptibility to DDS‐induced colitis (Becker *et al*, 2015); however, the precise function of FOXC1 and FOXC2 in vascular repair and intestinal regeneration after ischemic injury have yet to be determined.

In this study, we report that FOXC1 and FOXC2 in intestinal BECs and LECs contribute to vascular repair and intestinal regeneration after I/R injury by regulating the expression of paracrine signaling factors. Inducible, EC‐ and LEC‐specific, single and compound mutant mice for *Foxc1* and *Foxc2* showed that the *Foxc* mutations impair regeneration of the small intestine after I/R injury, accompanied by (i) defective repair of intestinal BECs and LECs, (ii) reduced expression of CXCL12 and RSPO3 in intestinal BECs and LECs, respectively, and (iii) decreased activation of the Wnt/β‐catenin pathway in ISCs. Importantly, treatment with either CXCL12 or RSPO3 partially rescues the defects in intestinal repair and regeneration associated with EC‐ and LEC‐*Foxc1/c2* deficiency. Chromatin immunoprecipitation (ChIP) assays reveal that both FOXC1 and FOXC2 proteins bind to the regulatory elements of the *CXCL12* and *RSPO3* loci in BECs and LECs, respectively. Together, our data show a new role for FOXC1 and FOXC2 as key transcriptional regulators of paracrine signaling in the intestinal blood/lymphatic vessels during postischemic intestinal repair/regeneration, and our findings may have important implications for the treatment of ischemic bowel disease by modulating the vascular paracrine signaling pathways.

## Results

### *Foxc1* and *Foxc2* expression in the murine intestine

**Figure 1:**
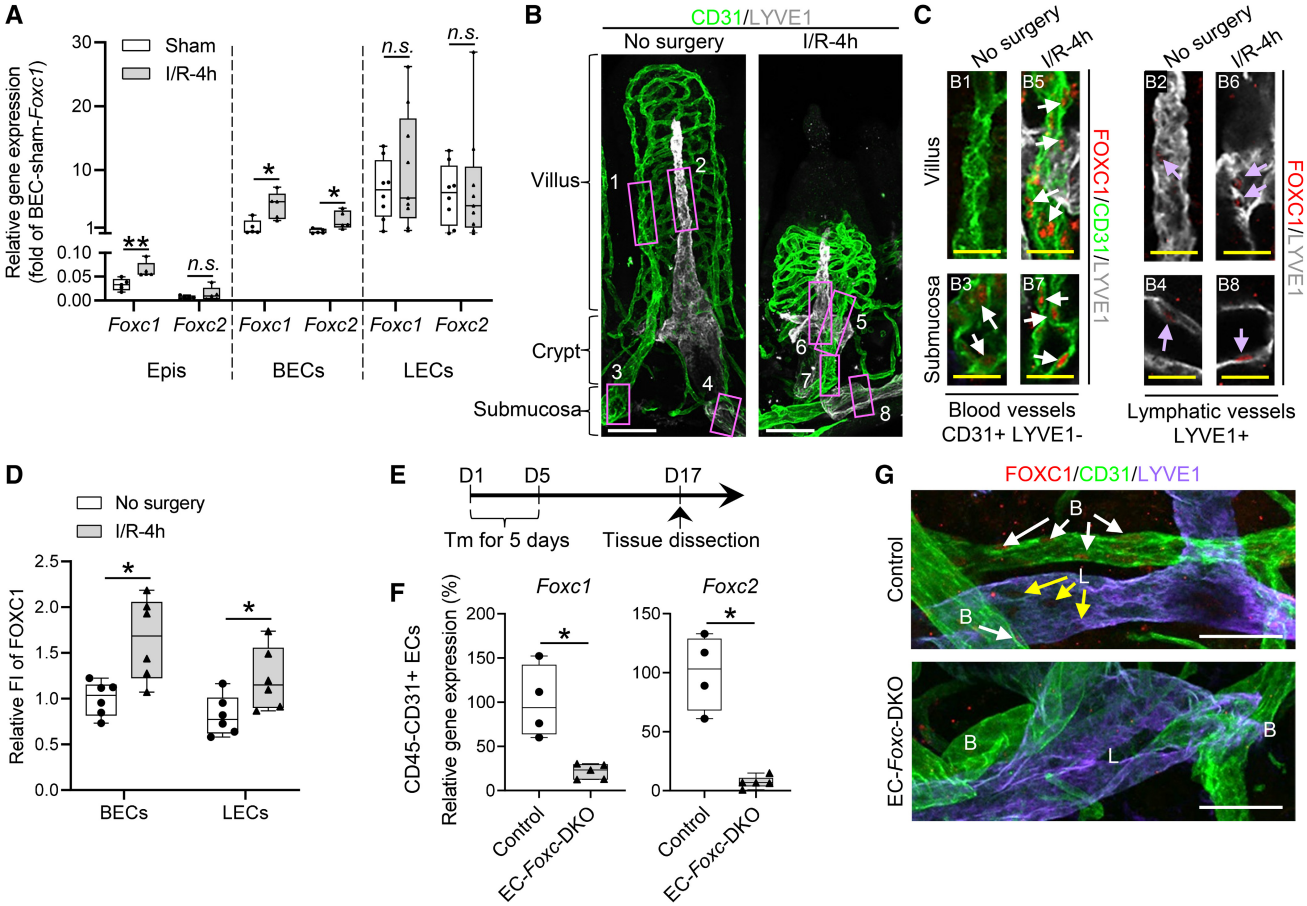
Expression levels of *Foxc1* and *Foxc2* in the mouse small intestine ARelative mRNA expression of *Foxc1* and *Foxc2* in isolated blood endothelial cells (BECs) and lymphatic endothelial cells (LECs) from *Foxc1* ^*fl/fl*^;*Foxc2* ^*fl/fl*^ mouse distal jejuna in sham and I/R‐4 h groups. Isolated epithelial cells (Epis) were used as a relative control, Data are box‐and‐whisker plots, Mann–Whitney *U* test, each symbol represents one mouse, *N* = 5 ~ 9, *P < 0.05, **P < 0.01, *n.s*., not significant.B, CRepresentative confocal images of the whole‐mount intestine stained with CD31/LYVE1 (B), FOXC1/CD31/LYVE1 (C, left), and FOXC1/LYVE1 (C, right) in *Foxc1* ^*fl/fl*^;*Foxc2* ^*fl/fl*^ mice. Images of maximum intensity projections (B) show the intestinal blood (green) and lymphatic (white) vasculatures from villous to submucosa. The regions in pink box 1 ~ 8 in Fig 1B are chosen for the specific images in Fig 1C. Scale bars = 50 μm. (C) Images of optical sections with high‐magnification chosen from specific regions shown in Fig 1B (B1 ~ B8), with an additional channel of FOXC1 staining (red), show the upregulation of FOXC1 in BECs (white arrows in blood vessels) and LECs (violet arrows in lymphatic vessels) at the level of villus and submucosa 4 h after I/R. Scale bars = 20 μm.DQuantification of fluorescent intensity (FI) of FOXC1 staining in FOXC1^+^ BECs and LECs based on the whole‐mount staining as shown in Fig 1C. Data are box‐and‐whisker plots, Mann–Whitney *U* test, each symbol represents one mouse, *N* = 6, *P < 0.05.ESchematic showing the time of tamoxifen (Tm) injection and tissue dissection for Fig 1F and G.FRelative mRNA expression of *Foxc1* and *Foxc2* in isolated CD45^−^CD31^+^ ECs from distal jejuna 12 days after Tm treatment. Data are box‐and‐whisker plots, Mann–Whitney *U* test, each symbol represents one mouse, *N* = 4 ~ 5, *P < 0.05.GRepresentative confocal images of whole‐mount intestines stained with FOXC1/CD31/LYVE1 showing intestinal submucosal blood (B) and lymphatic (L) vessels. In control submucosa, FOXC1 is detected in the nuclei of both BECs (white arrows) and LECs (yellow arrows), but FOXC1 expression in LECs is much weaker than that in BECs. FOXC1 is downregulated in both BECs and LECs after Tm treatment in EC‐*Foxc*‐DKO intestinal submucosa compared with control. Scale bars = 50 μm. Note that the red tiny spots are nonspecific staining. Data information: The box‐and‐whisker plots in (A), (D), and (F) display the median value (central band in the box), second and third quartiles (bottom and top ends of the box, respectively), as well as minimum/maximum values (whiskers blow/above the box) of the data sets. Source data are available online for this figure.

*Foxc1* and *Foxc2* mRNA expression in intestinal BECs and LECs was evaluated via quantitative PCR (qPCR) of BECs and LECs isolated from the small intestine in adult mice (Fig 1A, Sham). Intestinal ECs are particularly vulnerable to I/R injury, and they undergo apoptosis in response to oxidative stress (Bertoni *et al*, 2018). Thus, we next investigated whether I/R injury alters *Foxc1/Foxc2* expression in the small intestine of adult mice. Intestinal I/R injury (Yoshiya *et al*, 2011) was induced by clamping the superior mesenteric artery for 30 min to induce occlusion; then, the clip was removed, and the small intestine was allowed to reperfuse. *Foxc1/Foxc2* expression was significantly greater in the intestinal BECs of mice that underwent I/R injury than in the BECs of sham‐operated animals (Fig 1A). These results are consistent with previous studies showing that FOXC2 expression in renal tubular cells of the cortex and outer medulla also increases 24 h after kidney I/R injury (Hader *et al*, 2010). There's an increased but not significant trend of *Foxc1* and *Foxc2* in LECs after I/R injury. *Foxc1* but not *Foxc2* was also found increased in epithelial cells (Epis), which were used as a relative control (Fig 1A).

**Figure EV1:**
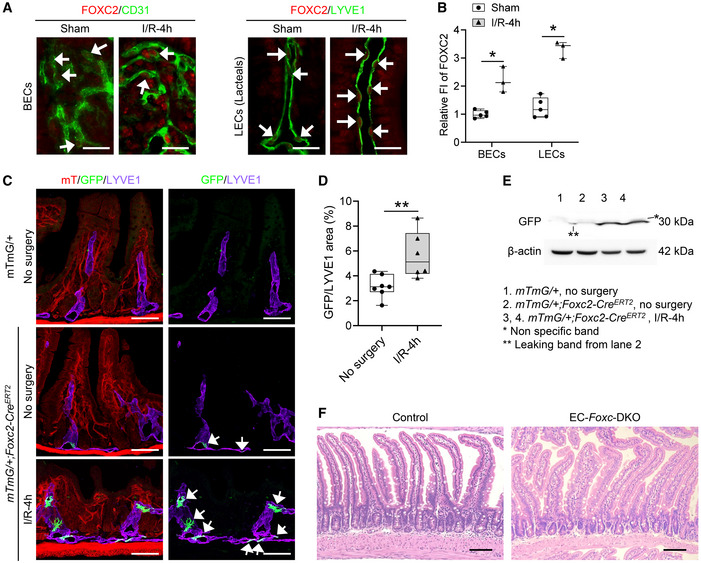
FOXC2 expression in intestinal ECs Representative immunostaining images of villi show FOXC2 is upregulated in intestinal BECs (CD31^+^) and LECs (LYVE1^+^) indicated by arrows after I/R at 4 h in control adult mice (*Foxc1* ^*f/f*^;*Foxc2* ^*f/f*^). Scale bars = 20 μm.Quantification of fluorescent intensity (FI) of FOXC2 in BECs and LECs was performed based on the IHC staining as shown in Fig EV1A. Data are box‐and‐whisker plots, Mann–Whitney *U* test, each symbol represents one mouse, *N* = 3 ~ 5, *P < 0.05.Mice were treated with Tm for 5 days and subjected to intestinal I/R surgery 12 day post‐Tm treatment. Representative intestinal mucosal images of GFP/LYVE1 immunostaining with mT signals on frozen sections (15 μm) in *mTmG*/+;*Foxc2‐Cre* ^*ERT2*^ mice without surgery or 4 h after I/R. *mTmG*/+ mice without surgery were used as control. FOXC2‐GFP^+^ cells (arrows) were found mainly in LYVE1^+^ lymphatic vessels. The increased number of FOXC2‐GFP^+^ cells found in LECs after I/R suggested the proliferation of FOXC2‐GFP^+^ LECs induced by I/R. Scale bars = 100 μm.Quantification of the density of GFP^+^ cells in lymphatic vessels. GFP/LYVE1 area (%) = (GFP^+^ area)/(LYVE1^+^ area) × 100%. Data are box‐and‐whisker plots, Mann–Whitney *U* test, each symbol represents one mouse, *N* = 6 ~ 7, **P < 0.01.Representative western blots show the increased level of FOXC2‐GFP in intestinal lysates of *mTmG*/+;*Foxc2‐Cre* ^*ERT*2^ mice 4 h after I/R compared with the mice without surgery.Representative H&E staining images of intestinal mucosa in control and EC‐*Foxc*‐DKO mice after Tm treatment without surgery. Scale bars = 100 μm. Data information: The box‐and‐whisker plots in (B) and (D) display the median value (central band in the box), second and third quartiles (bottom and top ends of the box, respectively), as well as minimum/maximum values (whiskers blow/above the box) of the data sets. Source data are available online for this figure.

To further investigate FOXC1 protein expression in the adult mouse intestinal ECs, we performed whole‐mount immunostaining labeled with CD31 (EC marker), LYVE1 (LEC marker), and FOXC1 (Fig 1B and C). In the villus, FOXC1 protein was hardly detectable in both BECs and LECs (Fig 1C, B1 and B2). In the submucosa, FOXC1 was detected in both BECs and LECs, while the levels of FOXC1 in LECs were much weaker than those in BECs. (Fig 1C, B3 and B4). Four hours after I/R injury, intestinal blood and lymphatic vessels were severely damaged (Fig 1B), and FOXC1 protein was upregulated in both intestinal BECs (Fig 1C, B5 and B7) and LECs (Fig 1C, B6 and B8) (Fig 1D). Similar to FOXC1, FOXC2 protein was also upregulated in both intestinal BECs and LECs (Fig EV1A and B) after I/R injury. Together, these results indicate that both FOXC1 and FOXC2 are upregulated in intestinal BECs and LECs after I/R injury.

The levels of FOXC2 were low in LECs and hardly detectable in BECs in sham intestine (Fig EV1A). To further confirm the expression pattern of FOXC2 in the intestinal vasculature in sham and after I/R injury, we crossed tamoxifen‐inducible *Foxc2‐Cre* ^*ERT2*^ knock‐in mice (Amin *et al*, 2017) with dual Rosa26mTmG reporter mice (Muzumdar *et al*, 2007). Adult *mTmG*/+*;Foxc2‐Cre* ^*ERT2*^ mice were then treated with tamoxifen (150 mg/kg) by oral gavage for 5 consecutive days and subjected to the I/R injury 12 day post tamoxifen treatment. FOXC2‐GFP^+^ cells were detected mainly in the LECs in sham‐ and I/R‐intestines (Fig EV1C). The number of FOXC2‐GFP^+^ LECs (Fig EV1D) and the intestinal GFP expression (Fig EV1E) were increased after I/R, suggesting the proliferation of FOXC2^+^ LECs induced by I/R injury.

**Figure EV2:**
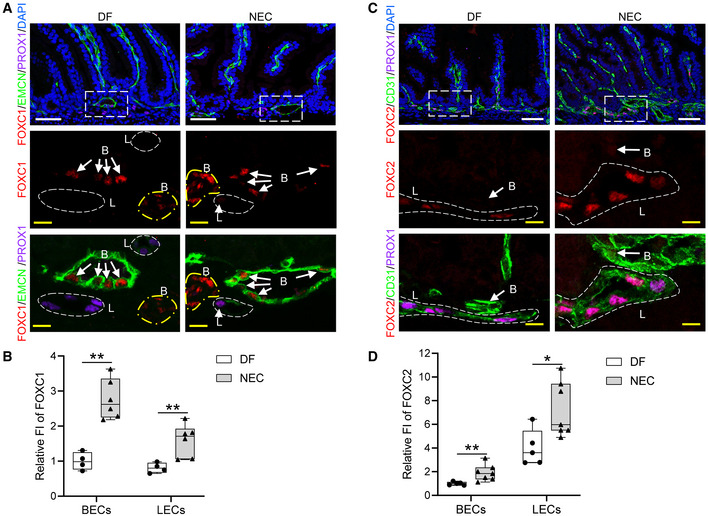
FOXC1 and FOXC2 are increased in BECs and LECs in mouse NEC model A–DImmunostaining of (A) FOXC1/EMCN/PROX1/DAPI and (C) FOXC2/CD31/PROX1/DAPI was performed on paraffin sections (4 μm) of small intestines from 2‐day old neonatal mice 24 h after being subjected to the necrotizing enterocolitis (NEC) protocol (Tian *et al*, 2010). Dam‐fed (DF) pup littermates were used as control. (A) FOXC1 is mainly expressed in BECs in intestinal submucosa. The level of FOXC1 is increased obviously in submucosal BECs (EMCN^+^, B with arrow; as well as yellow circled area) in NEC intestine compared with DF intestine. FOXC1^+^ cells in yellow circled area are arterial BECs with EMCN^−^. FOXC1 can be found weakly expressed in the submucosal LECs (L with arrow, PROX1^+^) in NEC intestine but is hardly detectable in LECs in DF intestine. PROX1 is a nuclear marker for LECs. White/yellow scale bars = 50 or 10 μm, respectively. (C) FOXC2 can be detected in LECs (L, CD31^+^PROX1^+^; circled) in DF intestine, and is increased in LECs in NEC intestine compared with DF intestine. FOXC2 can be found weakly expressed in the submucosal BECs (B, CD31^+^PROX1^−^) in NEC intestine (white arrow) but is hardly detectable in BECs in DF intestine. White/yellow scale bars = 50 or 10 μm, respectively. Quantification of fluorescent intensity (FI) of (B) FOXC1 and (D) FOXC2 in submucosal BECs and LECs was performed based on the IHC staining as shown in Fig EV2A and C, respectively. Data are box‐and‐whisker plots, Mann–Whitney *U* test, each symbol represents one mouse, *N* = 4 ~ 7, *P < 0.05, **P < 0.01. The box‐and‐whisker plots display the median value (central band in the box), second and third quartiles (bottom and top ends of the box, respectively), as well as minimum/maximum values (whiskers blow/above the box) of the data sets. Source data are available online for this figure.

Intestinal ischemia is associated with a broad range of clinical conditions such as neonatal necrotizing enterocolitis (NEC), which is characterized by gut microbiota induced intestinal inflammation and injury and intestinal ischemia arising from derangements in the intestinal microcirculation (Koike *et al*, 2020; Cuna *et al*, 2021), as well as in term infants with congenital heart disease who have development of intestinal necrosis (Young *et al*, 2011). We therefore examined the expression patterns of FOXC1 and FOXC2 in the intestine in a mouse NEC model (Tian *et al*, 2010), which includes initial orogastric inoculation of neonatal mice with a standardized adult mouse commensal bacteria preparation and lipopolysaccharide (LPS) to perturb the normal intestinal colonization process, gavage with formula every 3 h, and exposure to brief episodes of hypoxia for 1 min followed immediately by cold stress (10 min at 4°C) twice daily. With this protocol, about 50–70% of mice typically develop intestinal injuries ranging from epithelial injury to transmural necrosis between 36 and 72 h (Tian *et al*, 2010). At 24 h after the neonates were subjected to the NEC protocol, immunohistochemical analyses of BEC (CD31 and endomucin [EMCN]) and LEC (PROX1) markers revealed that levels of FOXC1 and FOXC2 proteins were increased in intestinal BECs and LECs (Fig EV2A–D), compared with dam‐fed (DF) littermate controls.

### Generation of tamoxifen‐inducible, EC‐specific, *Foxc1/c2*‐mutant mice in the adult

**Figure EV3:**
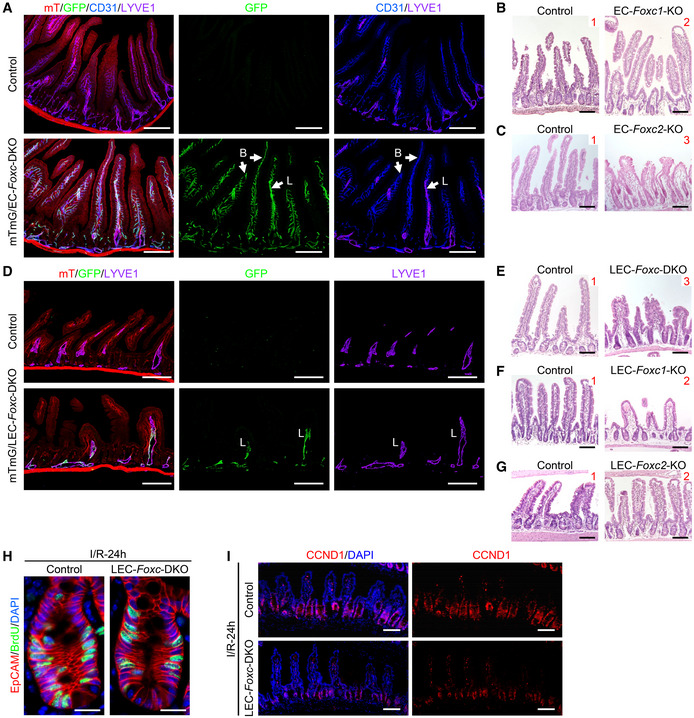
Histological detection in EC‐ and LEC‐ specific *Foxc* KO mouse intestines A–G(A and D) Cre recombination efficiency detection in EC‐*Foxc*‐DKO and LEC‐*Foxc*‐DKO mouse strains. (A) Mice (control: *mTmG*/+;*Foxc1* ^*f/f*^;*Foxc2* ^*f/f*^, mTmG/EC‐*Foxc*‐DKO: *mTmG*/+;*Cdh5‐Cre* ^*ERT2*^;*Foxc1* ^*f/f*^;*Foxc2* ^*f/f*^) were treated with 150 mg/kg Tm by oral gavage for 5 days. Seven days after Tm treatment, the distal jejunum was collected and the frozen sections (15 μm) were stained with GFP/CD31/LYVE1 for the detection of GFP signal in blood vessels (B, CD31^+^LYVE1^−^, blue) and lacteals (L, CD31^+^LYVE1^+^, purple). Scale bars = 200 μm. (D) Mice (control: *mTmG*/+;*Foxc1* ^*f/f*^;*Foxc2* ^*f/f*^, mTmG/LEC‐Foxc‐DKO: *mTmG*/+;*Vegfr3‐Cre* ^*ERT2*^;*Foxc1* ^*f/f*^;*Foxc2* ^*f/f*^) were treated with 150 mg/kg tamoxifen by oral gavage for 5 days. Twelve days after Tm dose, the distal jejunum was collected and the frozen sections (15 μm) were stained with GFP and LYVE1 antibodies. Confocal images show the VEGFR3‐GFP is expressed in the LYVE1^+^ lymphatic vessels (L). Scale bars = 200 μm. (B, C, E–G) Representative H&E staining images of the distal jejuna 24 h after I/R in different mouse strains: (B) EC‐*Foxc1*‐KO, (C) EC‐*Foxc2*‐KO, (E) LEC‐*Foxc*‐DKO, (F) LEC‐*Foxc1*‐KO, (G) LEC‐*Foxc2*‐KO and their control mice. The intestinal ischemic injury grading in the Chiu scoring system is indicated by red numbers (0 ~ 5). Scale bars = 100 μm. The quantification of Chiu Score for these mouse strains is shown in Figs 2D and E, and 3B–D, respectively.HRepresentative immunostaining images of intestinal crypts labeled with BrdU (proliferative marker, injection performed 2 h before tissue collection) and EpCAM (epithelial marker) show the proliferative epithelial cells in crypts. Paraffin sections (4 μm), scale bars = 20 μm. The quantification of the number of BrdU^+^ epithelial cells per crypt is shown in Fig 3E.IRepresentative images of intestinal mucosa labeled with Cyclin D1 (CCND1) in LEC‐*Foxc*‐DKO mice compared with the control group 24 h after I/R. Scale bars = 100 μm. Quantification data for CCND1^+^ epithelial cells per crypt are shown in Fig 3I. Source data are available online for this figure.

Murine *Foxc1* and *Foxc2* are both required for vascular development (Kume *et al*, 1998, 2001; Seo *et al*, 2006) but attempts to determine how the two genes function during *pathological* (lymph)angiogenesis have been generally unsuccessful because global single and compound *Foxc1/Foxc2*‐mutant mice die perinatally with severe cardiovascular abnormalities (Kume *et al*, 2001). Therefore, we crossed conditional‐null *Foxc1* ^*fl*^ and *Foxc2* ^*fl*^ mutant mice (Sasman *et al*, 2012) with *Cdh5‐Cre* ^*ERT2*^ mice (Sorensen *et al*, 2009) to generate tamoxifen‐inducible, EC‐specific, compound *Foxc1;Foxc2*‐mutant (*Cdh5*‐*Cre* ^*ERT2*^;*Foxc1* ^*fl/fl*^ *;Foxc2* ^*fl/fl*^) mice, which (after the mutation is induced) are referred to as EC‐*Foxc*‐DKO mice (Norden *et al*, 2020). To induce the mutations, adult mice were treated with tamoxifen (150 mg/kg) by oral gavage for 5 consecutive days, and 12 days after tamoxifen treatment the tissue was collected (Fig 1E), qPCR and immunohistochemical analyses confirmed that *Foxc1* and *Foxc2* expression was significantly reduced in intestinal ECs of EC‐*Foxc*‐DKO mice than in the corresponding cells of control littermates (Fig 1F and G). Importantly, the small intestines of EC‐*Foxc*‐DKO mice appeared morphologically normal 12 days after tamoxifen treatment (Fig EV1F), suggesting that EC expression of *Foxc1* and *Foxc2* is not required for maintaining intestinal epithelium homeostasis. We also crossed EC‐*Foxc*‐DKO mice with Rosa26mTmG reporter mice (Muzumdar *et al*, 2007), then treated their adult offspring with tamoxifen as described above, and immunohistochemically identified recombined EGFP^+^ cells in intestinal blood (CD31^+^LYVE1^−^) and lymphatic (CD31^+^LYVE1^+^) vessels to confirm Cre‐mediated recombination in intestinal BECs and LECs (Fig EV3A).

### EC‐specific deletion of *Foxc1* and *Foxc2* impairs intestinal mucosal recovery after I/R injury

**Figure 2:**
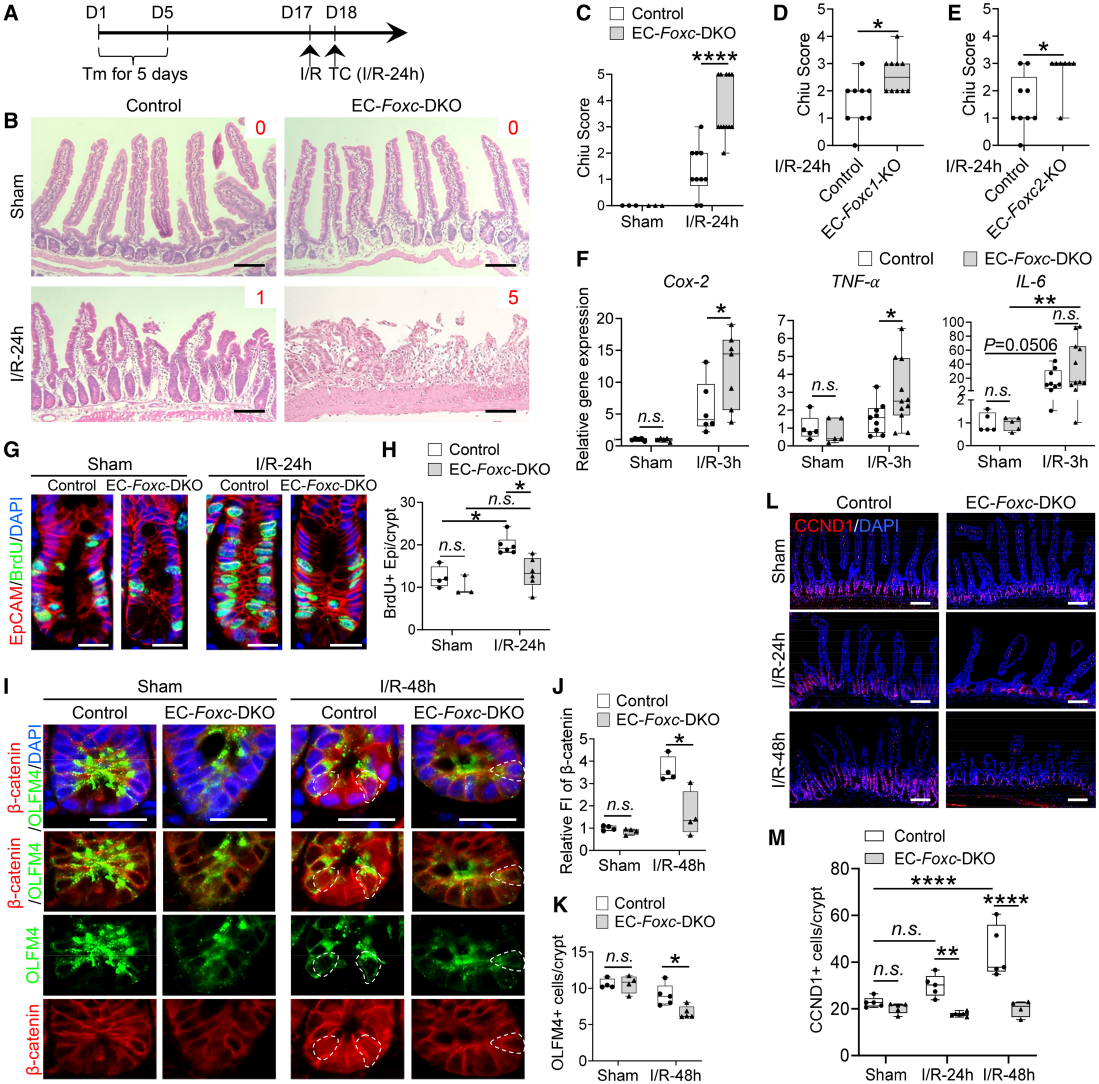
Characterization of defects in the intestinal mucosa in mice with EC‐specific deletion of *Foxc1/2* after I/R ASchematic showing the time of tamoxifen (Tm) injection, I/R surgery, and tissue collection (TC).BRepresentative H&E staining images of the distal jejuna in EC‐*Foxc*‐DKO and control mice 24 h after I/R. The intestinal ischemic injury grading in the Chiu scoring system is indicated by red numbers (0 ~ 5). Scale bars = 100 μm.C–EQuantification of Chiu Score for control and EC‐*Foxc*‐DKO (C), control and EC‐*Foxc1*‐KO (D), and control and EC‐*Foxc2*‐KO groups (E) at I/R‐24 h. Figure 2D and E are based on Fig EV3B and C. Data are box‐and‐whisker plots, Mann–Whitney *U* test, each symbol represents one mouse, *N* = 3 in (C) sham groups, *N* = 7 ~ 12 in (C–E) I/R‐24 h groups, *P < 0.05, ****P < 0.0001.FRelative mRNA expression of proinflammatory mediators *Cox2*, *TNF‐α*, and *IL‐6* from intestinal tissue lysates in sham and I/R‐3 h groups. Data are box‐and‐whisker plots, Mann–Whitney *U* test for *Cox2* and *TNF‐α*, Kruskal–Wallis one‐way ANOVA test for *IL‐6*, each symbol represents one mouse, *N* = 5 ~ 11, *P < 0.05, **P < 0.01, *n.s*., not significant.GRepresentative immunostaining images of intestinal crypts labeled with BrdU (proliferative marker, injection performed 2 h before tissue collection) and EpCAM (epithelial marker) show the proliferation of epithelial cells in crypts. Paraffin sections (4 μm), scale bars = 20 μm.HQuantification of the number of BrdU^+^ epithelial cells per crypt based on Fig 2G. Data are box‐and‐whisker plots, Kruskal–Wallis one‐way ANOVA test, each symbol represents one mouse, *N* = 3 ~ 6, *P < 0.05, *n.s*., not significant.IRepresentative images of intestinal crypts immunostained with β‐catenin and the intestinal epithelial stem cell (ISC) marker OLFM4. At I/R‐48 h, the nuclear translocation of β‐catenin in ISCs (dotted circles) was found in the control, whereas it was seldom found in EC‐*Foxc*‐DKO mice. Paraffin sections (4 μm), scale bars = 20 μm.J, K(J) Quantification of relative fluorescent intensity (FI) of β‐catenin immunostaining within ISC and (K) quantification of the number of OLFM4^+^ ISCs per crypt were performed based on Fig 2I. Data are box‐and‐whisker plots, Mann–Whitney *U* test, each symbol represents one mouse, *N* = 4 ~ 5, *P < 0.05, *n.s*., not significant.LRepresentative images of intestinal mucosa labeled with Cyclin D1 (CCND1) in EC‐*Foxc*‐DKO mice compared with the control group in sham, 24 h, and 48 h after I/R. Paraffin sections (4 μm), scale bars = 100 μm.MQuantification of the number of CCND1^+^ epithelial cells per crypt based on Fig 2L. Data are box‐and‐whisker plots, Kruskal–Wallis one‐way ANOVA test, each symbol represents one mouse, *N* = 4 ~ 6, **P < 0.01, ****P < 0.0001, *n.s*., not significant. Data information: The box‐and‐whisker plots in (C–F), (H), (J), (K), and (M) display the median value (central band in the box), second and third quartiles (bottom and top ends of the box, respectively), as well as minimum/maximum values (whiskers blow/above the box) of the data sets. Source data are available online for this figure.

When intestinal I/R injury was induced 12 days after tamoxifen treatment in adult mice (Fig 2A), tissue from the distal jejunum was histologically graded and quantified 24 h after I/R injury according to the Chiu scoring system (Chiu *et al*, 1970). Compared with the control mice, the intestinal mucosa remained severely injured in EC‐*Foxc*‐DKO mice (Fig 2B and C). To further characterize how the loss of EC‐specific *Foxc1/c2* expression affects the repair of the intestinal mucosa during recovery from I/R injury, we also examined mice carrying tamoxifen‐inducible, EC‐specific mutations of each individual gene (i.e., EC‐*Foxc1*‐KO and EC‐*Foxc2*‐KO mice) and their control littermates. Recovery from intestinal damage was impaired in both EC‐*Foxc1*‐KO and EC‐*Foxc2*‐KO mice 24 h after I/R injury (Figs 2D and E, and EV3B and C). As the extent of intestinal injury in the EC‐specific *Foxc* single mutant mice was less than that in the EC‐double *Foxc1/c2‐*mutant mice, these results indicate that EC‐*Foxc1* and ‐*Foxc2* expression is required for intestinal epithelial regeneration. Since I/R‐induced local inflammatory response is critically associated with intestinal damage (Cerqueira *et al*, 2005; Kannan *et al*, 2013), mRNA levels of proinflammatory regulators (*Cox2*, *TNF‐α*, and *IL‐6*) were measured under homeostasis and at an early time point (3 h) after I/R injury via qPCR. No significant difference was found in the levels of these regulators between sham‐operated mice (Fig 2F). After I/R injury, expression levels of *Cox2* and *TNF‐α* were significantly higher in EC‐*Foxc*‐DKO mice than in their control mice (Fig 2F), whereas increased *IL‐6* expression in EC‐*Foxc*‐DKO mice exhibited a trend toward significance (*P* = 0.2014) vs. the control mice but a significant increase vs. the sham‐operated EC‐*Foxc*‐DKO mice. Furthermore, EC‐specific loss of *Foxc1/c2* significantly reduced the proliferative response of intestinal epithelial cells to I/R injury as assessed via immunostaining of EpCAM (intestinal epithelial cell marker) and BrdU (proliferative marker; Fig 2G), as well as the quantification of BrdU^+^ epithelial cell number per crypt (Fig 2H).

### Wnt signaling in the small intestine is diminished in EC‐*Foxc*‐DKO mice after I/R injury

β‐catenin regulates the maintenance and regeneration of intestinal epithelial cells (Santos *et al*, 2018; Hageman *et al*, 2020) by translocating from the cytosol to the nucleus of ISCs and altering gene expression in response to activation of the canonical Wnt signaling pathway. This mechanism is consistent with our observations in control mice, because although β‐catenin was located at the adherens junctions of epithelial cells in the villus, nuclear β‐catenin was detected in ISCs co‐immunostained with the ISC marker OLFM4 (van der Flier *et al*, 2009) at the crypt base after I/R injury (Shoshkes‐Carmel *et al*, 2018; Fig 2I). However, nuclear localization of β‐catenin was impaired (Fig 2I and J), and the numbers of OLFM4^+^ ISCs (Fig 2I and K) and the cells expressing the Wnt target cyclin D1 (CCND1; Shoshkes‐Carmel *et al*, 2018; Fig 2L and M) were significantly reduced in the crypts of EC‐*Foxc*‐DKO mice 24 and 48 h after I/R injury. These data suggest that the loss of EC‐specific *Foxc1/c2* expression impedes I/R‐induced Wnt signaling in ISCs.

### Defective mucosal recovery in LEC‐specific *Foxc* mutant mice after intestinal I/R injury

**Figure 3:**
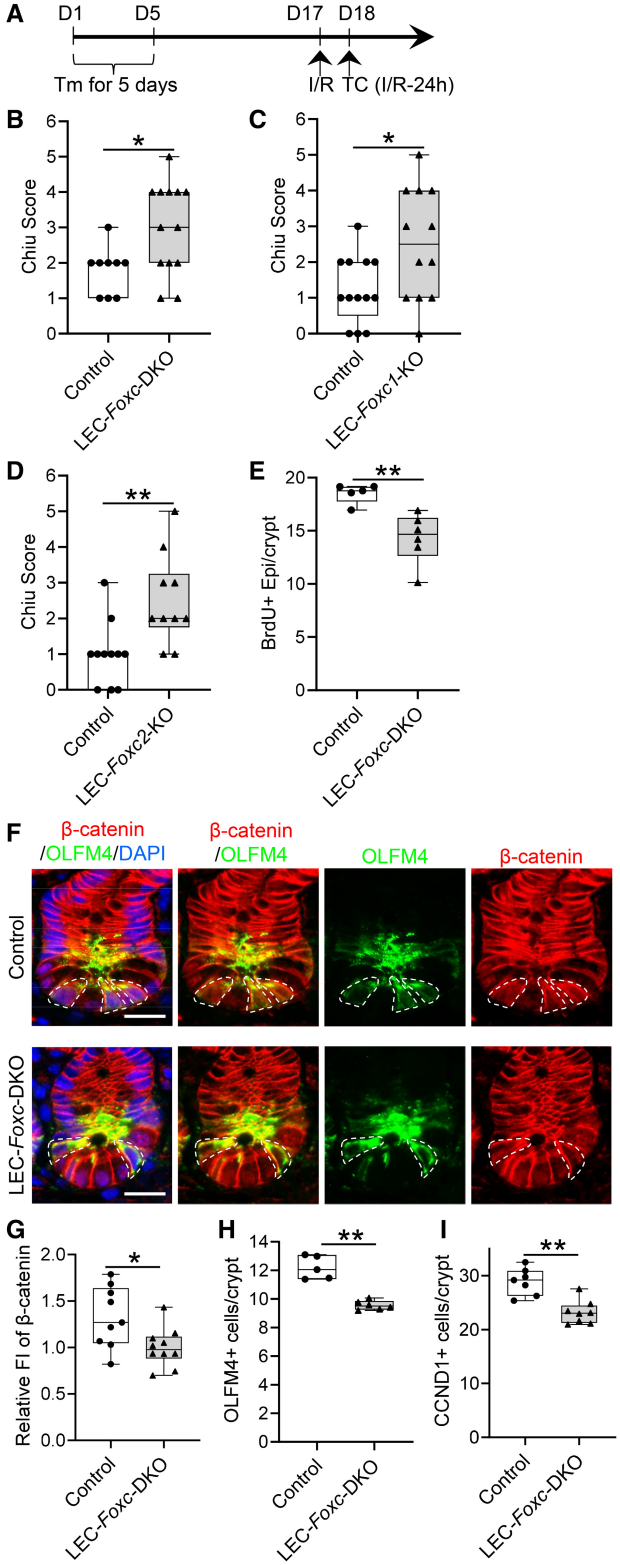
Characterization of defects in the intestinal mucosa in mice with LEC‐specific deletion of *Foxc1/2* after I/R injury ASchematic showing the time of tamoxifen (Tm) injection, I/R surgery, and tissue collection (TC) for the LEC‐specific *Foxc* mutant mice and their control.B–DChiu Score analysis from H&E stained distal jejunum 24 h after I/R for control (*Foxc1* ^*f/f*^ *;Foxc2* ^*f/f*^) and LEC‐*Foxc*‐DKO (*Vegfr3‐Cre* ^*ERT2*^ *;Foxc1* ^*f/f*^ *;Foxc2* ^*f/f*^) mice (B), control (*Foxc1* ^*f/f*^) and LEC‐*Foxc1*‐KO (*Vegfr3‐Cre* ^*ERT2*^ *;Foxc1* ^*f/f*^) mice (C), and control (*Foxc2* ^*f/f*^) and LEC‐*Foxc2*‐KO (*Vegfr3‐Cre* ^*ERT2*^ *;Foxc2* ^*f/f*^) mice (D) based on Fig EV3E–G. Data are box‐and‐whisker plots, Mann–Whitney *U* test, each symbol represents one mouse, *N* = 9 ~ 13, *P < 0.05, **P < 0.01.EQuantification of the number of BrdU^+^ epithelial cells per crypt in control and LEC‐*Foxc*‐DKO mice at 24 h after I/R based on Fig EV3H. Data are box‐and‐whisker plots, Mann–Whitney *U* test, each symbol represents one mouse, *N* = 5 ~ 6, **P < 0.01.FRepresentative images of crypts immunostained with OLFM4 and β‐catenin in control and LEC‐*Foxc*‐DKO mice 24 h after I/R. The accumulation of β‐catenin in the nuclei of ISCs (dotted circles) was found in control mice but inhibited in LEC‐Foxc‐DKO mice. Paraffin sections (4 μm), scale bars = 20 μm.G, H(G) Quantification of relative fluorescent intensity (FI) of β‐catenin immunostaining within ISC and (H) quantification of the number of OLFM4^+^ ISCs were performed based on Fig 3F. Data are box‐and‐whisker plots, Mann–Whitney *U* test, each symbol represents one mouse, *N* = 9 ~ 10 in Fig 3G, *N* = 5 ~ 6 in Fig 3H, *P < 0.05.IQuantification of the number of CCND1^+^ epithelial cells per crypt at I/R‐24 h based on the immunostaining of CCND1 as shown in Fig EV3I. Data are box‐and‐whisker plots, Mann–Whitney *U* test, each symbol represents one mouse, *N* = 7 ~ 8, **P < 0.01. Data information: The box‐and‐whisker plots in (B–E) and (G–I) display the median value (central band in the box), second and third quartiles (bottom and top ends of the box, respectively), as well as minimum/maximum values (whiskers blow/above the box) of the data sets. Source data are available online for this figure.

As tamoxifen‐induced Cre recombination in *Cdh5‐Cre* ^*ERT2*^ mice occurs in both BECs and LECs, we generated mice carrying a tamoxifen‐inducible, LEC‐specific, compound homozygous *Foxc1* ^−/−^;*Foxc2* ^−/−^ mutation (referred to herein as LEC‐*Foxc*‐DKO mice) by breeding conditional‐null *Foxc1* ^*fl/fl*^ and *Foxc2* ^*fl/fl*^ mice (Sasman *et al*, 2012) with LEC‐specific *Vegfr3‐Cre* ^*ERT2*^ mice (Martinez‐Corral *et al*, 2016) to investigate LEC‐specific functions of *Foxc1* and *Foxc2* following intestinal I/R injury. We first confirmed *Vegfr3‐Cre*‐mediated recombination limited to intestinal LECs of adult LEC‐*Foxc*‐DKO mice crossed with the Rosa26mTmG reporter mice (Fig EV3D). The LEC‐*Foxc*‐DKO mice and their littermate control mice were subjected to the sham or I/R injury procedures (Fig 3A), and LEC‐specific deletion of *Foxc1* and *Foxc2* significantly increased the severity of intestinal mucosa injury 24 h after I/R (Figs 3B and EV3E). Furthermore, increased intestinal damage was also noted in single LEC‐*Foxc1*‐KO and LEC‐*Foxc2*‐KO mice after I/R injury (Fig 3C and D, and EV3F and G), suggesting that both FOXC1 and FOXC2 are required in intestinal LECs for intestinal repair in response to I/R injury. In addition, proliferating (BrdU^+^) intestinal epithelial cells (Fig EV3H) were quantified in the crypts of LEC‐*Foxc*‐DKO mice 24 h after I/R injury, and the LEC‐specific loss of *Foxc1/c2* reduced the proliferative response of intestinal epithelial cells to I/R injury (Fig 3E). Consistent with the increased severity of intestinal mucosa defects in the LEC‐*Foxc*‐DKO mice (Fig 3B), the activation of Wnt signaling (i.e., nuclear localization of β‐catenin, Fig 3F and G) in ISCs, the numbers of OLFM4^+^ ISCs (Fig 3F and H) and Cyclin D1^+^ (CCND1^+^) cells (Figs EV3I and 3I) per crypt after intestinal I/R injury were all reduced in these mutant mice compared with the control mice.

### Defective vascular recovery in EC‐ and LEC‐specific deletions of *Foxc1/c2* after intestinal I/R injury

**Figure 4:**
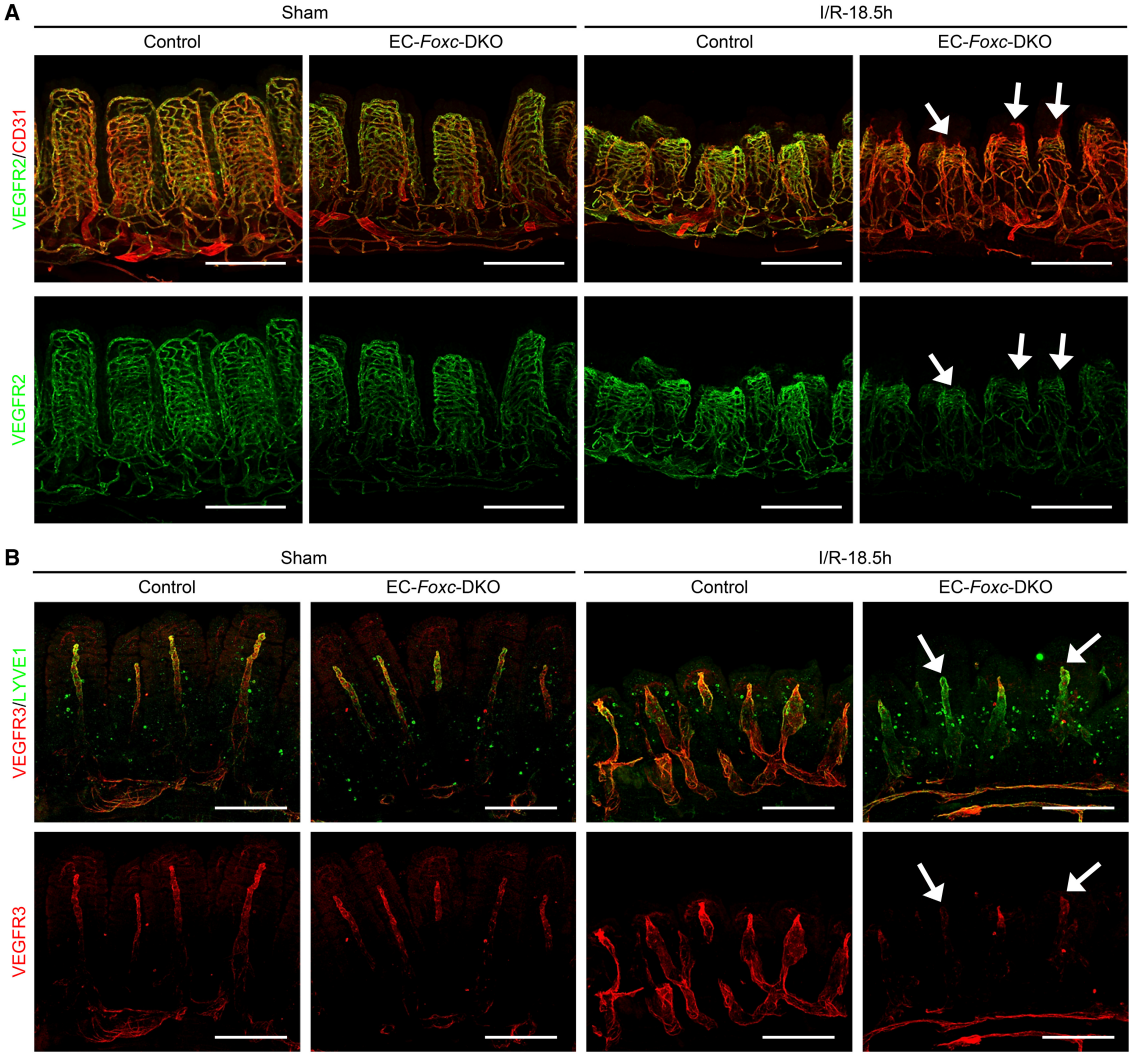
Defects in vascular regeneration after intestinal I/R injury in EC‐*Foxc*‐DKO mice Representative images of intestinal whole‐mount VEGFR2/CD31 (green/red) immunostaining show increased VEGFR2 (green) expression at the angiogenic front of villous blood capillaries in control mice at I/R‐18.5 h. In EC‐*Foxc*‐DKO mice, the increase of VEGFR2 is inhibited in villous blood vessels and the blood vasculatures are damaged (arrow). Scale bars = 200 μm.Representative images of intestinal whole‐mount VEGFR3/LYVE1 (red/green) immunostaining. 18.5 h after I/R, VEGFR3 (red) is increased in lacteals especially at the lacteal tips in the control but is inhibited in the EC‐*Foxc*‐DKO lacteals (arrow). Scale bars = 200 μm. Source data are available online for this figure.

**Figure 5:**
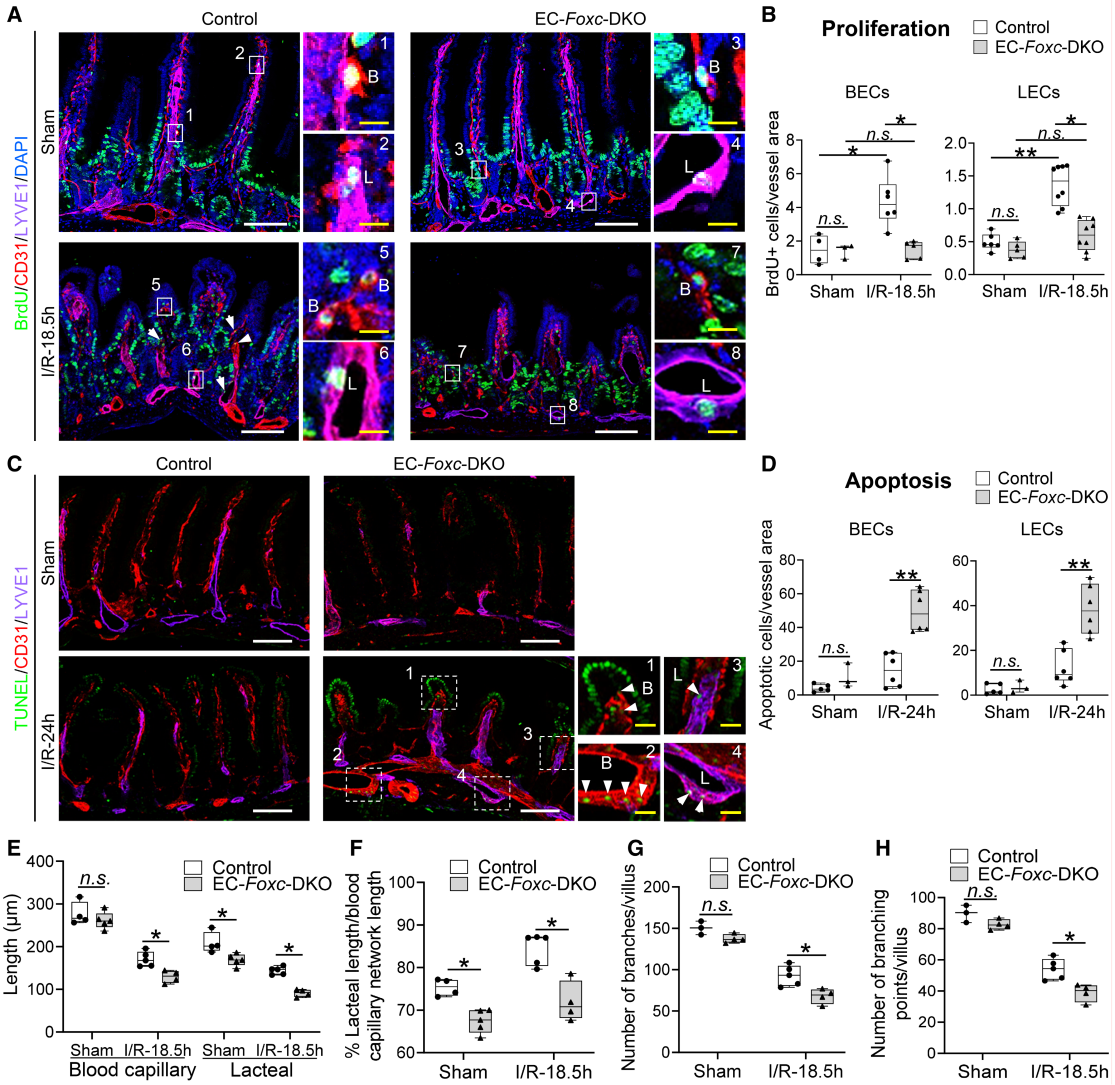
Impaired vascular regeneration after intestinal I/R injury in EC‐*Foxc*‐DKO mice ARepresentative BrdU/CD31/LYVE1/DAPI immunostaining images of intestinal paraffin sections (15 μm) from mice injected with BrdU 18.5 h before euthanasia for the analysis of proliferative BECs (B in 1, 3, 5, 7) and LECs (L in 2, 4, 6, 8) in intestines. Arrow heads show BrdU^+^ BECs or LECs. White/yellow bars = 100 or 10 μm, respectively.BThe numbers of BrdU^+^ BECs and LECs per 0.1 mm^2^ blood vessel (CD31^+^LYVE1^−^) and lymphatic vessel (CD31^+^LYVE1^+^) area in intestinal mucosa were quantified respectively based on Fig 5A. Data are box‐and‐whisker plots, Kruskal–Wallis one‐way ANOVA test, each symbol represents one mouse, *N* = 3 ~ 8, *P < 0.05, **P < 0.01, *n.s*., not significant.CRepresentative TUNEL/CD31/LYVE1/DAPI immunostaining images of distal jejunums for the analysis of apoptotic BECs/LECs in intestinal paraffin sections (15 μm). High‐magnification images are from the dotted line boxes (1 ~ 4). Arrow heads show the apoptotic BECs (B in 1, 2) and LECs (L in 3, 4) at villus (1, 3) or submucosa (2, 4). White/yellow scale bars = 100 or 20 μm, respectively.D–H(D) The numbers of apoptotic BECs and LECs per 0.1 mm^2^ blood vessel (CD31^+^LYVE1^−^) and lymphatic vessel (CD31^+^LYVE1^+^) area in intestinal mucosa were quantified respectively based on Fig 5C. Data are box‐and‐whisker plots, Mann–Whitney *U* test, each symbol represents one mouse, *N* = 3 ~ 6, **P < 0.01, *n.s*., not significant. The length of blood capillary vasculature and lacteals were measured (E) based on Fig EV4A and B. The percentage (%) of the lacteal length/blood capillary network length was then calculated (F). The numbers of branches (G) and the branching points (H) of the villous blood vasculatures were counted based on the whole‐mount staining of VEGFR2 (Fig 4A) as previously described (Bernier‐Latmani & Petrova, 2016) using ImageJ software. Data are box‐and‐whisker plots, Mann–Whitney *U* test, each symbol represents one mouse, *N* = 3 ~ 5, *P < 0.05, *n.s*., not significant. Data information: The box‐and‐whisker plots in (B), (D), and (E–H) display the median value (central band in the box), second and third quartiles (bottom and top ends of the box, respectively), as well as minimum/maximum values (whiskers blow/above the box) of the data sets. Source data are available online for this figure.

**Figure EV4:**
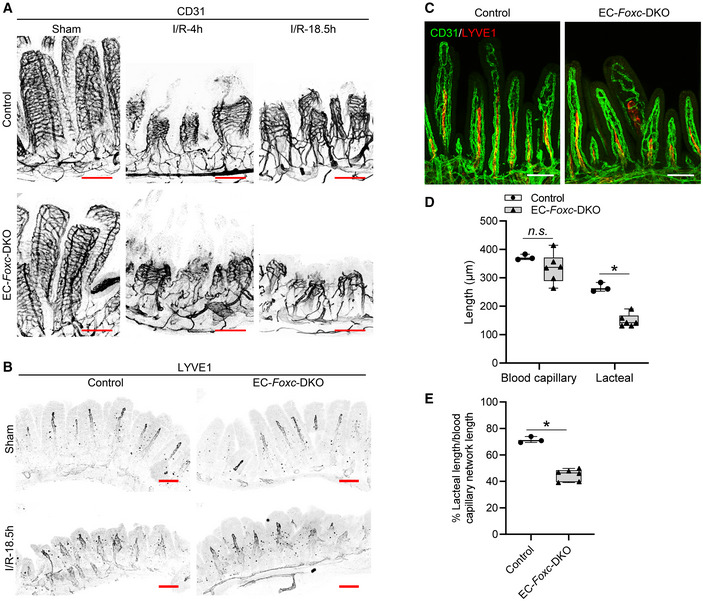
EC‐iKO of *Foxc1/c2* results in severe damage of blood and lymphatic vasculatures in intestinal villi after I/R A, BRepresentative images of whole‐mount distal jejuna stained with CD31 (A) and LYVE1 (B) show the damage of blood (A) and lymphatic vasculatures (B) in control and EC‐*Foxc*‐DKO villi at 4 h and/or 18.5 h after I/R. Scale bars = 100 μm.C–E(C) Representative whole‐mount proximal jejuna immunostained with CD31 (green) and LYVE1 (red) in neonatal mice treated with Tm from P1 to P5 and euthanized at P7. Proximal jejuna were collected from neonatal mice due to the ease of operation and similar lacteal length/blood capillary network length ratio between proximal and distal jejuna. The length of blood capillary vasculature and lacteals were measured (D) based on Fig EV4C. The percentage (%) of the lacteal length/blood capillary network length was then calculated (E). Data are box‐and‐whisker plots, Mann–Whitney *U* test, each symbol represents one mouse, *N* = 3 ~ 6, *P < 0.05, *n.s*., not significant. The box‐and‐whisker plots display the median value (central band in the box), second and third quartiles (bottom and top ends of the box, respectively), as well as minimum/maximum values (whiskers blow/above the box) of the data sets. Source data are available online for this figure.

The vascular recovery of villus BECs and LECs after I/R injury proceeds via a stepwise process in which blood capillaries (BECs) regrow earlier than lacteals (LECs) in the villous stroma (Meng *et al*, 2007). We found that the EC‐*Foxc*‐DKO mutation was associated with the defective vascular repair of intestinal BECs and LECs after I/R injury (Fig 4A and B). Vascular endothelial growth factor (VEGF) receptor (R) 2 (VEGFR2) and VEGFR3 were highly expressed in the growing tips of villous BECs and LECs, respectively, in control mice after intestinal I/R injury but were severely diminished in EC‐*Foxc*‐DKO mice (arrows in Fig 4). Notably, the proliferation of intestinal BECs and LECs was also significantly reduced (Fig 5A and B), while the number of apoptotic BECs and LECs was significantly increased (Fig 5C and D), in EC‐*Foxc*‐DKO mice than in their control mice after I/R injury. Following whole‐mount immunostaining for CD31 and LYVE1 (Fig EV4A and B, and Movies EV1 and EV2), the length of blood capillaries and lacteals (Fig 5E) as well as the percentage of lacteal length to blood capillary length (Fig 5F) were measured. The regrowth of both blood capillaries and lacteals in the villi was significantly decreased in EC‐*Foxc*‐DKO mice after intestinal I/R injury, whereas EC‐*Foxc*‐double mutant lacteals were shorter than controls in sham treatments. Similarly, shorter lacteals were found in the intestines of EC‐*Foxc*‐DKO neonatal mice at P7 after Tm treatment from P1 to P5 (Fig EV4C–E), suggesting that *Foxc1/c2* are required for the maintenance of lacteal length. Interestingly, no significant difference in the lacteal permeability between control and EC‐*Foxc*‐DKO mice was found by the BODIPY C16 uptake experiment (Appendix Fig S1A–H). After I/R injury, whole‐mount immunostaining of intestinal VEGFR2 (Fig 4A) and subsequent quantification revealed a reduction in branches (Fig 5G) and branching points (Fig 5H) of blood capillaries in EC‐*Foxc*‐DKO mice. Together, these results suggest that EC‐*Foxc1/c2* expression contributes to the repair of ischemic intestinal mucosa by promoting the recovery of BECs and LECs.

### Reduced *Rspo3* and *Cxcl12* expression in intestinal LECs and BECs of EC‐*Foxc*‐DKO mice, respectively, after I/R injury

**Figure 6:**
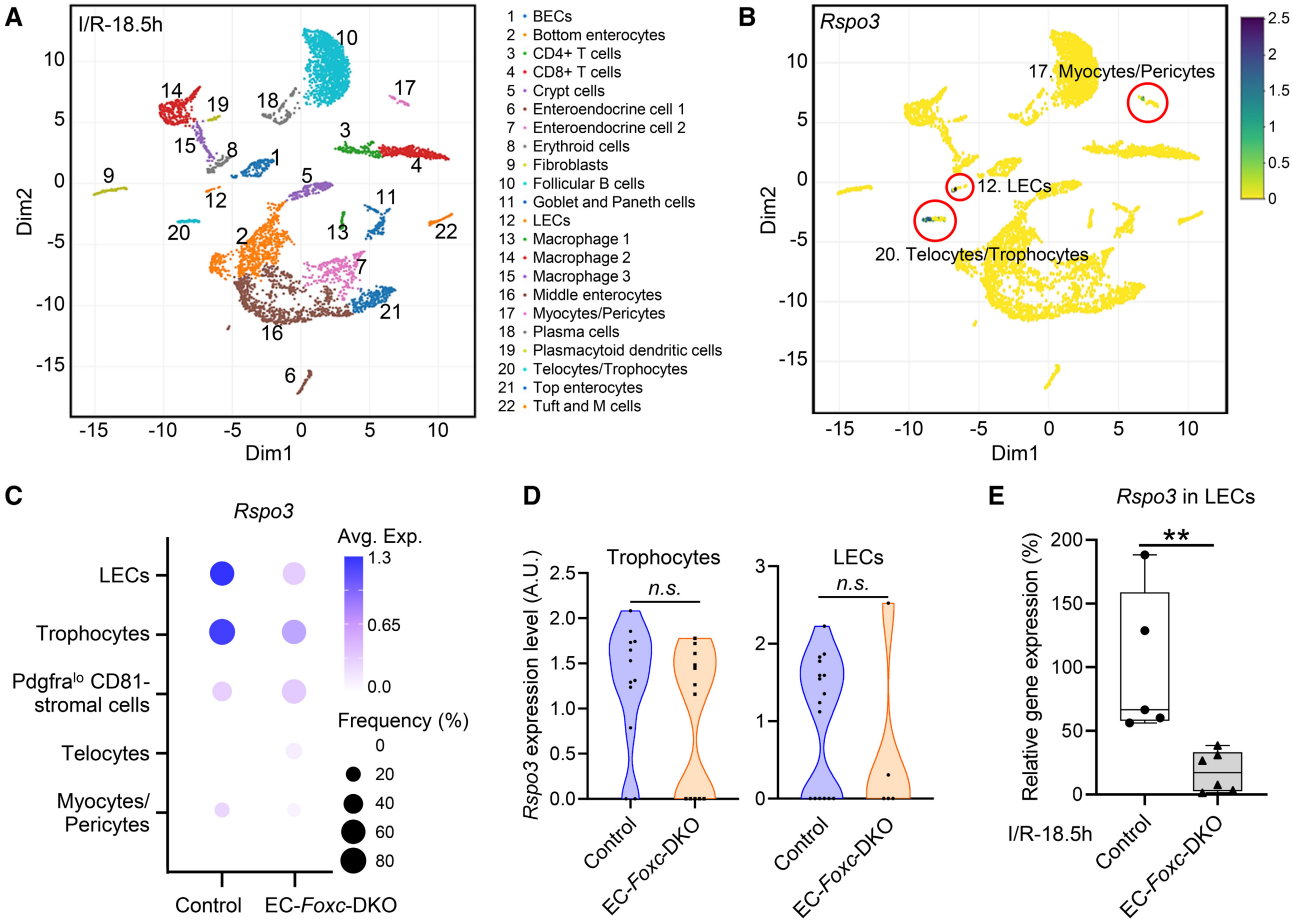
Intestinal *Rspo3* expression in control and EC‐*Foxc*‐DKO mice after I/R identified by single‐cell RNA sequencing Visualization of unsupervised clustering of 22 distinct clusters by UMAP from the distal jejunum of both control and EC‐*Foxc*‐DKO mice after I/R at 18.5 h.UMAP visualization of *Rspo3* expression in three different cell clusters identified in Fig 6A.Dot plot showing relative expression of *Rspo3* in five cell clusters identified by scRNA‐seq. Fill colors represent normalized mean expression levels and circle sizes represent the within‐cluster frequency of positive gene detection.Violin plots of the *Rspo3* expression in trophocytes and LECs in the intestine at I/R‐18.5 h. Mann–Whitney *U* test, each symbol represents one cell; *N* = 12 and 12 in control and EC‐*Foxc*‐DKO trophocytes, respectively; *N* = 16 and 5 in control and EC‐*Foxc*‐DKO LECs.Validation study by qPCR for the detection of relative mRNA expression of *Rspo3* in the sorted intestinal LECs at I/R‐18.5 h. Data are box‐and‐whisker plots, Mann–Whitney *U* test, each symbol represents one mouse, *N* = 5 ~ 6, **P < 0.01. The box‐and‐whisker plots display the median value (central band in the box), second and third quartiles (bottom and top ends of the box, respectively), as well as minimum/maximum values (whiskers blow/above the box) of the data sets. Source data are available online for this figure.

**Figure EV5:**
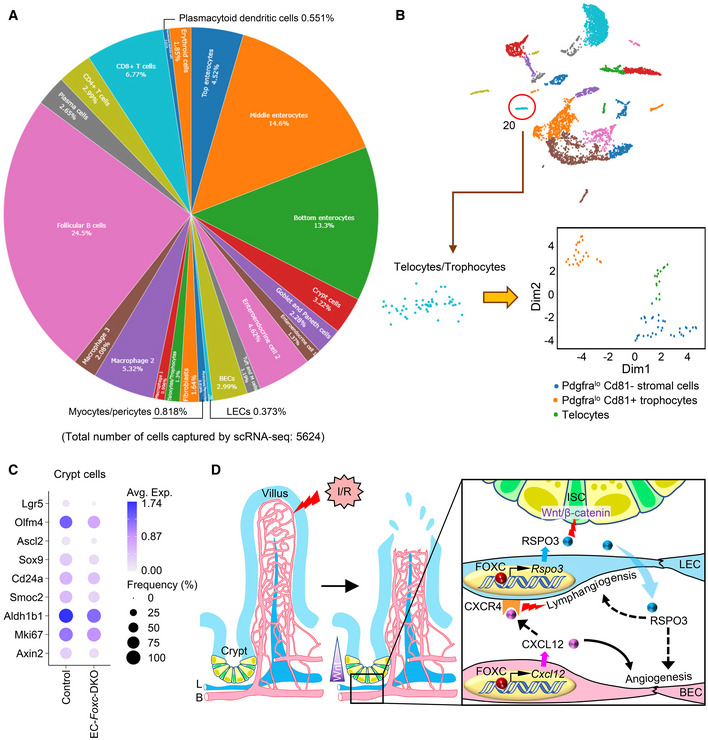
ScRNA‐seq analysis on the small intestines from control and EC‐*Foxc*‐DKO mice 18.5 h after I/R Pie chart showing the percentage of each cell cluster identified in Fig 6A of total cell population.Sub‐clustering performed on the cluster 20 (Telocytes/Trophocytes).Dot plot showing relative expression of different genes identified by scRNA‐seq were decreased in crypt cell cluster in EC‐*Foxc*‐DKO mice compared with control mice at I/R‐18.5 h. Fill colors represent normalized mean expression levels and circle sizes represent the within‐cluster frequency of positive gene detection. *Lgr5*, *Olfm4*, *Ascl2*, *Sox9*, *Cd24a*, *Smoc2* and *Aldh1b1* are ISC markers. *Mik67* is a proliferative marker. *Ascl2*, *Sox9* and *Axin2* are Wnt/β‐catenin target genes.Schematic drawing of the mechanism by which endothelial FOXC1 and FOXC2 promote mouse intestinal regeneration after I/R injury. The intestinal mucosa is damaged after I/R injury. FOXC (both FOXC1 and FOXC2) regulate the expression of *Rspo3/Cxcl12* through binding to their regulatory elements in LECs/BECs of the lymphatic/blood vessels near the crypts, respectively. RSPO3 secreted by LECs (blue arrow) is an agonist of the canonical Wnt/β‐catenin signaling pathway and promotes the intestinal epithelial regeneration and repair. RSPO3 derived from LECs also promotes angiogenesis and lymphangiogenesis (dashed arrows). BEC‐derived CXCL12 (pink arrow) not only regulates angiogenesis (arrow) but also stimulates CXCR4 on LECs to enhance lymphangiogenesis (dashed arrow). L, lymphatic vessel; B, blood vessel; ISC, intestinal stem cell; LEC, lymphatic endothelial cell; BEC, blood endothelial cell. Source data are available online for this figure.

To investigate molecular mechanisms associated with impaired intestinal regeneration in EC‐*Foxc*‐DKO mutants following I/R, we performed single‐cell RNA sequencing (scRNA‐seq) analyses of distal jejuna from control and EC‐*Foxc*‐DKO mice 18.5 h after I/R injury. Dimensionality reduction and clustering analysis identified 22 transcriptionally distinct cell clusters (Figs 6A and EV5A) based on known gene markers for each specific cell type (Table 1), including BECs, LECs, stromal cells, epithelial cells, and other cell types. When interpreted according to the recent classification of stromal cell populations in the ISC niche (McCarthy *et al*, 2020a), the results from our scRNA‐seq experiments indicate that *Rspo3* is mainly expressed in two clusters (LECs and Telocytes/Trophocytes) after intestinal I/R injury (Fig 6B, cluster 12 and 20, respectively). Low expression of *Rspo3* was found in Myocytes/Pericytes cluster (Fig 6B, cluster 17). Further sub‐clustering performed on Telocytes/Trophocytes cluster based on their known markers (McCarthy *et al*, 2020b) identified three cell clusters, Trophocytes, Pdgfra^lo^ Cd81‐stromal cells, and Telocytes (Fig EV5B). A dot plot used for visualizing differential gene expression (mean expression level) and gene expression frequency in different cell clusters (Fig 6C) showed that *Rspo3* was decreased in both LECs and Trophocyte clusters in EC‐*Foxc*‐DKO intestines compared with controls after I/R injury. In Trophocyte cluster, the moderately decreased trend of *Rspo3* was not significant (Fig 6D). Similar *Rspo3* levels were found in the other three cell clusters between the two groups (Fig 6C). Since the percentage of the LECs captured by scRNA‐seq was very low (0.373%, Fig EV5A), the number of LECs obtained and used for analysis was very limited, and the decrease of *Rspo3* expression in LECs was not significant (Fig 6D). However, by validation study, the mRNA expression level of *Rspo3* was lower in the sorted intestinal LECs of EC‐*Foxc*‐DKO mice than in the control mice 24 h after I/R injury (Fig 6E). Taken together, these findings indicate that EC‐*Foxc1/c2* deletion results in reduced RSPO3 expression in intestinal LECs after I/R injury.

**Table 1:** Top 10 marker genes used for the identification of specific cell clusters in scRNA‐seq data analysis.

Cluster	Marker	Cluster	Marker	Cluster	Marker	Cluster	Marker
BECs	Fabp4	Enteroendocrine cell 2	Rbp2	Macrophage 1	S100a9	Plasmacytoid dendritic cells	Bst2
	Ly6c1		S100a6		Cxcl2		Siglech
	Igfbp7		Lgals3		Il1b		Ccl4
	Plvap		Tm4sf20		S100a8		Rnase6
	Ly6a		Serpinb6a		G0s2		Mpeg1
	Ptprb		Anxa2		Srgn		Lsp1
	Flt1		Sprr2a3		Tyrobp		Ly6c2
	Pecam1		Gsta1		Cebpb		Cybb
	Cd36		Fam162a		Cd14		St8sia4
	Epas1		Pmp22		Fcer1g		Ccr9
Bottom enterocytes	Reg3b	Erythroid cells	Hba‐a1	Macrophage 2	Lyz2	Telocytes/Trophocytes	Dcn
	Fabp1		Hbb‐b1		Ccl6		Col3a1
	Reg3g		Hba‐a2		Ccl2		Gsn
	Plac8		Alas2		Ccl9		Col1a2
	Sis		Ube2l6		Mafb		Col1a1
	Mgst1		Snca		Ctsb		Mgp
	Maoa		Fech		Psap		Sfrp1
	Arg2		Cyb561d1		Ctsc		Lum
	Aldh1a1		Irgc1		Lgmn		Bgn
	Gstm3		Nudt15		Ms4a6c		Serping1
CD4^+^ T cells	Emb	Fibroblasts	Apoe	Macrophage 3	C1qa	Top Enterocytes	Apoa4
	Lat		Scn7a		C1qb		Ada
	Tcf7		Sparc		C1qc		Apob
	Ms4a4b		Apod		Acp5		Apoa1
	Itgb7		Fxyd1		Apol7c		Clca4a
	Cd69		Prnp		Il22ra2		Apoc3
	Lef1		Cryab		Dnase1l3		2010109I03Rik
	Bcl11b		Plp1		Pla2g2d		Selenop
	Cd27		Chl1		Batf3		Nt5e
	Arl4c		Pmepa1		Tctex1d2		Dnpep
CD8^+^ T cells	Ccl5	Follicular B cells	Cd74	Middle Enterocytes	Fabp2	Tuft and M cells	Krt18
	Gzma		H2‐Ab1		Guca2b		Cd24a
	Cd7		Ly6d		Spink1		Adh1
	Cd3g		Vpreb3		Anpep		Hck
	Rgs1		Bank1		Aldob		Sh2d6
	Nkg7		Cd83		Clca4b		Lrmp
	Gzmb		Pou2f2		Smim24		Selenom
	AW112010		Gpr183		Slc5a1		Tm4sf4
	Cd8a		H2‐DMb2		Leap2		Dclk1
	Cd3e		Fcmr		Guca2a		Avil
Crypt cells	Krt19	Goblet and Paneth cells	Zg16	Myocytes/Pericytes	Acta2		
	Dmbt1		Tff3		Tagln		
	Gpx2		Fcgbp		Myh11		
	Hmgb2		Spink4		Sparcl1		
	H2afz		Clca1		Tpm1		
	Birc5		Lgals2		Tpm2		
	Ube2c		Agr2		Cald1		
	Cks2		Lypd8		Myl9		
	Pclaf		Gm1123		Rgs5		
	Ccdc34		Ido1		Flna		
Enteroendocrine cell 1	Sct	LECs	Mmrn1	Plasma cells	Jchain		
	Chgb		Ccl21a		Mzb1		
	Chga		Timp3		Pou2af1		
	Neurod1		Lyve1		Nap1l1		
	Cpe		Cavin2		Sec11c		
	Krt7		Reln		Dut		
	Fxyd3		Aqp1		Eaf2		
	Reg4		Flt4		Mef2b		
	Tph1		Fgl2		H2afx		
	Pcsk1		Tshz2		Top2a		

**Figure 7:**
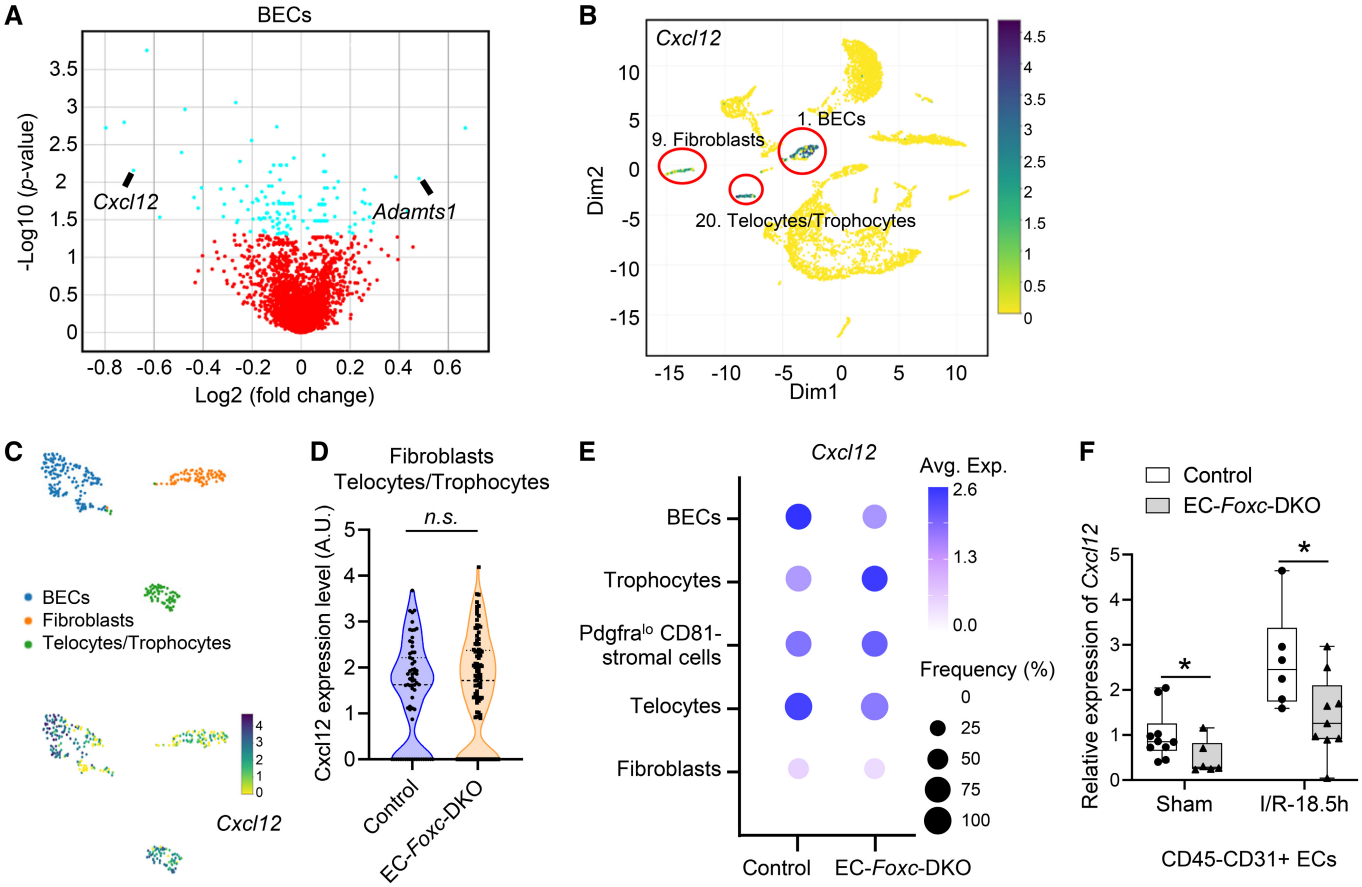
Intestinal *Cxcl12* expression in control and EC‐*Foxc*‐DKO mice after I/R identified by single‐cell RNA sequencing Volcano plots of differential expression analysis of BECs in control and EC‐*Foxc*‐DKO mice at I/R‐18.5 h using the MAST model. Blue dots denote genes with significant differential expression, *P* < 0.05.UMAP visualization of *Cxcl12* expression in three clusters identified in Fig 6A.Larger UMAP visualization of three *Cxcl12* expressing cell clusters (upper panel) and their *Cxcl12* expression (lower panel).Violin plots of the *Cxcl12* expression in fibroblasts and telocytes/trophocytes. Mann–Whitney *U* test, each symbol represents one cell, *N* = 67 and 98 in control and EC‐*Foxc*‐DKO mice, respectively; *n.s*., not significant.The cluster of Telocytes/trophocytes was further subclustered into three clusters: trophocytes, pdgfra^lo^ CD81^−^ stromal cells, and telocytes as shown in Fig EV5B. Dot plot showing relative expression of *Cxcl12* in five cell clusters identified by scRNA‐seq.Relative mRNA expression of *Cxcl12* in Dynabeads‐isolated ECs (CD45^−^CD31^+^) from distal jejunum. Data are box‐and‐whisker plots, Mann–Whitney *U* test, each symbol represents one mouse, *N* = 6 ~ 10, *P < 0.05. The box‐and‐whisker plots display the median value (central band in the box), second and third quartiles (bottom and top ends of the box, respectively), as well as minimum/maximum values (whiskers blow/above the box) of the data sets. Source data are available online for this figure.

The results from our scRNA‐seq experiments also identified numerous genes that were differentially expressed in intestinal BECs from EC‐*Foxc*‐DKO and control mice after I/R injury (Fig 7A), including the antiangiogenic factor *Adamts1* (Lee *et al*, 2006; Obika *et al*, 2012) and the CXC chemokine *Cxcl12*, which were up‐ and downregulated, respectively, in EC‐*Foxc*‐DKO BECs. Notably, although *Cxcl12* was also expressed in both Telocyte/Trophocyte and Fibroblast clusters (Fig 7B and C), no significant difference was found in the *Cxcl12* level in these two clusters (Fig 7D). *Cxcl12* was significantly downregulated only in BECs (Fig 7A and E), which had a larger cell population than the other two cell clusters (Figs 7C and EV5A). Furthermore, qPCR analysis validated that *Cxcl12* mRNA expression was significantly reduced in isolated intestinal CD45^−^/CD31^+^ ECs of both sham‐ and I/R‐treated EC‐*Foxc*‐DKO mice compared with their littermate controls (Fig 7F). These results suggest that *Foxc1/c2*‐deficient intestinal ECs fail to increase *Cxcl12* expression after I/R injury.

### FOXC1/C2 bind to regulatory elements containing FOXC‐binding sites located in the *RSPO3* and *CXCL12* loci in LECs and BECs, respectively

**Figure 8:**
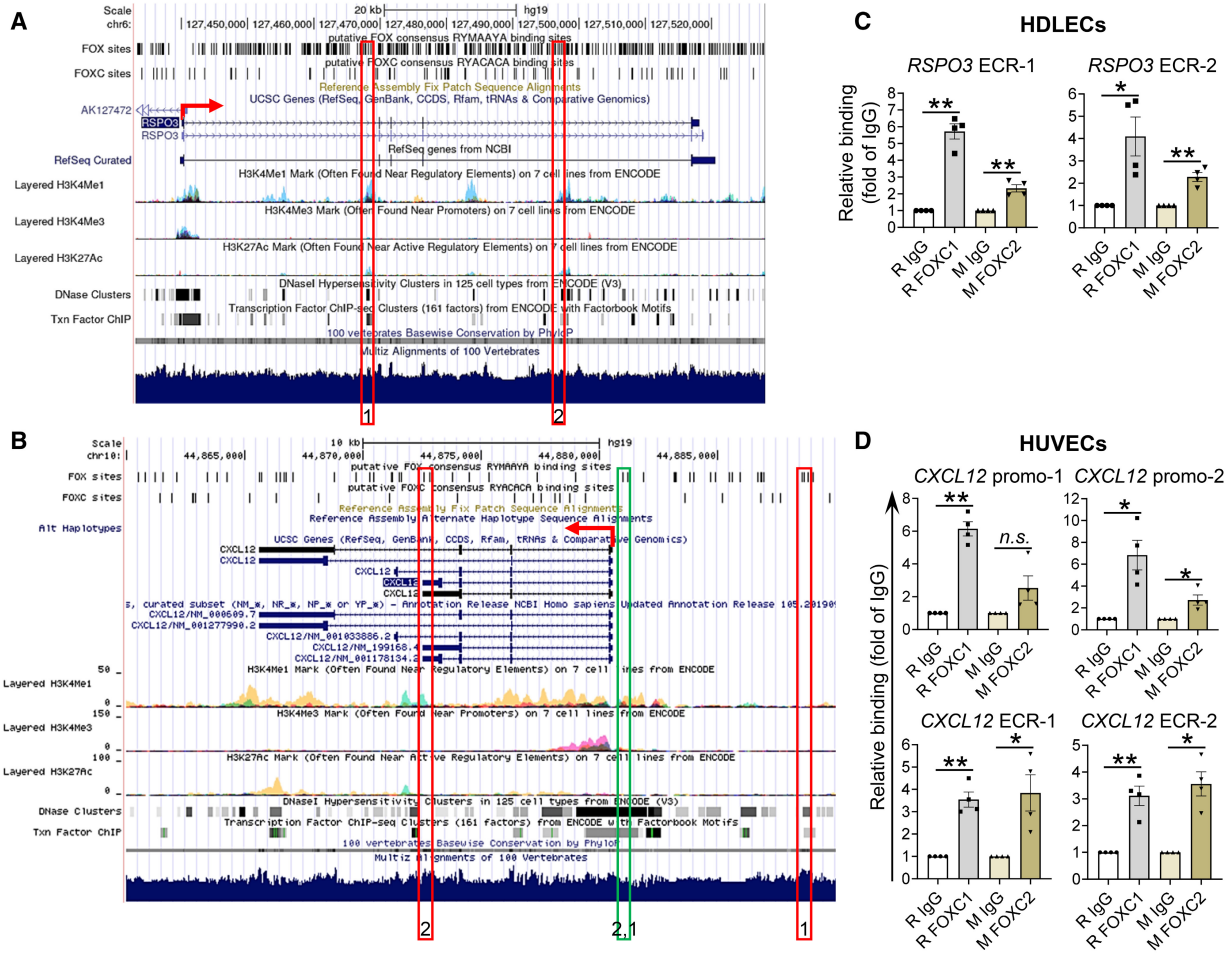
FOXC1 and FOXC2 regulate the expression of *RSPO3* in LECs and *CXCL12* in BECs through binding to their regulatory elements A–D(A and B) *In silico* identification of putative FOX‐binding sites in the *RSPO3* and *CXCL12* loci. Putative FOX‐binding sites in regions of the human *RSPO3* (A) and *CXCL12* (B) loci as viewed on the UCSC genome browser (https://genome.ucsc.edu; Kent *et al*, 2002). Vertical lines on the “FOX sites” and “FOXC sites” tracks indicate putative binding sites corresponding to the FOX “RYMAAYA” consensus sequence and the FOXC “RYACACA” consensus sequence (Chen *et al*, 2019) predicted using the Hypergeometric Optimization of Motif EnRichment (HOMER) suite of tools (Heinz *et al*, 2010). Red boxes indicate evolutionary conserved regions (ECRs) containing FOX‐binding sites between human and mouse genomes that are conserved and aligned as identified using the ECR browser tool (ecrbrowser.docde.org). Green boxes indicate promoter regions in *CXCL12* containing FOX‐binding sites according to the prediction of JASPAR. The red arrows indicate the site of transcription initiation for *RSPO3* or *CXCL12*. (C and D) FOXC1 and FOXC2 co‐occupy the ECRs of *RSPO3* in HDLECs (C), as well as the ECRs and promoters of *CXCL12* in HUVECs (D). Quantitative‐ChIP assay was performed using rabbit (R) anti‐FOXC1 and mouse (M) anti‐FOXC2 antibodies to analyze the recruitment of FOXC on promoters and/or ECRs in HDLECs and HUVECs respectively. Values were quantified against IgG controls. Data are mean ± SEM, paired *t*‐test, each symbol represents data collected from one experiment, *N* = 4, *P < 0.05, **P < 0.01, *n.s*., not significant. Source data are available online for this figure.

To determine whether FOXC1 and FOXC2 are capable of regulating the transcription of *RSPO3* and *CXCL12*, we first identified putative FOXC‐binding sites (RYMAAYA or RYACACA; Overdier *et al*, 1994; Pierrou *et al*, 1994; Kaufmann & Knochel, 1996) in the vicinity of the human *RSPO3* and *CXCL12* loci (Fig 8A and B, and Materials and Methods) by analyzing data deposited in the ENCODE database (Kent *et al*, 2002) with HOMER software (Heinz *et al*, 2010), as recently described in our analysis of the *PRICKLE1* locus (Norden *et al*, 2020). These sites are conserved between human and mouse, and contain histone‐methylated and ‐acetylated regions (e.g., H3K4Me1 and H3K27Ac ChIP peaks), DNaseI hypersensitive regions, and transcriptionally active regions (identified by Transcription Factor ChIP‐seq data). Putative FOXC‐binding sites in the human *CXCL12* promoter region were also identified according to the prediction of JASPAR (Fig 8B). We first confirmed the mRNA expression of *FOXC1* and *FOXC2* in human dermal lymphatic endothelial cells (HDLECs) and human umbilical vein endothelial cells (HUVECs; Appendix Fig S2A and B). We then performed chromatin immunoprecipitation (ChIP) assays with FOXC1 and FOXC2 antibodies and cell lysates from HDLECs (for testing the FOXC‐binding sites in the *RSPO3* locus, Fig 8A) and from HUVECs (for testing the FOXC‐binding sites in the *CXCL12* locus, Fig 8B). Quantitative analysis following ChIP showed that FOXC bindings were significantly enriched in ECRs of RSPO3 (Fig 8C), as well as in ECRs and promoter regions of CXCL12 (Fig 8D). Accordingly, these results indicate that FOXC1 and FOXC2 regulate the expression of *RSPO3* in LECs and *CXCL12* in BECs through binding to their regulatory elements.

### RSPO3 treatment rescues the defective repair of small intestines in EC‐ and LEC‐*Foxc*‐DKO mice after I/R injury

**Figure 9:**
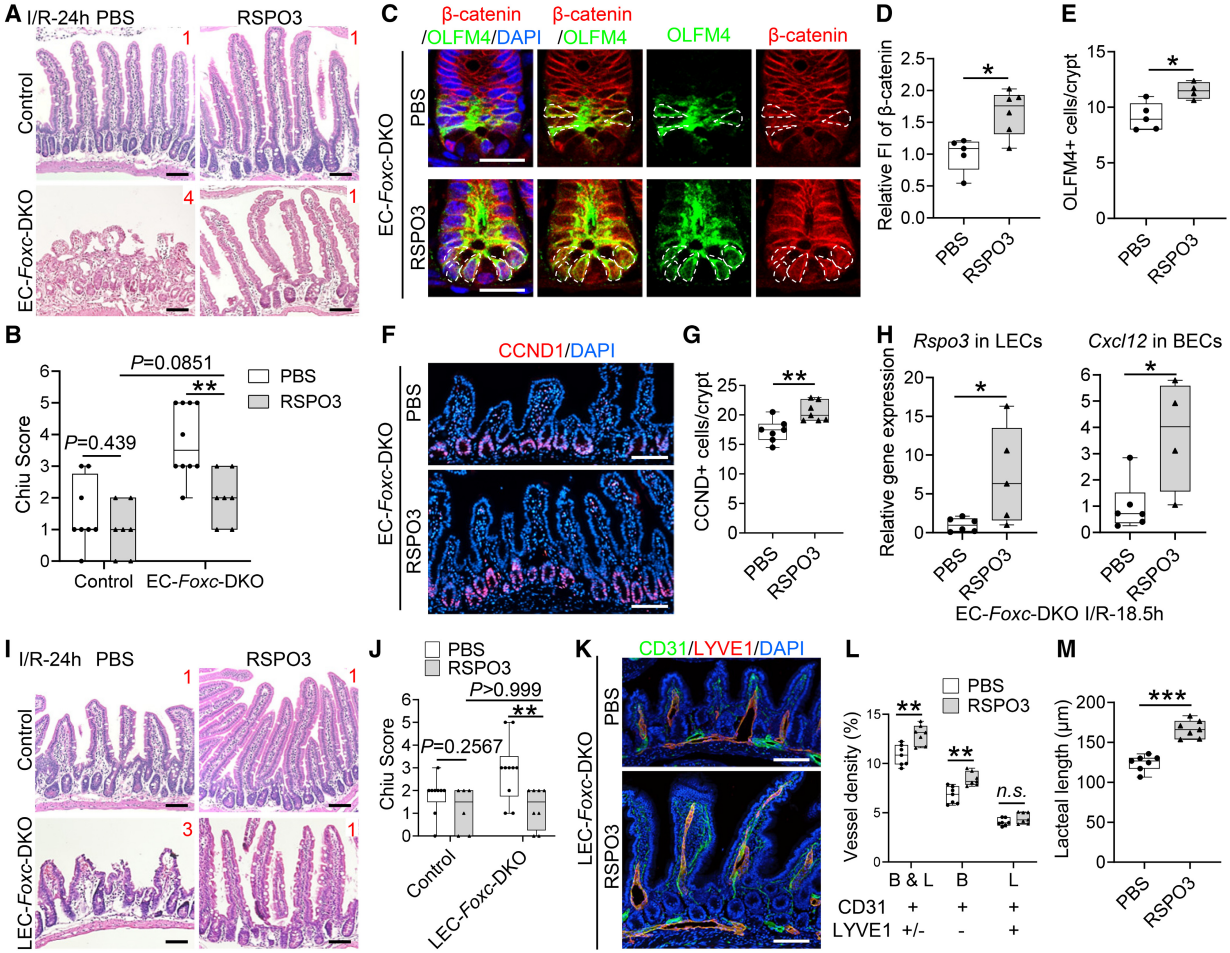
RSPO3 partially rescues impaired regeneration of intestinal mucosa in EC‐*Foxc*‐DKO and LEC‐*Foxc*‐DKO mice after I/R In RSPO3 rescue experiment, each mouse was treated with 5 μg RSPO3 in 100 μl PBS by retro‐orbital injection 30 min before ischemia. PBS‐treated mice were used as vehicle control.
A–J(A and I) Representative images of H&E staining show the rescue effects of RSPO3 in intestinal mucosa in EC‐*Foxc*‐DKO (A) and LEC‐*Foxc*‐DKO (I) mice, as well as their control mice 24 h after I/R. Red numbers indicate the Chiu scores. Scale bars = 100 μm. (B and J) Quantification of Chiu Score for the intestines at I/R‐24 h is based on H&E staining as shown in Fig 9A and I. Data are box‐and‐whisker plots, Mann–Whitney *U* test, each symbol represents one mouse, *N* = 6 ~ 13, **P < 0.01. (C) Representative images of crypts immunostained with OLFM4 and β‐catenin in PBS/RSPO3 treated EC‐*Foxc*‐DKO mice 24 h after I/R. The accumulation of β‐catenin in the nuclei of ISCs (dotted circles) was found in RSPO3‐rescued mice. Paraffin sections (4 μm), scale bars = 20 μm. (D) Quantification of relative fluorescent intensity (FI) of β‐catenin immunostaining within ISC and (E) quantification of the number of OLFM4^+^ ISCs were performed based on Fig 9C. Data are box‐and‐whisker plots, Mann–Whitney *U* test, each symbol represents one mouse, *N* = 4 ~ 6, *P < 0.05. (F) Representative images of immunostaining of CCND1 in intestines in PBS/RSPO3 treated EC‐Foxc‐DKO mice at I/R‐24 h. Scale bars = 100 μm. (G) Quantification of the number of CCND1^+^ epithelial cells per crypt at I/R‐24 h is based on Fig 9F. Data are box‐and‐whisker plots, Mann–Whitney *U* test, each symbol represents one mouse, *N* = 7, **P < 0.01. (H) Relative mRNA expression of *Rspo3* in sorted LECs and *Cxcl12* in sorted BECs from intestines of PBS/RSPO3 treated EC‐*Foxc‐*DKO mice at I/R‐18.5 h. Data are box‐and‐whisker plots, Mann–Whitney *U* test, each symbol represents one mouse, *N* = 4 ~ 6, *P < 0.05.KRepresentative images of CD31/LYVE1 immunostaining in the intestines of PBS/RSPO3 treated LEC‐*Foxc*‐DKO mice at I/R‐24 h. Scale bars = 100 μm.L, MQuantification of the vessel density (L) (= vessel area/total intestinal tissue area × 100%) for the blood (B) and/or lymphatic (L) vessels (markers listed below the graph were used to identify B and L), as well as the measurement of lacteal length (M) was performed based on Fig 9K. The Data are box‐and‐whisker plots, Mann–Whitney *U* test, each symbol represents one mouse, *N* = 7, **P < 0.01, *n.s*., not significant. Data information: The box‐and‐whisker plots in (B), (D), (E), (G), (H), (J), (L) and (M) display the median value (central band in the box), second and third quartiles (bottom and top ends of the box, respectively), as well as minimum/maximum values (whiskers blow/above the box) of the data sets. Source data are available online for this figure.

RSPO3 is a key regulator of Wnt signaling during intestinal regeneration (Kannan *et al*, 2013; Storm *et al*, 2016), and RSPO3 prevents I/R‐induced intestinal tissue damage and vascular leakage (Kannan *et al*, 2013). Accordingly, we investigated whether RSPO3 treatment can rescue the defective repair of small intestines in EC‐*Foxc*‐DKO mice following I/R injury. Adult control and EC‐*Foxc*‐DKO mice were treated with PBS or RSPO3 (5 μg in 100 μl PBS per mouse) by retro‐orbital injection 30 min before intestinal ischemia. Quantification of Chiu scores was then performed 24 h after I/R injury. In control mice, the rescue of intestinal mucosal damage was not significant after RSPO3 treatment at I/R‐24 h. However, in EC‐*Foxc*‐DKO mice, RSPO3 treatment could partially rescue the mucosal damage (Fig 9A and B), activate the Wnt signaling (i.e., nuclear localization of β‐catenin, Fig 9C and D) in ISCs, and increase the numbers of OLFM4^+^ ISCs (Fig 9C and E) and CCND1^+^ cells (Fig 9F and G) per crypt after intestinal I/R injury. Moreover, RSPO3 treatment significantly increased the mRNA expression level of *Rspo3* in sorted LECs and *Cxcl12* in sorted BECs in EC‐Foxc‐DKO mouse intestine after I/R (Fig 9H). Together, RSPO3 treatment rescues the intestinal mucosal damage and defects in BECs and LECs in EC‐*Foxc*‐DKO mice after I/R.

Given that RSPO3 is expressed in LECs of the small intestine, but not in BECs (Fig 6B), equivalent experiments were subsequently performed with adult mice carrying LEC‐specific mutations of both *Foxc* genes. Similar rescue effects of the RSPO3 treatment were observed in LEC‐*Foxc*‐DKO intestines 24 h after I/R injury with the improvement of the Chiu score to the level similar to I/R‐exposed control mice (i.e., grade 1; Fig 9I and J). RSPO3 treatment also rescued the vascular damage by increasing the blood vessel density and the lacteal length (Fig 9K–M) in LEC‐*Foxc*‐DKO mice after I/R. Thus, these results show that RSPO3 regulates angiogenesis and lymphangiogenesis in a paracrine and autocrine manner, respectively.

Immunoglobulin M (IgM) and complement also contribute to intestinal I/R injury (Kannan *et al*, 2013), but RSPO3 treatment suppresses the deposition of IgM and complement in damaged intestinal tissue (Kannan *et al*, 2013), likely by enhancing the activation of Wnt signaling in ISCs of the small intestine. Therefore, IgM deposition was evaluated in damaged intestines of control and EC‐*Foxc*‐DKO mice 24 h after I/R injury via immunostaining (Appendix Fig S3A). Compared with control mice, IgM deposition was increased in EC‐*Foxc*‐DKO mice, whereas administration of RSPO3 reduced the level of IgM in EC‐*Fox*‐DKO mice. The levels of IgM deposition were further quantified via western blotting as described previously (Yoshiya *et al*, 2011; Kannan *et al*, 2013). I/R‐enhanced IgM levels in the intestinal tissues were greater in EC‐*Foxc*‐DKO mice compared with controls (Appendix Fig S3B and C). More importantly, RSPO3 treatment significantly reduced the levels of IgM in the EC‐*Foxc*‐DKO mice after I/R injury (Appendix Fig S3D and E). Collectively, these observations demonstrate that *Foxc1* and *Foxc2* expression in the intestinal vasculature contributes to intestinal mucosal recovery and regeneration after I/R injury.

### CXCL12 treatment partially rescues the defective repair of small intestines in EC‐*Foxc*‐DKO mice after I/R injury

**Figure 10:**
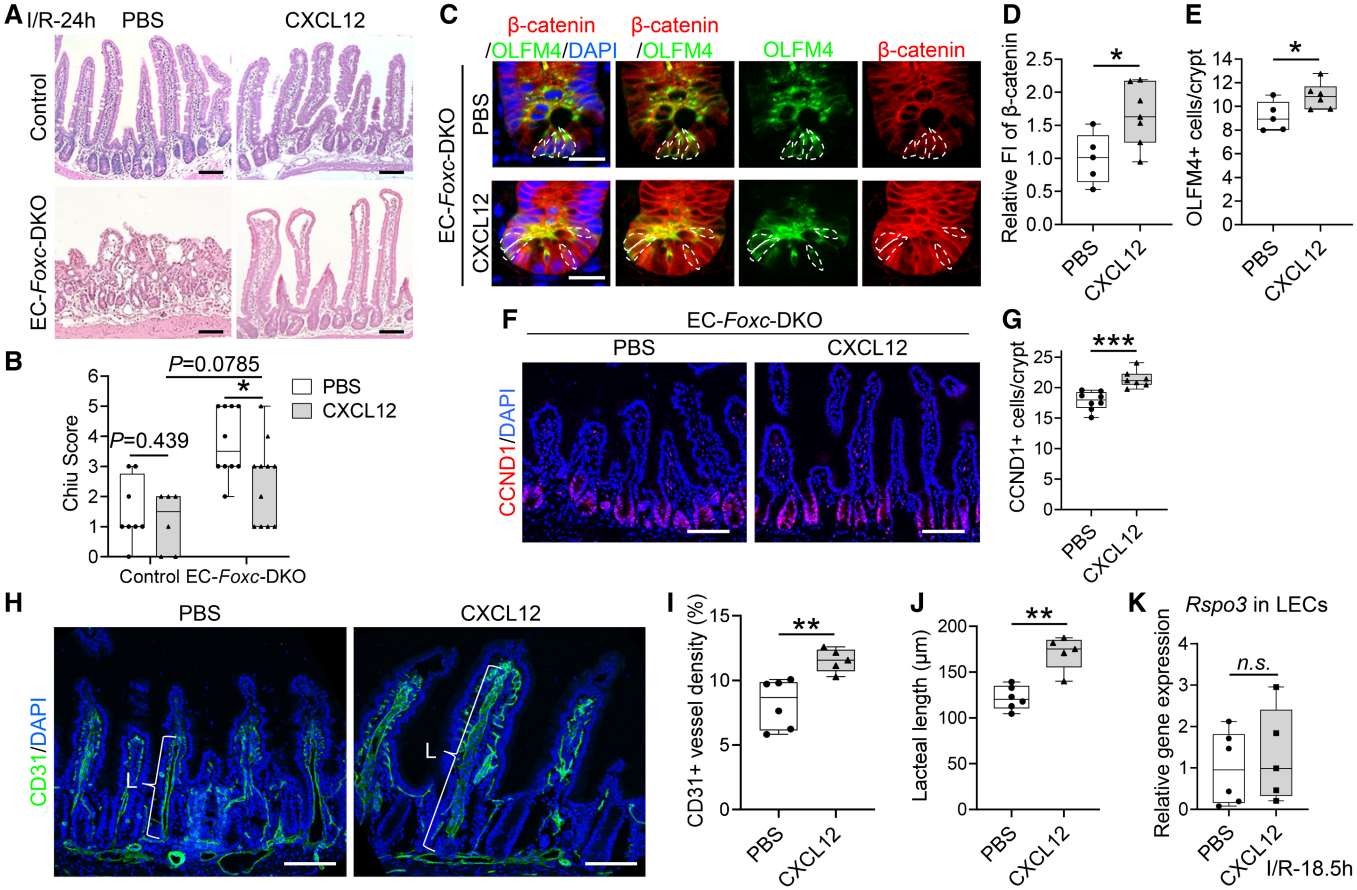
CXCL12 partially rescues impaired regeneration of intestinal mucosa in EC‐*Foxc*‐DKO mice after I/R In CXCL12 rescue experiments, mice were treated with 50 μg/kg CXCL12 in PBS by retro‐orbital injection 30 min before ischemia. Mice treated with PBS were used as control.
ARepresentative images of H&E staining show the rescue effects of CXCL12 in intestinal mucosa in control and EC‐*Foxc*‐DKO mice 24 h after I/R. Red numbers indicate Chiu scores. Scale bars = 100 μm.BQuantification of Chiu Scores for mouse intestines 24 h after I/R based on H&E staining as shown in Fig 10A. Data are box‐and‐whisker plots, Mann–Whitney *U* test, each symbol represents one mouse, *N* = 6 ~ 11, *P < 0.05.CRepresentative images of crypts immunostained with OLFM4 and β‐catenin in PBS‐ and CXCL12‐treated EC‐*Foxc*‐DKO mice 24 h after I/R. The total protein signal of β‐catenin is upregulated in CXCL12‐treated crypts compared with the PBS‐treated group. The accumulation of β‐catenin in the nuclei of ISCs (dotted circles) was found in CXCL12‐treated mice but inhibited in PBS‐treated mice. Paraffin sections (4 μm), scale bars = 20 μm.D, E(D) Quantification of relative fluorescent intensity (FI) of β‐catenin immunostaining within ISC and (E) quantification of the number of OLFM4^+^ ISCs per crypt were performed based on Fig 10C. Data are box‐and‐whisker plots, Mann–Whitney *U* test, each symbol represents one mouse, *N* = 5 ~ 7, *P < 0.05.F, G(F) Representative immunostaining images of CCND1 and (G) Quantification of CCND1^+^ epithelial cells per crypt after I/R at 24 h. Scale bars = 100 μm. Data are box‐and‐whisker plots, Mann–Whitney *U* test, each symbol represents one mouse, *N* = 7 ~ 8, ***P < 0.001.HRepresentative confocal images of CD31 immunostaining of distal jejuna in PBS‐ and CXCL12‐ treated EC‐*Foxc*‐DKO mice after I/R at 24 h. Paraffin sections (15 μm), scale bars = 100 μm. L represents the lacteal length measured in Fig 10J.I, J(I) Quantification of CD31^+^ vessel density (% = total CD31^+^ vessel area/total intestinal tissue area × 100%) and (J) quantification of lacteal length were performed based on Fig 10H. Data are box‐and‐whisker plots, Mann–Whitney *U* test, each symbol represents one mouse, *N* = 4 ~ 6, **P < 0.01.KRelative mRNA expression of *Rspo3* in sorted intestinal LECs from PBS/CXCL12‐treated EC‐*Foxc‐*DKO mice at I/R‐18.5 h. Data are box‐and‐whisker plots, Mann–Whitney *U* test, each symbol represents one mouse, *N* = 5 ~ 6, *n.s*., not significant. Data information: The box‐and‐whisker plots in (B), (D), (E), (G) and (I–K) display the median value (central band in the box), second and third quartiles (bottom and top ends of the box, respectively), as well as minimum/maximum values (whiskers blow/above the box) of the data sets. Source data are available online for this figure.

*Cxcl12* was only downregulated in intestinal BECs but not in the Telocyte/Trophocyte and Fibroblast clusters of EC‐*Foxc*‐DKO mutant intestine after I/R injury (Fig 7A, D and E). Given evidence that hypoxia upregulates CXCL12 (Hitchon *et al*, 2002; Santiago *et al*, 2011) as a key regulator of angiogenesis (Santagata *et al*, 2021), we examined whether pretreatment with CXCL12 can rescue the defects in the vascular regrowth and intestinal repair associated with EC‐*Foxc1/c2* deficiencies. Adult control and EC‐*Foxc*‐DKO mice were treated with PBS or CXCL12 via retro‐orbital injection as described previously (Garnica *et al*, 2005), and I/R induction was performed 30 min later. The degree of intestinal mucosal damage 24 h after I/R injury was then quantified via the Chiu scoring system. CXCL12 treatment didn't significantly rescue the mucosal damage in control mice, but partially rescued the defective repair of small intestines in the EC‐*Foxc*‐DKO mice (Fig 10A and B). Moreover, nuclear localization of β‐catenin was observed in crypt ISCs of CXCL12‐pretreated EC‐*Foxc*‐DKO mice after I/R injury compared with the PBS treatment (Fig 10C and D). The numbers of OLFM4^+^ ISCs (Fig 10C and E) and cells expressing the Wnt target CCND1 (Shoshkes‐Carmel *et al*, 2018; Fig 10F and G) were significantly increased in the crypts of EC‐*Foxc*‐DKO mice treated with CXCL12 after I/R injury. Administration of CXCL12 also improved the defective vascular recovery (CD31^+^ vessel density), as well as the lacteal length of EC‐*Foxc*‐DKO mice 24 h after I/R injury (Fig 10H–J). However, CXCL12 treatment did not affect the mRNA expression level of *Rspo3* in sorted LECs from EC‐*Foxc*‐DKO mice at I/R‐18.5 h (Fig 10K). Together, CXCL12 treatment rescues intestinal repair associated with EC‐*Foxc1/c2* deficiencies by enhancing vascular recovery (angiogenesis and lymphangiogenesis) and stimulating Wnt signaling in the crypts of the intestinal epithelium.

## Discussion

Accumulating evidence indicates the importance of BEC/LEC‐derived paracrine factors in tissue regeneration. In the present study, we demonstrate that EC‐*Foxc1/Foxc2* expression is crucial for the repair of the intestinal mucosa, BECs, and LECs after I/R injury and that the EC‐ and LEC‐*Foxc*‐DKO mutations in mice impair canonical Wnt/β‐catenin signaling in ISCs at the crypt base. Furthermore, our scRNA‐seq data indicate that RSPO3 expression is attenuated in LECs and stromal cells of the EC‐*Foxc*‐DKO mice after intestinal I/R injury, which is at least partially attributable to impairments in intestinal regeneration because ISC activity appears to be crucially dependent on Wnt/β‐catenin signaling in the subepithelial cellular microenvironment in the ISC niche (Santos *et al*, 2018; Hageman *et al*, 2020). We also show that CXCL12 is reduced in intestinal BECs of the EC‐*Foxc*‐DKO mice after I/R injury. Importantly, we show that FOXC1 and FOXC2 are key transcription factors for the paracrine factors *RSPO3* and *CXCL12* via binding to their regulatory elements in LECs and BECs, respectively. Most significantly, treatment with RSPO3 and CXCL12 rescues the defective repair of small intestines in the LEC‐*Foxc*‐DKO and EC‐*Foxc*‐DKO mice, respectively. Consequently, this study elucidates the novel molecular and cellular mechanisms that mediate the role of EC‐specific FOXC1/C2 in the repair of the intestinal mucosa and vasculature, regulation of paracrine RSPO3 and CXCL12 factors, and activation of ISCs after intestinal I/R injury.

Adult single and compound EC‐ (or LEC)‐specific *Foxc1/c2‐*mutant mice provide us with the *first* opportunity to comprehensively characterize how the two *Foxc* genes cooperatively regulate the postischemic repair of intestinal blood/lymphatic vessels after I/R injury and intestinal epithelial regeneration by modulating Wnt/RSPO3 and CXCL12 signaling. Because the EC‐*Foxc*‐DKO and LEC‐*Foxc*‐DKO mutations are induced by the different drivers (i.e., *Cdh5‐Cre* ^*ERT2*^ and *Vegfr3‐Cre* ^*ERT2*^), the extent of *Foxc* downregulation in the EC‐ and LEC‐specific mutant mouse lines may not be equal. However, the degree of impairments in intestinal repair is consistently greater in the EC‐*Foxc*‐DKO mutant line than in the LEC‐*Foxc*‐DKO mutant line, suggesting that *Foxc1* and *Foxc2* are required in both BECs and LECs for intestinal tissue repair. Equivalent experiments were also performed with adult mice carrying EC‐ (and LEC‐) specific KO mutations of each individual *Foxc* gene to determine the similarities and differences between the phenotypes associated with each deletion. While our qPCR and scRNA‐seq analyses show that expression levels of *Foxc1* in intestinal ECs are higher than those of *Foxc2*, the phenotypic differences between EC‐ (or LEC‐) *Foxc1*‐KO and EC‐ (or LEC‐) *Foxc2*‐KO mice are not particularly distinct. Although the reason(s) for the phenotypic similarities remains unclear, recent evidence indicates that *Foxc2* is essential for the maintenance of intestinal LECs and that treatment with antibiotics to deplete gut microbiota rescues the phenotype of LEC‐specific *Foxc2* mutants, including lymphatic dilation in the small intestine (Gonzalez‐Loyola *et al*, 2021). Bacterial translocation occurs when the intestinal barrier is disrupted by I/R injury, and I/R‐induced intestinal damage can be attenuated by pretreatment of an antibiotic cocktail to deplete gut commensal bacteria (Yoshiya *et al*, 2011). It remains to be elucidated whether commensal bacteria contribute to aberrant intestinal recovery and regeneration in our EC/LEC‐specific *Foxc* mutant mice. TNF‐α and IL‐6 regulate the proliferation of intestinal epithelial cells (Kuhn *et al*, 2014; Ruder *et al*, 2019). However, EC‐*Foxc1/c2* deletions result in reduced proliferation of epithelial cells in crypts, and it may be attributable to decreased EC expression of R‐spondin3 and CXCL12 in the mutant mice.

The EC‐specific mutations of both *Foxc* genes in mice impair the regulation of RSPO3 in LECs (Fig 6), accompanied by reduced expression of ISC markers and Wnt target genes in crypt cells (Fig EV5C), and that RSPO3 treatment rescues the defective repair of small intestines in both EC‐*Foxc*‐DKO and LEC‐*Foxc*‐DKO mice after I/R injury (Fig 9). These observations are consistent with previous reports that RSPO3 prevents I/R‐induced intestinal tissue damage (Kannan *et al*, 2013) and that RSPO3 is a key regulator of Wnt signaling during intestinal regeneration (Kannan *et al*, 2013; Storm *et al*, 2016). While intestinal RSPO3 is known to be produced by LECs (Ogasawara *et al*, 2018; McCarthy *et al*, 2020a), our results indicate that FOXC1 and FOXC2 regulate *RSPO3* expression in LECs by binding to its regulatory elements (Fig 8), and pretreatment with RSPO3 almost completely rescues the impaired intestinal repair in the LEC‐*Foxc*‐DKO mice (Fig 9), implicating a paracrine effect of LEC‐mediated RSPO3 signaling on the activation of ISCs after I/R injury (Fig EV5D). Importantly, recent studies demonstrate that RSPO3 produced by intestinal LECs is required for maintaining ISCs in homeostasis and regeneration (Goto *et al*, 2022; Niec *et al*, 2022; Palikuqi *et al*, 2022) and reinforce our finding that FOXC transcription factors mediate LEC‐derived RSPO3 as a critical paracrine regulator in intestinal regeneration after I/R injury. Since it has been shown that the RSPO receptor Lgr5 is expressed in both intestinal BECs and LECs (Kalucka *et al*, 2020; Appendix Fig S4A–F), our results (Fig 9K–M) further indicate that RSPO3 promotes the regrowth of blood and lymphatic vessels after I/R injury. Given the relevance of other injury models that do not damage the relatively quiescent endothelium but elicit RSPO3 expression from the vasculature such as irradiation, cytotoxic damage via 5‐fluorouracil (FU), or dextran sodium sulfate (DSS)‐induced inflammatory response (Goto *et al*, 2022; Palikuqi *et al*, 2022), further investigation is warranted. However, in a similar way to our observation that EC‐*Foxc1/c2* deficiency does not impair intestinal epithelium homeostasis, LEC‐specific *Rspo3* mutant mice exhibit the normal formation of the small intestine without injury (Goto *et al*, 2022; Palikuqi *et al*, 2022).

The chemokine CXCL12 is an angiocrine factor that is secreted from BECs and regulates organ‐specific tissue repair, regeneration, and homeostasis, including the liver, bone marrow, and lung (Rafii *et al*, 2016; Ribatti *et al*, 2021; Yi *et al*, 2021). However, the function of BEC‐derived CXCL12 paracrine/autocrine signaling in damaged organs after injury remains largely unknown. The results from our study demonstrate that CXCL12 expression is downregulated in the intestinal BEC cluster and intestinal CD45^−^/CD31^+^ ECs of the EC‐*Foxc*‐DKO mice after I/R injury (Fig 7), and that FOXC1 and FOXC2 bind to the FOXC‐binding sites in the regulatory elements of the *CXCL12* locus in cultured BECs (Fig 8). Furthermore, CXCL12 treatment partially rescues the defective repair of small intestines in EC‐*Foxc*‐DKO mice after I/R injury (Fig 10). Our results are in accord with recent evidence that FOXC1 controls CXCL12 expression in CXCL12‐abundant reticular (CAR) progenitors and Schwann cells (Omatsu *et al*, 2014; Xia *et al*, 2020). EC‐derived CXCL12 promotes angiogenesis via an *autocrine* mechanism (Santagata *et al*, 2021), and CXCL12‐CXCR4 signaling cooperates with VEGF to enhance angiogenic processes such as the morphogenesis and sprouting of vascular tubes (Stratman *et al*, 2011). Since VEGFR2 levels at the angiogenic front of growing blood capillaries in the villus are lower in EC‐*Foxc*‐DKO mice than in control mice after intestinal I/R injury (Fig 4A), it is likely that intestinal EC‐derived CXCL12 autocrine signaling is defective in EC‐*Foxc*‐DKO mice. Consistently, impaired blood vascular recovery of the EC‐*Foxc*‐DKO mice after intestinal I/R injury is rescued by CXCL12 treatment (Fig 10H and I). Since the vascular regrowth of villous BECs and LECs after I/R injury proceeds via a stepwise, interactive process, and regeneration of villus blood vessels precedes the reconstruction of lacteals (Meng *et al*, 2007) (Fig 4 and Movies EV1 and EV2), the severity of mucosal damage is greater in the EC‐*Foxc*‐DKO mice than in the LEC‐*Foxc*‐DKO mice. Moreover, consistent with the role of the CXCL12/CXCR4 pathway in lymphangiogenesis (Zhuo *et al*, 2012), impaired lacteal length of the EC‐*Foxc*‐DKO mice after intestinal I/R injury is rescued by CXCL12 treatment (Fig 10H and J). Since CXCL12 treatment activates Wnt signaling in the ISC of the EC‐*Foxc*‐DKO mice (Fig 10), it is plausible that FOXC1/C2‐mediated, endothelial‐derived CXCL12 signaling enhances vascular regrowth in intestinal ischemia, thereby recovering the damaged ISC niche for intestinal regeneration (Fig EV5D).

In summary, our study demonstrates that BEC/LEC‐FOXC1/FOXC2 expression regulates the repair of the intestinal vasculature and mucosal damage during intestinal regeneration after I/R injury by controlling the CXCL12 and RSPO3 signaling pathways. Thus, FOXC1 and FOXC2 regulate blood/lymphatic vascular function in the small intestine, as well as vascular‐mediated signaling to ISCs and the ISC niche during the intestinal repair. Collectively, this study provides new insights into fundamental processes that are critically involved in recovery from ischemic disease and injury and may have important implications for the treatment of other ischemic conditions that are associated with impairments in tissue regeneration and stem cell activity, including cardiovascular disease.

## Materials and Methods

### Animal husbandry

*Foxc1* ^*fl/fl*^, *Foxc2* ^*fl/fl*^, *Foxc1* ^*fl/fl*^ *;Foxc2* ^*fl/fl*^ (Sasman *et al*, 2012), *Cdh5‐Cre* ^*ERT2*^ (Sorensen *et al*, 2009), *Vegfr3‐Cre* ^*ERT2*^ (Martinez‐Corral *et al*, 2016), and *Foxc2‐Cre* ^*ERT2*^ (Amin *et al*, 2017) mice were used. EC‐specific or LEC‐specific *Foxc1*, *Foxc2*, and compound *Foxc1;Foxc2*‐mutant mice were generated by crossing *Foxc*‐floxed females (*Foxc1* ^*fl/fl*^, *Foxc2* ^*fl/fl*^, and *Foxc1* ^*fl/fl*^ *;Foxc2* ^*fl/fl*^) with *Cdh5‐Cre* ^*ERT2*^ *;Foxc1* ^*fl/fl*^ (EC‐*Foxc1*‐KO), *Cdh5‐Cre* ^*ERT2*^ *;Foxc2* ^*fl/fl*^ (EC‐*Foxc2*‐KO), *Cdh5‐Cre* ^*ERT2*^ *;Foxc1* ^*fl/fl*^ *;Foxc2* ^*fl/fl*^ (EC‐*Foxc*‐DKO), *Vegfr3‐Cre* ^*ERT2*^ *;Foxc1* ^*fl/fl*^ (LEC‐*Foxc1*‐KO), *Vegfr3‐Cre* ^*ERT2*^ *;Foxc2* ^*fl/fl*^ (LEC‐*Foxc2*‐KO), *Vegfr3‐Cre* ^*ERT2*^ *;Foxc1* ^*fl/fl*^ *;Foxc2* ^*fl/fl*^ (LEC‐*Foxc*‐DKO) males, respectively, as described previously (Norden *et al*, 2020). For Cre recombination efficiency detection, *mTmG*/+*;Cdh5‐Cre* ^*ERT2*^ *;Foxc1* ^*fl/fl*^ *;Foxc2* ^*fl/fl*^ (mTmG/EC‐*Foxc*‐DKO), *mTmG*/+*;Vegfr3‐Cre* ^*ERT2*^ *;Foxc1* ^*fl/fl*^ *;Foxc2* ^*fl/fl*^ (mTmG/LEC‐*Foxc*‐DKO) and *mTmG*/+*;Foxc2‐Cre* ^*ERT2*^ mice were generated by crossing mTmG females (*mTmG/mTmG;Foxc1* ^*fl/fl*^ *;Foxc2* ^*fl/fl*^ and *mTmG/mTmG*) with EC‐*Foxc*‐DKO, LEC‐*Foxc*‐DKO and *Foxc2‐Cre* ^*ERT2*^ males, respectively. Genotyping of mice was performed by Transnetyx Inc.

### Tamoxifen treatment

For adult mice, Tamoxifen (Tm, Cayman Chemical #13258) was dissolved in corn oil (Sigma #C8267) at 40 mg/ml by shaking at 37°C for 3–4 h. 7–8‐week‐old male adult mice were treated with 150 mg/kg Tm by oral gavage once daily for 5 consecutive days. For neonatal mice, each individual was treated with Tm (20 mg/ml, 75 μg) by oral gavage once daily from postnatal day 1 (P1) to day 5 (P5) (Norden *et al*, 2020).

### Cre recombination efficiency detection

mTmG/EC‐*Foxc*‐DKO, mTmG/LEC‐*Foxc*‐DKO and *mTmG*/+*;Foxc2‐Cre* ^*ERT2*^ mice were used. The Cre negative mTmG mice were used as control. Severn or 12 days after Tm treatment, the distal jejunum was harvested and fixed in 4% paraformaldehyde (PFA), followed by dehydration in 30% sucrose, and OCT (Sakura Finetek, USA) embedding. Ten or 15 μm cryosections were cut and immunostained with CD31 and/or LYVE1 antibody (Table 2), counterstained with GFP antibody and a nuclear‐specific dye DAPI. EGFP fluorescent signal was detected by imaging to evaluate the Cre recombination efficiency.

**Table 2:** Antibodies used for whole‐mount, sections, and western blot.

Primary antibodies					
Antibody	Supplier	Catalog number	Host species	Clonality	Application
β‐Actin	Sigma	A1978	mouse	mAb	WB
β‐catenin	Santa Cruz	sc‐59737	mouse	mAb	IHC‐P
BrdU	Abcam	ab6326	Rat	mAb	IHC‐P
CCND1	Invitrogen	MA5‐14512	Rabbit	mAb	IHC‐P
CD31	BD Biosciences	553370	Rat	mAb	WM, IHC‐F
CD31	Cell Signaling	77699	Rabbit	mAb	IHC‐P
CD31	R&D	AF3628	Goat	pAb	WM
EMCN	Abcam	ab106100	Rat	mAb	IHC‐P
EpCAM	Cell Signaling	93790S	Rabbit	mAb	IHC‐P
FOXC1	Abcam	ab227977	Rabbit	mAb	WM
FOXC2	Kind gift from Dr. N Miura (Miura *et al*, 1997)	‐	Rat	mAb	IHC‐P
GFP	Invitrogen	A‐11122	Rabbit	pAb	IHC‐F, WB
IgM	Invitrogen	61‐6800	Rabbit	pAb	IHC‐P, WB
LYVE1	Abcam	ab14917	Rabbit	pAb	WM, IHC‐P, IHC‐F
LYVE1	R&D	AF2125	Goat	pAb	WM, IHC‐P, IHC‐F
OLFM4	Cell Signaling	39141T	Rabbit	mAb	IHC‐P
PROX1	R&D	AF2727	Goat	pAb	IHC‐P
RSPO3	Sigma	HPA029957	Rabbit	pAb	IHC‐P
VEGFR2	R&D	AF644	Goat	pAb	WM
VEGFR3	R&D	AF743	Goat	pAb	WM

Secondary antibodies				
Antibody	Reactivity	Host species	Supplier	Application
Alexa 405‐conjugated	Rabbit	Donkey	Jackson Immuno Research Labs	WM, IHC‐P, IHC‐F
Alexa 488‐conjugated	Rat/Rabbit/Goat	Donkey	Thermo Fisher	
Alexa 568‐conjugated	Rat	Donkey	Abcam	
Alexa 568‐conjugated	Rabbit/Goat	Donkey	Thermo Fisher	
Alexa 594‐conjugated	Rabbit/Goat	Donkey	Thermo Fisher	
Alexa 594‐conjugated	Mouse (IgG1)	Goat	Thermo Fisher	
Alexa 647‐conjugated	Rat/Goat	Donkey	Thermo Fisher	
HRP conjugated	mouse	Goat	Thermo Fisher	WB
HRP conjugated	Rabbit	Goat	EMD	

IHC‐P, immunohistochemistry staining on paraffin sections; IHC‐F, immunohistochemistry staining on frozen sections; WB, western blot; WM, whole‐mount staining.

### Mouse small intestinal ischemia and reperfusion (I/R) surgery

Twelve days after Tm treatment, mice were subjected to small intestinal ischemia and reperfusion (I/R) surgery as previously described (Yoshiya *et al*, 2011). Briefly, mice were anesthetized with inhalation of Isoflurane. A midline laparotomy was performed, and the superior mesenteric artery (SMA) was then identified, isolated, and clamped by a small nontraumatic vascular clip. Heparinized saline (500 μl, 10 U/ml) was added into the peritoneal cavity via syringe to avoid coagulation of blood. After this ischemic phase for 30 min, the clip was removed, and the intestine was allowed to reperfuse. Five hundred microgram of sterile saline is administered to the peritoneal cavity to compensate for the fluid loss during surgery. A Chromic Gut Suture (4–0) was then used to close the muscle layer, followed by the skin closure with wound clips. Sham‐operated mice were subjected to the exact same surgical procedure, aside from clip placement.

### BrdU treatment

Mice were treated with one dose of BrdU (Sigma #B5002, 10 mg/ml in PBS, 5 mg/kg) by intraperitoneal (i.p.) injection 2 or 18.5 h (for evaluation of proliferative intestinal epithelium or proliferative BECs and LECs, respectively) before tissue dissection (at I/R‐24 h and I/R‐18.5 h respectively).

### Tissue collection

Distal jejunum was selected for this study because this segment has the most severe mucosal damage after intestinal I/R surgery compared with other segments according to pilot experiments. Different time points (3, 4 h for an early injury stage, 18.5, 24, and 48 h for a late repair stage) were chosen for different analysis purposes. For histological analysis, transcardial perfusion was performed on the adult mice with cold PBS followed by 4% PFA after anesthesia. Distal jejunum was harvested and cut longitudinally to expose the lumen. After several washes with PBS, the intestine was postfixed in 4% PFA at 4°C for 4 h (for frozen or whole‐mount samples) or for O/N (for paraffin‐embedded samples). For qPCR and western blot on whole tissue lysates of the small intestine, blood was removed by transcardial perfusion with cold PBS. Distal jejunum was harvested, opened longitudinally, and washed with cold PBS, then snap‐frozen in liquid nitrogen for RNA isolation and protein extraction. For the tissue collection for neonatal intestinal whole‐mount staining, the neonates were euthanized at P7 after Tm treatment from P1 to P5. The proximal jejuna were collected, washed with cold PBS, and fixed in 4% PFA at 4°C for 4 h, then subjected to the whole‐mount staining protocol. Proximal jejunum was collected from neonatal mouse due to the ease of operation and similar lacteal length/blood capillary network length ratio between proximal and distal jejuna (Bernier‐Latmani *et al*, 2015).

### Histopathological evaluation of intestinal mucosal damage

Paraffin sections of distal jejuna from the mice 24 h after intestinal I/R were stained with Hematoxylin and Eosin (H&E). Based on the H&E staining, Chiu Score (Chiu *et al*, 1970) was used for evaluating the intestinal mucosal damage after I/R: grade 0, normal mucosa; grade 1, development of subepithelial Gruenhagen's space at the apex of the villus; grade 2, extension of the space with moderate epithelial lifting from the lamina propria; grade 3, massive epithelial lifting with a few denuded villi; grade 4, denuded villi with exposed lamina propria and dilated capillaries; and grade 5, digestion and disintegration of the lamina propria, hemorrhage, and ulceration. Higher scores represent more severe damage.

### Whole‐mount (WM) staining

Whole‐mount staining of the small intestine was performed as previously described (Bernier‐Latmani & Petrova, 2016). Briefly, distal jejunum was dissected, and fixed in fixative (0.5% PFA, 15% picric acid, and 100 mM phosphate buffer, pH 7.0) at 4°C for O/N. Samples were washed with PBS, and subsequently dehydrated by 10% sucrose for 3 h, and 20% sucrose + 10% glycerol in PBS for O/N at 4°C. After PBS‐wash, samples were incubated with blocking buffer (5% donkey serum, 0.5% BSA, 0.3% Triton X‐100, 0.1% NaN_3_ in PBS) for 2 h at 4°C, and then incubated with the indicated primary antibodies (Table 2) diluted in the blocking buffer for O/N at 4°C. Samples were washed with PBST (0.3% Triton X‐100 in PBS) for several times, followed by incubation with indicated fluorochrome‐conjugated secondary antibodies (Table 2) diluted in the blocking buffer for O/N at 4°C. The samples were washed again with PBST, postfixed with 4% PFA, cut into thin strips (one or two villi wide), cleared with FocusClear (CelExplorer Labs #FC‐101), and mounted on slides in the mounting medium.

### Immunohistochemistry (IHC) staining

For IHC‐P, 4 or 15 μm paraffin sections were deparaffinized, rehydrated, subjected to antigen retrieval, permeabilized with PBST, blocked with blocking buffer containing 5% donkey serum in PBST for 30 min at room temperature (RT), and incubated with indicated antibodies (Table 2) in blocking solution for O/N at 4°C. The sections were washed with PBS and incubated with indicated fluorochrome‐conjugated secondary antibodies (Table 2) in PBS for 1 h at RT. After wash with PBS, the sections were counterstained with DAPI and mounted with the mounting medium. For IHC‐F, 10 or 15 μm frozen sections were washed with PBS, and then subjected to the same blocking and antibody incubation protocols as IHC‐P but without the antigen retrieval step. TUNEL staining was performed using *In Situ* Cell Death Detection Kit (Roche #11684795910) according to the manufacturer's instructions.

### Imaging

H&E staining images were acquired using an Olympus Vanox AHBT3 Research Microscope (original magnification 100×, Tokyo, Japan) and an Apple iPhone 12 Pro Max. Fluorescent images were acquired using a Zeiss AxioVision fluorescence microscope, a Nikon A1 Confocal Laser Microscope, or a Nikon AXR confocal microscope with the software of Zeiss AxioVision SE64 Rel. 4.9.1 or NIS‐Elements Viewer, respectively. Images were processed and analyzed with Adobe Photoshop, Imaris, and Fiji (ImageJ) software. Imaris imaging software was used to create videos (Movies EV1 and EV2) for the 3D structure of the blood and lymphatic vasculatures in villi after intestinal I/R.

### Lacteal permeability detection

The mouse lacteal permeability was assessed as previously described (Liu *et al*, 2019; Hu *et al*, 2021) with modifications. Briefly, mice were fasted for 9 ~ 12 h before the treatment with 50 μg BODIPY™ FL C16 (ThermoFisher D3821) dissolved in 200 μl Intralipid (Sigma I141‐100ML) by oral gavage. Mice treated with Intralipid only were used as the vehicle control. For WM staining, 1 h after BODIPY C16 treatment, the intestine was dissected and processed for the WM staining of lymphatic vessel marker LYVE1. Confocal imaging was performed for the lacteals. The fluorescent intensity (FI) of BODIPY C16 within the lacteals and the lacteal area were quantified and measured. The BODIPY C16 uptake index represents the ratio of BODIPY C16 intensity in lacteal vs. lacteal area. To detect the absorption of BODIPY C16 at a later time point, 3 h after BODIPY C16 treatment, the mice were euthanized and their blood was collected by cardiac puncture. The serum was collected by centrifugation after blood clotting for 30 min at RT. The whole length of the small intestine was dissected, opened longitudinally, and washed in 10 ml PBS. The supernatant was collected as the intestinal content after centrifugation. The FI of BODIPY C16 in intestinal content and serum was measured by using a microplate reader (SpectraMax M2). The dissected distal jejunum and the liver were imaged under Nikon AZ100 fluorescent microscope and the FI of BODIPY C16 in the tissue was quantified.

### Rescue experiments

In rescue experiments, mice were treated with 5 μg RSPO3 (R&D systems #4120RS025CF) in 100 μl PBS (Kannan *et al*, 2013), or CXCL12α (PeproTech #250‐20A) in PBS at a dose of 50 μg/kg of body weight (BW; Garnica *et al*, 2005) by retro‐orbital injection 30 min before intestinal ischemia. Distal jejunum was harvested after I/R at 18.5 h (WB) or 24 h (H&E, IHC) for further analysis.

### Cell isolation from mouse small intestine

Epithelial cells (Epis), blood endothelial cells (BECs), and lymphatic endothelial cells (LECs) were isolated from the distal jejunum for further qPCR analysis as previously described (McCarthy *et al*, 2020b) with slight modifications. Briefly, the intestine was washed with cold PBS after dissection and incubated in 10 ml Hank's Balanced Salt Solution (HBSS) containing 10 mM EDTA at 37°C for 20 min. After shaking the tube vigorously for 30 s, the epithelium was then removed from the intestine. The supernatant containing the Epis was filtered through a 70 μm and a 40 μm strainer. The Epis were then sorted by using magnetic Dynabeads (Invitrogen #11035) labeled with CD326 antibody (BD 552370). The remaining intestine without epithelium was further washed in HBSS and processed upon dissociating into single‐cell suspension in a digestion buffer (1.5 mg/ml Collagenase D, 60 U/ml DNAase I, 5% FBS, 100 units/ml penicillin and 100 μg/ml streptomycin in DMEM) for 40 min at 37°C, followed by filtration through a 70 μm and a 40 μm cell strainer. After several washes, the cell suspension was then incubated with Dynabeads labeled with CD45 antibody (Biolegend #103102) to deplete the CD45^+^ cell population. The CD45^−^ cell suspension was then incubated with Dynabeads labeled with LYVE1 antibody (ThermoFisher 14‐0443‐82) to get the CD45^−^LYVE1^+^ LECs. The CD45^−^LYVE1^−^ cell suspension was incubated with Dynabeads labeled with CD31 antibody (BD #553369) to get the CD45^−^LYVE1^−^CD31^+^ BECs. Finally, the sorted Epis, LECs, and BECs were used for RNA isolation and qPCR analysis. For the detection of gene deletion in ECs in EC‐*Foxc*‐DKO mice, CD45^−^CD31^+^ ECs (including BECs and LECs) were isolated by using Dynabeads labeled with CD45 antibody (depletion of CD45^+^ cells) followed by Dynabeads labeled with CD31 antibody (positive selection of CD45^−^CD31^+^ cells).

### RNA isolation and qPCR analysis

A RNeasy Mini Kit (Qiagen #74104) was used for RNA extraction from cells. TRIzol™ Reagent (Invitrogen #15596026) was used to isolate RNA from whole distal jejunum. The concentration of RNA was determined using NanoDrop™ 2000 Spectrophotometers (Thermo Scientific). cDNA was synthesized using an iScript reverse transcriptase kit (Bio‐Rad #170‐8891). qPCR was performed on triplicates of cDNA samples by using QuantStudio® 3 Real‐Time PCR System (Applied Biosystems), Fast SYBR reaction mix (Applied Biosystems), and gene‐specific primer sets. 18S (for mouse samples) and PPIA (for human cells) were used as internal standards for mRNA expression in mouse samples. Primer sequences are provided in Table 3.

**Table 3:** Primers used for qPCR analysis.

Species	Gene	Forward	Reverse
Mouse	*Cox2*	GCATTCTTTGCCCAGCACTT	GGCGCAGTTTATGTTGTCTGT
	*Cxcl12*	GCTCTGCATCAGTGACGGTAA	CGTGCAACAATCTGAAGGGC
	*Foxc1*	TTCTTGCGTTCAGAGACTCG	TCTTACAGGTGAGAGGCAAGG
	*Foxc2*	AAAGCGCCCCTCTCTCAG	TCAAACTGAGCTGCGGATAA
	*IL‐6*	AGAGGATACCACTCCCAACAGA	CCACGATTTCCCAGAGAACA
	*Rspo3*	TGTGAGGCCAGTGAATGGAG	ATCTCGGACCCGTGTTTCAG
	*TNF‐α*	ACAGAAAGCATGATCCGCGA	CTGCCACAAGCAGGAATGAG
	*18S*	GAAACTGCGAATGGCTCATTAAA	CCACAGTTATCCAAGTAGGAGAGGA
Human	*FOXC1*	TCACAGAGGATCGGCTTGAAC	CGTGCGGTACAGAGACTGG
	*FOXC2*	GGGGACCTGAACCACCTC	AACATCTCCCGCACGTTG
	*ICAM1*	CCTTCCTCACCGTGTACTGG	AGCGTAGGGTAAGGTTCTTGC
	*LYVE1*	GGTCTTGGATTTTGCTATGTCA	CTCATTAGGGTTGCTATCATTGG
	*PPIA*	CCTAAAGCATACGGGTCCTG	TTTCACTTTGCCAAACACCA

### Western blot

The frozen intestinal tissue was grinned using mortar and pestle chilled with liquid nitrogen, followed by lysis in RIPA buffer (150 mM NaCl, 1% Nonidet P‐40, 0.5% sodium deoxycholate, 0.1% SDS and 50 mM Tris, pH 7.4) containing protease inhibitors (Roche #4693116001). After centrifugation, the supernatant of the tissue lysates was collected and mixed with 5× Protein Loading Buffer. Equal amount of total protein for each sample was loaded and run on an SDS–PAGE gel. Samples were transferred to 0.45 μm nitrocellulose (Invitrogen) and western blotted with the antibodies listed in Table 2, followed by the reaction with ECL substrates. The chemiluminescent signal was then detected by imaging the blot with Azure c600 imaging system. Bands were quantified using ImageJ software.

### Preparation of single‐cell suspension from mouse small intestine

For single‐cell RNA sequencing, mouse distal jejunums were collected 18.5 h after intestinal I/R surgery. Two mice were used for each group: control I/R‐18.5 h and EC‐*Foxc*‐DKO I/R‐18.5 h. Briefly, mice were anesthetized by isoflurane. Blood was removed by cardiac perfusion with cold PBS. The distal jejunum was then dissected, washed with cold PBS, and cut into small pieces. The tissue was processed for scRNA‐seq upon dissociating into single‐cell suspension in a digestion buffer as mentioned above for 35 min at 37°C, followed by filtration through a 70 μm and a 40 μm cell strainer. Cells were washed with washing buffer (0.5% BSA, 2 mM EDTA in PBS, pH 7.4) for three times and resuspended in PBS with 0.04% BSA at a concentration of 1,200 cells/μl (according to 10× Genomics Document #CG00053 Rev B) before being passed through a 30 μm MACS SmartStrainer. The cell viability was tested by using the Cellometer Auto 2000 Cell Viability Counter (Nexcelom Bioscience, USA). The cell sample was processed for scRNA‐seq only when the cell viability was more than 70%. Average cell viability for samples was determined to be 80.96%.

### Single‐cell 3′ gene expression library construction and sequencing

Single‐cell 3′ gene expression libraries were constructed by using the Chromium Next GEM Single‐Cell 3' Reagent Kits v3.1 (10× Genomics, Pleasanton, CA, USA) according to the manufacturer's manual CG000204 Rev D. The single‐cell libraries were assessed for quality (TapeStation 4200, Agilent, Santa Clara, CA, USA) and then run by using paired‐end 50 bp sequencing on the Illumina HiSeq 4000 platform (Illumina, San Diego, CA, USA). Ten thousand cells were targeted for each sample with a sequencing depth of 20,000 read pairs per cell.

### Preprocessing of single‐cell RNA data

Following library generation and sequencing, raw sequencing data were de‐multiplexed and mapped to the mouse reference genome (mm10) using the CellRanger toolkit (10X Genomics, version 2.1.0). Gene expression matrices were then generated from both control and EC‐*Foxc‐*DKO mice using CellRanger. The matrix files were then utilized for data processing and downstream analysis using the BIOMEX browser‐based software platform and its incorporated packages developed in R (Taverna *et al*, 2020). Quality control and data pretreatment was performed in BIOMEX with the following manually set parameters: (i) genes with a row average of < 0.001 were excluded for downstream analysis and (ii) cells in which over 10% of unique molecular identifiers (UMIs) were derived from the mitochondrial genome were considered as dead cells and removed from downstream analysis. The data were then normalized in BIOMEX using a similar methodology to the *NormalizeData* function as implemented in the *Seurat* package (Satija *et al*, 2015).

### Variable gene identification, dimensionality reduction, clustering analysis, and differential gene expression analysis

Following data pretreatment, BIOMEX was utilized for downstream dimensionality reduction in data and clustering analysis using the incorporated R packages. First, highly variable genes were identified utilizing the following feature selections: mean lower threshold = 0.01, mean higher threshold = 8, and dispersion threshold = 0.5. Data (using highly variable genes only) were then auto‐scaled and summarized by principal component analysis (PCA), followed by visualization using Uniform Manifold Approximation and Projection (UMAP; top 15 principal components (PCs)) to reduce the data into a two‐dimensional space. Graph‐based clustering was then performed in BIOMEX to cluster cells according to their respective gene expression profile using a methodology similar to the *FindClusters* function in *Seurat* (clustering resolution = 0.5, k‐nearest neighbors = 15). Marker set analysis was then performed in BIOMEX on highly variable genes to identify the top 10 gene markers expressed in each initial cluster using a similar methodology described previously (Kalucka *et al*, 2020). Marker genes were then compared with single‐cell RNA‐seq data from the small intestine of adult mice available from the Mouse Cell Atlas (MCA, http://bis.zju.edu.cn/MCA) and previously reported the data (Haber *et al*, 2017) to identify transcriptionally unique cell populations. Clusters with highly similar expression patterns indicative of the same cell phenotype were merged into the same cluster. Differential gene expression analysis between control and EC‐*Foxc*‐DKO mice for individual cell clusters was performed in BIOMEX using the Model‐based Analysis of Single‐cell Transcriptomics (MAST) package (Finak *et al*, 2015). *P*‐values < 0.05 were considered statistically significant for differentially expressed genes.

### Data visualization

BIOMEX implementation of the *Plotly* software was used for UMAP and volcano plot visualization.

### Examination of published scRNA‐seq data

The scRNA‐seq data for mouse small intestinal ECs from the publication entitled “Single‐Cell Transcriptome Atlas of Murine Endothelial Cells” (Kalucka *et al*, 2020) was examined. The data are available from the website of “Single Cell Expression Atlas” (https://www.ebi.ac.uk/gxa/sc/home) and was exported to check *Lgr5* expression in mouse small intestinal ECs.

### Forkhead box C transcription factor binding prediction analysis in *RSPO3* and *CXCL12* loci

Putative FOX‐binding sites in the *RSPO3* and *CXCL12* loci were first identified using the Hypergeometric Optimization of Motif EnRichment (HOMER; Heinz *et al*, 2010) suite of tools to scan the entire Genome Reference Consortium Human Build 37 (GRCh37 or hg19) genome corresponding to the conserved RYMAAYA FOX transcription factor binding motif or the reported RYACACA FOXC transcription factor binding motif (Chen *et al*, 2019). The output files were then uploaded to the UCSC genome browser (Kent *et al*, 2002) to identify putative binding sites corresponding to transcriptionally active areas denoted by histone modification, DNAse sensitivity, and additional transcription factor chromatin immunoprecipitation data as per work reported and summarized on the Encyclopedia of DNA Elements (ENCODE; https://genome.ucsc.edu/ENCODE/; 2012). Putative sites in the human genome were then searched against the Genome Reference Consortium Mouse Build 38 (mm10) genome using the Evolutionary Conserved Region (ECR) Browser (https://ecrbrowser.dcode.org) and rVista 2.0 tools to identify conserved and aligned putative binding sites between mouse and human sequences. Conserved and aligned putative FOX‐binding site sequences are underlined and bolded within the human *RSPO3* and *CXCL12* ECRs shown below:

#### *RSPO3* *ECR‐1*. > hg19 chr6:127468065–127468506

TCTTGAAATGGATAATGATAGATTCCAAACACATGGAAATCTCTTGCCCCTTTTACTTTTTAGGATCTTTGCAAGCTTACAATATGTACACGTTTTCTGTAAGTCACCAATGCTGAGTTACTGGCATGAAAAATGACCCTGTTACTTGGAAAGTAGTTTCACTTACAAGTCCCCCAGGCCCTGTAATGTCTAAACCTCCTGTGCCACTTTATGTGACTACCCCGCCCCCACAGAGGAGCATGCACAGGAAAAGCAGACTTCCCTTCCCCCACACATTTCCTTAGTT **TATTTAC** AAAACGTCTTGGAATGAGAATGAGCTGCTTGTGGTTCCTGTGGCTGATTCAGGGATGGTTTCCTACAGGCAGAGGATGCTGGTCAACCGAATGACCTCTCTGTAACTAACCCGTGCACCCCTGTGGTAAGGCTGTTTGGTCTTATAGGTACCTCTTCTAACTAAGCTTGGAGGGATTTGTTTTTGTGGTAAAGAACTTAGTAATAACCAAACGTCACTGTAAAGACAGATTTAATAATGTTAAGGTCCATCAGAGCCTACTCCTTCTACTACCAACAAGAGAAGCCAGAAATACACTGGGATGCCTTTAGATTCCTGTGCATCAATCTTTCTTTCTCTAAGGATTATGG

#### *RSPO3* *ECR‐2*. > hg19 chr6:127497100–127497633

AATACCAGTAAATGGGGACACATTATATTGAATAAGGGTATTGTTAGCCAAATTCTAAGATTCATCTTAAATTGTTTTCTTATAAGAATTGTGTATTTACCATTTTAAAAATCACTATTATTTTAAAACACTTAGAAAGTGAACATTTGAAAATGATGTGCCTTTGGATGCTCTGTAATGTTAAGCAGATCCAGACATAAAGACAAAAGTAAATTCCAGAGTATTTTTGTAGCCATGGAATCACCATAAAAAGGGGTTTTTGACCCCAATGTTACCGTAACATTGTCTTCAGCATTTCATATTTAATTACAGTAGATTACTCACCAATATA **ATAAATA** TAGATTTATGAGATACTTTAATGTTCTAAAACAAATGAAAACCACCCAAGAGGAGCCTCACCAAACCTGAGGTTGTCCAGATTGCATTGACTAAGATTAAGTAAAAGATCATTCATCTCCAGAGGTCATGCAATTAATCTCAGAGTGGGAGTTAAAGCAATGACTAAGCAGAAAAGGAAGCCAAATACAAGCTCGTAACAAAAGGTGCTGGGGCTCCAACATCAAGGAACTTGTTATTTCCTTTTTATTTATTTATTTTTTTTTAATAGACCTAAAACACTCATTCCTTACTACTGGTTTCTTTGGGTCCTAAAATTCCACTTGGTTAGGTCAGCTATTTTCCATGACTATTTTTGATACGGTCAAACAAATACAAAGAATAAGCTTTTAAAAAAC

#### *CXCL12* *ECR‐1*. > hg19 chr10:44888442–44889025

ATCAAAACCTTCACCTTTCTCTGCTGAAGGAATGGCCTTCTCTTATGGGCAGGGAGGGTTTCCTAGGGAAAGCCCACCCAGGCAGGAGATGAGGAGAGCAGCATCTGAGCACACTTCATCCCACAGTGCCCATCCCATGAGTATCCTCCATAAATTACAAAGAAAGAAAAAAAATAGGGAAAAAACAAACCTTTATTTCTC **TGTTTAC** TTCTCTGCATTTAATGAGCAGTTGTTAACATGACTGAAACCATCTGATGATTTTTACCAAATGGAAAAATCTGCCTACAGGGGCAATAAA **ATAAATA** TTCAGAATAGAGAGAGGCAGTCATAAAAGACATTACCCGGTTGTAAACGGAGGCGGGTGGTGGTGATCTATTACCCCTGCCTCGGCAGCTTTCAACAGAGTTCTGGAATTCCAGGAGGGGCCCTGACCCAAGGCAA **TATTTAC** TTCTGCGGCTTCTTCATCAGGTCAGCATGGGTATAATTCTGTCTACCAGTTGACTGGAGCTGAGGTTTCGAGCAGGAAGTGCAAACCCTGAGTGCTTATAACTCAGGAGGAGTGAGGCACCCCTTCCCAGAGTATGCCAAGAAAAGCACATTGTACTGTCCTGGCTGCAGGGGTGAGGCCCCTGCACACCCAGGCCATTATCAGCTTTGTGCCCTGGCCAACAGCGCCTCTGGTTGGTGCATTTGTCAGCTGTATTTTACCCTAGAGCTCTGGGAGGCTCATCCTTTTTTGGTATACCACCACGTGGAGAGAGCAGAGTTTTAATAGTGTGGCTGC

#### *CXCL12* *ECR‐2*. > hg19 chr10:44872457–44872993

GGGTTAAAAAAAAAAAAAAAAGATCCAAAAACTTGAGCTGCAGATCTAATCTGCTCGTGAGAAAAGCCCATACACTGTCACACATGGGCTGTGAGAAGGGGTCTCAGACACCTGACTGCAGGCAGGCTTAACTATATAAACCAGAAACGTCTATAAGCTCCATCACTAACAACTAATGAATTTTATTTCAG **GTAAATA** AATTCCCACATACAGTAGGACGTTTATACCATGAAACAATTAGCATTTTATTGCTAGTGCATATAATGTCACATTTGATACAATTTTAGTACAAGTGAAAAAATACACTGTGGCTAACATTGAAAAGCTGCAATCACATTTATATATCATATATATTTCTTTACAAATTGCCAGTAGTTTGAGATAATAGAGAAGTATAAACTACTGACATTCATATGGCTCCACTTCAAATATATGAATTGTTCGACT **ATAAATA** TATTTTGAAATACATTTGTTTTCTAAAGAAACGTAAAAAAAAATGTGCACAAAAATATATATAAAAAAATGCCTTGCAAAAAGTTACAAATACCACCAGGACCTTCTGTGGATCGCATTTATGCATGGAAATGTCACCTTGCCAACAGTTCTGATTGGAACCTGAAACCCTGCTGTGGCTTCAGGAGGGGGTAGTGGCAAGATGATGGTTTATTCACTGATTTTTTCGCTTCTGATTTCGGAAACCTCAGAGTTTGTTAGTGCCTC CATGGCATACATAGGCT

As comparison, in mouse, the conserved and aligned putative FOX‐binding sites (underlined and bolded) within the mouse sequences of *Rspo3* and *Cxcl12* ECRs corresponding to the human *RSPO3* and *CXCL12* ECRs are shown below:

#### *Rspo3 ECR‐1*. > mm10 chr10:29507907–29508615 (identity: 76.5%)

TAGACATGGACACTATTAATCTGTATTTGCAATGATGTCTGATAATTAAGCTCTTTATTGCAGAAACAAAGCCCTGCAGTCTTACGATGCCACACCAAGAAGCAGAGAACAGGAGGAGAACCATGGTCTGGGAAATAAACTTCAAAAGTCCCATATTAATAATTTTTCTCCTCCAGCAAGGCCACACCTAAGGGCTCCATCTCTCCCCCAGATATTATCACCTACTAGCTAGGGATCAAGTATTCAAAACATGAGCCGGGAAGAATTATTTCATATTTCAAGTATAATATTAGTCTGTAATCACAGTGATAATTGGTTATTAAGCTCTTCATTGGAACAACAAAGCCCTCCAGGCACAGTCTGAAATGATACCCATAACAAAGATCCTTCCTGTTGGGGTATAGGAGTTAGCTCCATAGAGGTCATTCAGTTGACCCGCTTCCCTGGCCTGCAGGAAACCATCCCTGAATAAGCCACAGGAACCACAAGCAGCTCGCTCTCATTCCAAGATGTTTC **ATAAATA** AACTGAGGAAATGTGTGGGGGAAGGGAGGGCTGCTTTTCCTGTGCATGTTCTTCTGTAGGGTGGGGCAGTCCTATAAAGTGGGTGAGGAGGGTTTGCCATTATGGGACCTGAGGGATTTGTACCAGAAACTCCTTTCCAAGGAAGGTCATTTTTTATGACAGGGGACTAGGGGTGGGGGAGTGCTT

#### *Rspo3 ECR‐2*. > mm10 chr10:29481015–29481541 (identity: 78.3%)

CAACAATAAGGAGTATTTTAGGTTTATTCAGGAGGAGGAAAAAAAAAAGGAAATAGCTCCTTGATGTTGGAGGCCAGGCCCCTCTTGTTATGGGCTTGCATGCTGGCTTTCTTTTCTGCTTAATCATCGCCTGAACTCCCACTCTAAGATTGCTTACATAACCCTGGGAGATGAATGATCTTCTACTTAATTTCAGCCAGTGCAATCTGGACAACCTCAGGTTGGATTGGTGAGGCTCCTCTTGGGTGGTTTTCACTTGCTTTAAGAACATTAAAATATCTCATAAATCTA **TATTTAT** TATATTAGTGAATACTATACTACAATTAGATATGAAATGCTGAAGACAATGTTAAGGTGAGAGTGGGTTCACAAACCTCTATATTGGTAGCTTCAGAATTGCAAAAACATTTGTGGAATATTGGTTTTGTCTCTATGCCTGGATCTGTTTAACATAACATAGTATCCACAGCACACTCTTTCAGACAGGGACTTTGTGAATCCTCTAAAACAACCATCATTTCTAAATG

#### *Cxcl12 ECR‐1*. > mm10 chr6:117160192–117160784 (identity: 82.5%)

CAGCCATCAGTACCGTCTGCCCAAGTACAAACAAGCTGTATATCCCACTGACAATGGCCTGGTTATGTGGGACCCTCACCTTGACAGCCAGGACGGTACCATGAGCTTTTCTTGGCACACCCTGGAAAGGGTGCCTTACTCTTCCTGAGTTATGAGTGCTTAGGGCTCTGGCTTCCTGCTCAAAACCTCAACTCCAGTCAACTGGTGGACAGAATTATACCCACAATGACCTGCCGAAAAAGCCACAGAA **GTAAATA** TTGCCTTGGGTCAGATCCTCTCCTGGAATCCCAGAACTGTTGAAAGCTGCCAAGGCAGGGGTAATAGATCACCACGACCCGCCTCTGTTTACAACCGGGTAATGCCTTTTACGACCGCCTCTCTCTGTCCTGAA **TATTTAT** TTTATTGCCCCTGTAGGGAGATTTTTCCATTTGGTAAAAATCATCAGATGGTTTCAGTCATGTTAACAACTGTTCATTAAACGCAGAGG **GTAAACA** GAGATGGAAAGGTTTGTTTCTTTCCTTTTTCTCTATTTATTTTTAACTTATGGAGAAAACTCAGGGAGTGAACCCAGGGAGGGAGGCATGTTCAGATAC

#### *Cxcl12 ECR‐2*. > mm10 chr6:117174613–117175137 (identity: 81.2%)

TGAAGCCACAGTGGGGATTCTGGGTTCCAATCAGAAATGGAGACAAGATAAAACTTGCATACATTCTTATGATCACAGACGGCCCTGGTGGTTTTTGGTAACTATTTACAAGGCATTTTTTTACATATATTTTTGTGCACTTTTTATGTTTCTTTGGAAGACAAATGTATTTCAGAATA **TATTTGT** AGTCAATTCATATATTTGAAGTGGAGCCATAGTAATGCCAGTAGATATCTCTATGATCTTGAGCTACTGGCAACTTGTAAAGAAATATATATGACATATAAATGTATTGTAGCTTTCCGGTGTCAGCCACGGTGTATTTTTCCACTTGGAATGAAATTGTATCAACTGTGACATTATATGCACTAGCAATAAAATGCTAATTGTTTCATGCTGTAAACCTCCTACCGTATGTGGGAATT **TATTTAC** CTGAAATAAAATCTACTAGTTGTTAGATGGAGTGCACATACATTTCTGAAGATGGAGAAAAACAGGTGTGCCTGCTGATCAGGTGCTGTGGGC

### Cell culture and chromatin immunoprecipitation (ChIP)

The ChIP experiment was performed as previously described (Xia *et al*, 2021). Briefly, the Human Umbilical Vein Endothelial Cells (HUVEC, ScienCell, #8000) and Human Dermal Lymphatic Endothelial Cells (HDLEC, PromoCell, C12216) were grown as seller's instructions and fixed with 1% formaldehyde for 12 min. The Pierce™ Magnetic ChIP Kit (ThermoFisher #26157) was used according to the manufacturer's instructions. Three microgram of ChIP grade FOXC1 antibody (Thermofisher, PA1‐807) and FOXC2 antibody (Santa Cruz, sc‐515472) was used for each reaction, while the same amount of normal rabbit IgG and mouse IgG2b was served as control. ChIP products were analyzed by Quantitative real‐time PCR. The sequences of primers were designed to target FOXC1‐ and FOXC2‐binding sites at *CXCL12* promoters (*CXCL12 promoter‐1*. > hg19 ch10: 44881184–44881195; *CXCL12 promoter‐2*. > hg19 ch10: 44880958–44880969) according to the prediction of JASPAR, as well as the binding sites at the ECRs of *CXCL12* and *RSPO3* mentioned above. The ChIP primers are listed below:

*CXCL12* promo‐1‐f, TTTCACCATTGAGAGGTCGG;

*CXCL12* promo‐1‐r, TCTTTGCAGTCAGCGTGG;

*CXCL12* promo‐2‐f, CCTCGGCTGCCTTGGGCCG;

*CXCL12* promo‐2‐r, CCTTTCGATGGCGGGAACTG;

*CXCL12* ECR‐1‐f, ACATGACTGAAACCATCTGATG;

*CXCL12* ECR‐1‐r, ACCCGCCTCCGTTTACAAC;

*CXCL12* ECR‐2‐f, AACTATATAAACCAGAAACGTCT;

*CXCL12* ECR‐2‐r, AAATGTGACATTATATGCACTAG;

*RSPO3* ECR‐1‐f, ATGCACAGGAAAAGCAGACTTCC;

*RSPO3* ECR‐1‐r, TCCCTGAATCAGCCACAGGAAC;

*RSPO3* ECR‐2‐f, TGTTACCGTAACATTGTCTTCAG;

*RSPO3* ECR‐2‐r, AGGTTTGGTGAGGCTCCTCTTG

### Mouse NEC model

24‐h‐old mice were submitted to a well‐characterized NEC model (Tian *et al*, 2010) conducted in a 33–35°C infant incubator or left with the dam. The NEC protocol includes: (i) initial orogastric inoculation with a standardized adult mouse commensal bacteria preparation (10^8^ colony‐forming units) and LPS (5 mg/kg) to perturb the normal intestinal colonization process; (ii) gavage with formula every 3 h (Esbilac, 200 ml/kg/day); and (iii) exposure to brief episodes of hypoxia (60 s in 100% N_2_) followed immediately by cold stress (10 min at 4°C) twice daily. This protocol induces intestinal injuries ranging from epithelial injury to transmural necrosis resembling human NEC, which typically develop after 36 h and has been widely used to study NEC pathogenesis (Yan *et al*, 2016). Pups were euthanized by decapitation at 24 h into NEC and whole intestinal tissues were collected and fixed in formalin for 24 h before tissue processing and paraffin block preparation.

### Quantification

For quantification of BrdU^+^, OLFM4^+^, or CCND1^+^ cells in crypts, images from different fields of section under a 20x objective were acquired and about 15 ~ 50 crypts were analyzed for each sample. The number of BrdU^+^, OLFM4^+^, or CCND1^+^ cells was counted for each crypt. For quantification of β‐catenin staining, about 5 ~ 16 ISCs (OLFM4^+^) per sample were analyzed for the fluorescent intensity (FI) of β‐catenin within the ISC by ImageJ. For quantification of FI of FOXC1 and FOXC2 in intestinal BECs and LECs, about 40 ~ 50 cells were quantified by ImageJ. For analysis of BEC and LEC proliferation and apoptosis, confocal Z‐stacks were acquired using a 20× objective from about 4 ~ 8 different fields of 15 μm paraffin sections for each sample. Area of blood vessels (CD31+LYVE1^−^) and lymphatic vessels (CD31^+^LYVE1^+^) were measured using ImageJ software. Then, the number of BrdU^+^ or TUNEL^+^ cells per 0.1 mm^2^ vessel areas was calculated and compared between groups. Measurements for the length of blood capillaries and lacteals were performed as previously described (Bernier‐Latmani & Petrova, 2016). The number of branches and branching points for the villous blood vasculature was calculated based on the whole‐mount staining of VEGFR2 as previously described (Bernier‐Latmani & Petrova, 2016). Around 30 ~ 50 villi were analyzed for each sample. For CD31^+^ vessel density quantification, 5 ~ 10 images were acquired using a 20× objective and the CD31^+^ vessel area and the intestinal tissue area in the images were measured using ImageJ software. CD31^+^ vessel density percentage was determined as the total CD31^+^ vessel area/total intestinal tissue area × 100%.

### Statistics

For quantification, statistical analysis was performed using GraphPad Prism 8.0 (GraphPad Software). *P*‐values were obtained by performing the Mann–Whitney *U* test or Kruskal–Wallis one‐way ANOVA test. Data are presented as box‐and‐whisker plots of representative experiments from at least three biological replicates. *P*‐values < 0.05 were considered statistically significant. For scRNA‐seq data, differential gene expression analysis between groups for individual cell clusters was performed in BIOMEX using the Model‐based Analysis of Single‐cell Transcriptomics (MAST) package (Finak *et al*, 2015). Adjusted *P*‐values < 0.05 were considered statistically significant for differentially expressed genes. For ChIP assay data, data are mean ± SEM and *P*‐values were obtained by performing the paired *t*‐test.

### Study approval

All experimental protocols and procedures used in this study were approved by the Institutional Animal Care and Use Committee (IACUC) at Northwestern University.

## Author contributions

**Can Tan:** Conceptualization; data curation; formal analysis; validation; investigation; visualization; methodology; writing – original draft; writing – review and editing. **Pieter R Norden:** Data curation; formal analysis; visualization; writing – original draft. **Wei Yu:** Data curation; formal analysis; investigation; visualization; methodology. **Ting Liu:** Data curation; investigation; methodology. **Naoto Ujiie:** Data curation; formal analysis; investigation. **Sun Kyong Lee:** Investigation. **Xiaocai Yan:** Investigation. **Yaryna Dyakiv:** Investigation. **Kazushi Aoto:** Resources. **Sagrario Ortega:** Resources. **Isabelle G De Plaen:** Resources; methodology. **Venkatesh Sampath:** Resources; funding acquisition. **Tsutomu Kume:** Conceptualization; supervision; funding acquisition; investigation; writing – original draft; project administration; writing – review and editing.

## Disclosure and competing interests statement

The authors declare that they have no conflict of interest.

## Supporting information



Appendix

Expanded View Figures PDF

Movie EV1

Movie EV2

Source Data for Expanded View and Appendix

PDF+

Source Data for Figure 1

Source Data for Figure 2

Source Data for Figure 3

Source Data for Figure 4

Source Data for Figure 5

Source Data for Figure 6

Source Data for Figure 7

Source Data for Figure 8

Source Data for Figure 9

Source Data for Figure 10

## Data Availability

All data needed to evaluate the conclusions in the paper are present in the paper and/or the EV materials. The scRNA‐seq data sets were uploaded to the NCBI tracking system‐GEO repository (GEO accession number: GSE190581). Additional data related to this paper may be requested from the authors.
