# Supplementary material for: Endothelial FOXC1 and FOXC2 promote intestinal regeneration after ischemia–reperfusion injury
Source: EMBO Rep. 2023 May 8;24(7):e56030. doi: 10.15252/embr.202256030 (PMC10328078; doi:10.15252/embr.202256030)
Supplement: Supplementary file 1 — Appendix [file EMBR-24-e56030-s006.pdf]

Endothelial FOXC1 and FOXC2 promote intestinal regeneration after ischemia-reperfusion injury

Can Tan, Pieter R. Norden, Wei Yu, Ting Liu, Naoto Ujiie, Sun Kyong Lee, Xiaocai Yan, Yaryna Dyakiv, Kazushi Aoto, Sagrario Ortega, Isabelle G. De Plaen, Venkatesh Sampath, and Tsutomu Kume*

*Correspondence to:

Tsutomu Kume, Feinberg Cardiovascular and Renal Research Institute, Department of Medicine, Northwestern University School of Medicine, 300 E. Superior Street, Chicago, Illinois 60611, USA

Phone: 312.503.0623; E-mail: t-kume@northwestern.edu

Contents

Appendix Figure S1. EC-iKO of *Foxc1/c2* does not affect the lacteal permeability. 2

Appendix Figure S2. FOXC1 and FOXC2 are expressed in HDLECs and HUVECs. 3

Appendix Figure S3. RSPO3 treatment alleviates IgM accumulation in intestinal mucosa of EC-*Foxc*-DKO mice after I/R. 4

Appendix Figure S4. *Lgr5* is expressed in both intestinal BECs and LECs by scRNAseq. 5

Appendix Figure S1

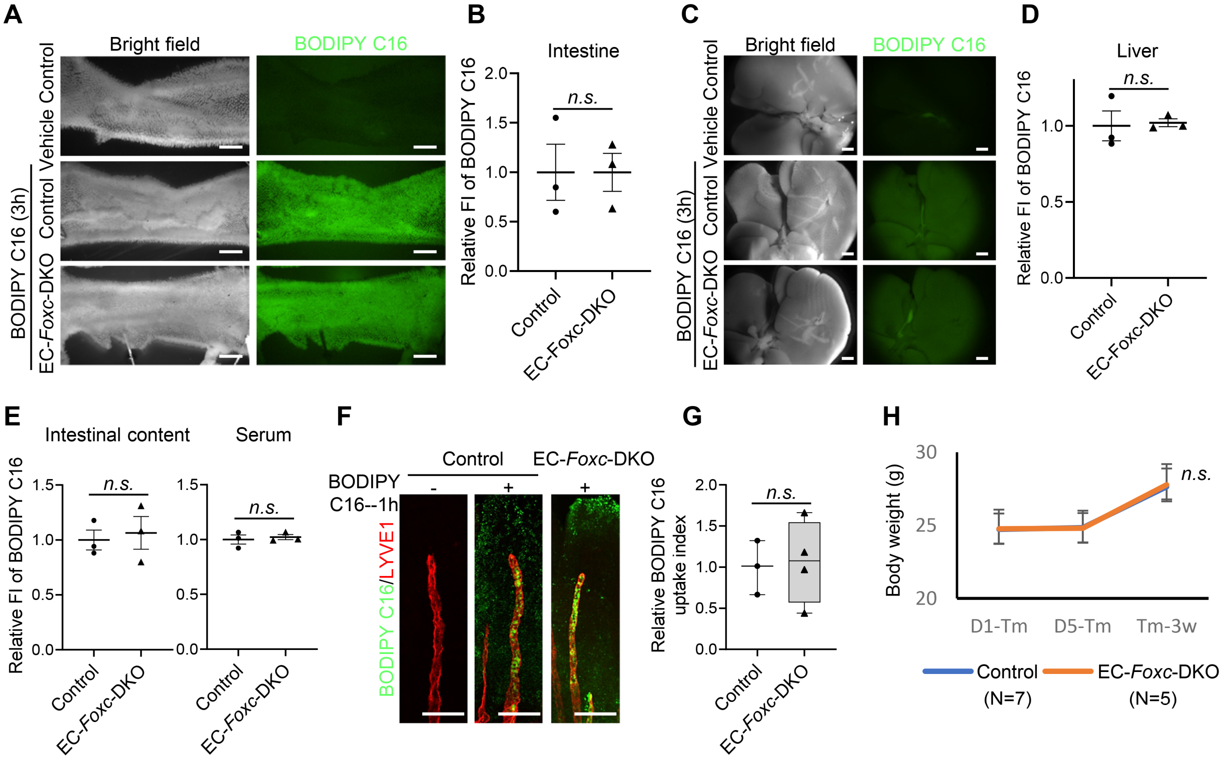

Appendix Figure S1. EC-iKO of *Foxc1/c2* does not affect the lacteal permeability. Mice were treated with 50 µg BODIPY C16 in 200 µL Intralipid by oral gavage after being fasted for 9~12h. Mice treated with Intralipid only were used as vehicle control. 3h after the treatment, the distal jejunum **(A)** and liver **(C)** were dissected and imaged under Nikon AZ100 fluorescent microscope. Scale bars = 1 mm. The quantification of fluorescent intensity (FI) of BODIPY C16 (green) in intestine **(B)** and liver **(D)** was then performed based on the images as shown in A and C. **(E)** Intestinal content and blood serum were also collected 3h post BODIPY dose for the detection of FI of BODIPY. Data are box-and-whisker plots, Mann-Whitney *U* test, each symbol represents one mouse, N = 3, *n.s.* = not significant. **(F)** Representative confocal images of whole-mount intestines immunostained with LYVE1 show the uptake of BODIPY C16 (green) by the lacteals (LYVE1+, red). Scale bars = 50 µm. **(G)** Quantification of the BODIPY C16 uptake index (= ratio of BODIPY C16 intensity in lacteal vs. lacteal area) was performed based on the images as shown in F. Data are box-and-whisker plots, Mann-Whitney *U* test, each symbol represents one mouse, N = 3~4, *n.s.* = not significant. **(H)** Mouse body weight was monitored at Tm-day 1 (D1-Tm), day 5 (D5-Tm) and 3 weeks post Tm treatment (Tm-3w). Data are Mean ± SEM, unpaired *t* test, N=7 in control, N=5 in EC-*Foxc*-DKO, *n.s.* = not significant.

Appendix Figure S2

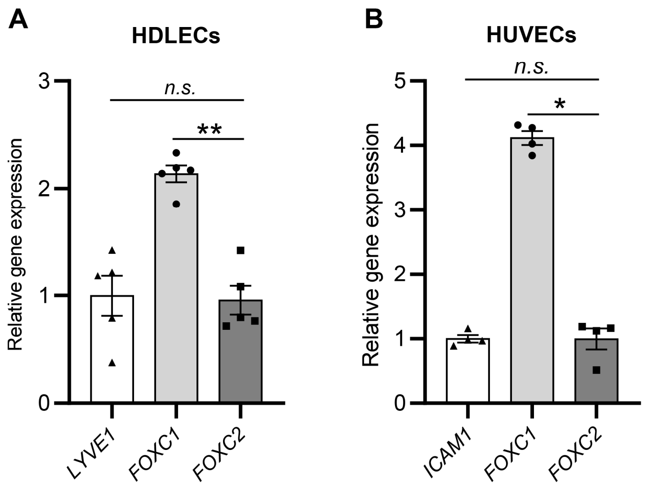

Appendix Figure S2. FOXC1 and FOXC2 are expressed in HDLECs and HUVECs. Before the ChIP assay experiment, the expression of *FOXC1* and *FOXC2* in **(A)** HDLECs and **(B)** HUVECs were confirmed by qPCR. Relative mRNA expression of *FOXC1* and *FOXC2* as well as the EC markers (*LYVE1* for HDLECs and *ICAM1* for HUVECs) show *FOXC2* has similar expression level as the EC markers, while the expression level of *FOXC1* is higher than that of *FOXC2* in both cell types. Data are box-and-whisker plots, Mann-Whitney *U* test, each symbol represents one experiment, N = 5 in HDLECs, N = 4 in HUVECs, **P*<0.05, ***P*<0.01, *n.s.* = not significant.

Appendix Figure S3

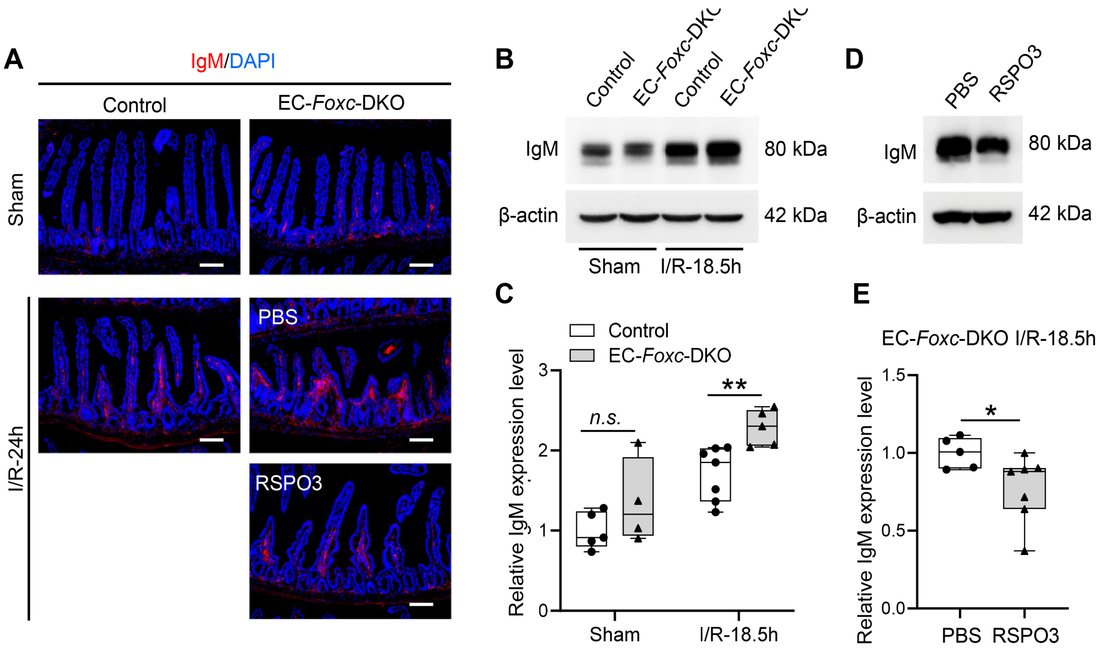

Appendix Figure S3. RSPO3 treatment alleviates IgM accumulation in intestinal mucosa of EC-*Foxc*-DKO mice after I/R. (A) Representative immunostaining images of small intestine labeled with IgM in control and EC-*Foxc*-DKO mice in sham or at 24h after I/R. Note that after I/R, more IgM accumulation in the intestinal mucosa was found in EC-*Foxc*-DKO mice compared with control mice. The accumulation of IgM was alleviated in RSPO3-treated compared with PBS-treated EC-*Foxc*-DKO mice. Paraffin sections (4 μm), scale bars = 100 μm. **(B-E)** Representative Western blots (**B** and **D**) and densitometry measurements (**C** and **E**) show IgM (heavy chain) in intestinal tissue lysates from control and EC-*Foxc*-DKO mice in sham and after I/R at 18.5h (**B** and **C**) as well as IgM (heavy chain) in PBS- and RSPO3- treated EC-*Foxc*-DKO mice after I/R at 18.5h (**D** and **E**). Data are box-and-whisker plots, Mann-Whitney *U* test, each symbol represents one mouse, N = 4~7, *P<0.05, **P<0.01, *n.s.*=not significant.

Appendix Figure S4

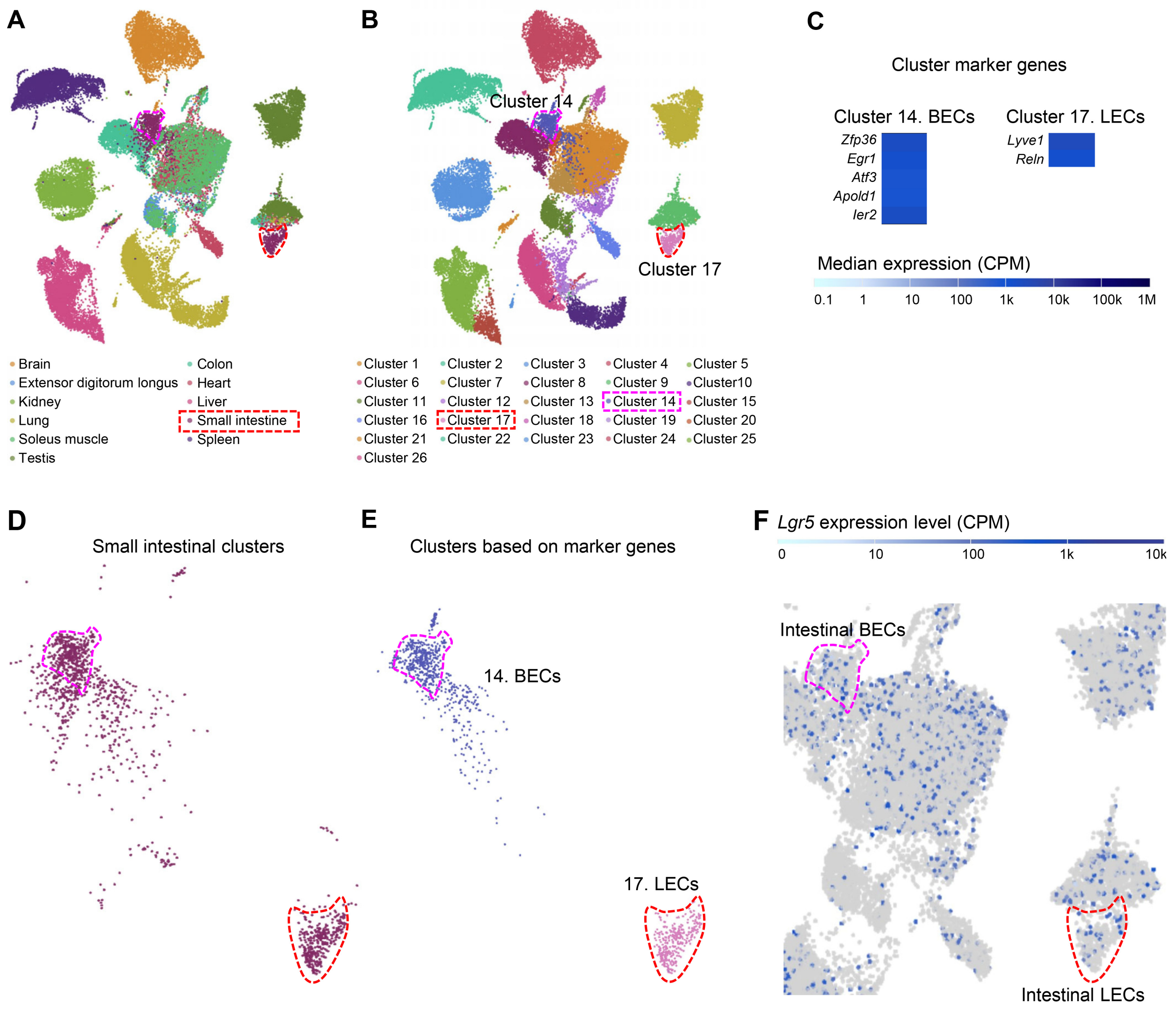

Appendix Figure S4. *Lgr5* is expressed in both intestinal BECs and LECs by scRNAseq. The scRNAseq data for mouse small intestinal ECs from the publication entitled "Single-Cell Transcriptome Atlas of Murine Endothelial Cells" (PMID: 32059779) was examined. The data is available from the website of "Single Cell Expression Atlas" (https://www.ebi.ac.uk/gxa/sc/home) and was exported to check *Lgr5* expression in mouse small intestinal ECs. **(A)** Color plot by organism parts. Pink and red circled area indicate the major cell populations from small intestine. **(B)** Color plot by marker genes. Pink (Cluster 14) and red (Cluster 17) circled area indicate the major cell populations from small intestine. **(C)** Cluster 14 and cluster 17 were identified as BECs and LECs respectively according to their marker genes. CPM: counts per million. **(D)** Small intestinal clusters identified from A. **(E)** Small intestinal clusters based on marker genes identified from B. Pink and red circled area in D and E indicate the intestinal major cell populations. **(F)** *Lgr5* expression in small intestinal BECs (pink circled) and LECs (red circled).
