# Supplementary material for: Endothelial FOXC1 and FOXC2 promote intestinal regeneration after ischemia–reperfusion injury
Source: EMBO Rep. 2023 May 8;24(7):e56030. doi: 10.15252/embr.202256030 (PMC10328078; doi:10.15252/embr.202256030)
Supplement: Supplementary file 2 — Expanded View Figures PDF [file EMBR-24-e56030-s007.pdf]

Expanded View Figures

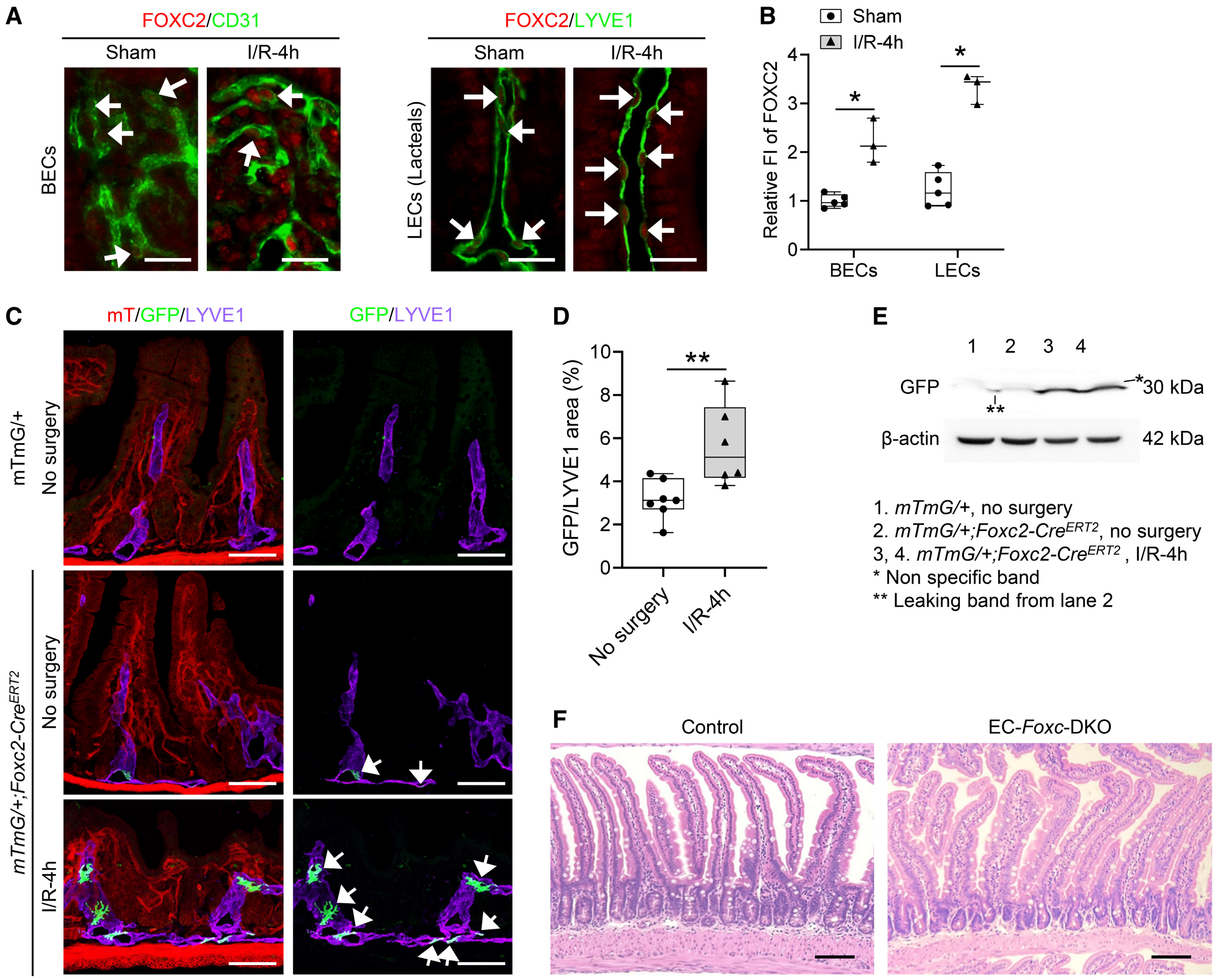

Figure EV1. FOXC2 expression in intestinal ECs.

A Representative immunostaining images of villi show FOXC2 is upregulated in intestinal BECs ($CD31^+$) and LECs ($LYVE1^+$) indicated by arrows after I/R at 4 h in control adult mice (*Foxc1*$^{f/f}$;*Foxc2*$^{f/f}$). Scale bars = 20 μm.

B Quantification of fluorescent intensity (FI) of FOXC2 in BECs and LECs was performed based on the IHC staining as shown in Fig EV1A. Data are box-and-whisker plots, Mann–Whitney *U* test, each symbol represents one mouse, $N = 3 \sim 5$, $^*P < 0.05$.

C Mice were treated with Tm for 5 days and subjected to intestinal I/R surgery 12 day post-Tm treatment. Representative intestinal mucosal images of GFP/LYVE1 immunostaining with mT signals on frozen sections (15 μm) in *mTmG/+;Foxc2-Cre*^{ERT2} mice without surgery or 4 h after I/R. *mTmG/+* mice without surgery were used as control. FOXC2-GFP^+ cells (arrows) were found mainly in $LYVE1^+$ lymphatic vessels. The increased number of FOXC2-GFP^+ cells found in LECs after I/R suggested the proliferation of FOXC2-GFP^+ LECs induced by I/R. Scale bars = 100 μm.

D Quantification of the density of GFP^+ cells in lymphatic vessels. GFP/LYVE1 area (%) = (GFP^+ area)/($LYVE1^+$ area) × 100%. Data are box-and-whisker plots, Mann–Whitney *U* test, each symbol represents one mouse, $N = 6 \sim 7$, $^{**}P < 0.01$.

E Representative western blots show the increased level of FOXC2-GFP in intestinal lysates of *mTmG/+;Foxc2-Cre*^{ERT2} mice 4 h after I/R compared with the mice without surgery.

F Representative H&E staining images of intestinal mucosa in control and EC-*Foxc*-DKO mice after Tm treatment without surgery. Scale bars = 100 μm.

Data information: The box-and-whisker plots in (B) and (D) display the median value (central band in the box), second and third quartiles (bottom and top ends of the box, respectively), as well as minimum/maximum values (whiskers blow/above the box) of the data sets.
Source data are available online for this figure.

 © 2023 The Authors

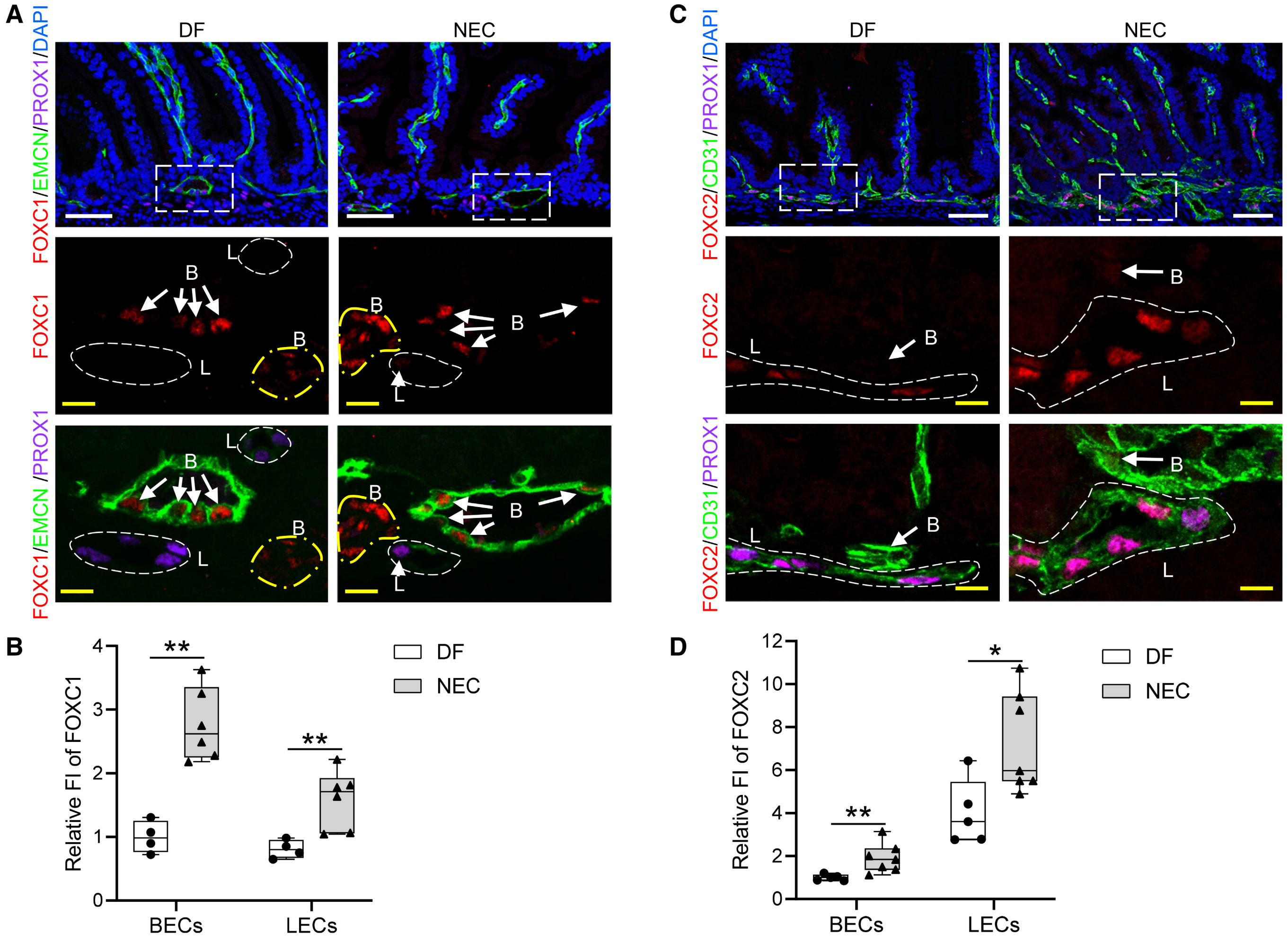

Figure EV2. FOXC1 and FOXC2 are increased in BECs and LECs in mouse NEC model.

A–D Immunostaining of (A) FOXC1/EMCN/PROX1/DAPI and (C) FOXC2/CD31/PROX1/DAPI was performed on paraffin sections (4 μm) of small intestines from 2-day old neonatal mice 24 h after being subjected to the necrotizing enterocolitis (NEC) protocol (Tian *et al*, 2010). Dam-fed (DF) pup littermates were used as control. (A) FOXC1 is mainly expressed in BECs in intestinal submucosa. The level of FOXC1 is increased obviously in submucosal BECs ($EMCN^+$, B with arrow; as well as yellow circled area) in NEC intestine compared with DF intestine. $FOXC1^+$ cells in yellow circled area are arterial BECs with $EMCN^-$. FOXC1 can be found weakly expressed in the submucosal LECs (L with arrow, $PROX1^+$) in NEC intestine but is hardly detectable in LECs in DF intestine. PROX1 is a nuclear marker for LECs. White/yellow scale bars = 50 or 10 μm, respectively. (C) FOXC2 can be detected in LECs (L, $CD31^+PROX1^+$; circled) in DF intestine, and is increased in LECs in NEC intestine compared with DF intestine. FOXC2 can be found weakly expressed in the submucosal BECs (B, $CD31^+PROX1^-$) in NEC intestine (white arrow) but is hardly detectable in BECs in DF intestine. White/yellow scale bars = 50 or 10 μm, respectively. Quantification of fluorescent intensity (FI) of (B) FOXC1 and (D) FOXC2 in submucosal BECs and LECs was performed based on the IHC staining as shown in Fig EV2A and C, respectively. Data are box-and-whisker plots, Mann–Whitney *U* test, each symbol represents one mouse, $N = 4 \sim 7$, $^*P < 0.05$, $^{**}P < 0.01$. The box-and-whisker plots display the median value (central band in the box), second and third quartiles (bottom and top ends of the box, respectively), as well as minimum/maximum values (whiskers blow/above the box) of the data sets.

Source data are available online for this figure.

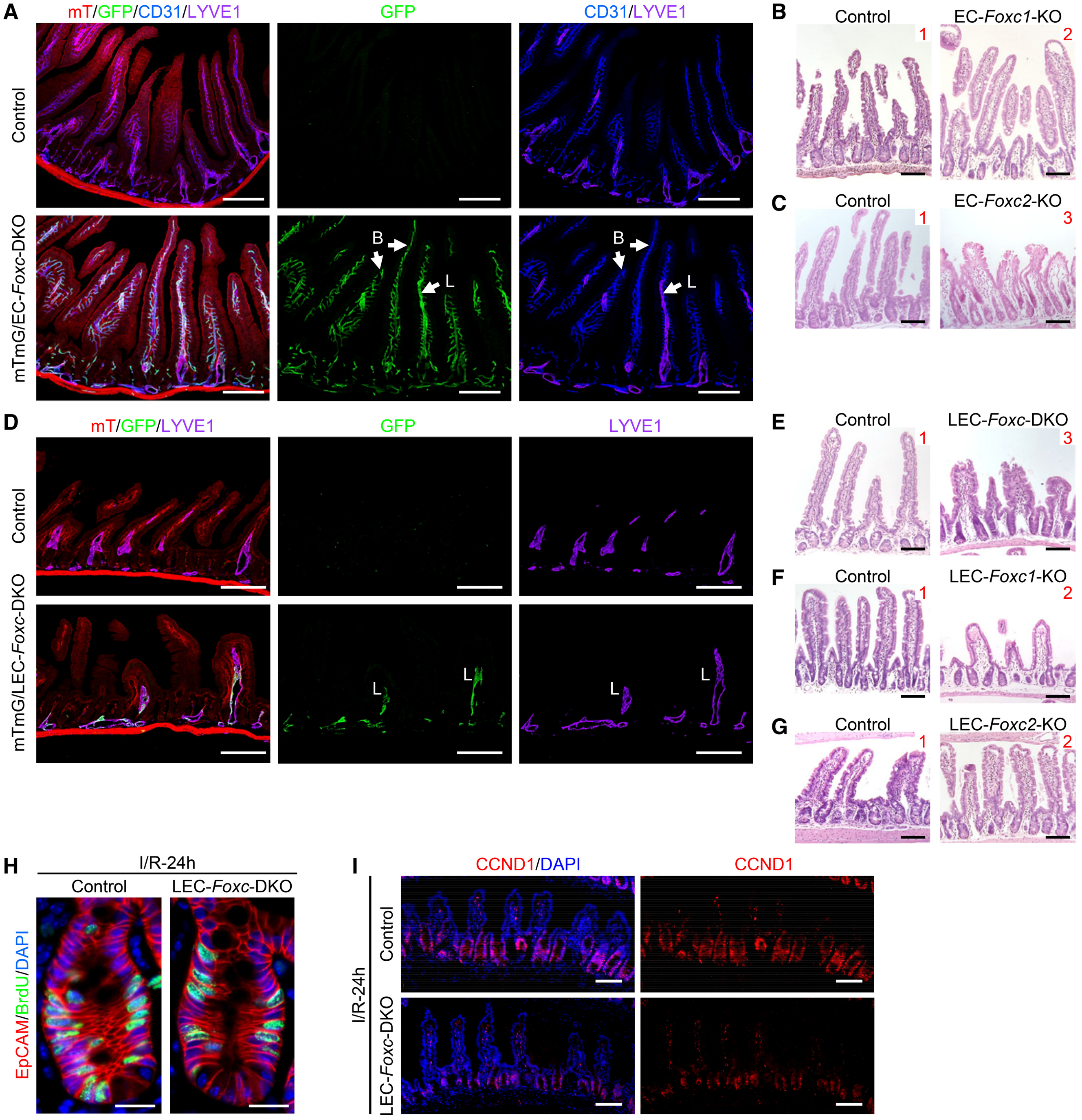

Figure EV3.

 © 2023 The Authors

Figure EV3. Histological detection in EC- and LEC- specific *Foxc* KO mouse intestines.

A–G (A and D) Cre recombination efficiency detection in EC-*Foxc*-DKO and LEC-*Foxc*-DKO mouse strains. (A) Mice (control: *mTmG/+;Foxc1*$^{f/f}$*;Foxc2*$^{f/f}$, mTmG/EC-*Foxc*-DKO: *mTmG/+;Cdh5-Cre*^{ERT2}*;Foxc1*$^{f/f}$*;Foxc2*$^{f/f}$) were treated with 150 mg/kg Tm by oral gavage for 5 days. Seven days after Tm treatment, the distal jejunum was collected and the frozen sections (15 μm) were stained with GFP/CD31/LYVE1 for the detection of GFP signal in blood vessels (B, CD31$^+$LYVE1$^-$, blue) and lacteals (L, CD31$^+$LYVE1$^+$, purple). Scale bars = 200 μm. (D) Mice (control: *mTmG/+;Foxc1*$^{f/f}$*;Foxc2*$^{f/f}$, mTmG/LEC-Foxc-DKO: *mTmG/+;Vegfr3-Cre*^{ERT2}*;Foxc1*$^{f/f}$*;Foxc2*$^{f/f}$) were treated with 150 mg/kg tamoxifen by oral gavage for 5 days. Twelve days after Tm dose, the distal jejunum was collected and the frozen sections (15 μm) were stained with GFP and LYVE1 antibodies. Confocal images show the VEGFR3-GFP is expressed in the LYVE1$^+$ lymphatic vessels (L). Scale bars = 200 μm. (B, C, E–G) Representative H&E staining images of the distal jejuna 24 h after I/R in different mouse strains: (B) EC-*Foxc1*-KO, (C) EC-*Foxc2*-KO, (E) LEC-*Foxc*-DKO, (F) LEC-*Foxc1*-KO, (G) LEC-*Foxc2*-KO and their control mice. The intestinal ischemic injury grading in the Chiu scoring system is indicated by red numbers (0 ~ 5). Scale bars = 100 μm. The quantification of Chiu Score for these mouse strains is shown in Figs 2D and E, and 3B–D, respectively.

H Representative immunostaining images of intestinal crypts labeled with BrdU (proliferative marker, injection performed 2 h before tissue collection) and EpCAM (epithelial marker) show the proliferative epithelial cells in crypts. Paraffin sections (4 μm), scale bars = 20 μm. The quantification of the number of BrdU$^+$ epithelial cells per crypt is shown in Fig 3E.

I Representative images of intestinal mucosa labeled with Cyclin D1 (CCND1) in LEC-*Foxc*-DKO mice compared with the control group 24 h after I/R. Scale bars = 100 μm. Quantification data for CCND1$^+$ epithelial cells per crypt are shown in Fig 3I.

Source data are available online for this figure.

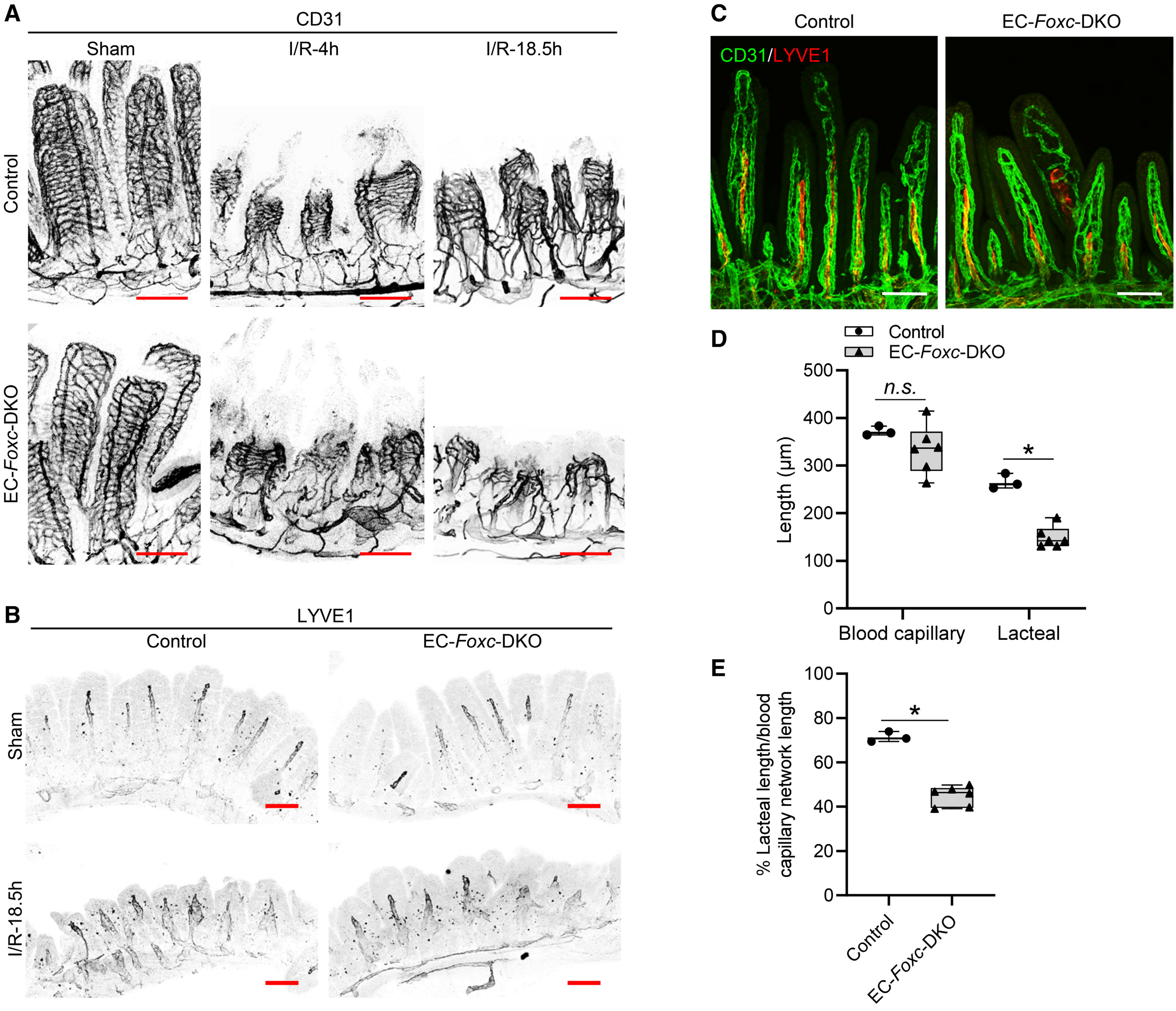

Figure EV4.

Figure EV4. EC-iKO of *Foxc1/c2* results in severe damage of blood and lymphatic vasculatures in intestinal villi after I/R.

A, B Representative images of whole-mount distal jejuna stained with CD31 (A) and LYVE1 (B) show the damage of blood (A) and lymphatic vasculatures (B) in control and EC-*Foxc*-DKO villi at 4 h and/or 18.5 h after I/R. Scale bars = 100 μm.

C–E (C) Representative whole-mount proximal jejuna immunostained with CD31 (green) and LYVE1 (red) in neonatal mice treated with Tm from P1 to P5 and euthanized at P7. Proximal jejuna were collected from neonatal mice due to the ease of operation and similar lacteal length/blood capillary network length ratio between proximal and distal jejuna. The length of blood capillary vasculature and lacteals were measured (D) based on Fig EV4C. The percentage (%) of the lacteal length/blood capillary network length was then calculated (E). Data are box-and-whisker plots, Mann–Whitney *U* test, each symbol represents one mouse, $N = 3 \sim 6$, $^*P < 0.05$, *n.s.*, not significant. The box-and-whisker plots display the median value (central band in the box), second and third quartiles (bottom and top ends of the box, respectively), as well as minimum/maximum values (whiskers blow/above the box) of the data sets.

Source data are available online for this figure.

 © 2023 The Authors

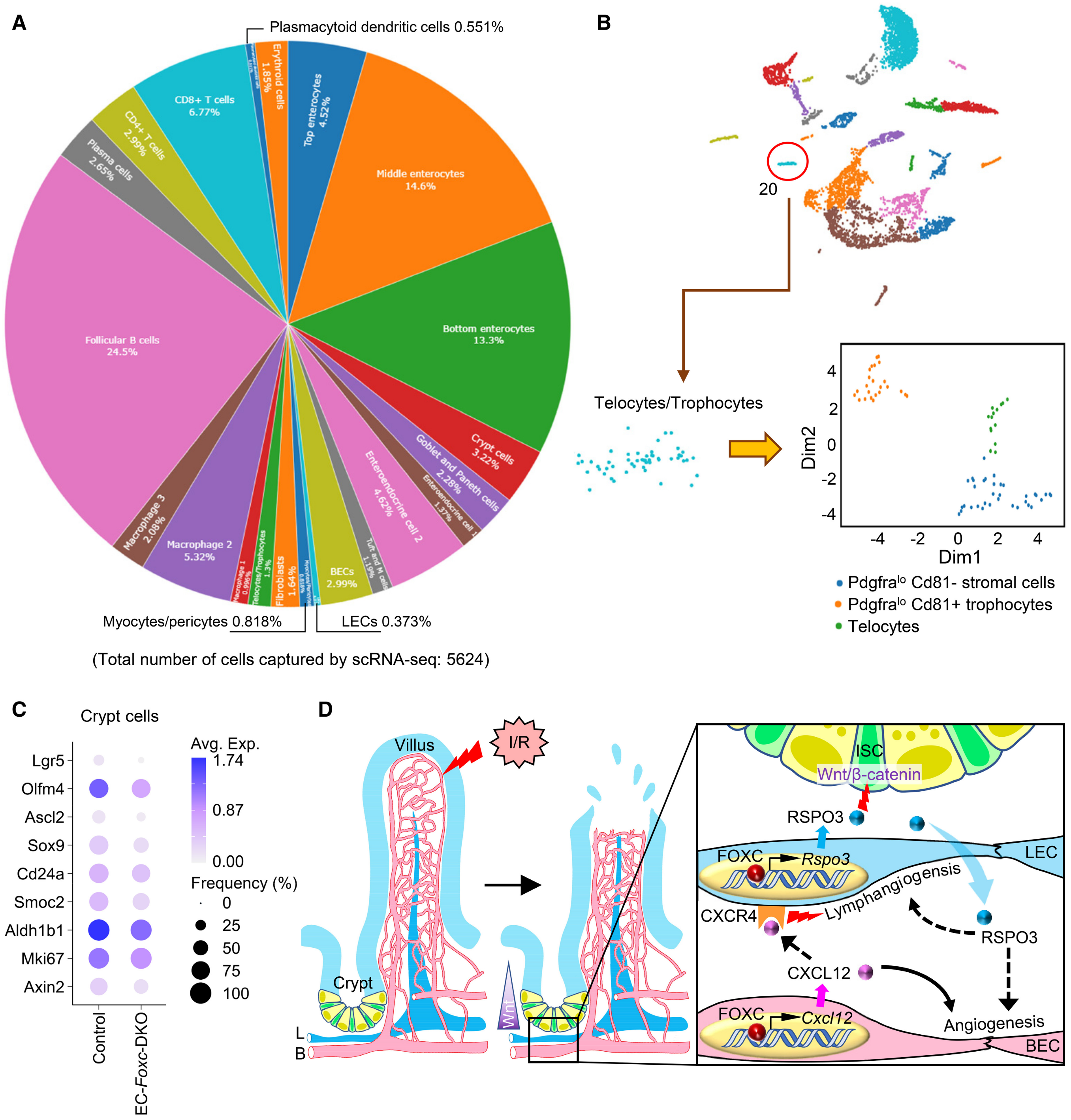
A
Plasmacytoid dendritic cells 0.551%
Erythroid cells 1.85%
Top enterocytes 4.52%
Middle enterocytes 14.6%
Bottom enterocytes 13.3%
Crypt cells 3.22%
Goblet and Paneth cells 2.28%
Enteroendocrine cell 1.37%
Enteroendocrine cell 2 4.62%
Tuft and M cells 1.19%
BECs 2.99%
Fibroblasts 1.64%
Telocytes/Trophocytes 1.3%
Macrophage 1 0.996%
Macrophage 2 5.32%
Macrophage 3 2.08%
Follicular B cells 24.5%
Plasma cells 2.65%
CD4+ T cells 2.99%
CD8+ T cells 6.77%
Myocytes/pericytes 0.818%
LECs 0.373%
(Total number of cells captured by scRNA-seq: 5624)
B
20
Telocytes/Trophocytes
Dim1
Dim2
Pdgfra^lo Cd81- stromal cells
Pdgfra^lo Cd81+ trophocytes
Telocytes
C
Crypt cells
Lgr5
Olfm4
Ascl2
Sox9
Cd24a
Smoc2
Aldh1b1
Mki67
Axin2
Control
EC-Foxc-DKO
Avg. Exp.
1.74
0.87
0.00
Frequency (%)
0
25
50
75
100
D
Villus
I/R
Crypt
L
B
Wnt
ISC
Wnt/β-catenin
RSPO3
FOXC
Rspo3
LEC
Lymphangiogensis
CXCR4
RSPO3
CXCL12
Angiogenesis
FOXC
Cxcl12
BEC

Figure EV5.

Figure EV5. ScRNA-seq analysis on the small intestines from control and EC-*Foxc*-DKO mice 18.5 h after I/R.

A Pie chart showing the percentage of each cell cluster identified in Fig 6A of total cell population.

B Sub-clustering performed on the cluster 20 (Telocytes/Trophocytes).

C Dot plot showing relative expression of different genes identified by scRNA-seq were decreased in crypt cell cluster in EC-*Foxc*-DKO mice compared with control mice at I/R-18.5 h. Fill colors represent normalized mean expression levels and circle sizes represent the within-cluster frequency of positive gene detection. *Lgr5*, *Olfm4*, *Ascl2*, *Sox9*, *Cd24a*, *Smoc2* and *Aldh1b1* are ISC markers. *Mik67* is a proliferative marker. *Ascl2*, *Sox9* and *Axin2* are Wnt/β-catenin target genes.

D Schematic drawing of the mechanism by which endothelial FOXC1 and FOXC2 promote mouse intestinal regeneration after I/R injury. The intestinal mucosa is damaged after I/R injury. FOXC (both FOXC1 and FOXC2) regulate the expression of *Rspo3/Cxcl12* through binding to their regulatory elements in LECs/BECs of the lymphatic/blood vessels near the crypts, respectively. RSPO3 secreted by LECs (blue arrow) is an agonist of the canonical Wnt/β-catenin signaling pathway and promotes the intestinal epithelial regeneration and repair. RSPO3 derived from LECs also promotes angiogenesis and lymphangiogenesis (dashed arrows). BEC-derived CXCL12 (pink arrow) not only regulates angiogenesis (arrow) but also stimulates CXCR4 on LECs to enhance lymphangiogenesis (dashed arrow). L, lymphatic vessel; B, blood vessel; ISC, intestinal stem cell; LEC, lymphatic endothelial cell; BEC, blood endothelial cell.

Source data are available online for this figure.

 © 2023 The Authors
