# Supplementary material for: Endothelial FOXC1 and FOXC2 promote intestinal regeneration after ischemia–reperfusion injury
Source: EMBO Rep. 2023 May 8;24(7):e56030. doi: 10.15252/embr.202256030 (PMC10328078; doi:10.15252/embr.202256030)
Supplement: Supplementary file 3 — Movie EV1 [file EMBR-24-e56030-s008.zip › Movie EV1/Movie EV1 Legend.docx]

**Movie EV1. 3D structures of intestinal blood and lymphatic vasculatures in Control mice 18.5h after I/R**

Representative 3D videos created using IMARIS software based on the confocal images of whole-mount immunostaining of distal jejunums show the 3D structures of intestinal blood capillaries (labeled with CD31, green) and lymphatic vessels (labeled with LYVE1, red) after I/R at 18.5h. In control mouse **(Movie EV1)**, damages found at top of the villous blood vasculatures form openings to the capillary cage, accompanied by endothelial cell projections being formed for the repair. Scale bars = 50 µm. The display rate is 24 frames/second.
