# Supplementary material for: Endothelial FOXC1 and FOXC2 promote intestinal regeneration after ischemia–reperfusion injury
Source: EMBO Rep. 2023 May 8;24(7):e56030. doi: 10.15252/embr.202256030 (PMC10328078; doi:10.15252/embr.202256030)
Supplement: Supplementary file 4 — Movie EV2 [file EMBR-24-e56030-s013.zip › Movie EV2/Movie EV2 Legend.docx]

**Movie EV2. 3D structures of intestinal blood and lymphatic vasculatures in EC-*Foxc*-DKO mice 18.5h after I/R**

Representative 3D videos created using IMARIS software based on the confocal images of whole-mount immunostaining of distal jejunums show the 3D structures of intestinal blood capillaries (labeled with CD31, green) and lymphatic vessels (labeled with LYVE1, red) after I/R at 18.5h. In EC-*Foxc*-DKO mouse **(Movie EV2)**, the intestine loses most of its villi together with the villous blood and lymphatic vessels. The remaining villous, cryptal and submucosal vasculatures are also damaged and less branches can be found in the broken vessels. Lymphatic vessels are dilated in the cryptal area. Scale bars = 50 µm. The display rate is 24 frames/second.
