# Supplementary material for: Endothelial FOXC1 and FOXC2 promote intestinal regeneration after ischemia–reperfusion injury
Source: EMBO Rep. 2023 May 8;24(7):e56030. doi: 10.15252/embr.202256030 (PMC10328078; doi:10.15252/embr.202256030)
Supplement: Supplementary file 5 — Source Data for Expanded View and Appendix [file EMBR-24-e56030-s011.zip › EMBR_202256030T_uncut gel file.pdf]

Full unedited gel for Figure EV1E

GFP

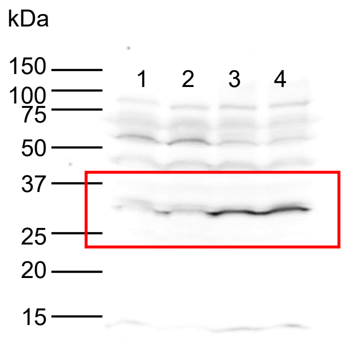

1. *mTmG/+*, no surgery
2. *mTmG/+;Foxc2-Cre*^{ERT2}, no surgery
3, 4. *mTmG/+;Foxc2-Cre*^{ERT2} , I/R-4h

Primary antibody: GFP, Invitrogen A-11122, Rabbit pAb, 1:1000
Secondary antibody: HRP conjugated Goat anti-Rabbit IgG, MilliporeSigma 12348, 1:2000

β-actin

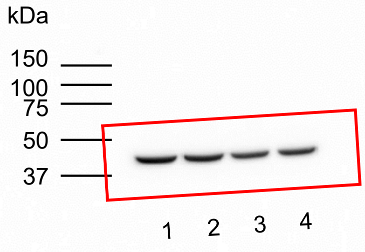

1. *mTmG/+*, no surgery
2. *mTmG/+;Foxc2-Cre*^{ERT2}, no surgery
3, 4. *mTmG/+;Foxc2-Cre*^{ERT2} , I/R-4h

Primary antibody: β-actin, Sigma A1978 , mouse mAb, 1:2000
Secondary antibody: HRP conjugated Goat anti-mouse IgG, ThermoFisher 31430, 1:2500

Full unedited gel for Appendix Figure S3B

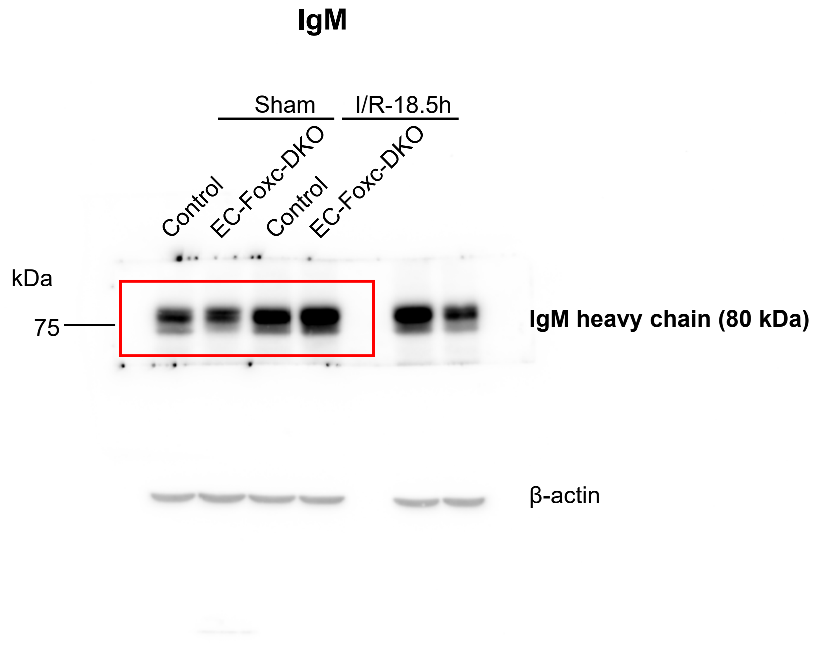

Primary antibody: IgM, Invitrogen 61-6800, Rb pAb, 1:1000
Secondary antibody: HRP conjugated Goat anti-Rabbit IgG, MilliporeSigma 12348, 1:2000

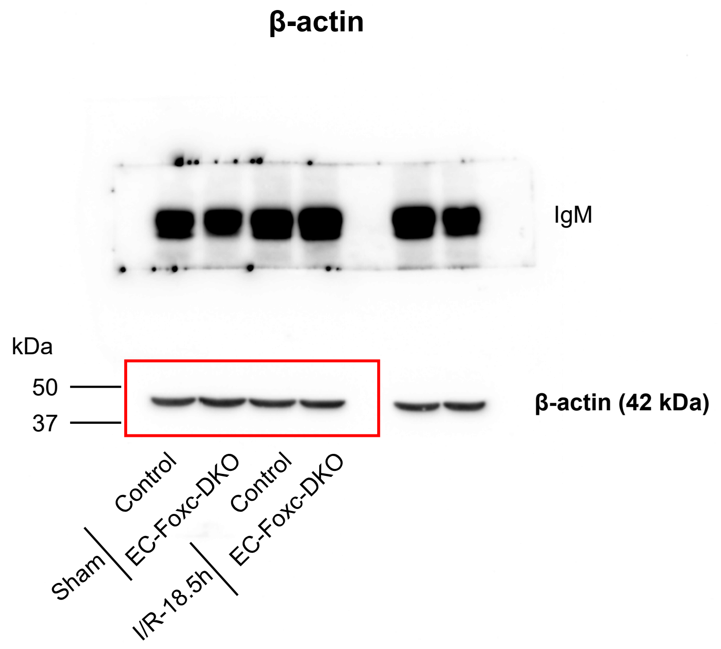

Primary antibody: β-actin, Sigma A1978 , mouse mAb, 1:2000
Secondary antibody: HRP conjugated Goat anti-mouse IgG, ThermoFisher 31430, 1:2500

Full unedited gel for Appendix Figure S3D

IgM

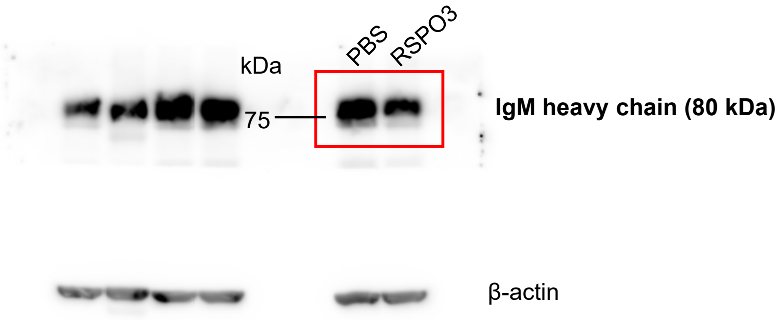

Primary antibody: IgM, Invitrogen 61-6800, Rb pAb, 1:1000
Secondary antibody: HRP conjugated Goat anti-Rabbit IgG, MilliporeSigma 12348, 1:2000

β-actin

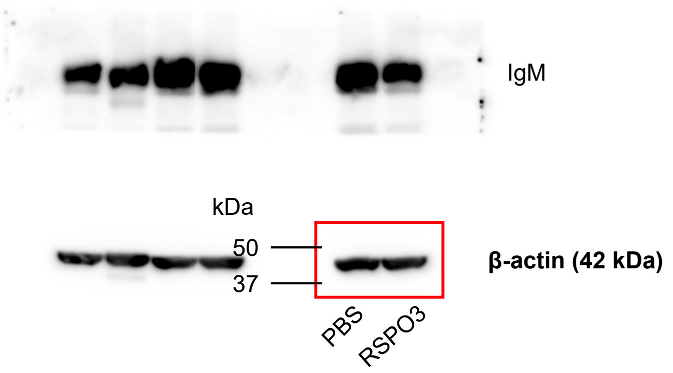

Primary antibody: β-actin, Sigma A1978 , mouse mAb, 1:2000
Secondary antibody: HRP conjugated Goat anti-mouse IgG, ThermoFisher 31430, 1:2500
