# Supplementary figures and images for: Endothelial FOXC1 and FOXC2 promote intestinal regeneration after ischemia–reperfusion injury (part 1 of 3)
Source: EMBO Rep. 2023 May 8;24(7):e56030. doi: 10.15252/embr.202256030 (PMC10328078; doi:10.15252/embr.202256030)

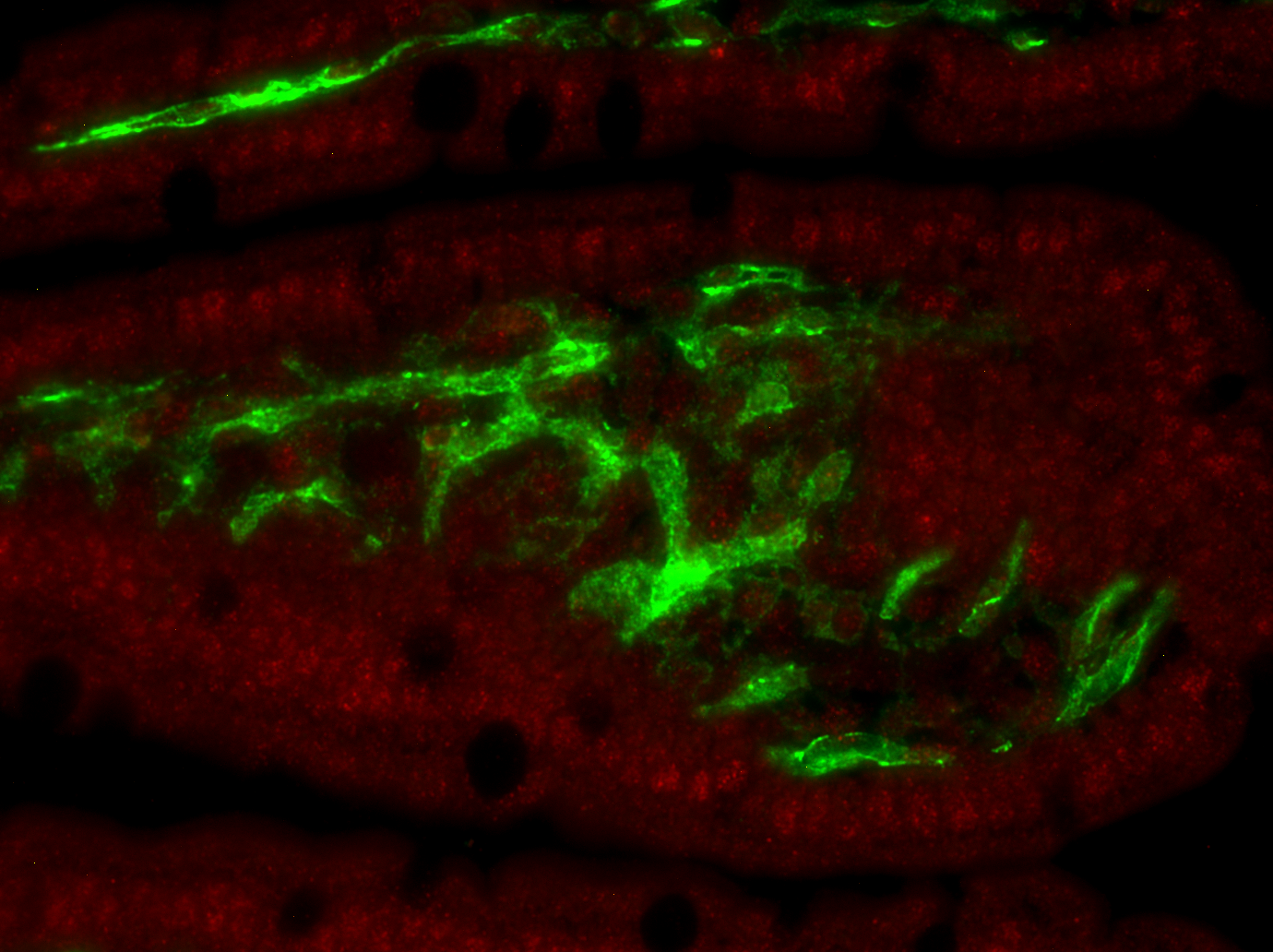

Supplement: Supplementary file 5 — Source Data for Expanded View and Appendix [file EMBR-24-e56030-s011.zip › Figure EV1-5, Appendix Figure S1-4/1. Figure EV1/Figure EV1A-IHC-FOXC2 with CD31 or LYVE1/1. FOXC2 CD31 sham.TIF]

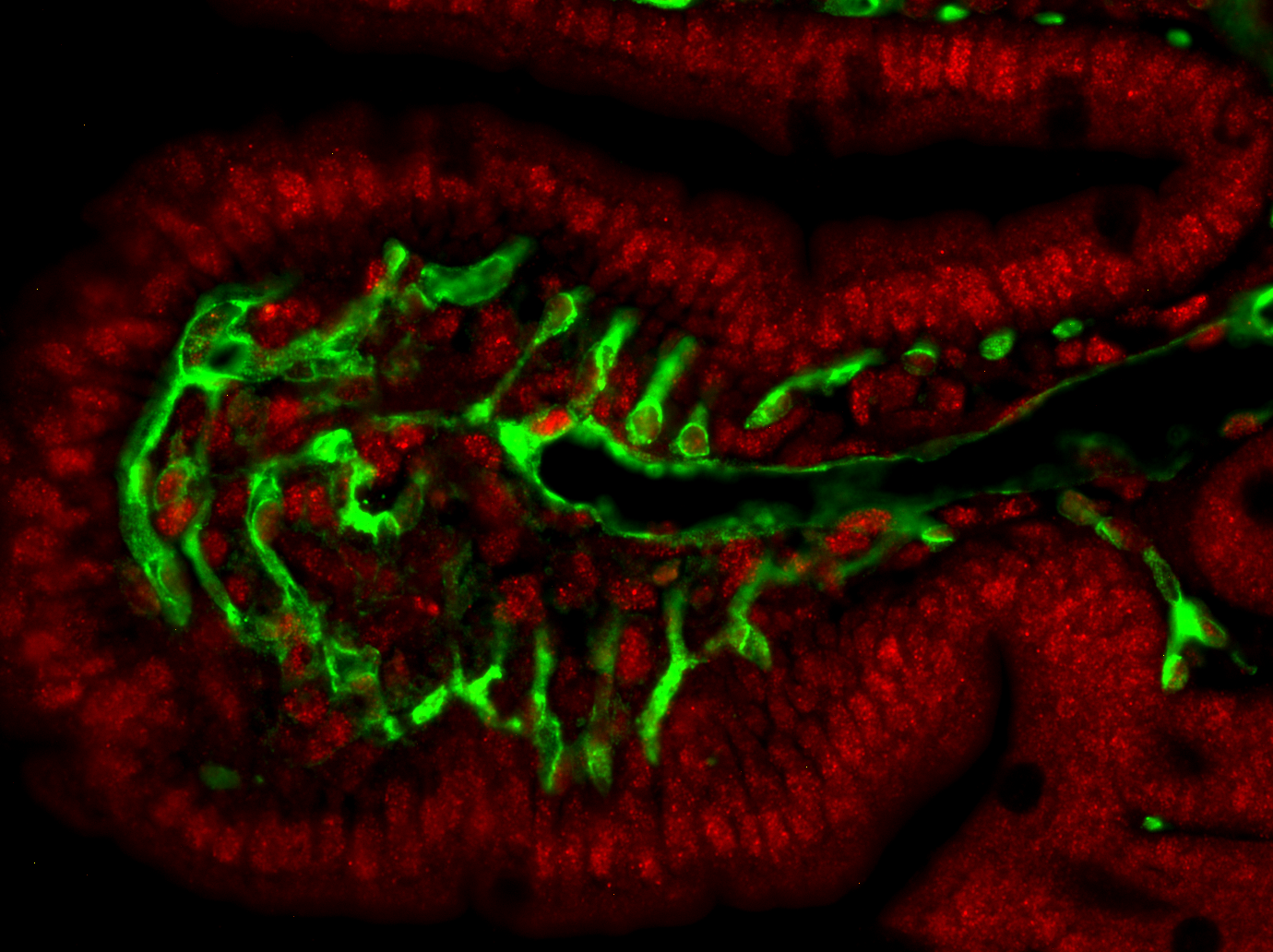

Supplement: Supplementary file 5 — Source Data for Expanded View and Appendix [file EMBR-24-e56030-s011.zip › Figure EV1-5, Appendix Figure S1-4/1. Figure EV1/Figure EV1A-IHC-FOXC2 with CD31 or LYVE1/2. FOXC2 CD31 IR.TIF]

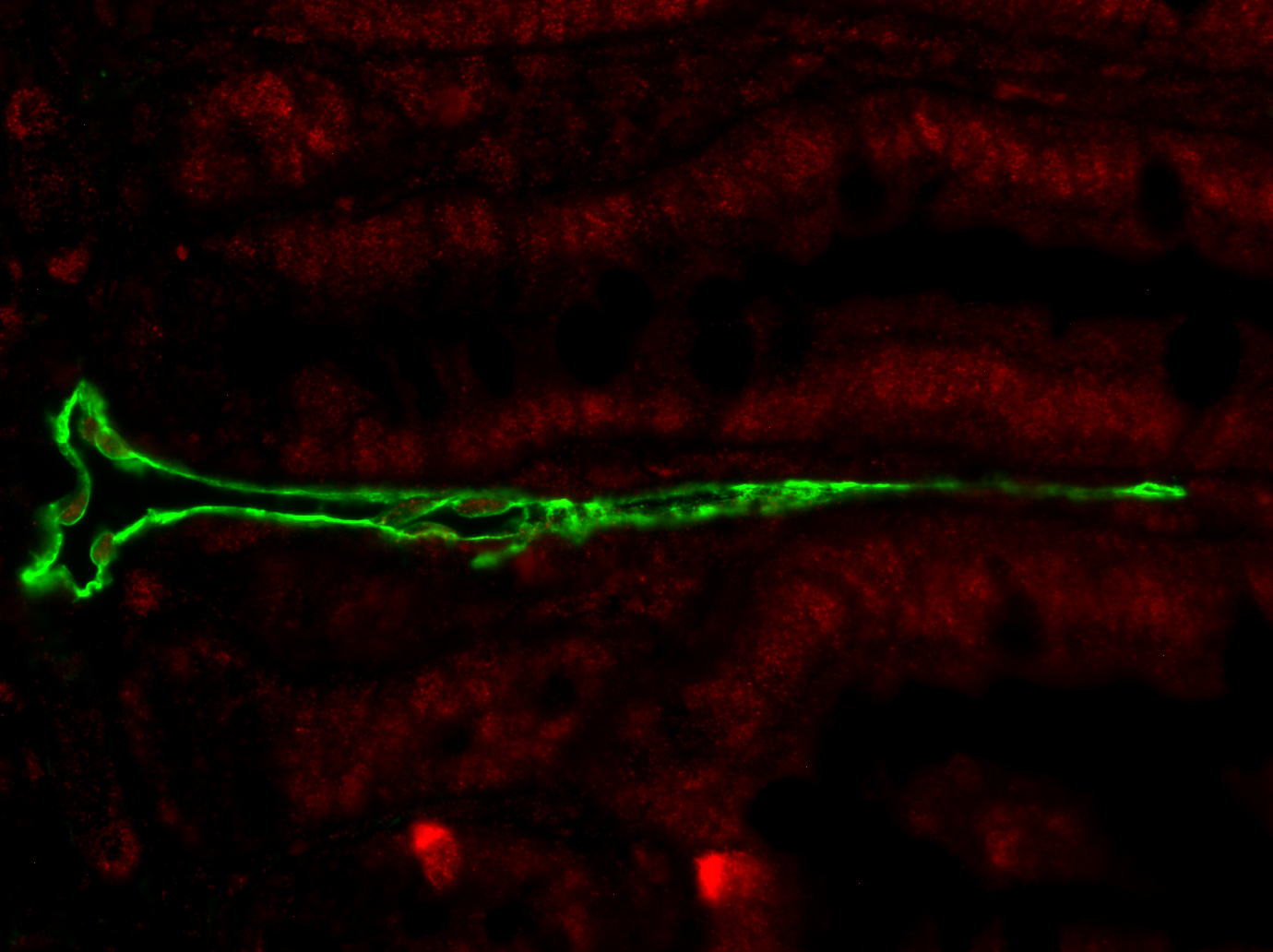

Supplement: Supplementary file 5 — Source Data for Expanded View and Appendix [file EMBR-24-e56030-s011.zip › Figure EV1-5, Appendix Figure S1-4/1. Figure EV1/Figure EV1A-IHC-FOXC2 with CD31 or LYVE1/3. FOXC2 LYVE1 sham.TIF]

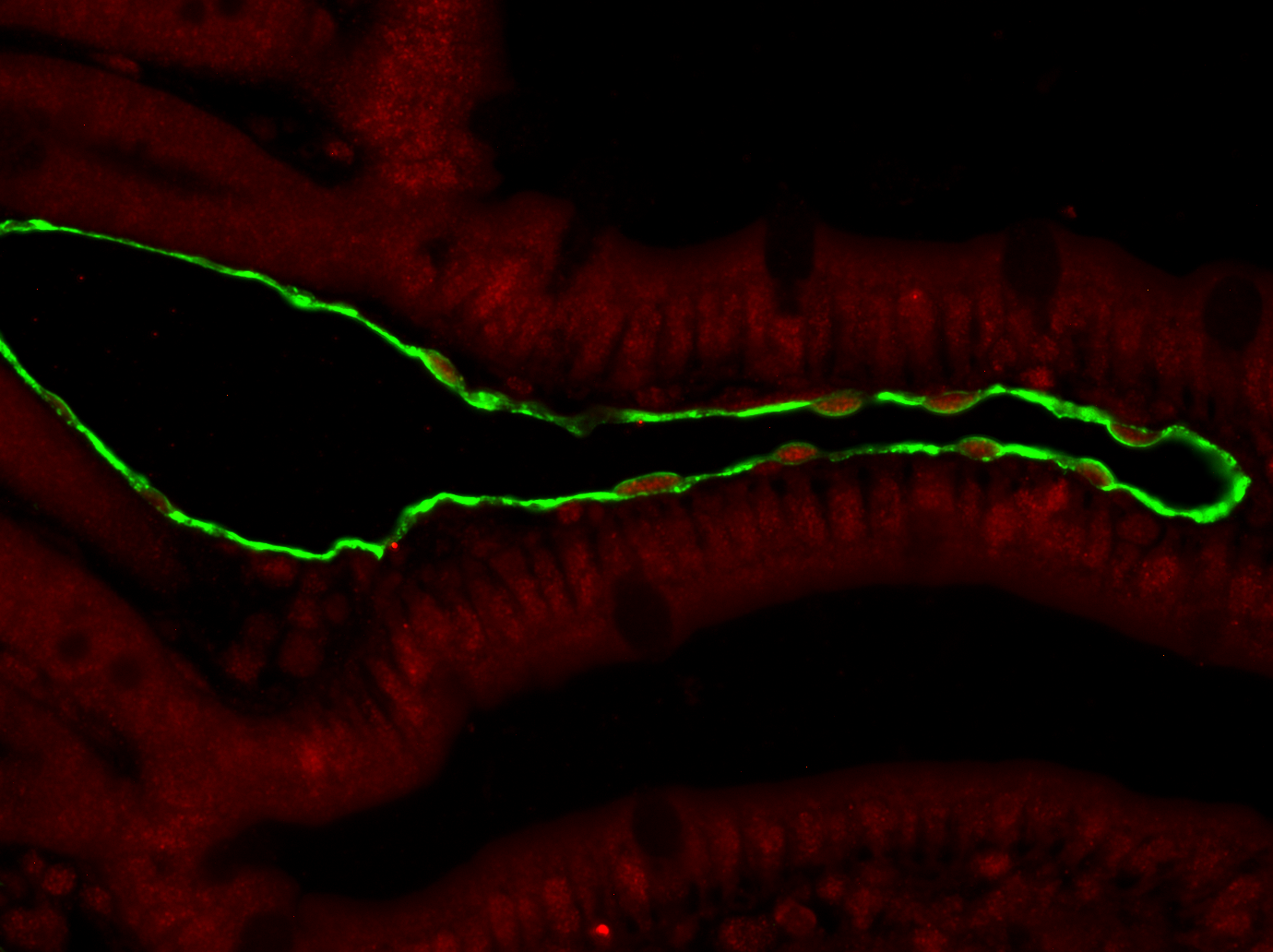

Supplement: Supplementary file 5 — Source Data for Expanded View and Appendix [file EMBR-24-e56030-s011.zip › Figure EV1-5, Appendix Figure S1-4/1. Figure EV1/Figure EV1A-IHC-FOXC2 with CD31 or LYVE1/4. FOXC2 LYVE1 IR.TIF]

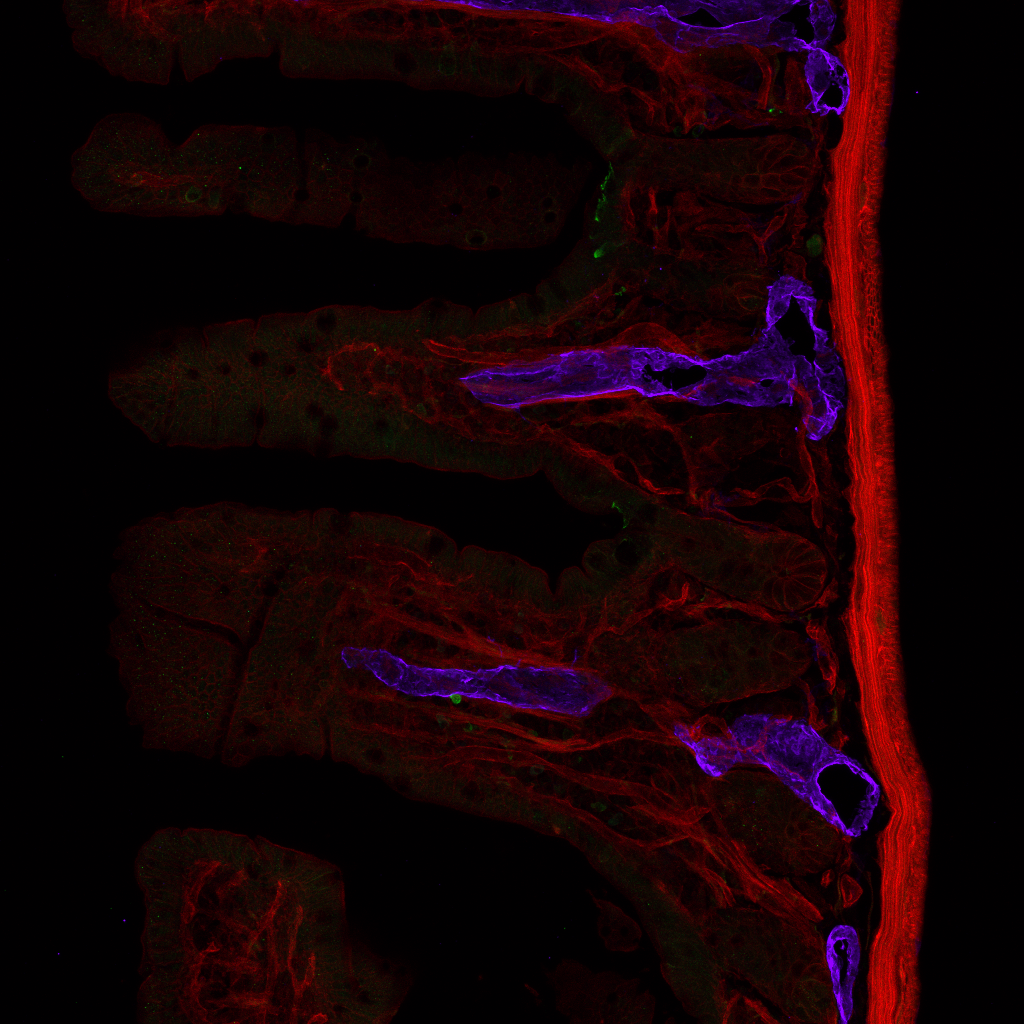

Supplement: Supplementary file 5 — Source Data for Expanded View and Appendix [file EMBR-24-e56030-s011.zip › Figure EV1-5, Appendix Figure S1-4/1. Figure EV1/Figure EV1C-IHC-GFP in Foxc2-Cre;mTmG mice/1. mTmG+, No surgery, 3 colors.tif]

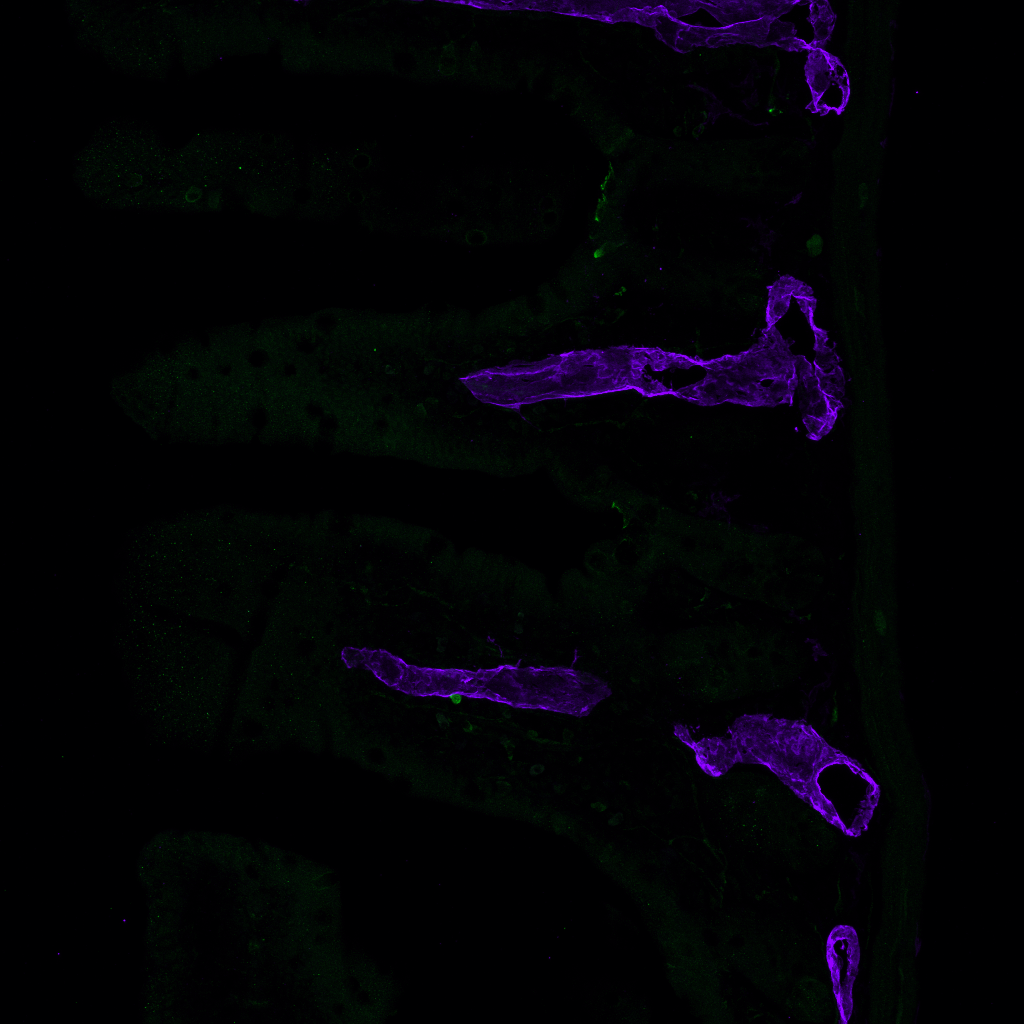

Supplement: Supplementary file 5 — Source Data for Expanded View and Appendix [file EMBR-24-e56030-s011.zip › Figure EV1-5, Appendix Figure S1-4/1. Figure EV1/Figure EV1C-IHC-GFP in Foxc2-Cre;mTmG mice/2. mTmG+, No surgery, LYVE1+GFP.tif]

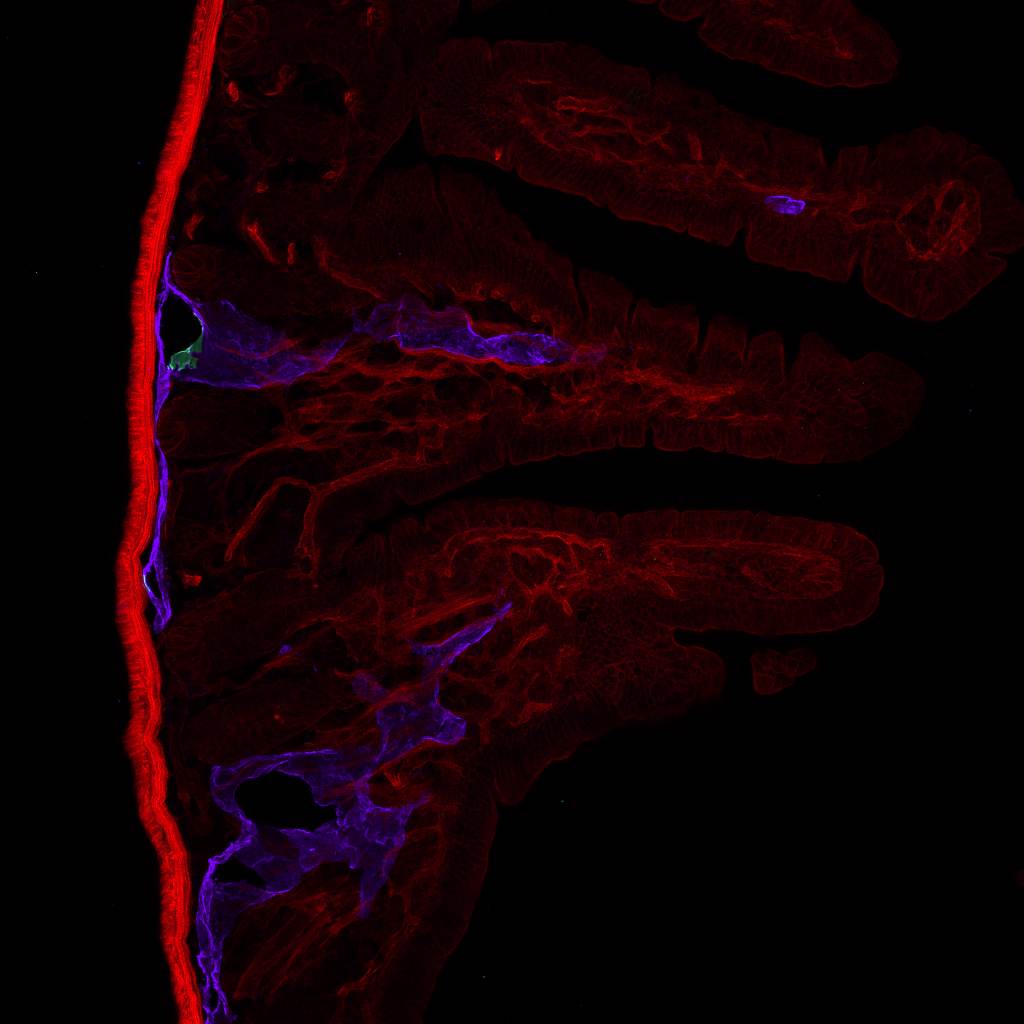

Supplement: Supplementary file 5 — Source Data for Expanded View and Appendix [file EMBR-24-e56030-s011.zip › Figure EV1-5, Appendix Figure S1-4/1. Figure EV1/Figure EV1C-IHC-GFP in Foxc2-Cre;mTmG mice/3. mTmG+;Foxc2-CreERT2, No surgery, 3 colors.tif]

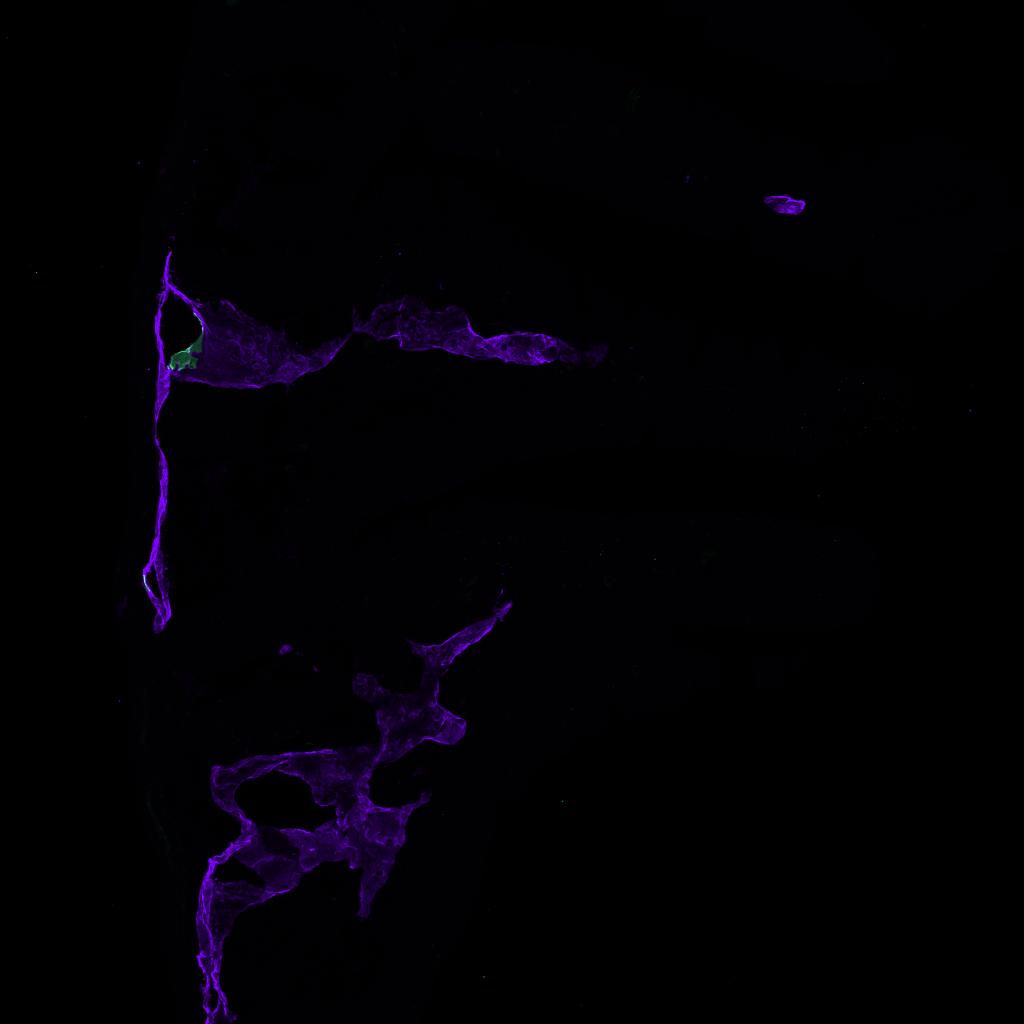

Supplement: Supplementary file 5 — Source Data for Expanded View and Appendix [file EMBR-24-e56030-s011.zip › Figure EV1-5, Appendix Figure S1-4/1. Figure EV1/Figure EV1C-IHC-GFP in Foxc2-Cre;mTmG mice/4. mTmG+;Foxc2-CreERT2, No surgery, LYVE1+GFP.tif]

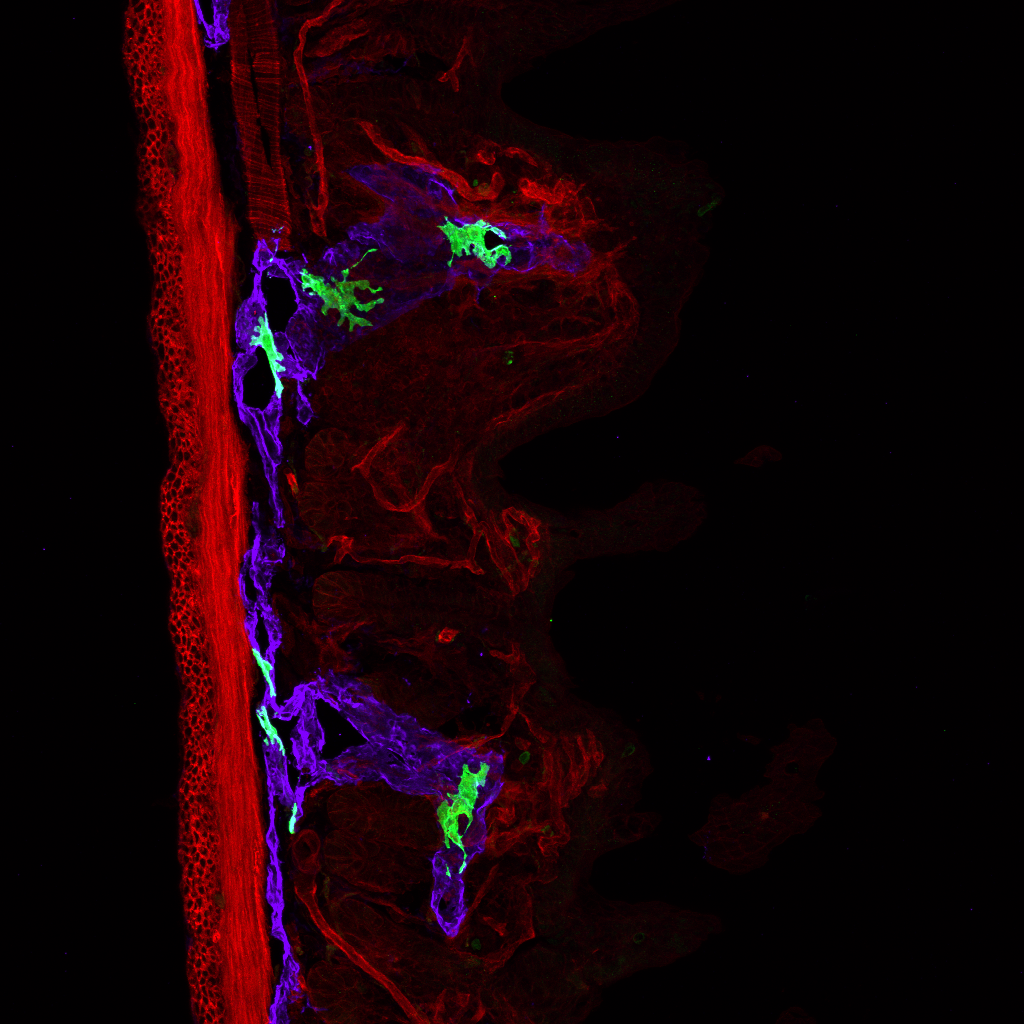

Supplement: Supplementary file 5 — Source Data for Expanded View and Appendix [file EMBR-24-e56030-s011.zip › Figure EV1-5, Appendix Figure S1-4/1. Figure EV1/Figure EV1C-IHC-GFP in Foxc2-Cre;mTmG mice/5. mTmG+;Foxc2-CreERT2, IR-4h, 3 colors.tif]

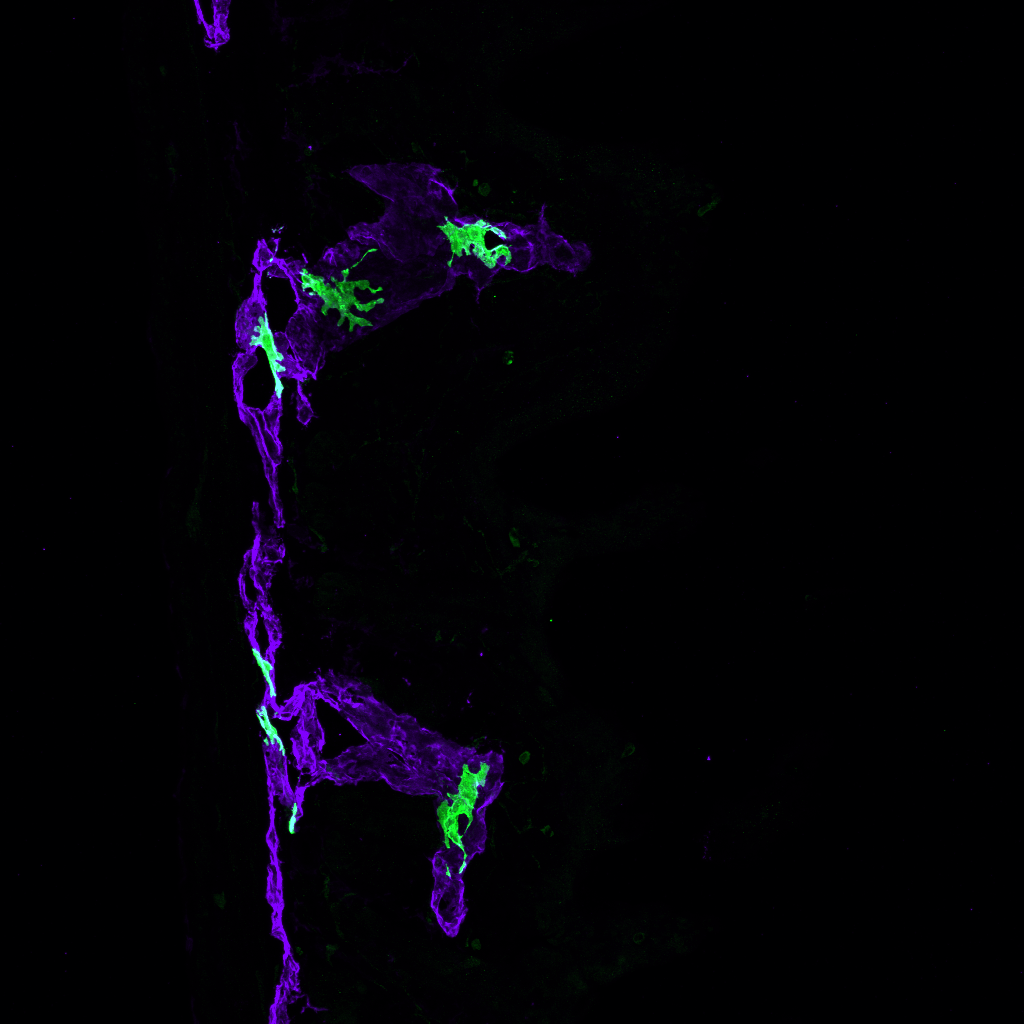

Supplement: Supplementary file 5 — Source Data for Expanded View and Appendix [file EMBR-24-e56030-s011.zip › Figure EV1-5, Appendix Figure S1-4/1. Figure EV1/Figure EV1C-IHC-GFP in Foxc2-Cre;mTmG mice/6. mTmG+;Foxc2-CreERT2, IR-4h, LYVE1+GFP.tif]

## Full unedited gel for Figure EV1E

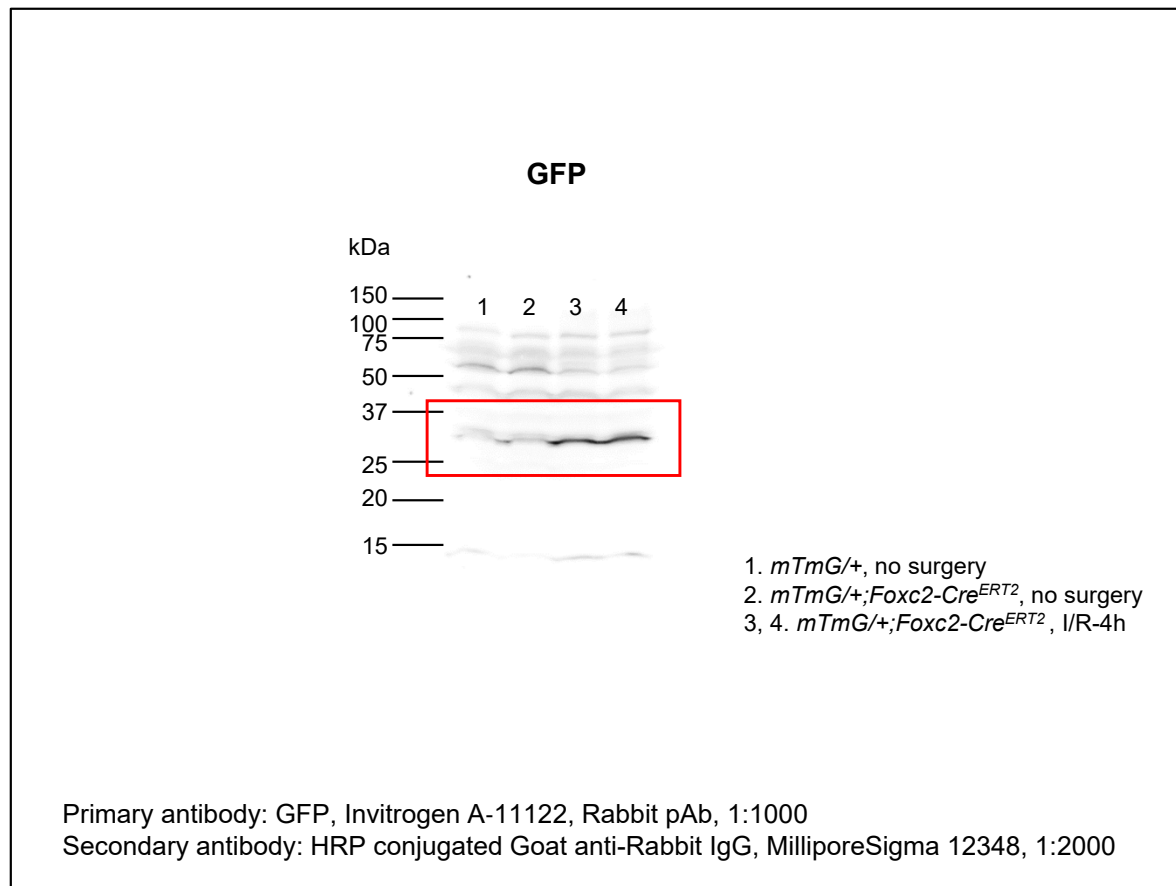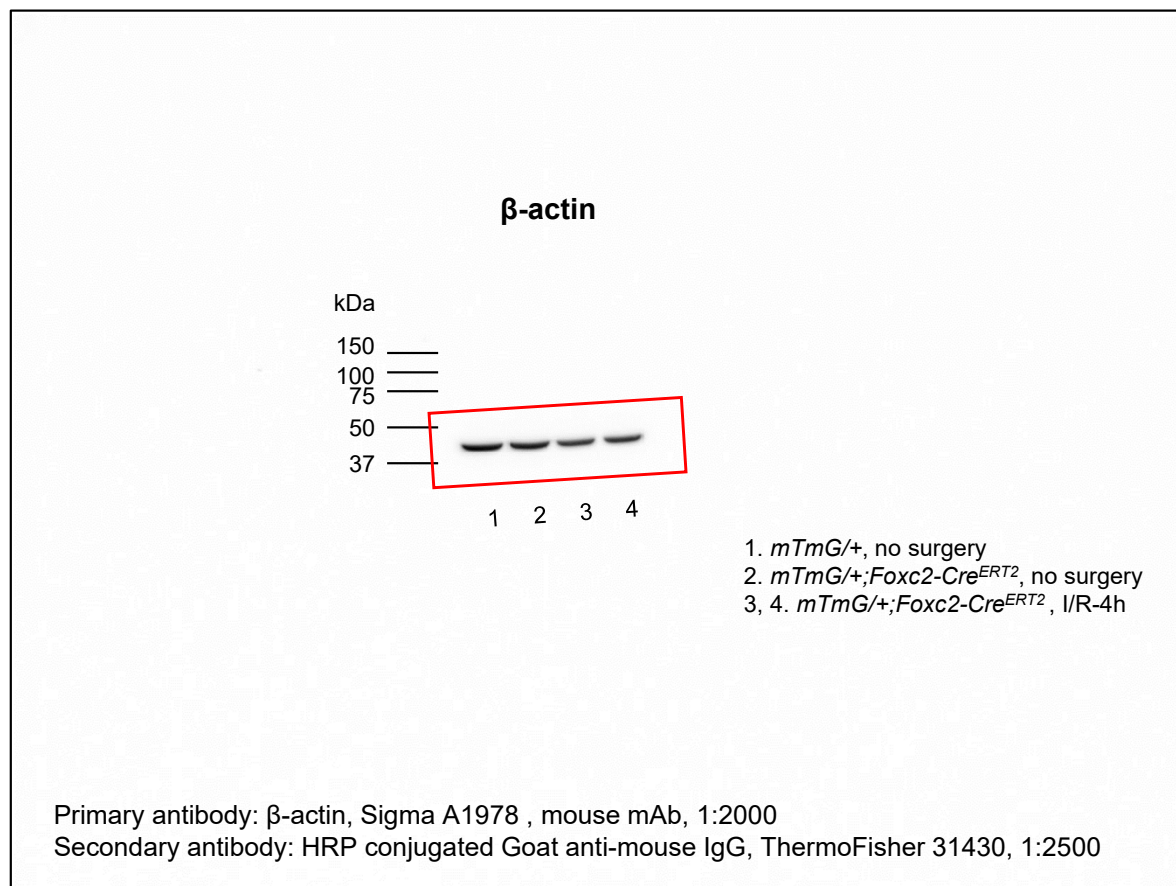

Supplement: Supplementary file 5 — Source Data for Expanded View and Appendix [file EMBR-24-e56030-s011.zip › Figure EV1-5, Appendix Figure S1-4/1. Figure EV1/Figure EV1E-WB, uncut gel file.pdf]

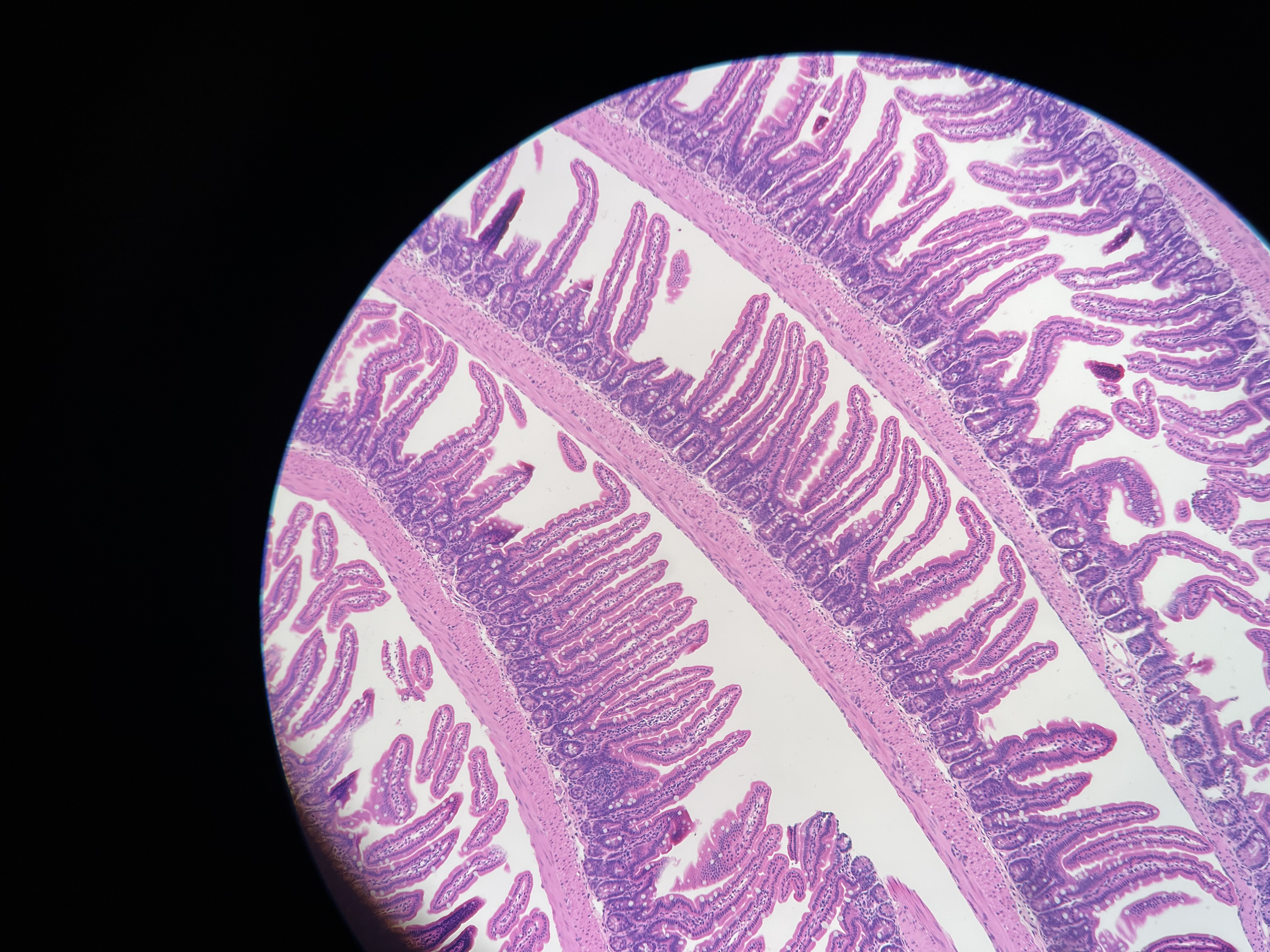

Supplement: Supplementary file 5 — Source Data for Expanded View and Appendix [file EMBR-24-e56030-s011.zip › Figure EV1-5, Appendix Figure S1-4/1. Figure EV1/Figure EV1F-HE for control and EC-Foxc-DKO under homeostasis/1. Control.jpeg]

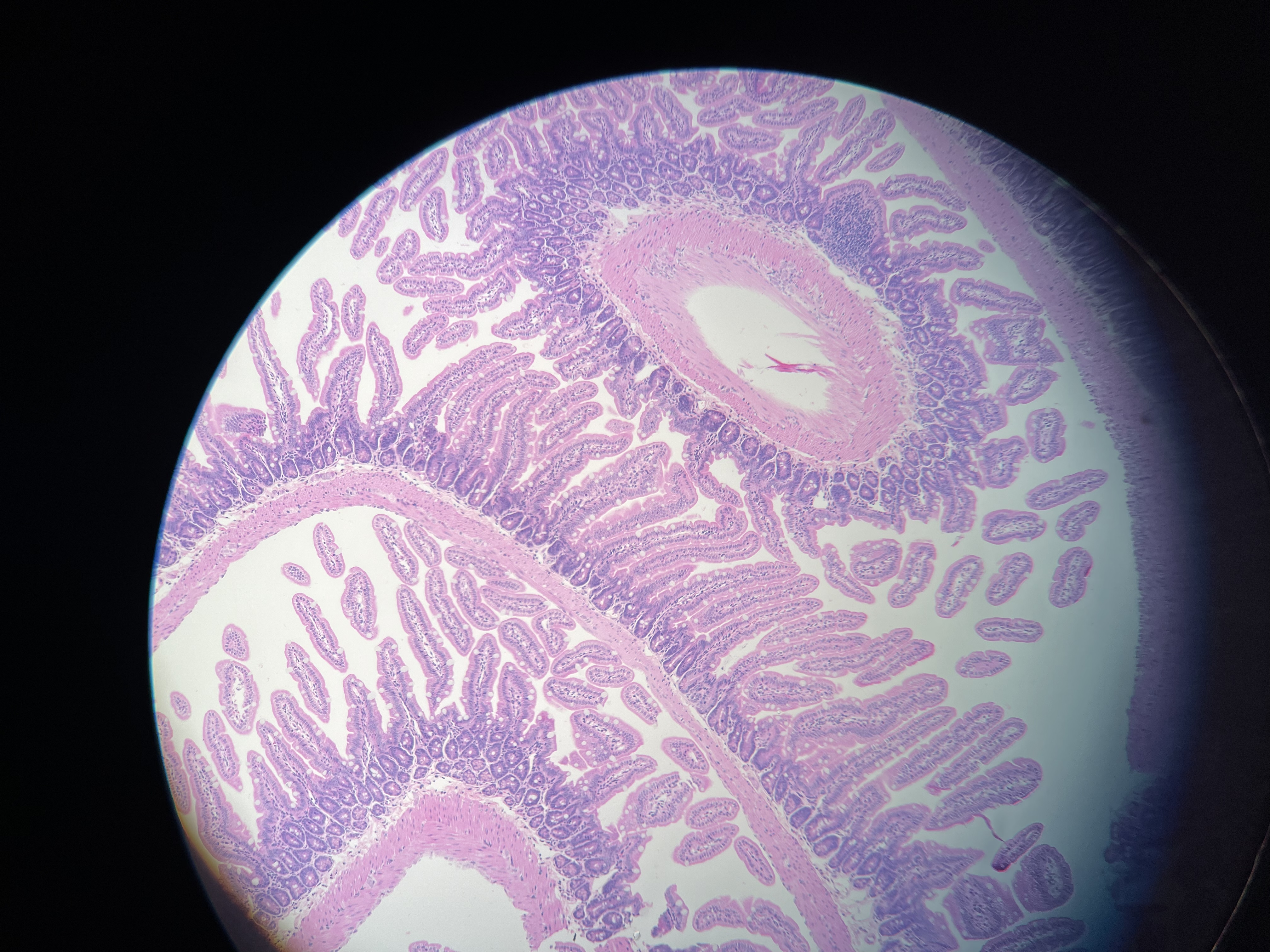

Supplement: Supplementary file 5 — Source Data for Expanded View and Appendix [file EMBR-24-e56030-s011.zip › Figure EV1-5, Appendix Figure S1-4/1. Figure EV1/Figure EV1F-HE for control and EC-Foxc-DKO under homeostasis/2. EC-Foxc-DKO.jpeg]

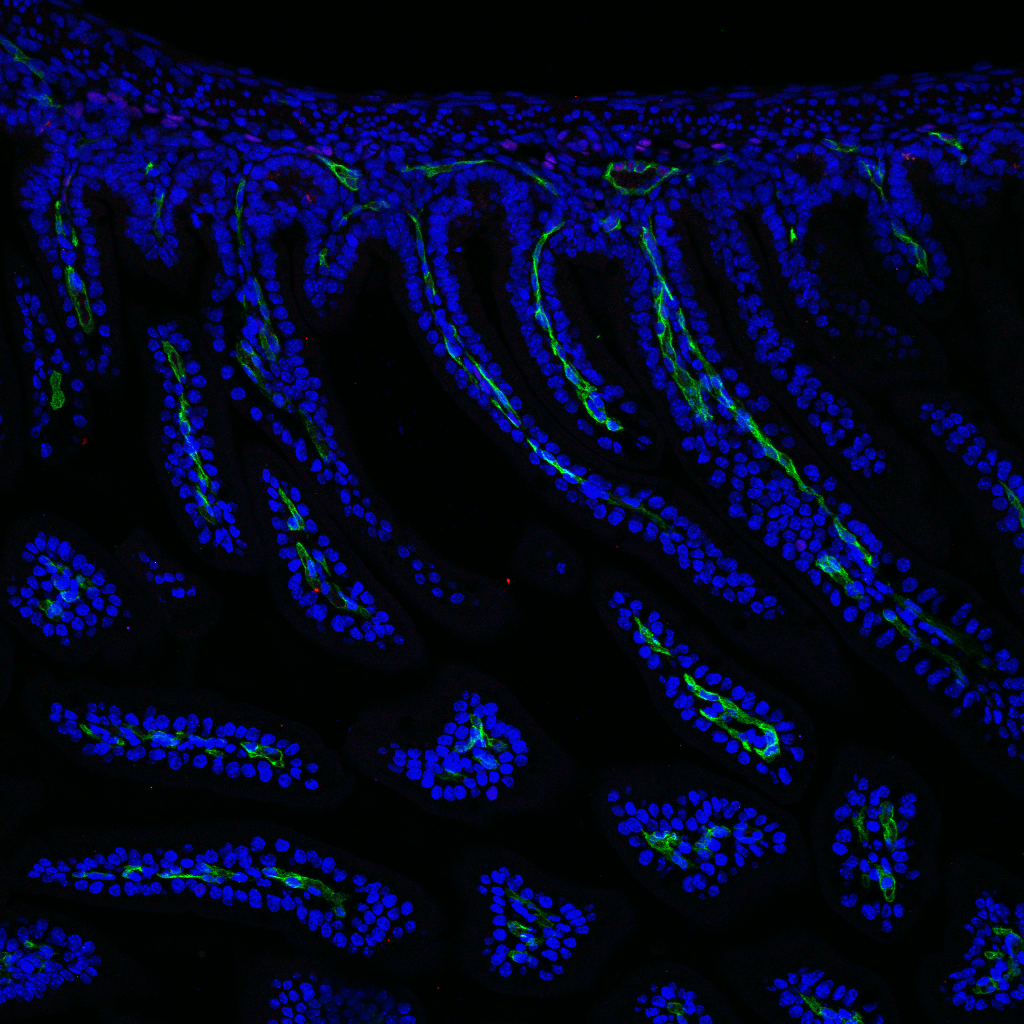

Supplement: Supplementary file 5 — Source Data for Expanded View and Appendix [file EMBR-24-e56030-s011.zip › Figure EV1-5, Appendix Figure S1-4/2. Figure EV2/Figure EV2A-IHC FOXC1 EMCN PROX1 in NEC model/DF-1, 4 colors.tif]

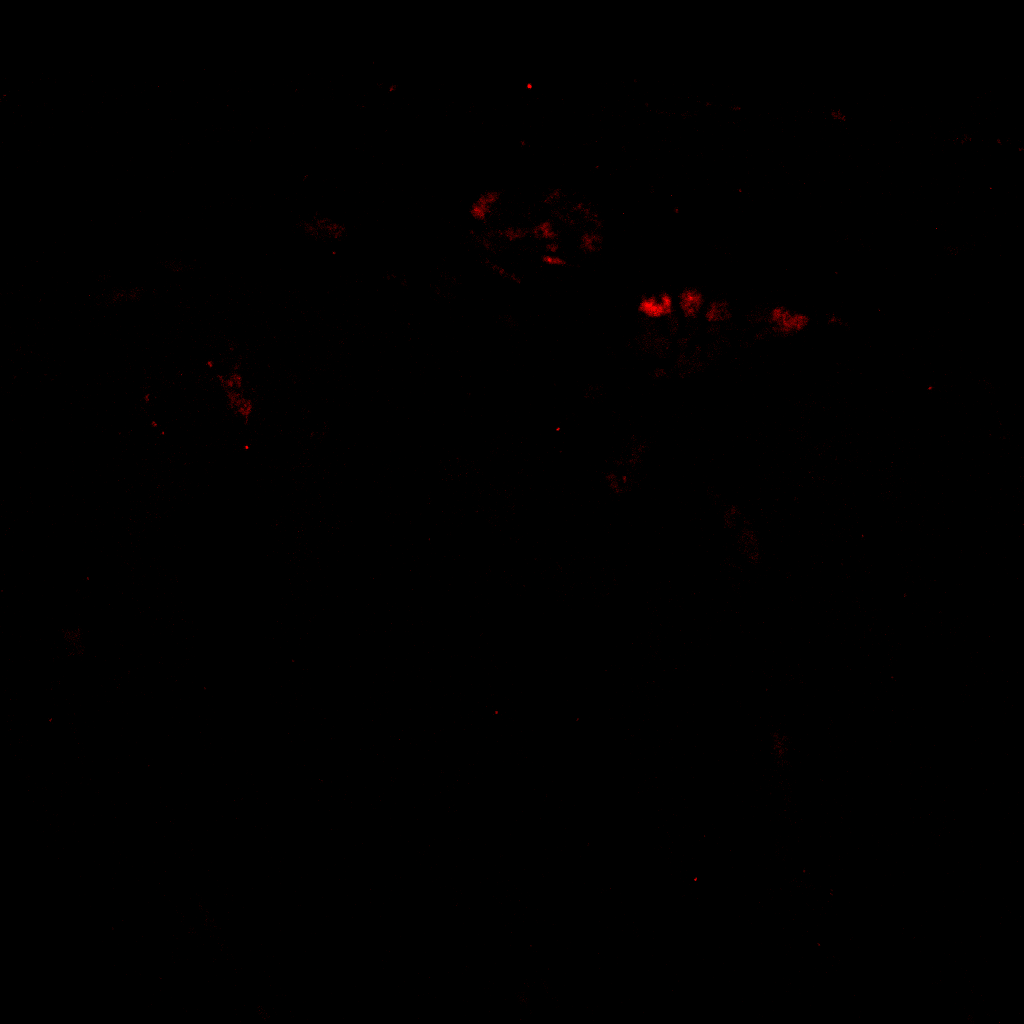

Supplement: Supplementary file 5 — Source Data for Expanded View and Appendix [file EMBR-24-e56030-s011.zip › Figure EV1-5, Appendix Figure S1-4/2. Figure EV2/Figure EV2A-IHC FOXC1 EMCN PROX1 in NEC model/DF-2, FOXC1.tif]

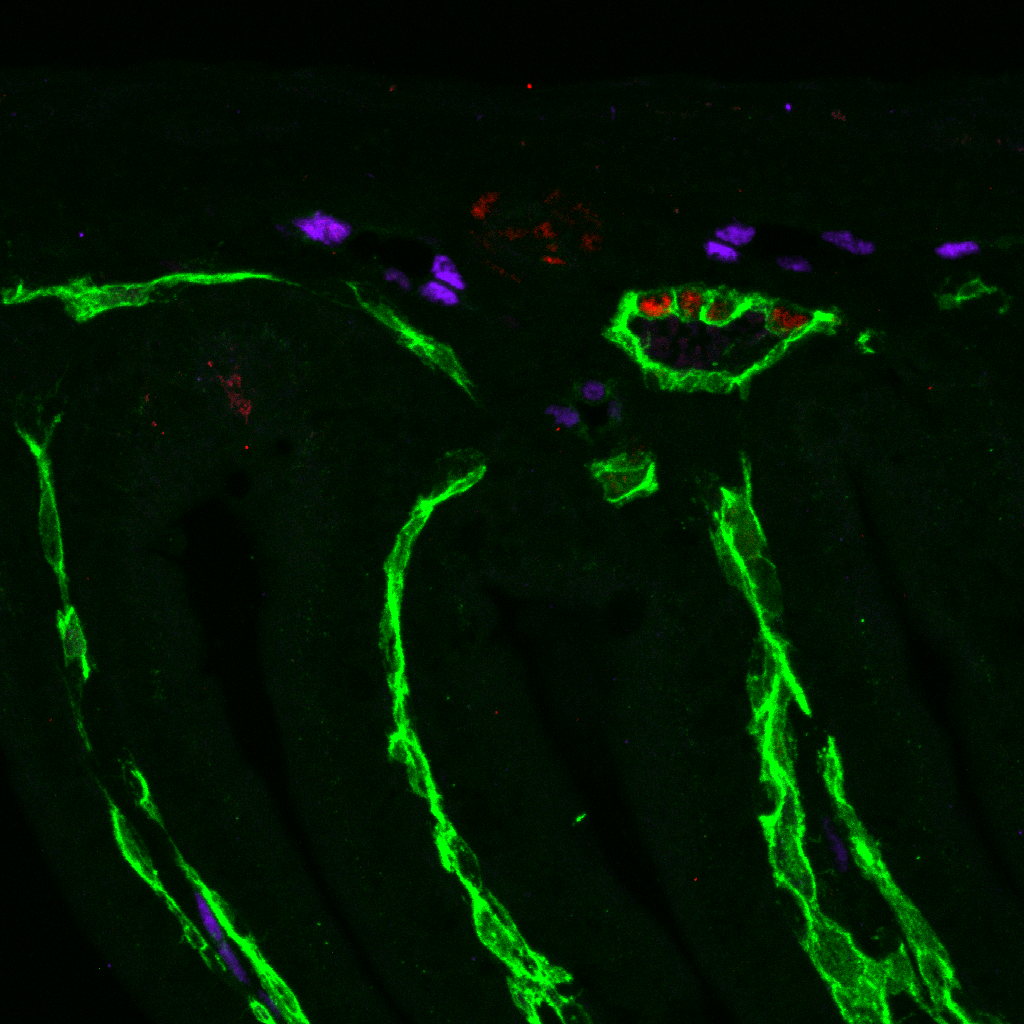

Supplement: Supplementary file 5 — Source Data for Expanded View and Appendix [file EMBR-24-e56030-s011.zip › Figure EV1-5, Appendix Figure S1-4/2. Figure EV2/Figure EV2A-IHC FOXC1 EMCN PROX1 in NEC model/DF-3, 3 colors.tif]

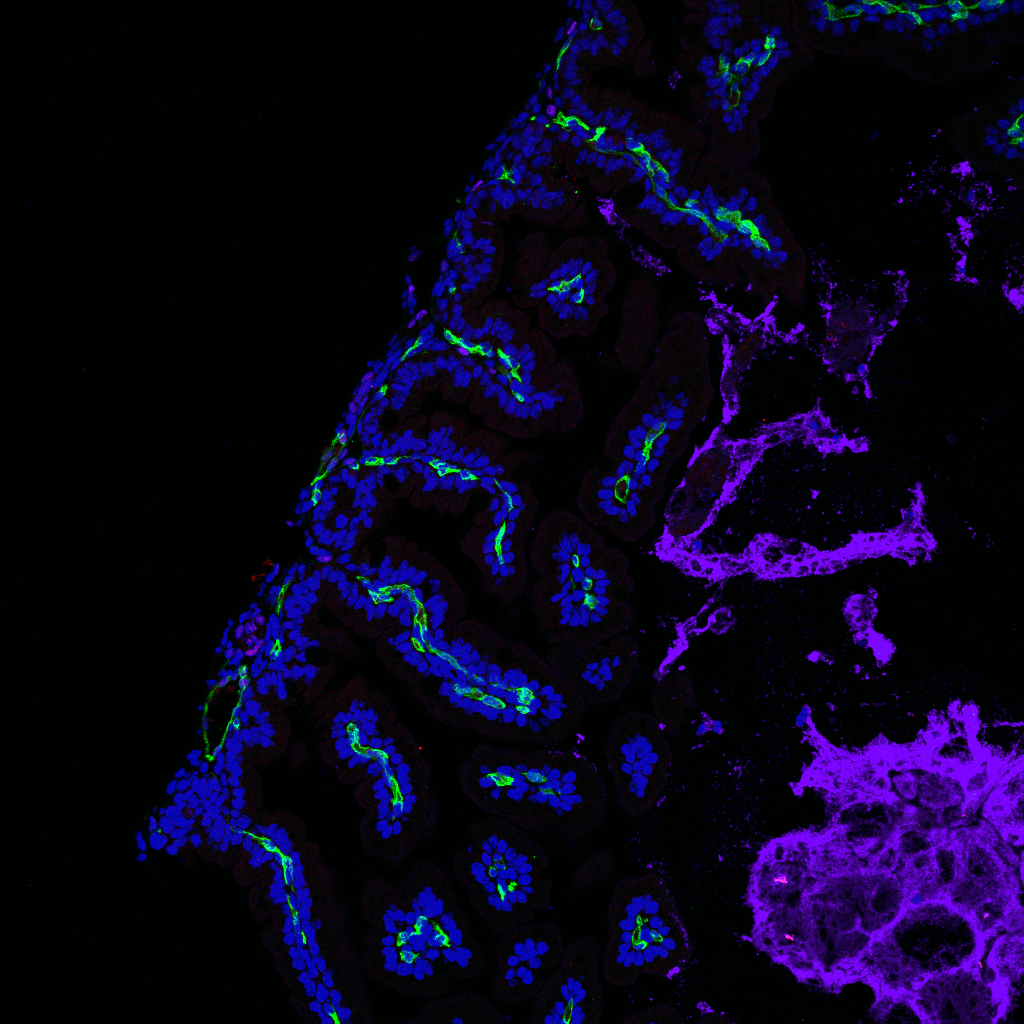

Supplement: Supplementary file 5 — Source Data for Expanded View and Appendix [file EMBR-24-e56030-s011.zip › Figure EV1-5, Appendix Figure S1-4/2. Figure EV2/Figure EV2A-IHC FOXC1 EMCN PROX1 in NEC model/NEC-1, 4 colors.tif]

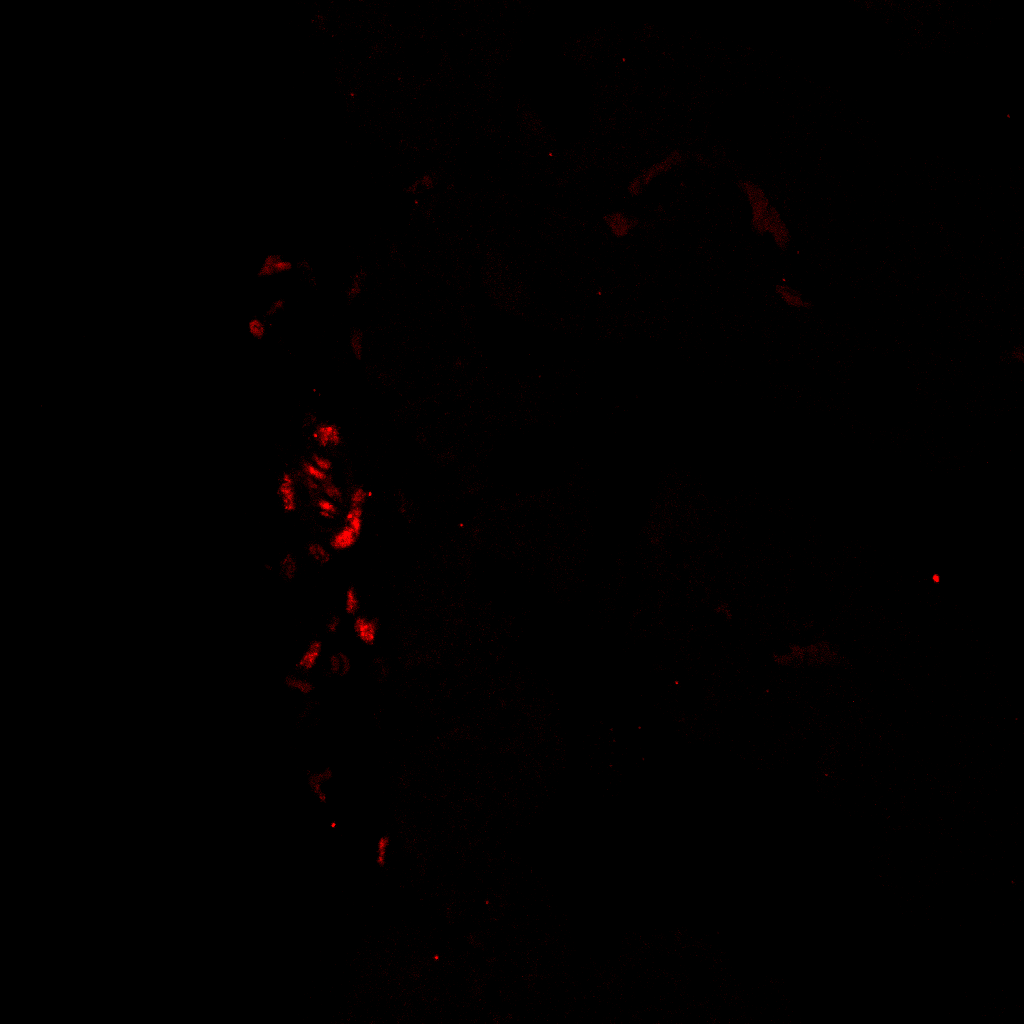

Supplement: Supplementary file 5 — Source Data for Expanded View and Appendix [file EMBR-24-e56030-s011.zip › Figure EV1-5, Appendix Figure S1-4/2. Figure EV2/Figure EV2A-IHC FOXC1 EMCN PROX1 in NEC model/NEC-2. FOXC1.tif]

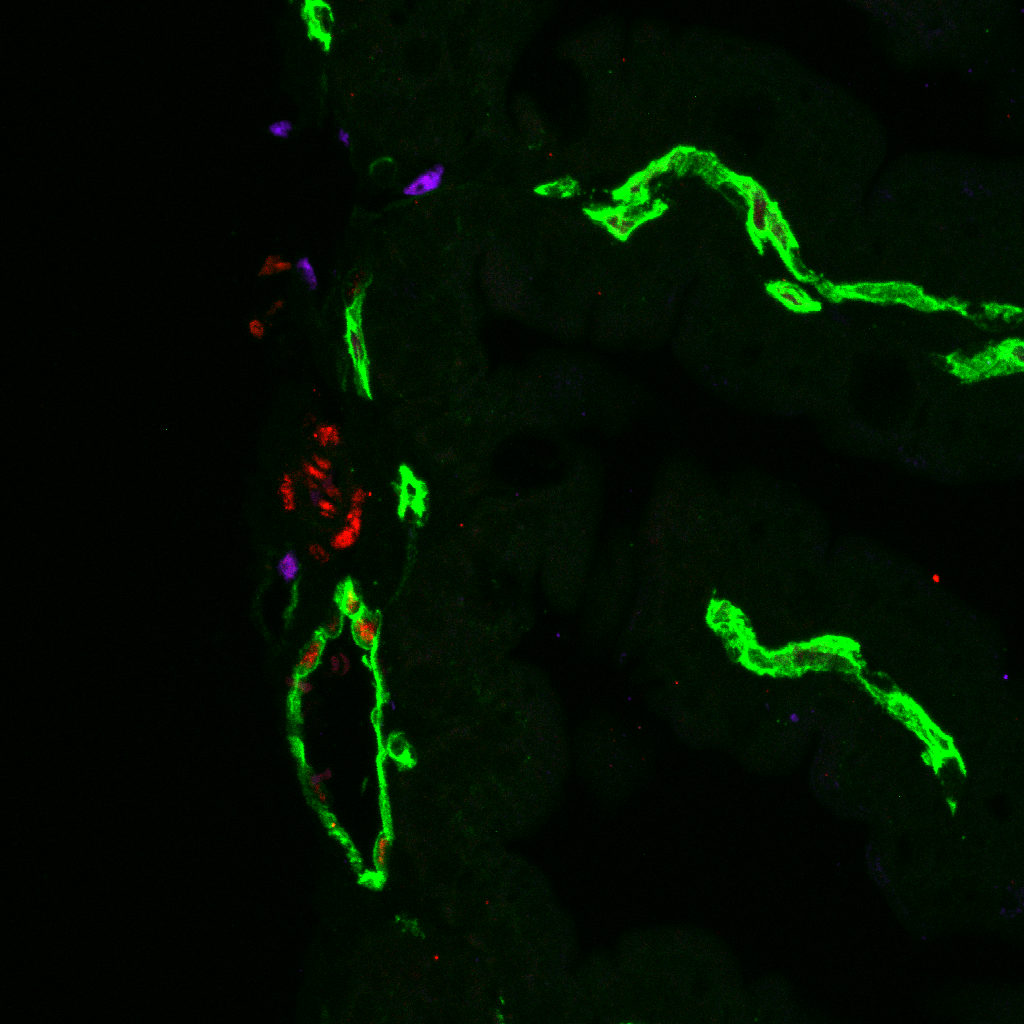

Supplement: Supplementary file 5 — Source Data for Expanded View and Appendix [file EMBR-24-e56030-s011.zip › Figure EV1-5, Appendix Figure S1-4/2. Figure EV2/Figure EV2A-IHC FOXC1 EMCN PROX1 in NEC model/NEC-3, 3 colors.tif]

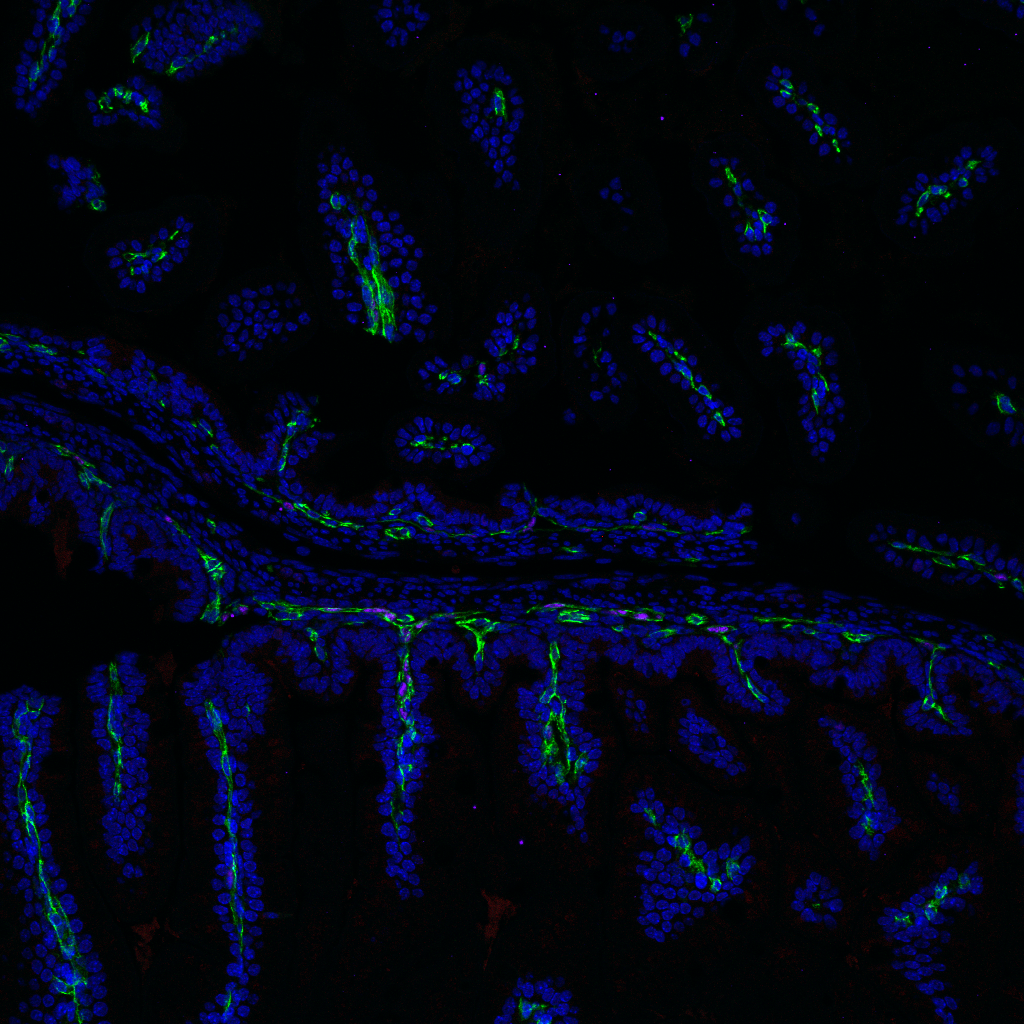

Supplement: Supplementary file 5 — Source Data for Expanded View and Appendix [file EMBR-24-e56030-s011.zip › Figure EV1-5, Appendix Figure S1-4/2. Figure EV2/Figure EV2C-IHC FOXC2 CD31 PROX1 in NEC model/DF-1. 4 colors.tif]

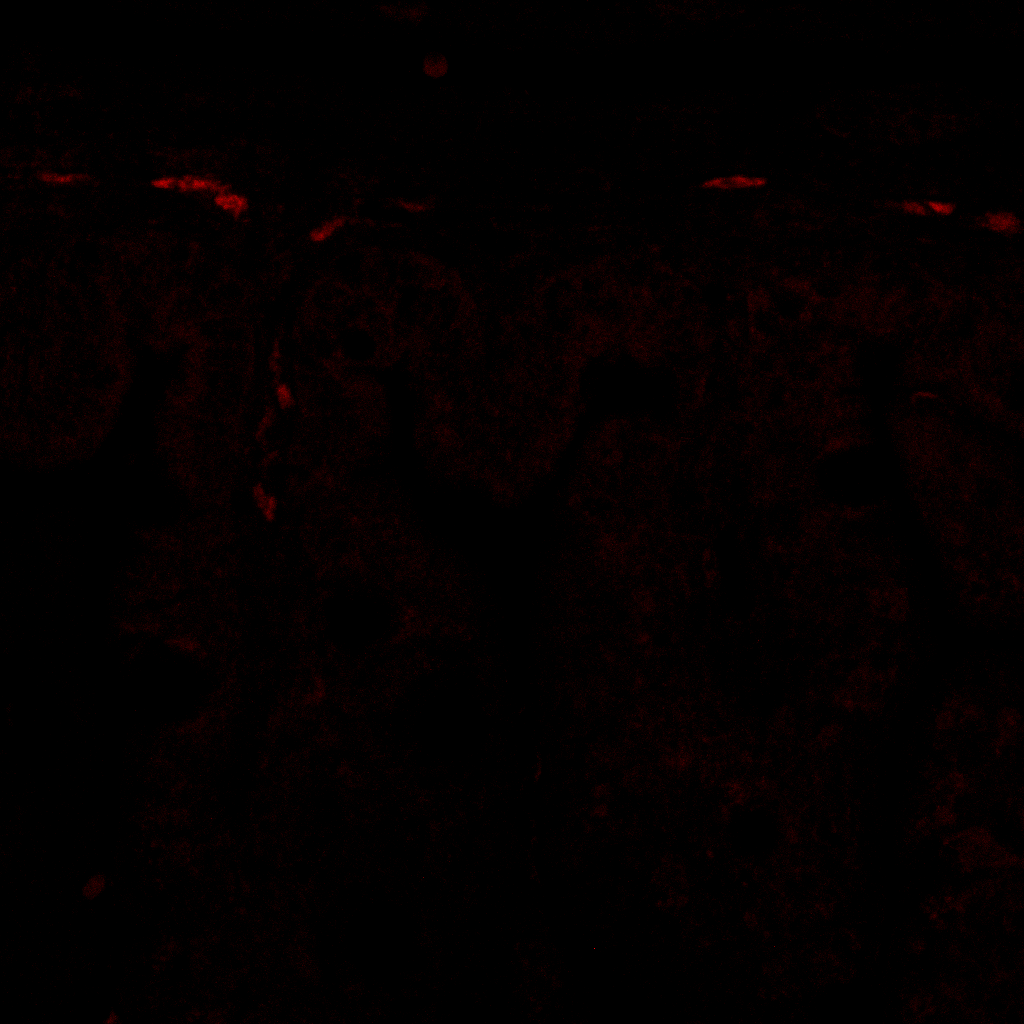

Supplement: Supplementary file 5 — Source Data for Expanded View and Appendix [file EMBR-24-e56030-s011.zip › Figure EV1-5, Appendix Figure S1-4/2. Figure EV2/Figure EV2C-IHC FOXC2 CD31 PROX1 in NEC model/DF-2. FOXC2.tif]

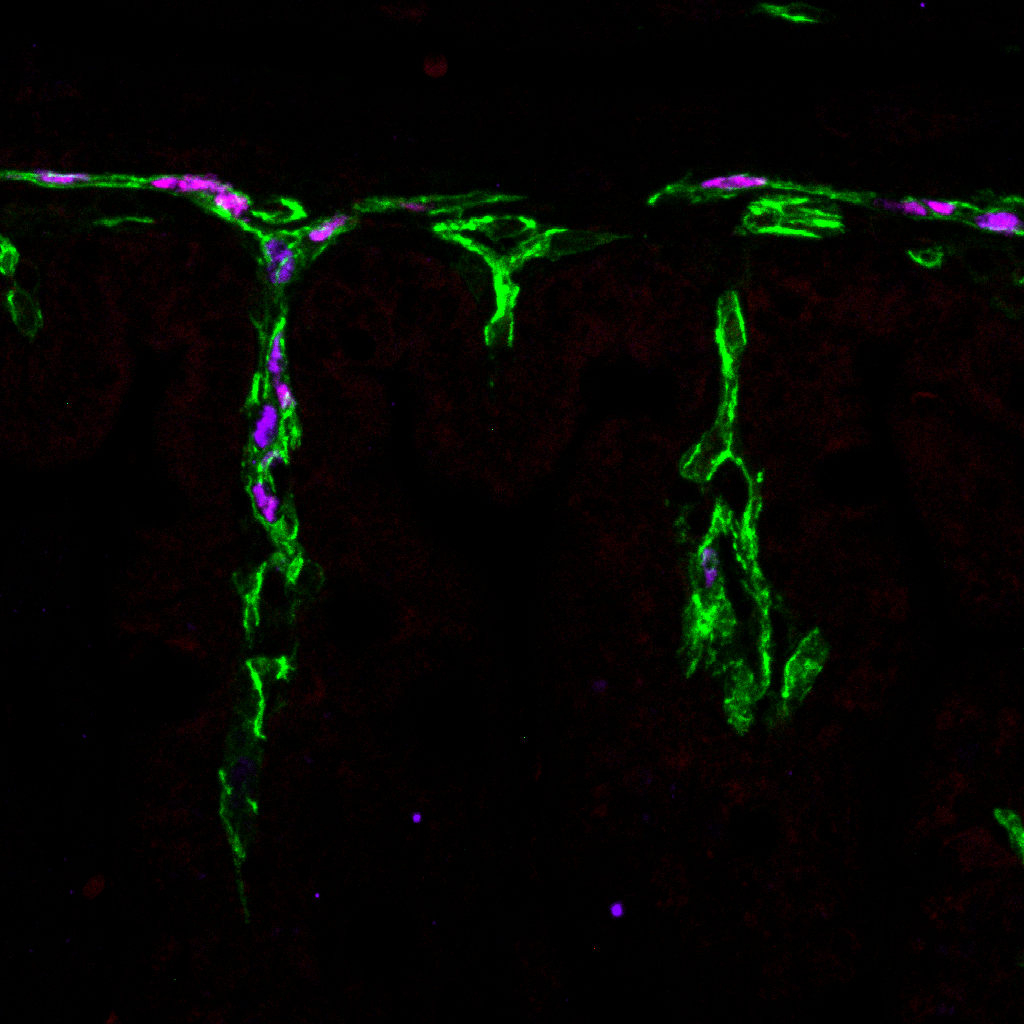

Supplement: Supplementary file 5 — Source Data for Expanded View and Appendix [file EMBR-24-e56030-s011.zip › Figure EV1-5, Appendix Figure S1-4/2. Figure EV2/Figure EV2C-IHC FOXC2 CD31 PROX1 in NEC model/DF-3. 3 colors.tif]

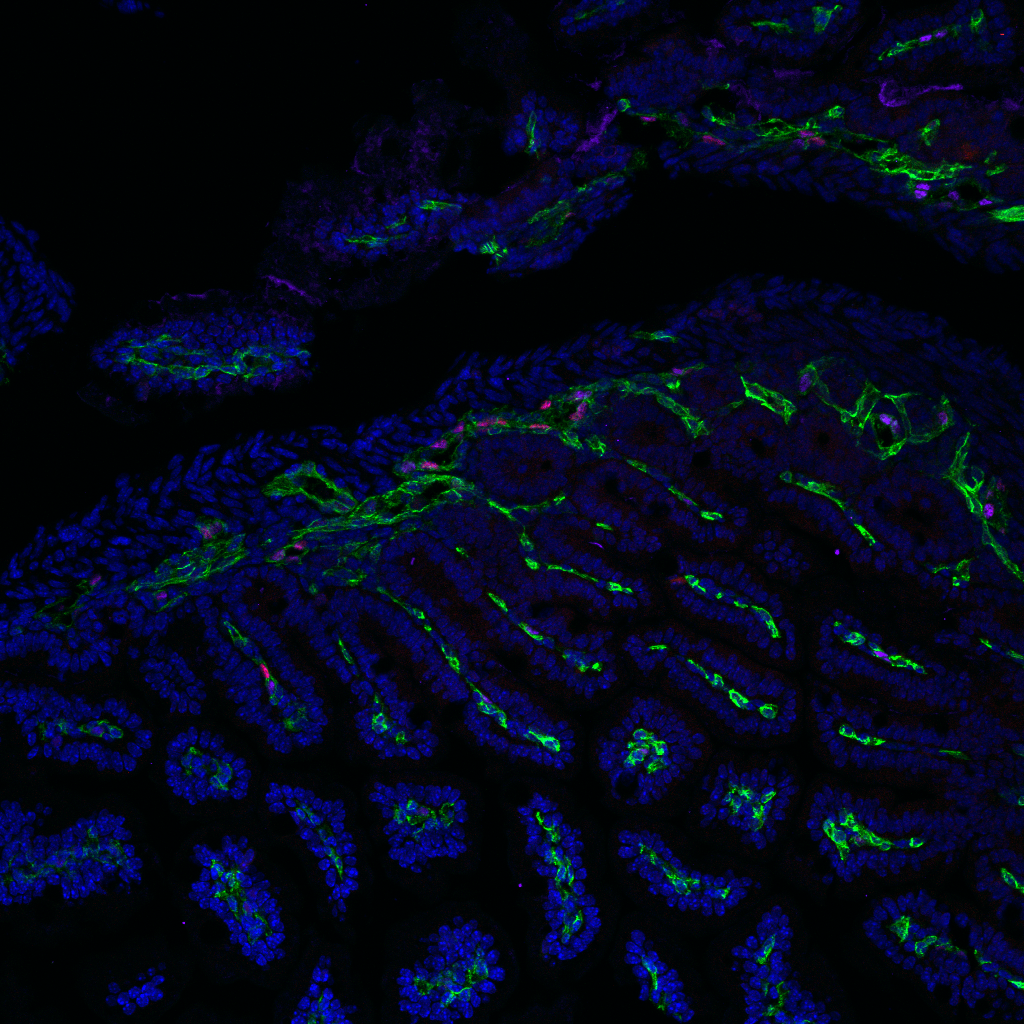

Supplement: Supplementary file 5 — Source Data for Expanded View and Appendix [file EMBR-24-e56030-s011.zip › Figure EV1-5, Appendix Figure S1-4/2. Figure EV2/Figure EV2C-IHC FOXC2 CD31 PROX1 in NEC model/NEC-1. 4 colors.tif]

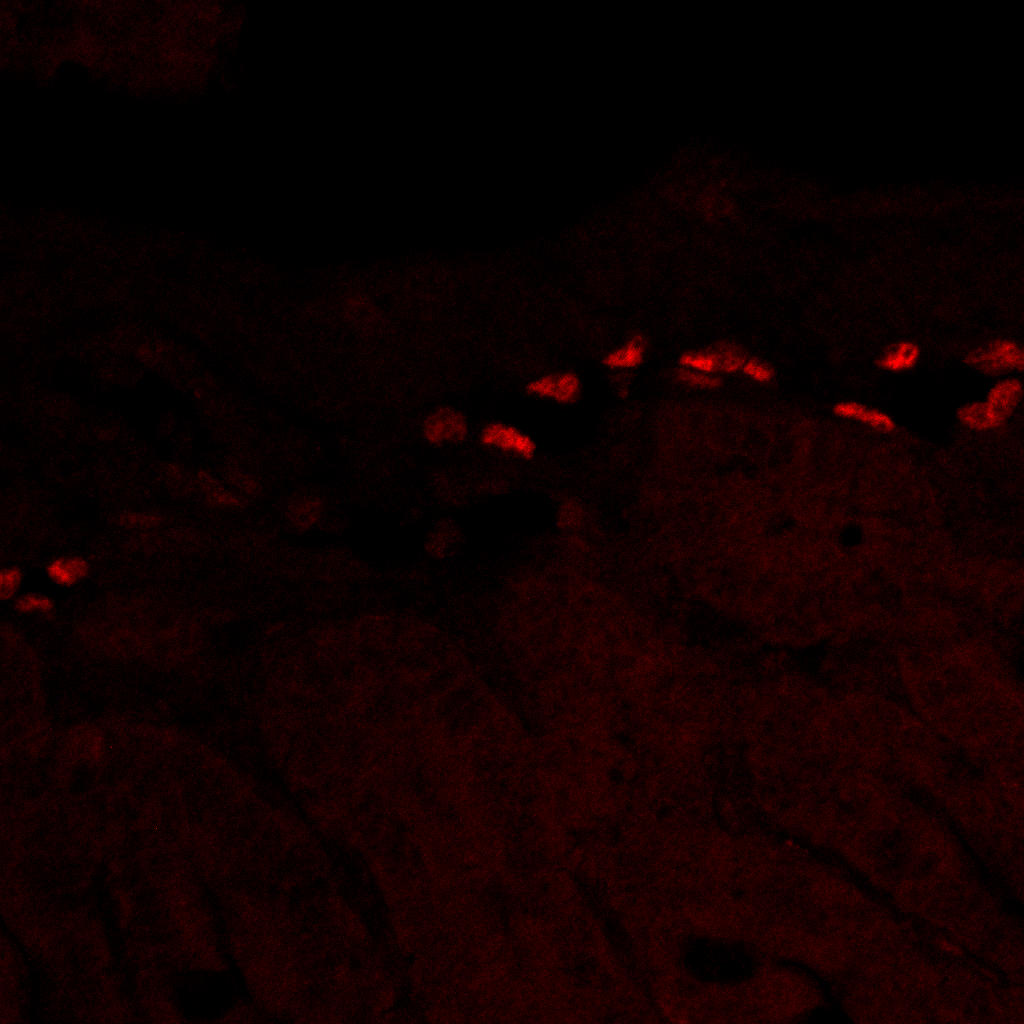

Supplement: Supplementary file 5 — Source Data for Expanded View and Appendix [file EMBR-24-e56030-s011.zip › Figure EV1-5, Appendix Figure S1-4/2. Figure EV2/Figure EV2C-IHC FOXC2 CD31 PROX1 in NEC model/NEC-2. FOXC2.tif]

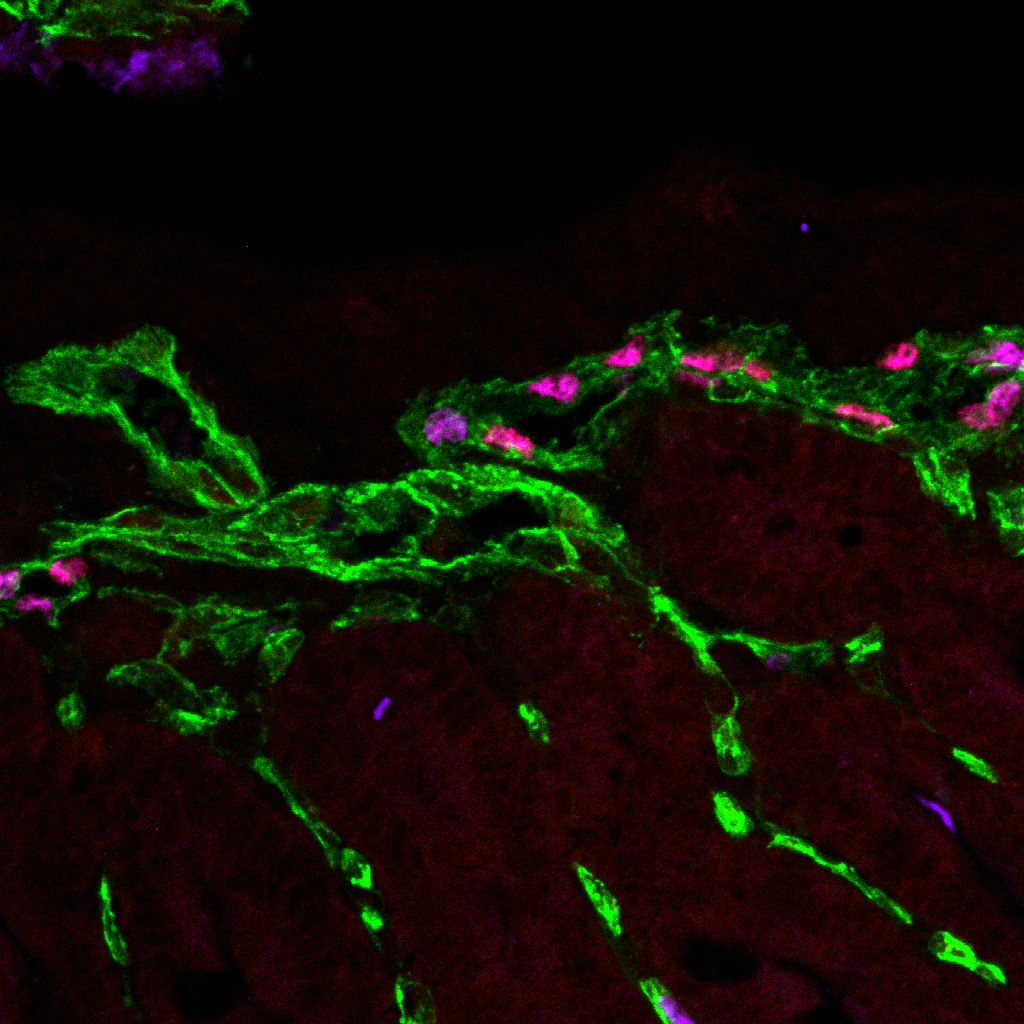

Supplement: Supplementary file 5 — Source Data for Expanded View and Appendix [file EMBR-24-e56030-s011.zip › Figure EV1-5, Appendix Figure S1-4/2. Figure EV2/Figure EV2C-IHC FOXC2 CD31 PROX1 in NEC model/NEC-3. 3 colors.tif]

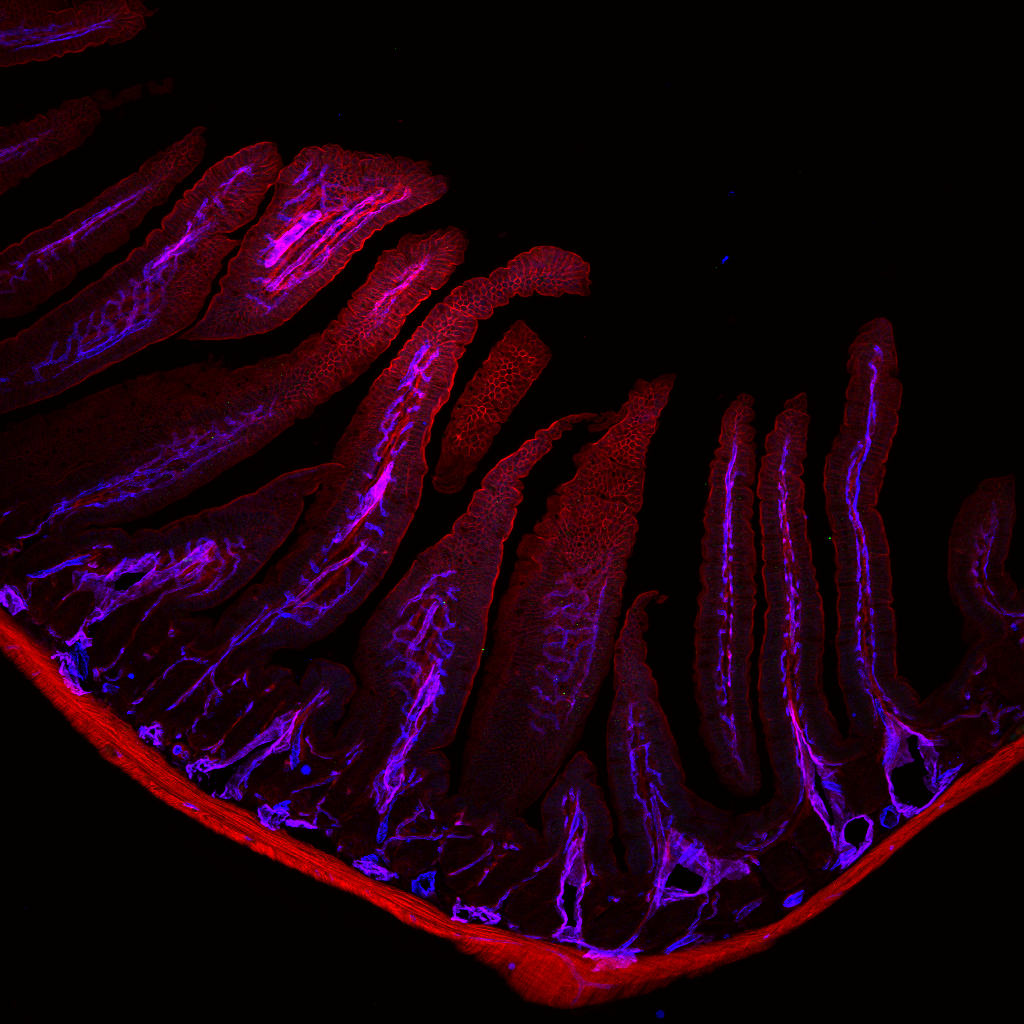

Supplement: Supplementary file 5 — Source Data for Expanded View and Appendix [file EMBR-24-e56030-s011.zip › Figure EV1-5, Appendix Figure S1-4/3. Figure EV3/Figure EV3A-IHC mTmG, CD31, LYVE1/1. Control, 4 colors.tif]

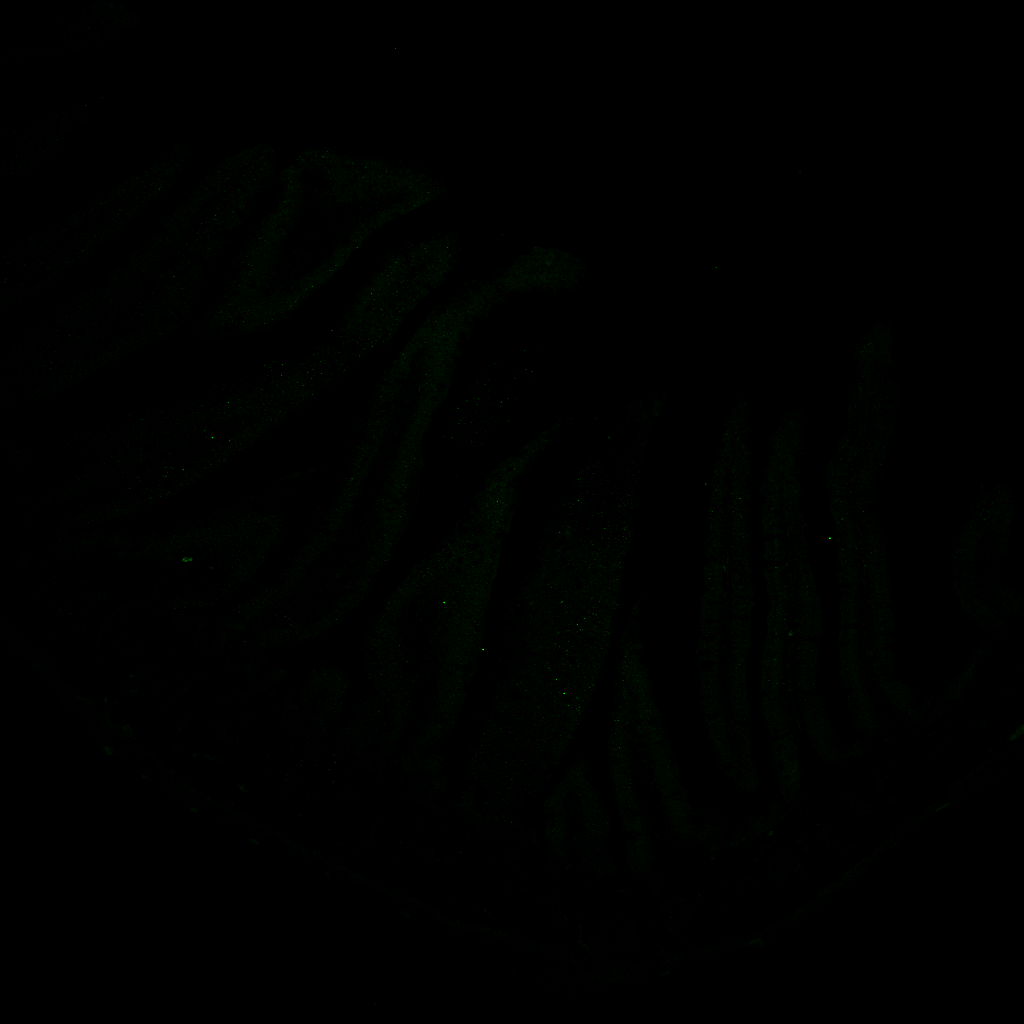

Supplement: Supplementary file 5 — Source Data for Expanded View and Appendix [file EMBR-24-e56030-s011.zip › Figure EV1-5, Appendix Figure S1-4/3. Figure EV3/Figure EV3A-IHC mTmG, CD31, LYVE1/2. Control, GFP.tif]

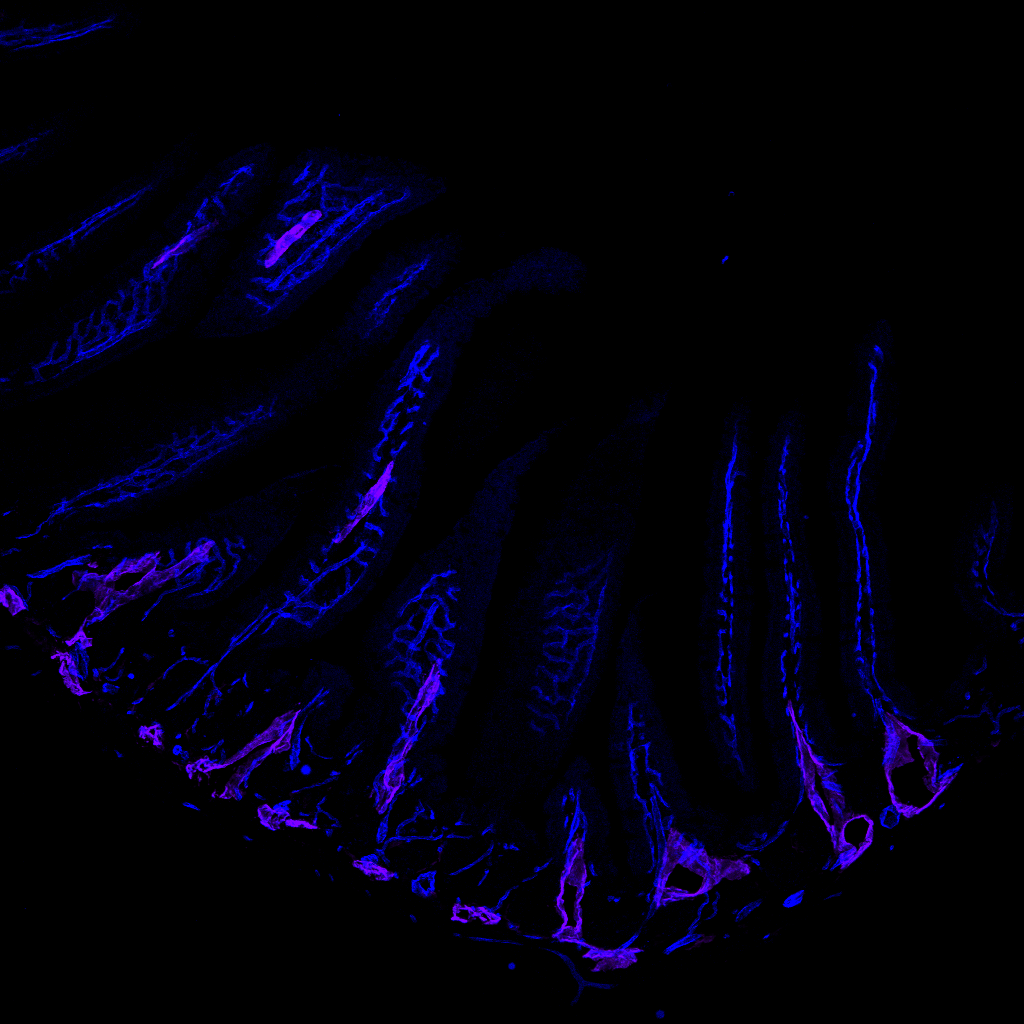

Supplement: Supplementary file 5 — Source Data for Expanded View and Appendix [file EMBR-24-e56030-s011.zip › Figure EV1-5, Appendix Figure S1-4/3. Figure EV3/Figure EV3A-IHC mTmG, CD31, LYVE1/3. Control, 2 colors.tif]

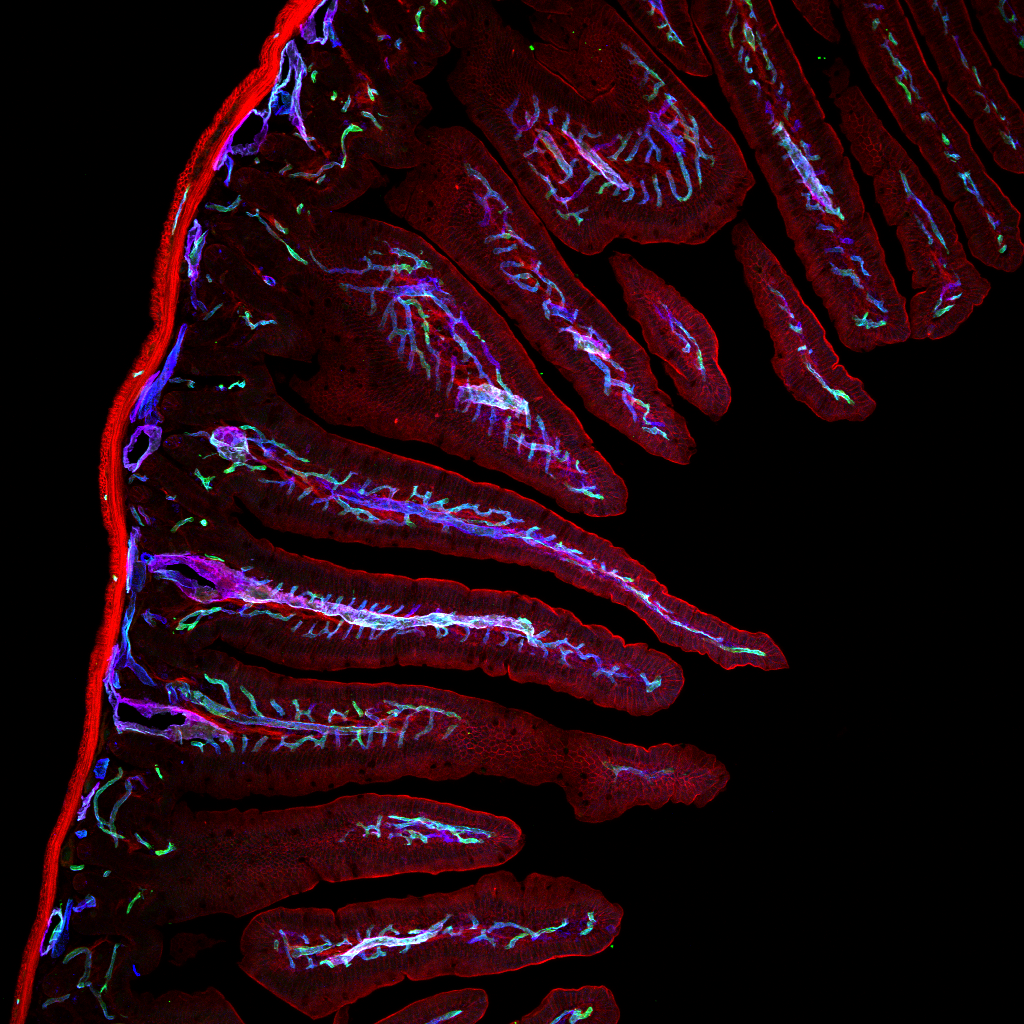

Supplement: Supplementary file 5 — Source Data for Expanded View and Appendix [file EMBR-24-e56030-s011.zip › Figure EV1-5, Appendix Figure S1-4/3. Figure EV3/Figure EV3A-IHC mTmG, CD31, LYVE1/4. mTmG;EC-Foxc-DKO, 4 colors.tif]

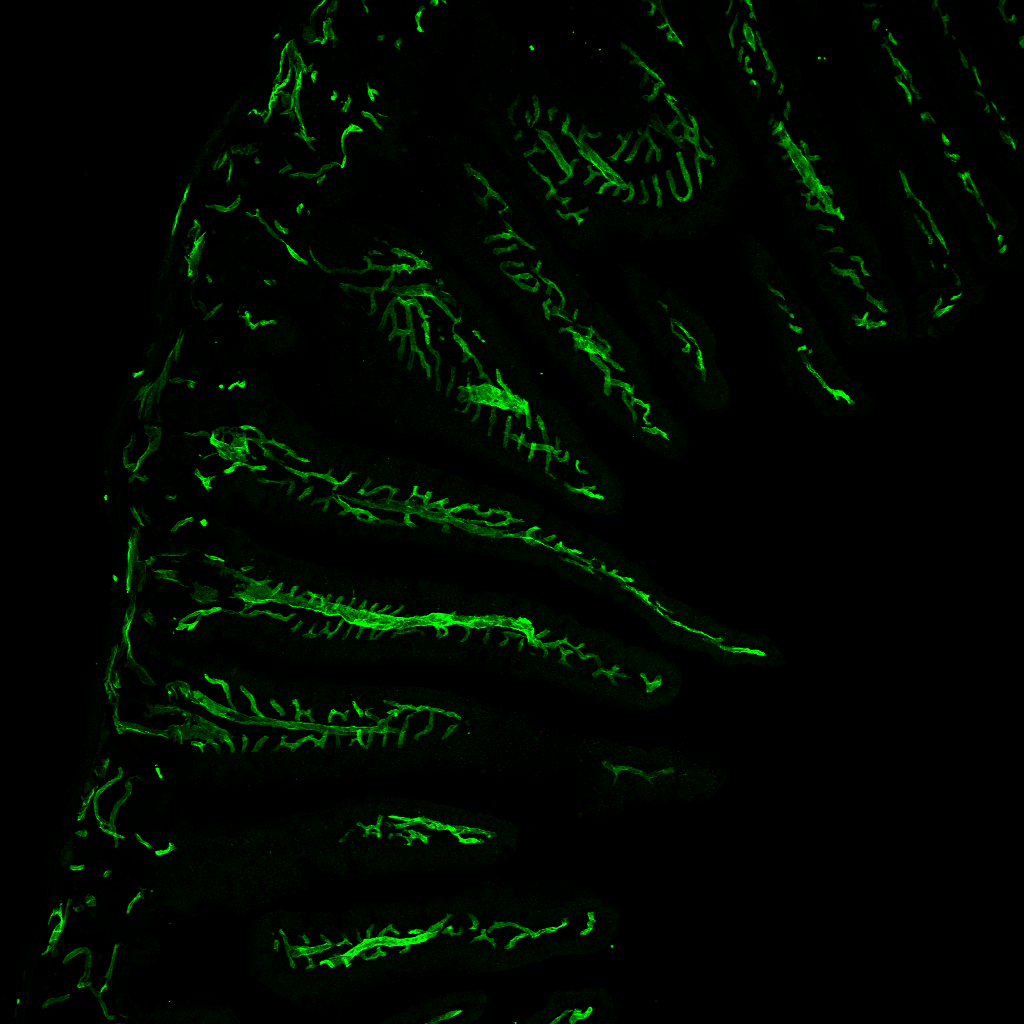

Supplement: Supplementary file 5 — Source Data for Expanded View and Appendix [file EMBR-24-e56030-s011.zip › Figure EV1-5, Appendix Figure S1-4/3. Figure EV3/Figure EV3A-IHC mTmG, CD31, LYVE1/5. mTmG;EC-Foxc-DKO, GFP.tif]

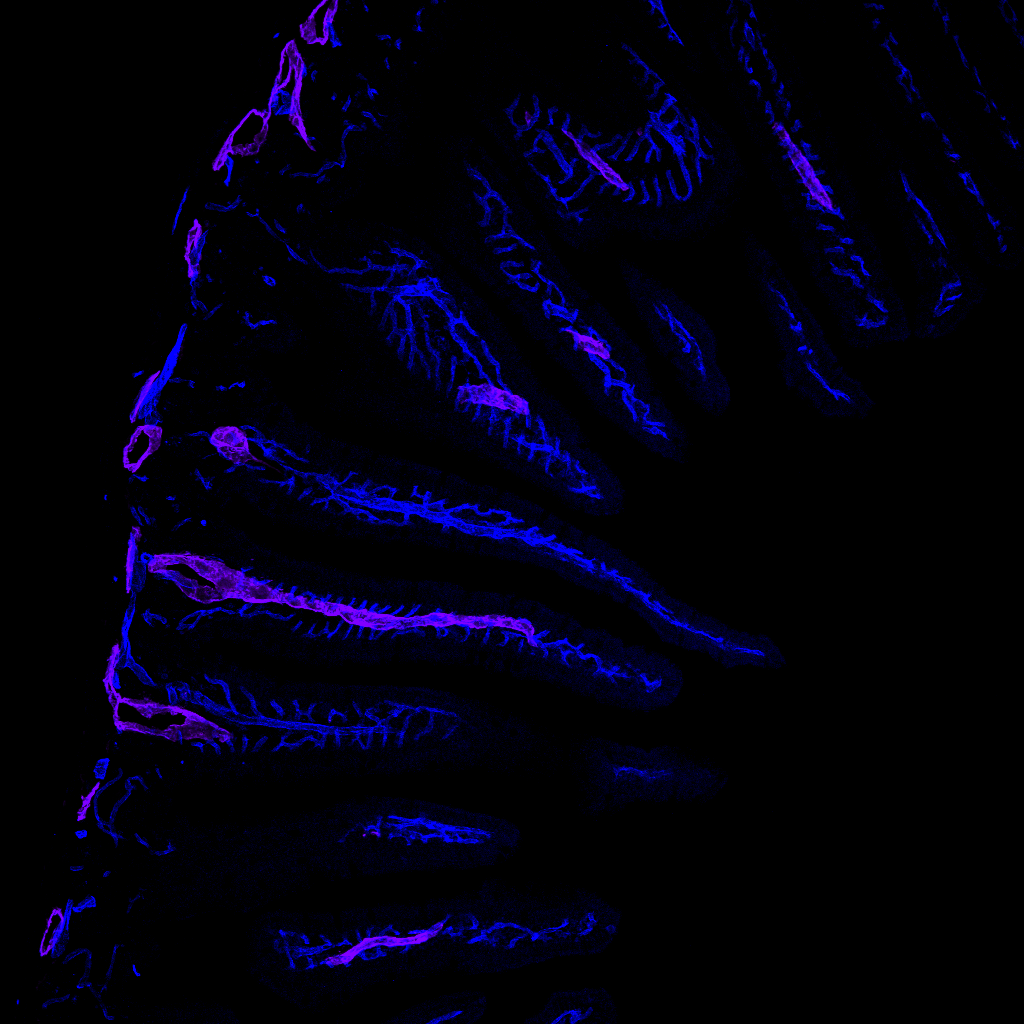

Supplement: Supplementary file 5 — Source Data for Expanded View and Appendix [file EMBR-24-e56030-s011.zip › Figure EV1-5, Appendix Figure S1-4/3. Figure EV3/Figure EV3A-IHC mTmG, CD31, LYVE1/6. mTmG;EC-Foxc-DKO, 2 colors.tif]

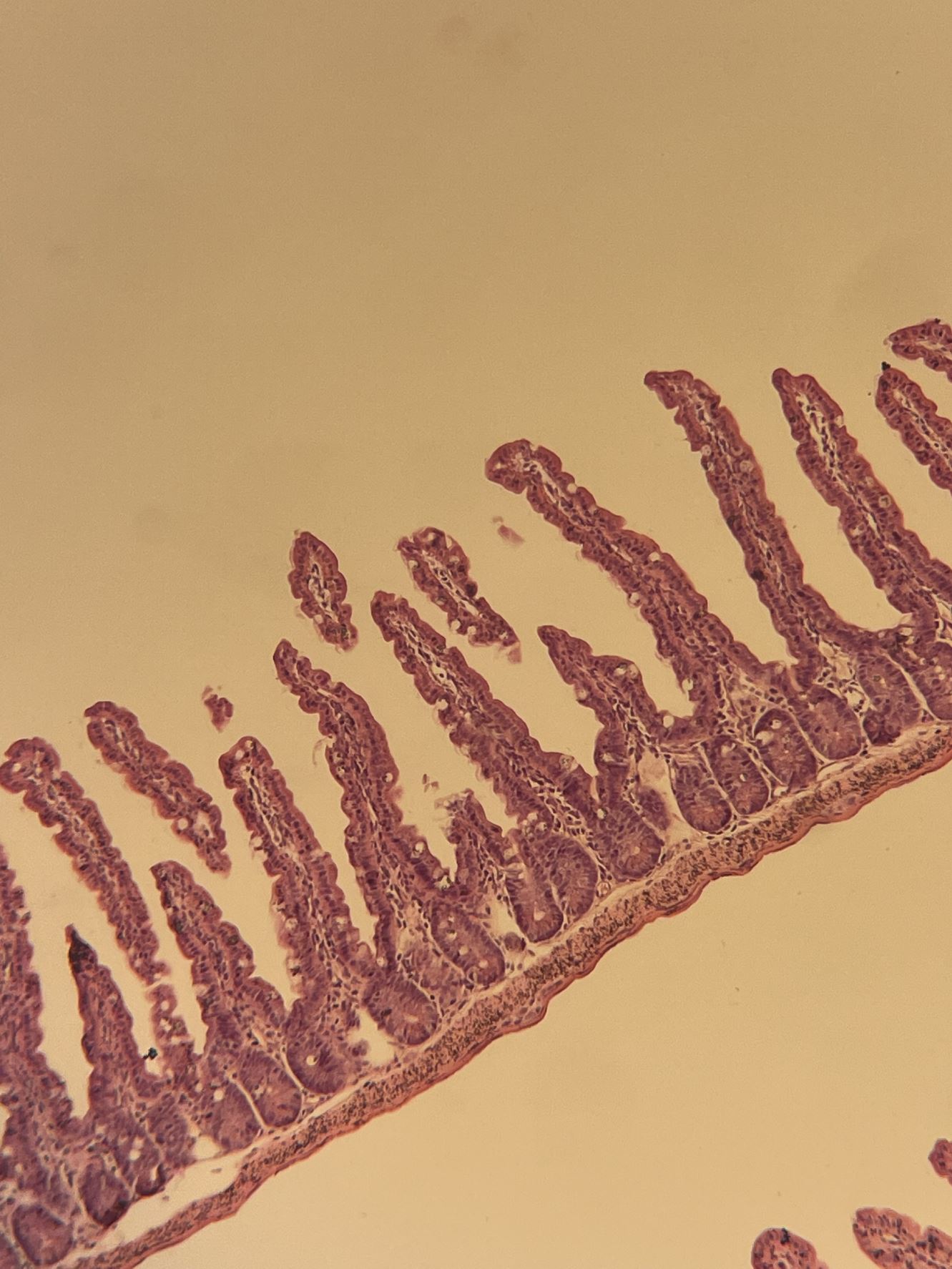

Supplement: Supplementary file 5 — Source Data for Expanded View and Appendix [file EMBR-24-e56030-s011.zip › Figure EV1-5, Appendix Figure S1-4/3. Figure EV3/Figure EV3B,C,E-G HE staining in different mouse strains/B. Control.jpg]

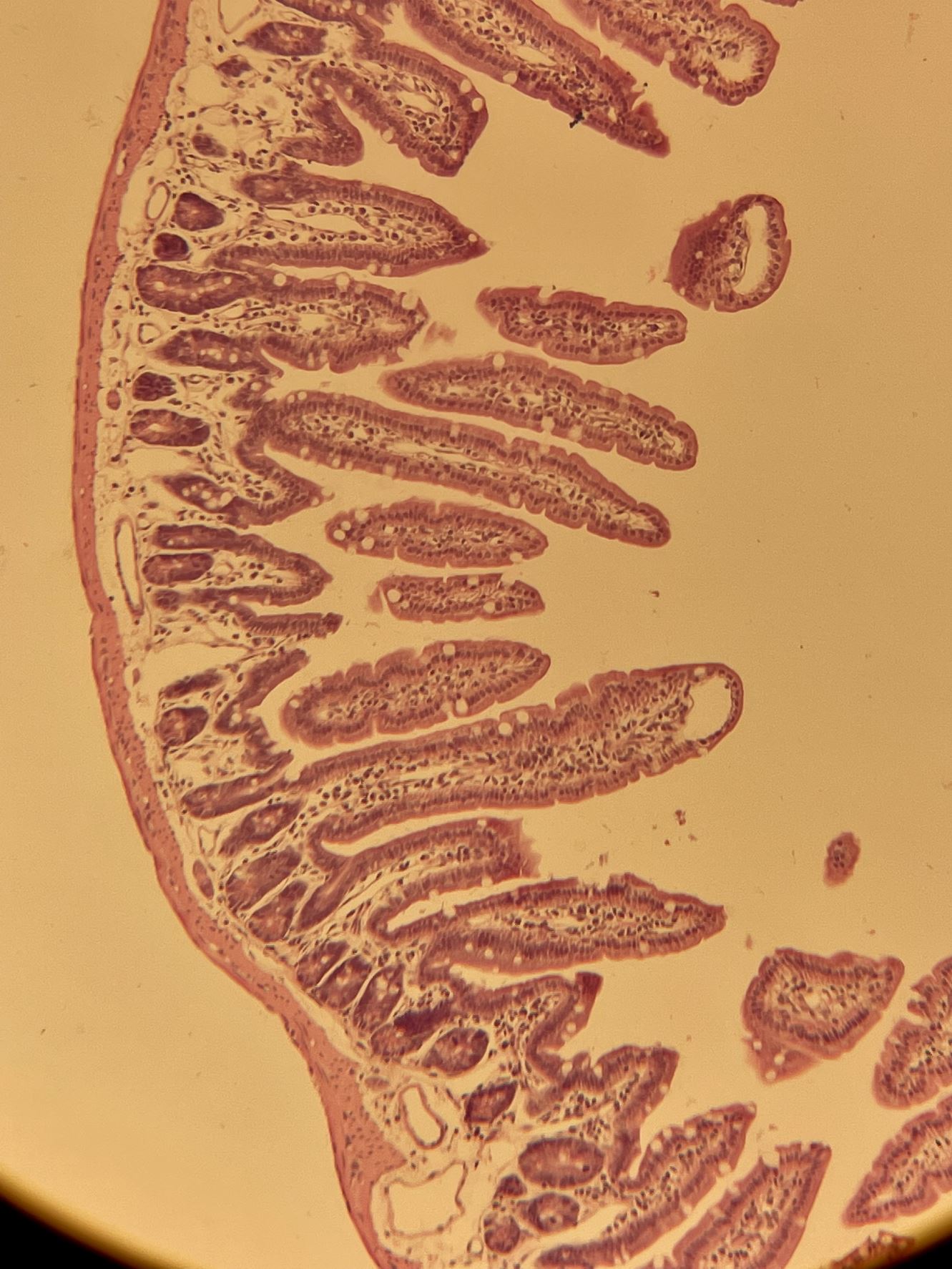

Supplement: Supplementary file 5 — Source Data for Expanded View and Appendix [file EMBR-24-e56030-s011.zip › Figure EV1-5, Appendix Figure S1-4/3. Figure EV3/Figure EV3B,C,E-G HE staining in different mouse strains/B. EC-Foxc1-KO.jpg]

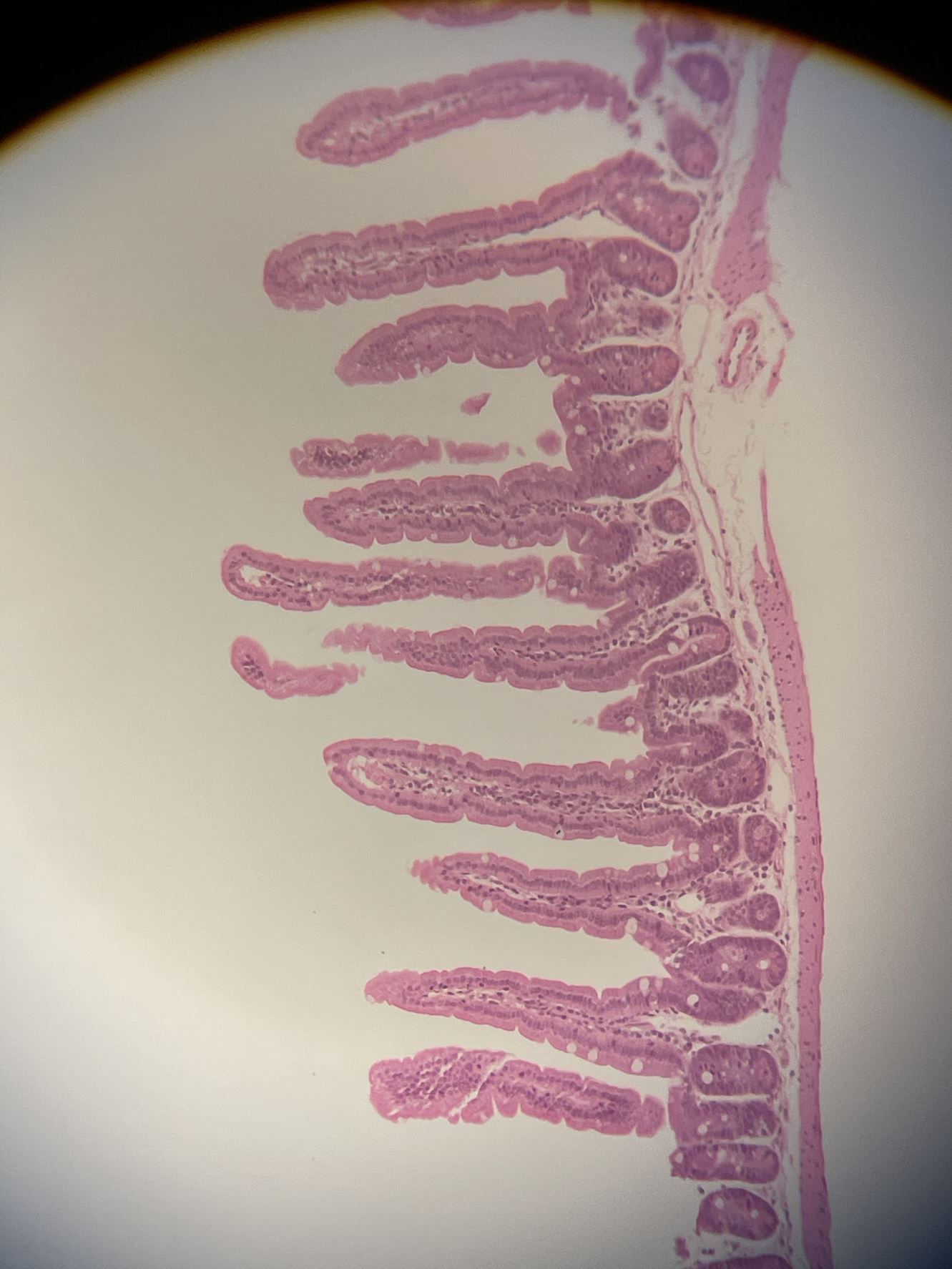

Supplement: Supplementary file 5 — Source Data for Expanded View and Appendix [file EMBR-24-e56030-s011.zip › Figure EV1-5, Appendix Figure S1-4/3. Figure EV3/Figure EV3B,C,E-G HE staining in different mouse strains/C. Control.jpg]

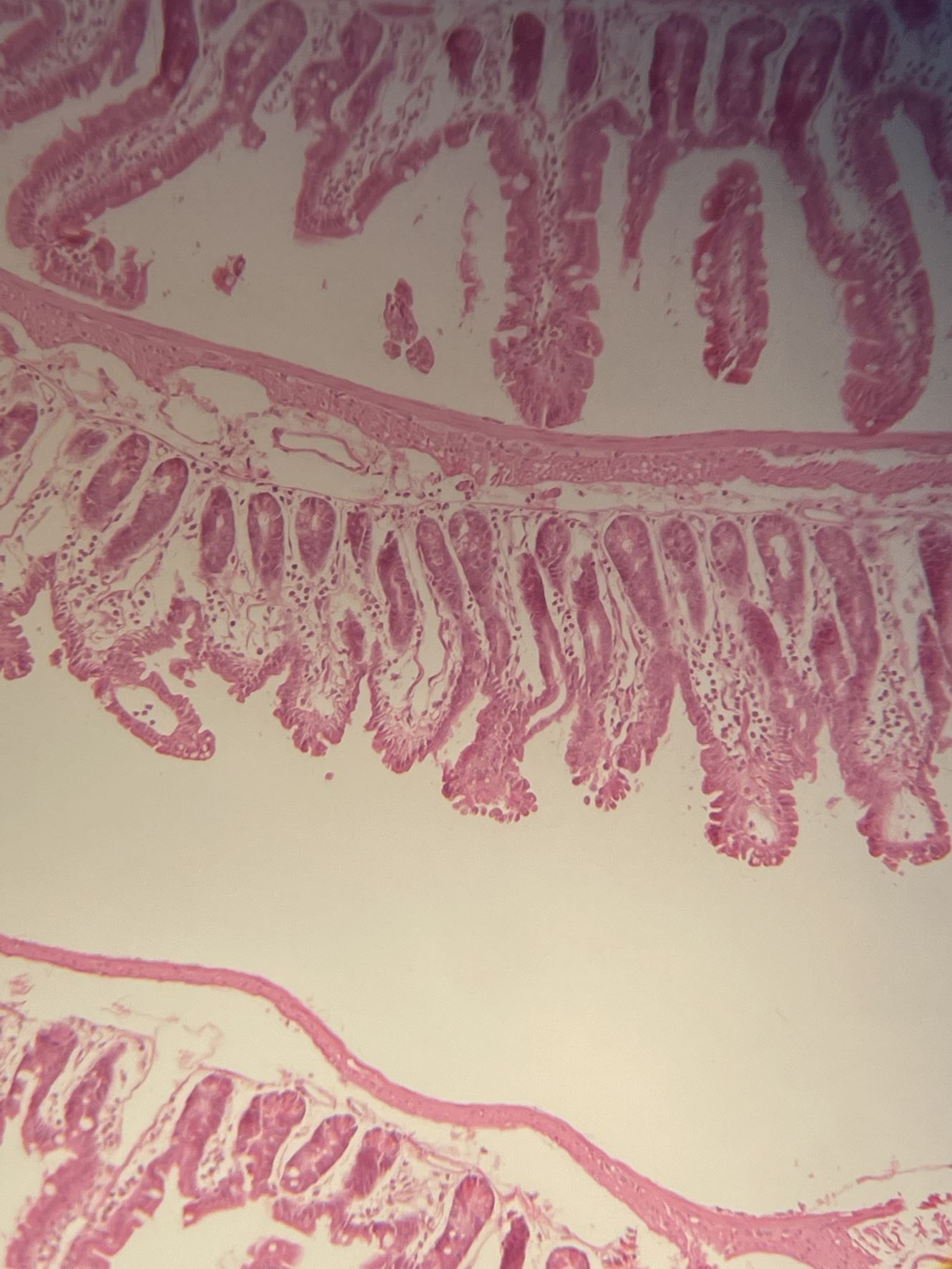

Supplement: Supplementary file 5 — Source Data for Expanded View and Appendix [file EMBR-24-e56030-s011.zip › Figure EV1-5, Appendix Figure S1-4/3. Figure EV3/Figure EV3B,C,E-G HE staining in different mouse strains/C. EC-Foxc2-KO.jpg]

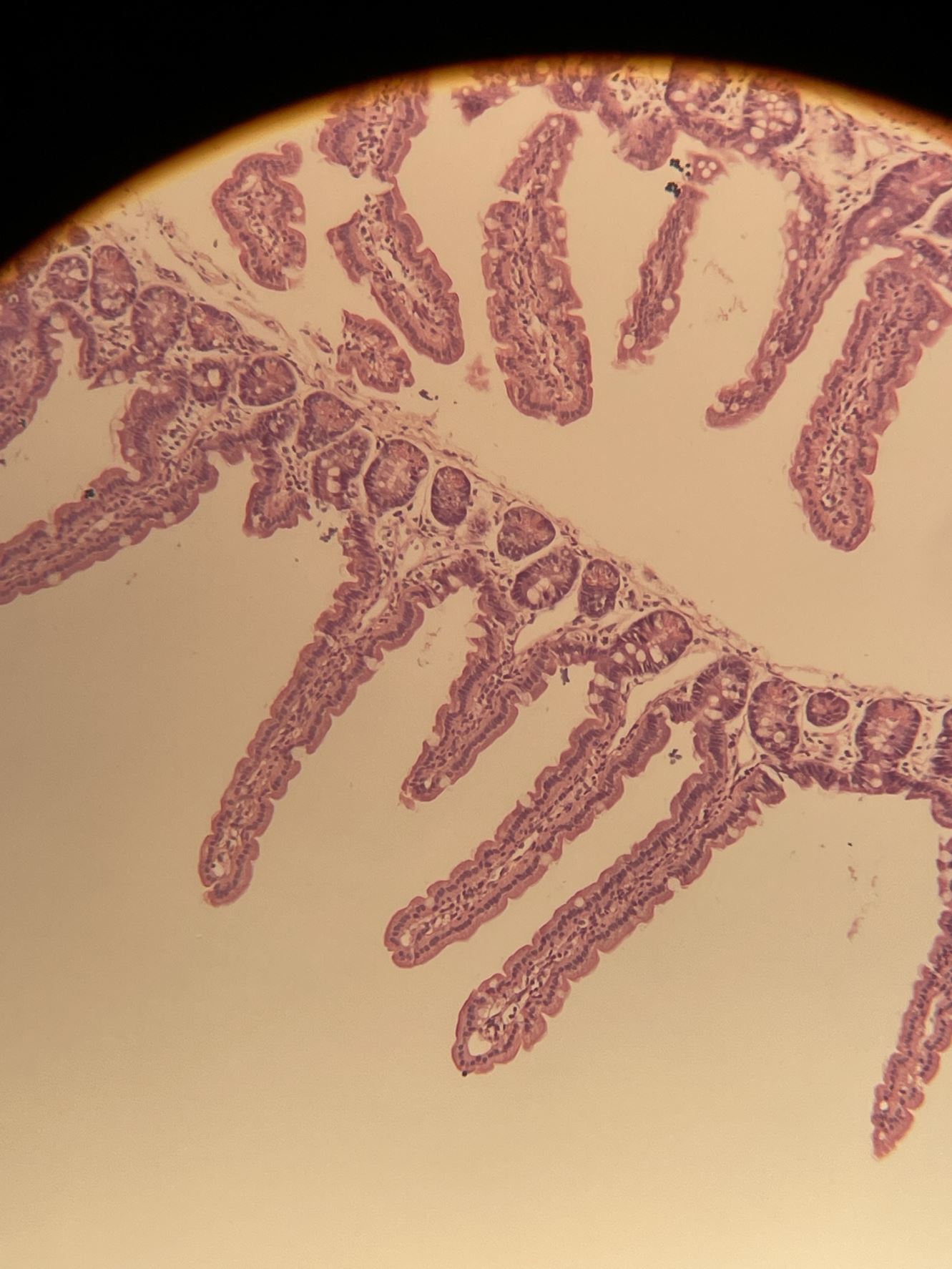

Supplement: Supplementary file 5 — Source Data for Expanded View and Appendix [file EMBR-24-e56030-s011.zip › Figure EV1-5, Appendix Figure S1-4/3. Figure EV3/Figure EV3B,C,E-G HE staining in different mouse strains/E. Control.jpg]

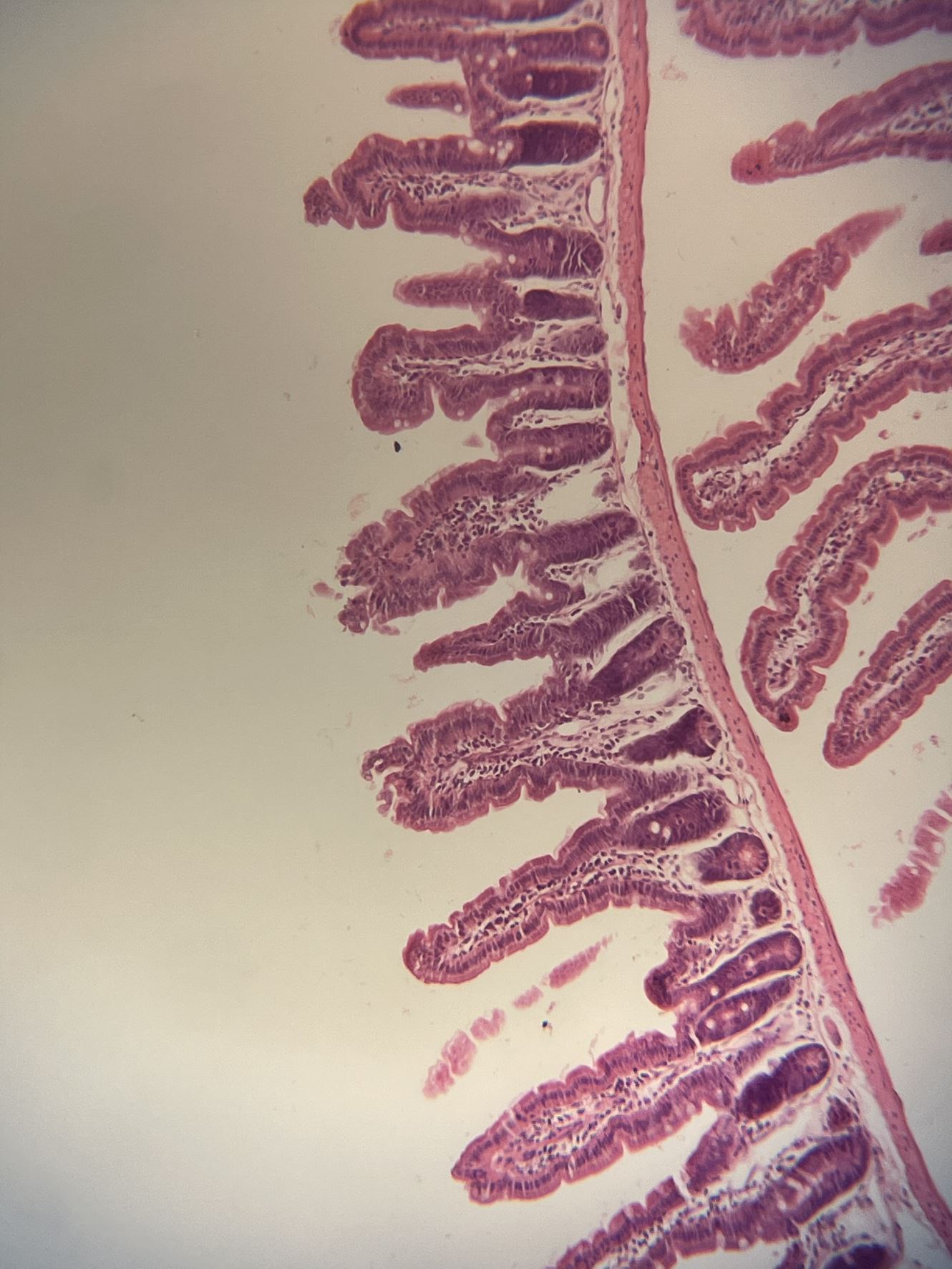

Supplement: Supplementary file 5 — Source Data for Expanded View and Appendix [file EMBR-24-e56030-s011.zip › Figure EV1-5, Appendix Figure S1-4/3. Figure EV3/Figure EV3B,C,E-G HE staining in different mouse strains/E. LEC-Foxc-DKO.jpg]

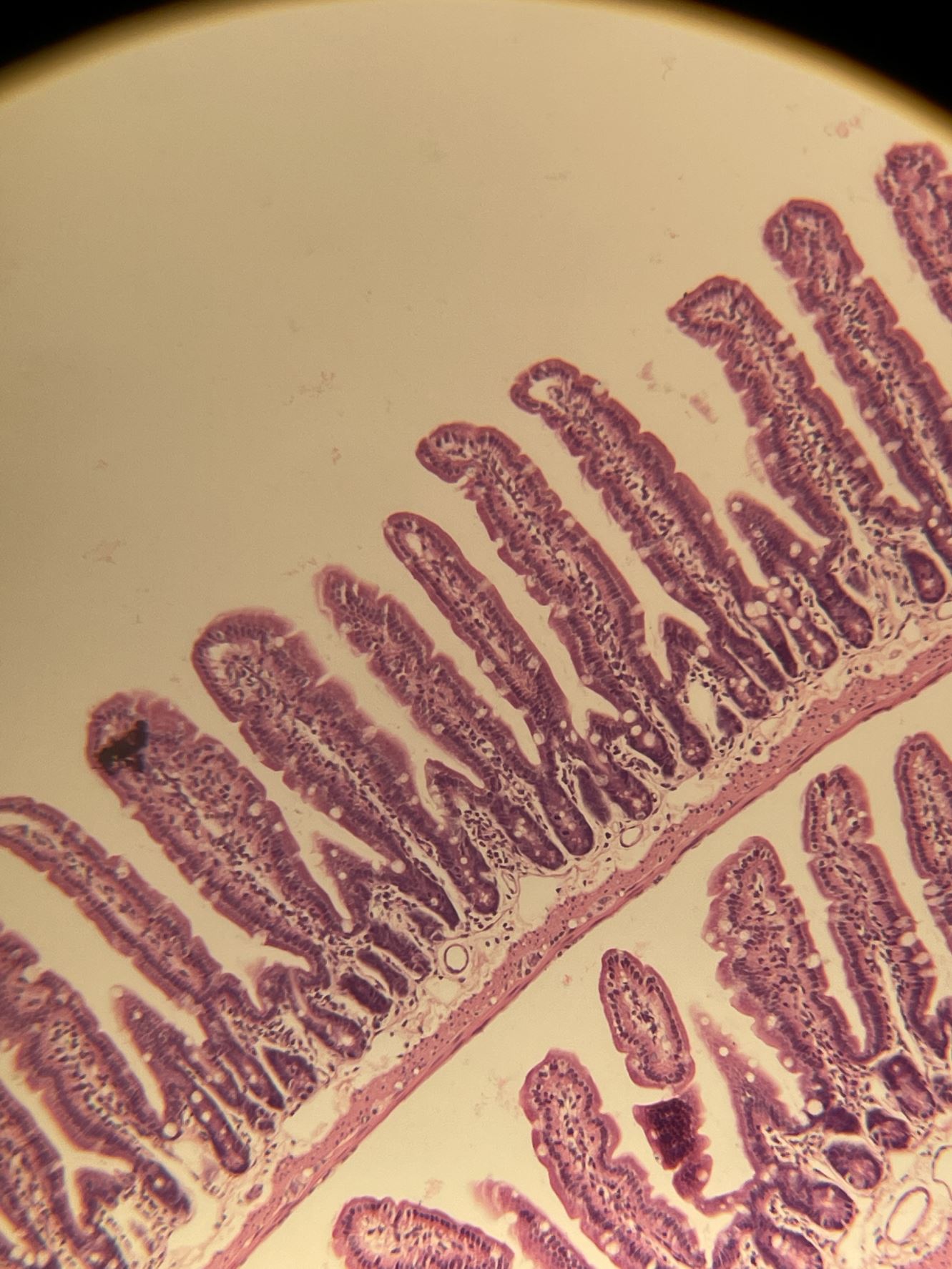

Supplement: Supplementary file 5 — Source Data for Expanded View and Appendix [file EMBR-24-e56030-s011.zip › Figure EV1-5, Appendix Figure S1-4/3. Figure EV3/Figure EV3B,C,E-G HE staining in different mouse strains/F. Control.jpg]

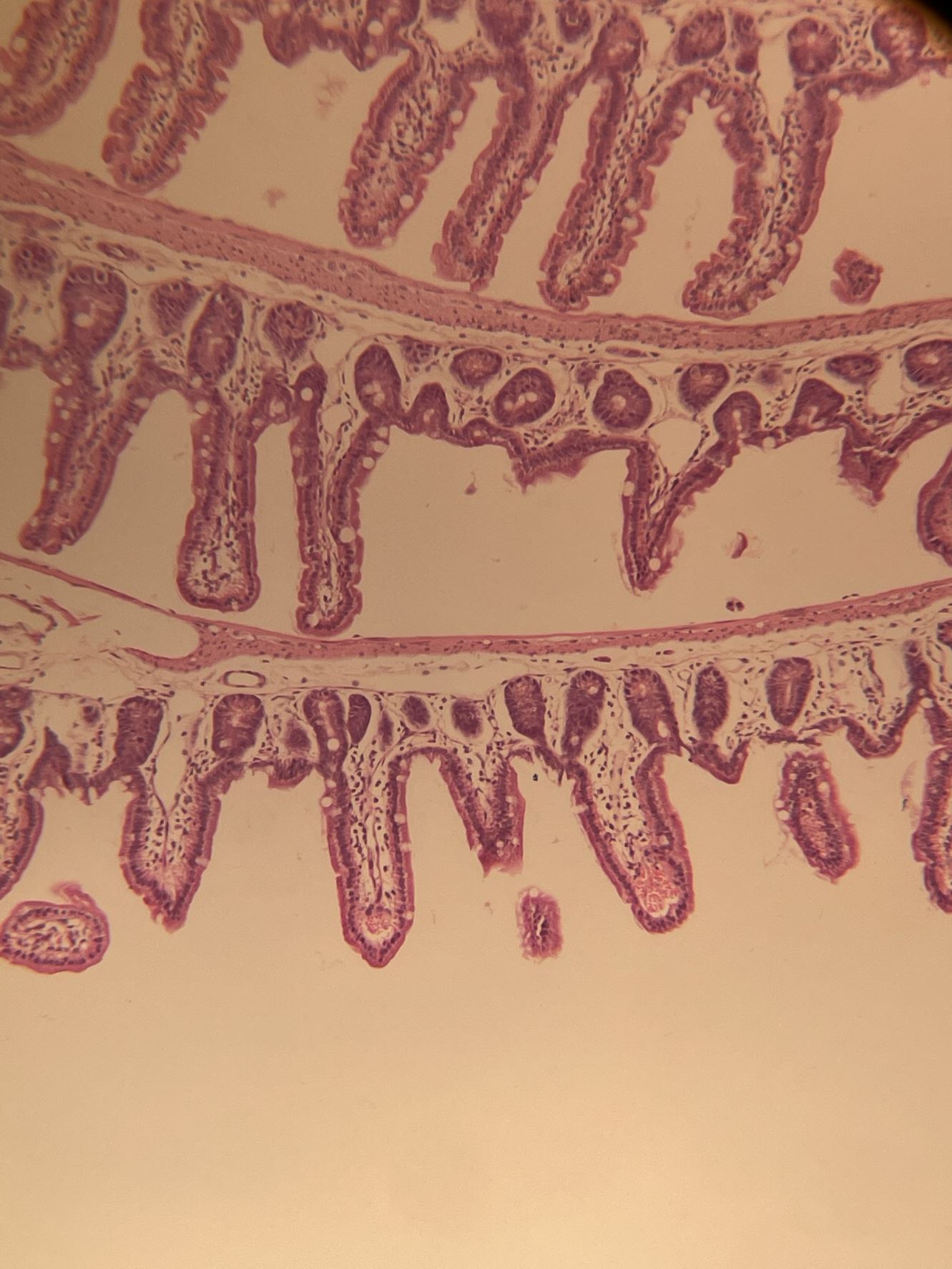

Supplement: Supplementary file 5 — Source Data for Expanded View and Appendix [file EMBR-24-e56030-s011.zip › Figure EV1-5, Appendix Figure S1-4/3. Figure EV3/Figure EV3B,C,E-G HE staining in different mouse strains/F. LEC-Foxc1-KO.jpg]

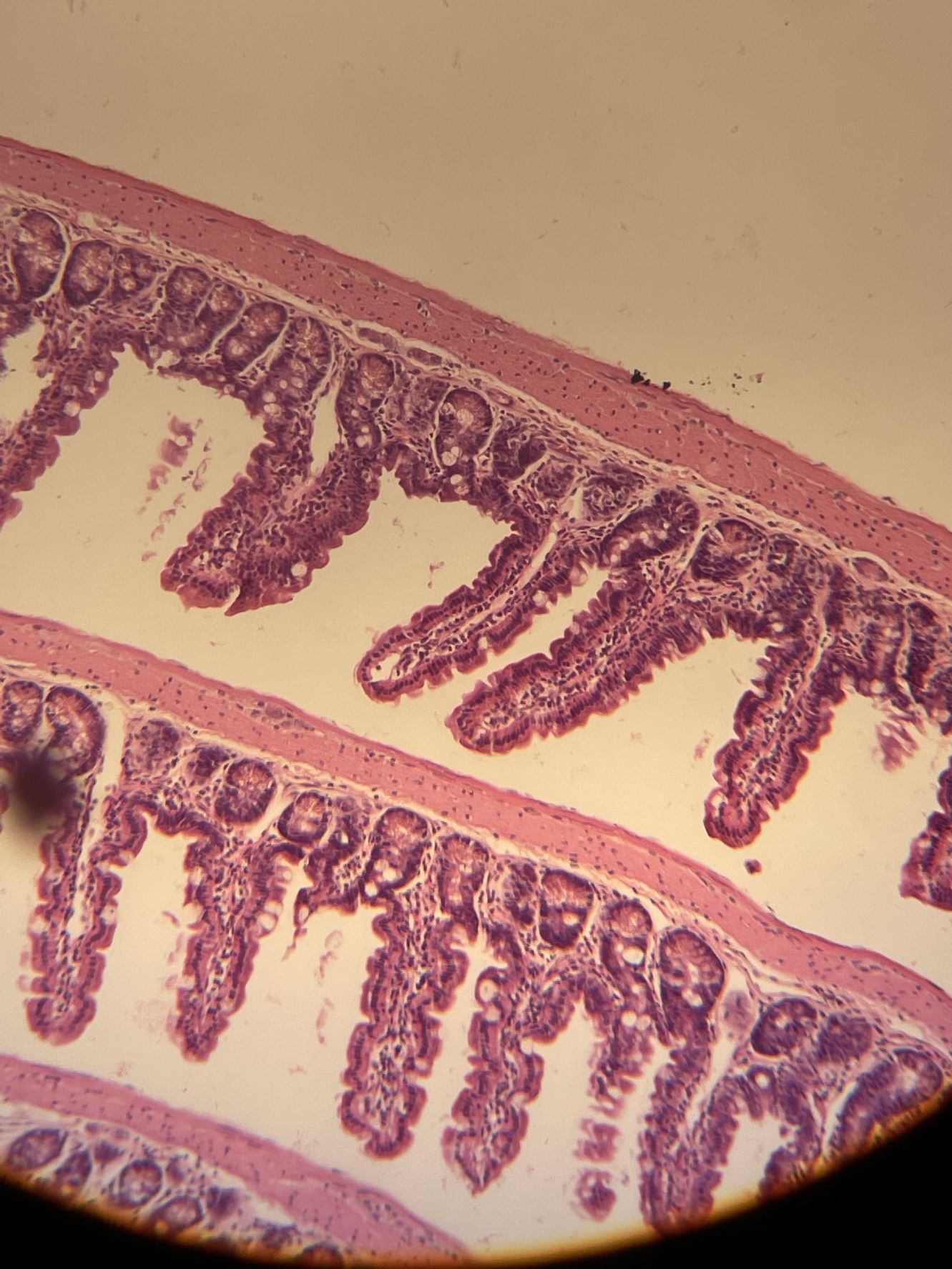

Supplement: Supplementary file 5 — Source Data for Expanded View and Appendix [file EMBR-24-e56030-s011.zip › Figure EV1-5, Appendix Figure S1-4/3. Figure EV3/Figure EV3B,C,E-G HE staining in different mouse strains/G. Control.jpg]

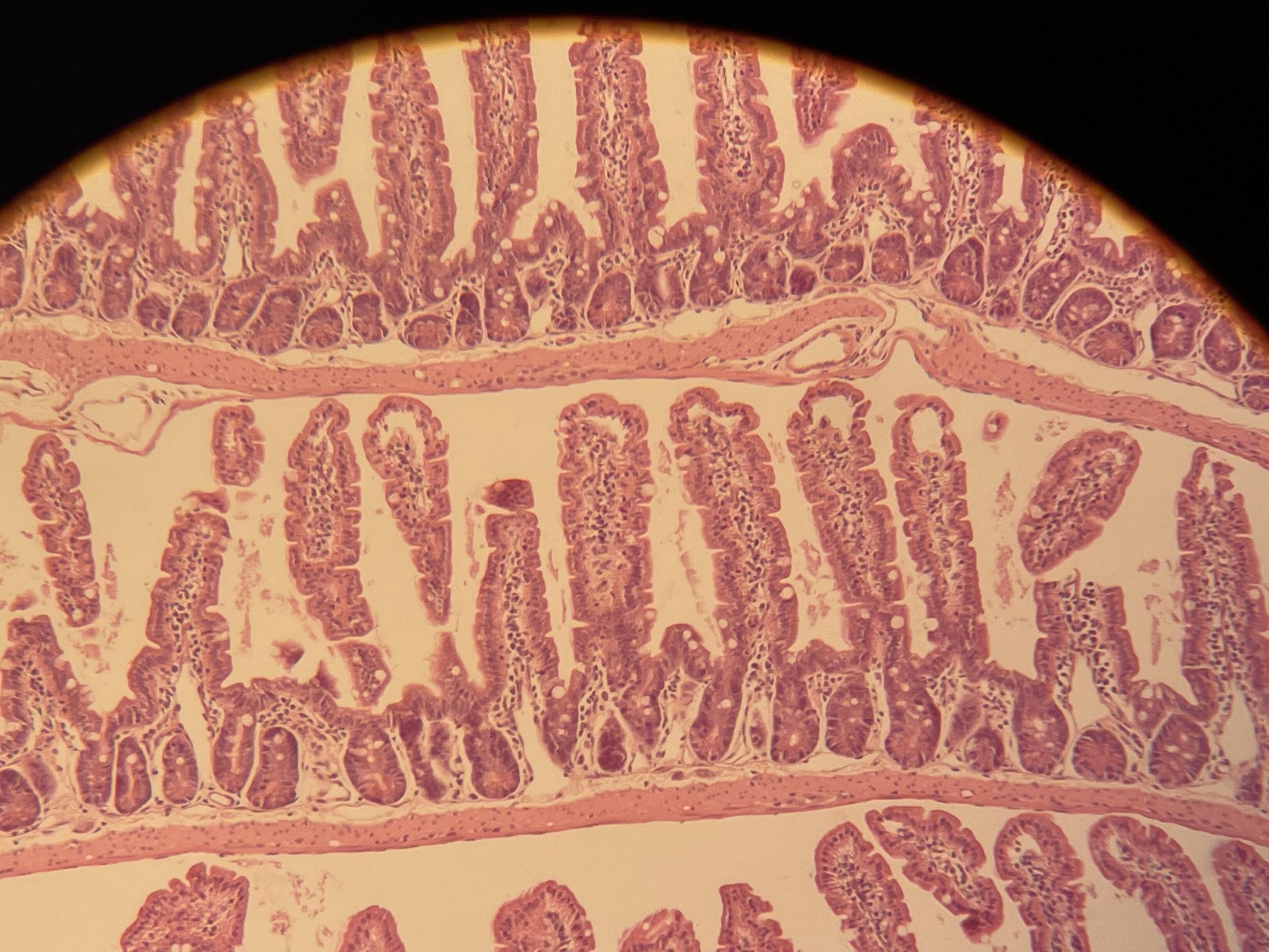

Supplement: Supplementary file 5 — Source Data for Expanded View and Appendix [file EMBR-24-e56030-s011.zip › Figure EV1-5, Appendix Figure S1-4/3. Figure EV3/Figure EV3B,C,E-G HE staining in different mouse strains/G. LEC-Foxc2-KO.jpg]

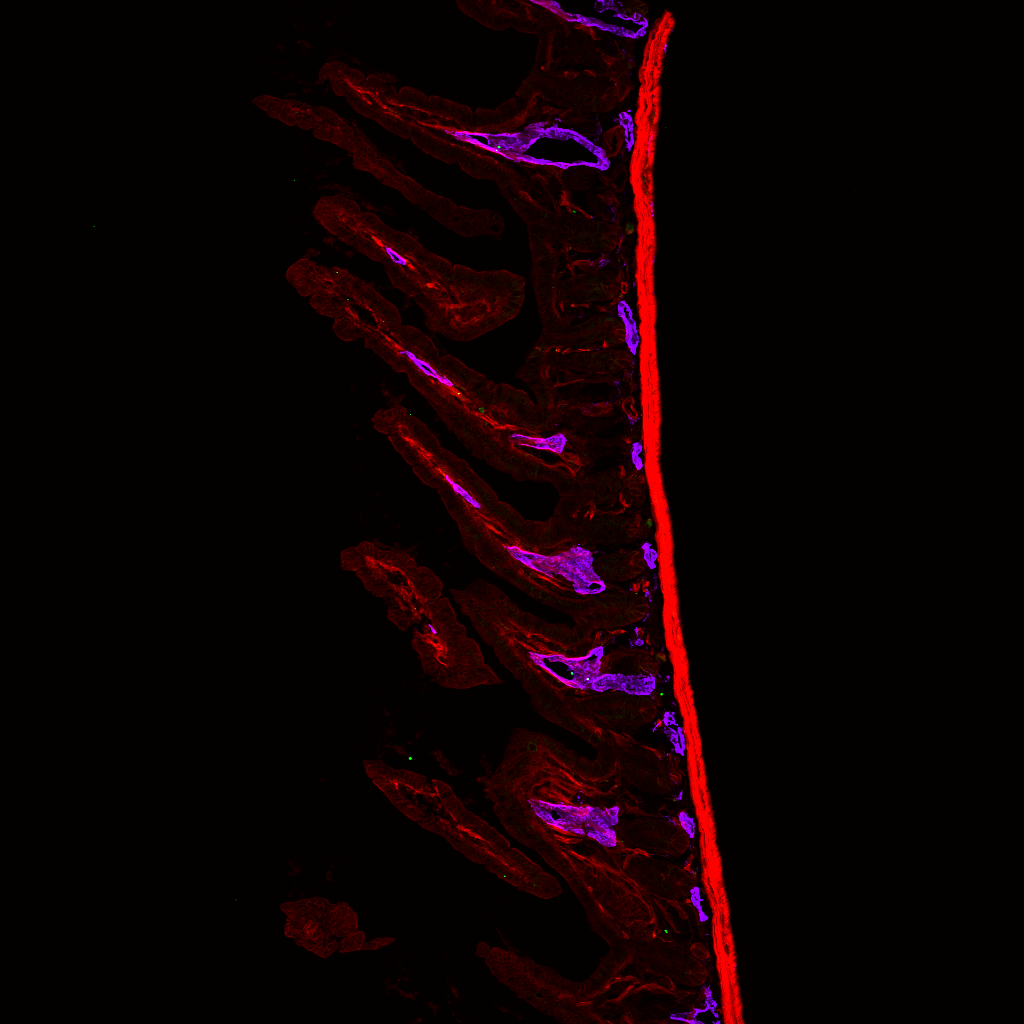

Supplement: Supplementary file 5 — Source Data for Expanded View and Appendix [file EMBR-24-e56030-s011.zip › Figure EV1-5, Appendix Figure S1-4/3. Figure EV3/Figure EV3D-IHC mTmG, LYVE1/1. Control, 3 colors.tif]

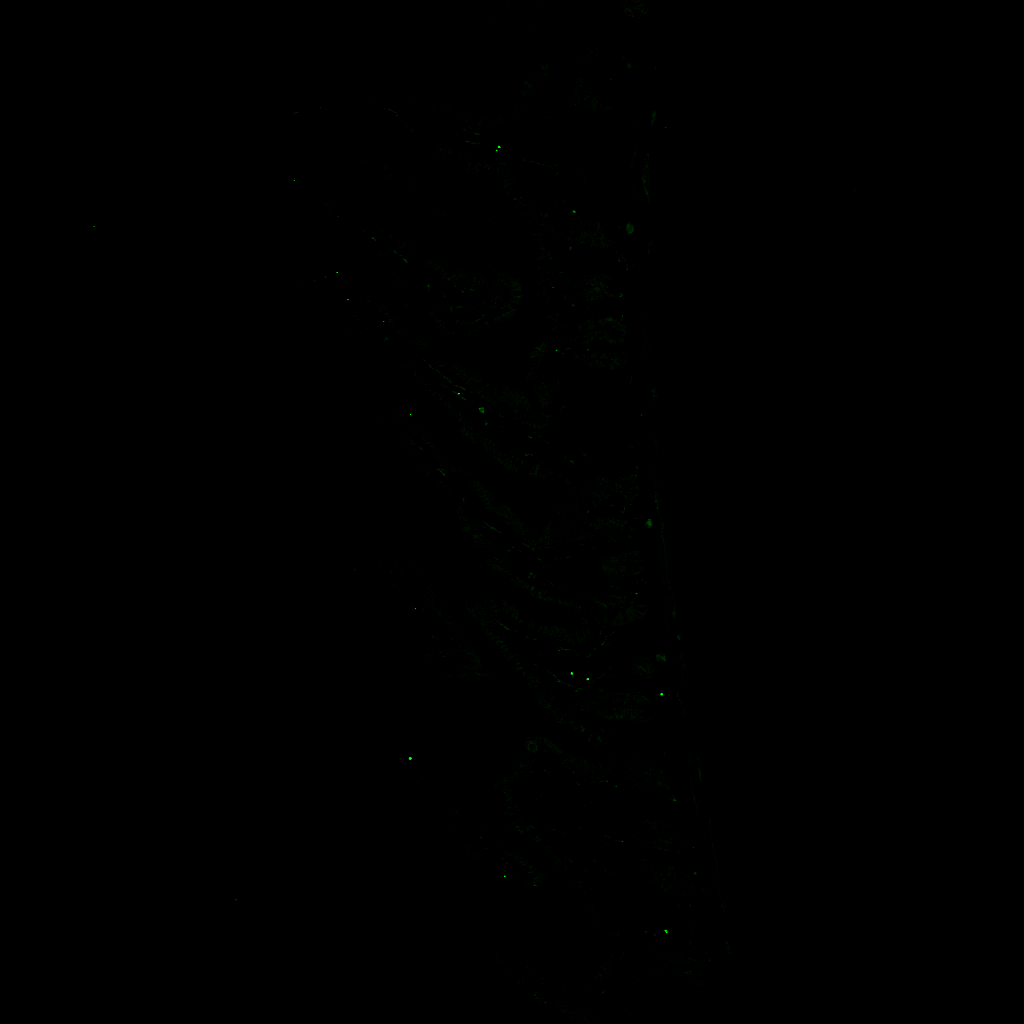

Supplement: Supplementary file 5 — Source Data for Expanded View and Appendix [file EMBR-24-e56030-s011.zip › Figure EV1-5, Appendix Figure S1-4/3. Figure EV3/Figure EV3D-IHC mTmG, LYVE1/2. Control, GFP.tif]

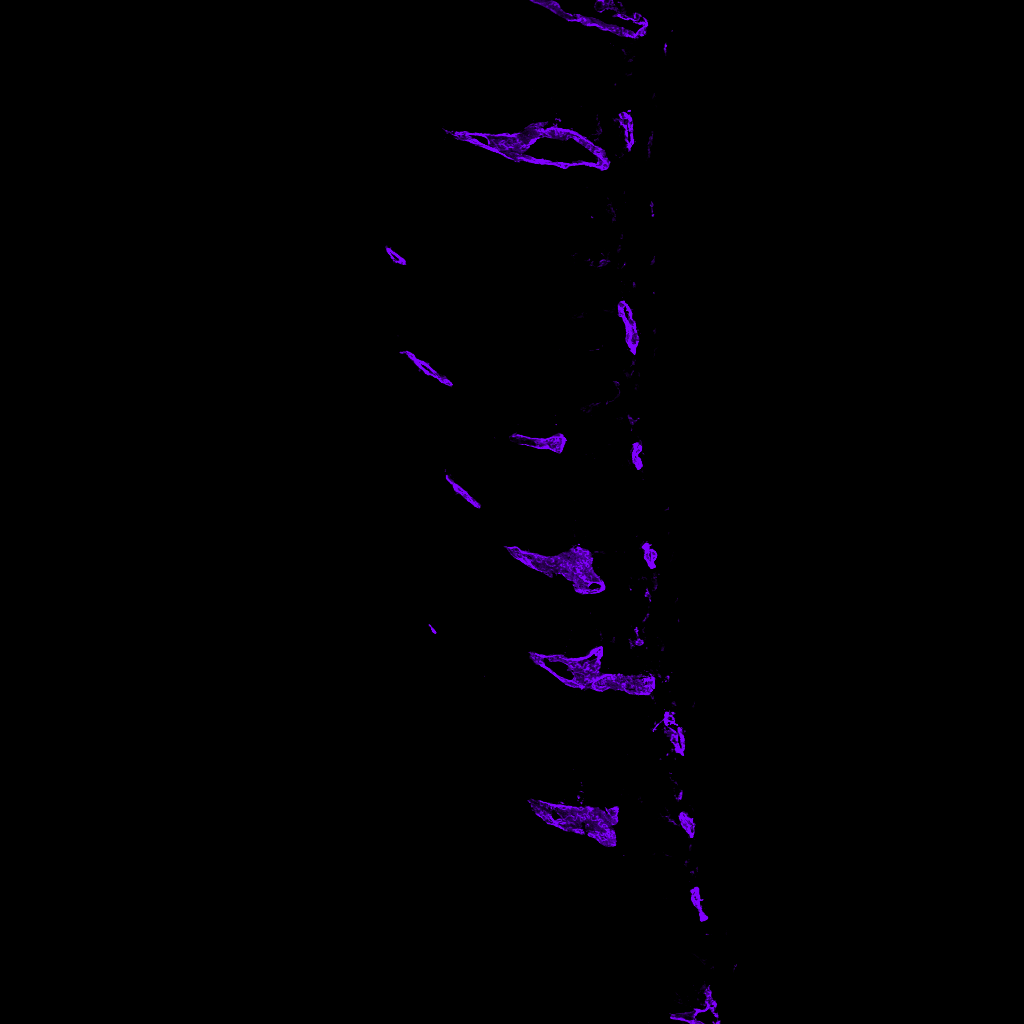

Supplement: Supplementary file 5 — Source Data for Expanded View and Appendix [file EMBR-24-e56030-s011.zip › Figure EV1-5, Appendix Figure S1-4/3. Figure EV3/Figure EV3D-IHC mTmG, LYVE1/3. Control, LYVE1.tif]

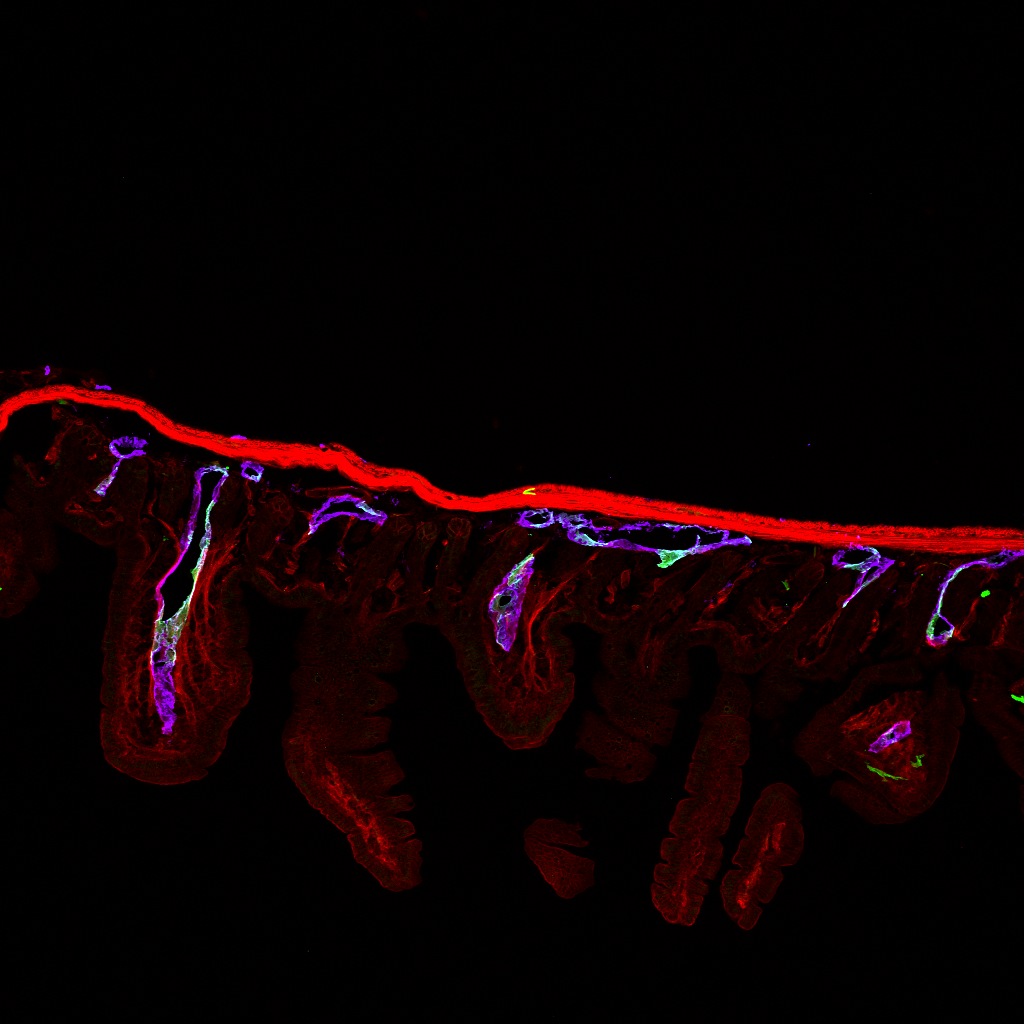

Supplement: Supplementary file 5 — Source Data for Expanded View and Appendix [file EMBR-24-e56030-s011.zip › Figure EV1-5, Appendix Figure S1-4/3. Figure EV3/Figure EV3D-IHC mTmG, LYVE1/4. mTmG;LEC-Foxc-DKO, 3 colors.tif]

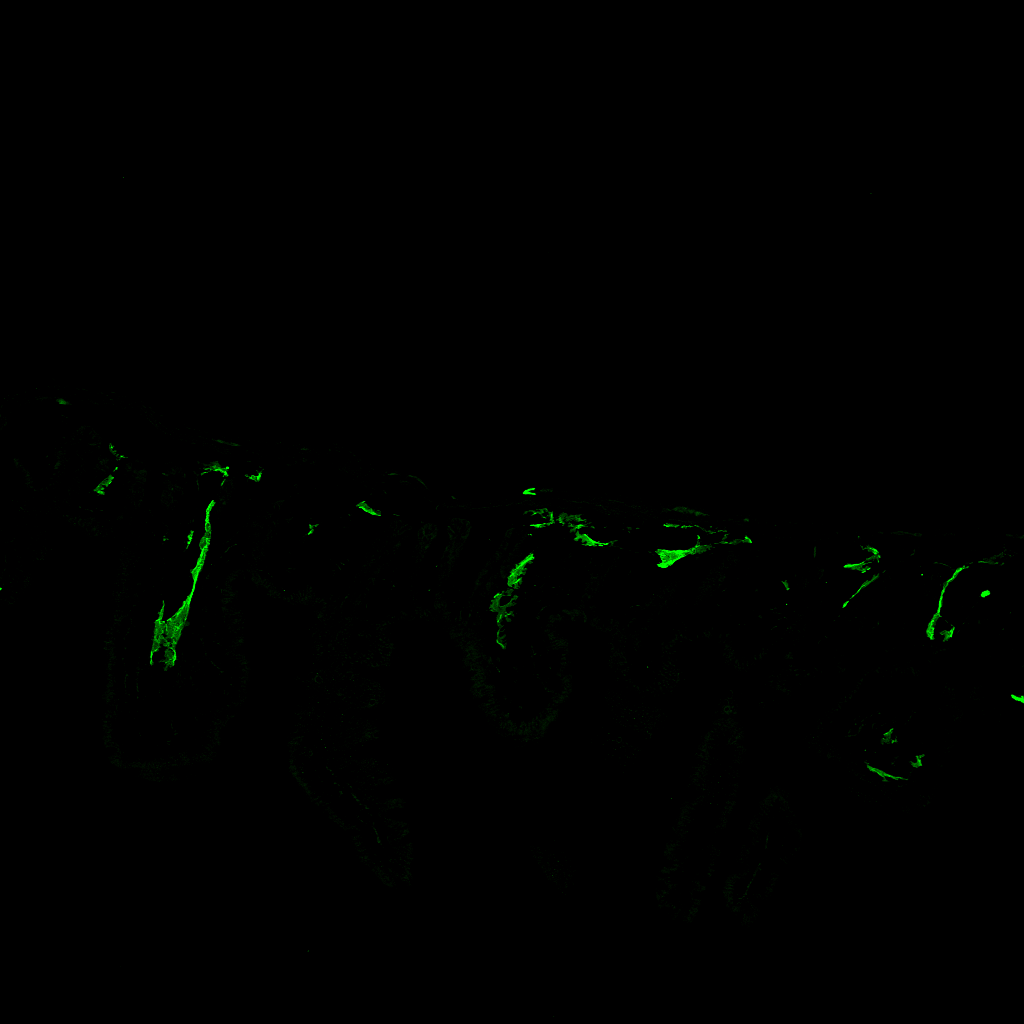

Supplement: Supplementary file 5 — Source Data for Expanded View and Appendix [file EMBR-24-e56030-s011.zip › Figure EV1-5, Appendix Figure S1-4/3. Figure EV3/Figure EV3D-IHC mTmG, LYVE1/5. mTmG;LEC-Foxc-DKO, GFP.tif]

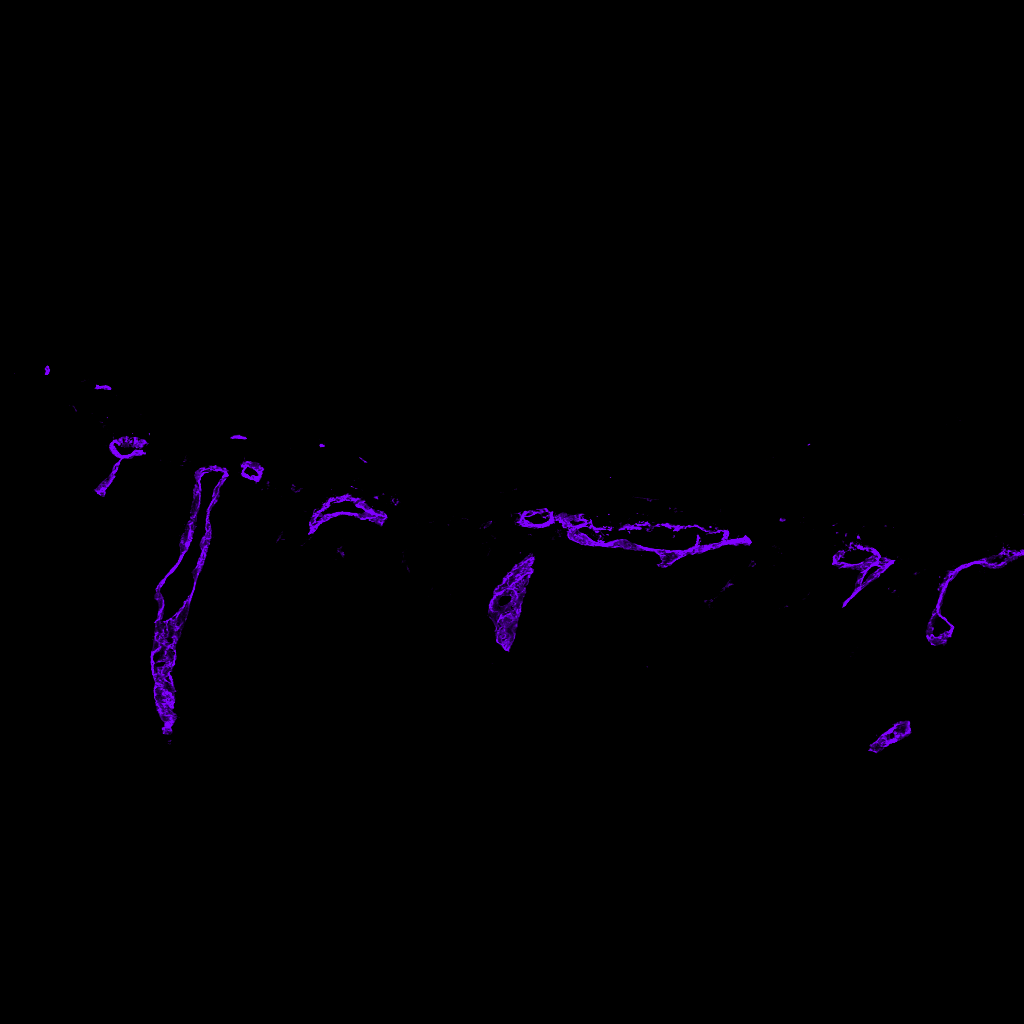

Supplement: Supplementary file 5 — Source Data for Expanded View and Appendix [file EMBR-24-e56030-s011.zip › Figure EV1-5, Appendix Figure S1-4/3. Figure EV3/Figure EV3D-IHC mTmG, LYVE1/6. mTmG;LEC-Foxc-DKO, LYVE1.tif]

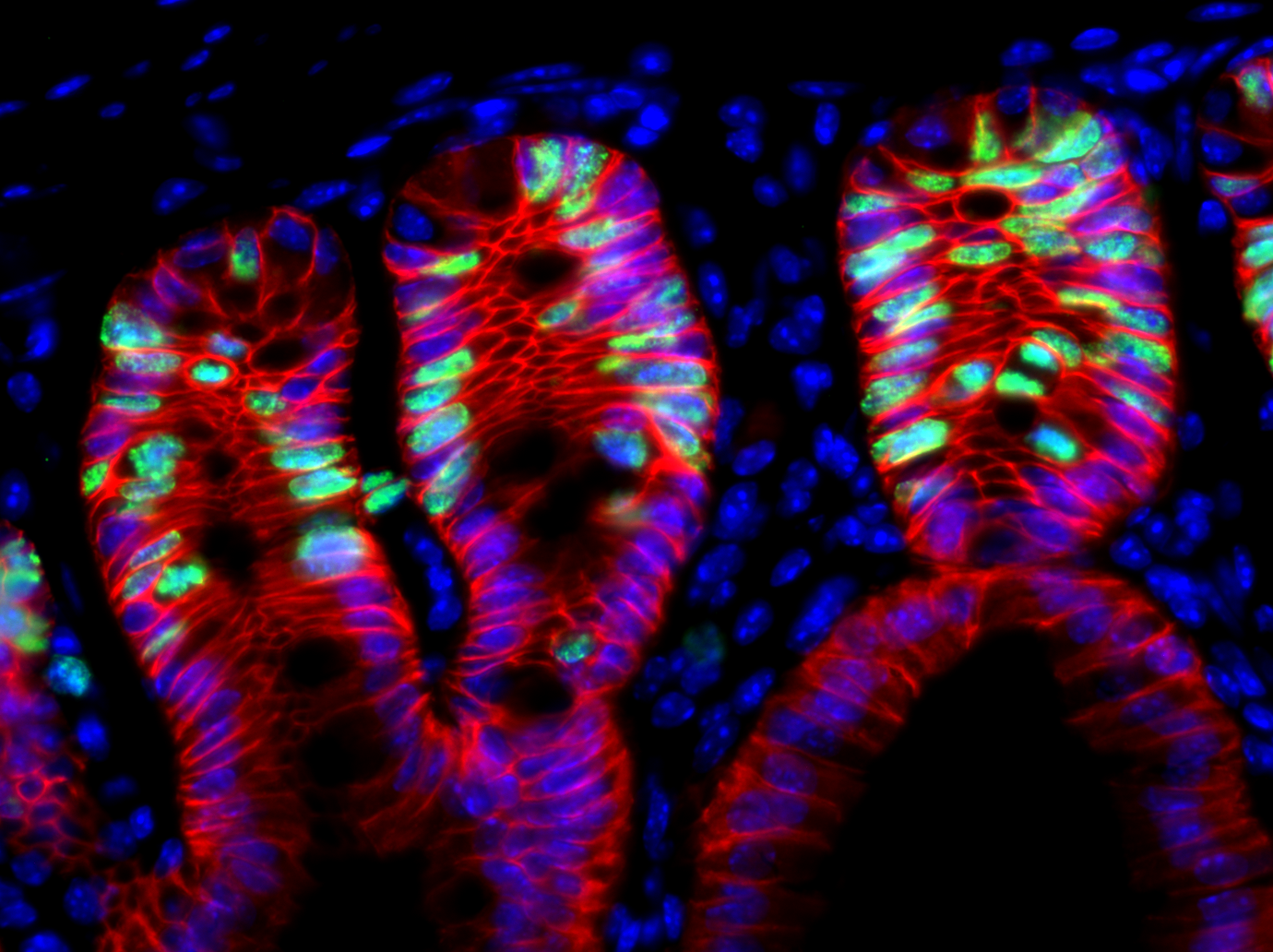

Supplement: Supplementary file 5 — Source Data for Expanded View and Appendix [file EMBR-24-e56030-s011.zip › Figure EV1-5, Appendix Figure S1-4/3. Figure EV3/Figure EV3H-IHC BrdU EpCAM/Control.TIF]

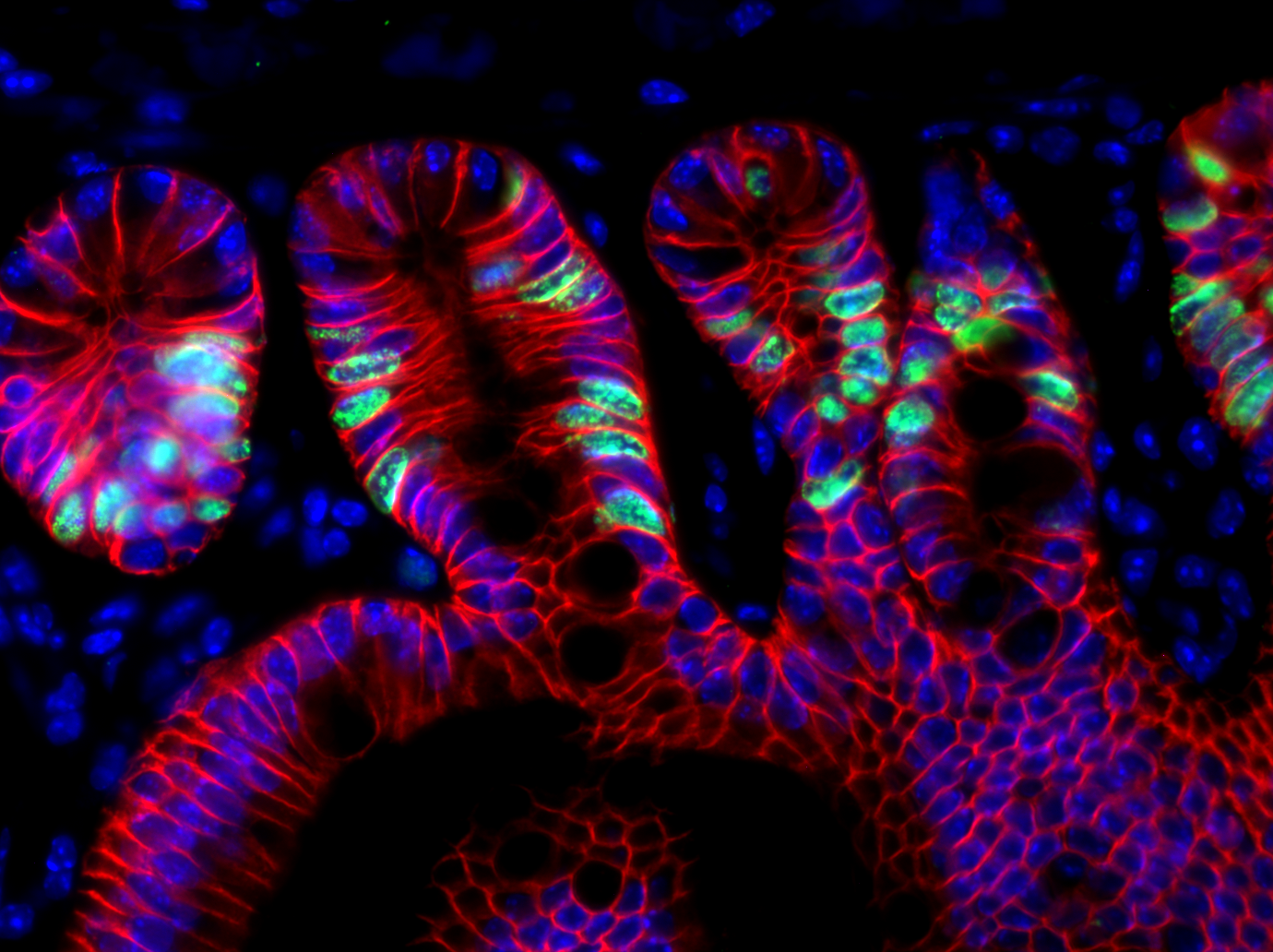

Supplement: Supplementary file 5 — Source Data for Expanded View and Appendix [file EMBR-24-e56030-s011.zip › Figure EV1-5, Appendix Figure S1-4/3. Figure EV3/Figure EV3H-IHC BrdU EpCAM/LEC-Foxc-DKO.TIF]

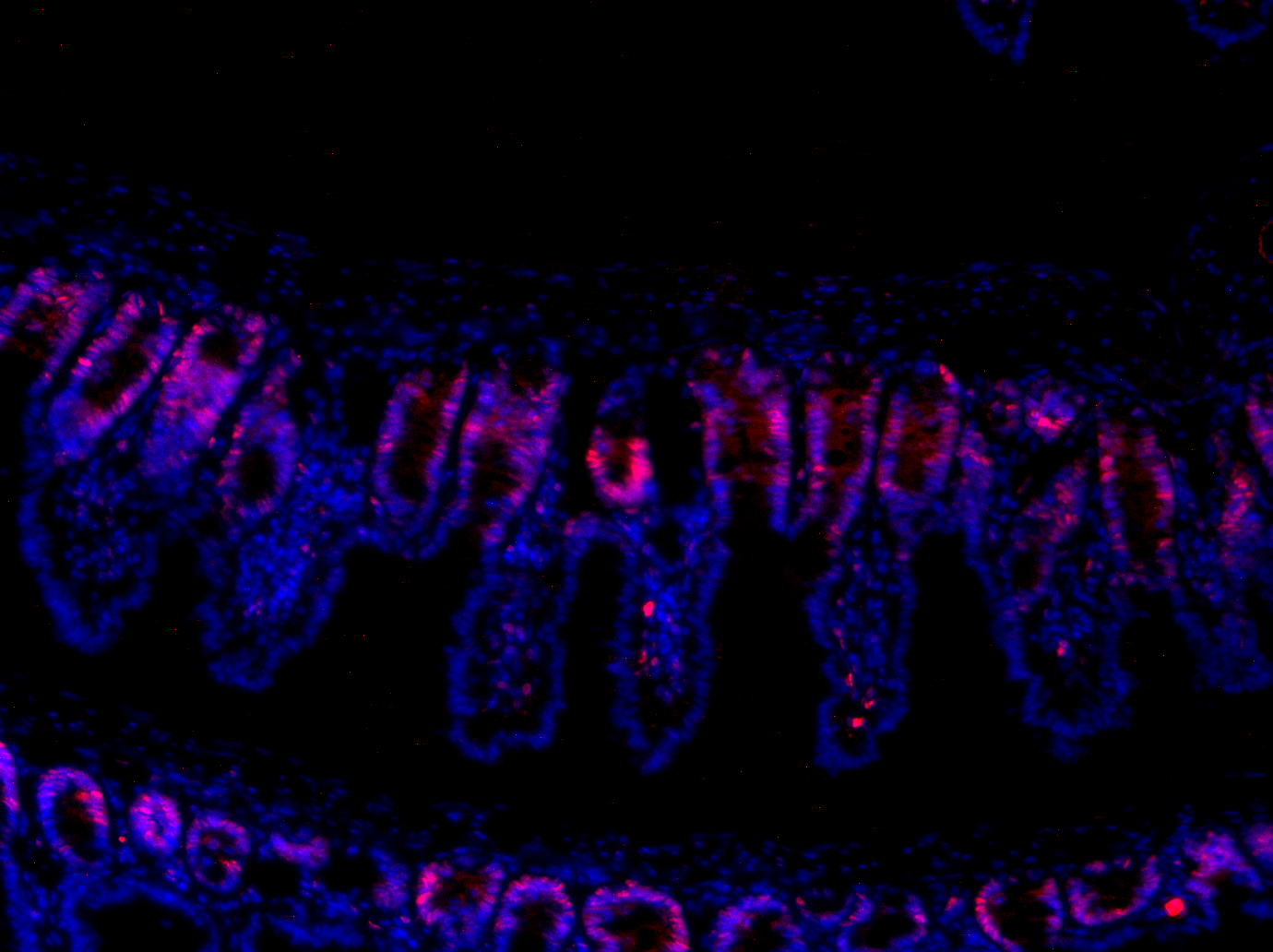

Supplement: Supplementary file 5 — Source Data for Expanded View and Appendix [file EMBR-24-e56030-s011.zip › Figure EV1-5, Appendix Figure S1-4/3. Figure EV3/Figure EV3I-IHC CCND1/1. Control, CCND1 DAPI.TIF]

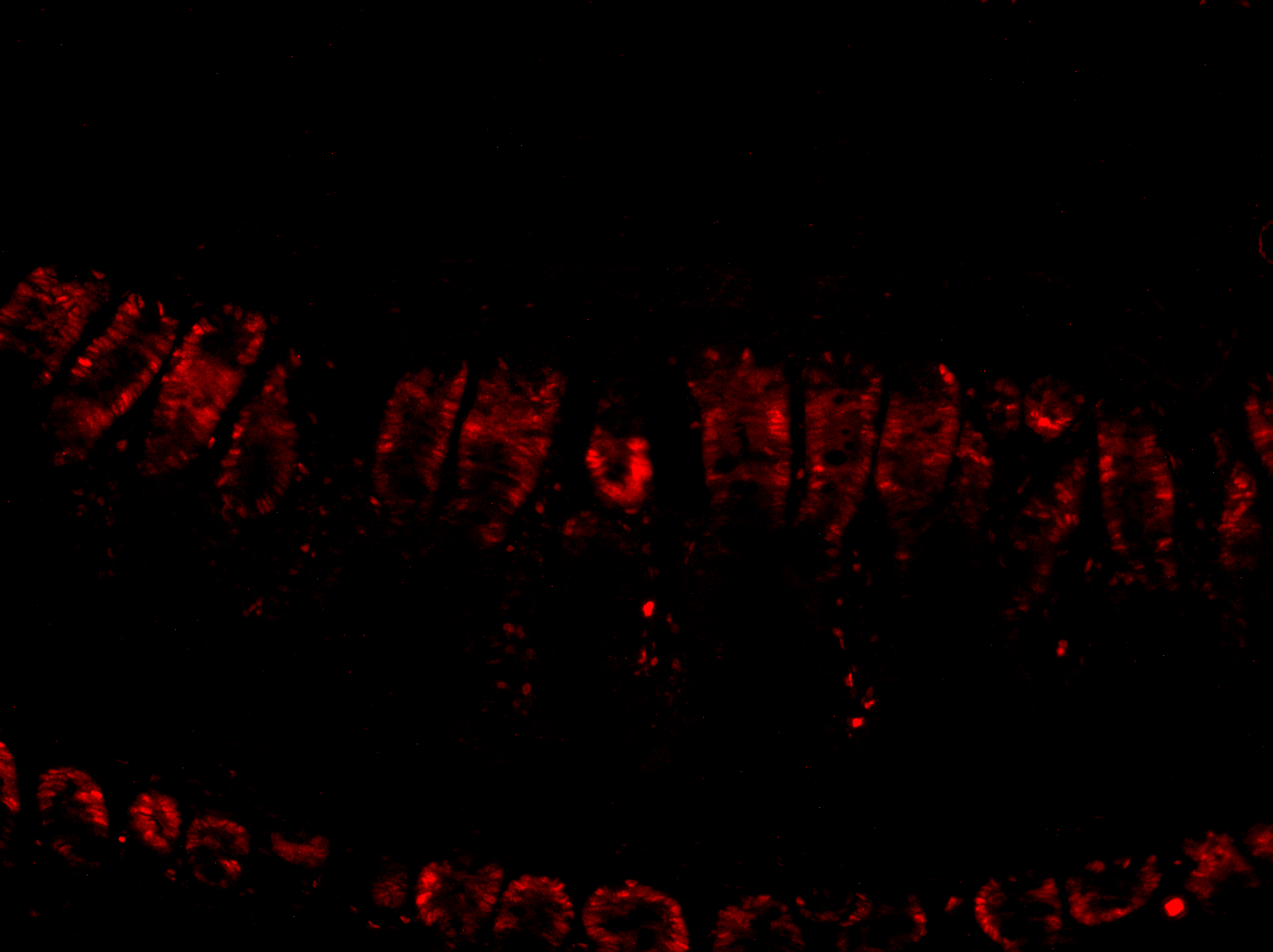

Supplement: Supplementary file 5 — Source Data for Expanded View and Appendix [file EMBR-24-e56030-s011.zip › Figure EV1-5, Appendix Figure S1-4/3. Figure EV3/Figure EV3I-IHC CCND1/2. Control, CCND1.TIF]

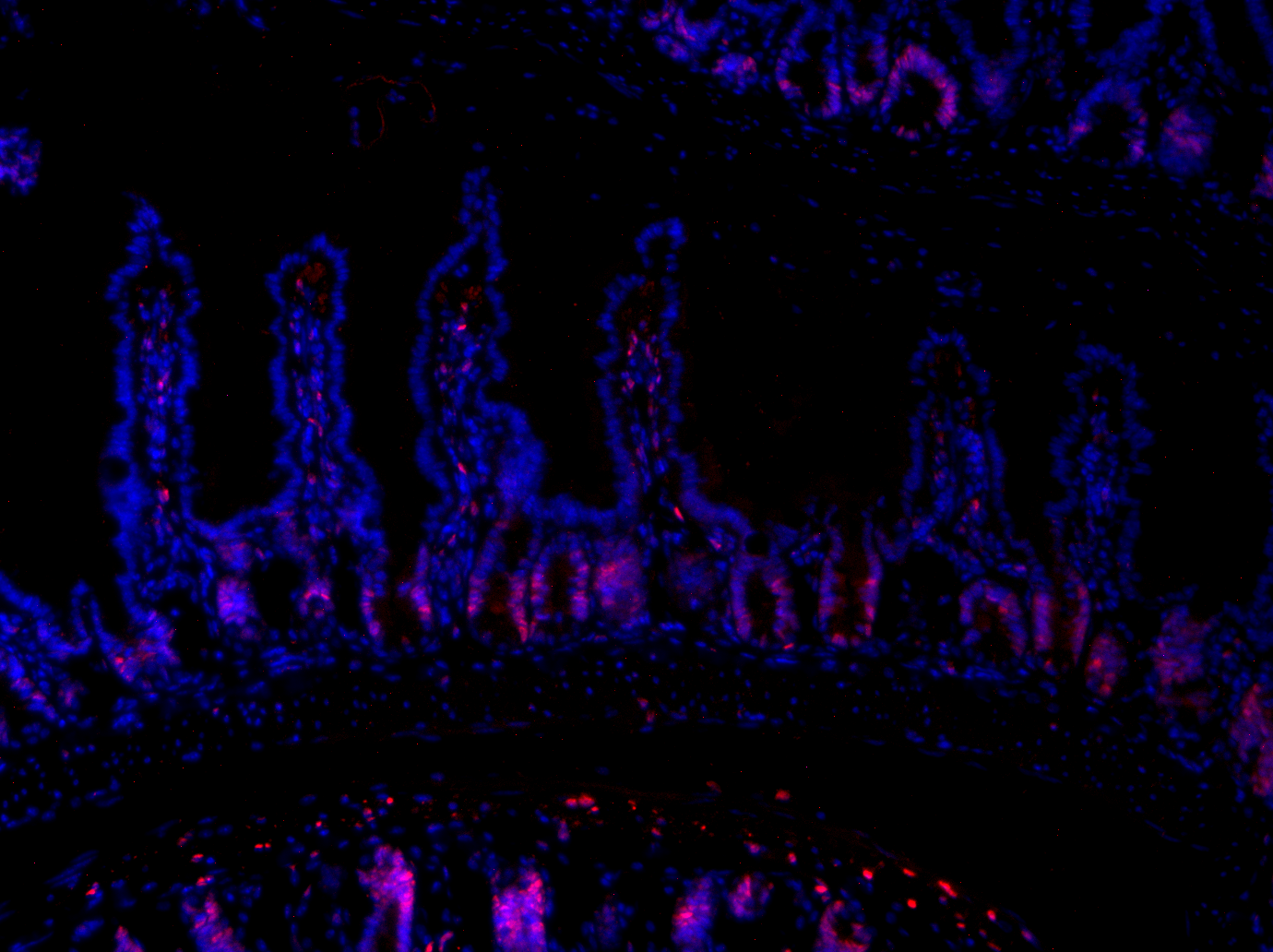

Supplement: Supplementary file 5 — Source Data for Expanded View and Appendix [file EMBR-24-e56030-s011.zip › Figure EV1-5, Appendix Figure S1-4/3. Figure EV3/Figure EV3I-IHC CCND1/3. LEC-Foxc-DKO, CCND1, DAPI.TIF]

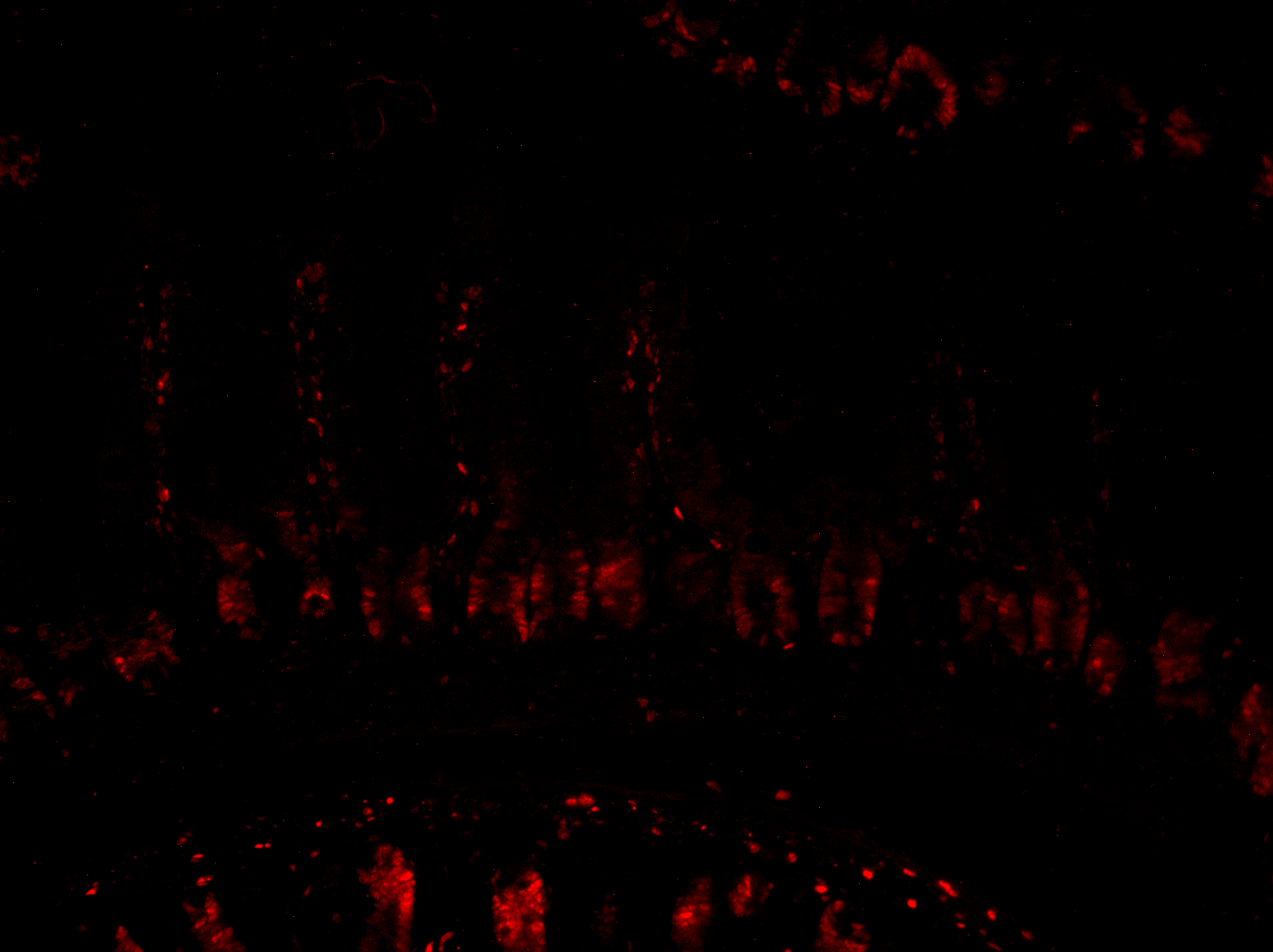

Supplement: Supplementary file 5 — Source Data for Expanded View and Appendix [file EMBR-24-e56030-s011.zip › Figure EV1-5, Appendix Figure S1-4/3. Figure EV3/Figure EV3I-IHC CCND1/4. LEC-Foxc-DKO, CCND1.TIF]

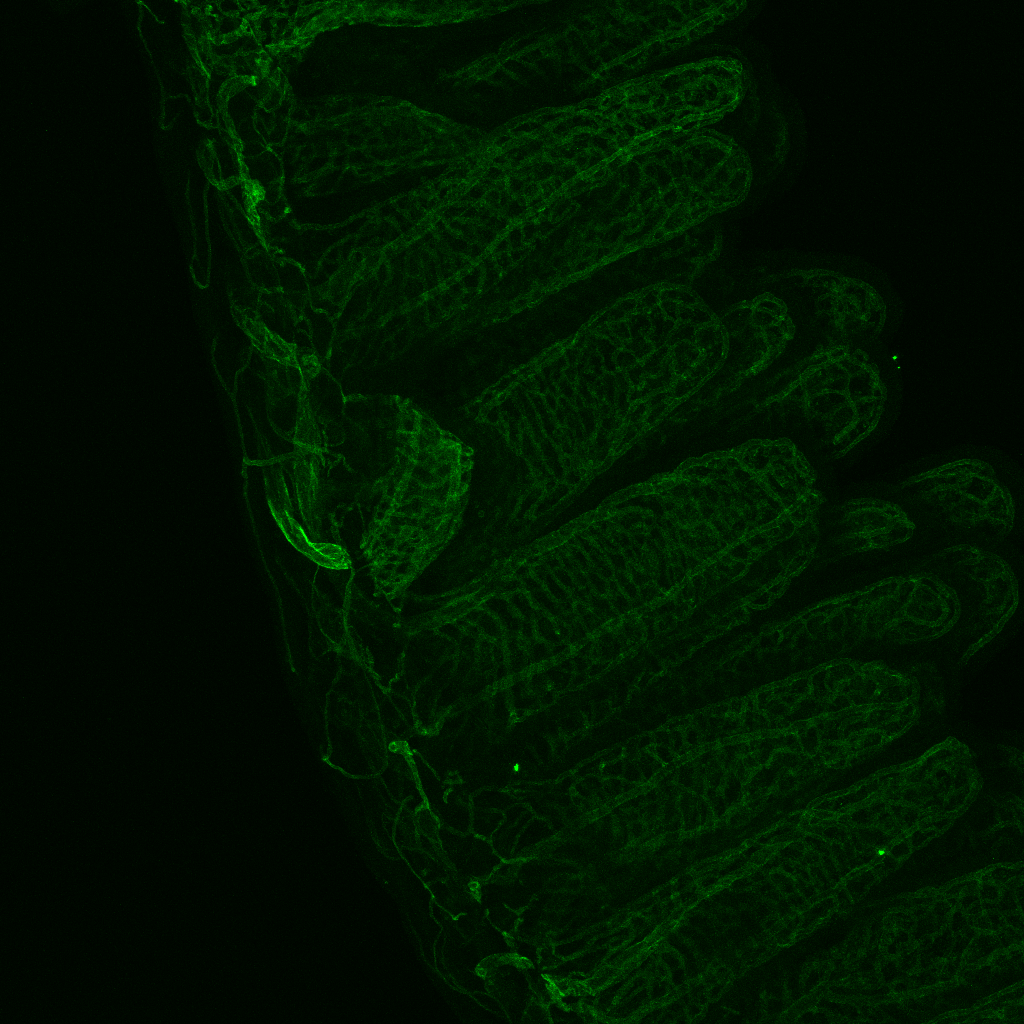

Supplement: Supplementary file 5 — Source Data for Expanded View and Appendix [file EMBR-24-e56030-s011.zip › Figure EV1-5, Appendix Figure S1-4/4. Figure EV4/Figure EV4A-WM-CD31/1. Control, sham.tif]

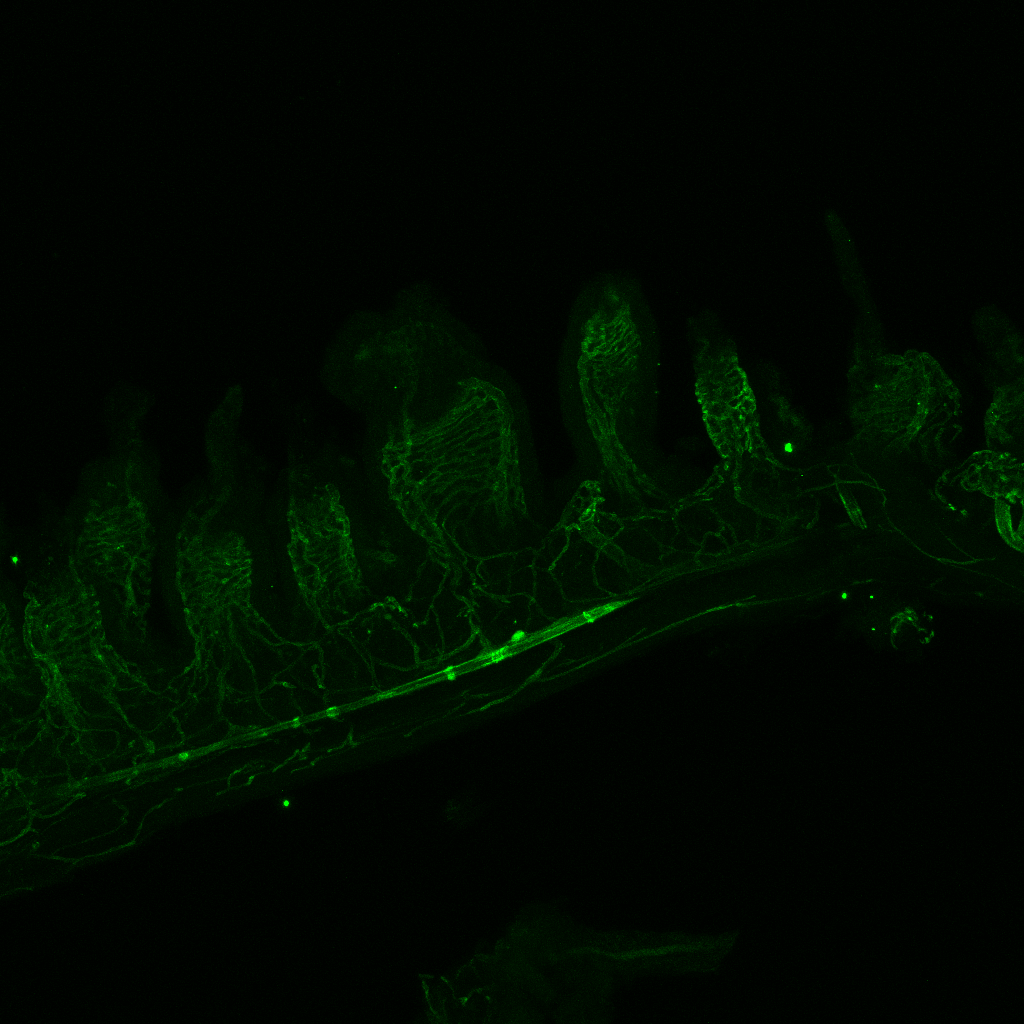

Supplement: Supplementary file 5 — Source Data for Expanded View and Appendix [file EMBR-24-e56030-s011.zip › Figure EV1-5, Appendix Figure S1-4/4. Figure EV4/Figure EV4A-WM-CD31/2. Control, IR-4h.tif]

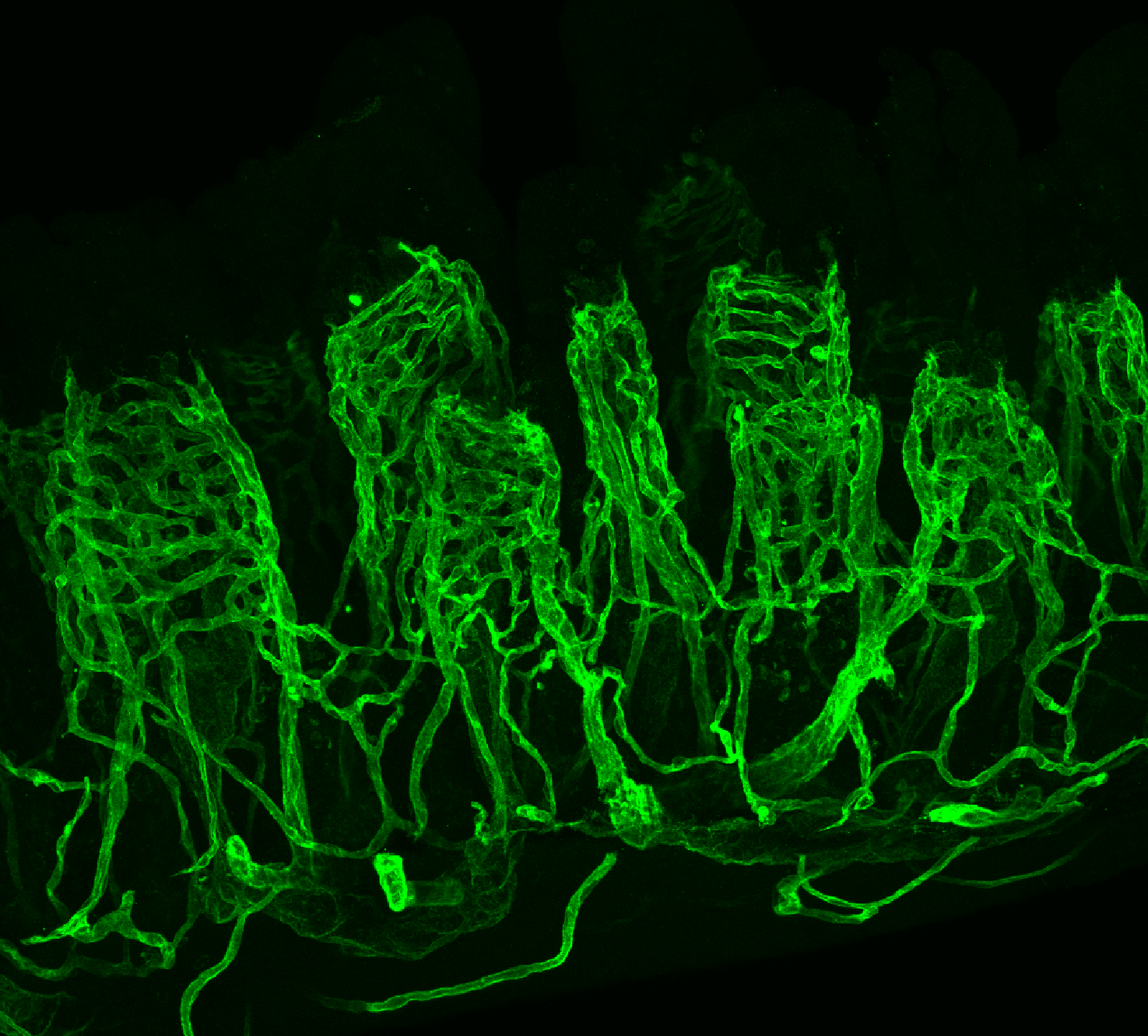

Supplement: Supplementary file 5 — Source Data for Expanded View and Appendix [file EMBR-24-e56030-s011.zip › Figure EV1-5, Appendix Figure S1-4/4. Figure EV4/Figure EV4A-WM-CD31/3. Control, IR-18.5h.tif]

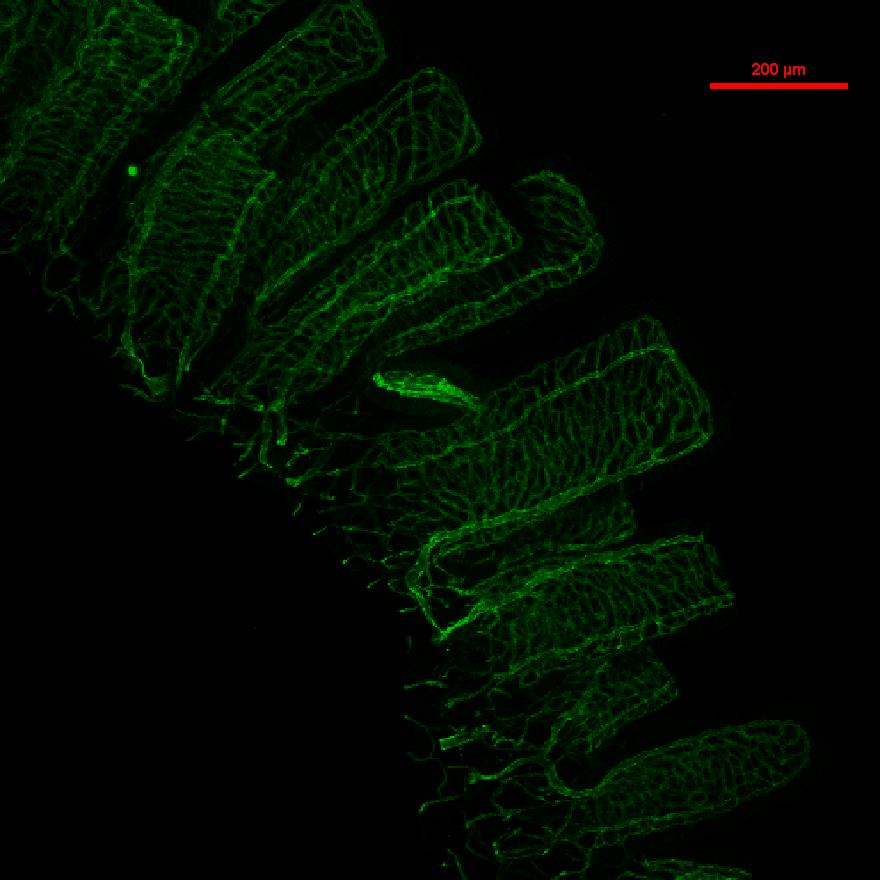

Supplement: Supplementary file 5 — Source Data for Expanded View and Appendix [file EMBR-24-e56030-s011.zip › Figure EV1-5, Appendix Figure S1-4/4. Figure EV4/Figure EV4A-WM-CD31/4. EC-Foxc-DKO, sham.tif]

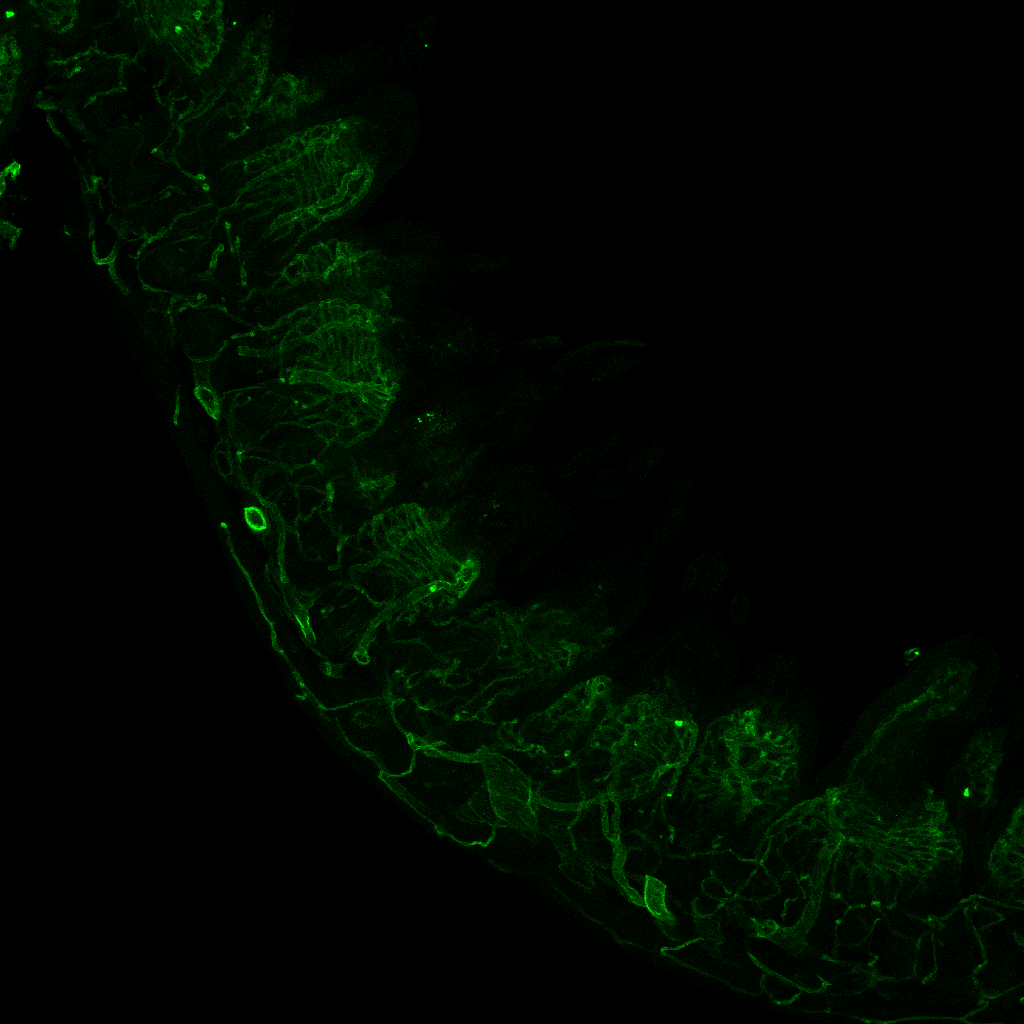

Supplement: Supplementary file 5 — Source Data for Expanded View and Appendix [file EMBR-24-e56030-s011.zip › Figure EV1-5, Appendix Figure S1-4/4. Figure EV4/Figure EV4A-WM-CD31/5. EC-Foxc-DKO, IR-4h.tif]

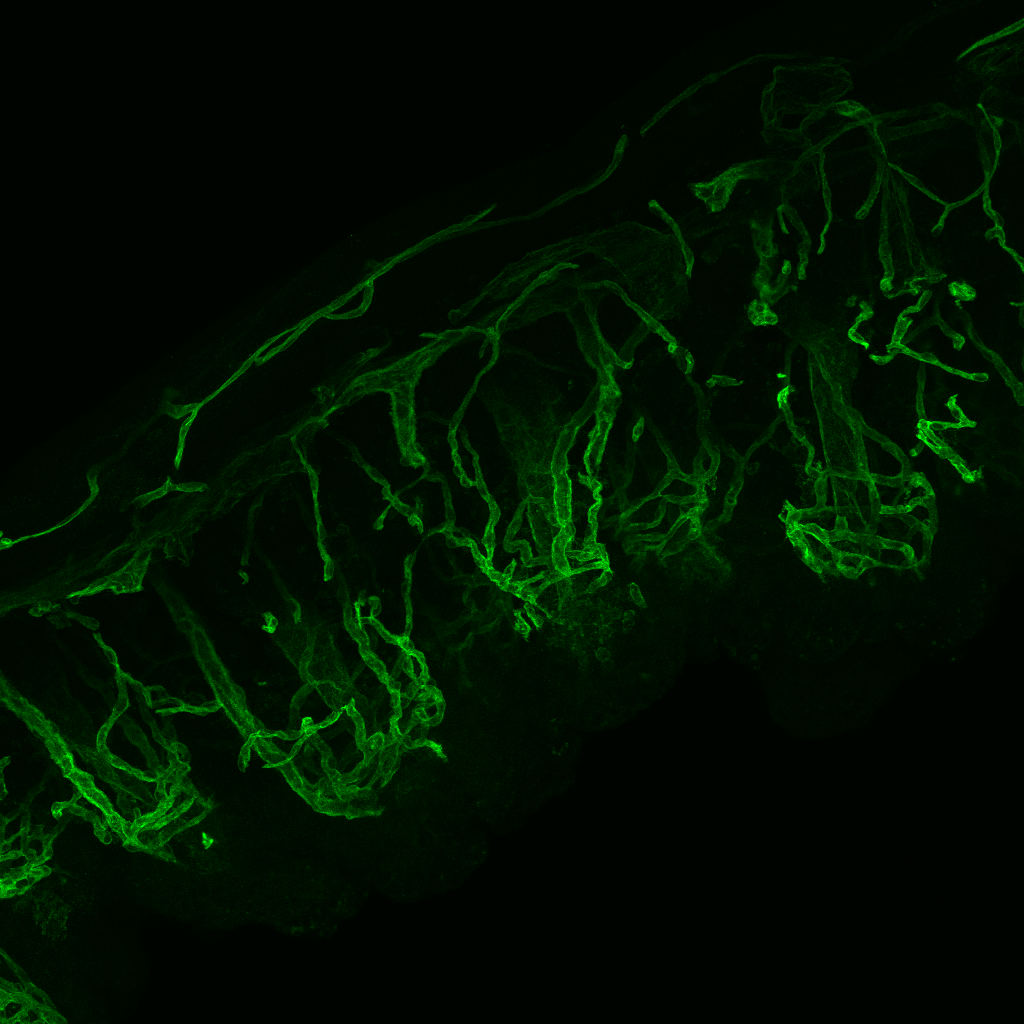

Supplement: Supplementary file 5 — Source Data for Expanded View and Appendix [file EMBR-24-e56030-s011.zip › Figure EV1-5, Appendix Figure S1-4/4. Figure EV4/Figure EV4A-WM-CD31/6. EC-Foxc-DKO, IR-18.5h.tif]

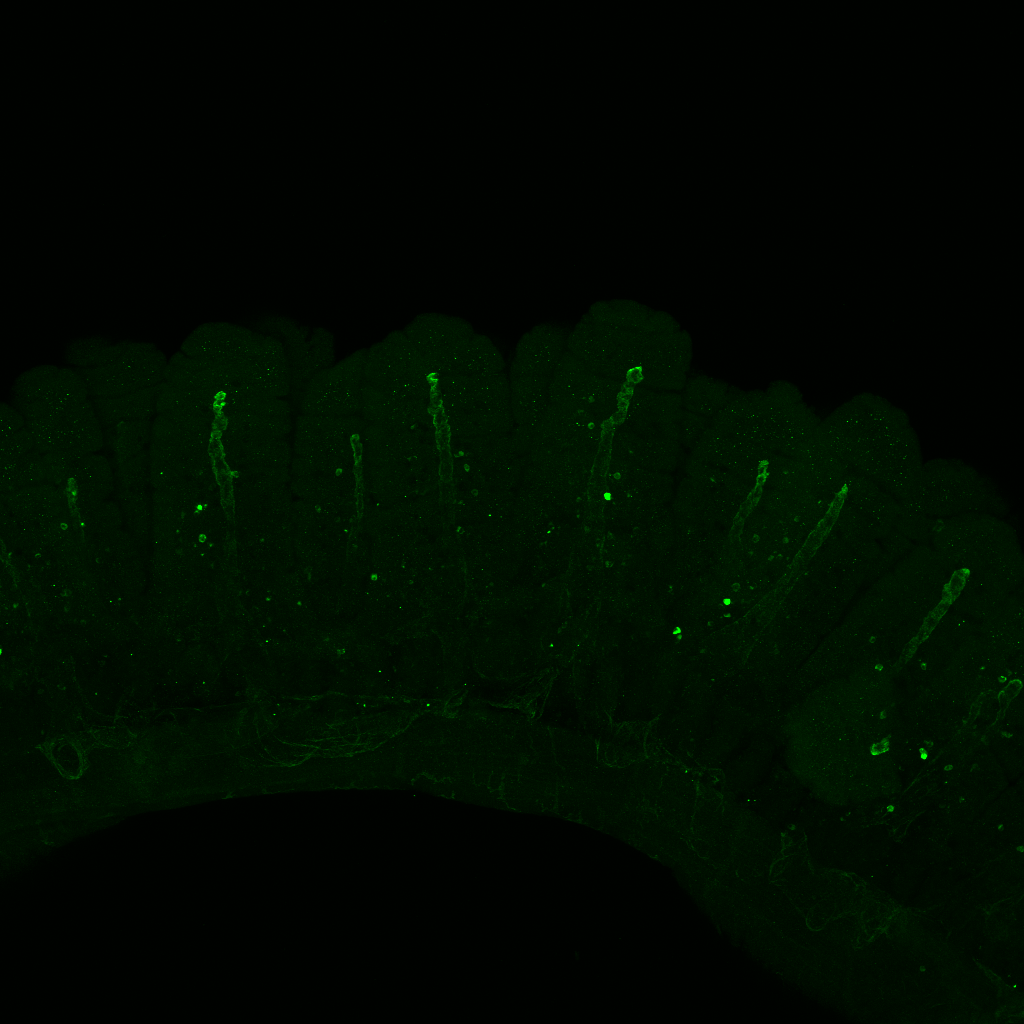

Supplement: Supplementary file 5 — Source Data for Expanded View and Appendix [file EMBR-24-e56030-s011.zip › Figure EV1-5, Appendix Figure S1-4/4. Figure EV4/Figure EV4B-WM LYVE1/1. Control, sham.tif]

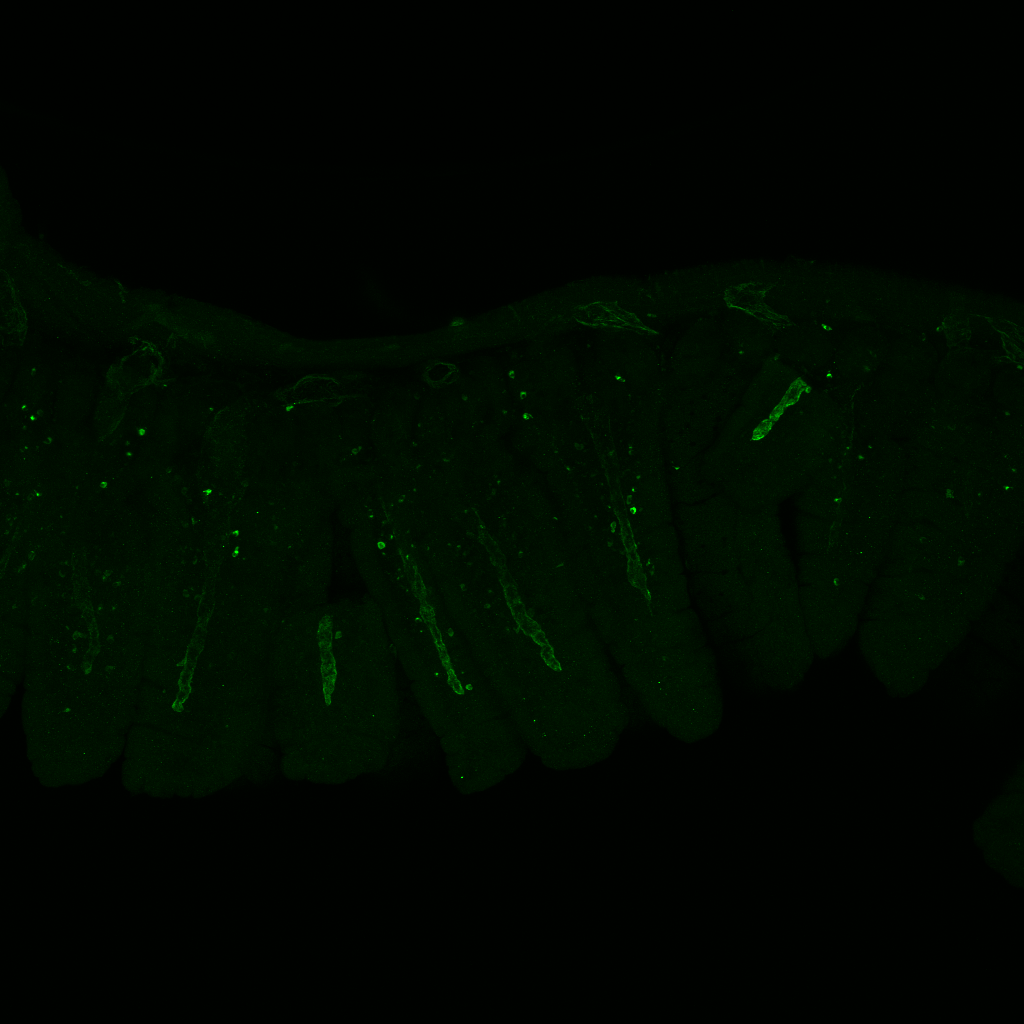

Supplement: Supplementary file 5 — Source Data for Expanded View and Appendix [file EMBR-24-e56030-s011.zip › Figure EV1-5, Appendix Figure S1-4/4. Figure EV4/Figure EV4B-WM LYVE1/2. EC-Foxc-DKO, sham.tif]

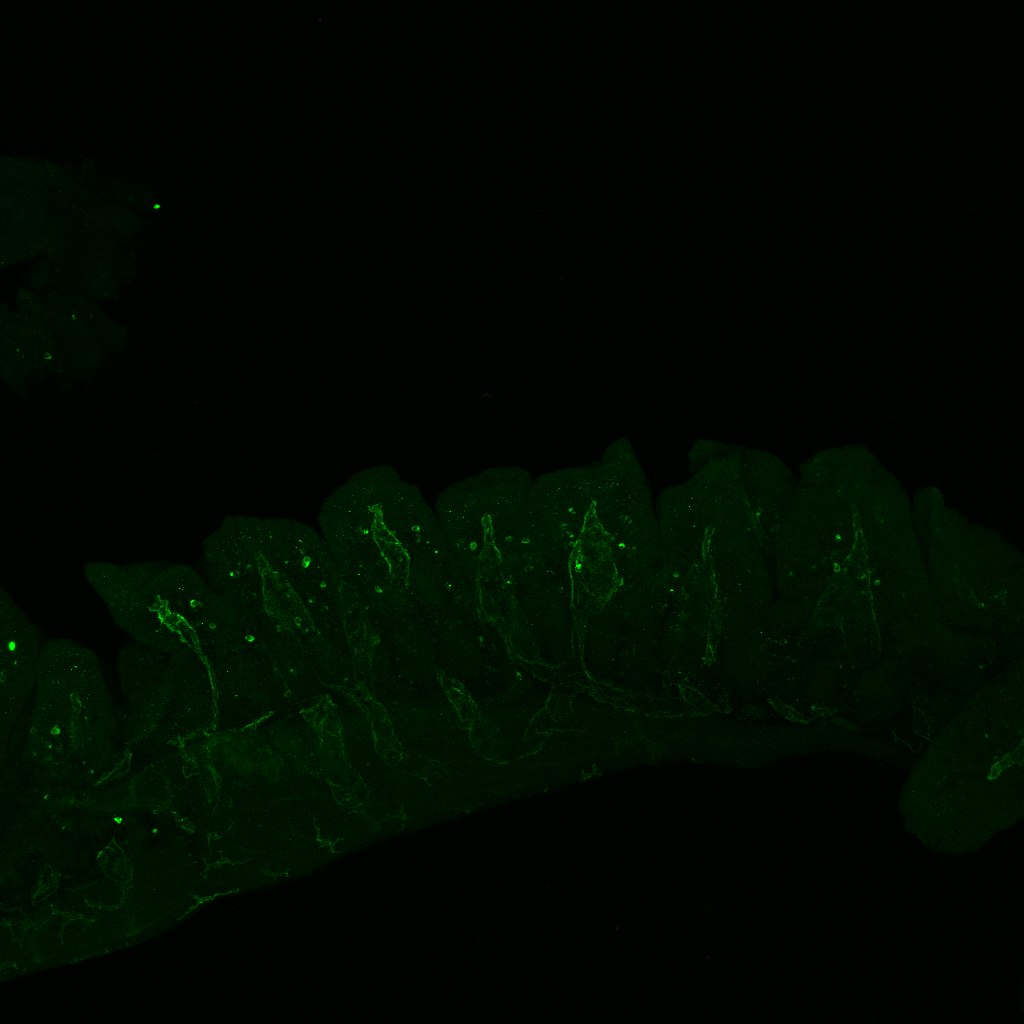

Supplement: Supplementary file 5 — Source Data for Expanded View and Appendix [file EMBR-24-e56030-s011.zip › Figure EV1-5, Appendix Figure S1-4/4. Figure EV4/Figure EV4B-WM LYVE1/3. Control, IR-18.5h.tif]

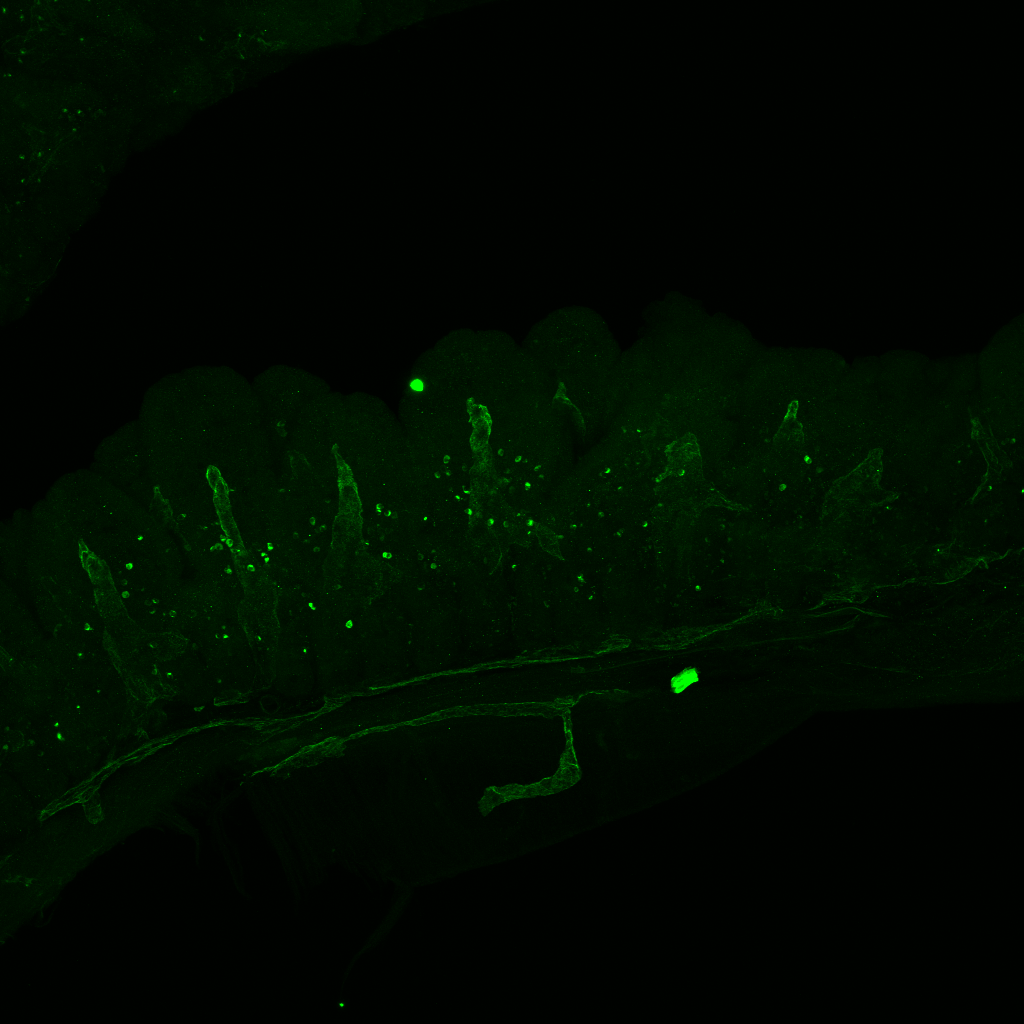

Supplement: Supplementary file 5 — Source Data for Expanded View and Appendix [file EMBR-24-e56030-s011.zip › Figure EV1-5, Appendix Figure S1-4/4. Figure EV4/Figure EV4B-WM LYVE1/4. EC-Foxc-DKO, IR-18.5h.tif]

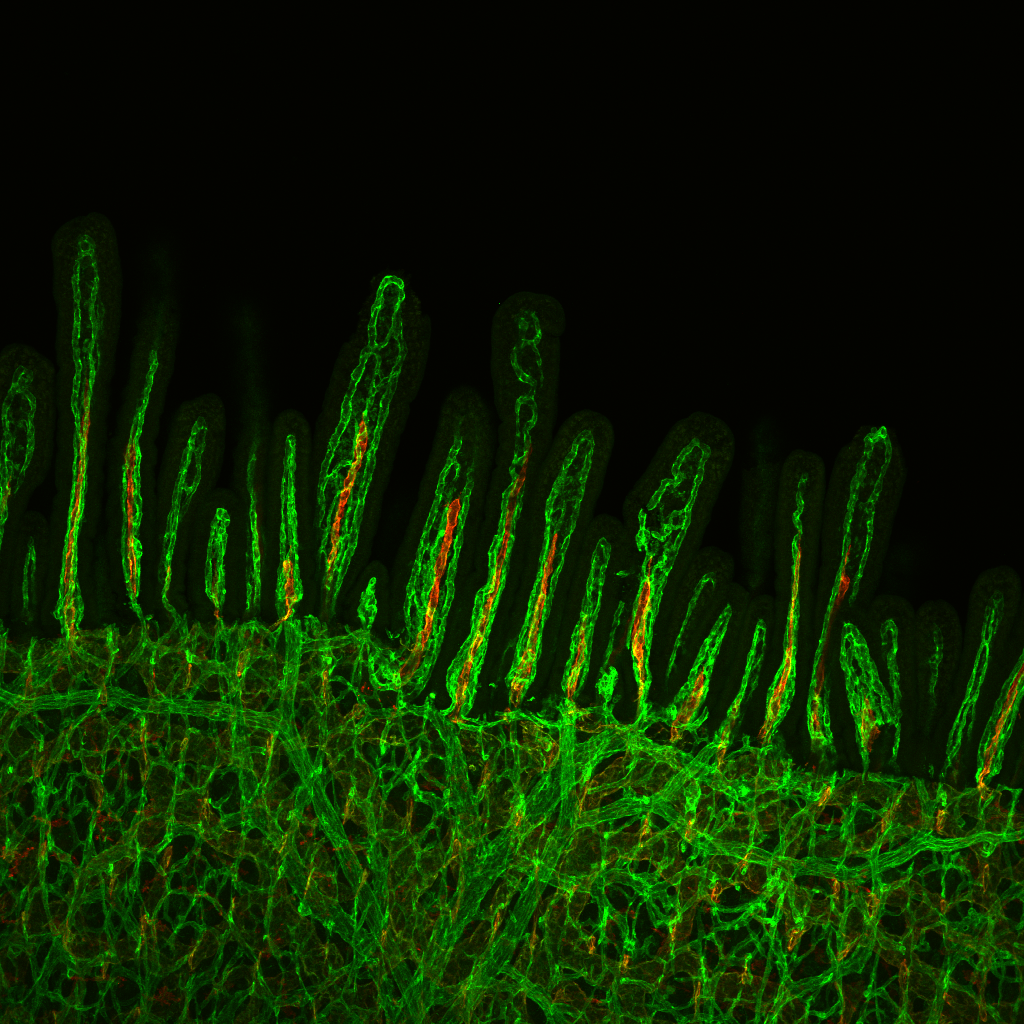

Supplement: Supplementary file 5 — Source Data for Expanded View and Appendix [file EMBR-24-e56030-s011.zip › Figure EV1-5, Appendix Figure S1-4/4. Figure EV4/Figurre EV4C-WM CD31 LYVE1 in neonatal intestine/1. Control.tif]

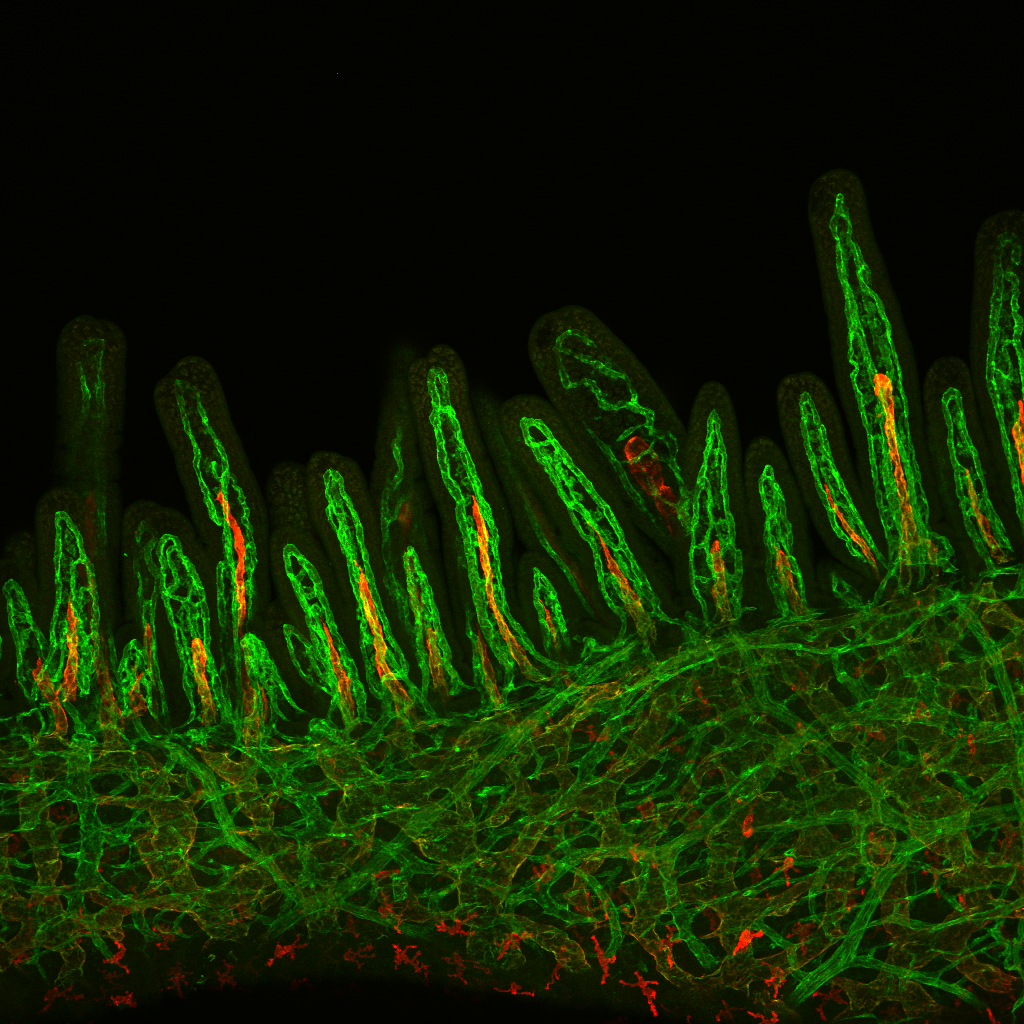

Supplement: Supplementary file 5 — Source Data for Expanded View and Appendix [file EMBR-24-e56030-s011.zip › Figure EV1-5, Appendix Figure S1-4/4. Figure EV4/Figurre EV4C-WM CD31 LYVE1 in neonatal intestine/2. EC-Foxc-DKO.tif]

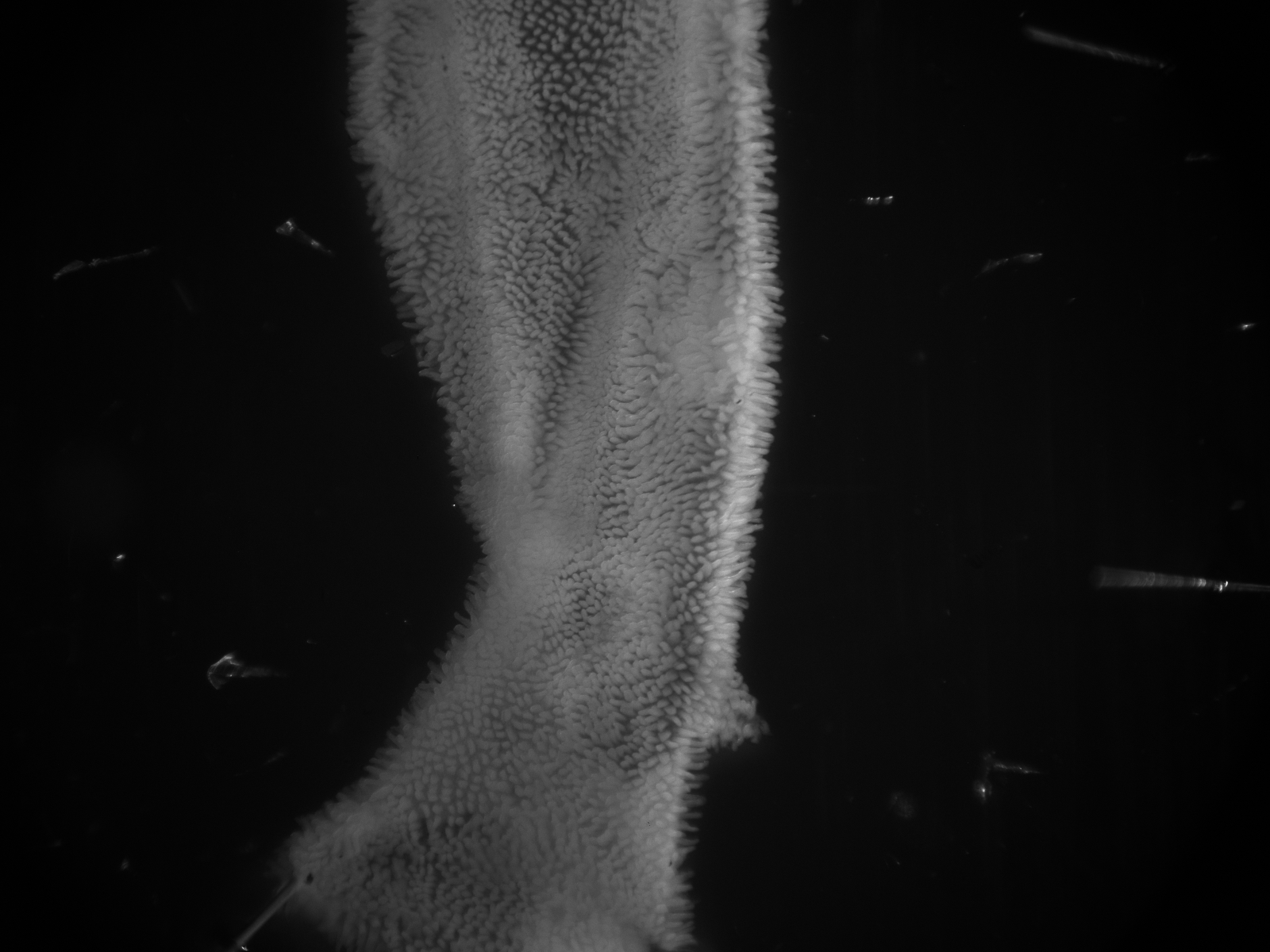

Supplement: Supplementary file 5 — Source Data for Expanded View and Appendix [file EMBR-24-e56030-s011.zip › Figure EV1-5, Appendix Figure S1-4/6. Appendix Figure S1/Appendix Figure S1A-BODIPY C16 in intestines/1-1. Vehicle control, bright field.tif]

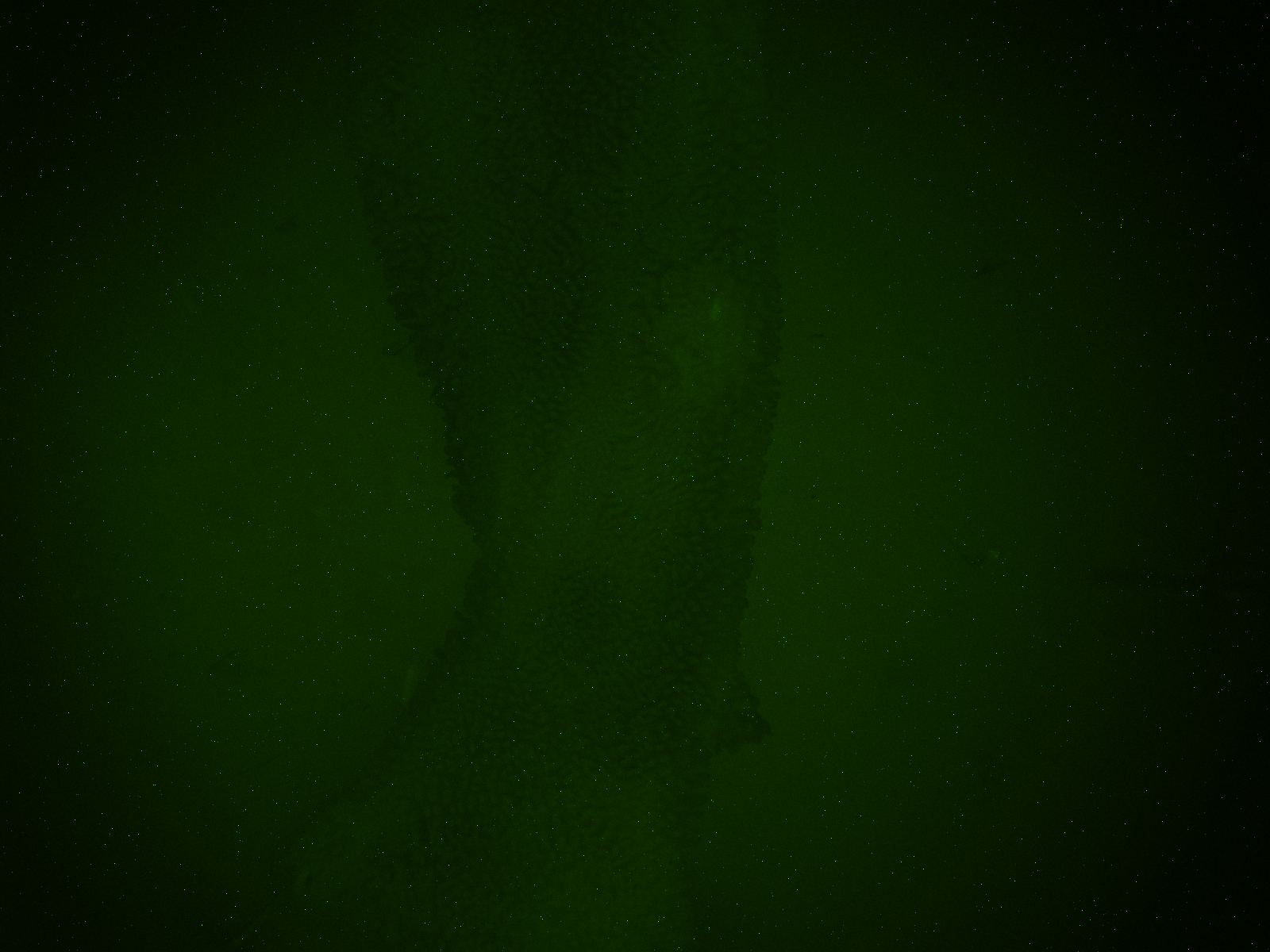

Supplement: Supplementary file 5 — Source Data for Expanded View and Appendix [file EMBR-24-e56030-s011.zip › Figure EV1-5, Appendix Figure S1-4/6. Appendix Figure S1/Appendix Figure S1A-BODIPY C16 in intestines/1-2. Vehicle control, BODIPY C16.tif]

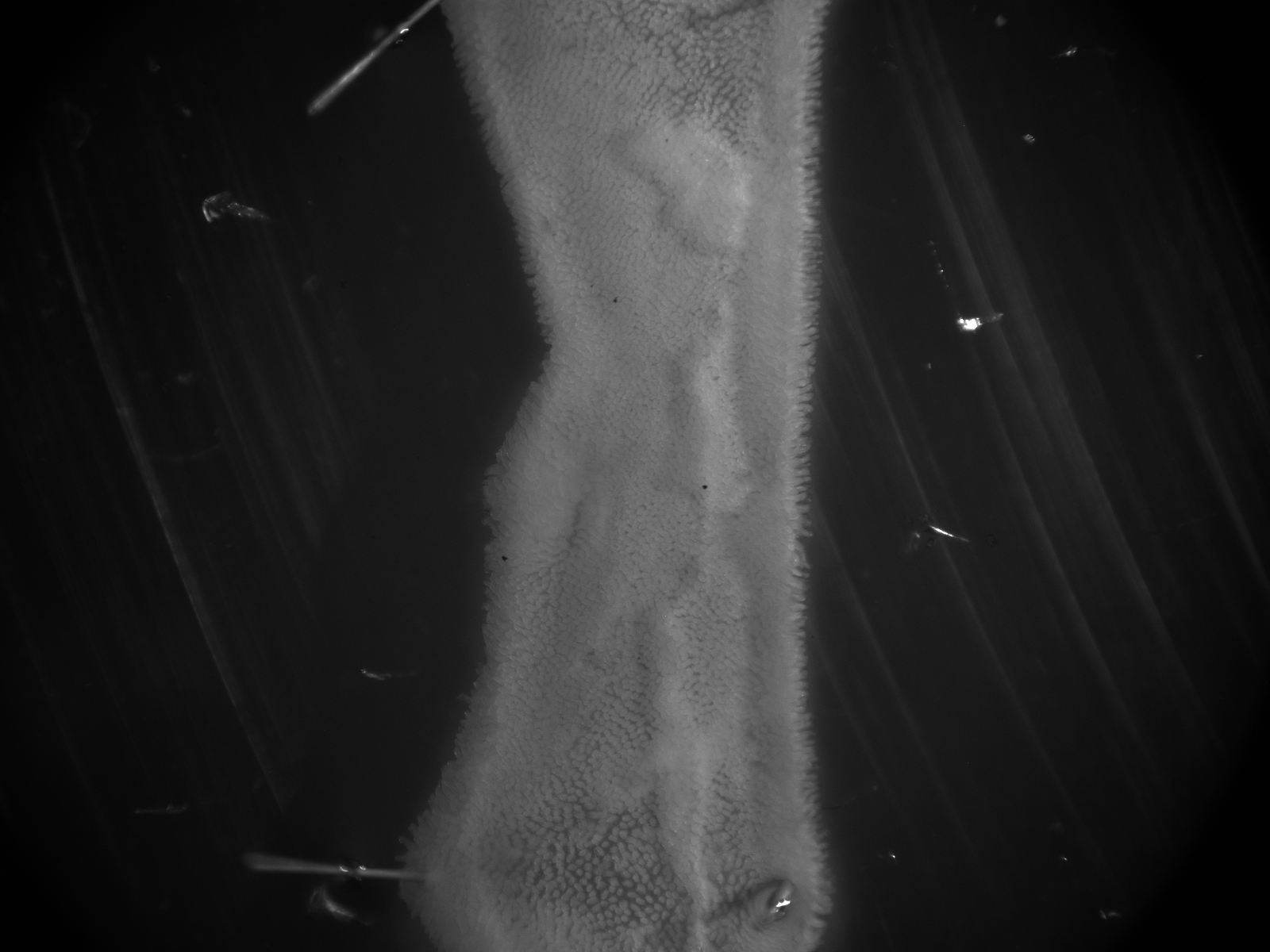

Supplement: Supplementary file 5 — Source Data for Expanded View and Appendix [file EMBR-24-e56030-s011.zip › Figure EV1-5, Appendix Figure S1-4/6. Appendix Figure S1/Appendix Figure S1A-BODIPY C16 in intestines/2-1. Control, bright field.tif]

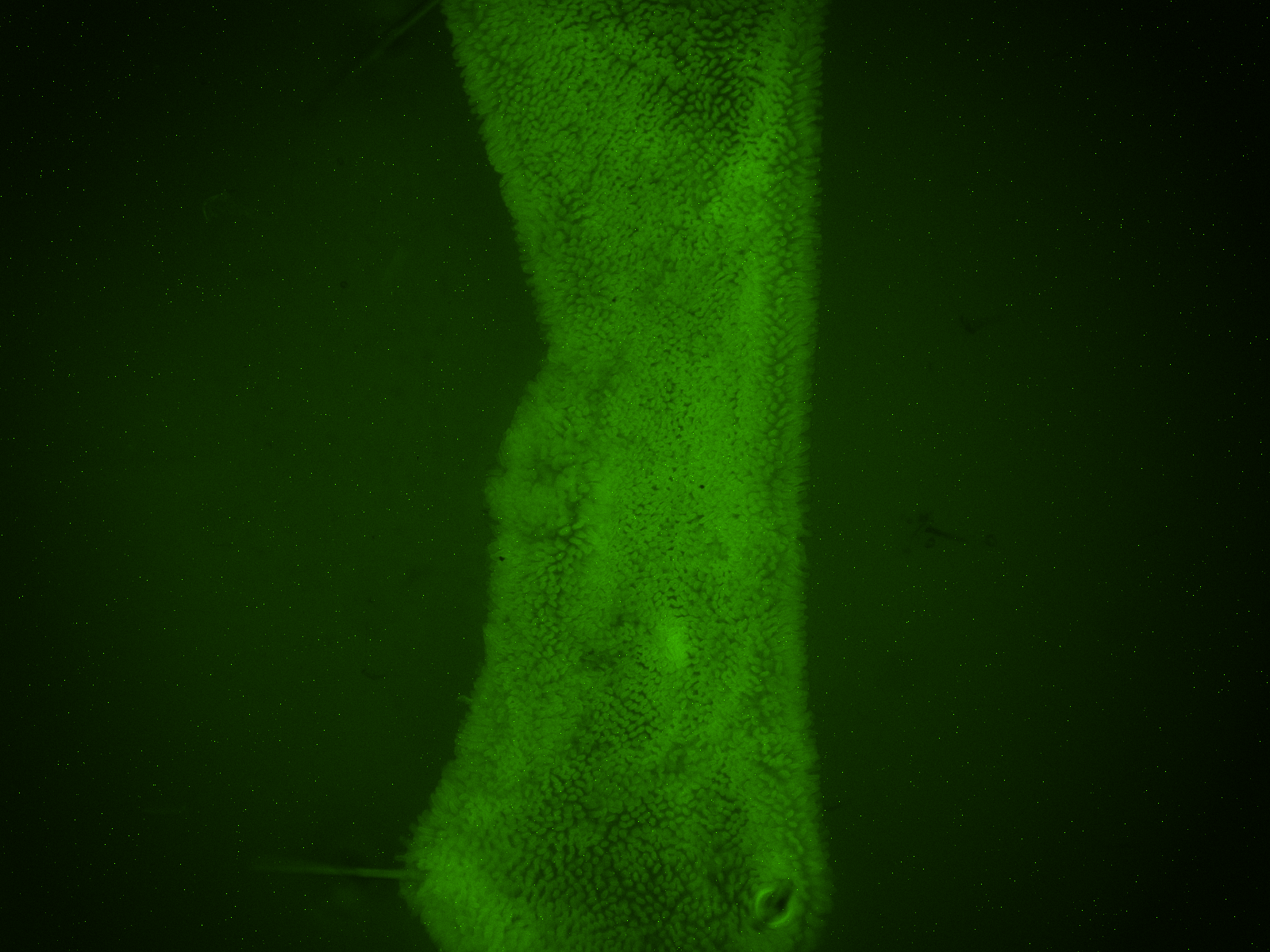

Supplement: Supplementary file 5 — Source Data for Expanded View and Appendix [file EMBR-24-e56030-s011.zip › Figure EV1-5, Appendix Figure S1-4/6. Appendix Figure S1/Appendix Figure S1A-BODIPY C16 in intestines/2-2. Control, BODIPY C16.tif]

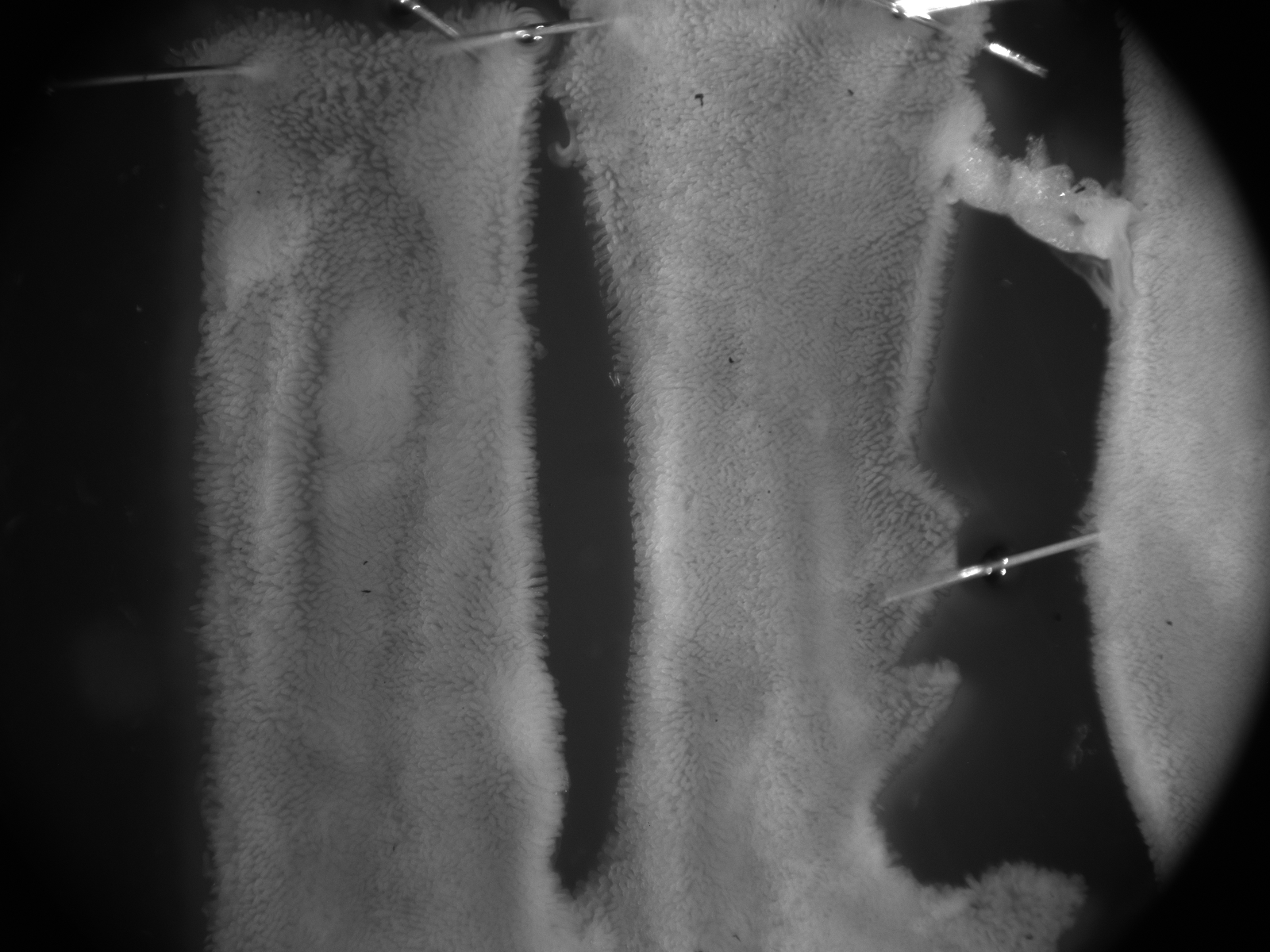

Supplement: Supplementary file 5 — Source Data for Expanded View and Appendix [file EMBR-24-e56030-s011.zip › Figure EV1-5, Appendix Figure S1-4/6. Appendix Figure S1/Appendix Figure S1A-BODIPY C16 in intestines/3-1. EC-Foxc-DKO, bright field.tif]

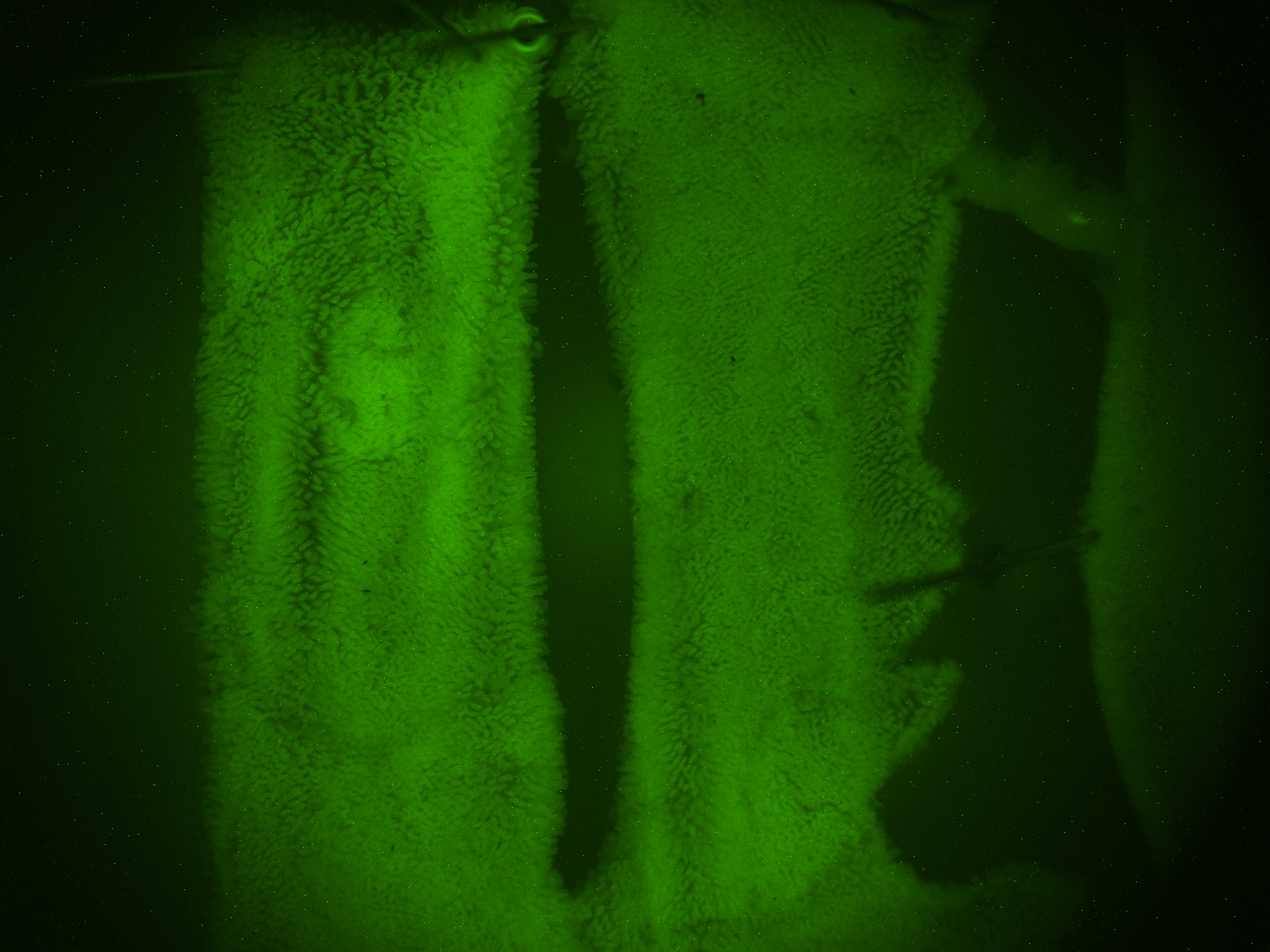

Supplement: Supplementary file 5 — Source Data for Expanded View and Appendix [file EMBR-24-e56030-s011.zip › Figure EV1-5, Appendix Figure S1-4/6. Appendix Figure S1/Appendix Figure S1A-BODIPY C16 in intestines/3-2. EC-Foxc-DKO, BODIPY C16.tif]

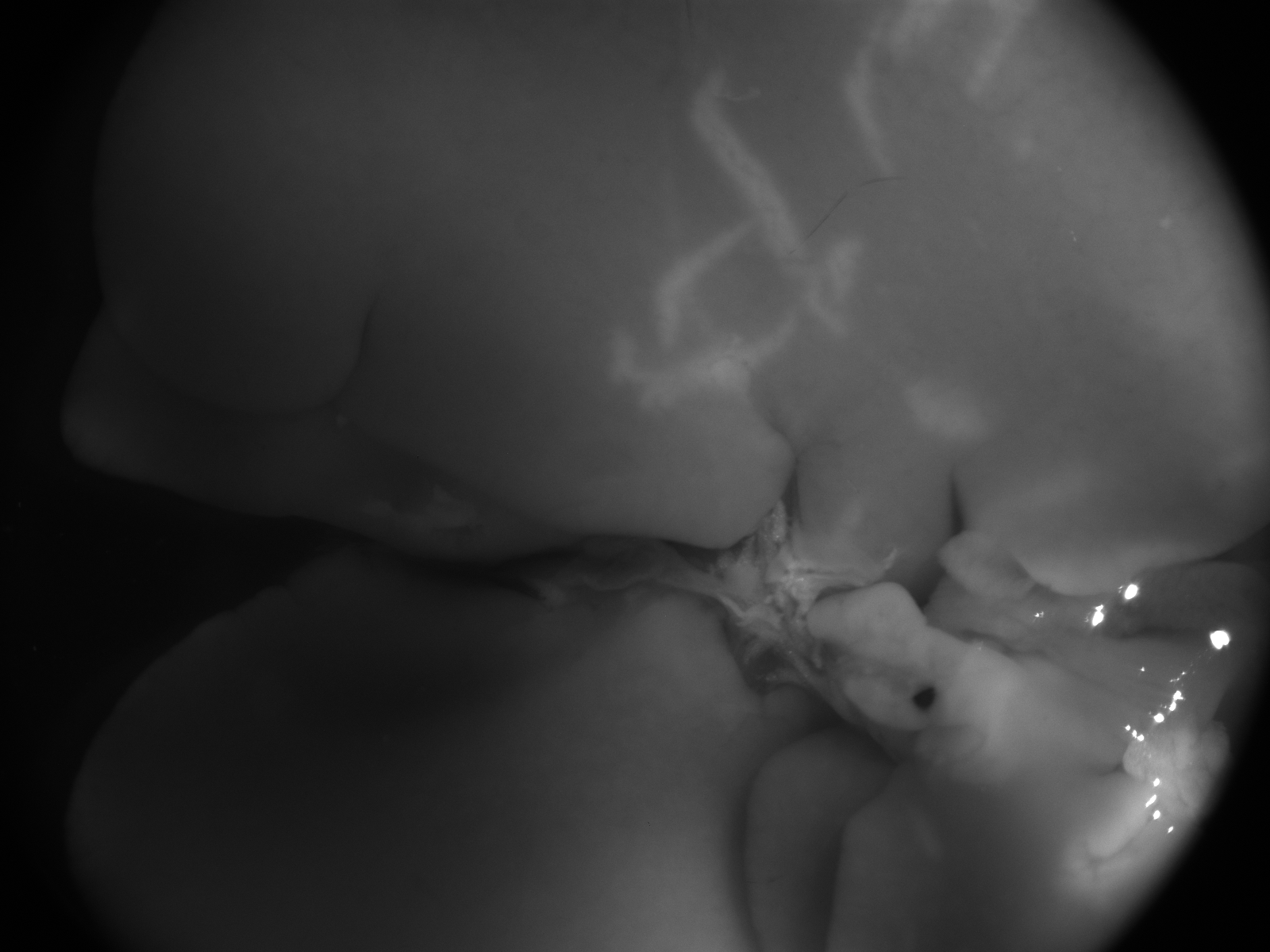

Supplement: Supplementary file 5 — Source Data for Expanded View and Appendix [file EMBR-24-e56030-s011.zip › Figure EV1-5, Appendix Figure S1-4/6. Appendix Figure S1/Appendix Figure S1C-BODIPY C16 in livers/1-1. Vehicle control, bright field.tif]

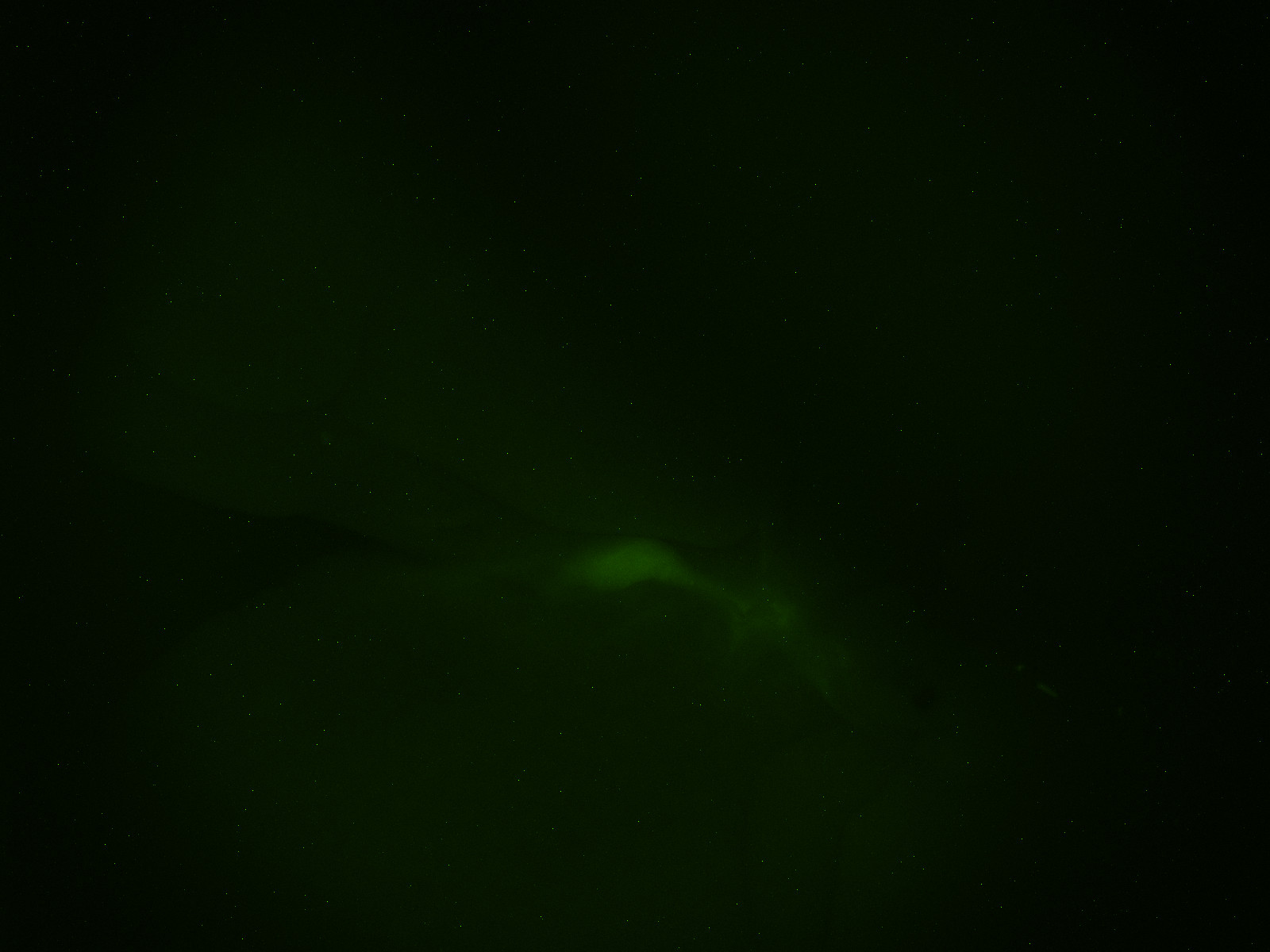

Supplement: Supplementary file 5 — Source Data for Expanded View and Appendix [file EMBR-24-e56030-s011.zip › Figure EV1-5, Appendix Figure S1-4/6. Appendix Figure S1/Appendix Figure S1C-BODIPY C16 in livers/1-2. Vehicle control, BODIPY C16.tif]

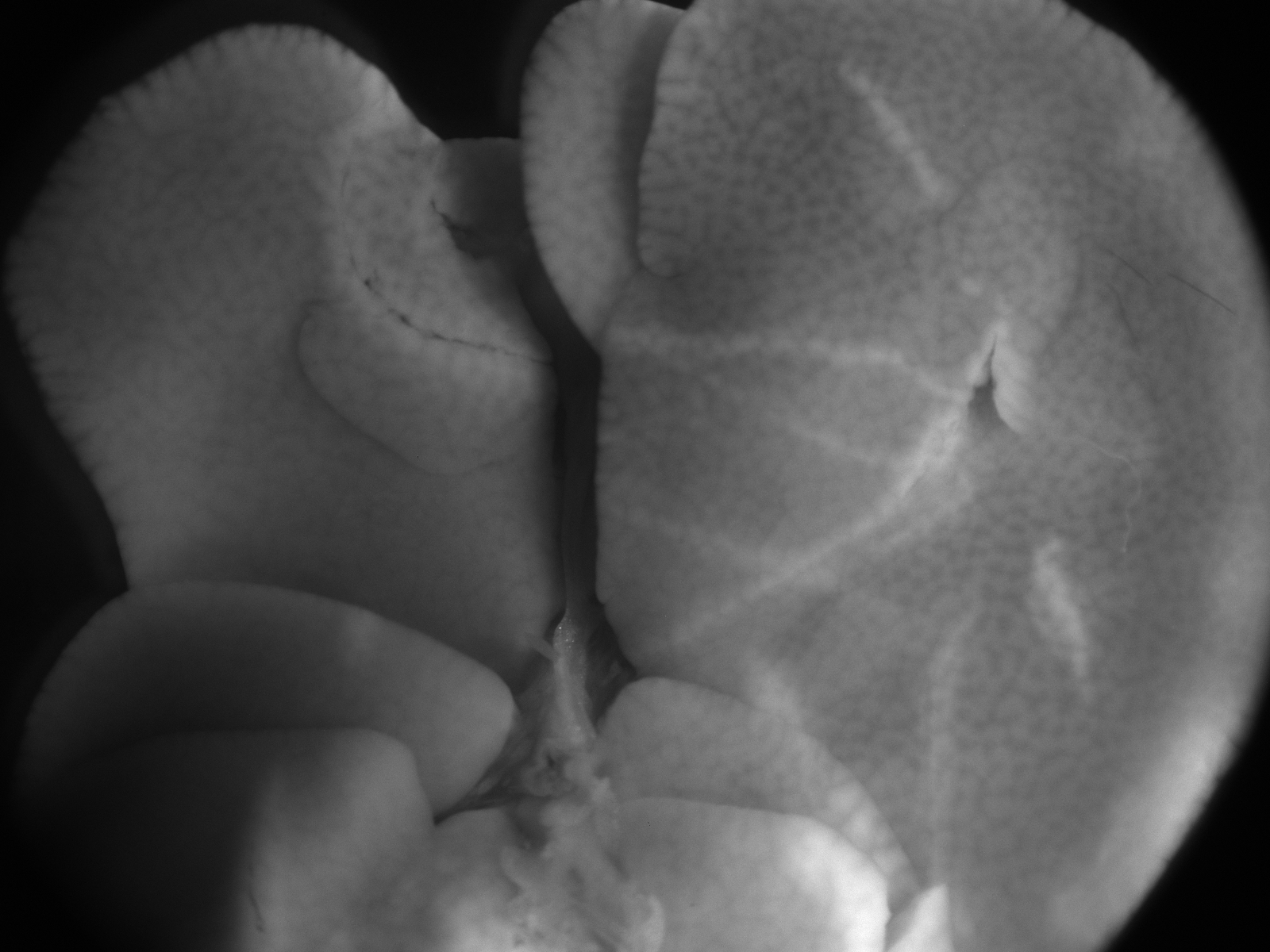

Supplement: Supplementary file 5 — Source Data for Expanded View and Appendix [file EMBR-24-e56030-s011.zip › Figure EV1-5, Appendix Figure S1-4/6. Appendix Figure S1/Appendix Figure S1C-BODIPY C16 in livers/2-1. Control, bright field.tif]

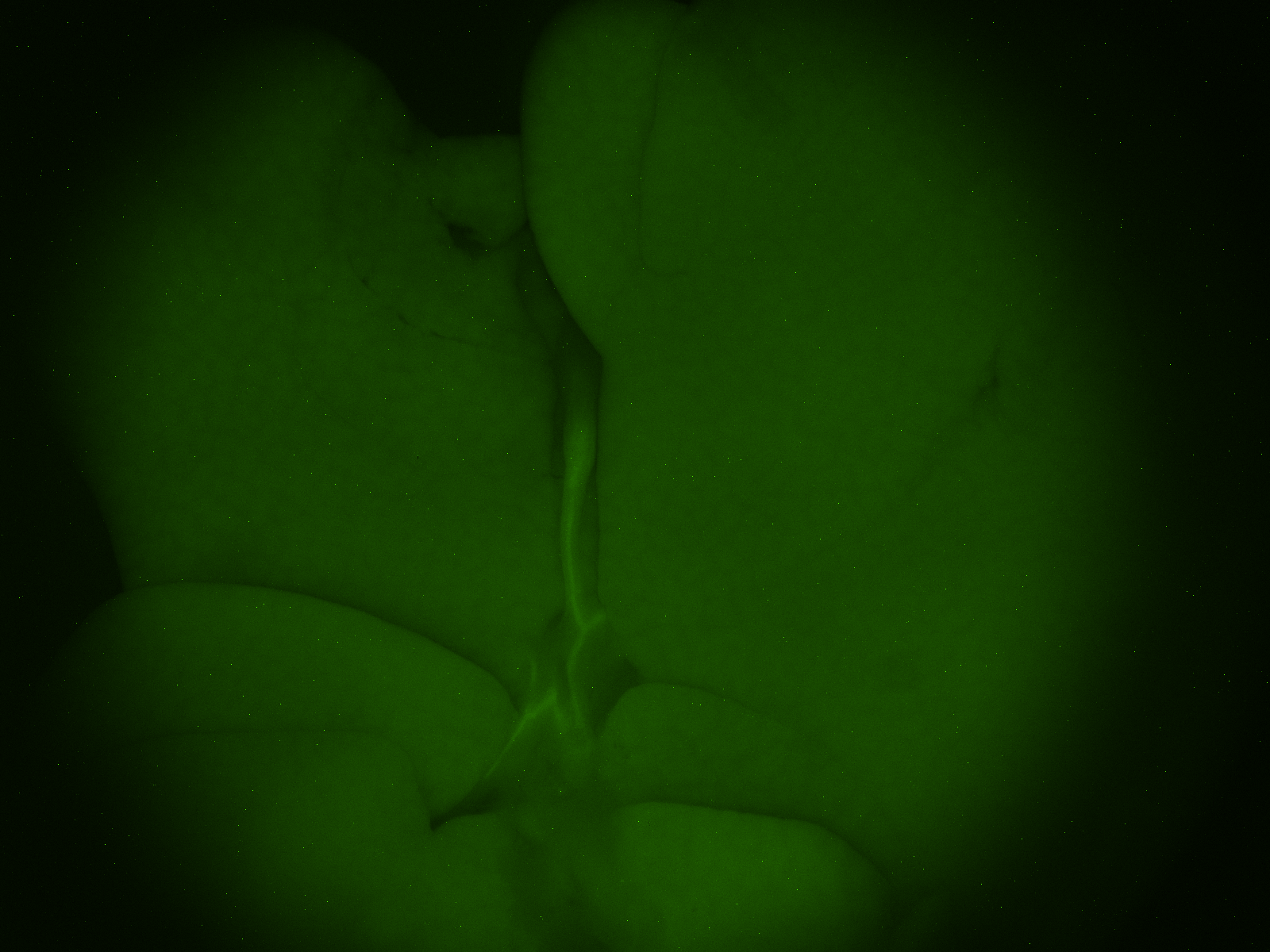

Supplement: Supplementary file 5 — Source Data for Expanded View and Appendix [file EMBR-24-e56030-s011.zip › Figure EV1-5, Appendix Figure S1-4/6. Appendix Figure S1/Appendix Figure S1C-BODIPY C16 in livers/2-2. Control, BODIPY C16.tif]

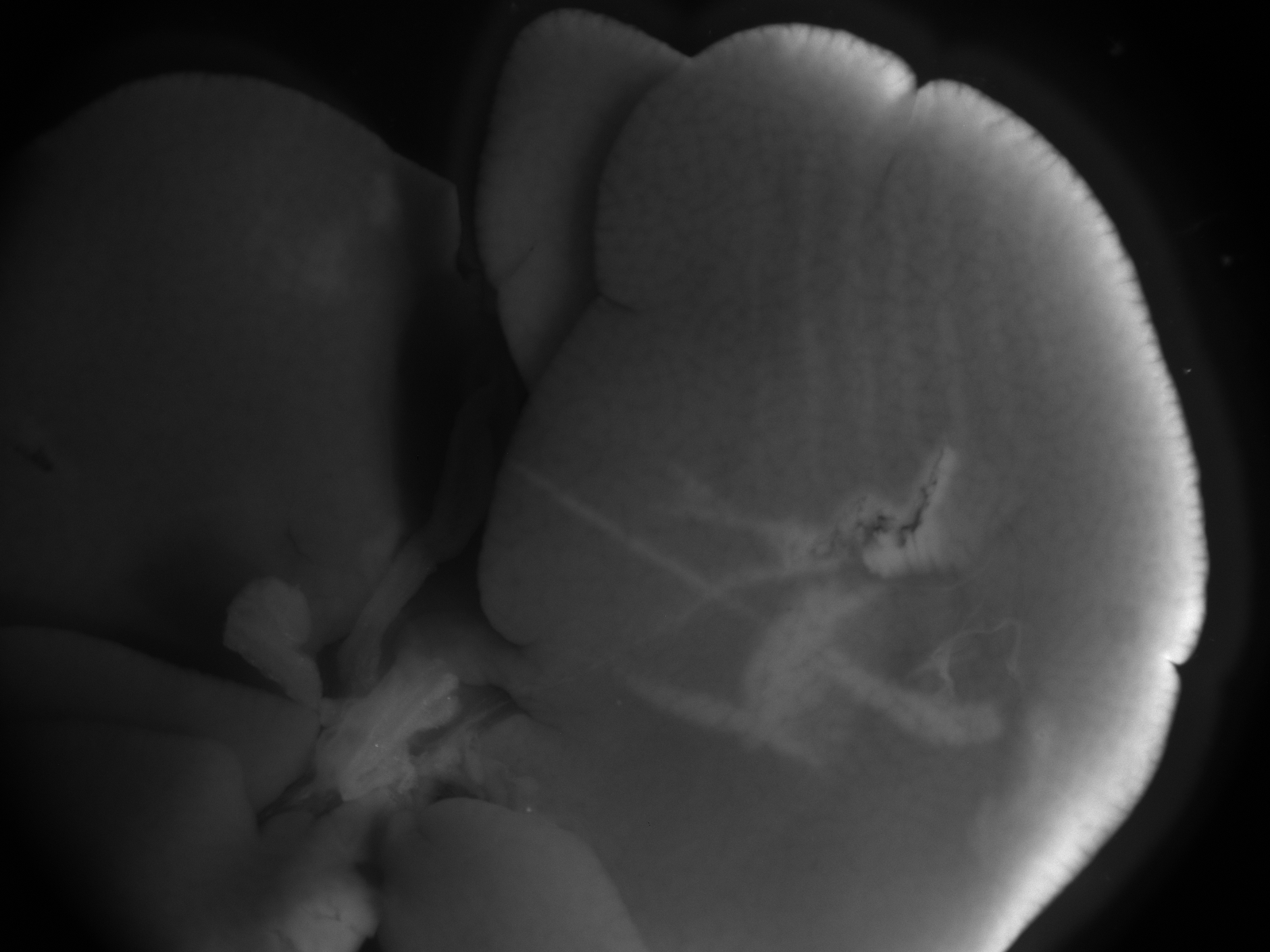

Supplement: Supplementary file 5 — Source Data for Expanded View and Appendix [file EMBR-24-e56030-s011.zip › Figure EV1-5, Appendix Figure S1-4/6. Appendix Figure S1/Appendix Figure S1C-BODIPY C16 in livers/3-1. EC-Foxc-DKO, bright field.tif]

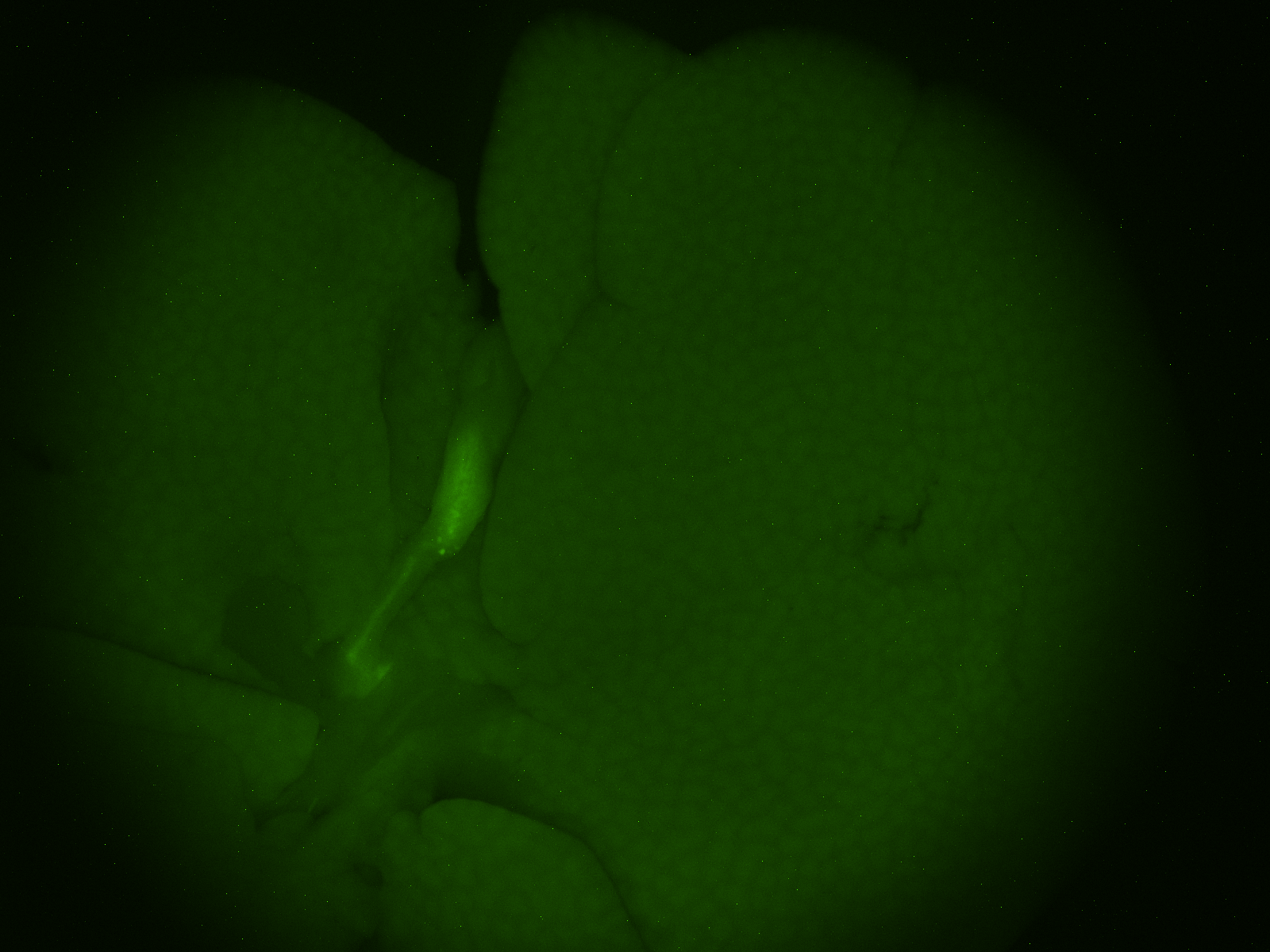

Supplement: Supplementary file 5 — Source Data for Expanded View and Appendix [file EMBR-24-e56030-s011.zip › Figure EV1-5, Appendix Figure S1-4/6. Appendix Figure S1/Appendix Figure S1C-BODIPY C16 in livers/3-2. EC-Foxc-DKO, BODIPY C16.tif]

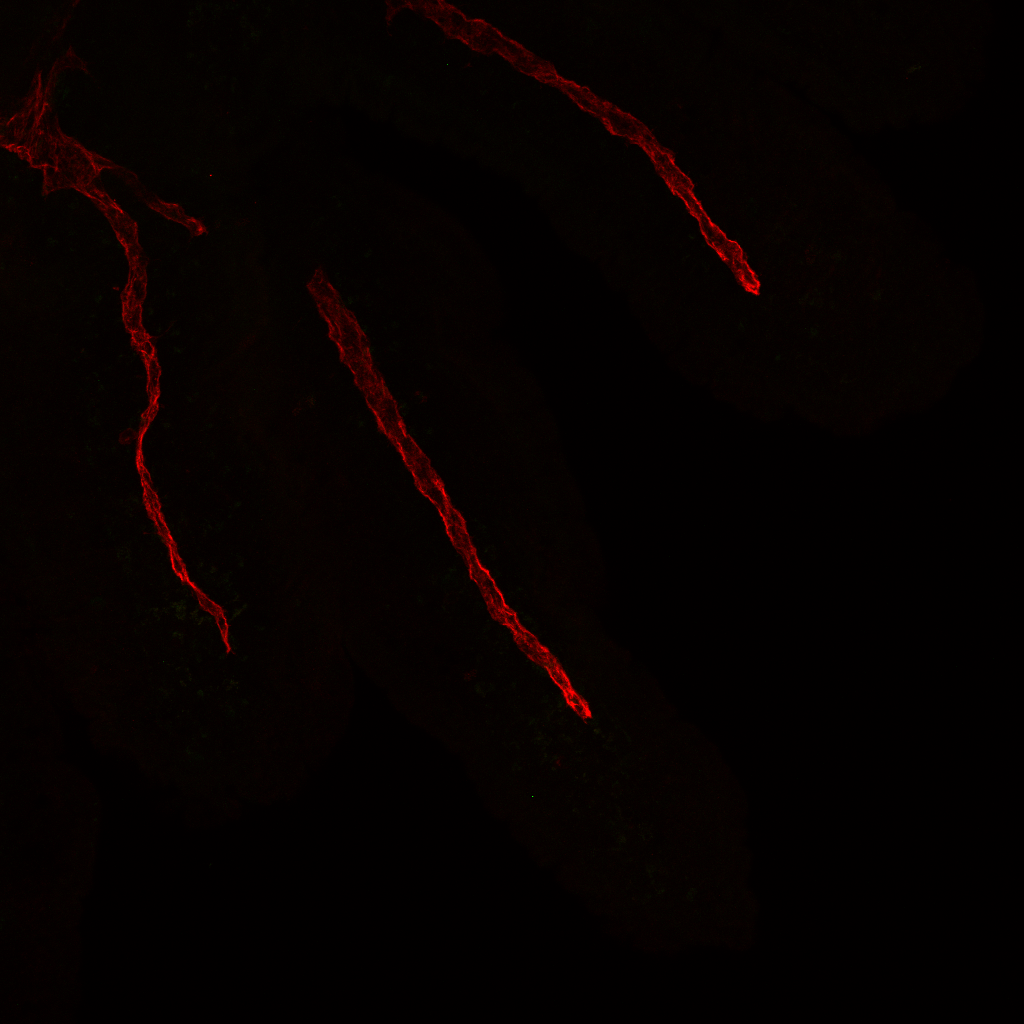

Supplement: Supplementary file 5 — Source Data for Expanded View and Appendix [file EMBR-24-e56030-s011.zip › Figure EV1-5, Appendix Figure S1-4/6. Appendix Figure S1/Appendix Figure S1F-WM-BODIPY C16 and LYVE1/1. Control -.tif]

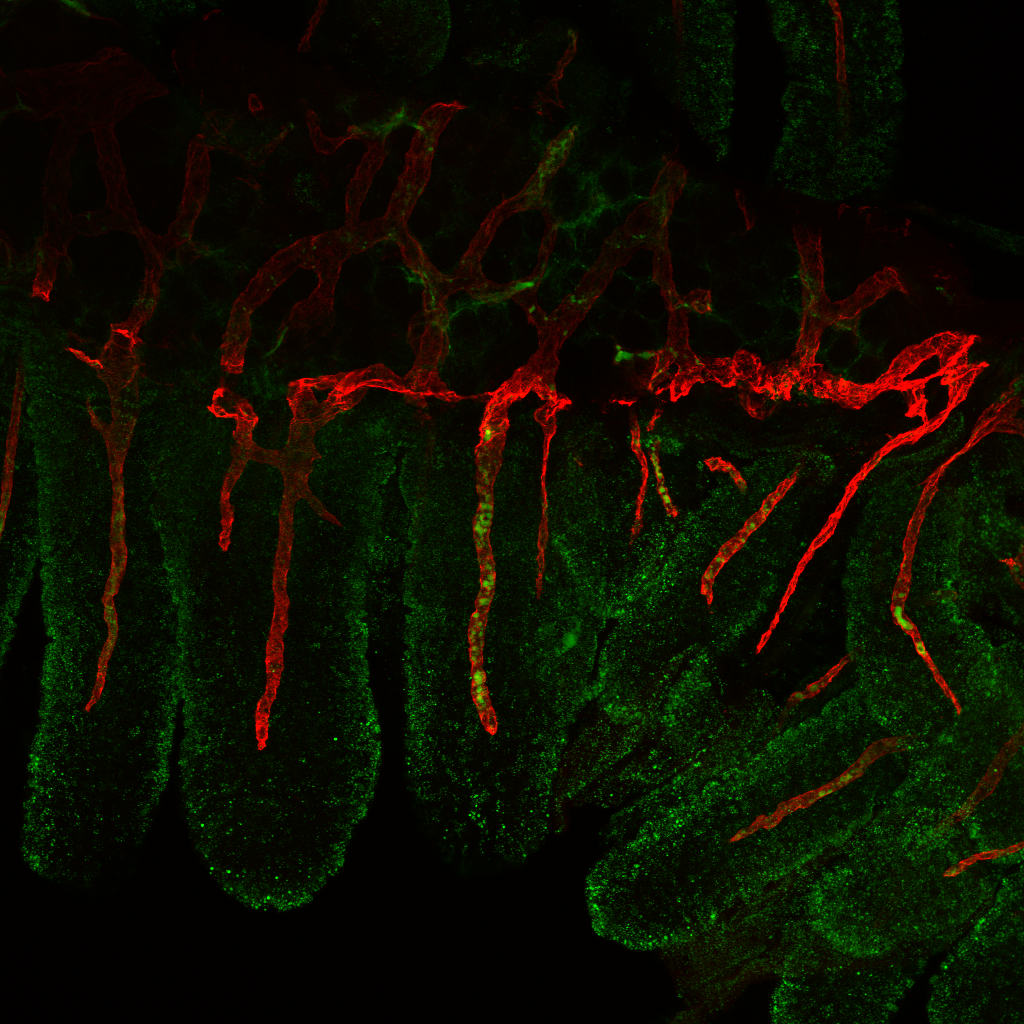

Supplement: Supplementary file 5 — Source Data for Expanded View and Appendix [file EMBR-24-e56030-s011.zip › Figure EV1-5, Appendix Figure S1-4/6. Appendix Figure S1/Appendix Figure S1F-WM-BODIPY C16 and LYVE1/2. Control +.tif]

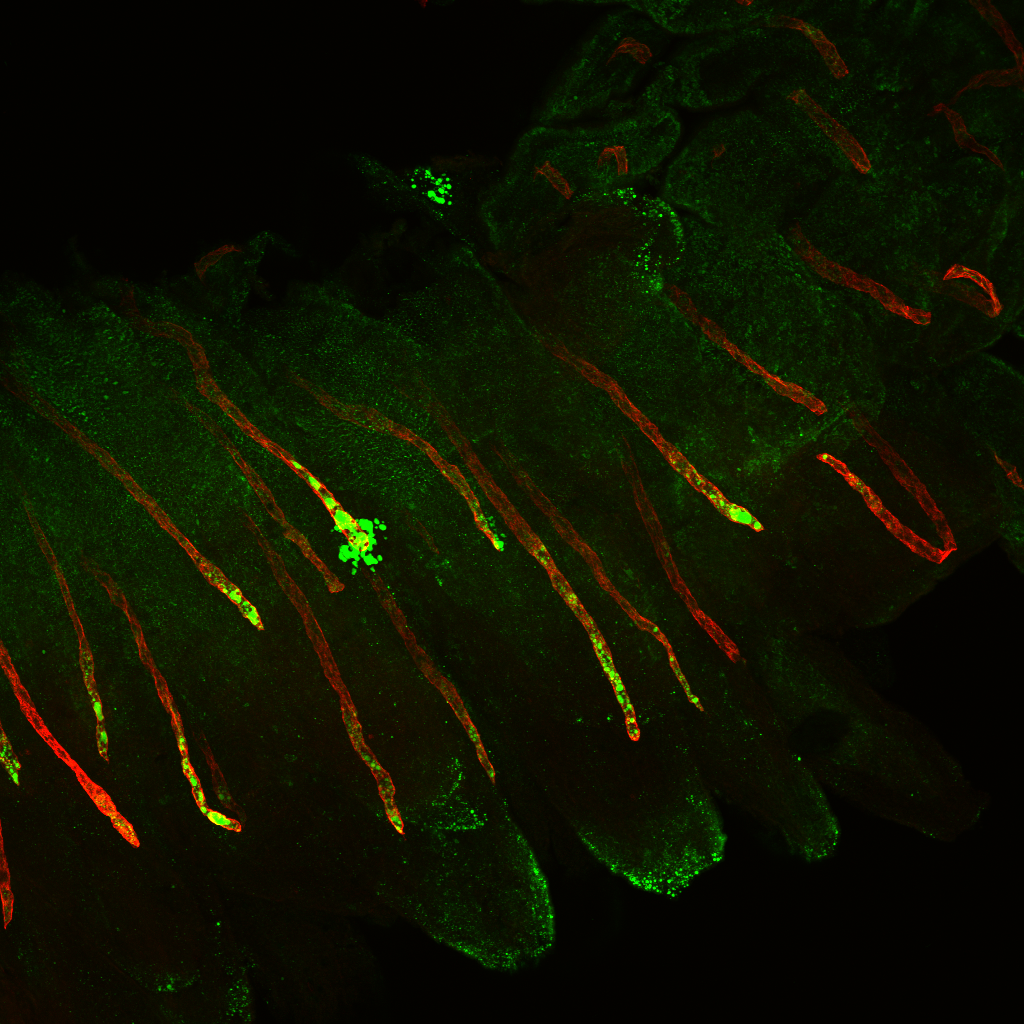

Supplement: Supplementary file 5 — Source Data for Expanded View and Appendix [file EMBR-24-e56030-s011.zip › Figure EV1-5, Appendix Figure S1-4/6. Appendix Figure S1/Appendix Figure S1F-WM-BODIPY C16 and LYVE1/3. EC-Foxc-DKO.tif]

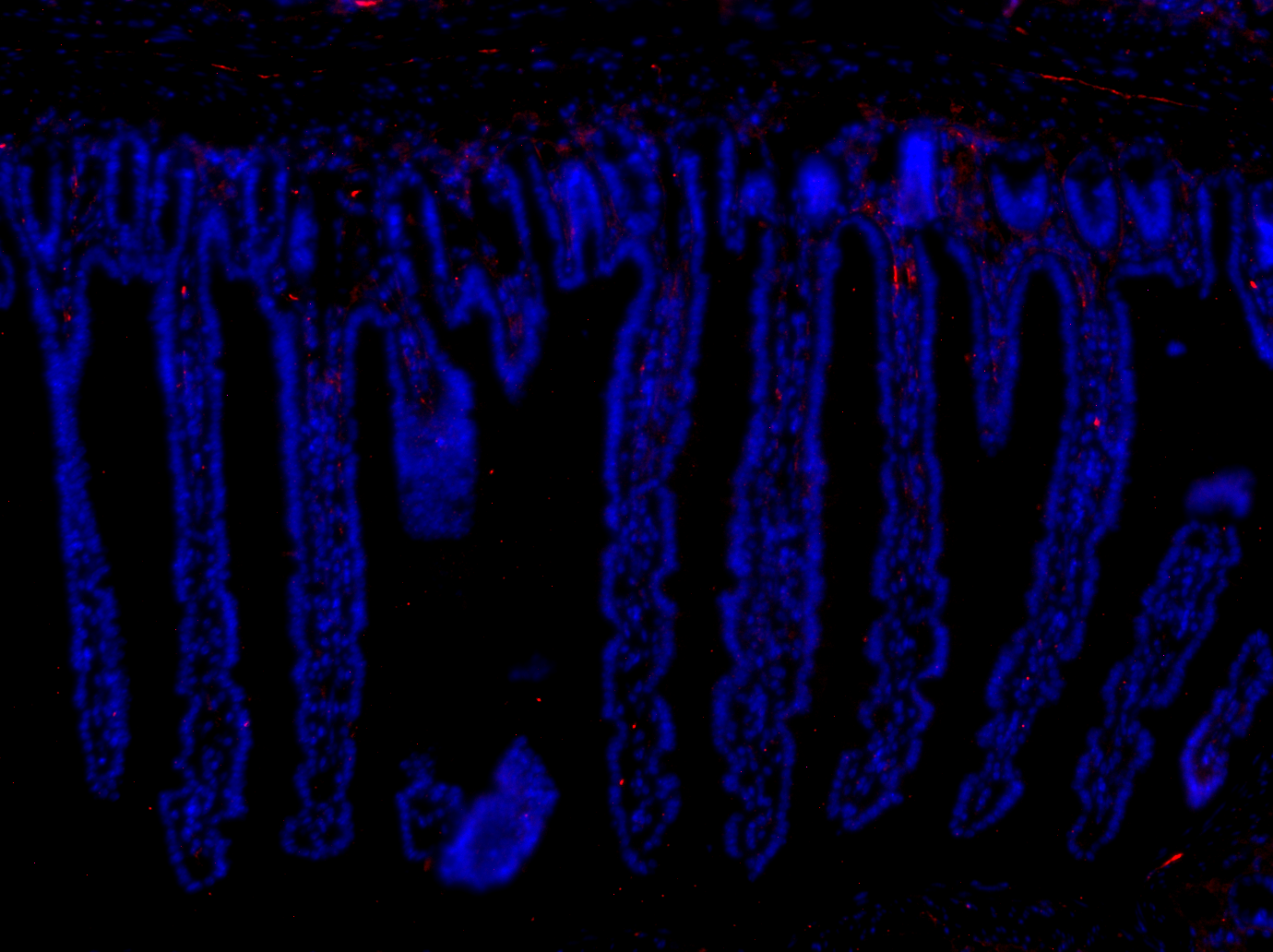

Supplement: Supplementary file 5 — Source Data for Expanded View and Appendix [file EMBR-24-e56030-s011.zip › Figure EV1-5, Appendix Figure S1-4/8. Appendix Figure S3/Appendix Figure S3A-IgM IHC/1. Control, sham.tif]

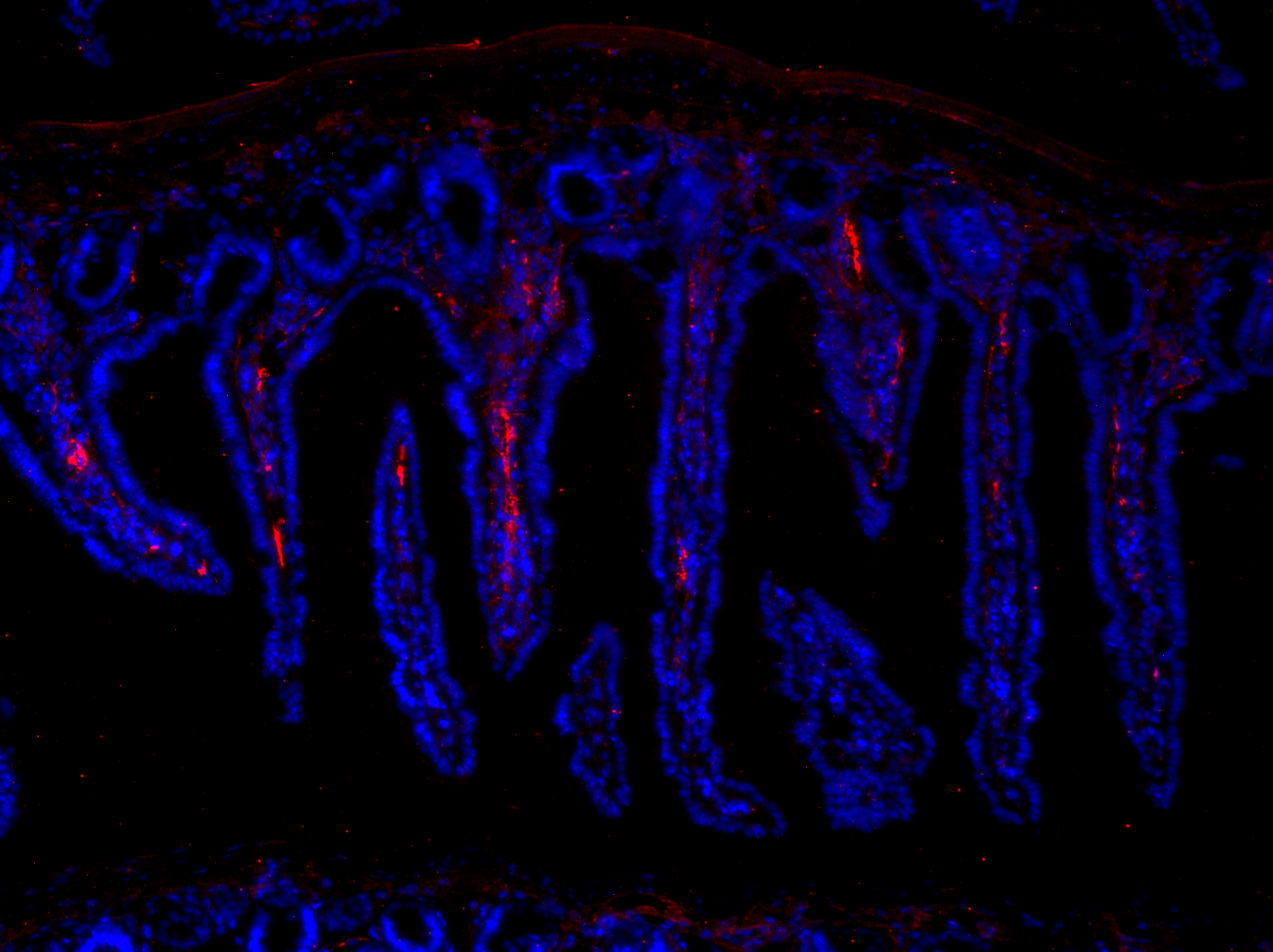

Supplement: Supplementary file 5 — Source Data for Expanded View and Appendix [file EMBR-24-e56030-s011.zip › Figure EV1-5, Appendix Figure S1-4/8. Appendix Figure S3/Appendix Figure S3A-IgM IHC/2. Control, IR.tif]

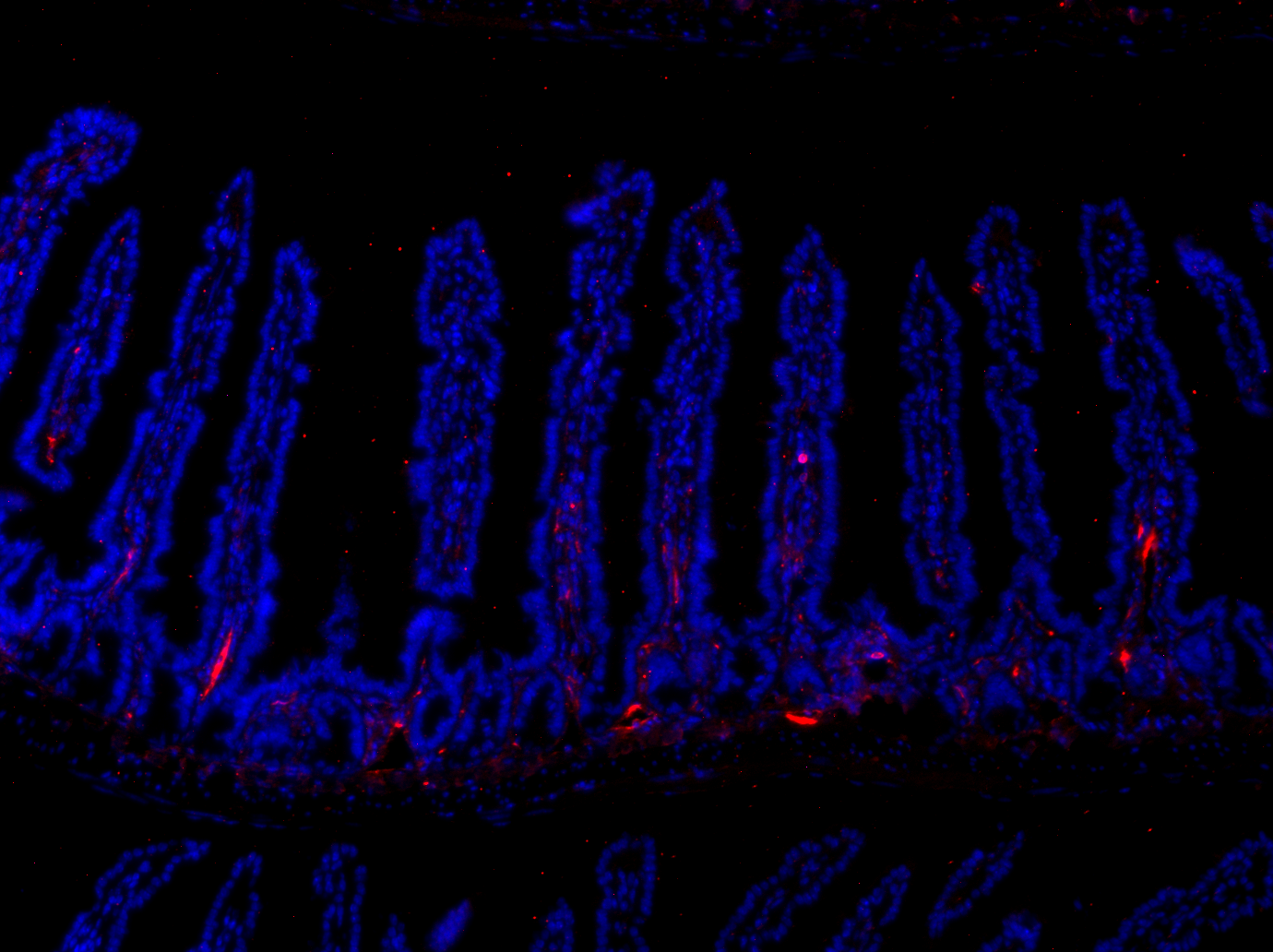

Supplement: Supplementary file 5 — Source Data for Expanded View and Appendix [file EMBR-24-e56030-s011.zip › Figure EV1-5, Appendix Figure S1-4/8. Appendix Figure S3/Appendix Figure S3A-IgM IHC/3. EC-Foxc-DKO, sham.tif]

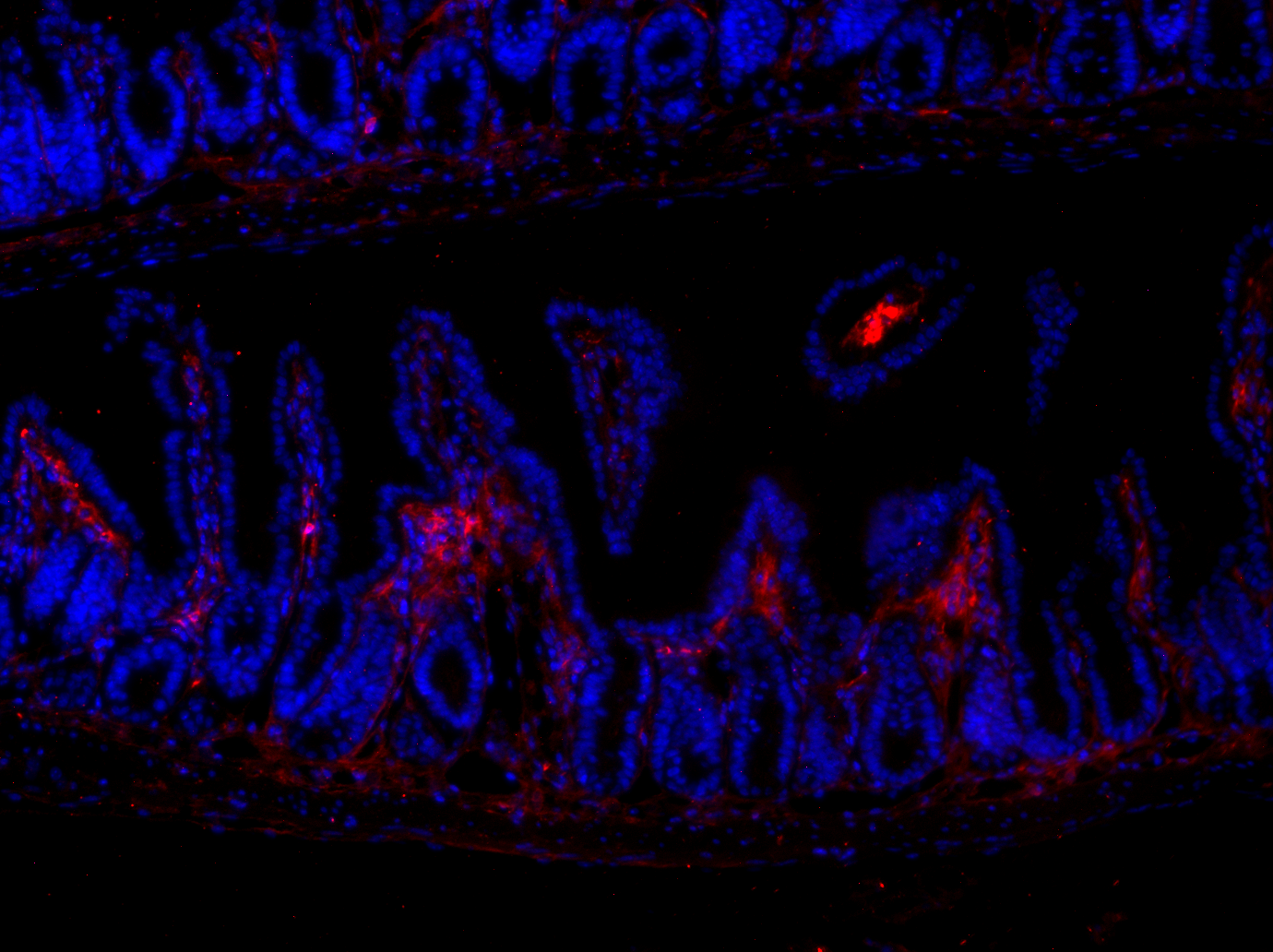

Supplement: Supplementary file 5 — Source Data for Expanded View and Appendix [file EMBR-24-e56030-s011.zip › Figure EV1-5, Appendix Figure S1-4/8. Appendix Figure S3/Appendix Figure S3A-IgM IHC/4. EC-Foxc-DKO, PBS-IR.tif]

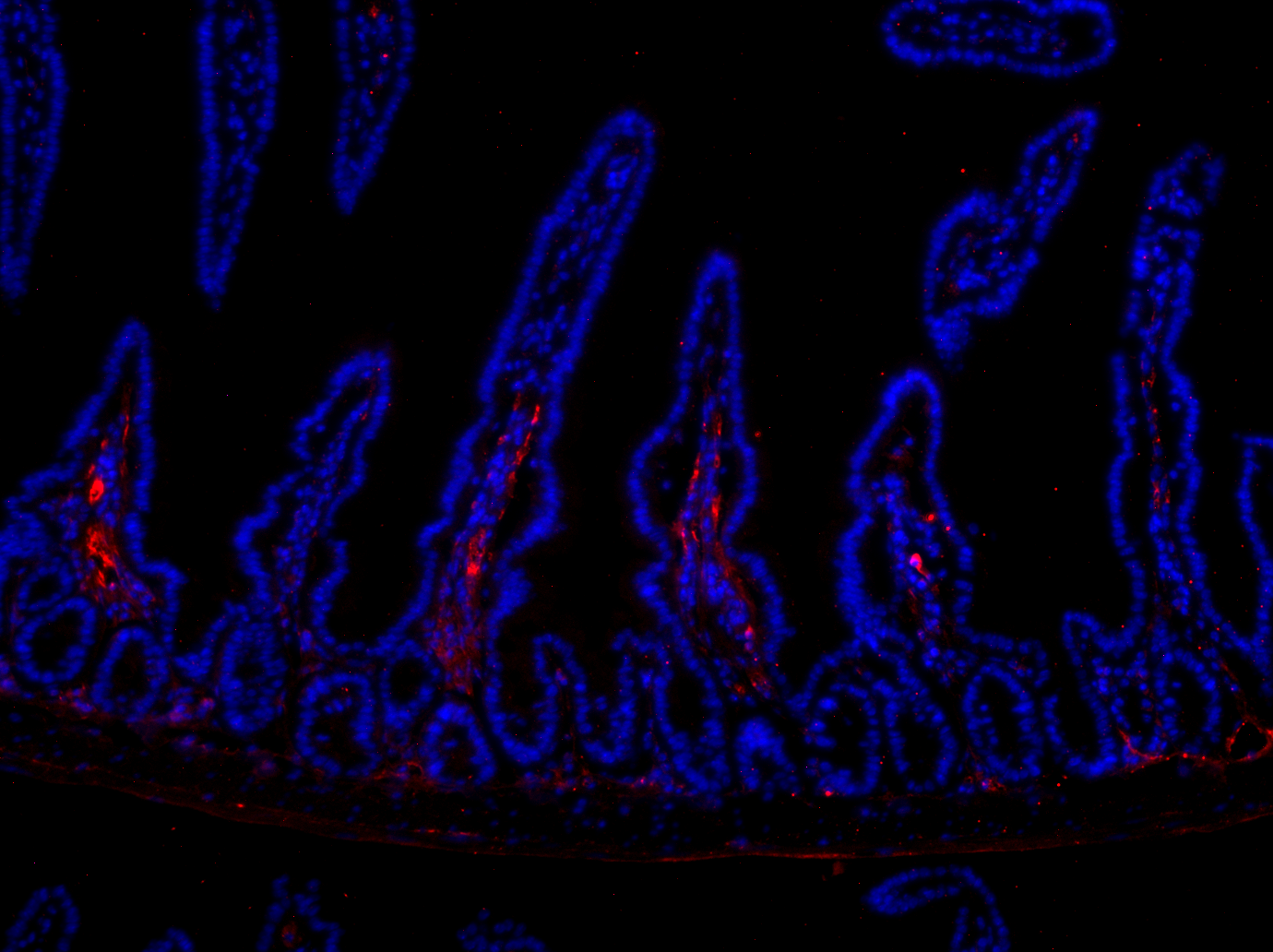

Supplement: Supplementary file 5 — Source Data for Expanded View and Appendix [file EMBR-24-e56030-s011.zip › Figure EV1-5, Appendix Figure S1-4/8. Appendix Figure S3/Appendix Figure S3A-IgM IHC/5. EC-Foxc-DKO, RSPO3-IR.tif]

**Full unedited gel for Appendix Figure S3B**

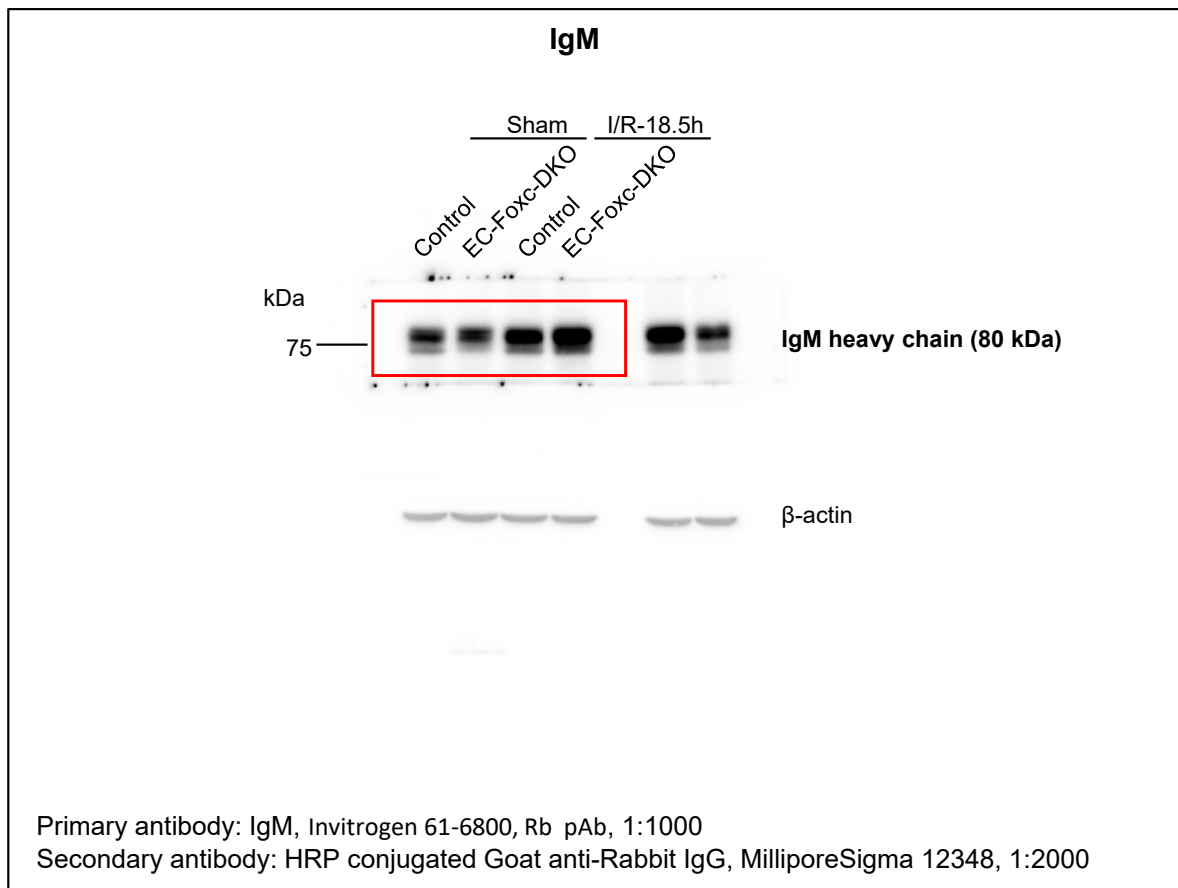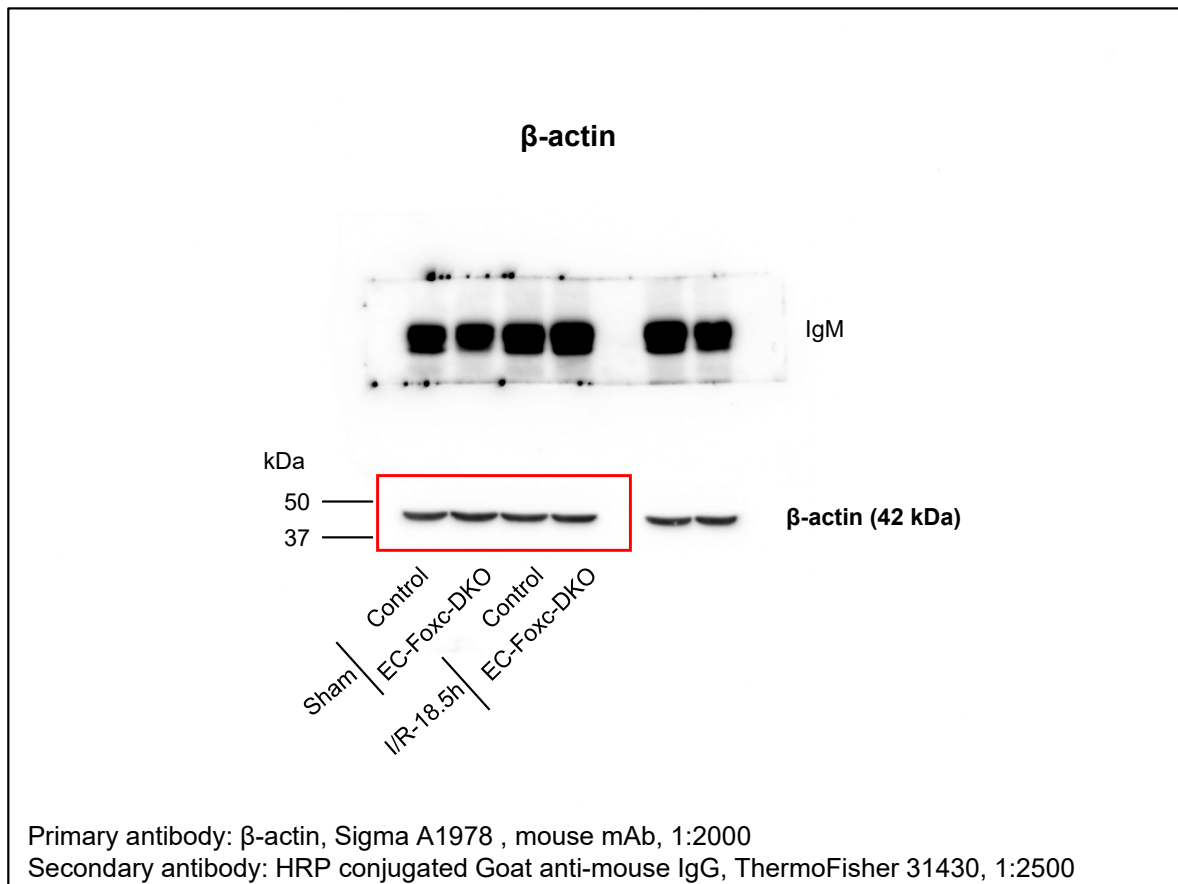

## Full unedited gel for Appendix Figure S3D

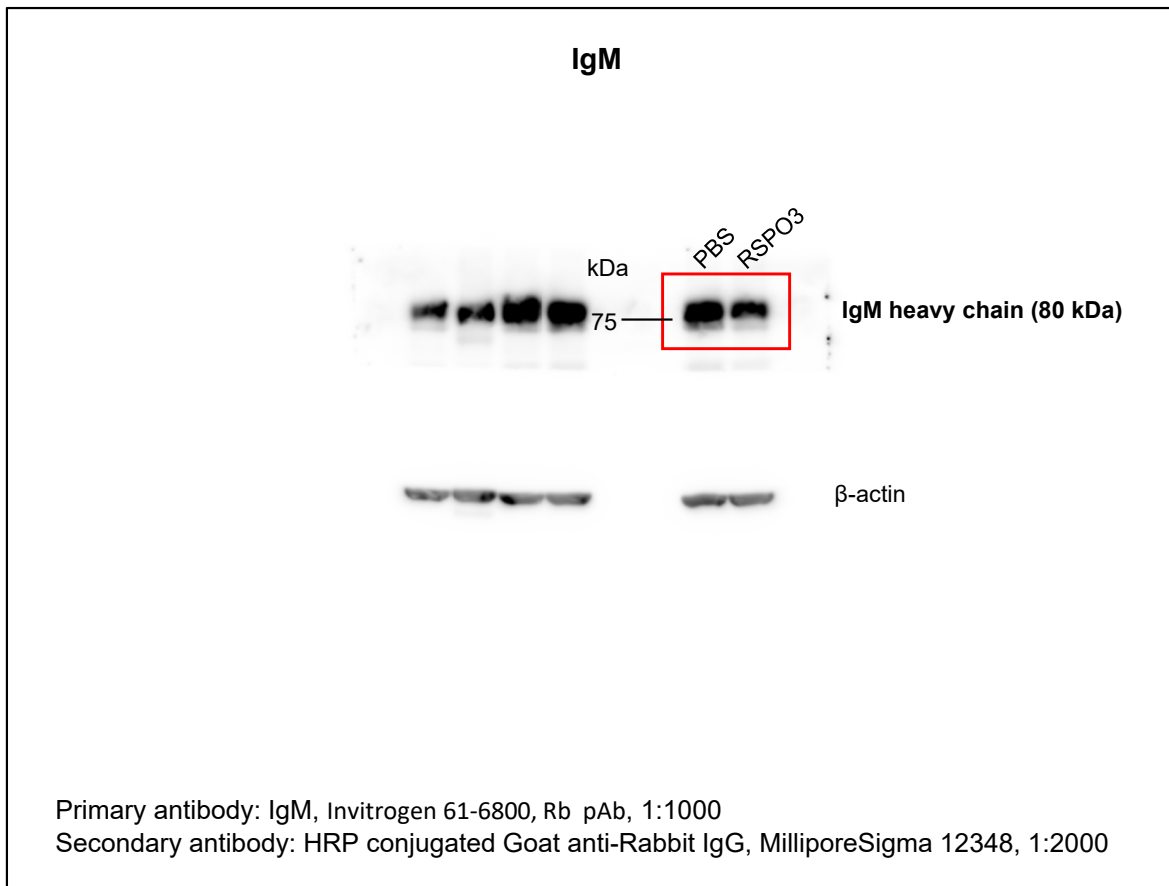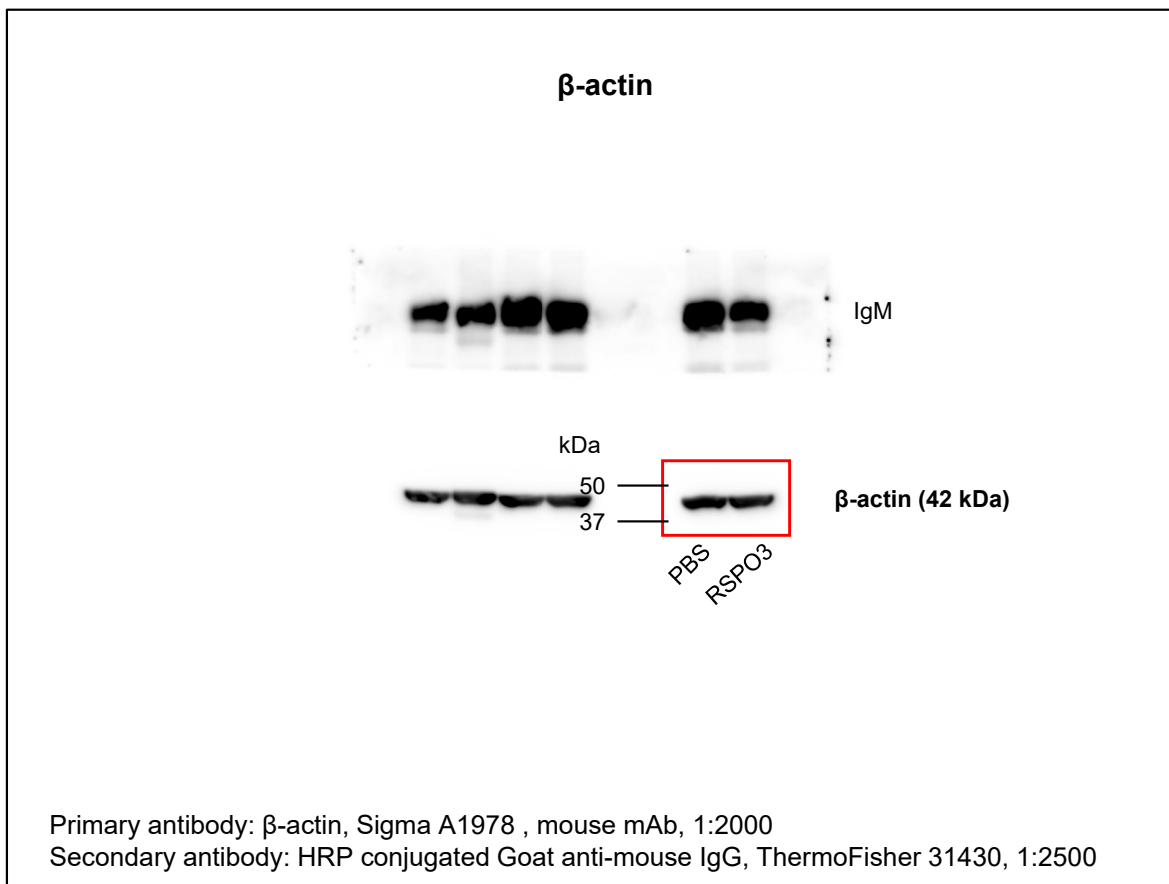

Supplement: Supplementary file 5 — Source Data for Expanded View and Appendix [file EMBR-24-e56030-s011.zip › Figure EV1-5, Appendix Figure S1-4/8. Appendix Figure S3/Appendix Figure S3B, S3D-WB uncut gel file.pdf]

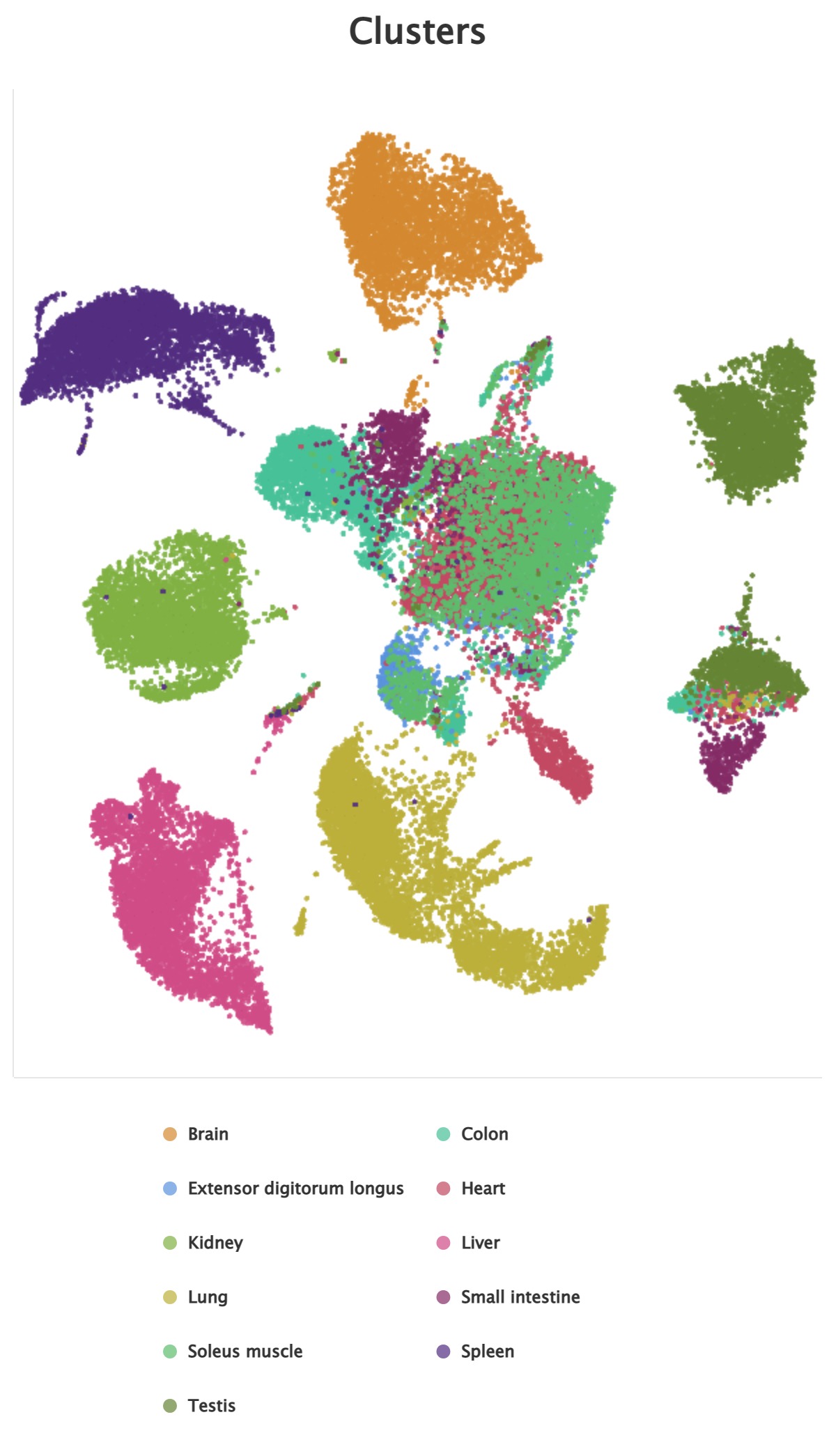

Supplement: Supplementary file 5 — Source Data for Expanded View and Appendix [file EMBR-24-e56030-s011.zip › Figure EV1-5, Appendix Figure S1-4/9. Appendix Figure S4/Appendix Figure S4A.jpeg]

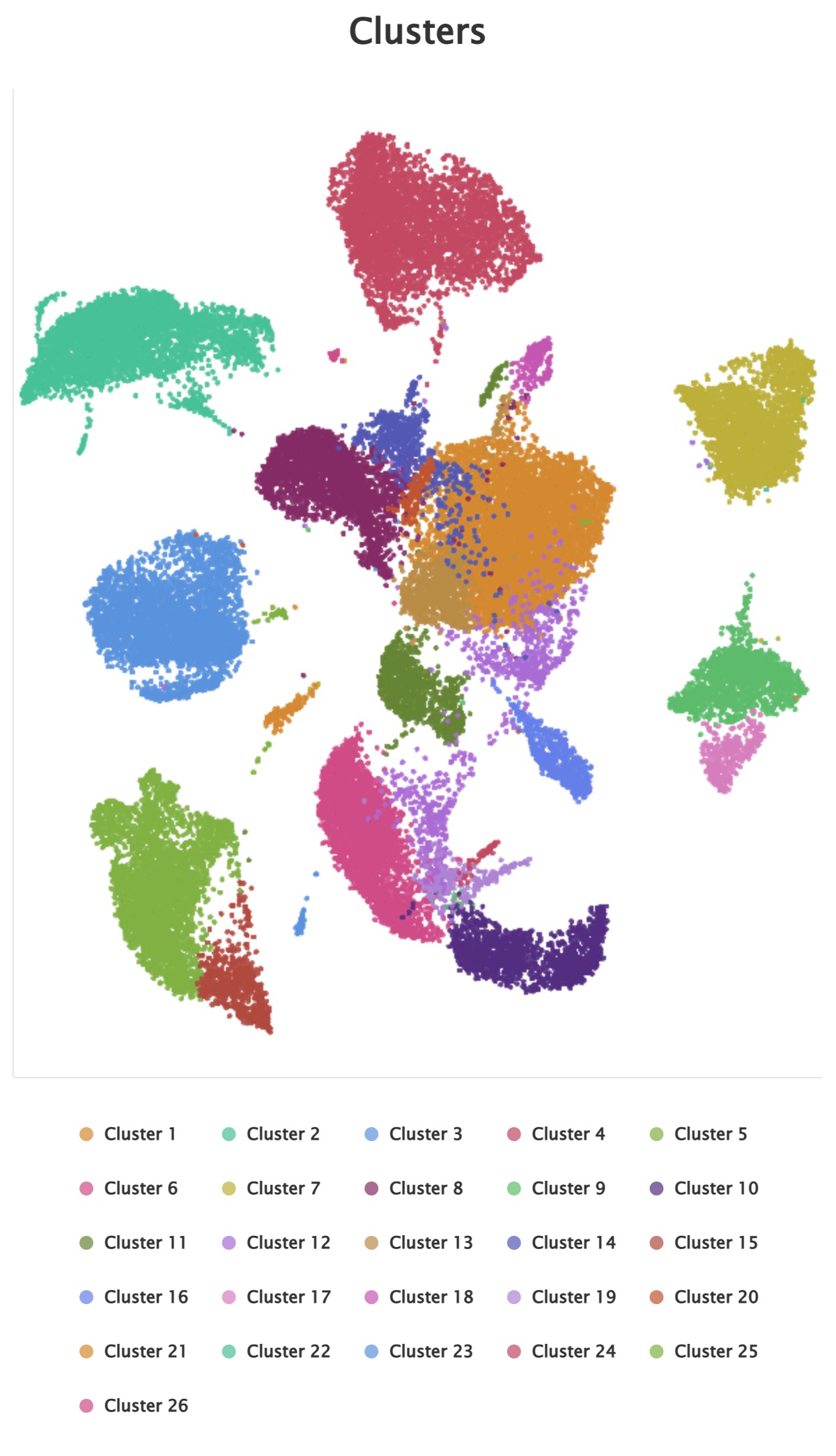

Supplement: Supplementary file 5 — Source Data for Expanded View and Appendix [file EMBR-24-e56030-s011.zip › Figure EV1-5, Appendix Figure S1-4/9. Appendix Figure S4/Appendix Figure S4B.jpeg]

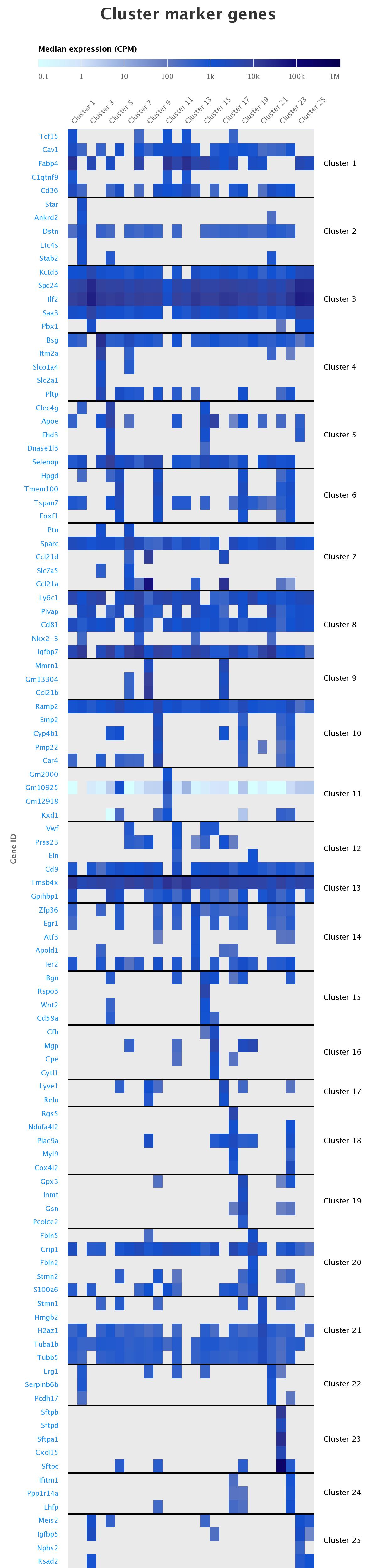

Supplement: Supplementary file 5 — Source Data for Expanded View and Appendix [file EMBR-24-e56030-s011.zip › Figure EV1-5, Appendix Figure S1-4/9. Appendix Figure S4/Appendix Figure S4C.jpeg]

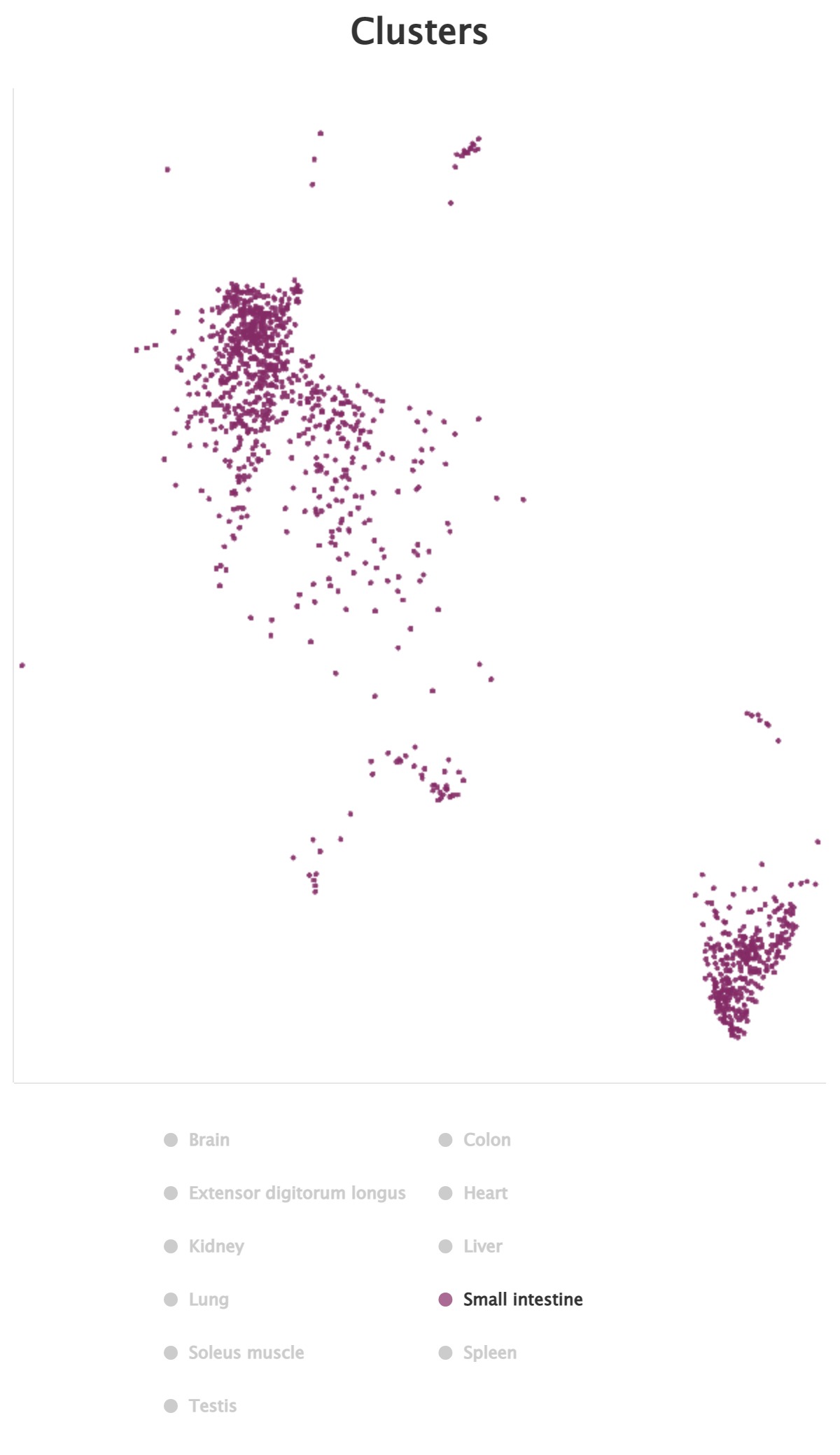

Supplement: Supplementary file 5 — Source Data for Expanded View and Appendix [file EMBR-24-e56030-s011.zip › Figure EV1-5, Appendix Figure S1-4/9. Appendix Figure S4/Appendix Figure S4D.jpeg]

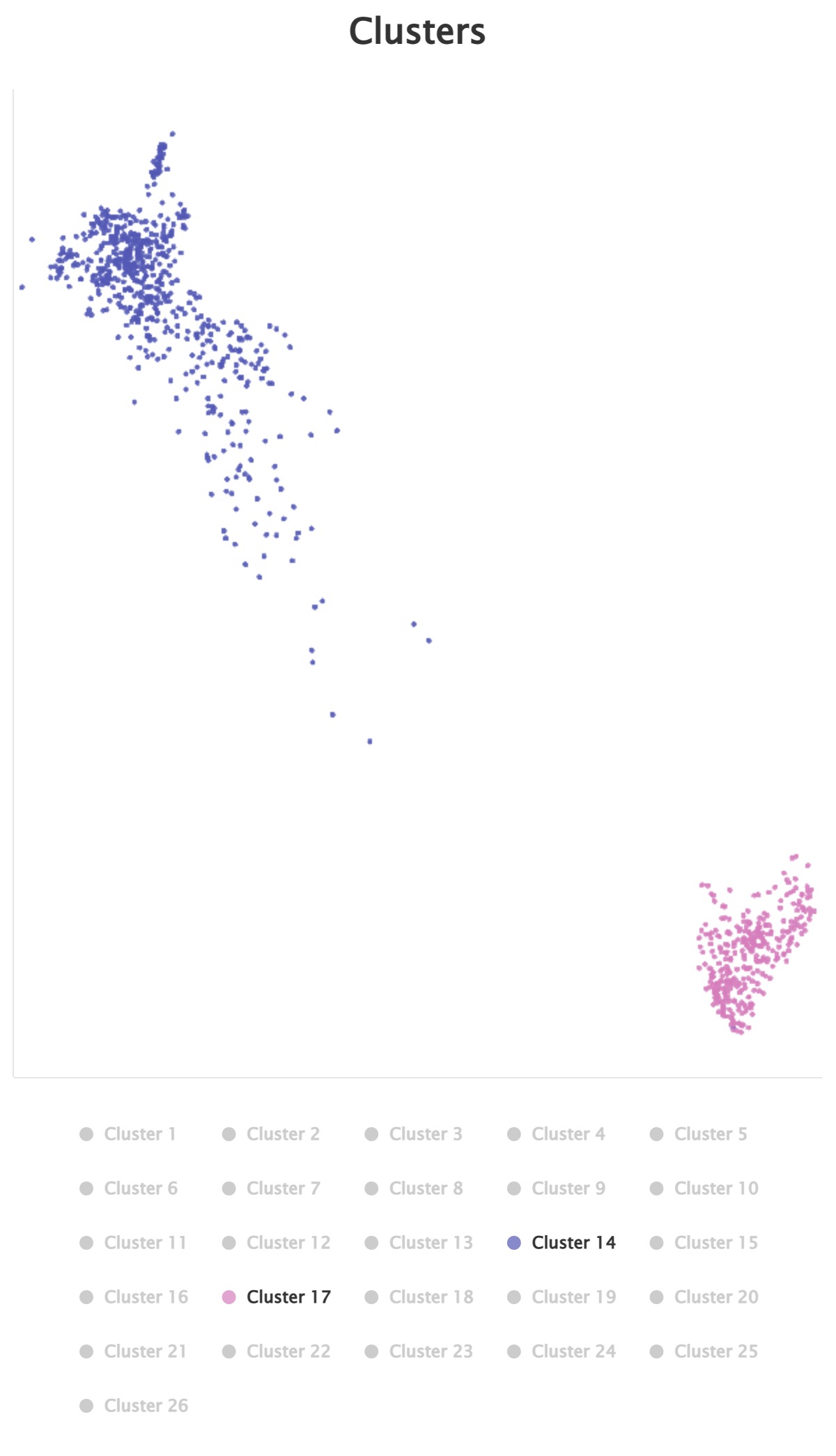

Supplement: Supplementary file 5 — Source Data for Expanded View and Appendix [file EMBR-24-e56030-s011.zip › Figure EV1-5, Appendix Figure S1-4/9. Appendix Figure S4/Appendix Figure S4E.jpeg]

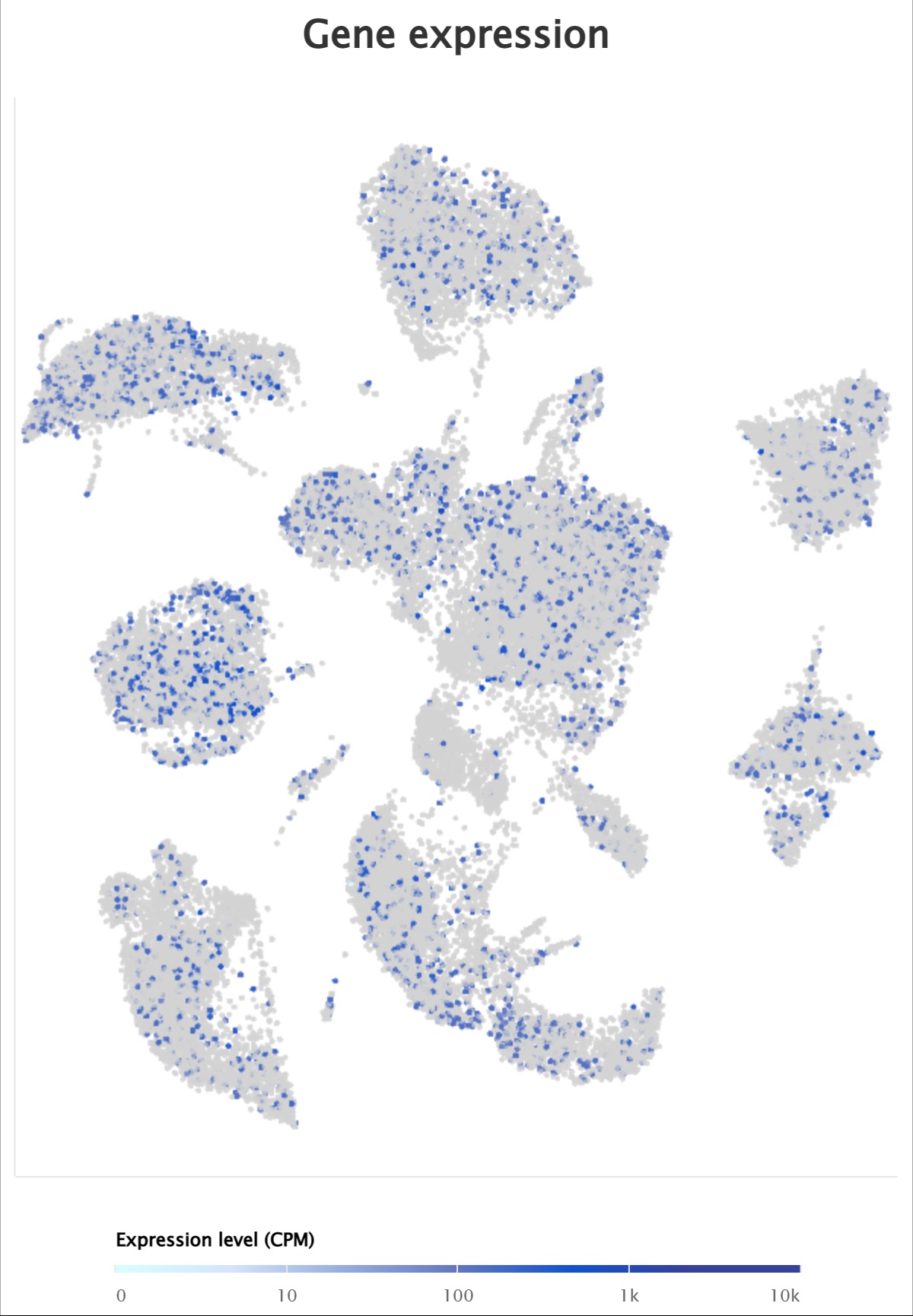

Supplement: Supplementary file 5 — Source Data for Expanded View and Appendix [file EMBR-24-e56030-s011.zip › Figure EV1-5, Appendix Figure S1-4/9. Appendix Figure S4/Appendix Figure S4F.jpeg]

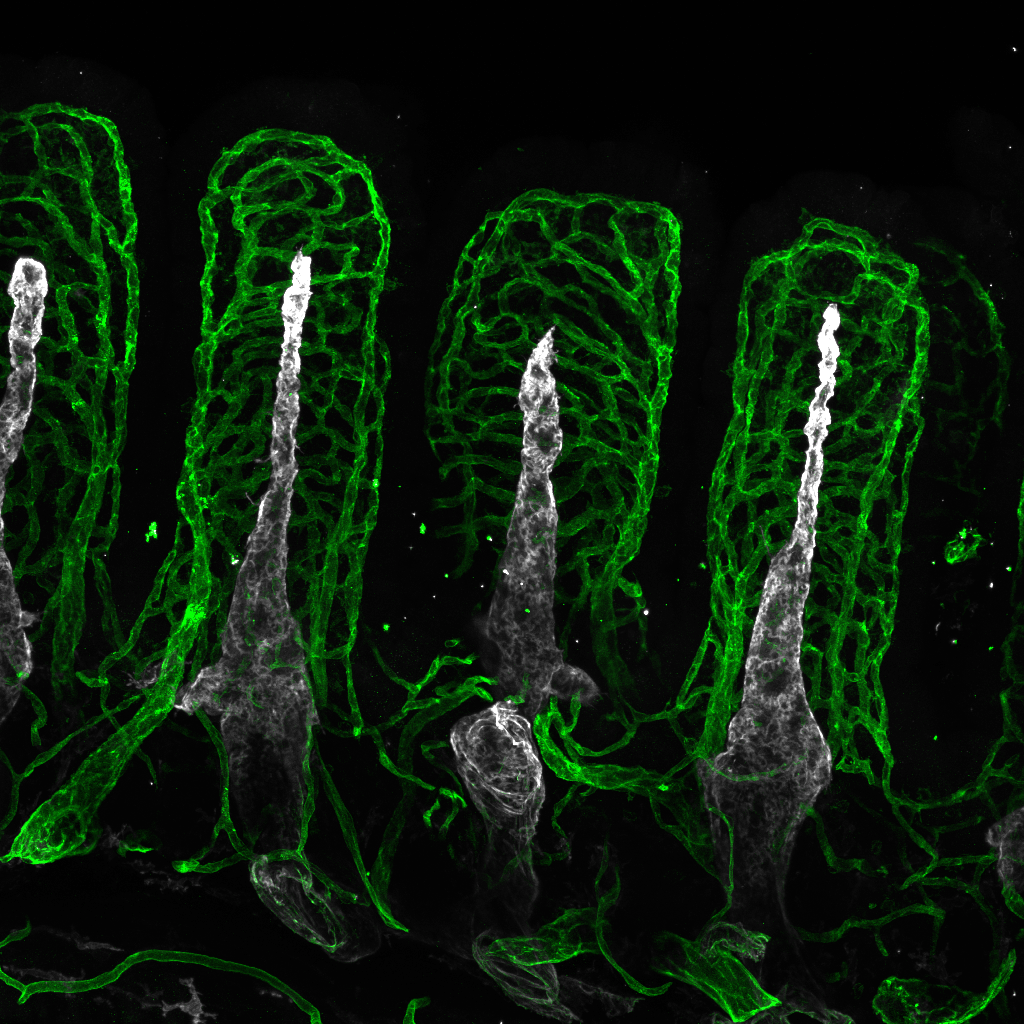

Supplement: Supplementary file 7 — Source Data for Figure 1 [file EMBR-24-e56030-s010.zip › Figure 1/Figure 1B WM-CD31 LYVE1/1. No surgery.tif]

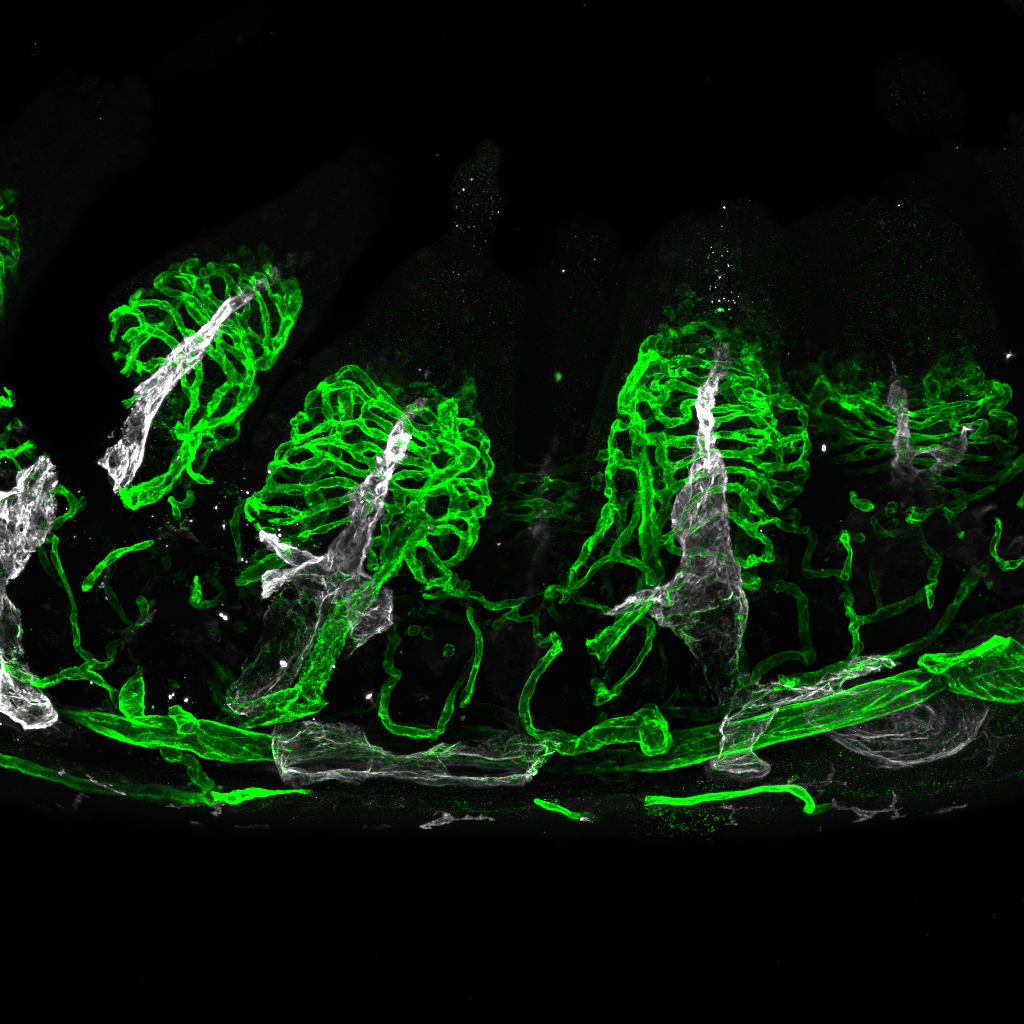

Supplement: Supplementary file 7 — Source Data for Figure 1 [file EMBR-24-e56030-s010.zip › Figure 1/Figure 1B WM-CD31 LYVE1/2. IR-4h.tif]

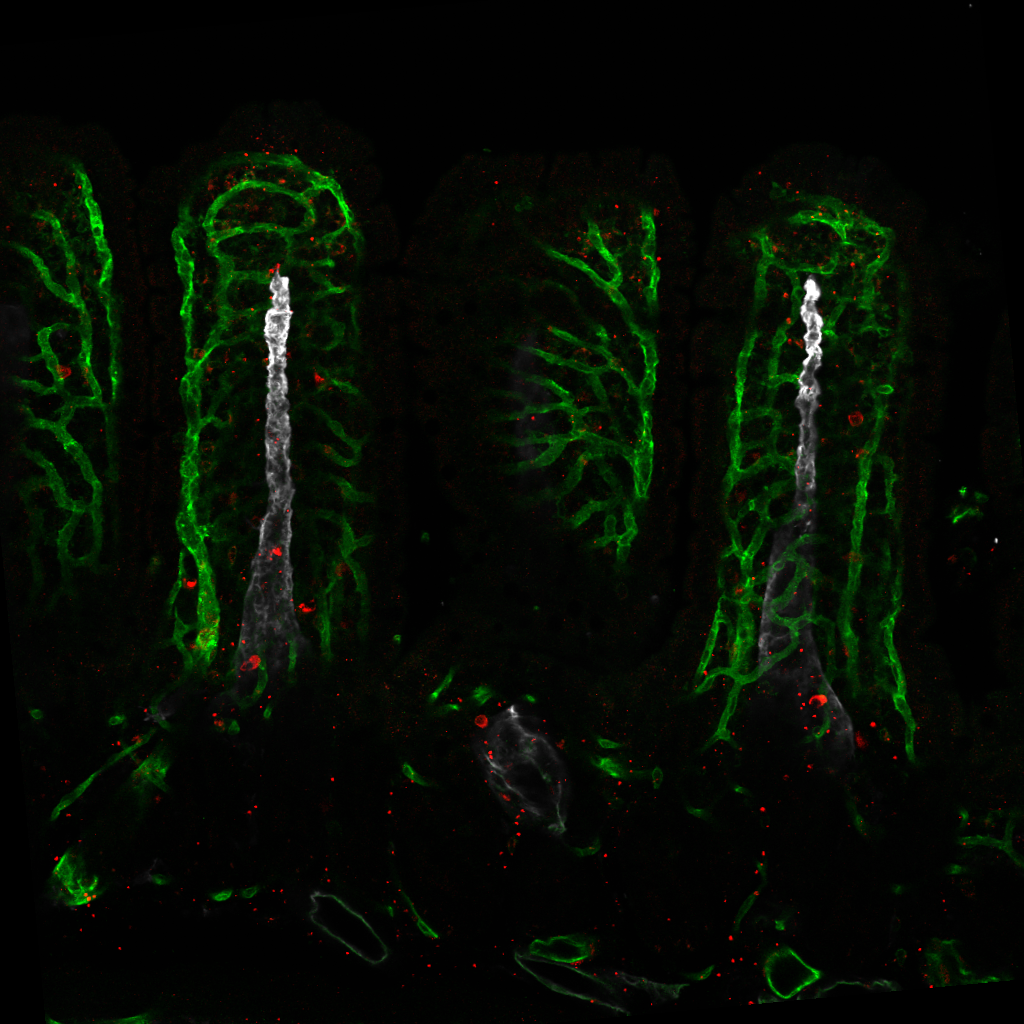

Supplement: Supplementary file 7 — Source Data for Figure 1 [file EMBR-24-e56030-s010.zip › Figure 1/Figure 1C WM-CD31 LYVE1 FOXC1/B1.tif]

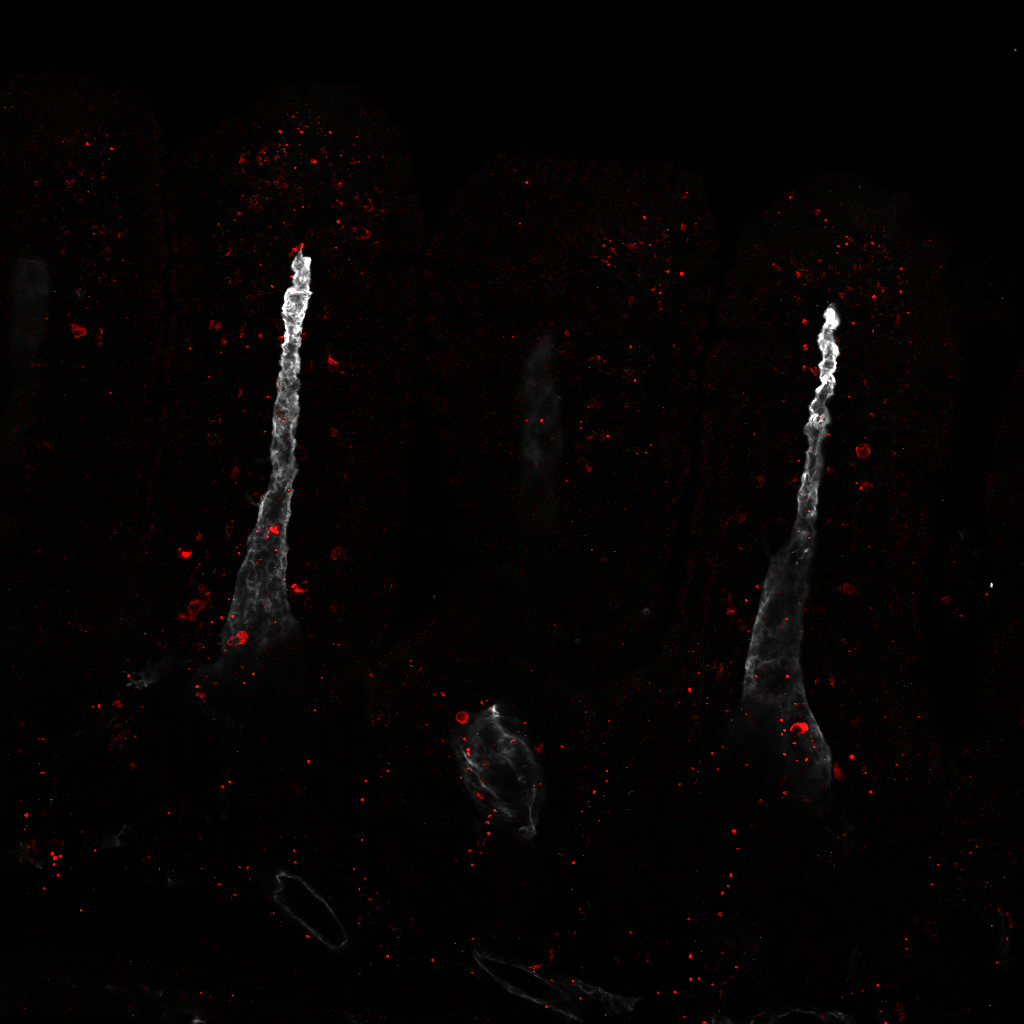

Supplement: Supplementary file 7 — Source Data for Figure 1 [file EMBR-24-e56030-s010.zip › Figure 1/Figure 1C WM-CD31 LYVE1 FOXC1/B2.tif]

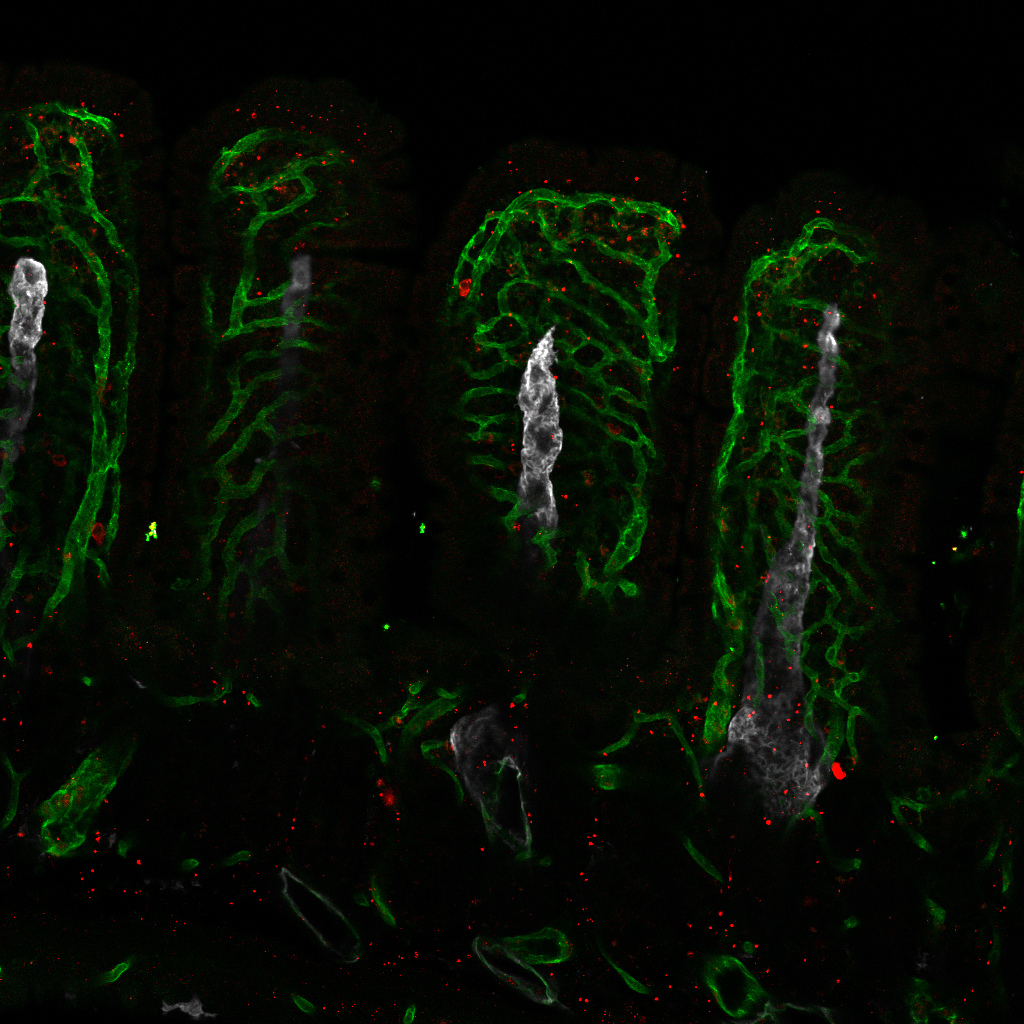

Supplement: Supplementary file 7 — Source Data for Figure 1 [file EMBR-24-e56030-s010.zip › Figure 1/Figure 1C WM-CD31 LYVE1 FOXC1/B3.tif]

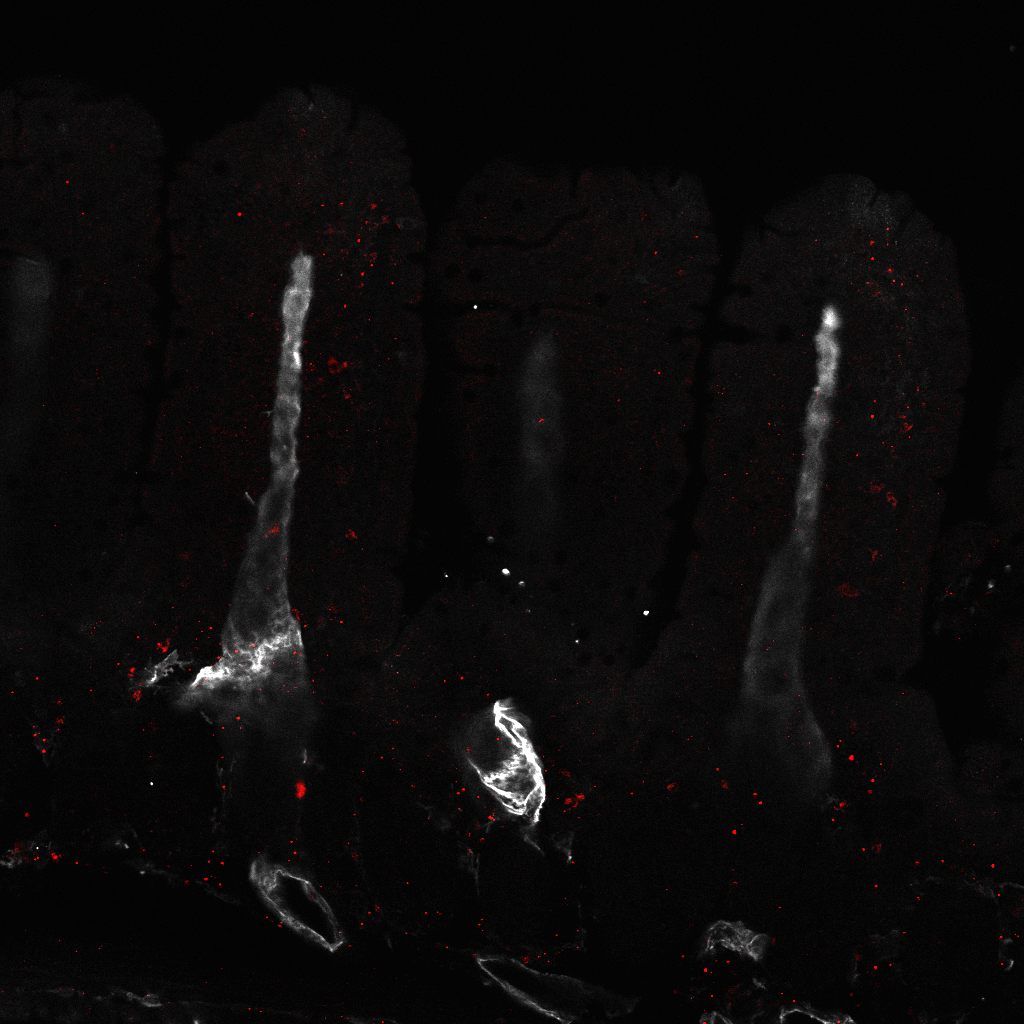

Supplement: Supplementary file 7 — Source Data for Figure 1 [file EMBR-24-e56030-s010.zip › Figure 1/Figure 1C WM-CD31 LYVE1 FOXC1/B4.tif]

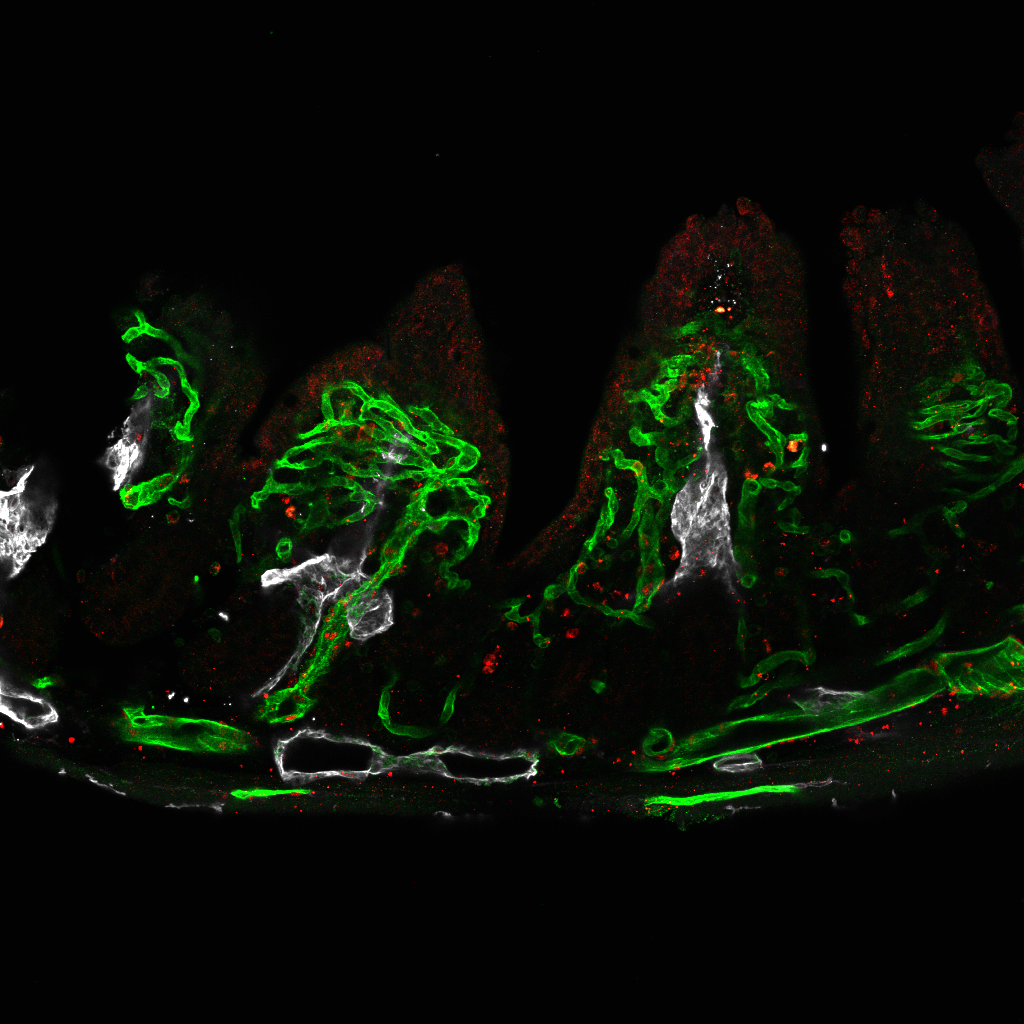

Supplement: Supplementary file 7 — Source Data for Figure 1 [file EMBR-24-e56030-s010.zip › Figure 1/Figure 1C WM-CD31 LYVE1 FOXC1/B5.tif]

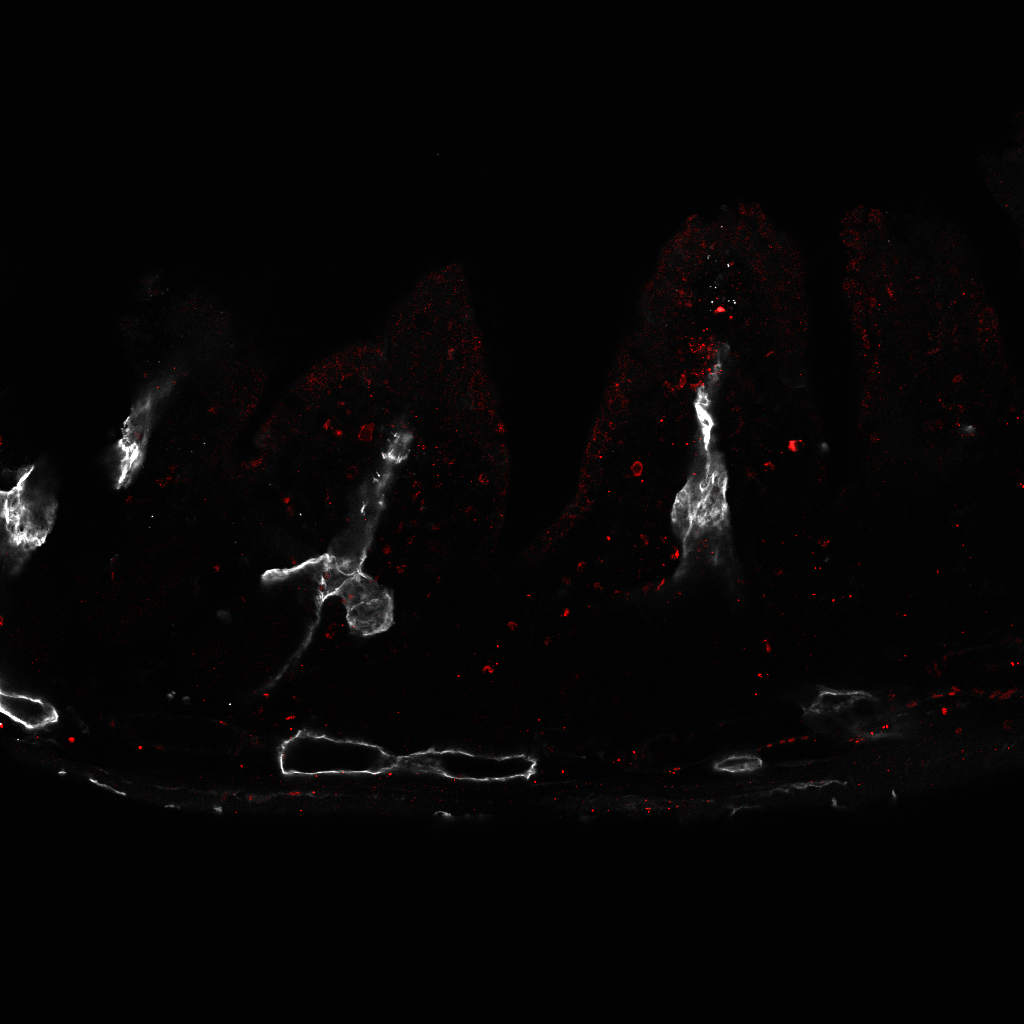

Supplement: Supplementary file 7 — Source Data for Figure 1 [file EMBR-24-e56030-s010.zip › Figure 1/Figure 1C WM-CD31 LYVE1 FOXC1/B6.tif]
