# Supplementary figures and images for: Endothelial FOXC1 and FOXC2 promote intestinal regeneration after ischemia–reperfusion injury (part 2 of 3)
Source: EMBO Rep. 2023 May 8;24(7):e56030. doi: 10.15252/embr.202256030 (PMC10328078; doi:10.15252/embr.202256030)

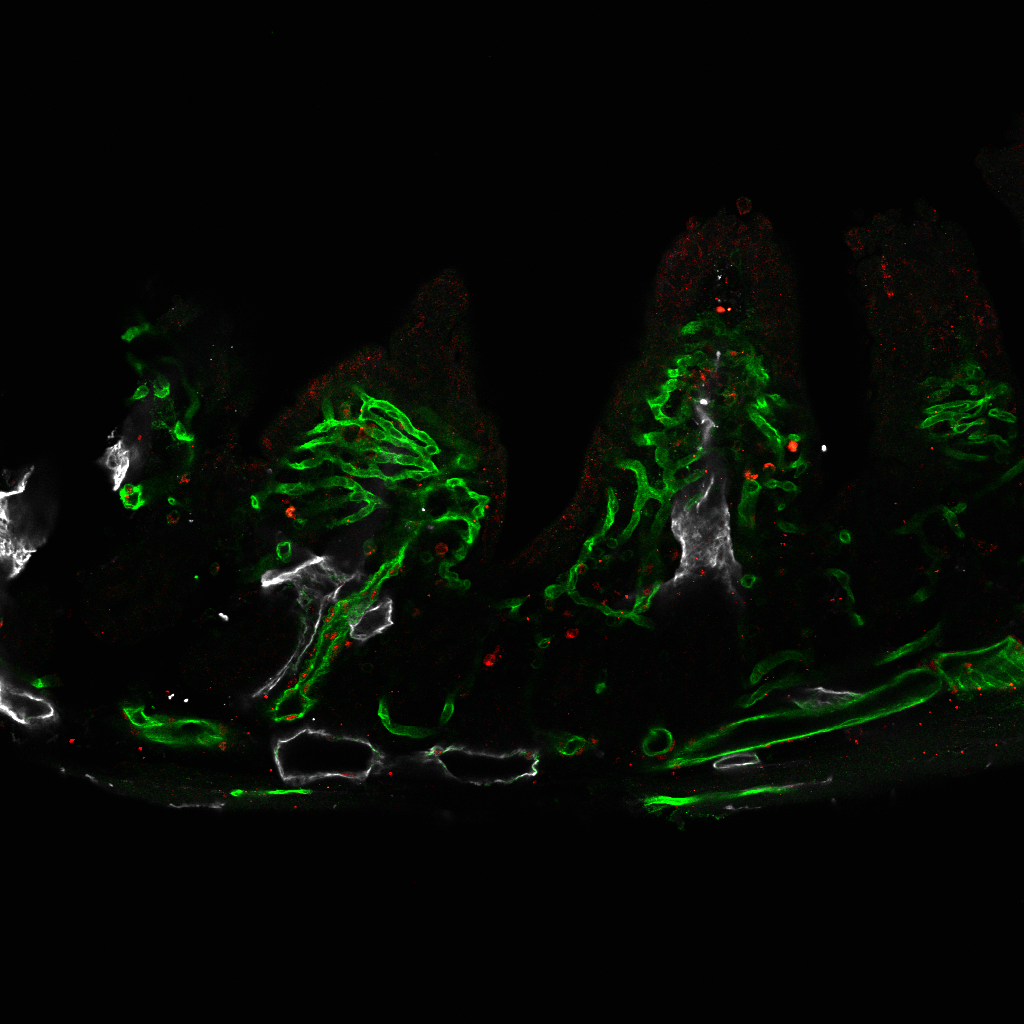

Supplement: Supplementary file 7 — Source Data for Figure 1 [file EMBR-24-e56030-s010.zip › Figure 1/Figure 1C WM-CD31 LYVE1 FOXC1/B7.tif]

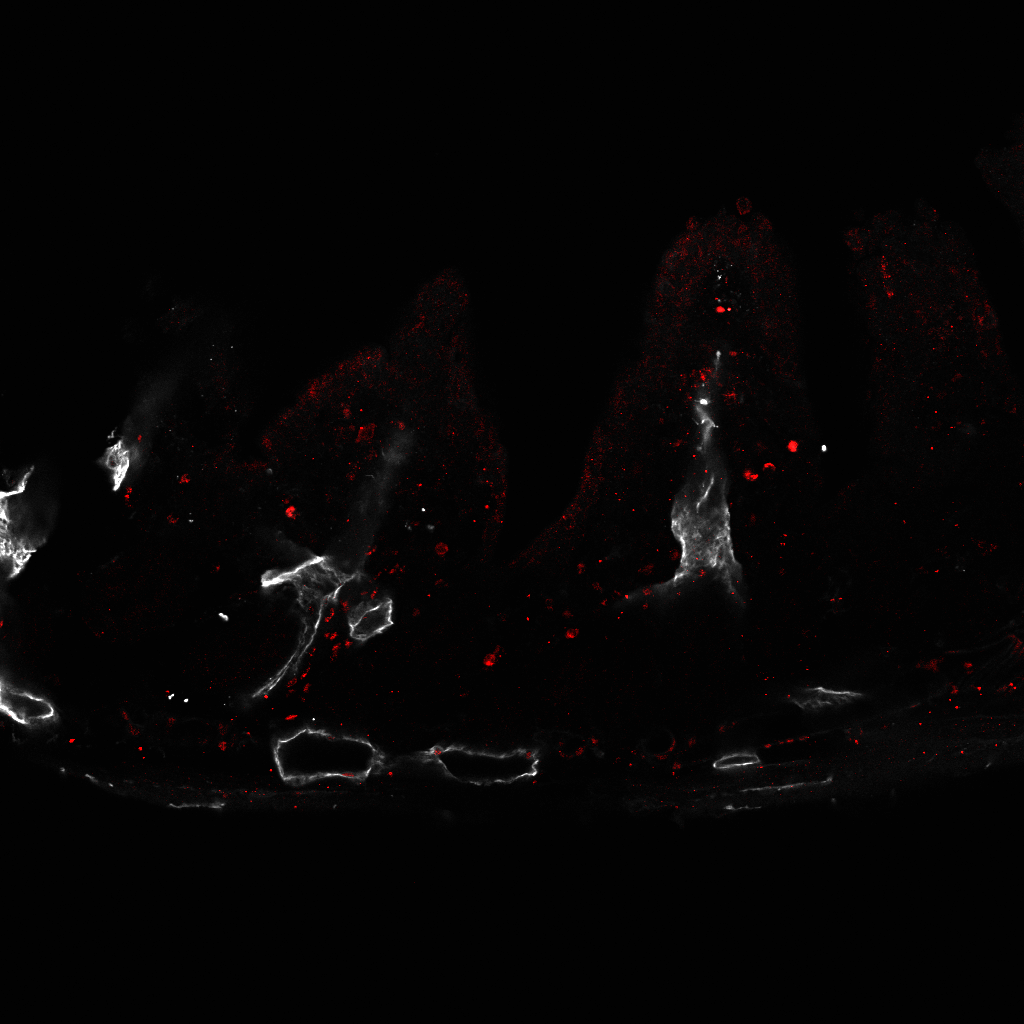

Supplement: Supplementary file 7 — Source Data for Figure 1 [file EMBR-24-e56030-s010.zip › Figure 1/Figure 1C WM-CD31 LYVE1 FOXC1/B8.tif]

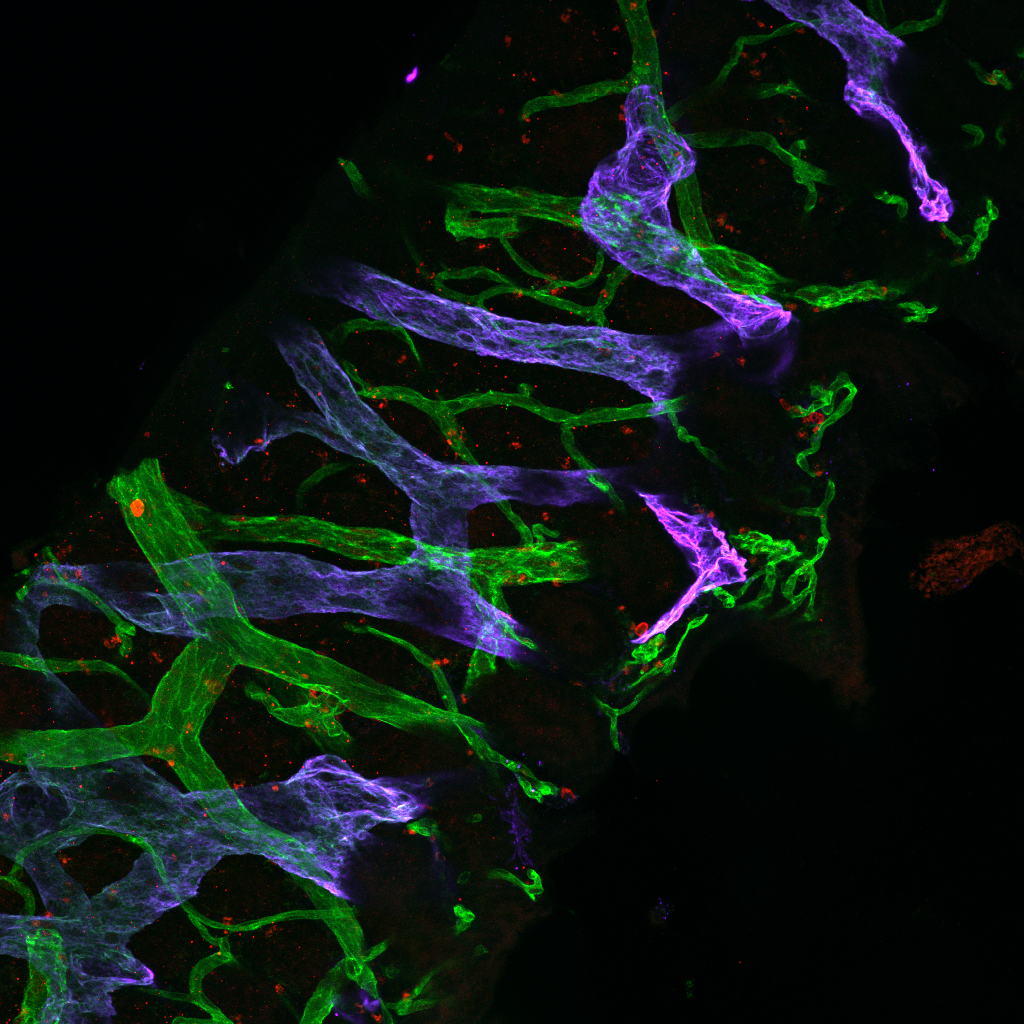

Supplement: Supplementary file 7 — Source Data for Figure 1 [file EMBR-24-e56030-s010.zip › Figure 1/Figure 1G WM-CD31 LYVE1 FOXC1/Control.tif]

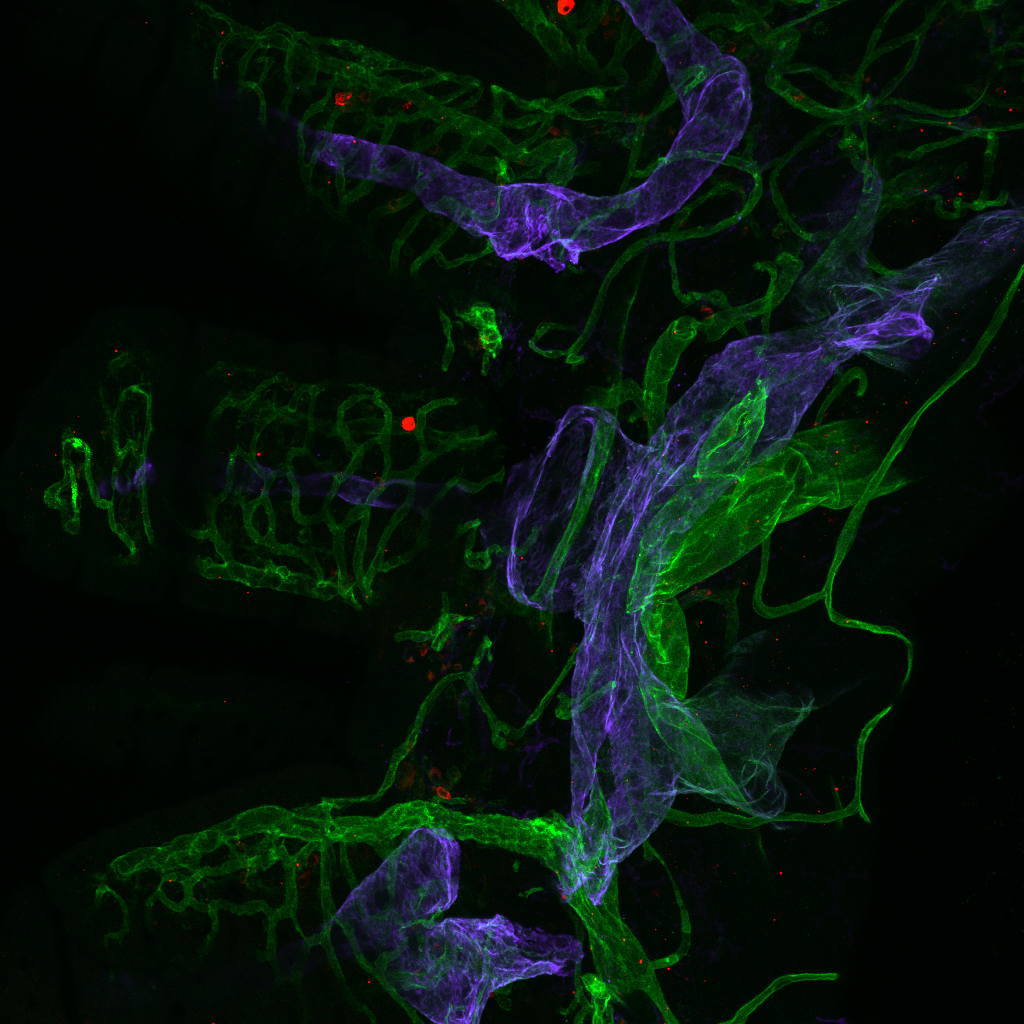

Supplement: Supplementary file 7 — Source Data for Figure 1 [file EMBR-24-e56030-s010.zip › Figure 1/Figure 1G WM-CD31 LYVE1 FOXC1/EC-Foxc-DKO.tif]

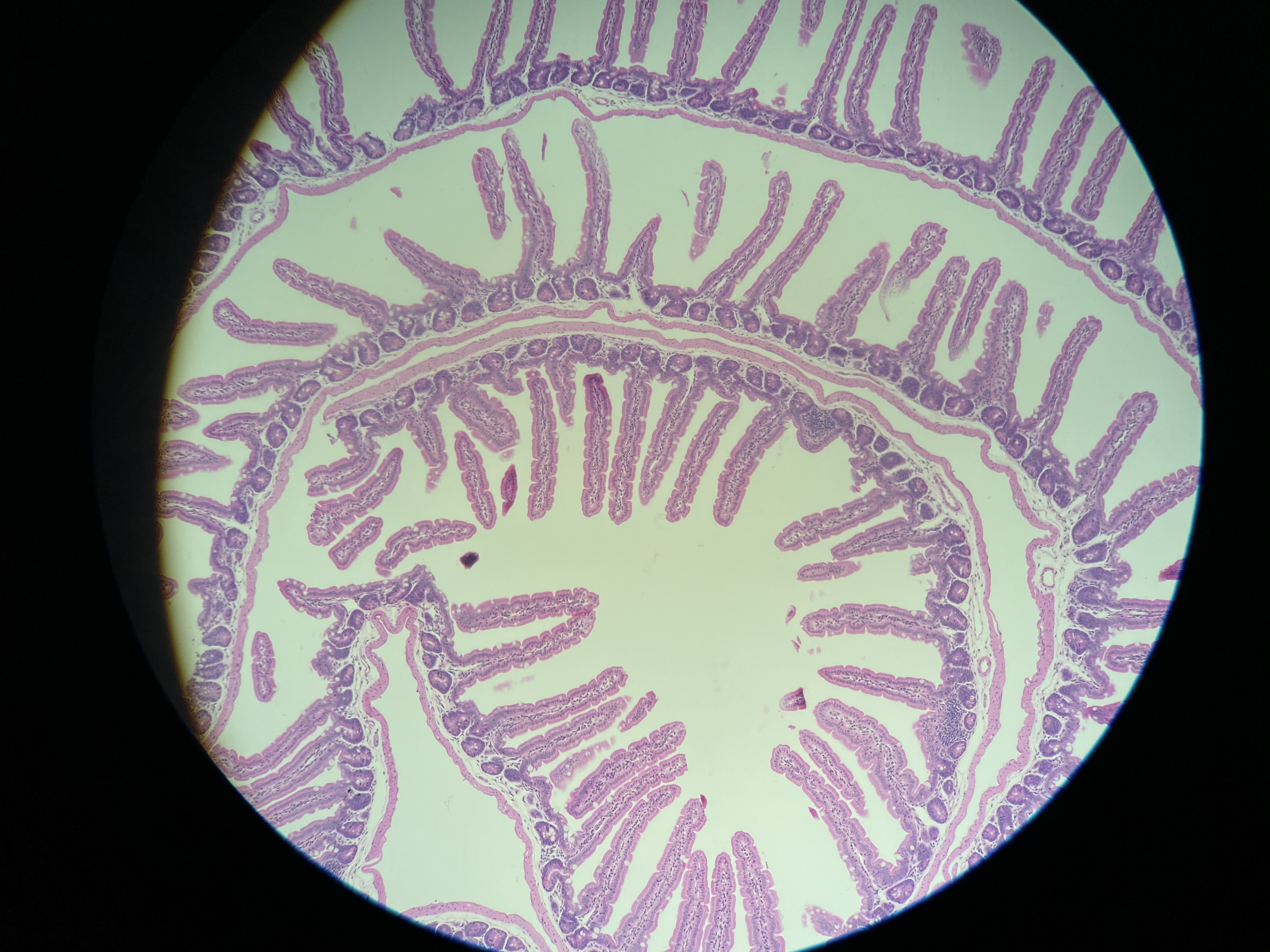

Supplement: Supplementary file 8 — Source Data for Figure 2 [file EMBR-24-e56030-s016.zip › Figure 2/Figure 2B-HE/1. Control sham.jpeg]

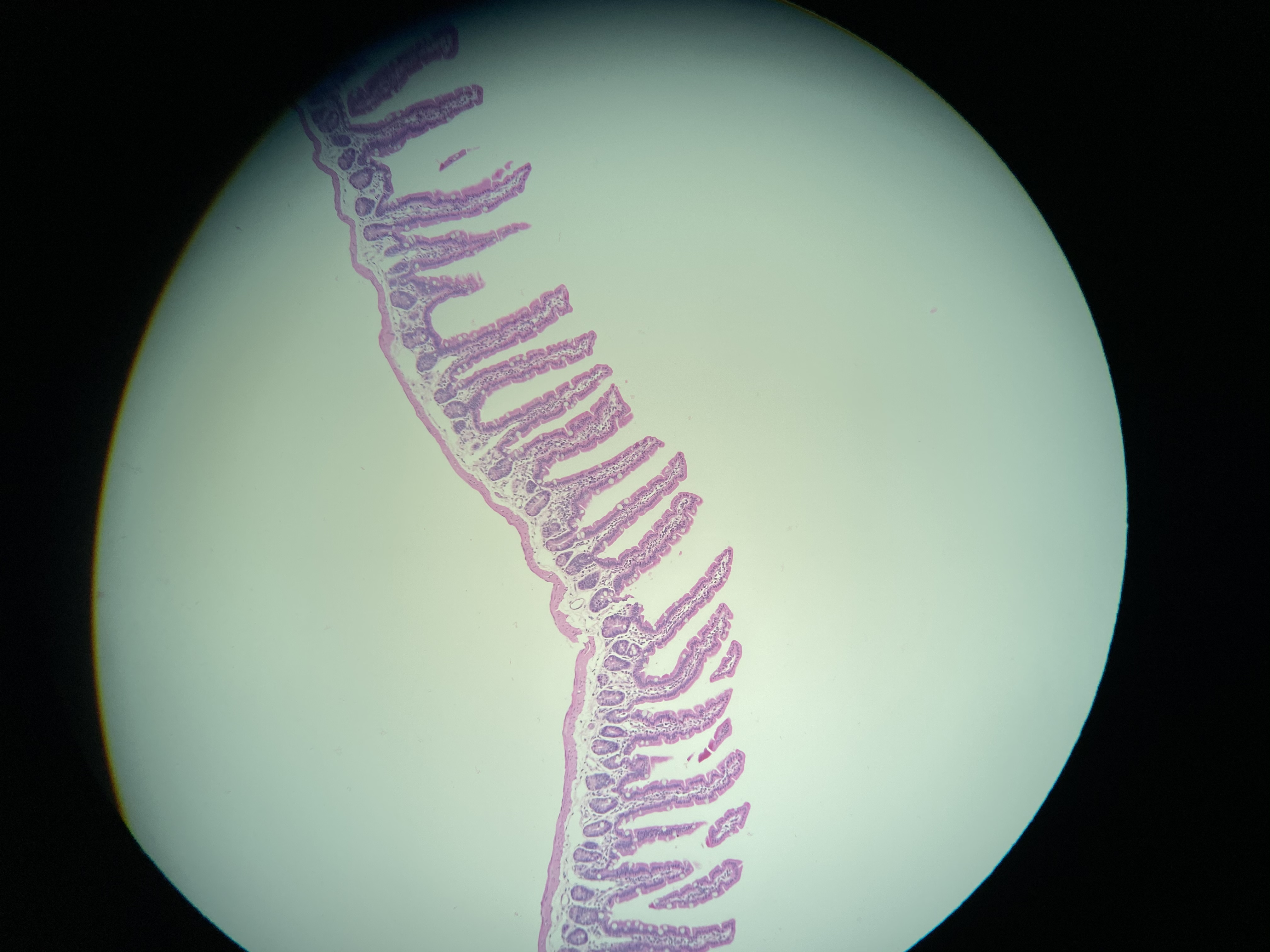

Supplement: Supplementary file 8 — Source Data for Figure 2 [file EMBR-24-e56030-s016.zip › Figure 2/Figure 2B-HE/2. EC-Foxc-DKO sham.jpeg]

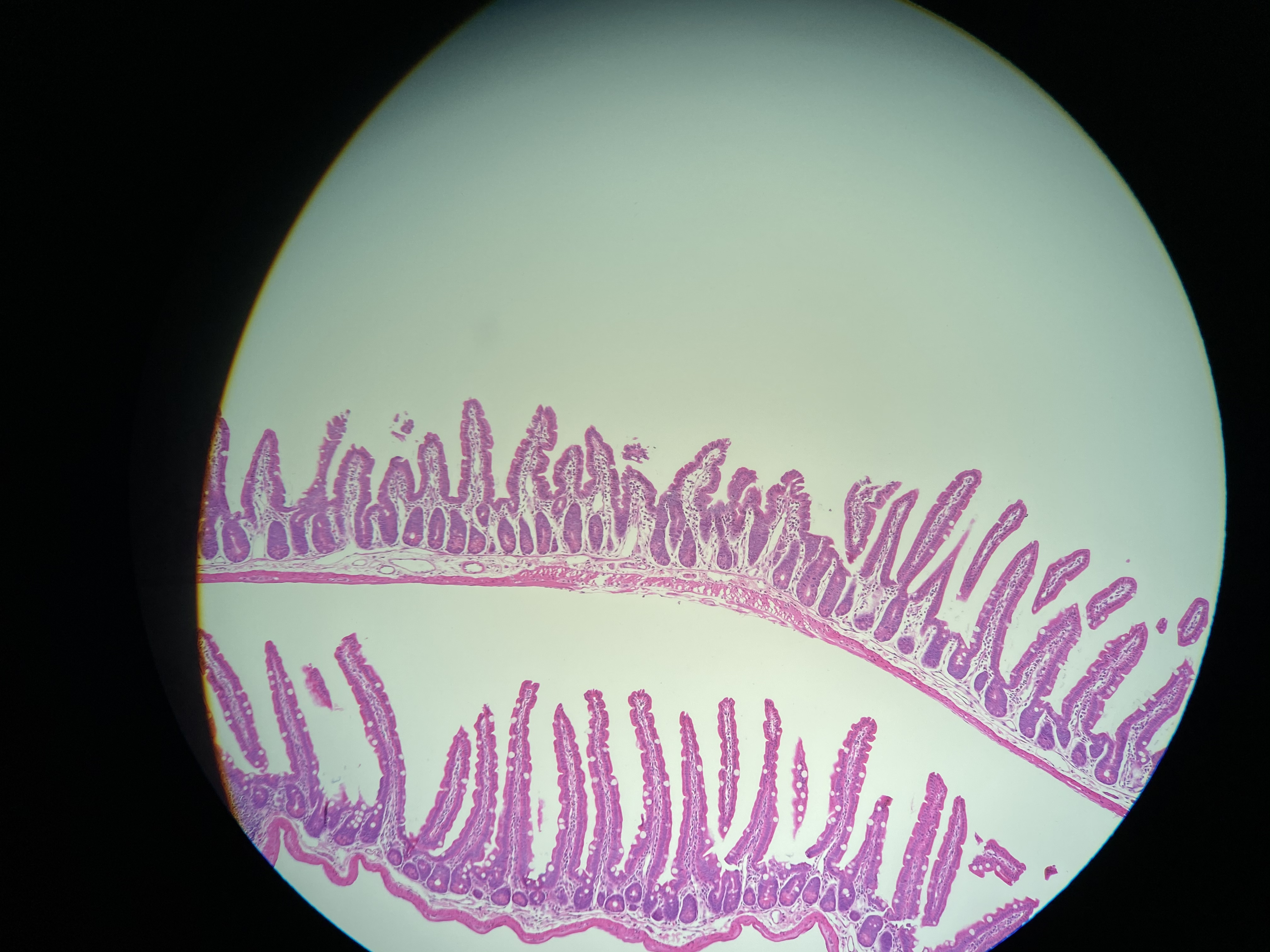

Supplement: Supplementary file 8 — Source Data for Figure 2 [file EMBR-24-e56030-s016.zip › Figure 2/Figure 2B-HE/3. Control IR.jpeg]

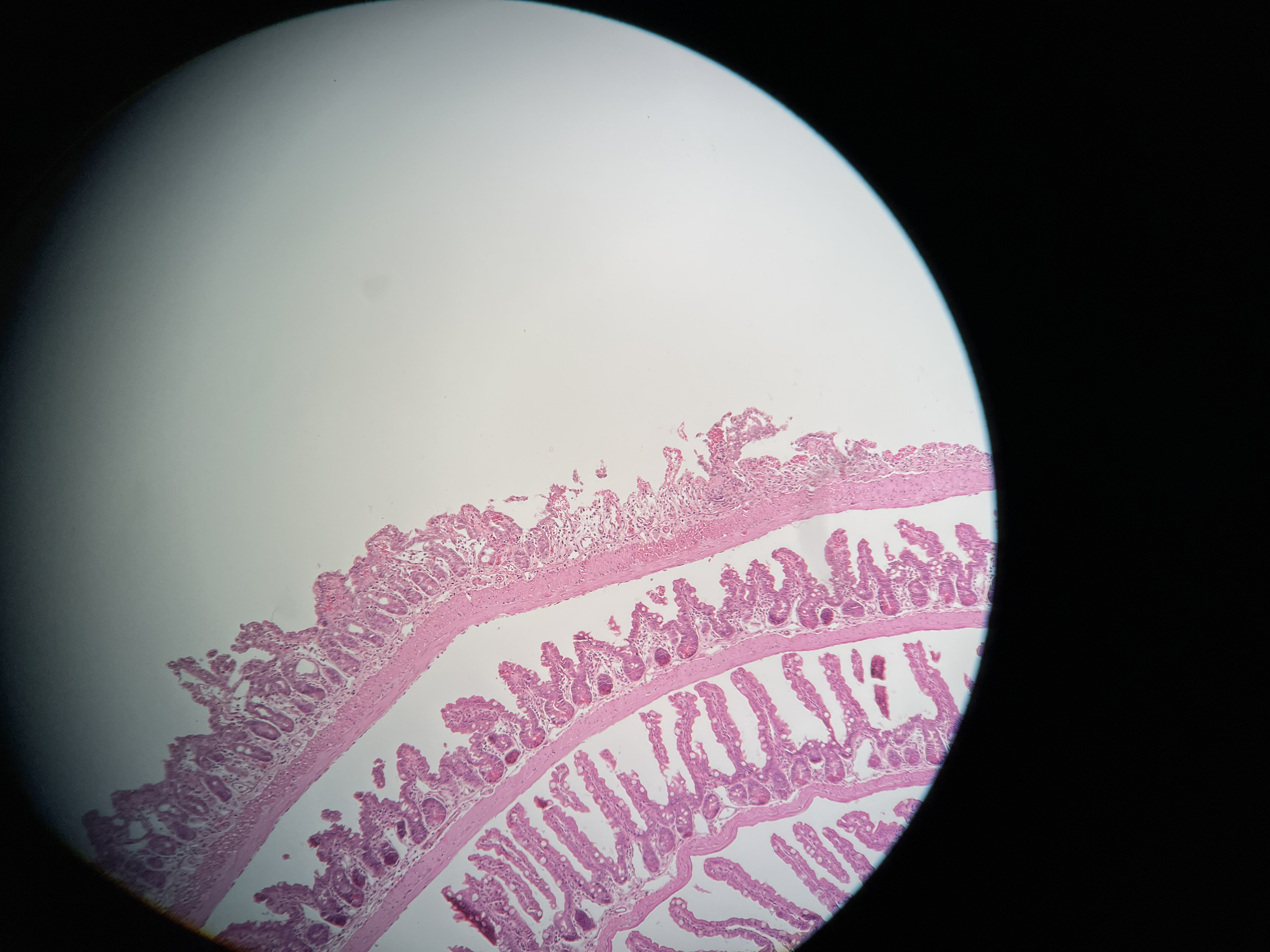

Supplement: Supplementary file 8 — Source Data for Figure 2 [file EMBR-24-e56030-s016.zip › Figure 2/Figure 2B-HE/4. EC-Foxc-DKO IR.jpeg]

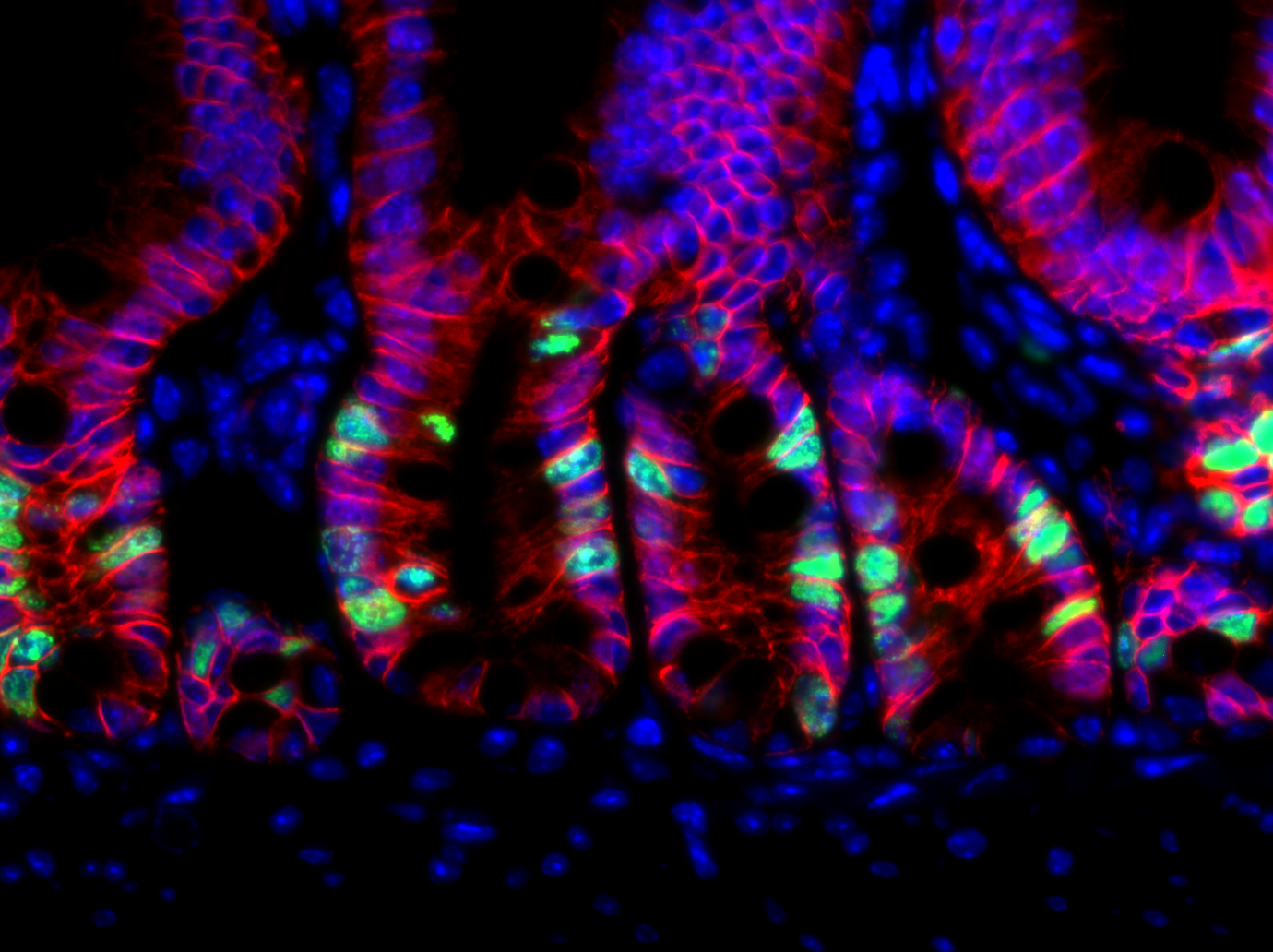

Supplement: Supplementary file 8 — Source Data for Figure 2 [file EMBR-24-e56030-s016.zip › Figure 2/Figure 2G-BrdU&EpCAM staining/1. Control, sham.TIF]

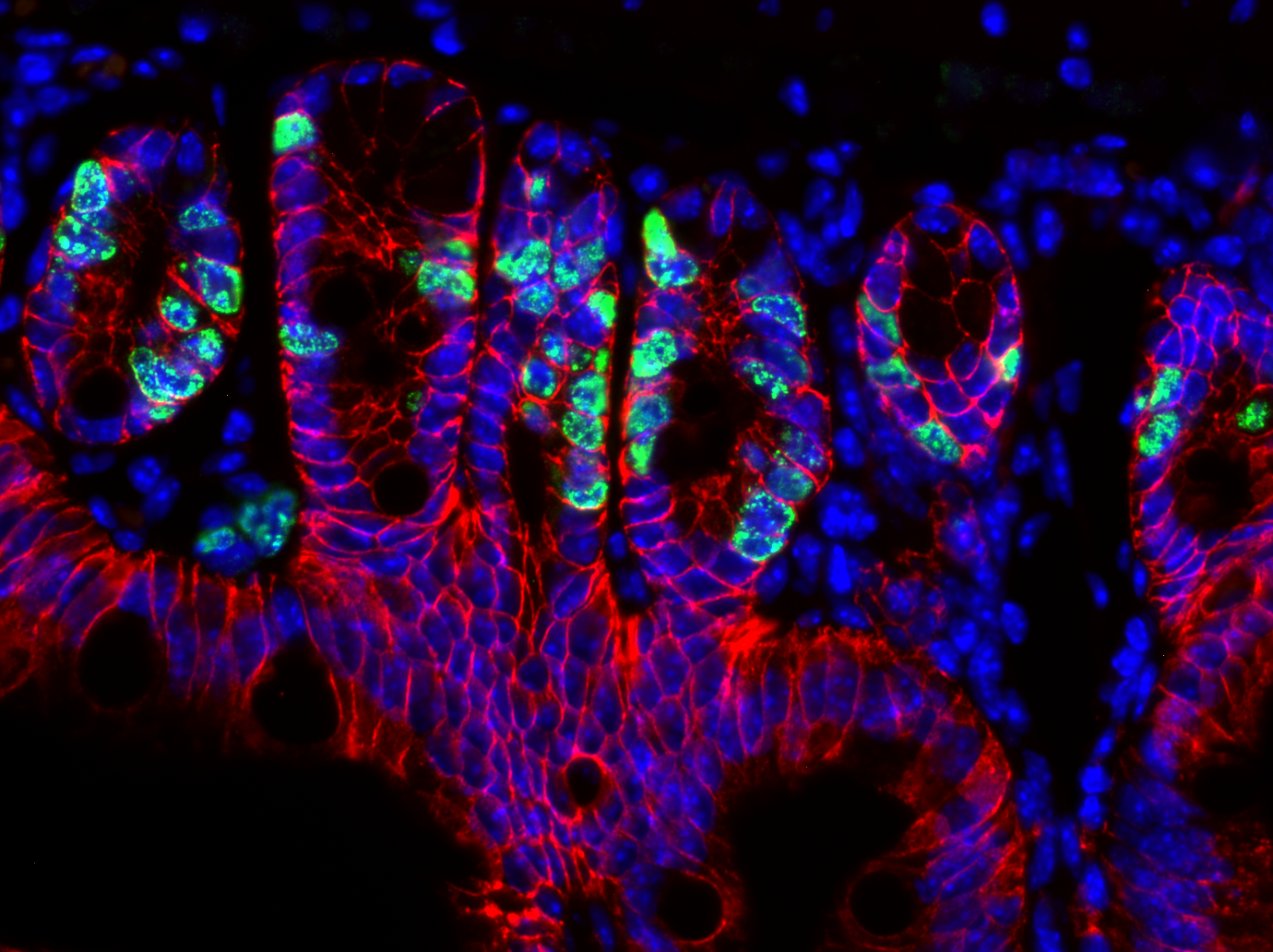

Supplement: Supplementary file 8 — Source Data for Figure 2 [file EMBR-24-e56030-s016.zip › Figure 2/Figure 2G-BrdU&EpCAM staining/2. EC-Foxc-DKO, sham.TIF]

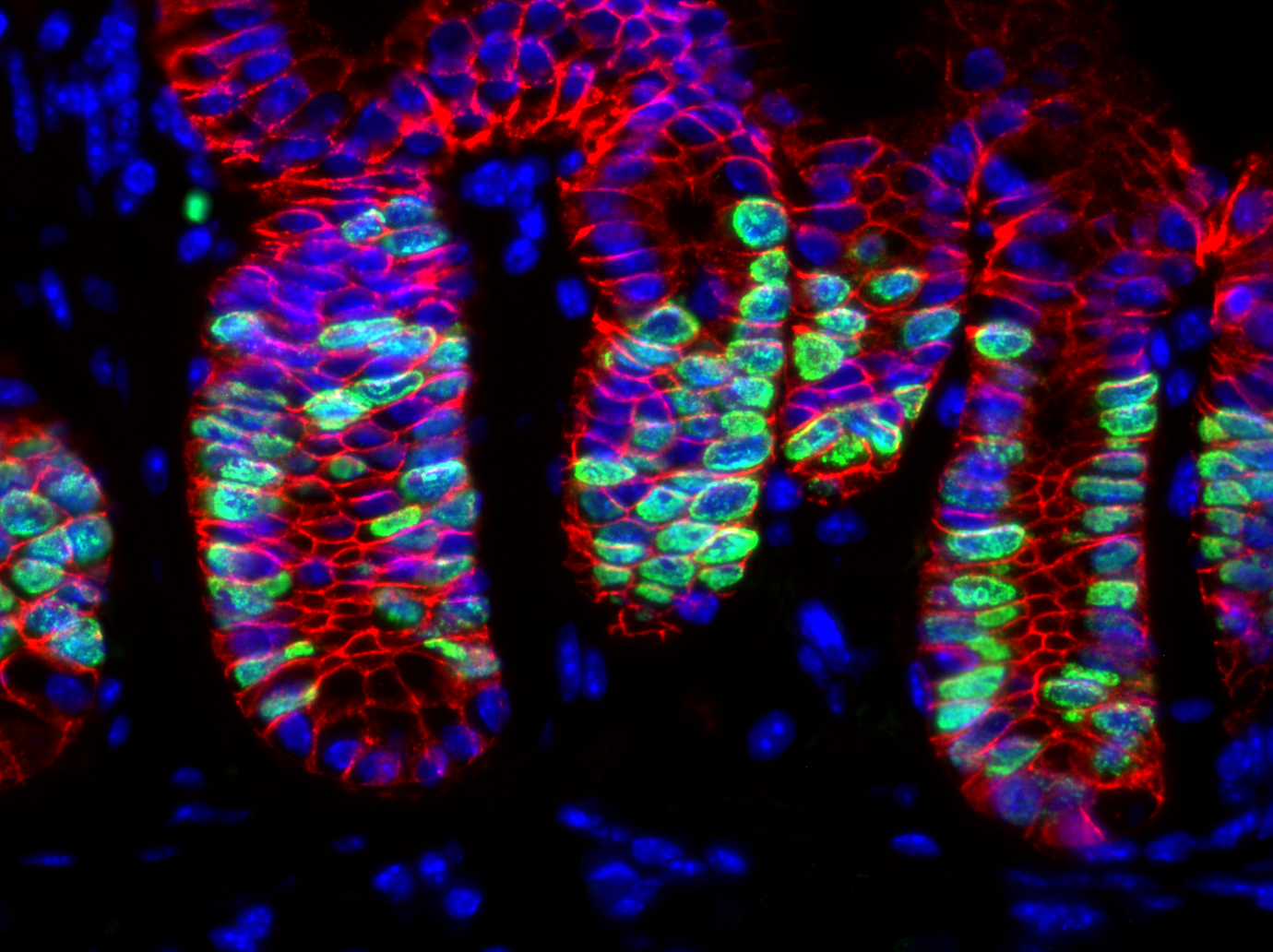

Supplement: Supplementary file 8 — Source Data for Figure 2 [file EMBR-24-e56030-s016.zip › Figure 2/Figure 2G-BrdU&EpCAM staining/3. Control, IR.TIF]

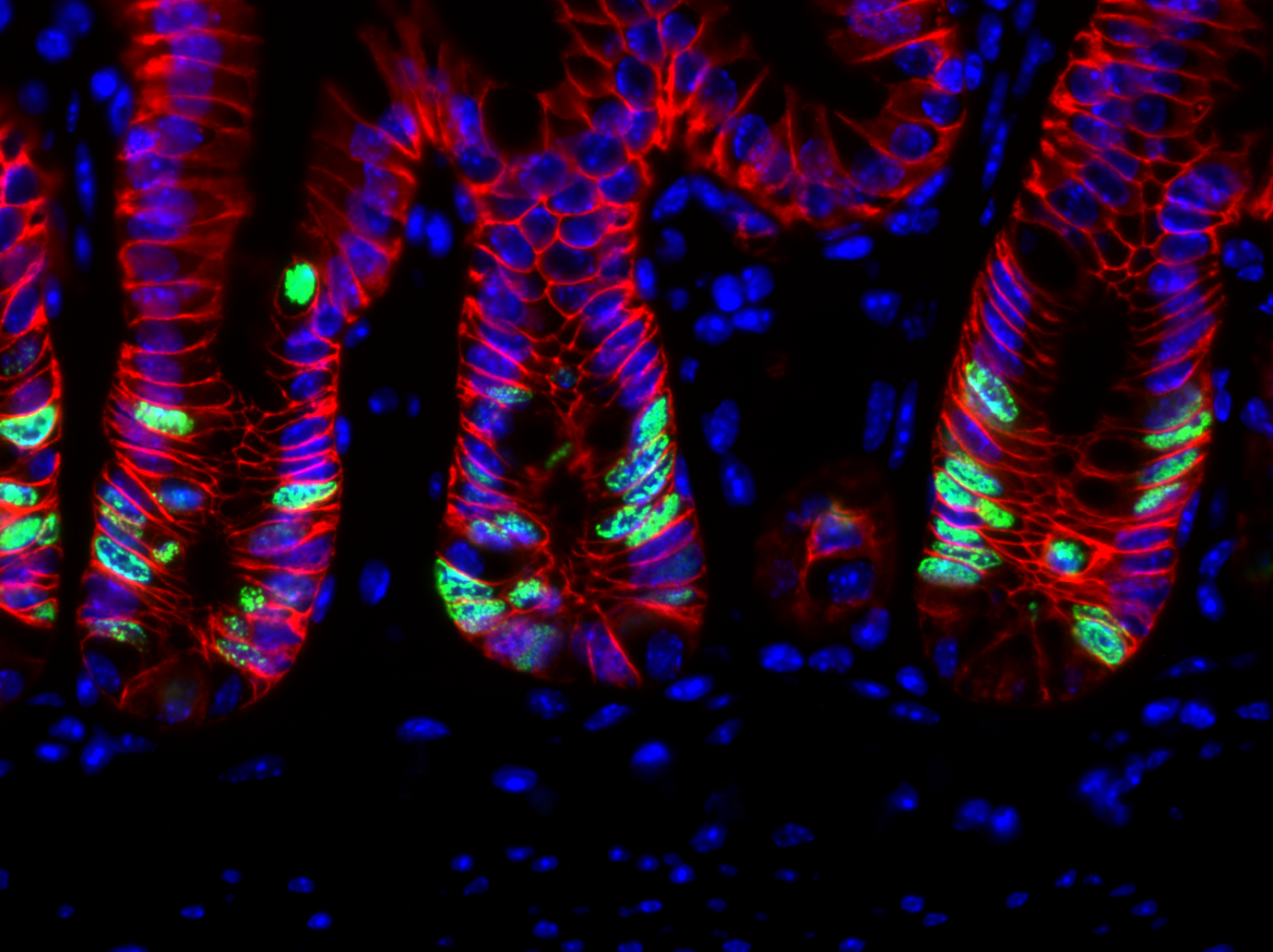

Supplement: Supplementary file 8 — Source Data for Figure 2 [file EMBR-24-e56030-s016.zip › Figure 2/Figure 2G-BrdU&EpCAM staining/4. EC-Foxc-DKO, IR.TIF]

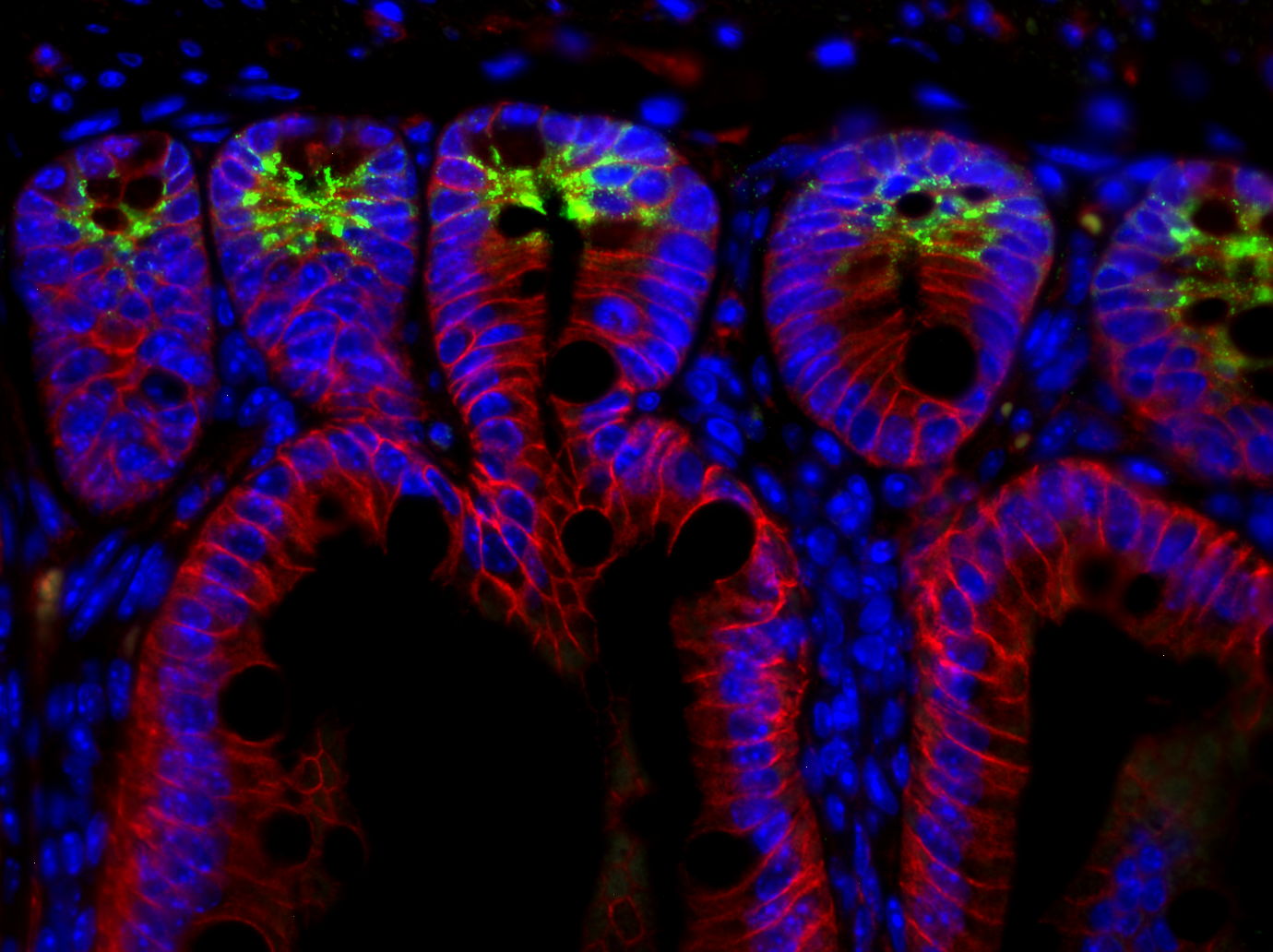

Supplement: Supplementary file 8 — Source Data for Figure 2 [file EMBR-24-e56030-s016.zip › Figure 2/Figure 2I-IHC-b-catenin OLFM4/1-1. Control, sham, 3 colors.TIF]

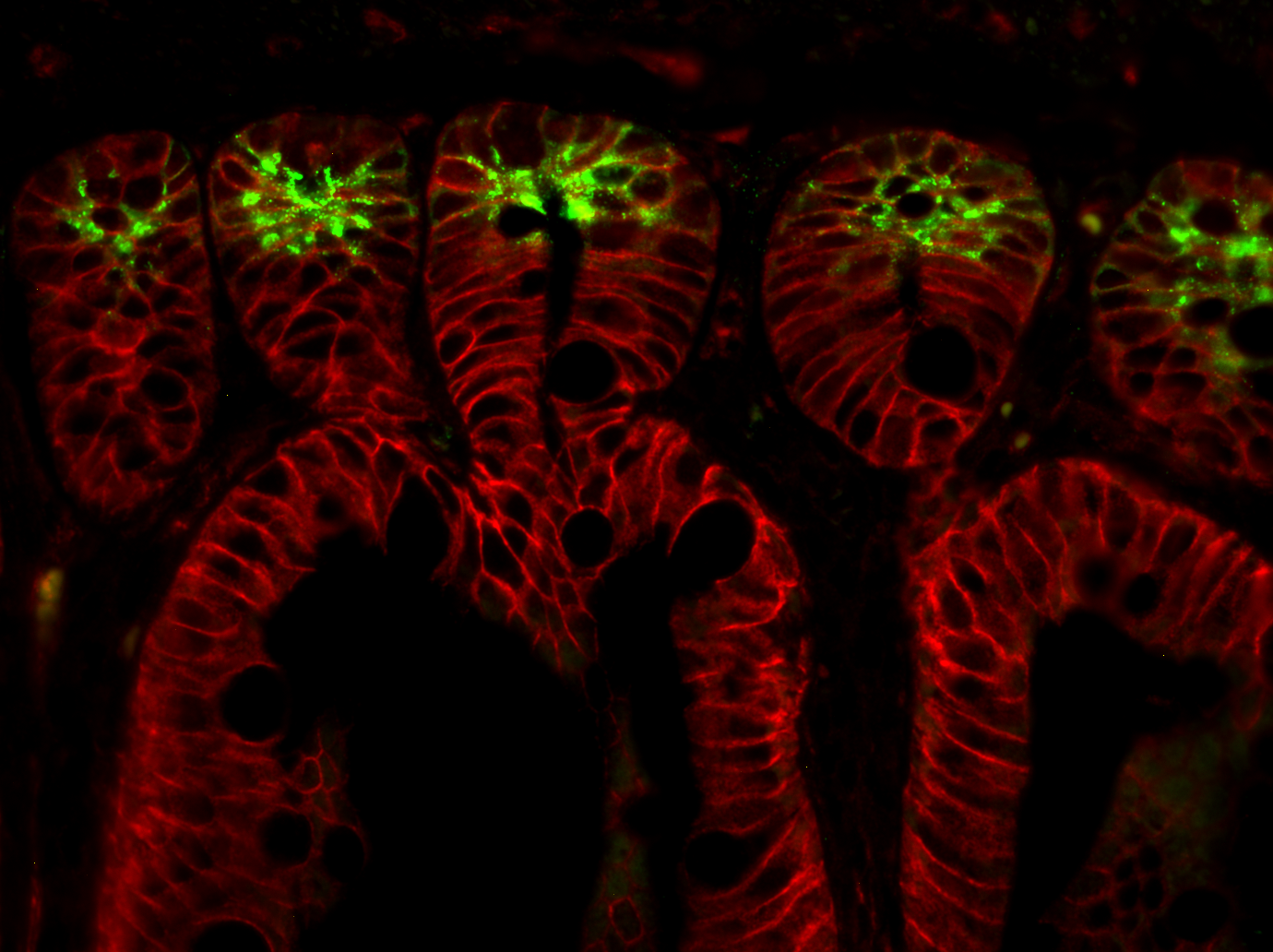

Supplement: Supplementary file 8 — Source Data for Figure 2 [file EMBR-24-e56030-s016.zip › Figure 2/Figure 2I-IHC-b-catenin OLFM4/1-2. Control, sham, 2 colors.TIF]

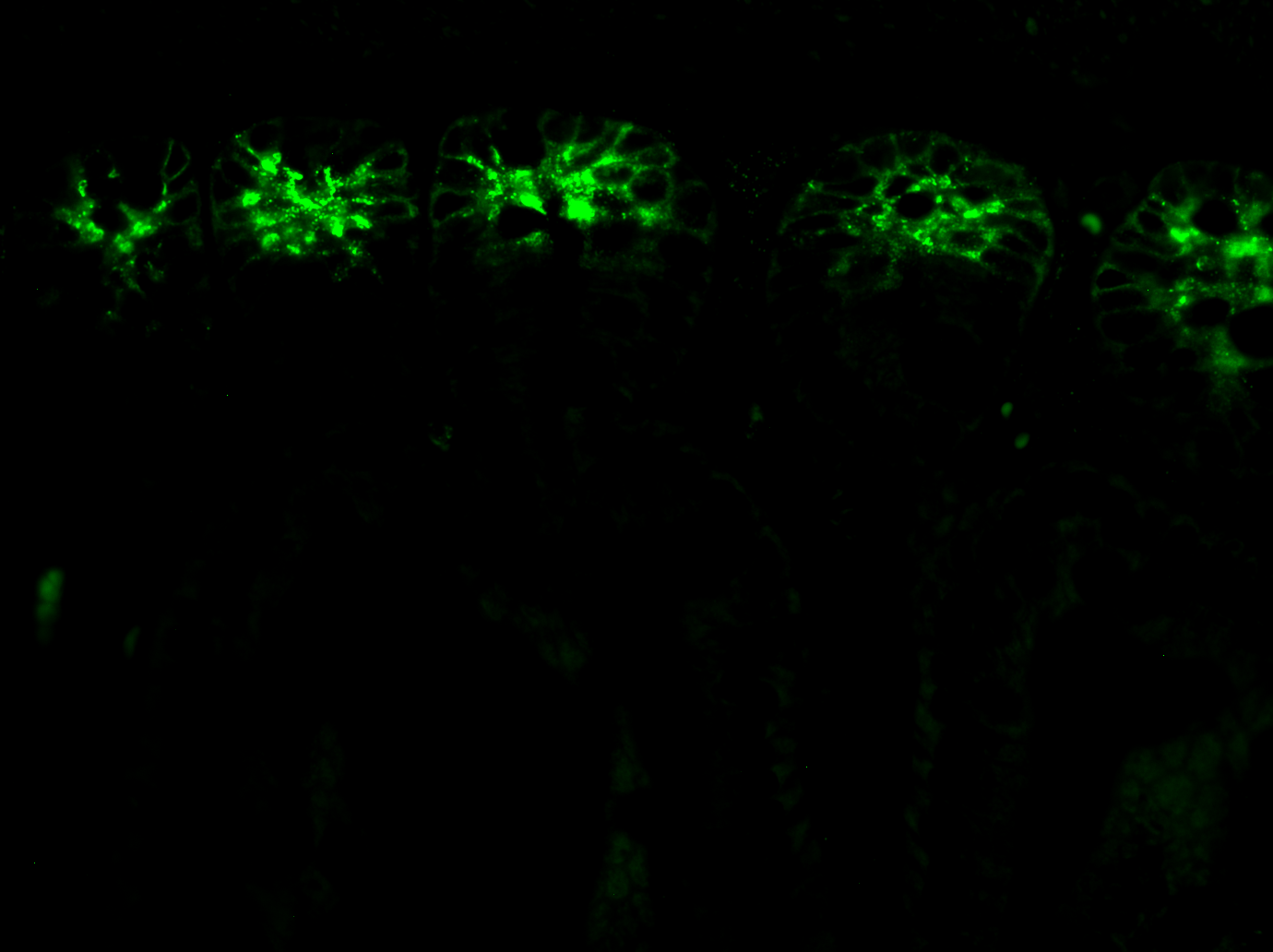

Supplement: Supplementary file 8 — Source Data for Figure 2 [file EMBR-24-e56030-s016.zip › Figure 2/Figure 2I-IHC-b-catenin OLFM4/1-3. Control, sham, OLFM4.TIF]

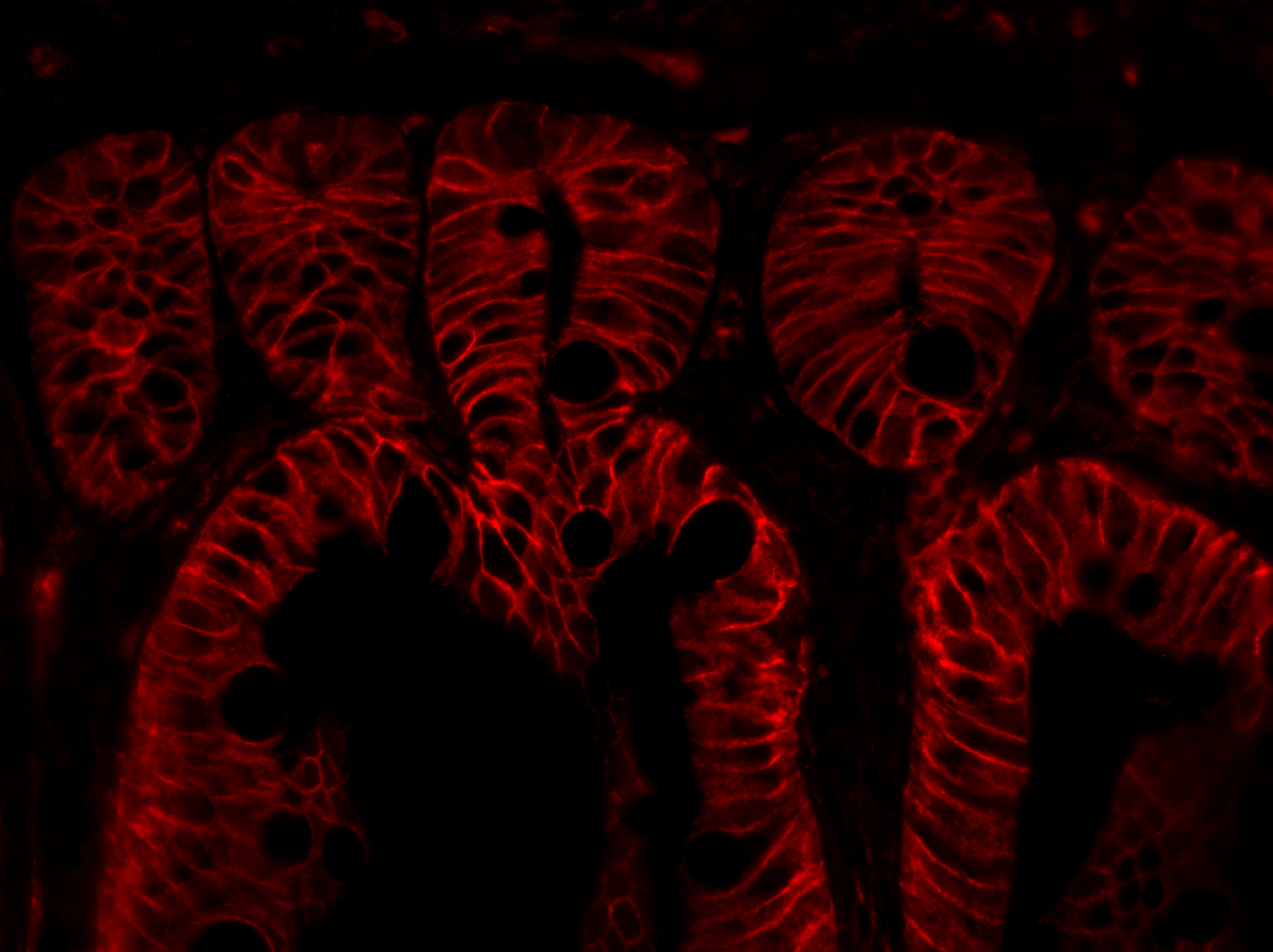

Supplement: Supplementary file 8 — Source Data for Figure 2 [file EMBR-24-e56030-s016.zip › Figure 2/Figure 2I-IHC-b-catenin OLFM4/1-4. Control, sham, b-catenin.TIF]

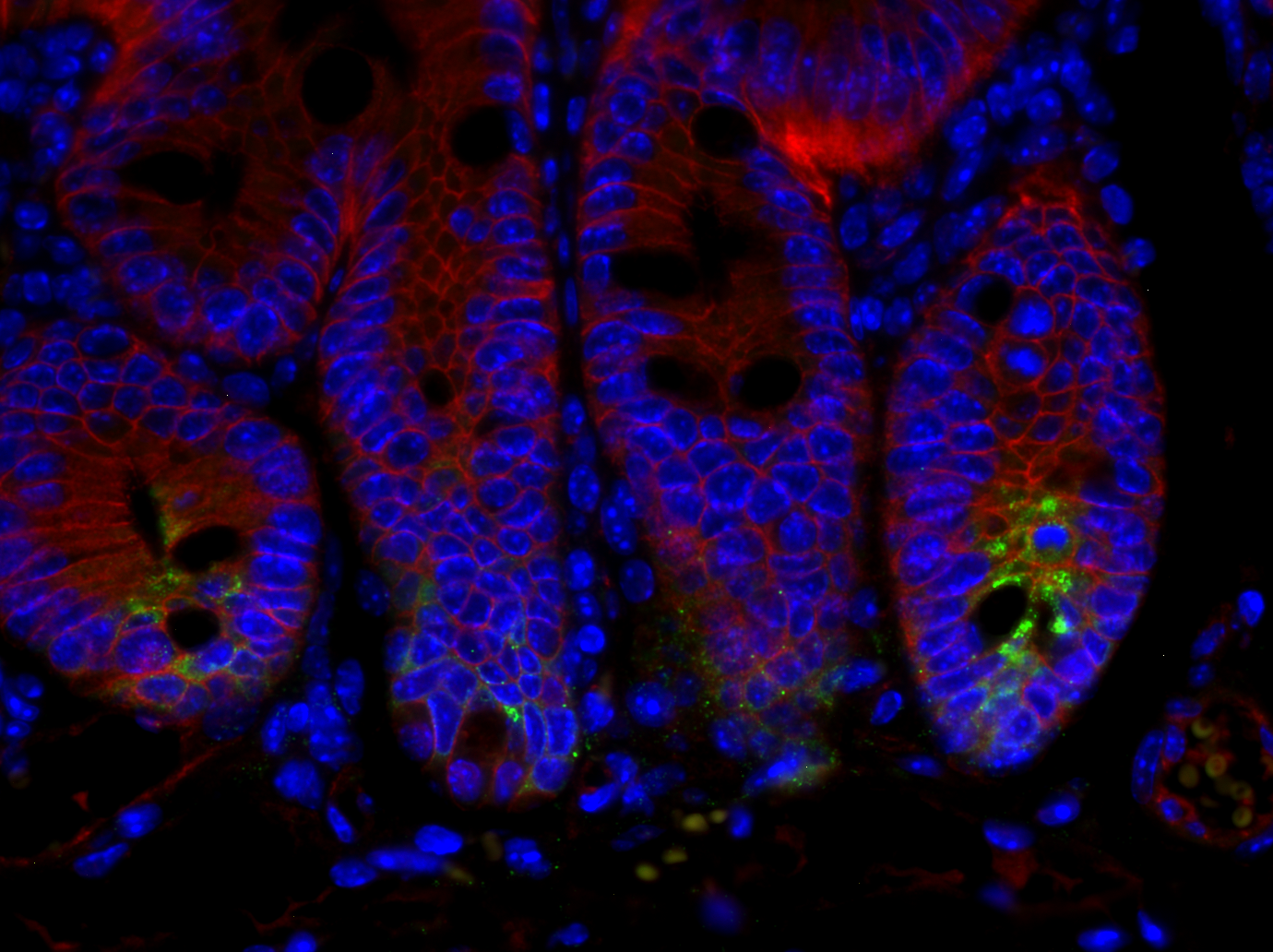

Supplement: Supplementary file 8 — Source Data for Figure 2 [file EMBR-24-e56030-s016.zip › Figure 2/Figure 2I-IHC-b-catenin OLFM4/2-1. EC-Foxc-DKO, sham, 3 colors.TIF]

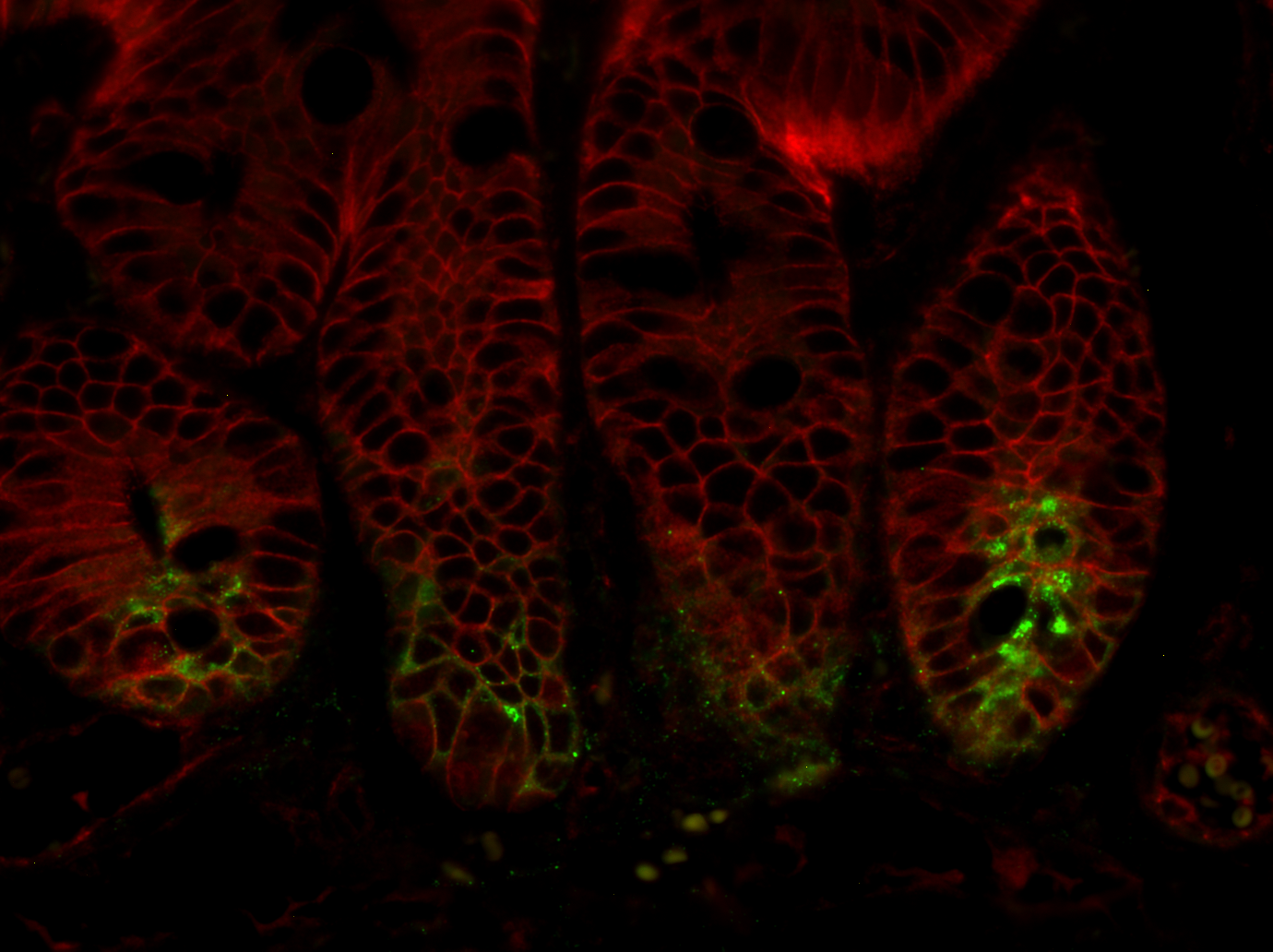

Supplement: Supplementary file 8 — Source Data for Figure 2 [file EMBR-24-e56030-s016.zip › Figure 2/Figure 2I-IHC-b-catenin OLFM4/2-2. EC-Foxc-DKO, sham, 2 colors.TIF]

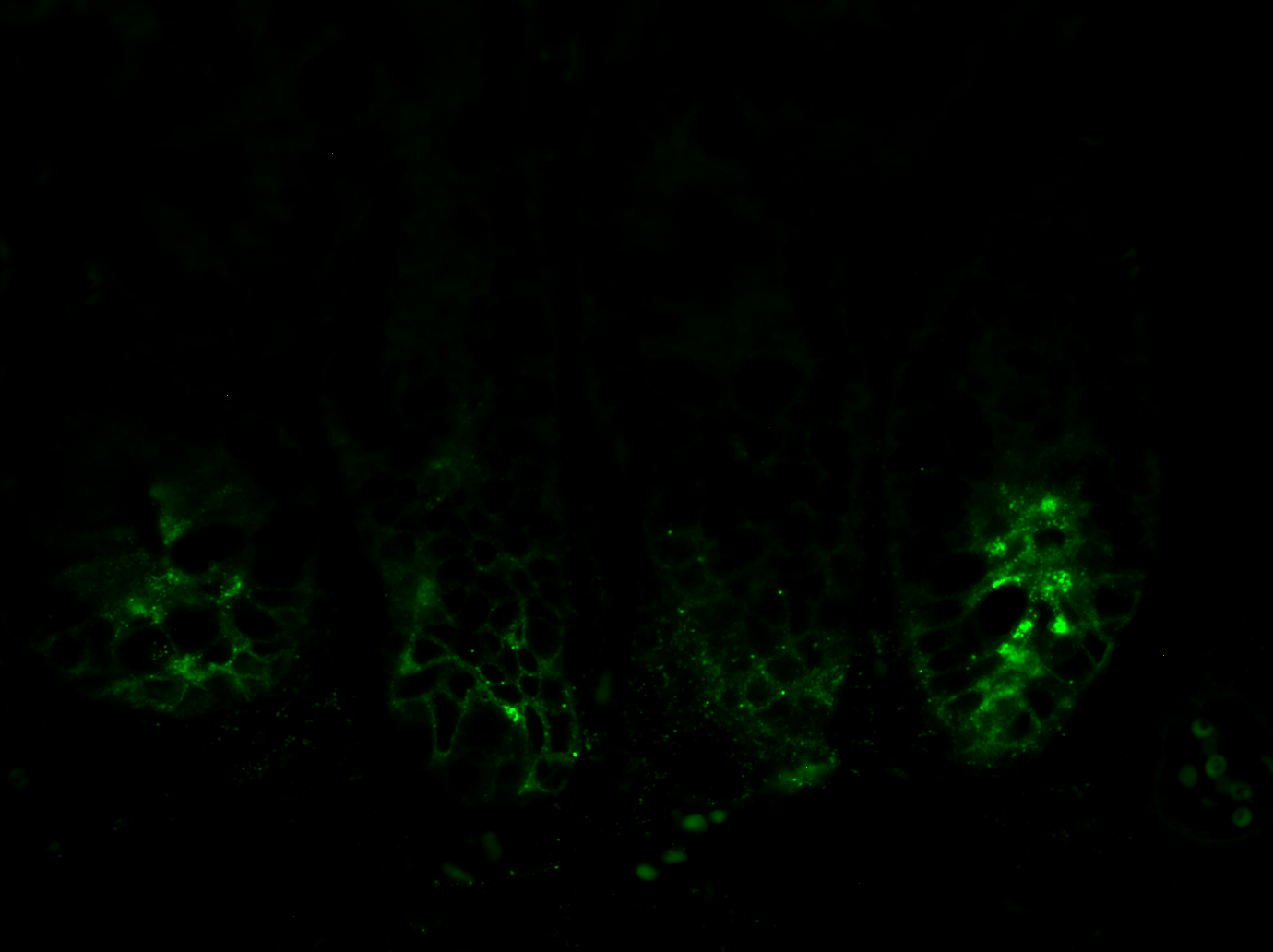

Supplement: Supplementary file 8 — Source Data for Figure 2 [file EMBR-24-e56030-s016.zip › Figure 2/Figure 2I-IHC-b-catenin OLFM4/2-3. EC-Foxc-DKO, sham, OLFM4.TIF]

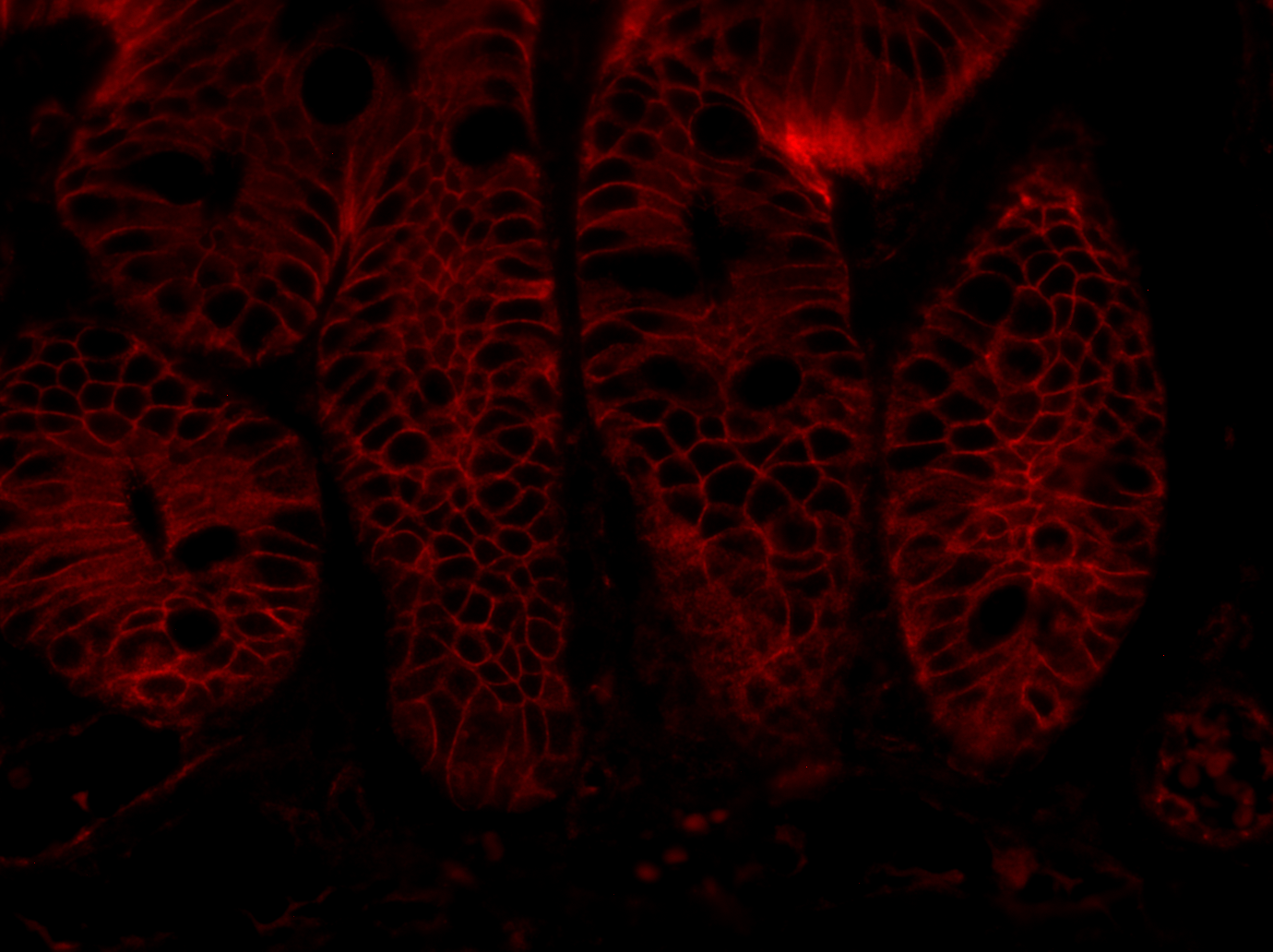

Supplement: Supplementary file 8 — Source Data for Figure 2 [file EMBR-24-e56030-s016.zip › Figure 2/Figure 2I-IHC-b-catenin OLFM4/2-4. EC-Foxc-DKO, sham, b-catenin.TIF]

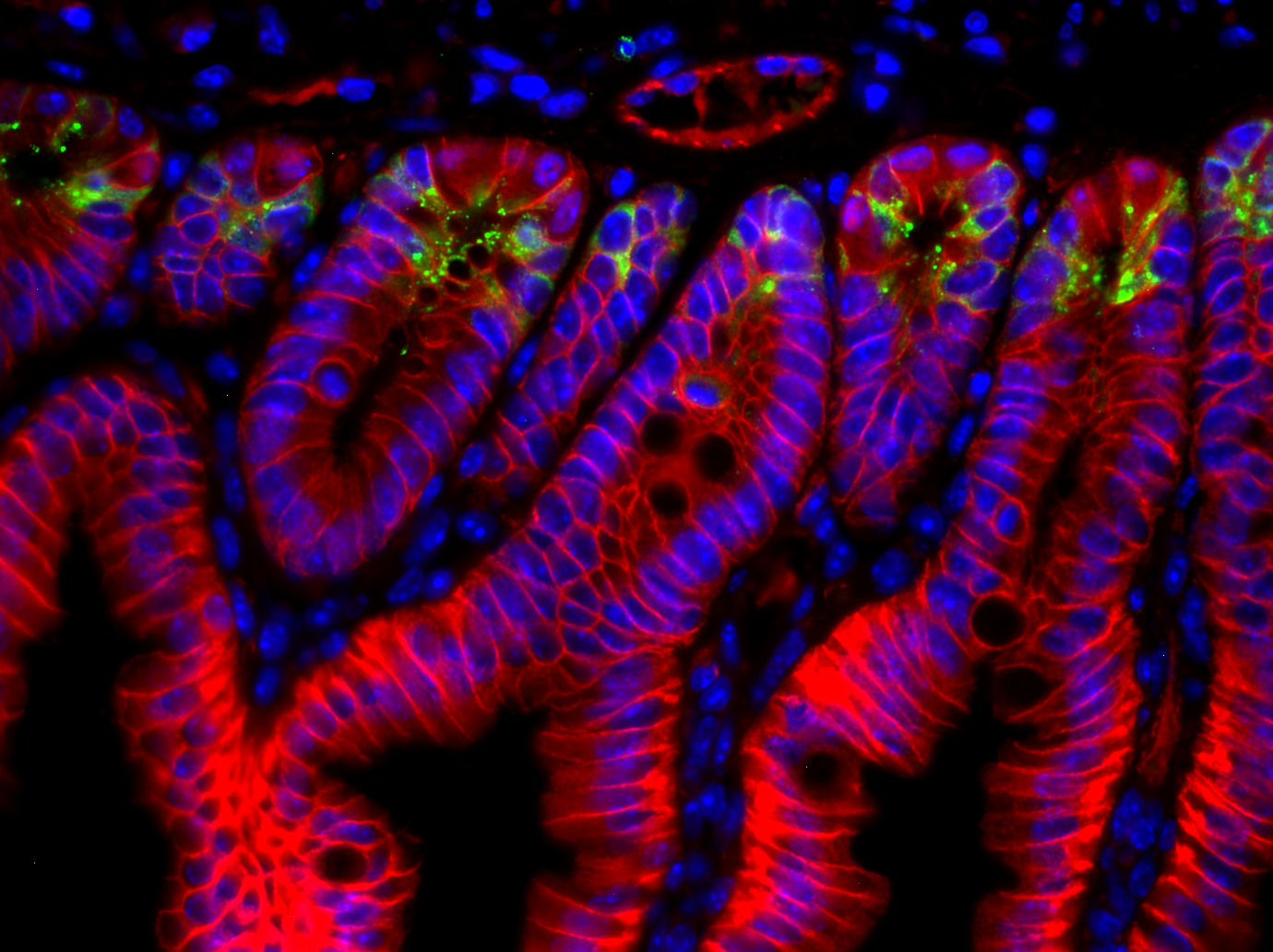

Supplement: Supplementary file 8 — Source Data for Figure 2 [file EMBR-24-e56030-s016.zip › Figure 2/Figure 2I-IHC-b-catenin OLFM4/3-1. Control, IR, 3 colors.TIF]

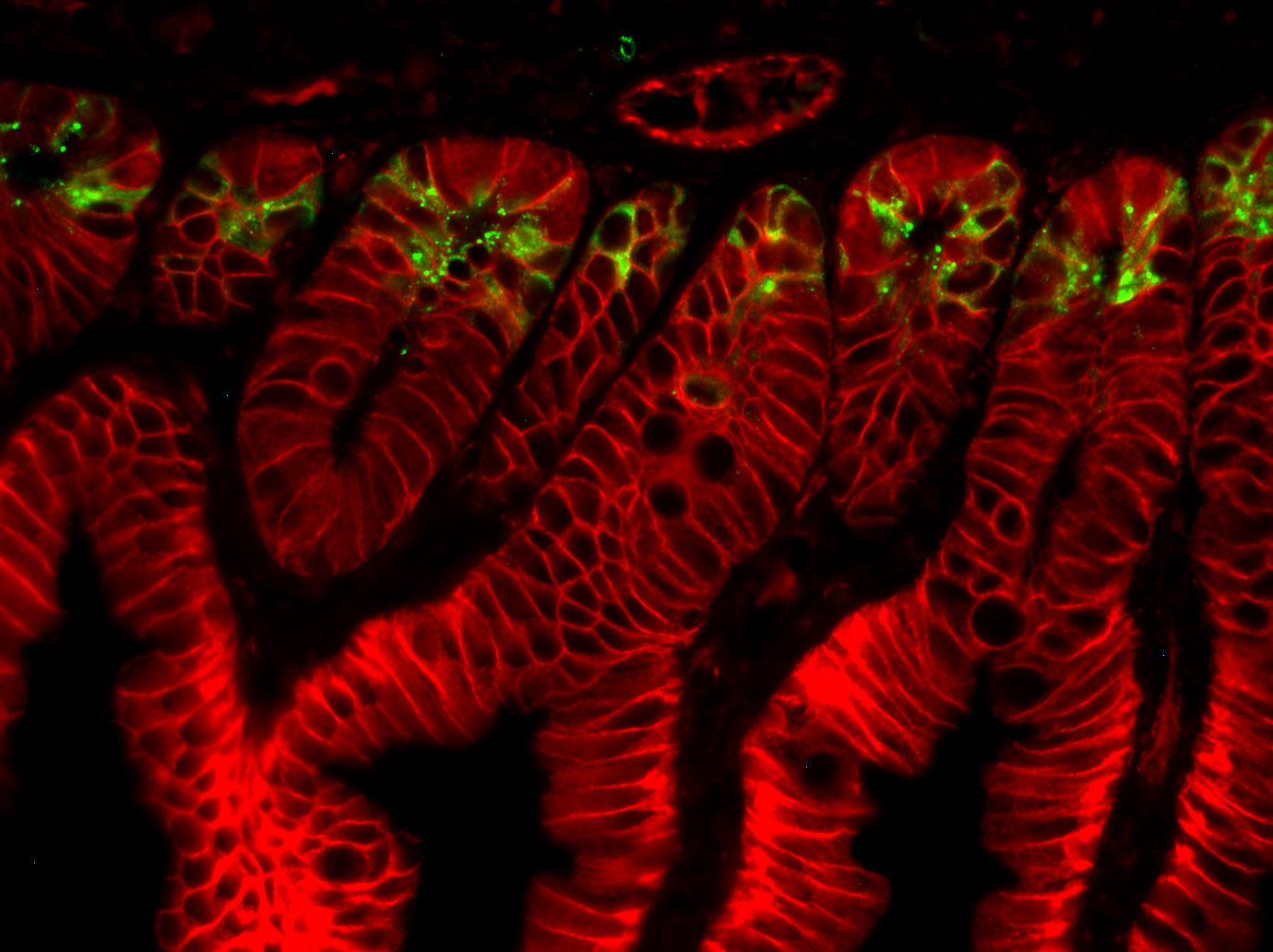

Supplement: Supplementary file 8 — Source Data for Figure 2 [file EMBR-24-e56030-s016.zip › Figure 2/Figure 2I-IHC-b-catenin OLFM4/3-2. Control, IR, 2 colors.TIF]

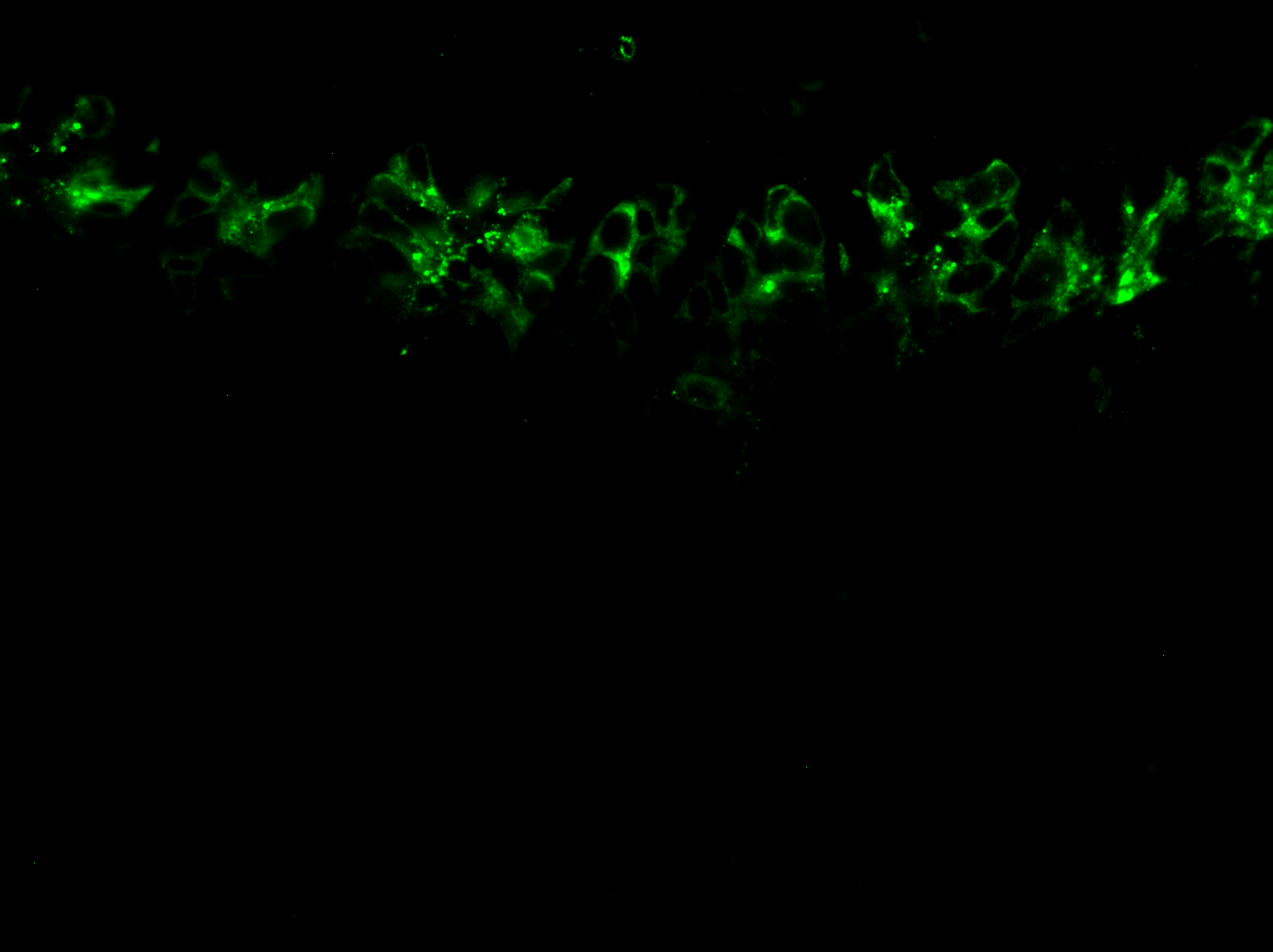

Supplement: Supplementary file 8 — Source Data for Figure 2 [file EMBR-24-e56030-s016.zip › Figure 2/Figure 2I-IHC-b-catenin OLFM4/3-3. Control, IR, OLFM4.TIF]

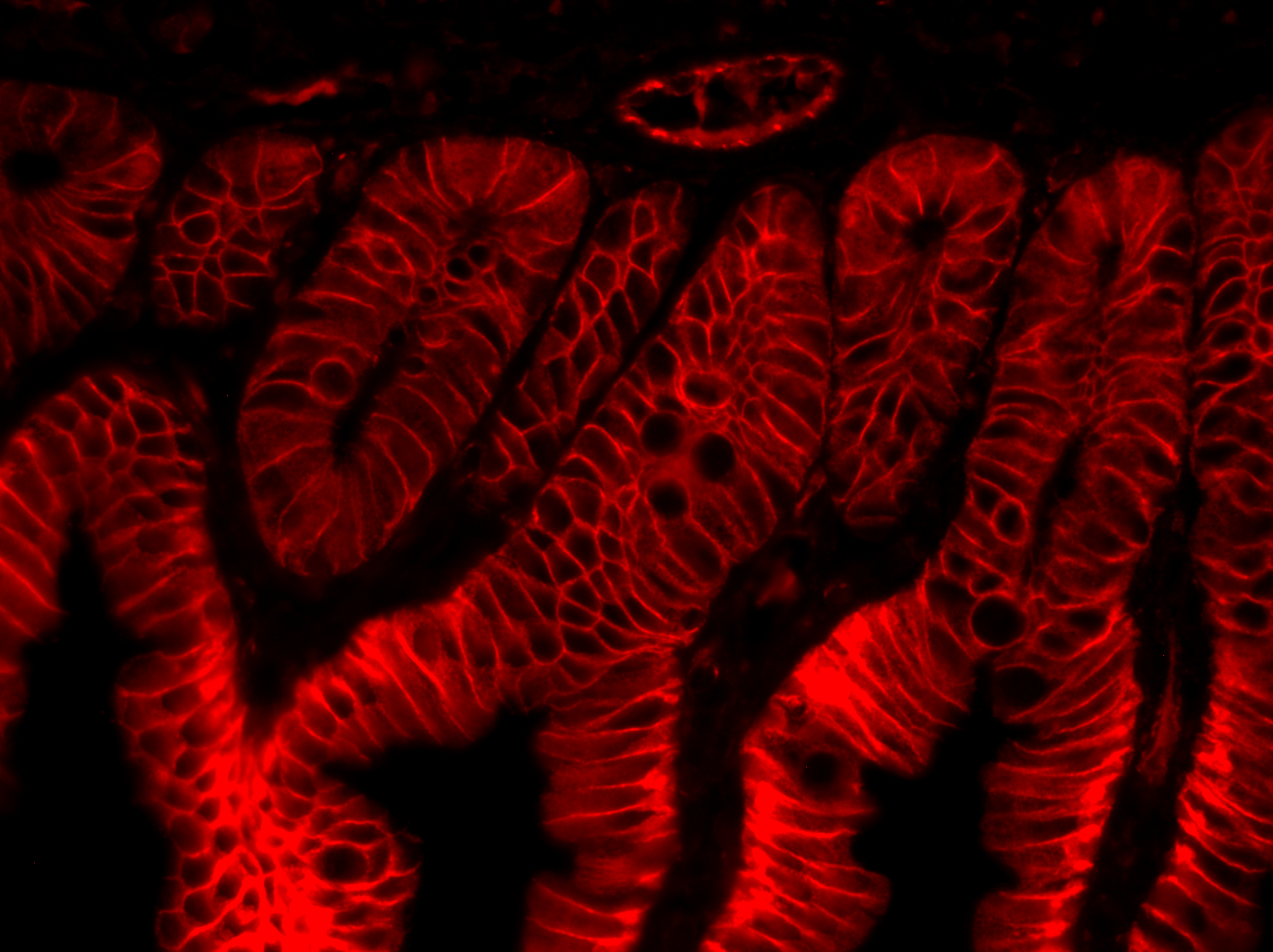

Supplement: Supplementary file 8 — Source Data for Figure 2 [file EMBR-24-e56030-s016.zip › Figure 2/Figure 2I-IHC-b-catenin OLFM4/3-4. Control, IR, b-catenin.TIF]

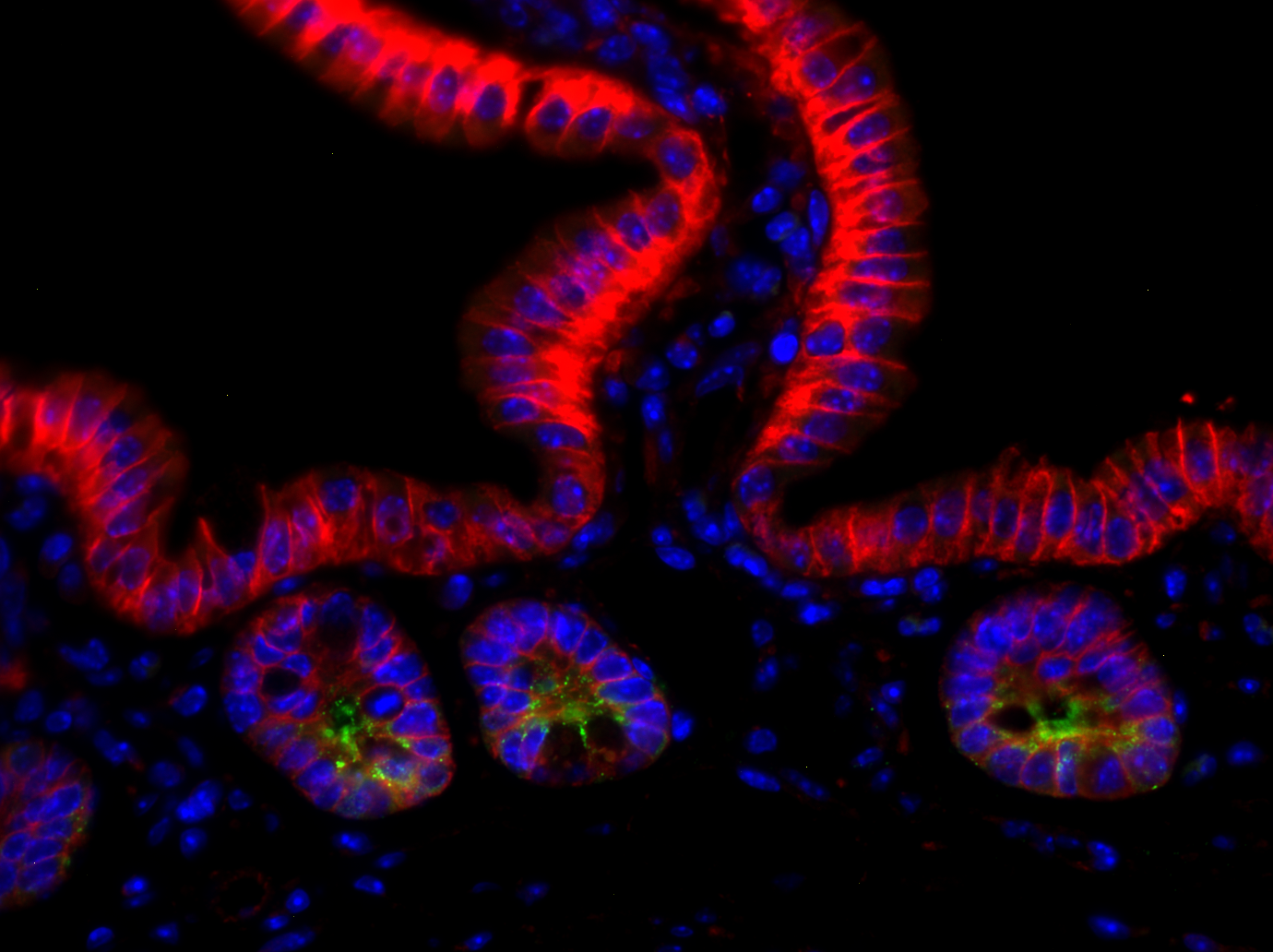

Supplement: Supplementary file 8 — Source Data for Figure 2 [file EMBR-24-e56030-s016.zip › Figure 2/Figure 2I-IHC-b-catenin OLFM4/4-1. EC-Foxc-DKO, IR, 3 colors.TIF]

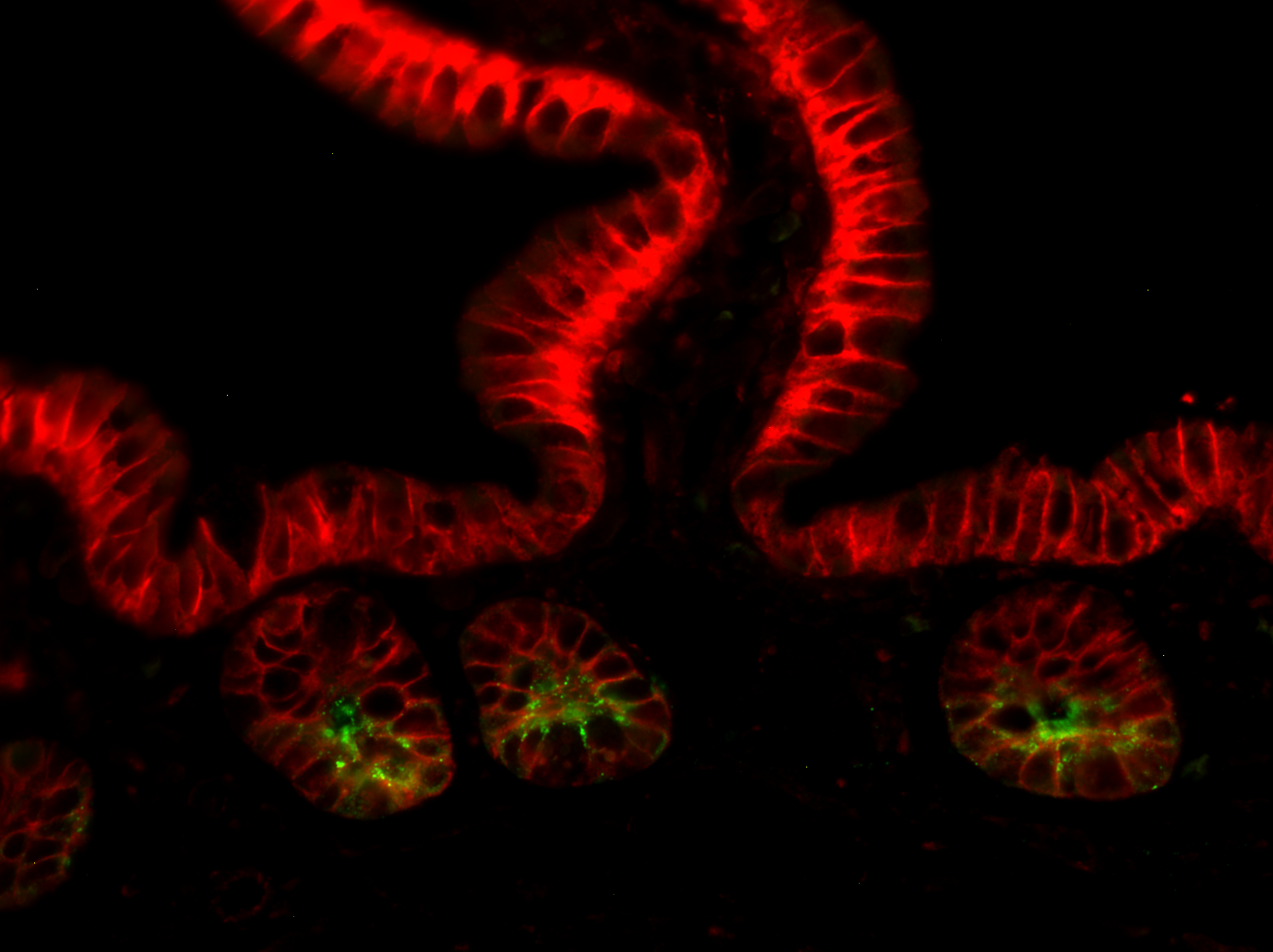

Supplement: Supplementary file 8 — Source Data for Figure 2 [file EMBR-24-e56030-s016.zip › Figure 2/Figure 2I-IHC-b-catenin OLFM4/4-2, EC-Foxc-DKO, IR, 2 colors.TIF]

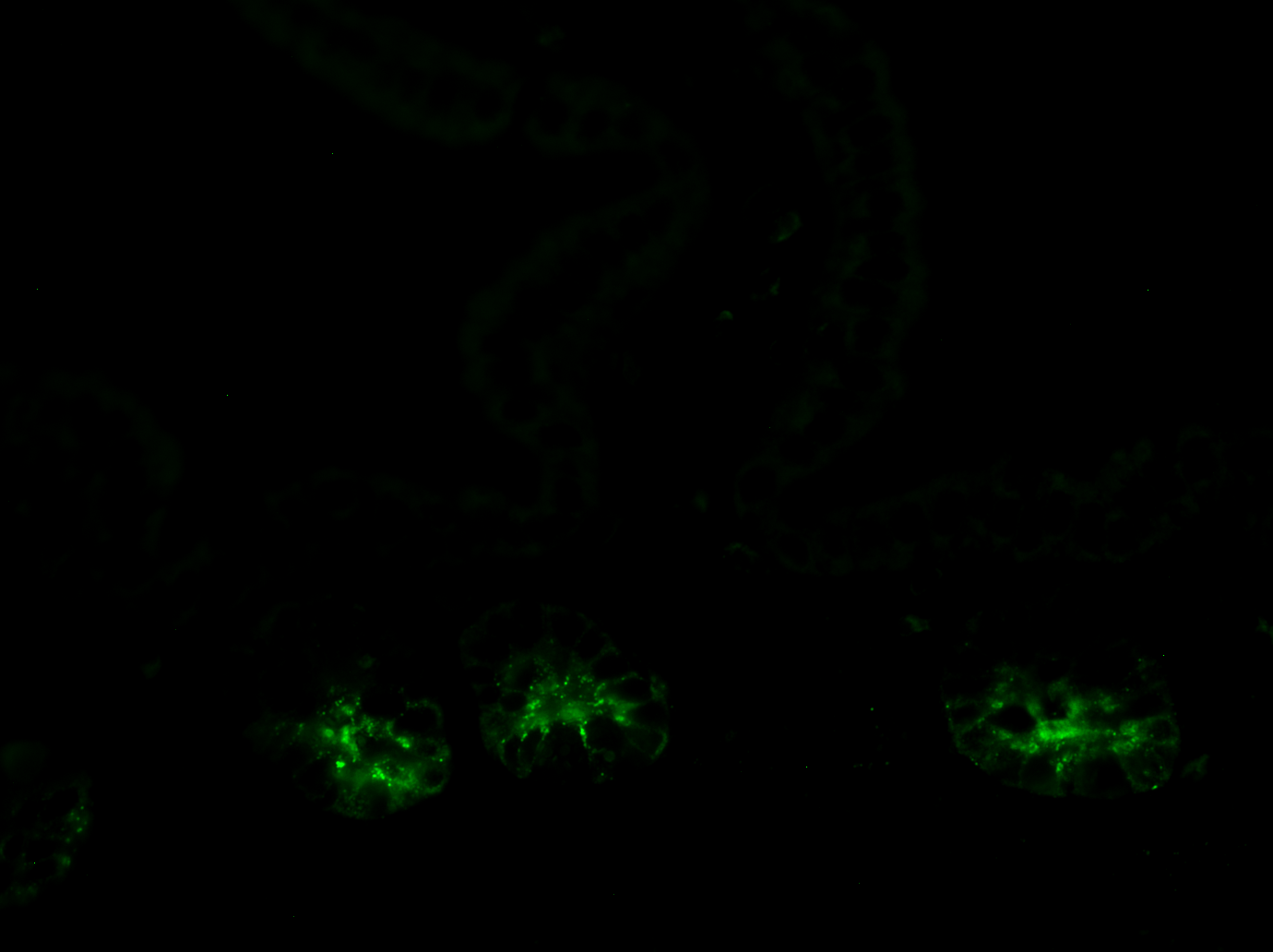

Supplement: Supplementary file 8 — Source Data for Figure 2 [file EMBR-24-e56030-s016.zip › Figure 2/Figure 2I-IHC-b-catenin OLFM4/4-3. EC-Foxc-DKO, IR, OLFM4.TIF]

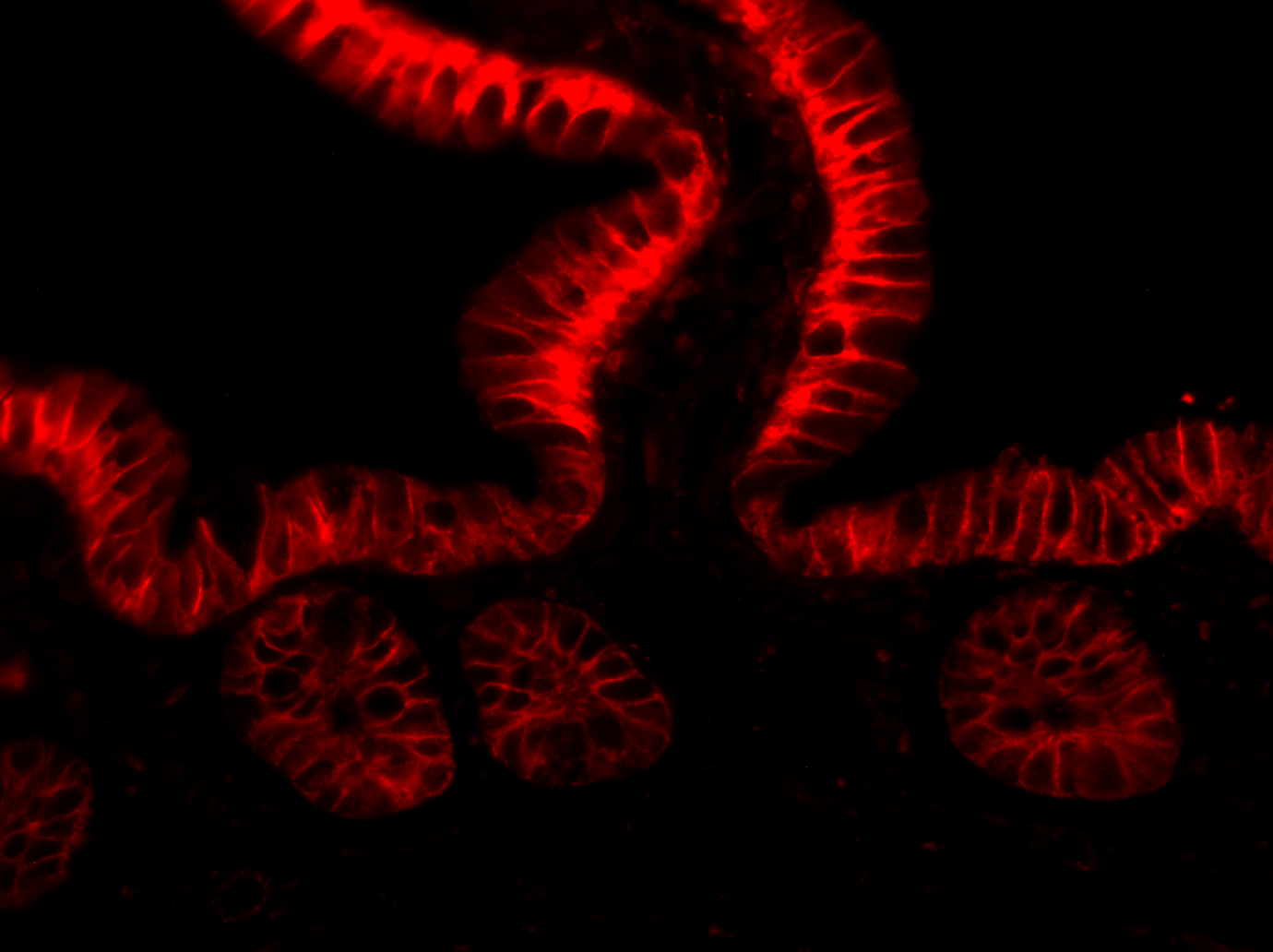

Supplement: Supplementary file 8 — Source Data for Figure 2 [file EMBR-24-e56030-s016.zip › Figure 2/Figure 2I-IHC-b-catenin OLFM4/4-4. EC-Foxc-DKO, IR, b-catenin.TIF]

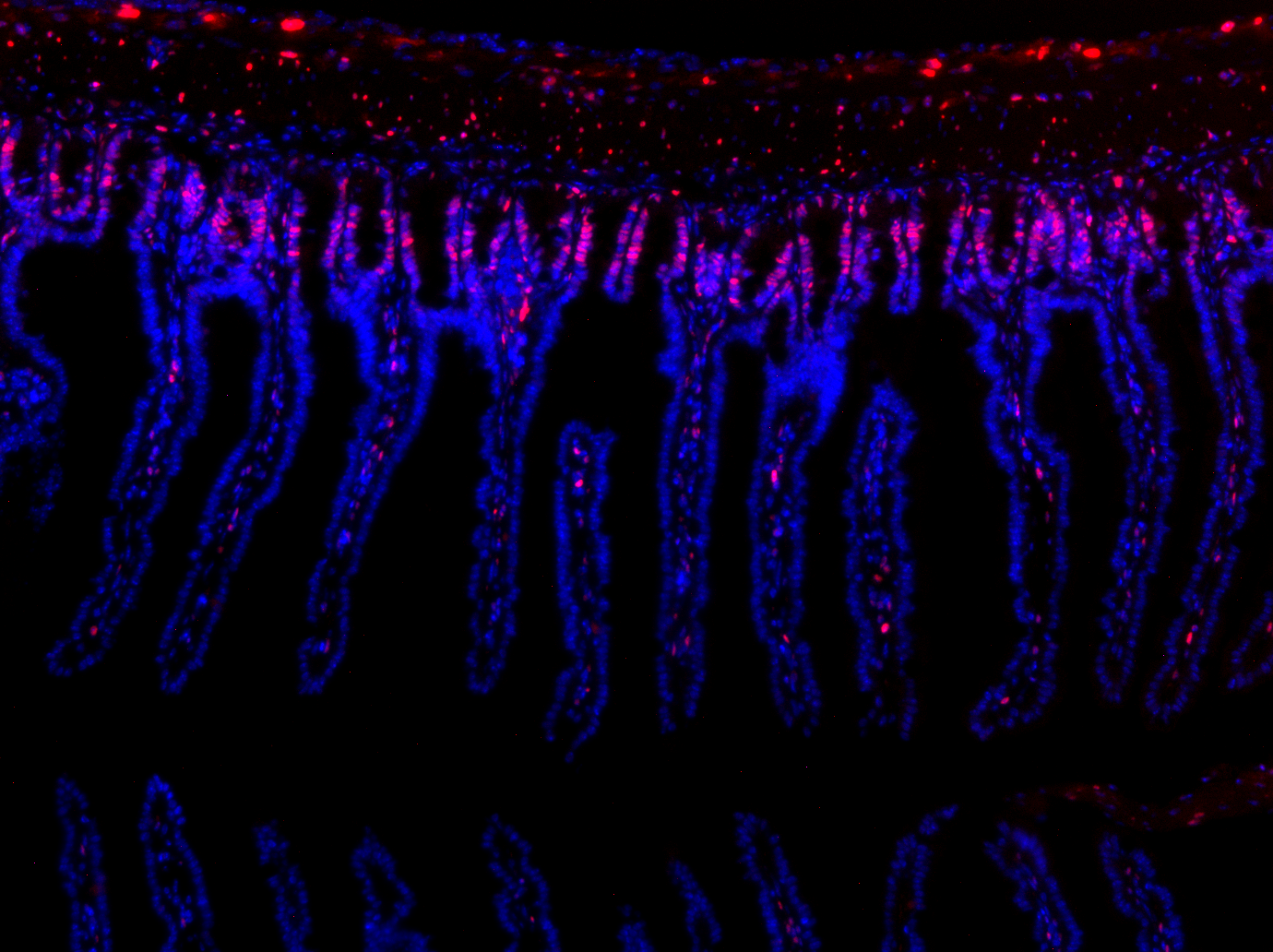

Supplement: Supplementary file 8 — Source Data for Figure 2 [file EMBR-24-e56030-s016.zip › Figure 2/Figure 2L-IHC-CCND1/1. Control sham.TIF]

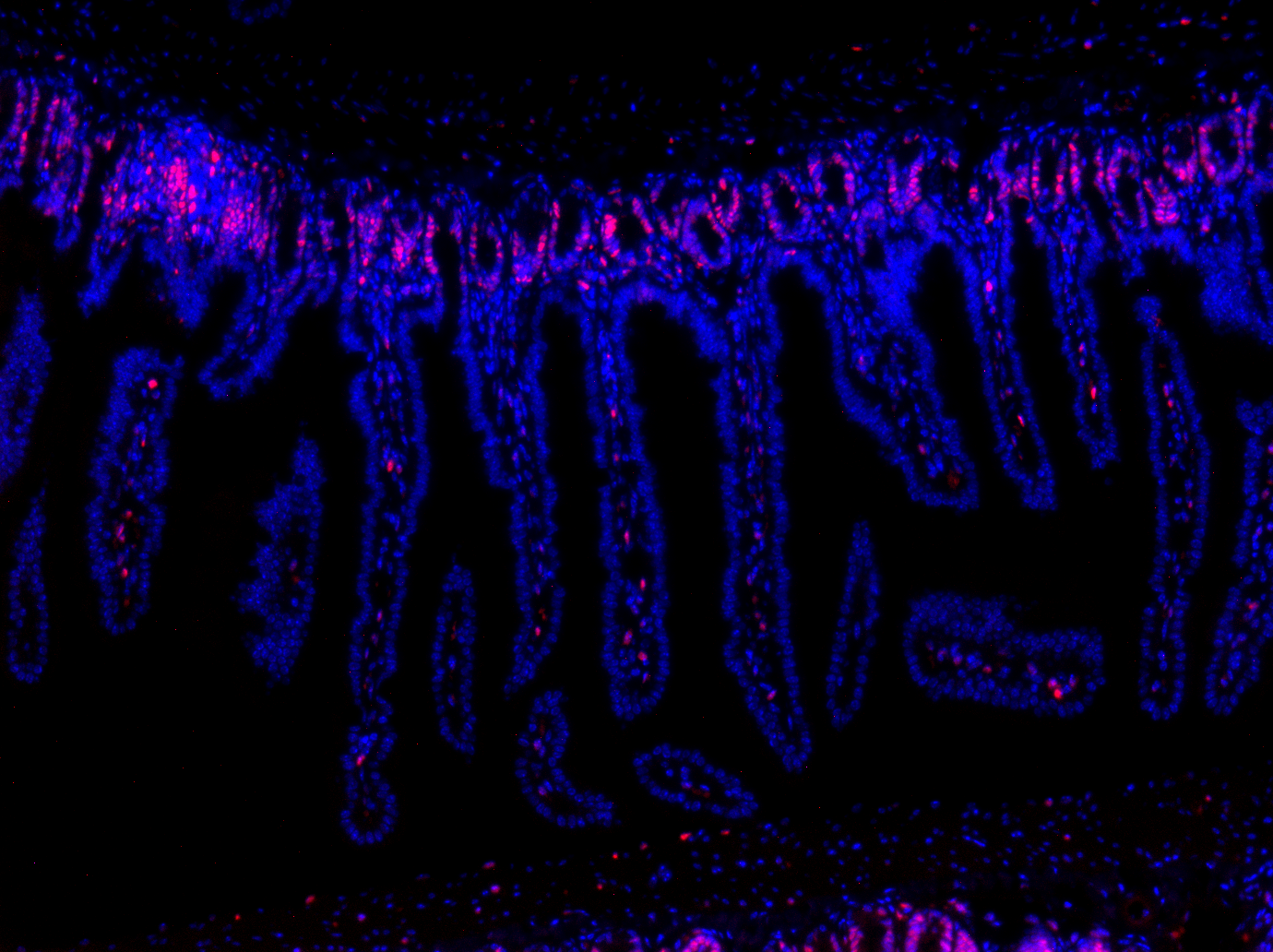

Supplement: Supplementary file 8 — Source Data for Figure 2 [file EMBR-24-e56030-s016.zip › Figure 2/Figure 2L-IHC-CCND1/2. EC-Foxc-DKO, sham.TIF]

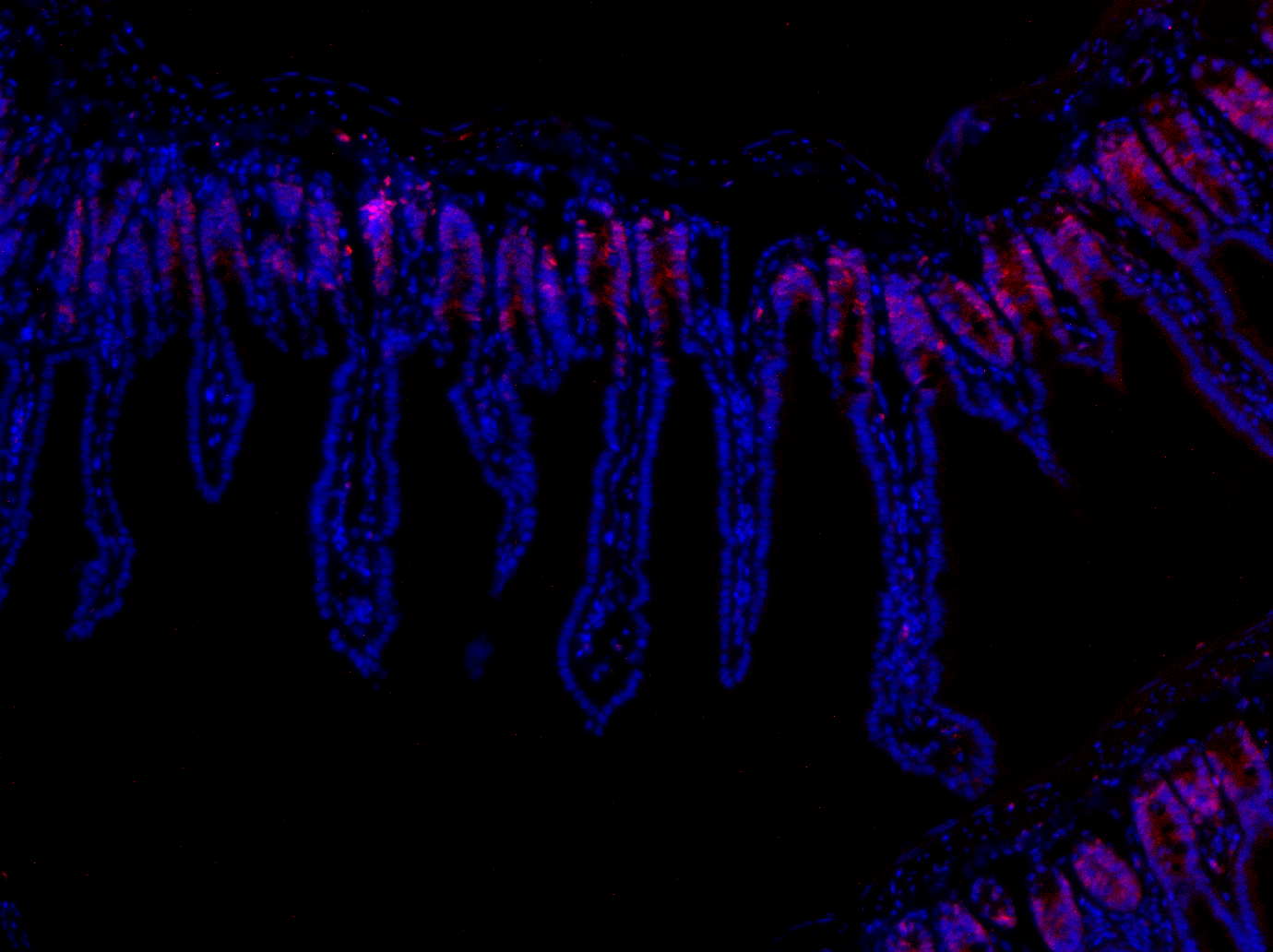

Supplement: Supplementary file 8 — Source Data for Figure 2 [file EMBR-24-e56030-s016.zip › Figure 2/Figure 2L-IHC-CCND1/3. Control, IR-24h.TIF]

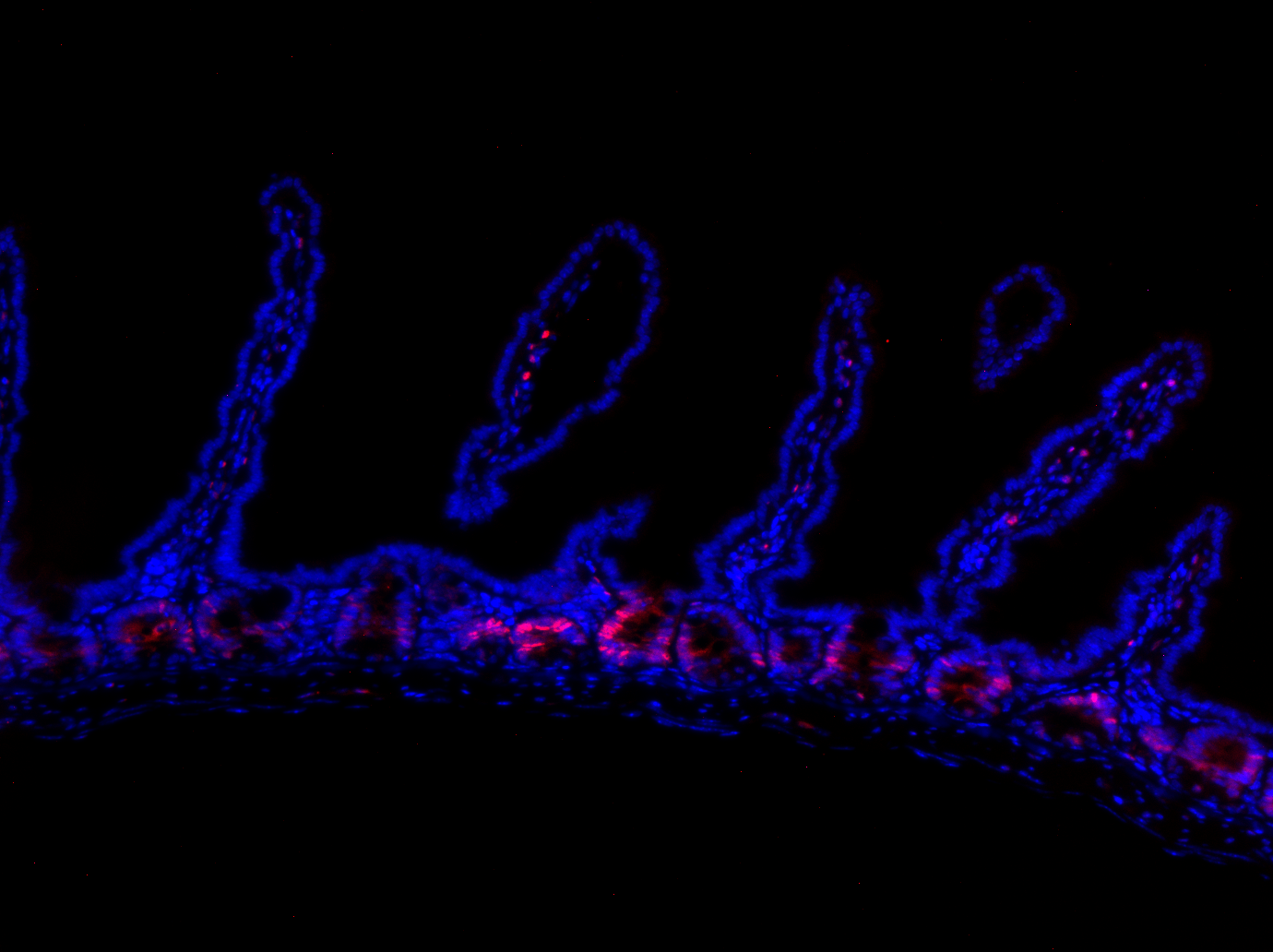

Supplement: Supplementary file 8 — Source Data for Figure 2 [file EMBR-24-e56030-s016.zip › Figure 2/Figure 2L-IHC-CCND1/4. EC-Foxc-DKO, IR-24h.TIF]

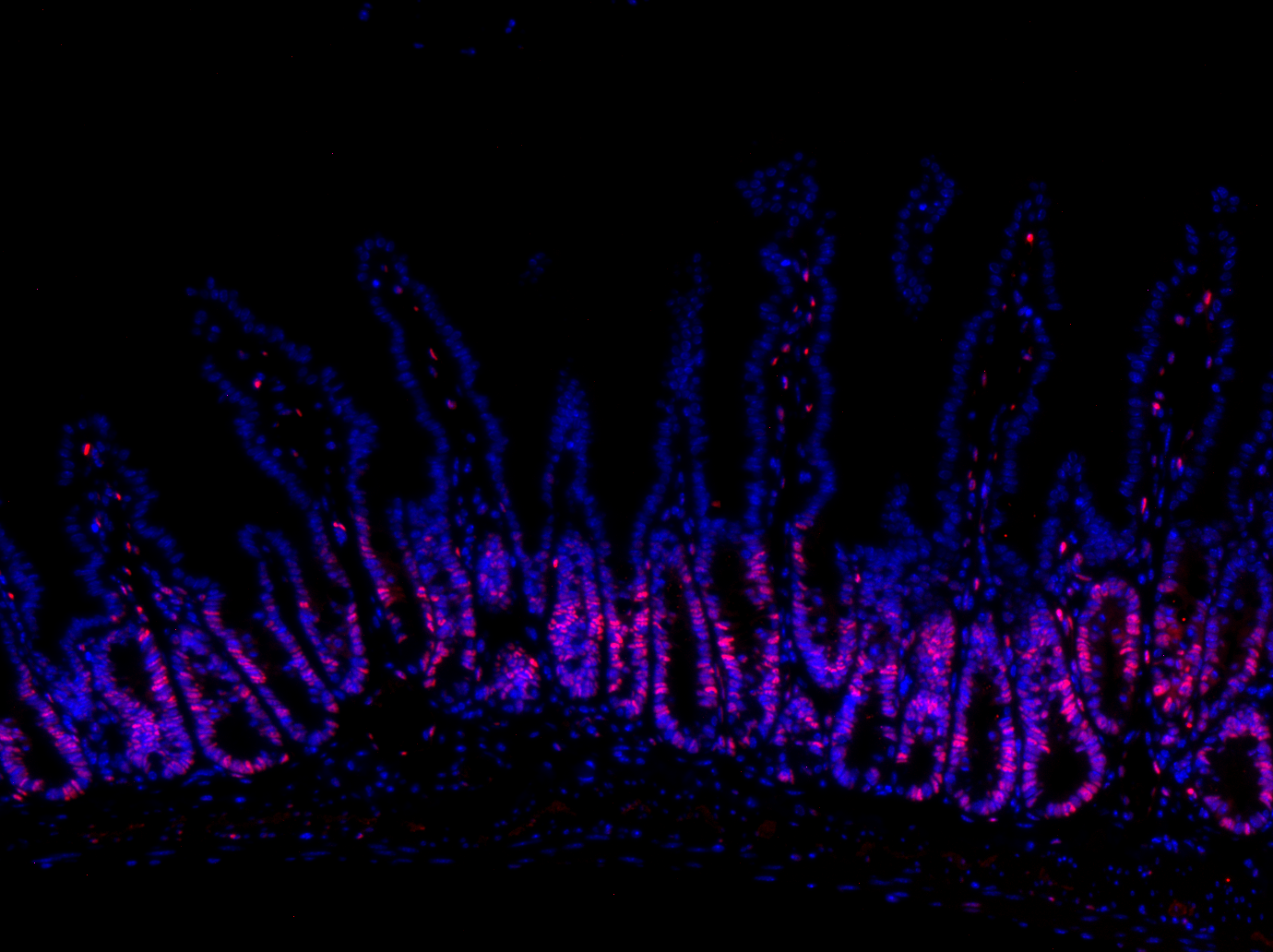

Supplement: Supplementary file 8 — Source Data for Figure 2 [file EMBR-24-e56030-s016.zip › Figure 2/Figure 2L-IHC-CCND1/5. Control, IR-48h.TIF]

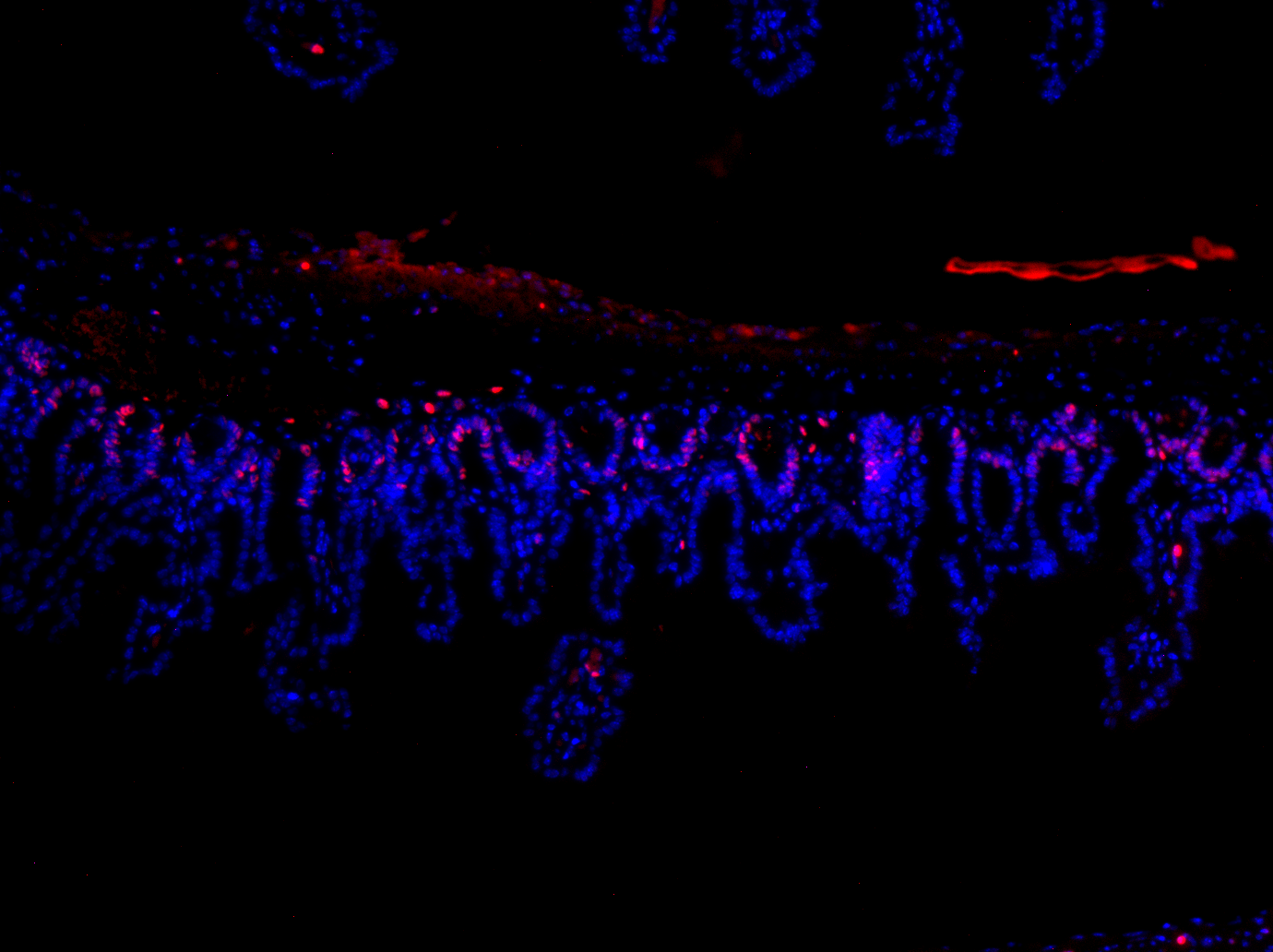

Supplement: Supplementary file 8 — Source Data for Figure 2 [file EMBR-24-e56030-s016.zip › Figure 2/Figure 2L-IHC-CCND1/6. EC-Foxc-DKO, IR-48h.TIF]

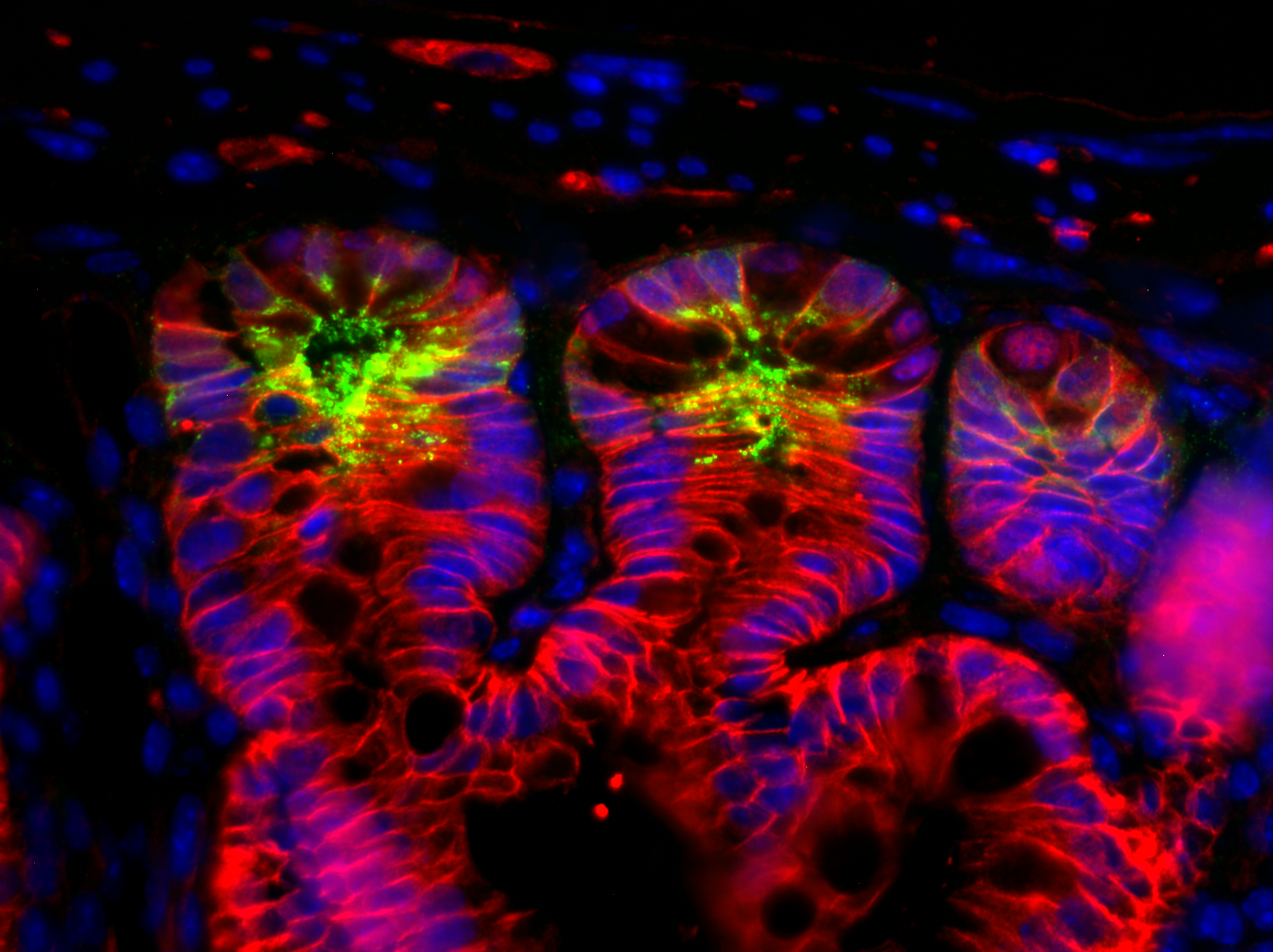

Supplement: Supplementary file 9 — Source Data for Figure 3 [file EMBR-24-e56030-s002.zip › Figure 3/Figure 3F-b-catenin OLFM4 staining/1. Control, 3 colors.TIF]

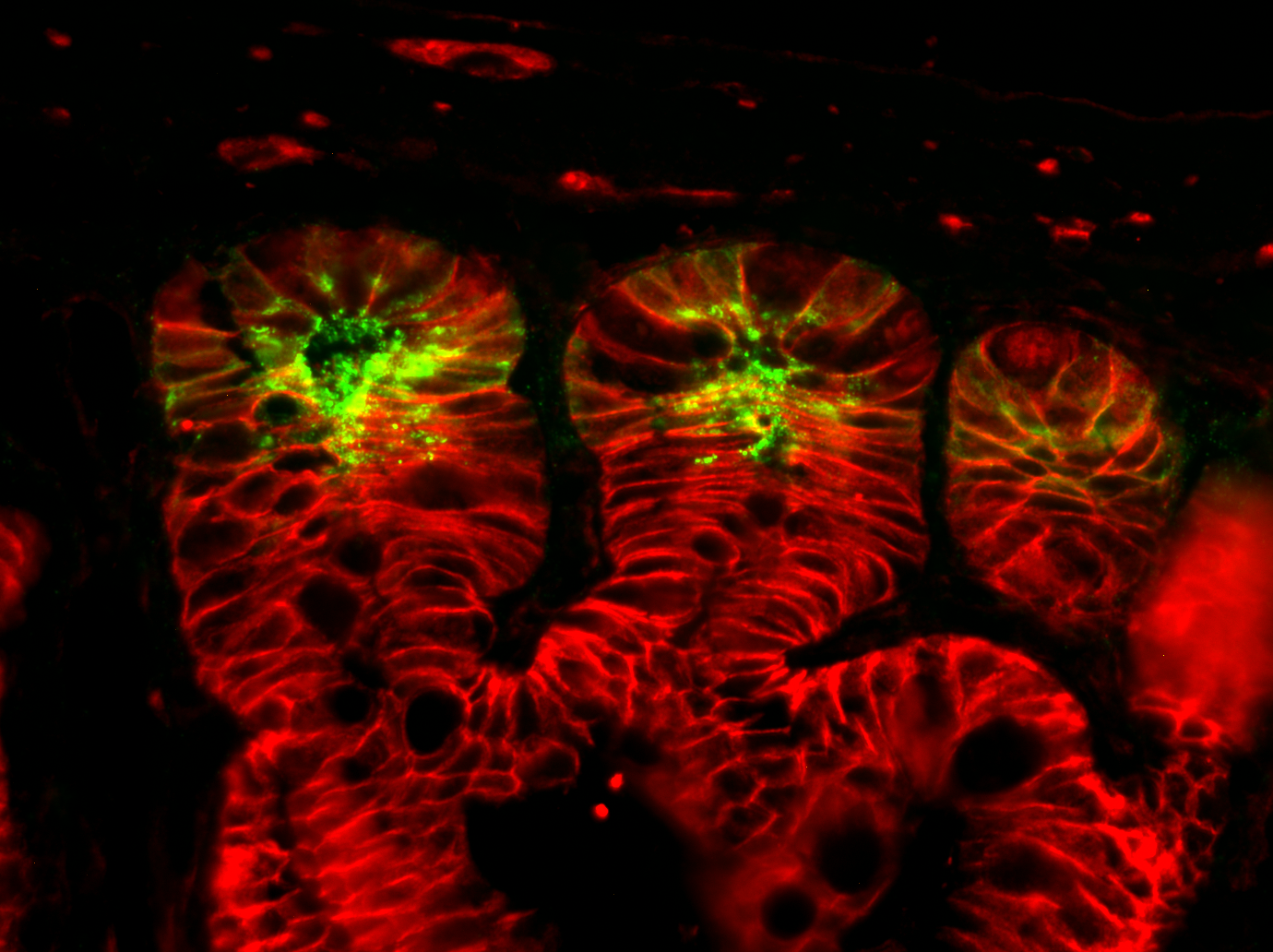

Supplement: Supplementary file 9 — Source Data for Figure 3 [file EMBR-24-e56030-s002.zip › Figure 3/Figure 3F-b-catenin OLFM4 staining/2. Control, 2 colors.TIF]

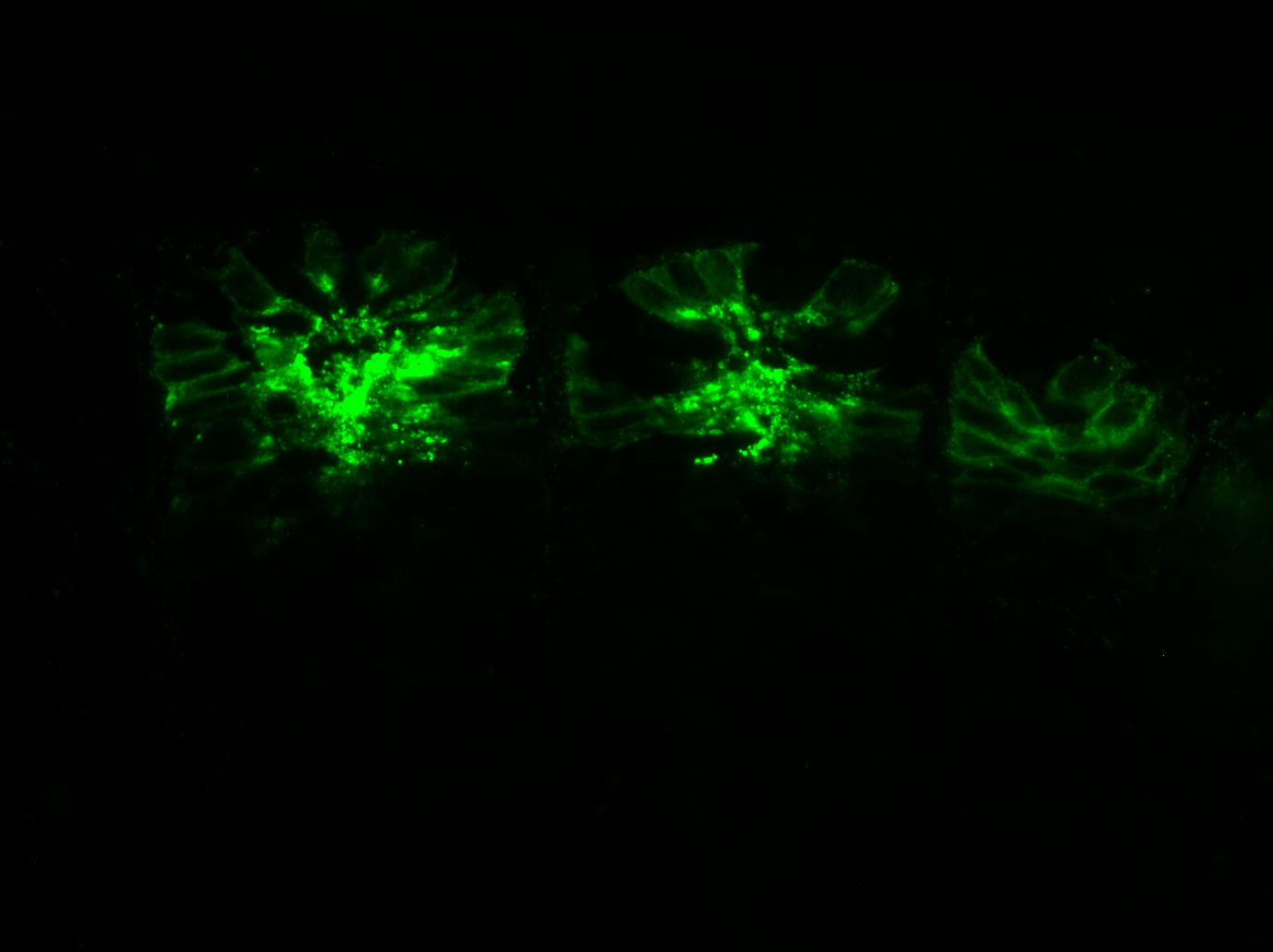

Supplement: Supplementary file 9 — Source Data for Figure 3 [file EMBR-24-e56030-s002.zip › Figure 3/Figure 3F-b-catenin OLFM4 staining/3. Control, OLFM4.TIF]

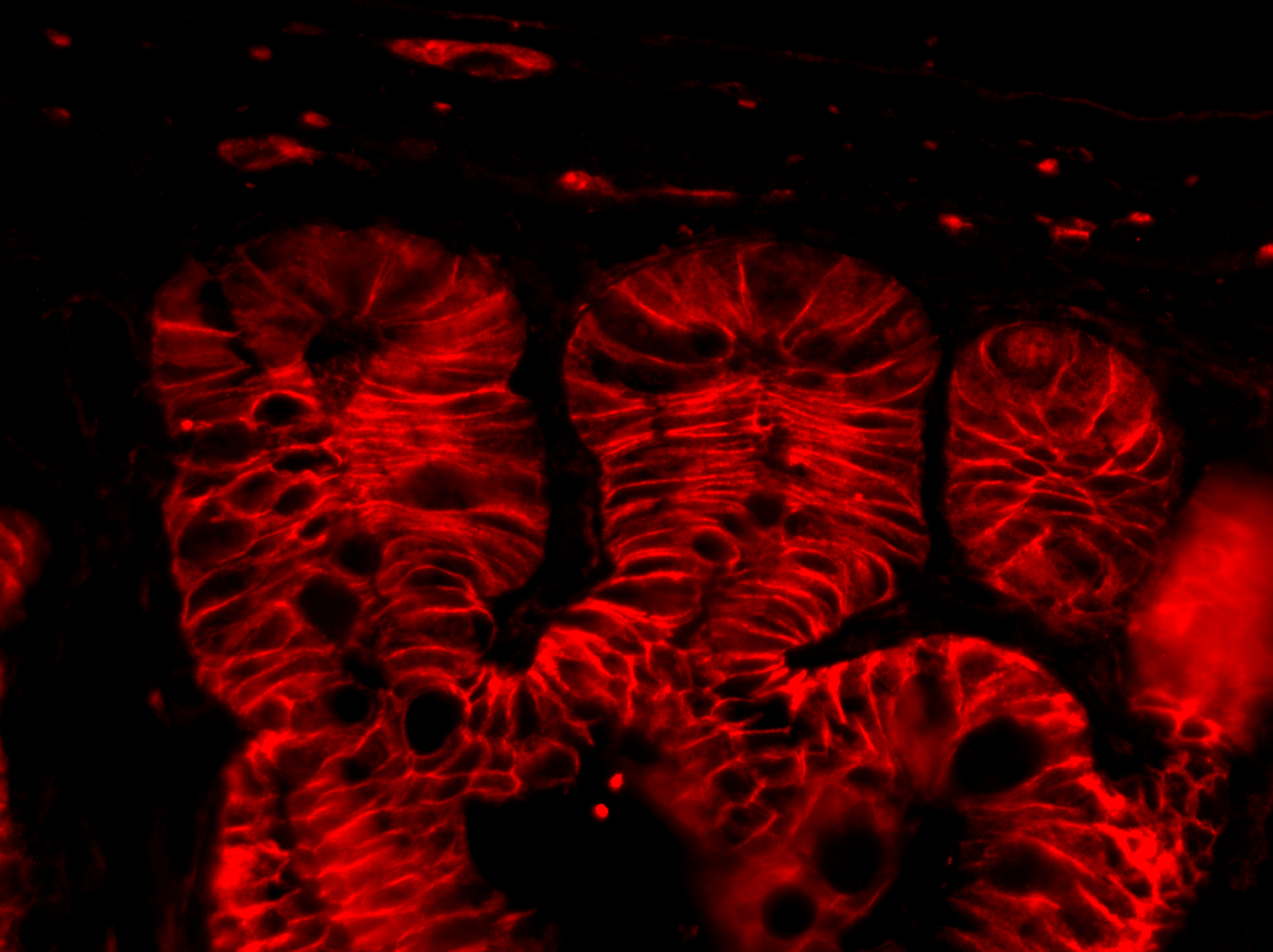

Supplement: Supplementary file 9 — Source Data for Figure 3 [file EMBR-24-e56030-s002.zip › Figure 3/Figure 3F-b-catenin OLFM4 staining/4. Control, b-catenin.TIF]

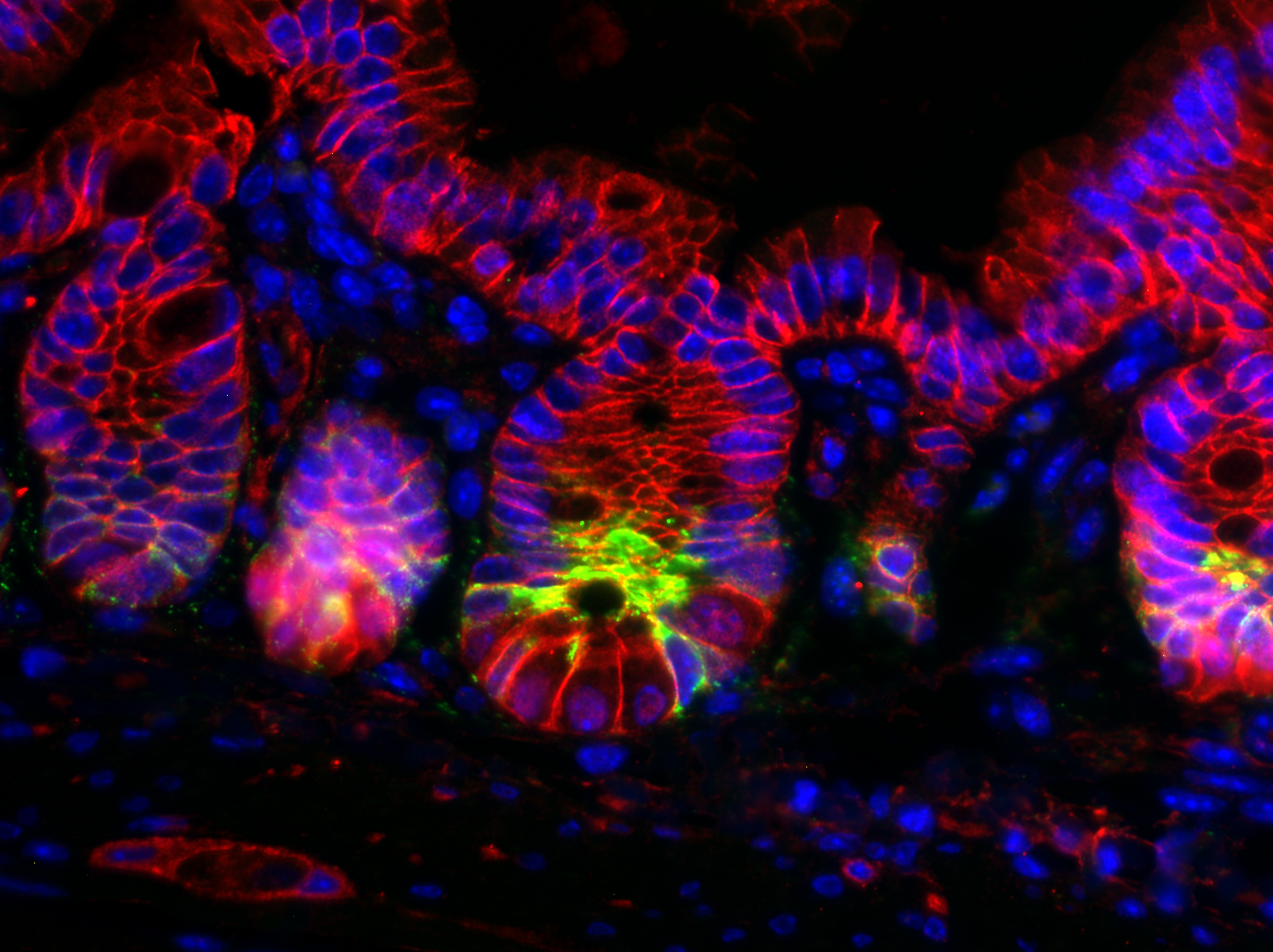

Supplement: Supplementary file 9 — Source Data for Figure 3 [file EMBR-24-e56030-s002.zip › Figure 3/Figure 3F-b-catenin OLFM4 staining/5. LEC-Foxc-DKO, 3 colors.TIF]

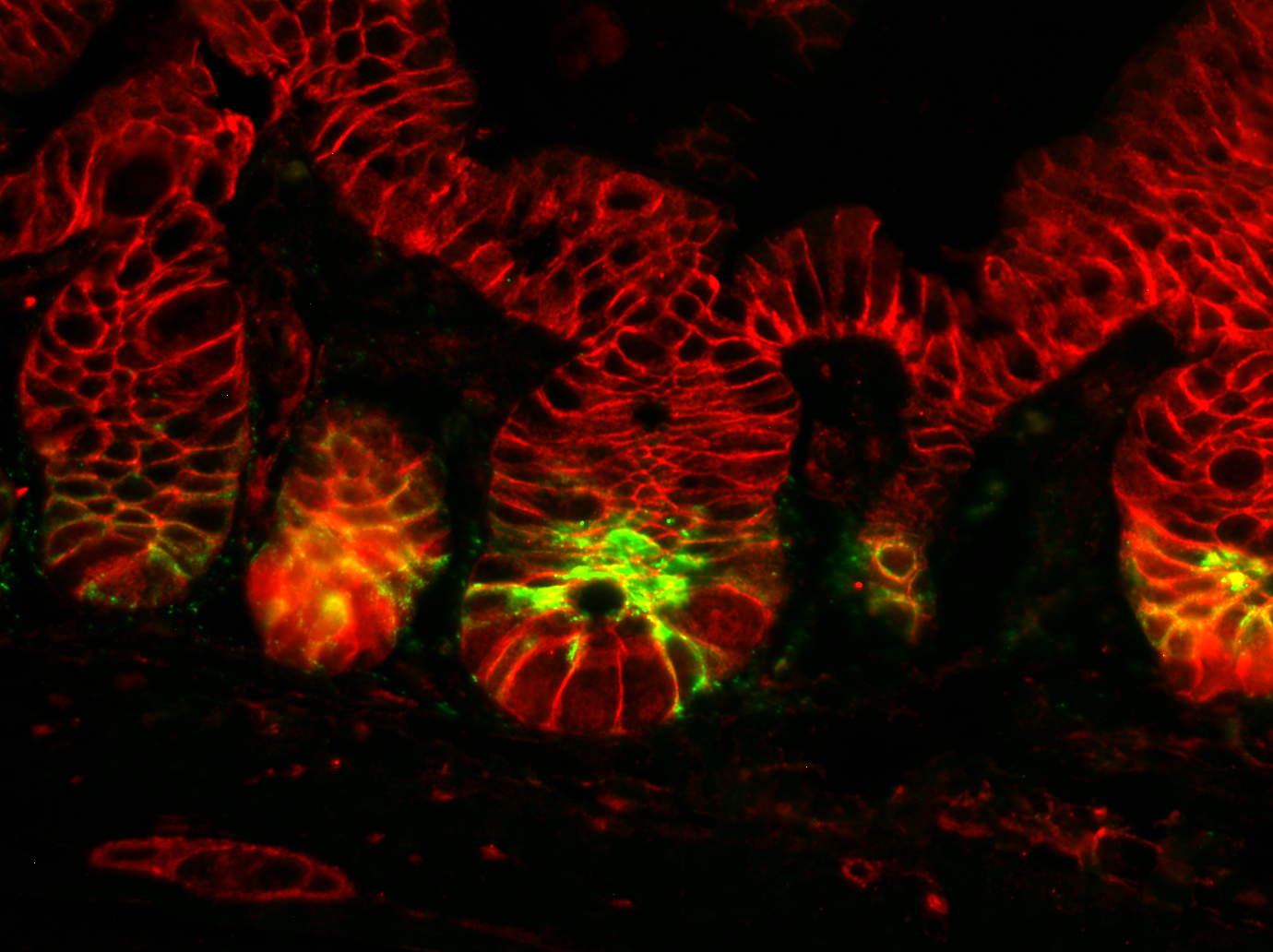

Supplement: Supplementary file 9 — Source Data for Figure 3 [file EMBR-24-e56030-s002.zip › Figure 3/Figure 3F-b-catenin OLFM4 staining/6. LEC-Foxc-DKO, 2 colors.TIF]

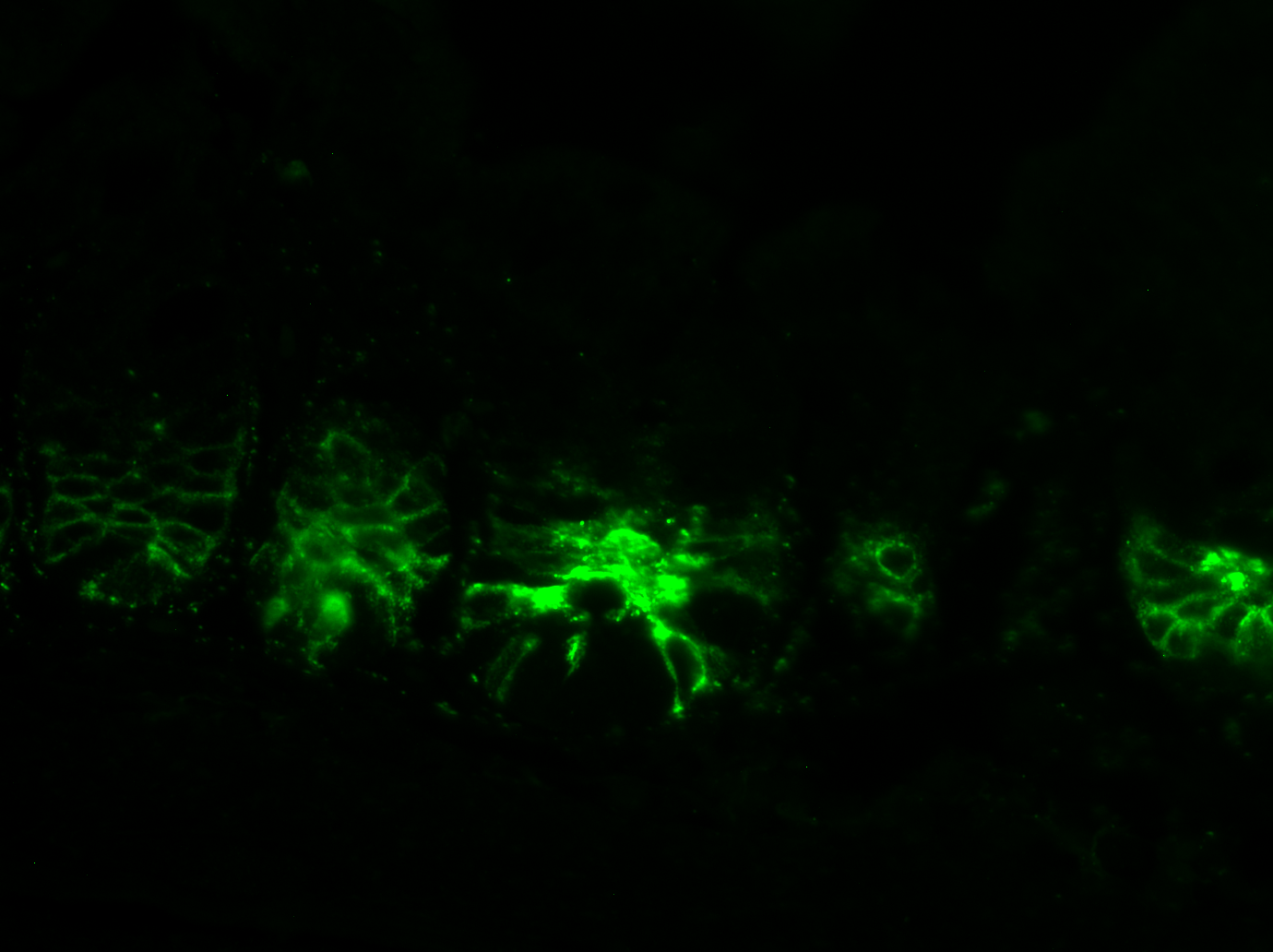

Supplement: Supplementary file 9 — Source Data for Figure 3 [file EMBR-24-e56030-s002.zip › Figure 3/Figure 3F-b-catenin OLFM4 staining/7. LEC-Foxc-DKO, OLFM4.TIF]

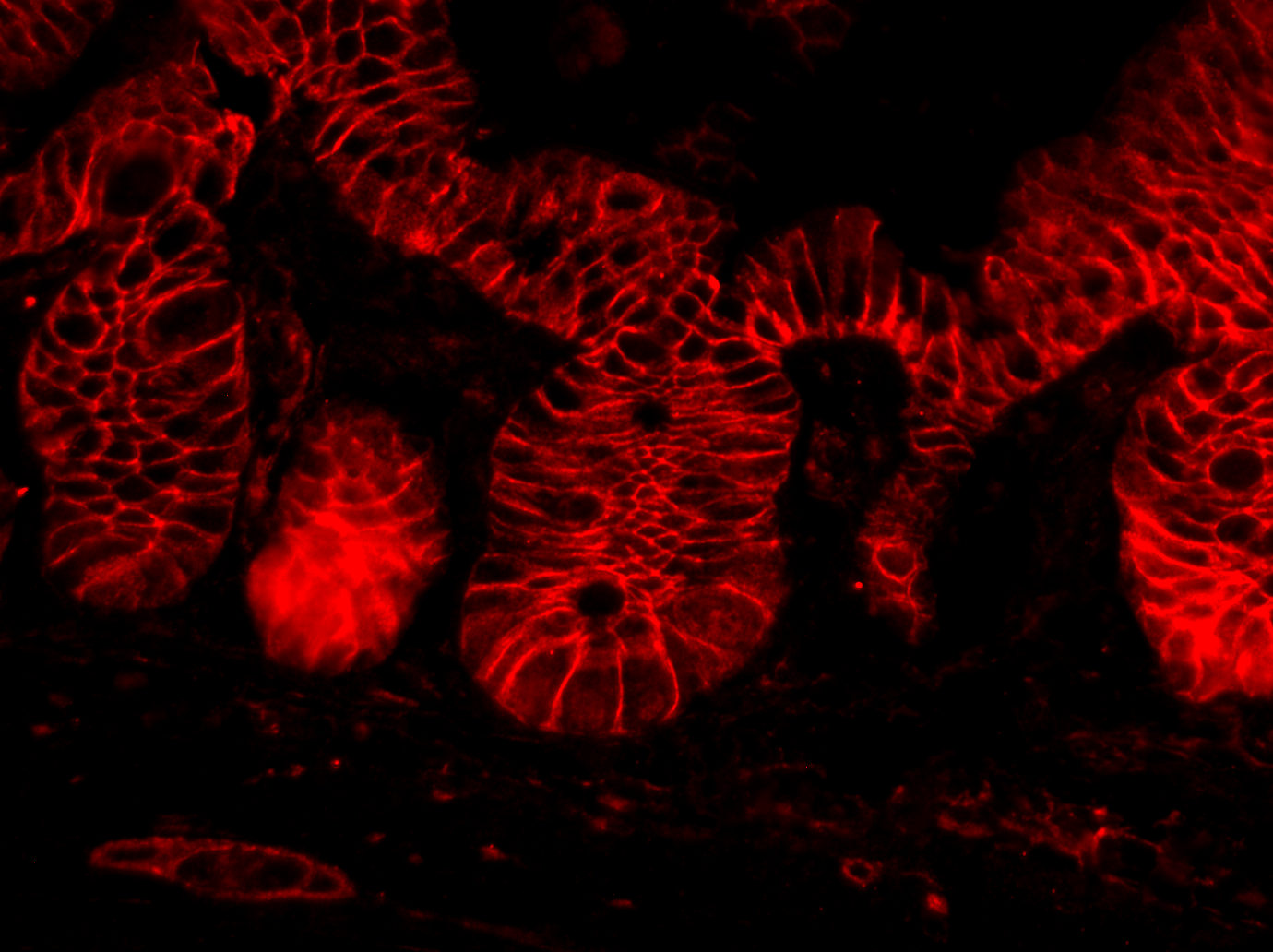

Supplement: Supplementary file 9 — Source Data for Figure 3 [file EMBR-24-e56030-s002.zip › Figure 3/Figure 3F-b-catenin OLFM4 staining/8. LEC-Foxc-DKO, b-catenin.TIF]

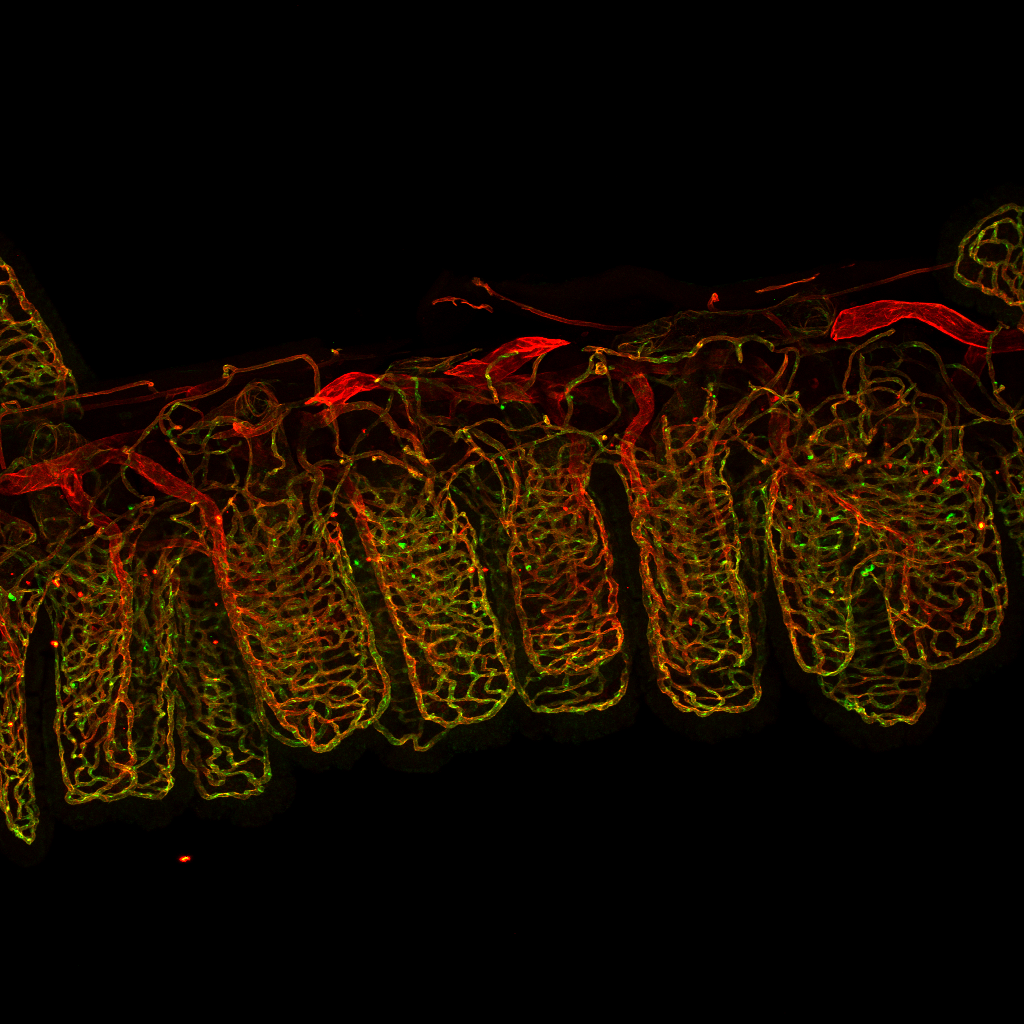

Supplement: Supplementary file 10 — Source Data for Figure 4 [file EMBR-24-e56030-s003.zip › Figure 4/Figure 4A-WM-VEGFR2 CD31/1-1. Control, sham, 2 colors.tif]

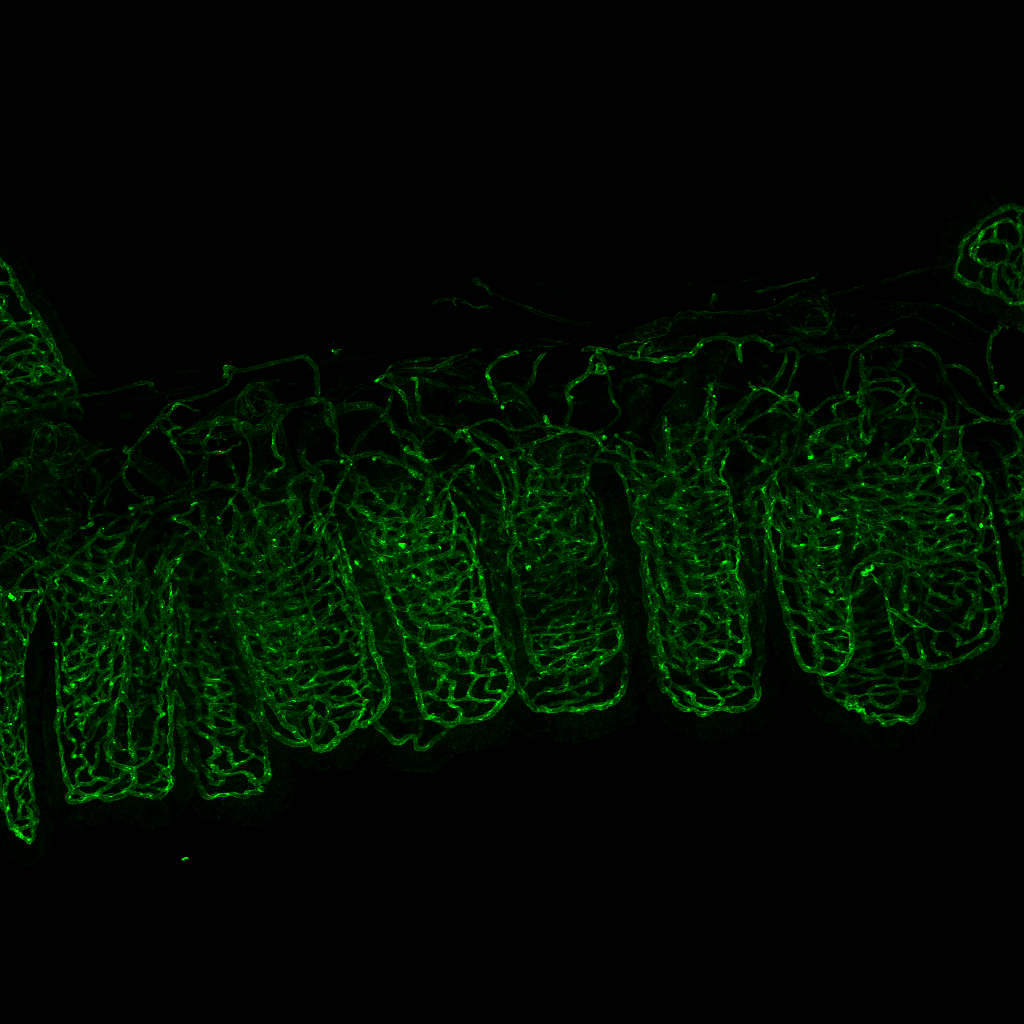

Supplement: Supplementary file 10 — Source Data for Figure 4 [file EMBR-24-e56030-s003.zip › Figure 4/Figure 4A-WM-VEGFR2 CD31/1-2. Control, sham, VEGFR2.tif]

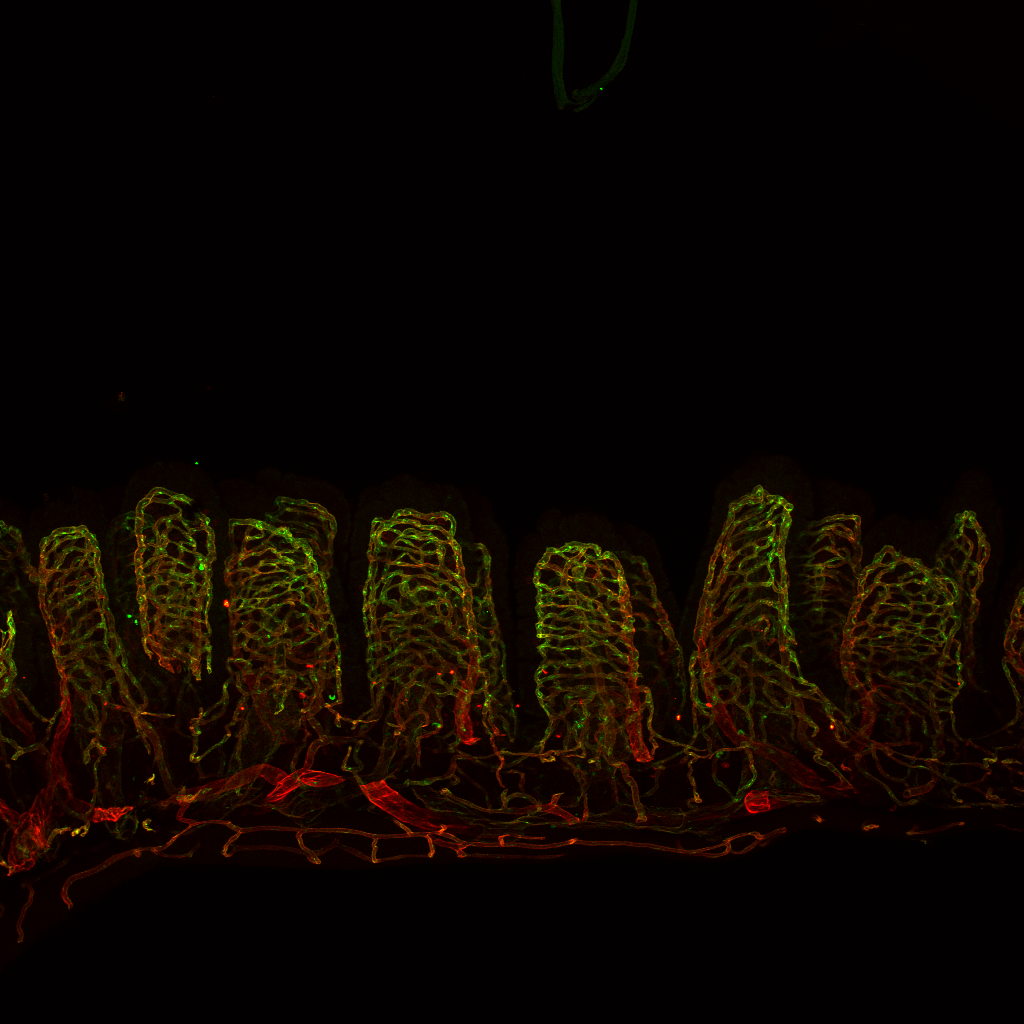

Supplement: Supplementary file 10 — Source Data for Figure 4 [file EMBR-24-e56030-s003.zip › Figure 4/Figure 4A-WM-VEGFR2 CD31/2-1. EC-Foxc-DKO, sham, 2 colors.tif]

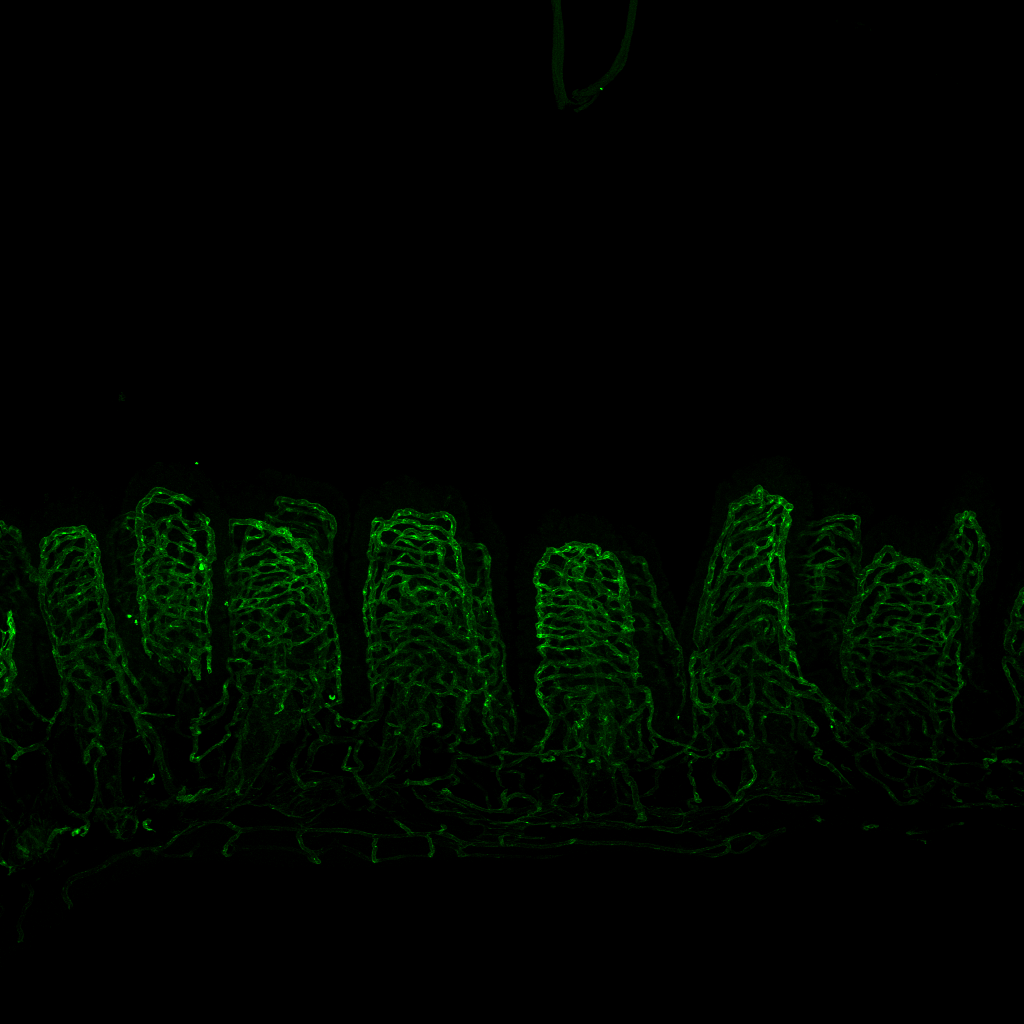

Supplement: Supplementary file 10 — Source Data for Figure 4 [file EMBR-24-e56030-s003.zip › Figure 4/Figure 4A-WM-VEGFR2 CD31/2-2. EC-Foxc-DKO, sham, VEGFR2.tif]

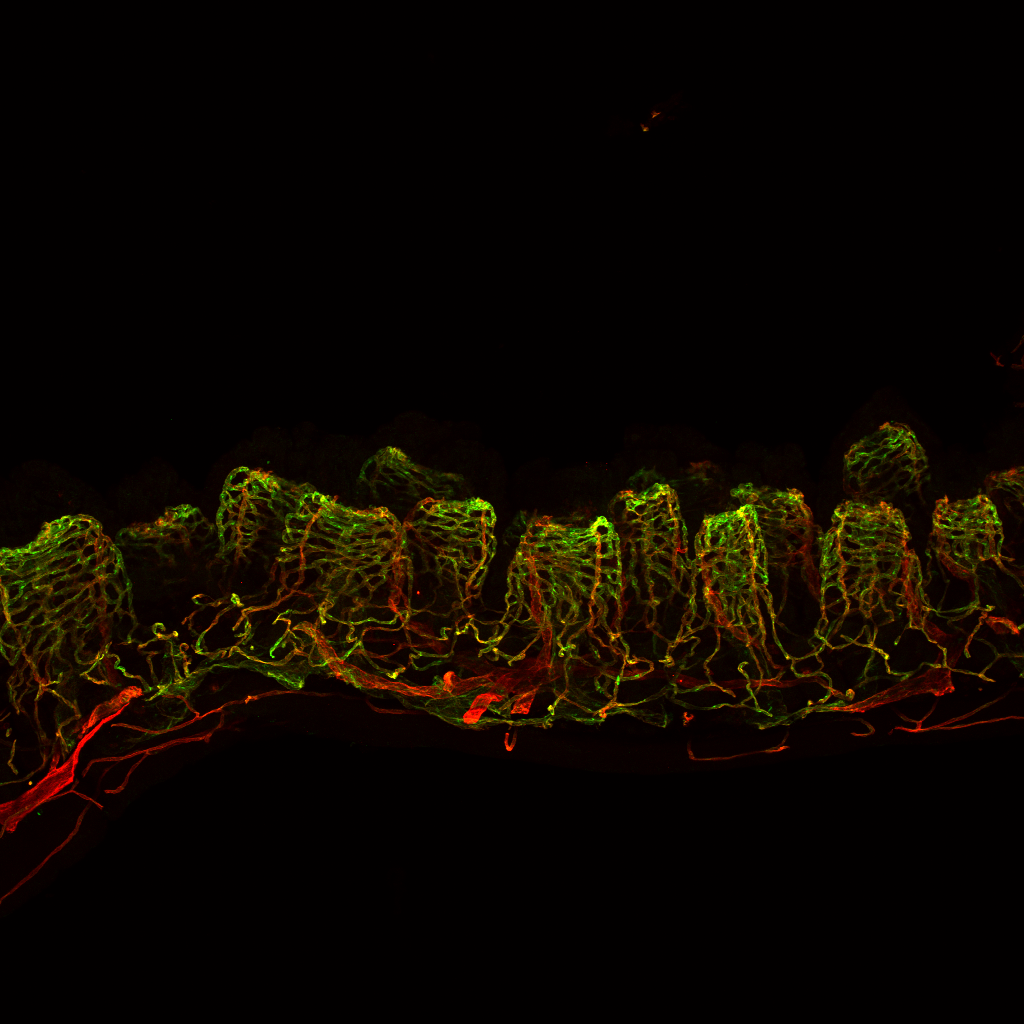

Supplement: Supplementary file 10 — Source Data for Figure 4 [file EMBR-24-e56030-s003.zip › Figure 4/Figure 4A-WM-VEGFR2 CD31/3-1. Control, IR, 2 colors.tif]

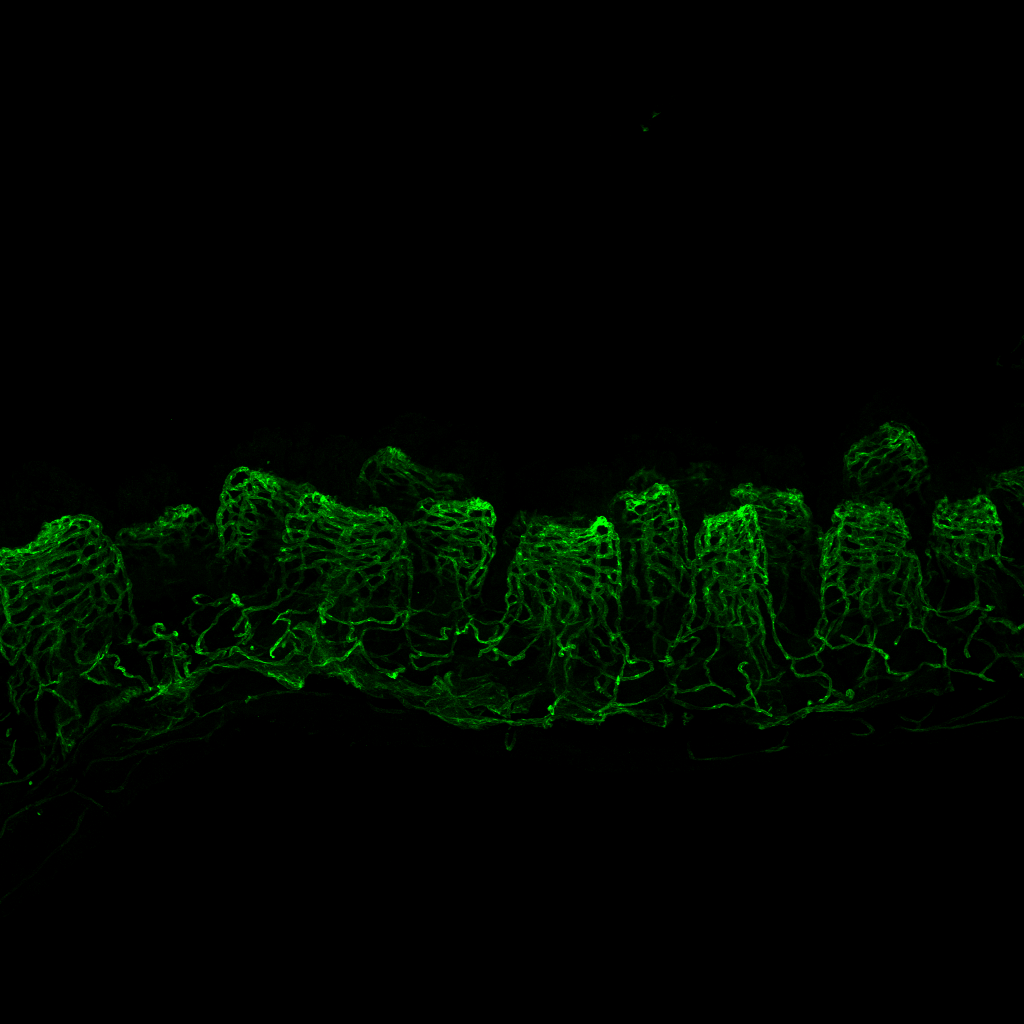

Supplement: Supplementary file 10 — Source Data for Figure 4 [file EMBR-24-e56030-s003.zip › Figure 4/Figure 4A-WM-VEGFR2 CD31/3-2. Control, IR, VEGFR2.tif]

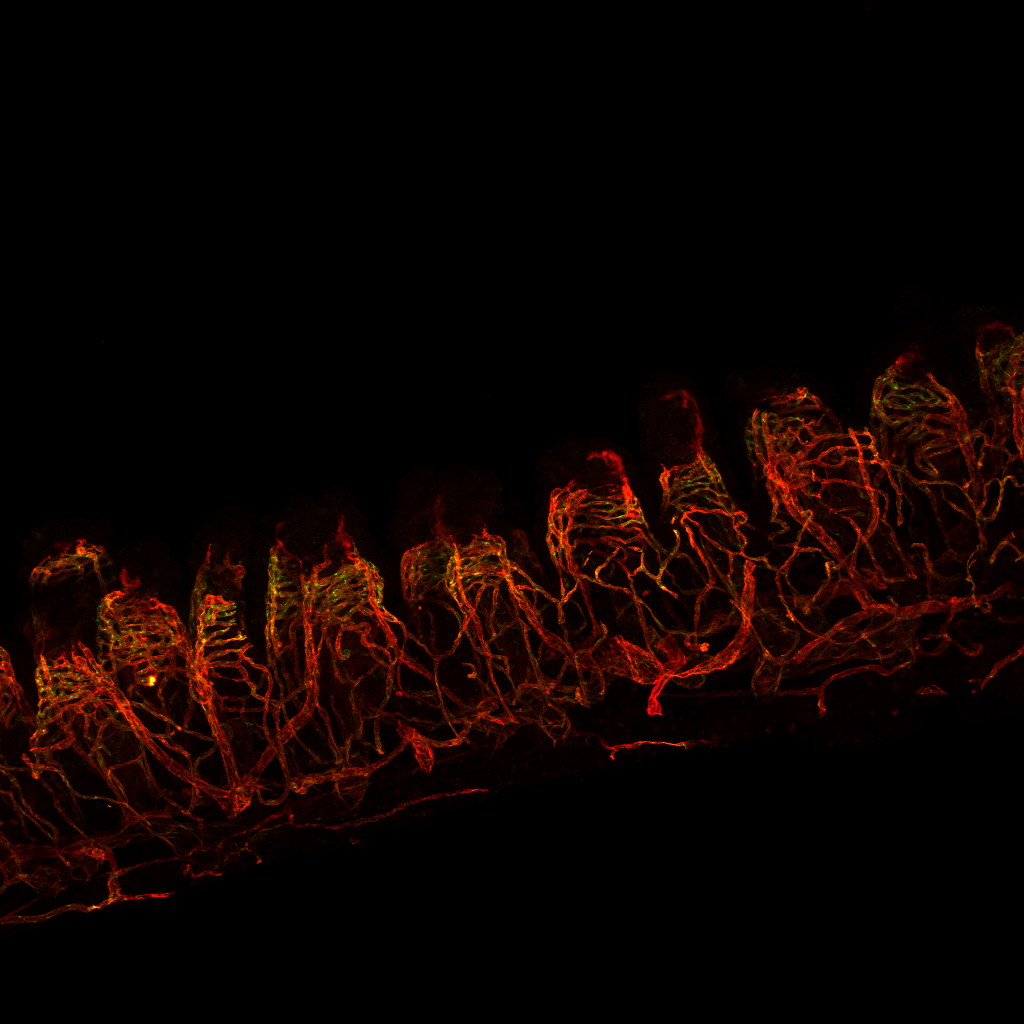

Supplement: Supplementary file 10 — Source Data for Figure 4 [file EMBR-24-e56030-s003.zip › Figure 4/Figure 4A-WM-VEGFR2 CD31/4-1. EC-Foxc-DKO, IR, 2 colors.tif]

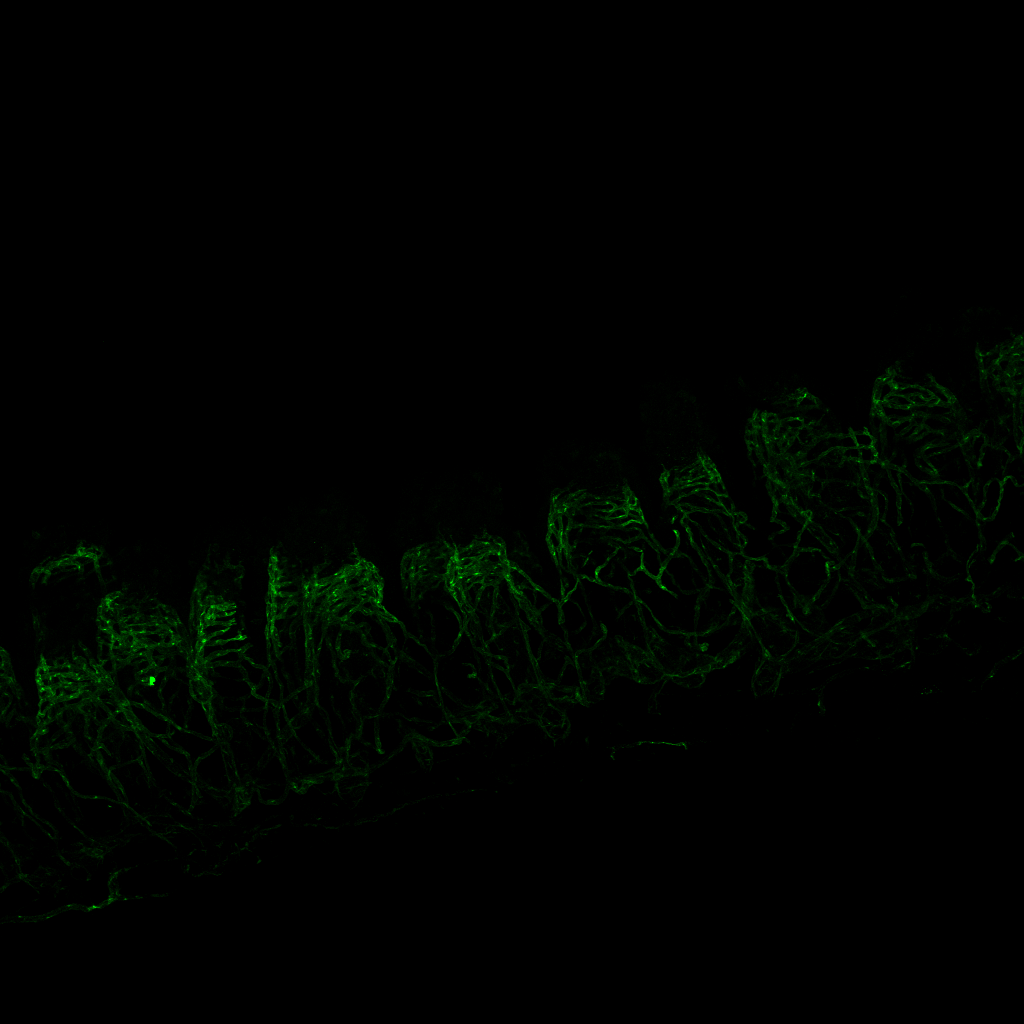

Supplement: Supplementary file 10 — Source Data for Figure 4 [file EMBR-24-e56030-s003.zip › Figure 4/Figure 4A-WM-VEGFR2 CD31/4-2. EC-Foxc-DKO, IR, VEGFR2.tif]

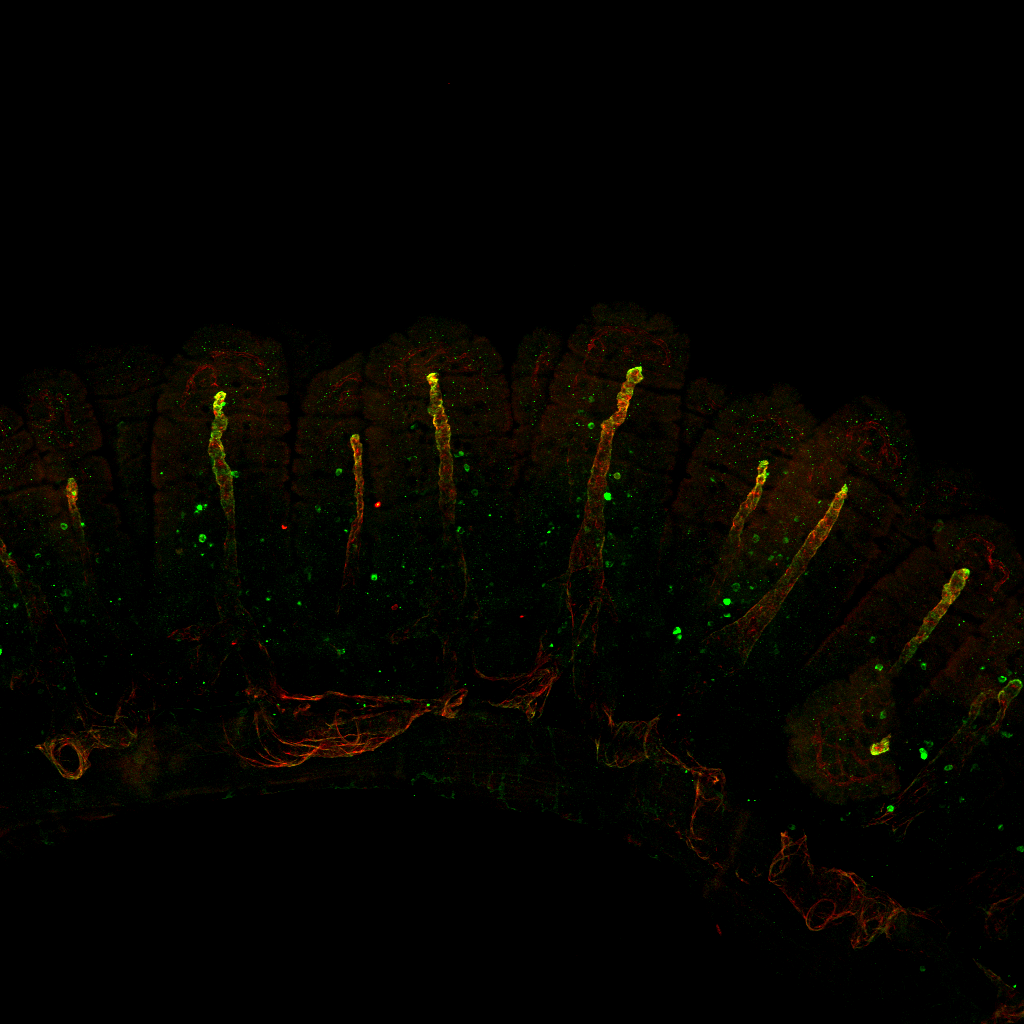

Supplement: Supplementary file 10 — Source Data for Figure 4 [file EMBR-24-e56030-s003.zip › Figure 4/Figure 4B-WM-VEGFR3 LYVE1/1-1. Control, sham, 2 colors.tif]

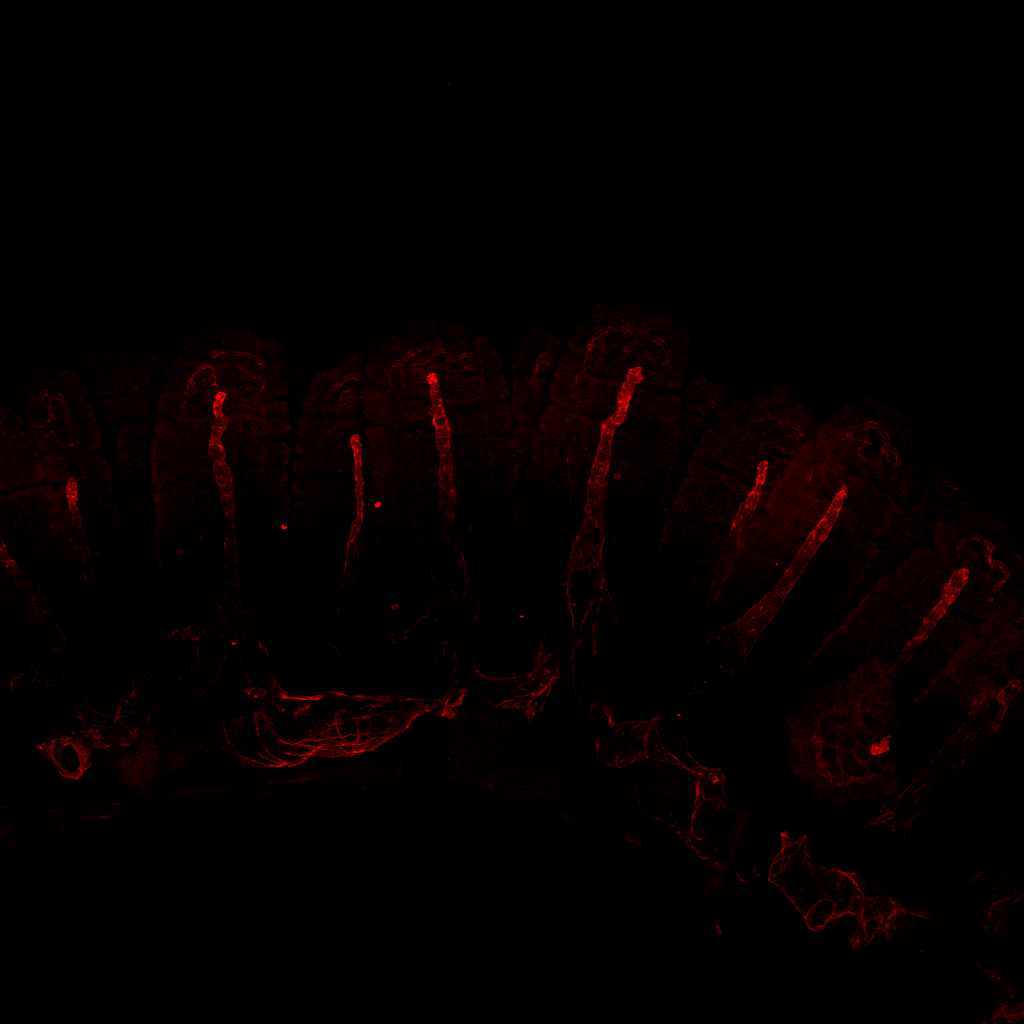

Supplement: Supplementary file 10 — Source Data for Figure 4 [file EMBR-24-e56030-s003.zip › Figure 4/Figure 4B-WM-VEGFR3 LYVE1/1-2. Control, sham, VEGFR3.tif]

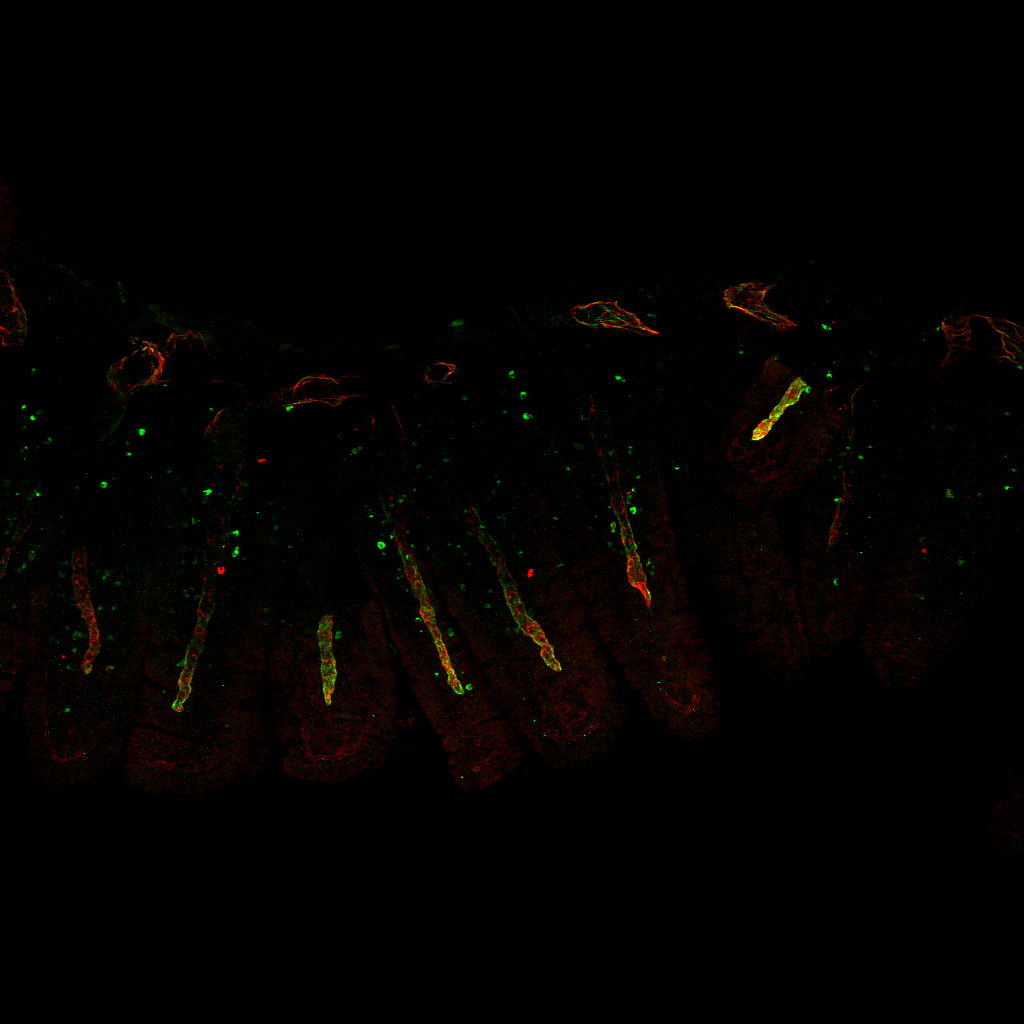

Supplement: Supplementary file 10 — Source Data for Figure 4 [file EMBR-24-e56030-s003.zip › Figure 4/Figure 4B-WM-VEGFR3 LYVE1/2-1. EC-Foxc-DKO, sham, 2 colors.tif]

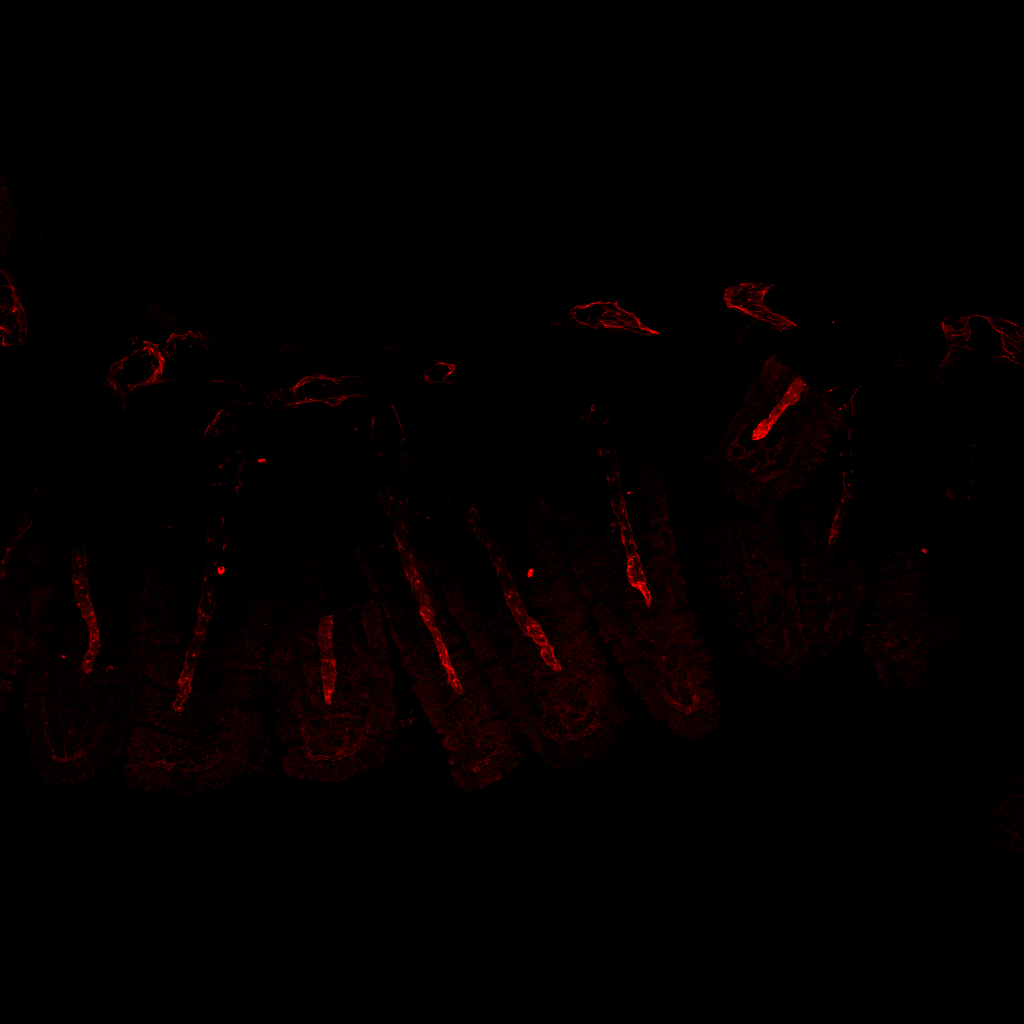

Supplement: Supplementary file 10 — Source Data for Figure 4 [file EMBR-24-e56030-s003.zip › Figure 4/Figure 4B-WM-VEGFR3 LYVE1/2-2. EC-Foxc-DKO, sham, VEGFR3.tif]

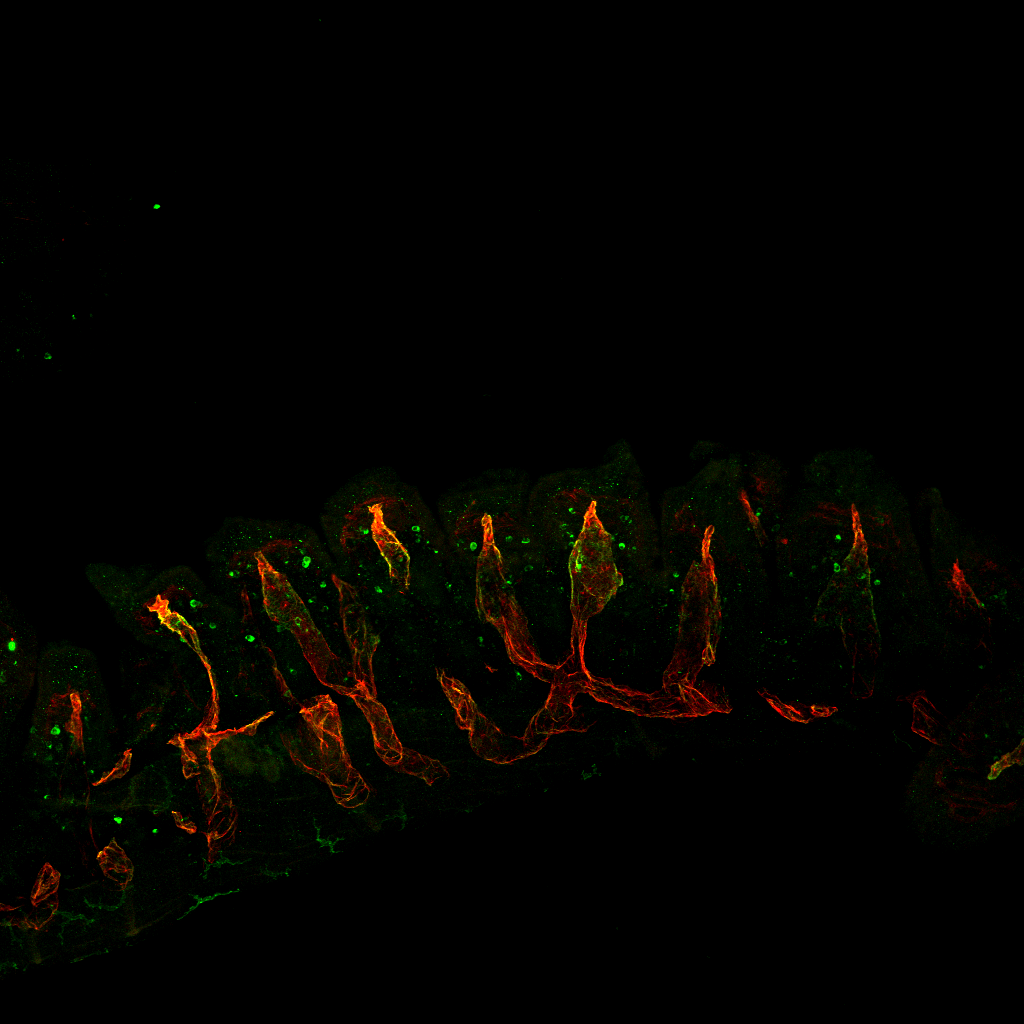

Supplement: Supplementary file 10 — Source Data for Figure 4 [file EMBR-24-e56030-s003.zip › Figure 4/Figure 4B-WM-VEGFR3 LYVE1/3-1. Control, IR, 2 colors.tif]

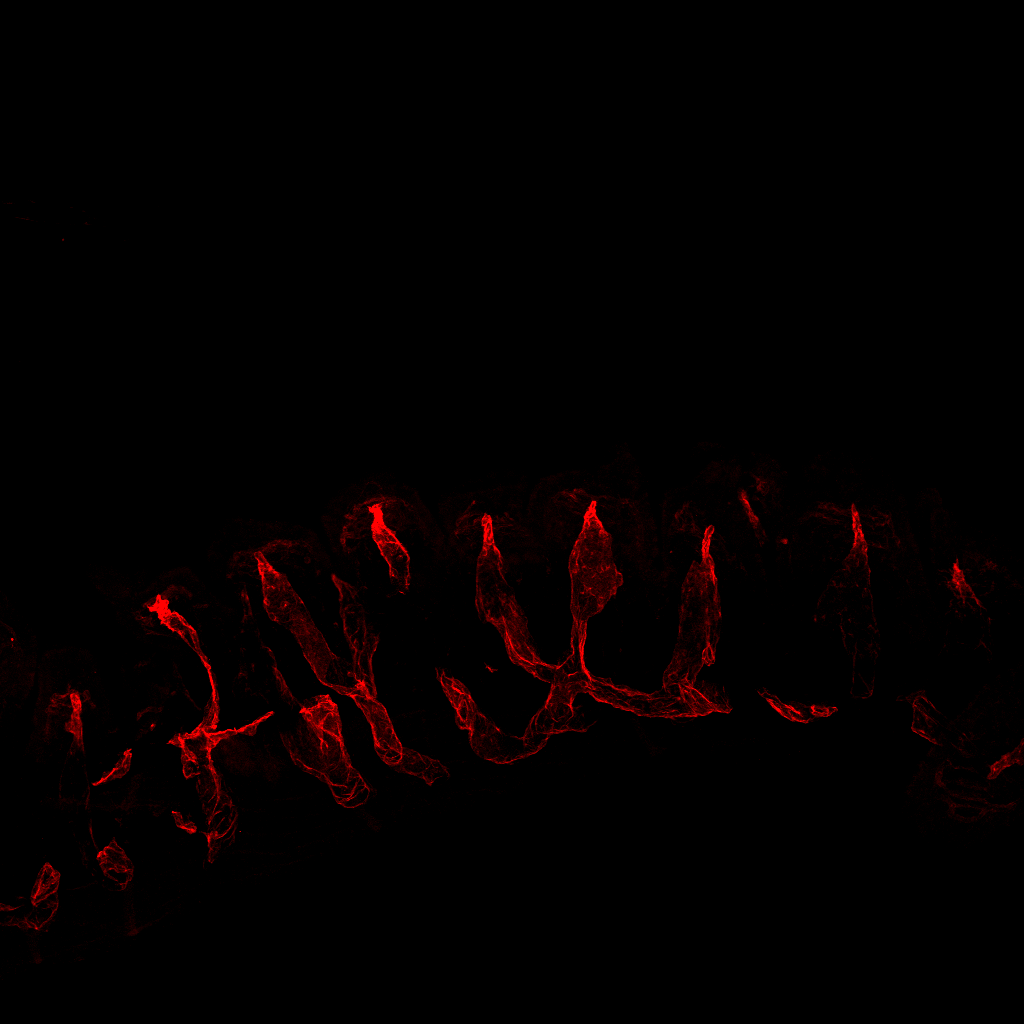

Supplement: Supplementary file 10 — Source Data for Figure 4 [file EMBR-24-e56030-s003.zip › Figure 4/Figure 4B-WM-VEGFR3 LYVE1/3-2. Control, IR, VEGFR3.tif]

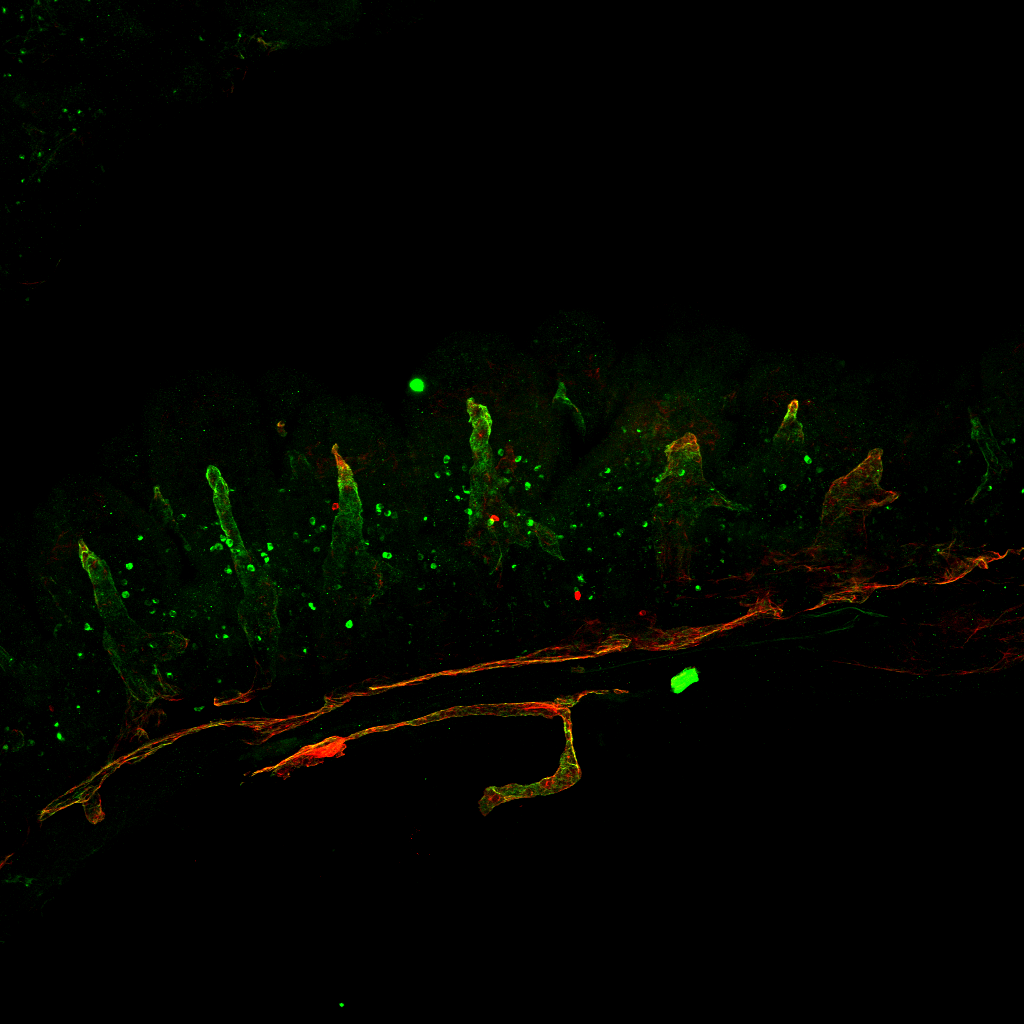

Supplement: Supplementary file 10 — Source Data for Figure 4 [file EMBR-24-e56030-s003.zip › Figure 4/Figure 4B-WM-VEGFR3 LYVE1/4-1. EC-Foxc-DKO, IR, 2 colors.tif]

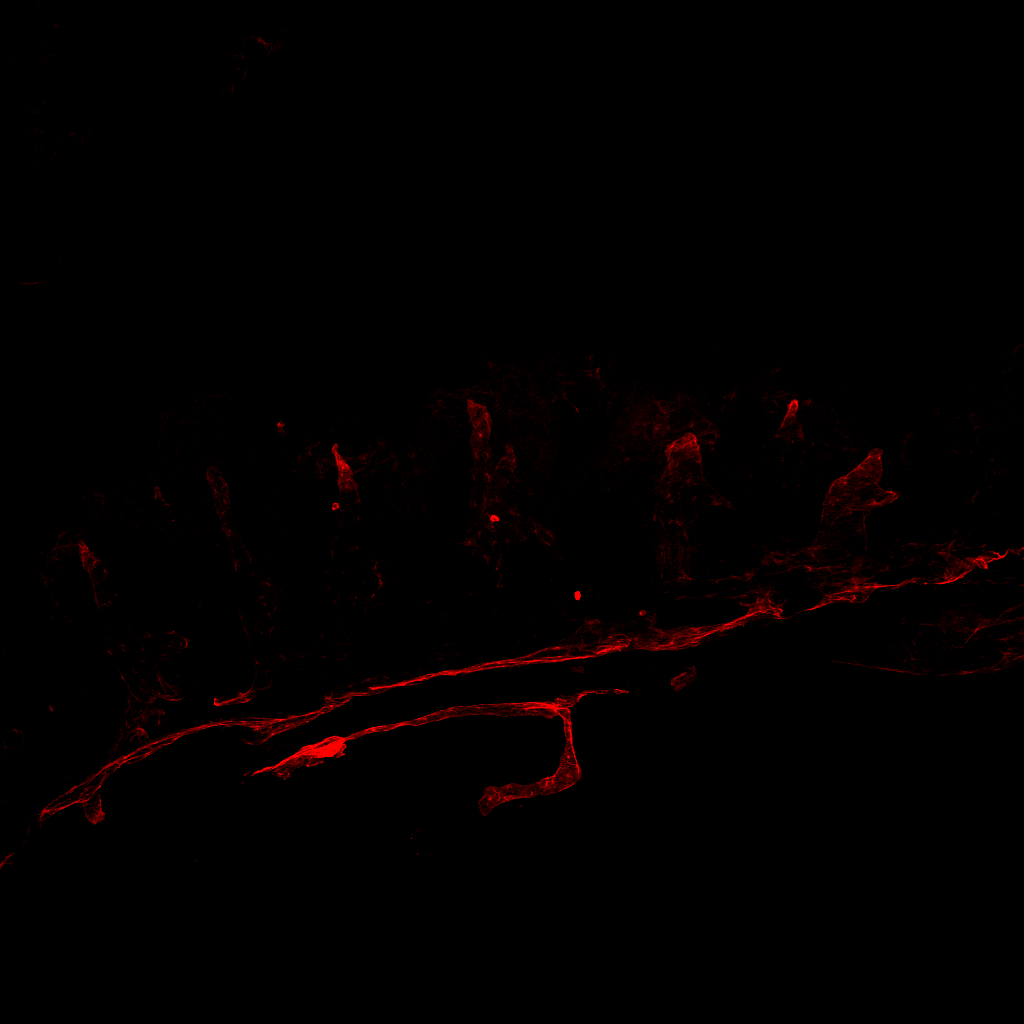

Supplement: Supplementary file 10 — Source Data for Figure 4 [file EMBR-24-e56030-s003.zip › Figure 4/Figure 4B-WM-VEGFR3 LYVE1/4-2. EC-Foxc-DKO, IR, VEGFR3.tif]

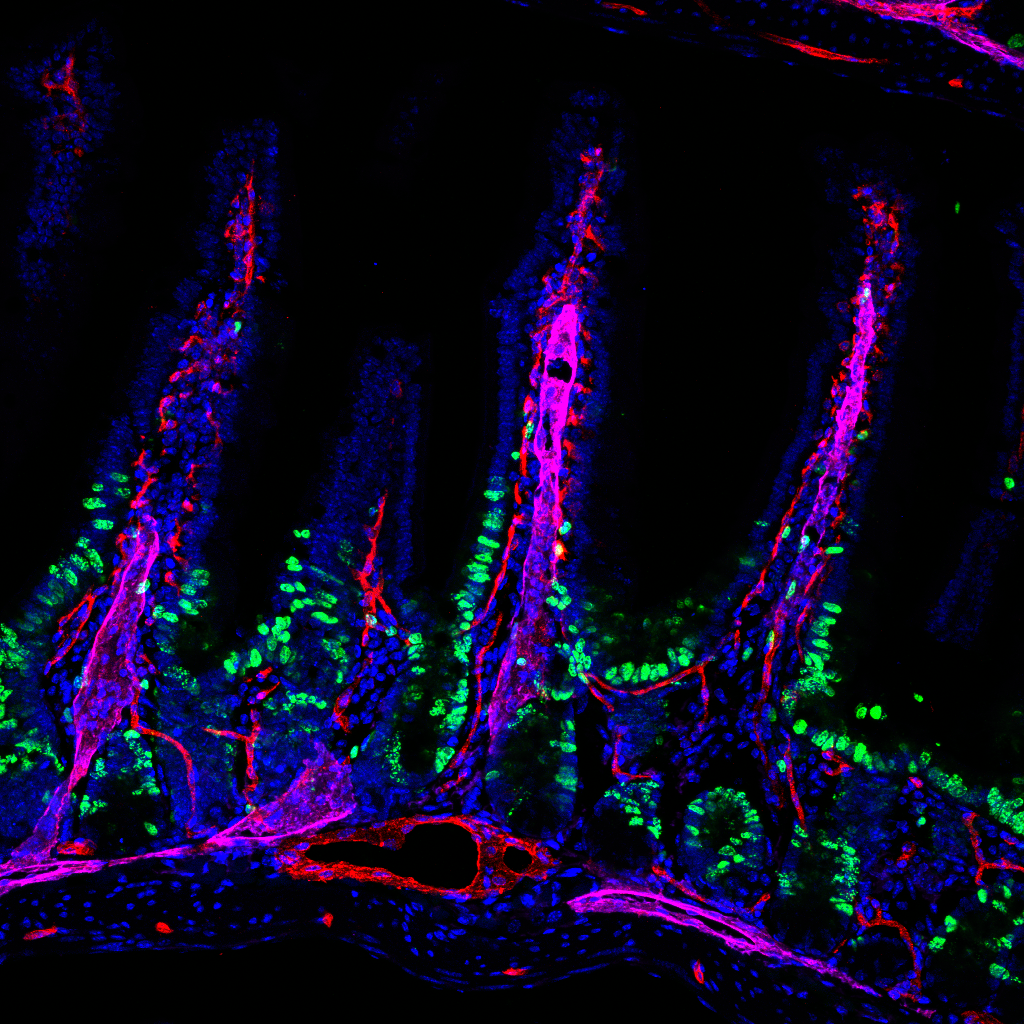

Supplement: Supplementary file 11 — Source Data for Figure 5 [file EMBR-24-e56030-s012.zip › Figure 5/Figure 5A-IHC-BrdU CD31 LYVE1/1. Control, sham (insert 1,2 are included).tif]

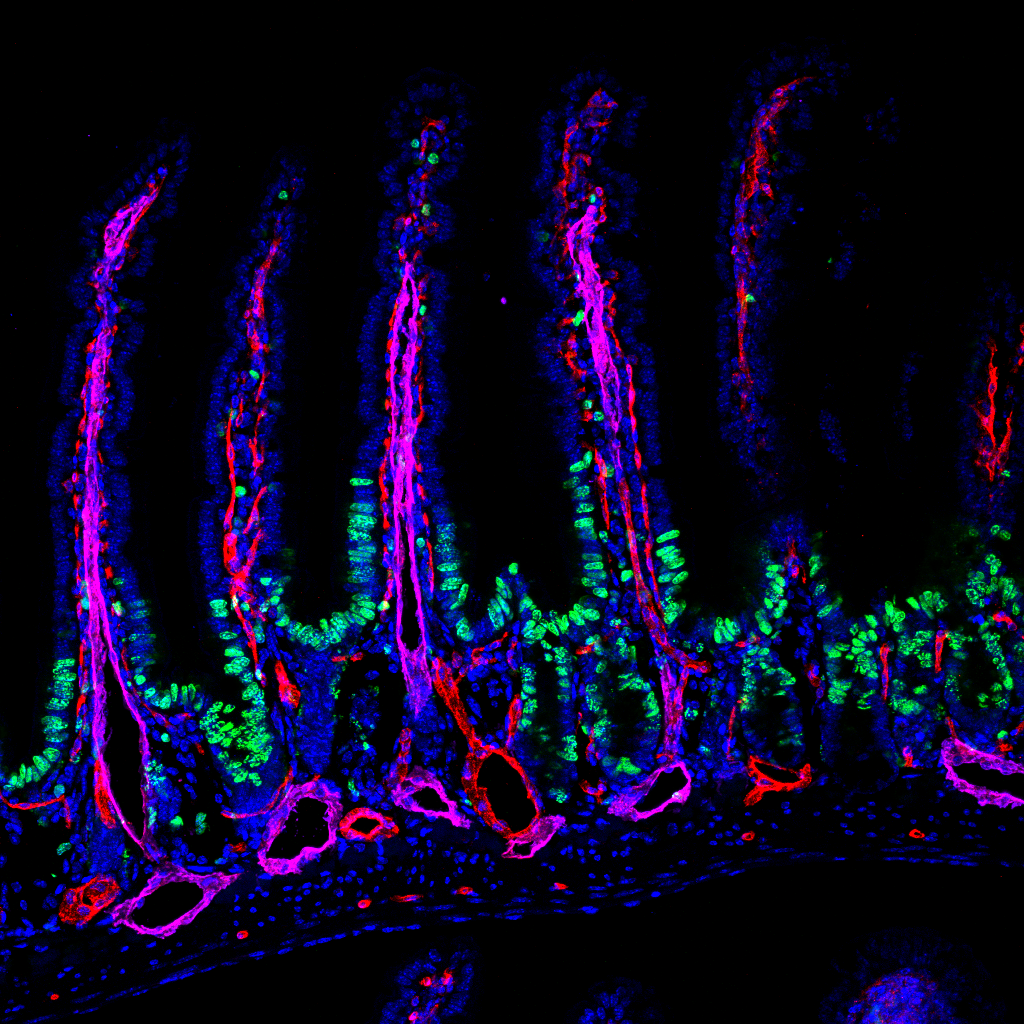

Supplement: Supplementary file 11 — Source Data for Figure 5 [file EMBR-24-e56030-s012.zip › Figure 5/Figure 5A-IHC-BrdU CD31 LYVE1/2. EC-Foxc-DKO, sham (insert 3,4 are included).tif]

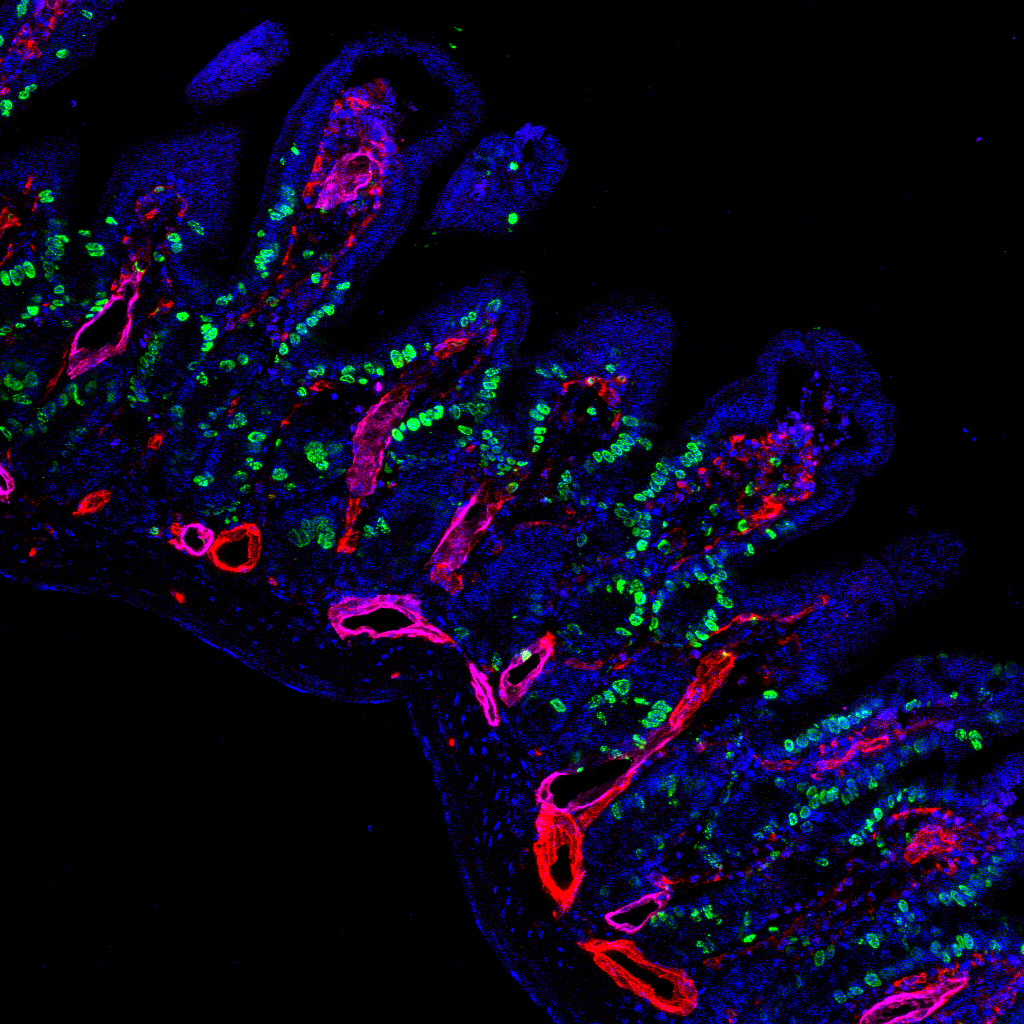

Supplement: Supplementary file 11 — Source Data for Figure 5 [file EMBR-24-e56030-s012.zip › Figure 5/Figure 5A-IHC-BrdU CD31 LYVE1/3. Control, IR (insert 5,6 are included).tif]

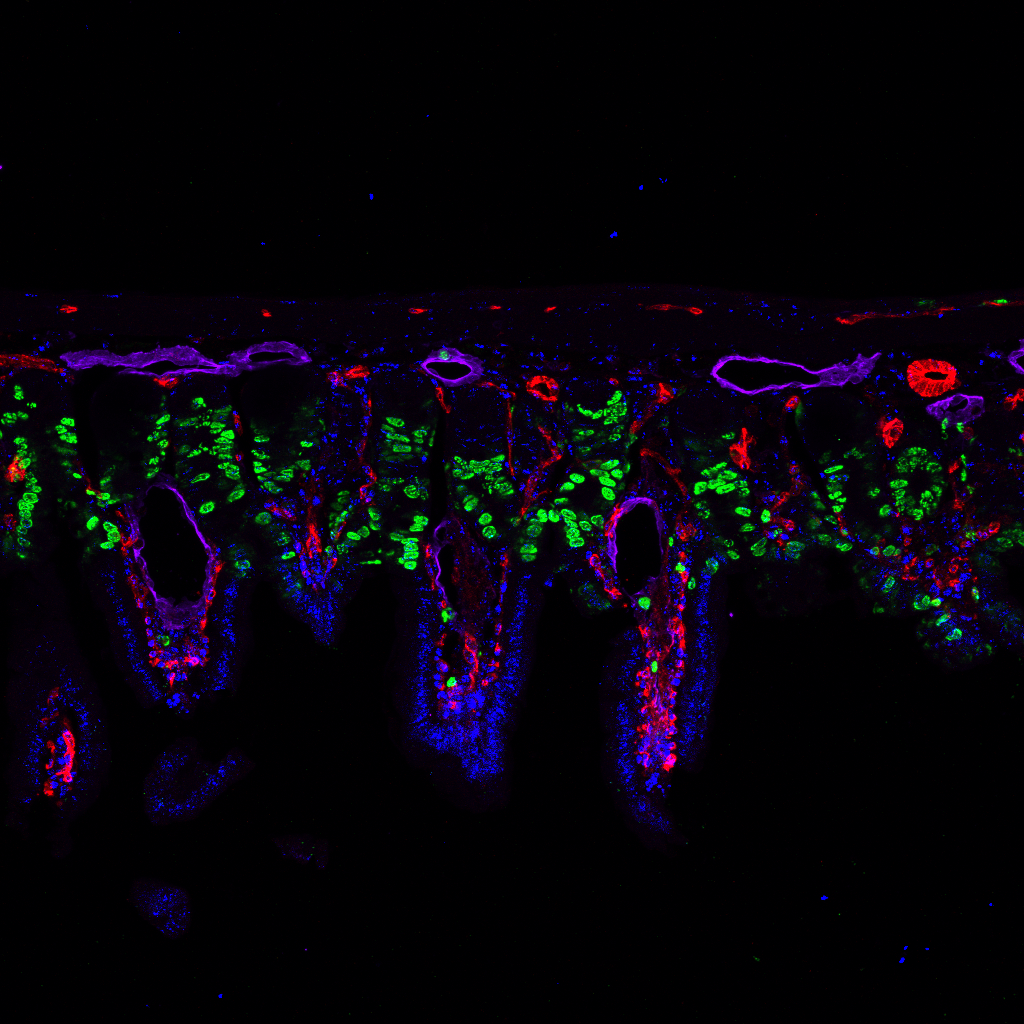

Supplement: Supplementary file 11 — Source Data for Figure 5 [file EMBR-24-e56030-s012.zip › Figure 5/Figure 5A-IHC-BrdU CD31 LYVE1/4. EC-Foxc-DKO, IR (insert 7,8 are included).tif]

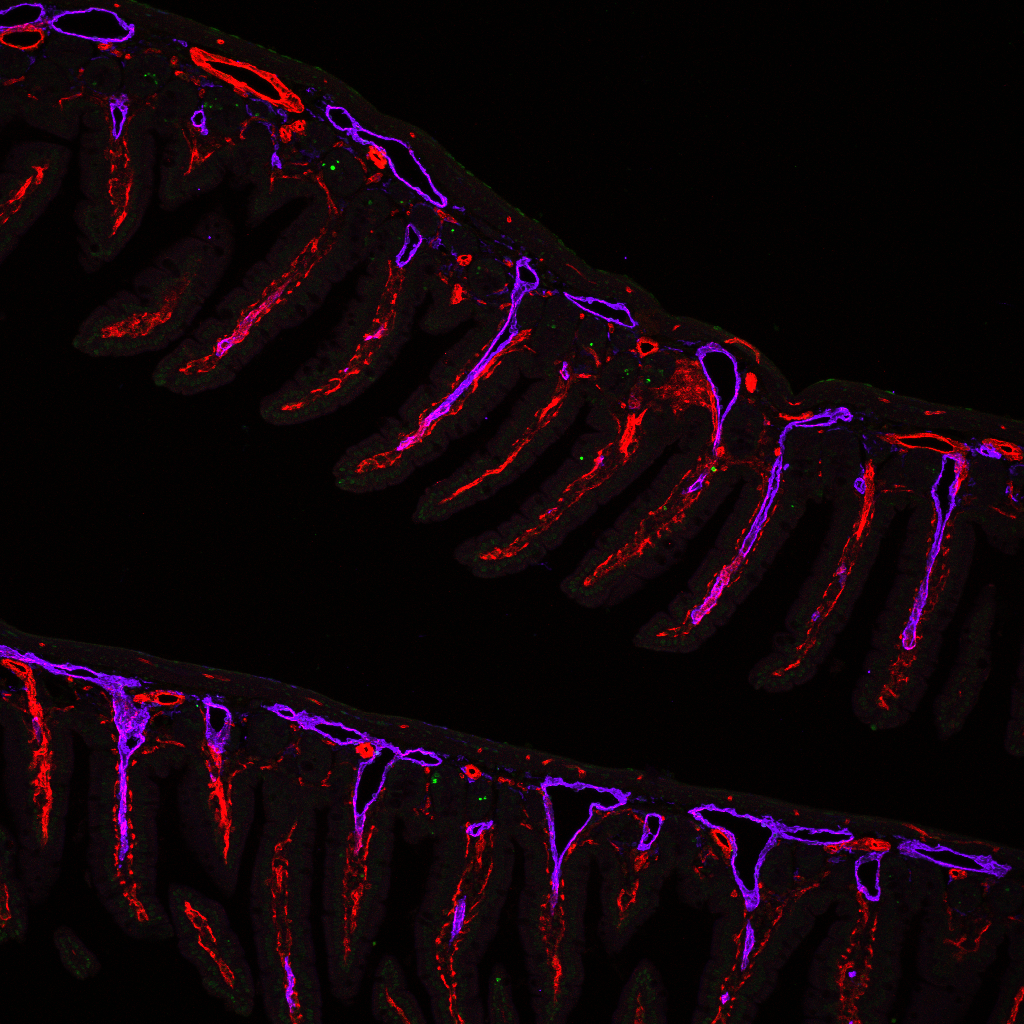

Supplement: Supplementary file 11 — Source Data for Figure 5 [file EMBR-24-e56030-s012.zip › Figure 5/Figure 5C-IHC-TUNEL CD31 LYVE1/1. Control sham.tif]

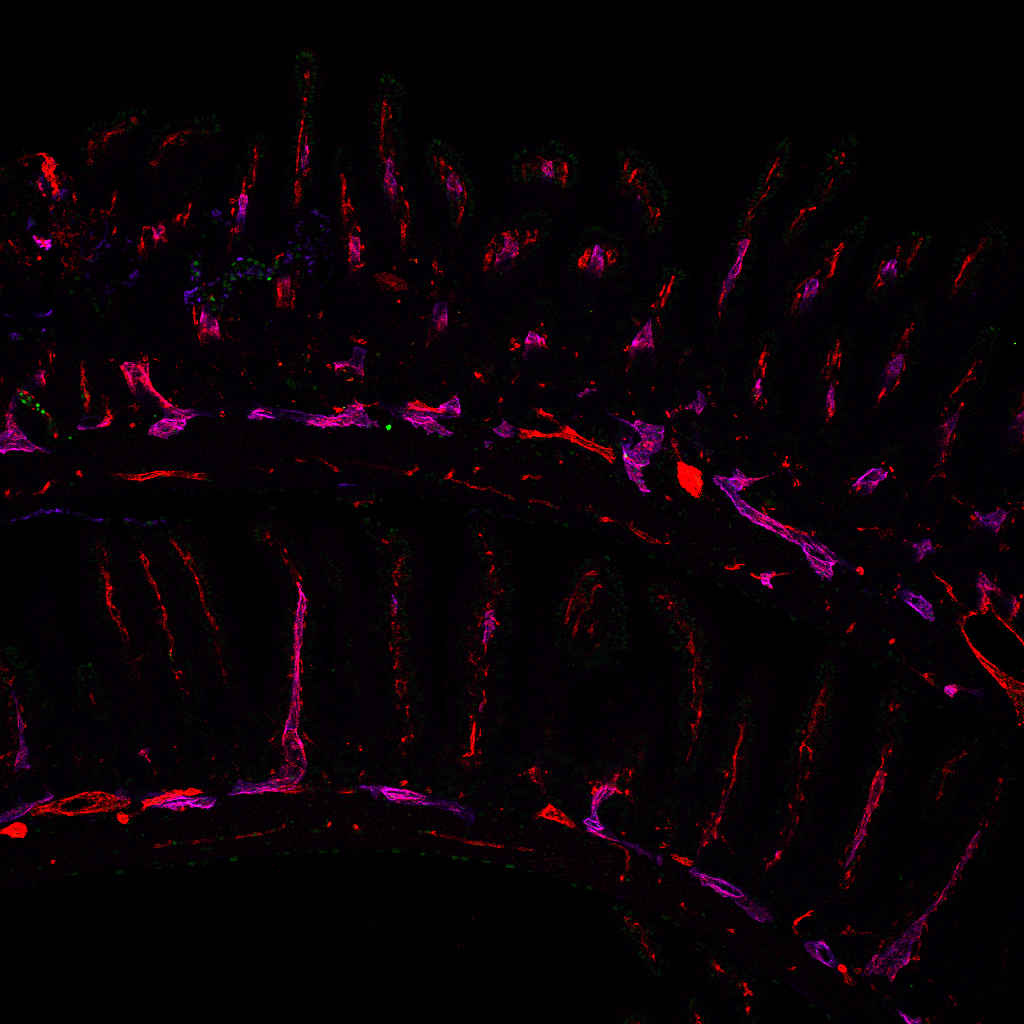

Supplement: Supplementary file 11 — Source Data for Figure 5 [file EMBR-24-e56030-s012.zip › Figure 5/Figure 5C-IHC-TUNEL CD31 LYVE1/2. EC-Foxc-DKO sham.tif]

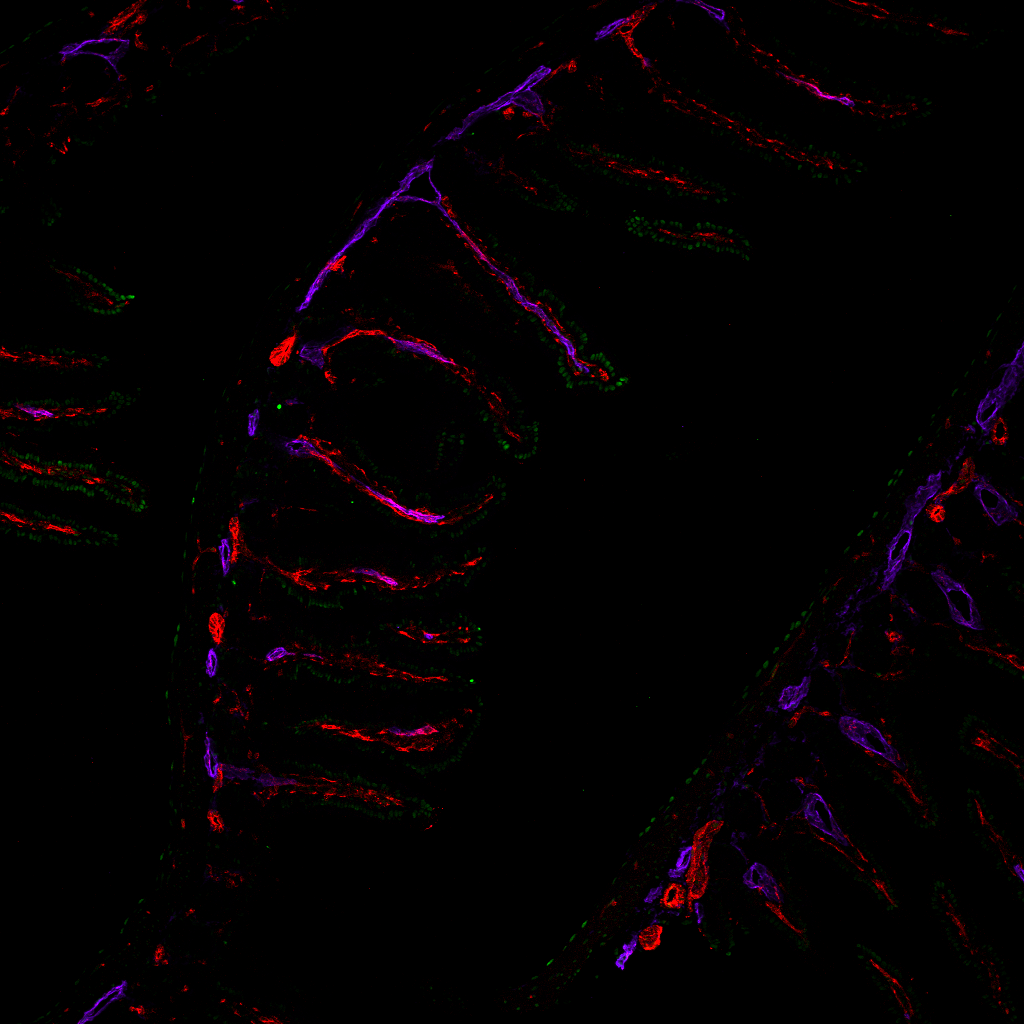

Supplement: Supplementary file 11 — Source Data for Figure 5 [file EMBR-24-e56030-s012.zip › Figure 5/Figure 5C-IHC-TUNEL CD31 LYVE1/3. Control IR.tif]

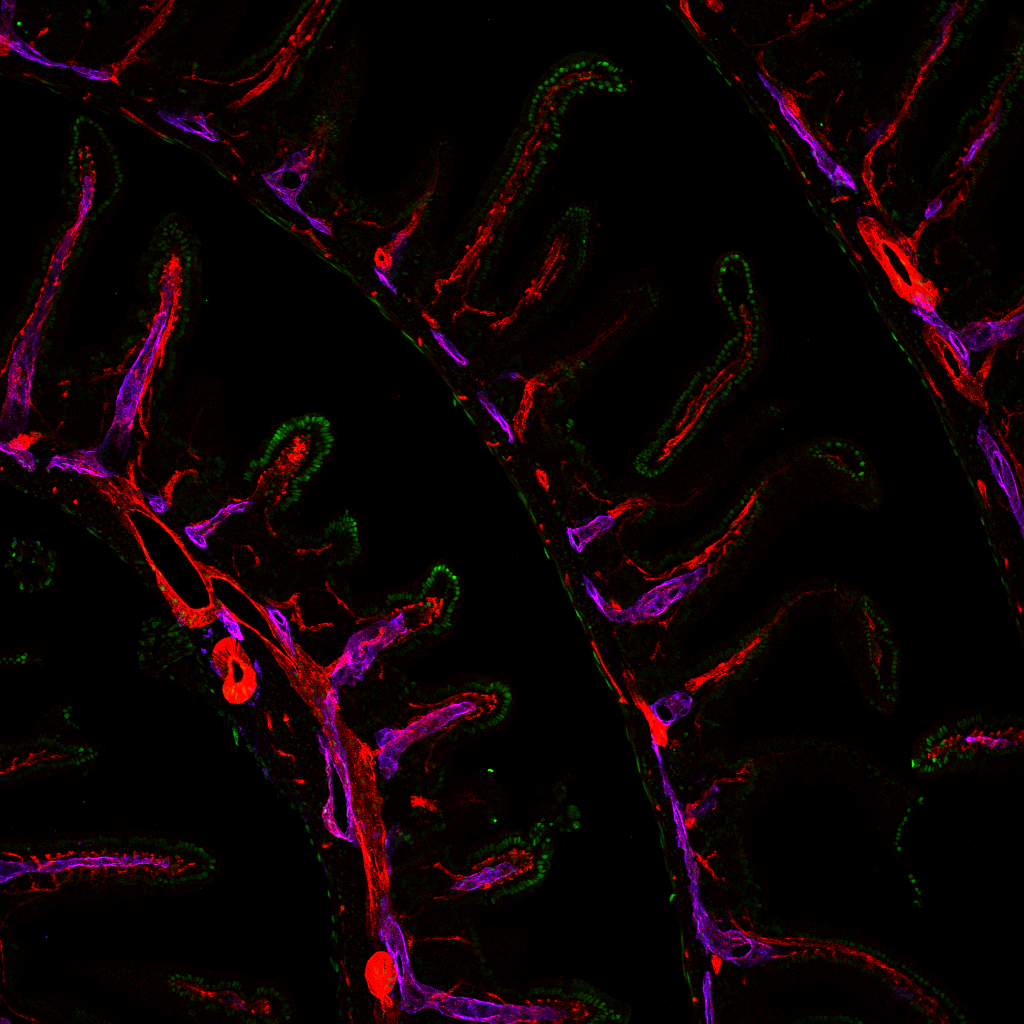

Supplement: Supplementary file 11 — Source Data for Figure 5 [file EMBR-24-e56030-s012.zip › Figure 5/Figure 5C-IHC-TUNEL CD31 LYVE1/4. EC-Foxc-DKO IR (insert 1,2,3,4 are included).tif]

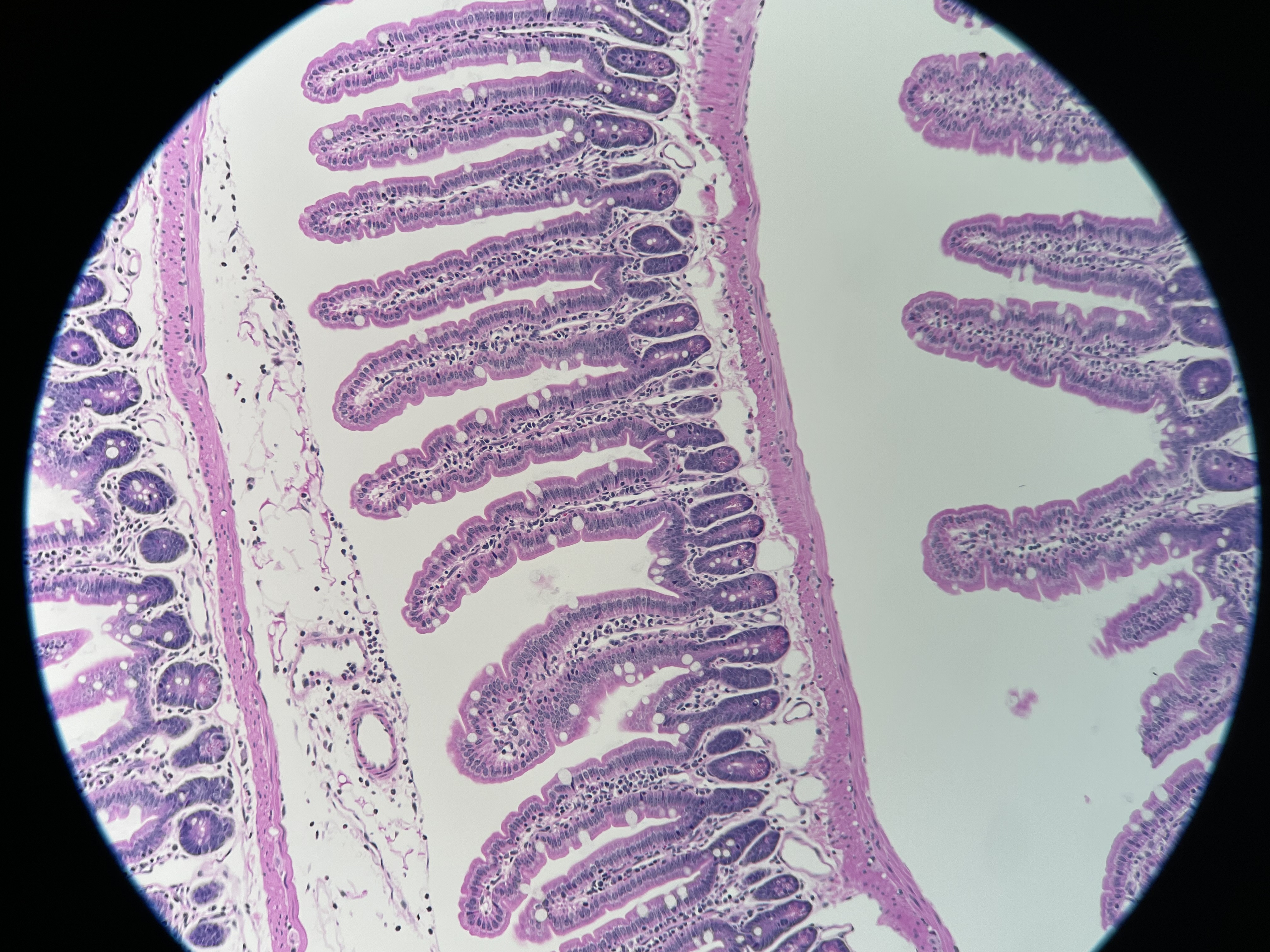

Supplement: Supplementary file 15 — Source Data for Figure 9 [file EMBR-24-e56030-s009.zip › Figure 9/Figure 9A-HE (EC-specific line)/1. Control, PBS.jpeg]

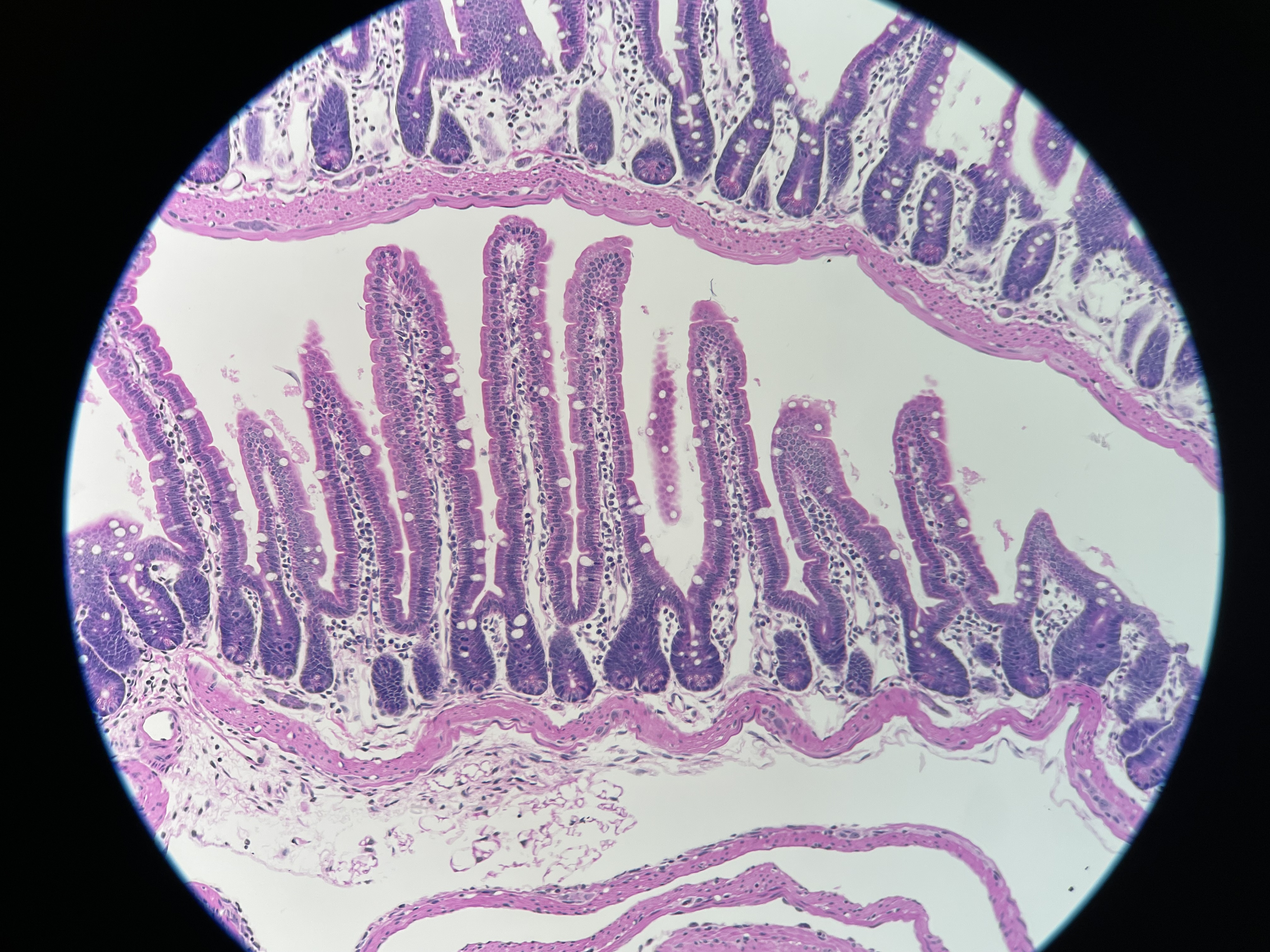

Supplement: Supplementary file 15 — Source Data for Figure 9 [file EMBR-24-e56030-s009.zip › Figure 9/Figure 9A-HE (EC-specific line)/2. Control, RSPO3.jpeg]

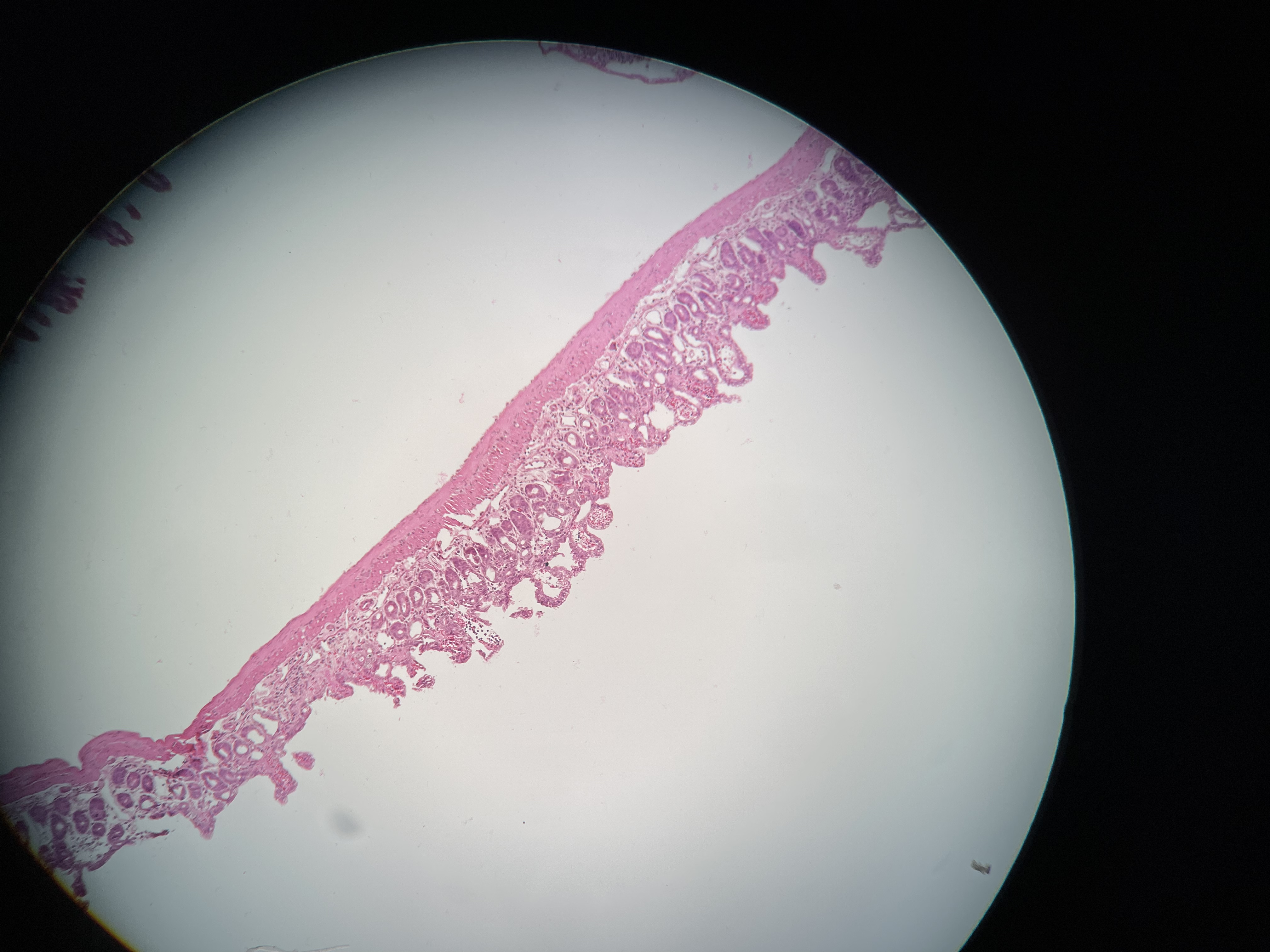

Supplement: Supplementary file 15 — Source Data for Figure 9 [file EMBR-24-e56030-s009.zip › Figure 9/Figure 9A-HE (EC-specific line)/3. EC-Foxc-DKO, PBS.jpeg]

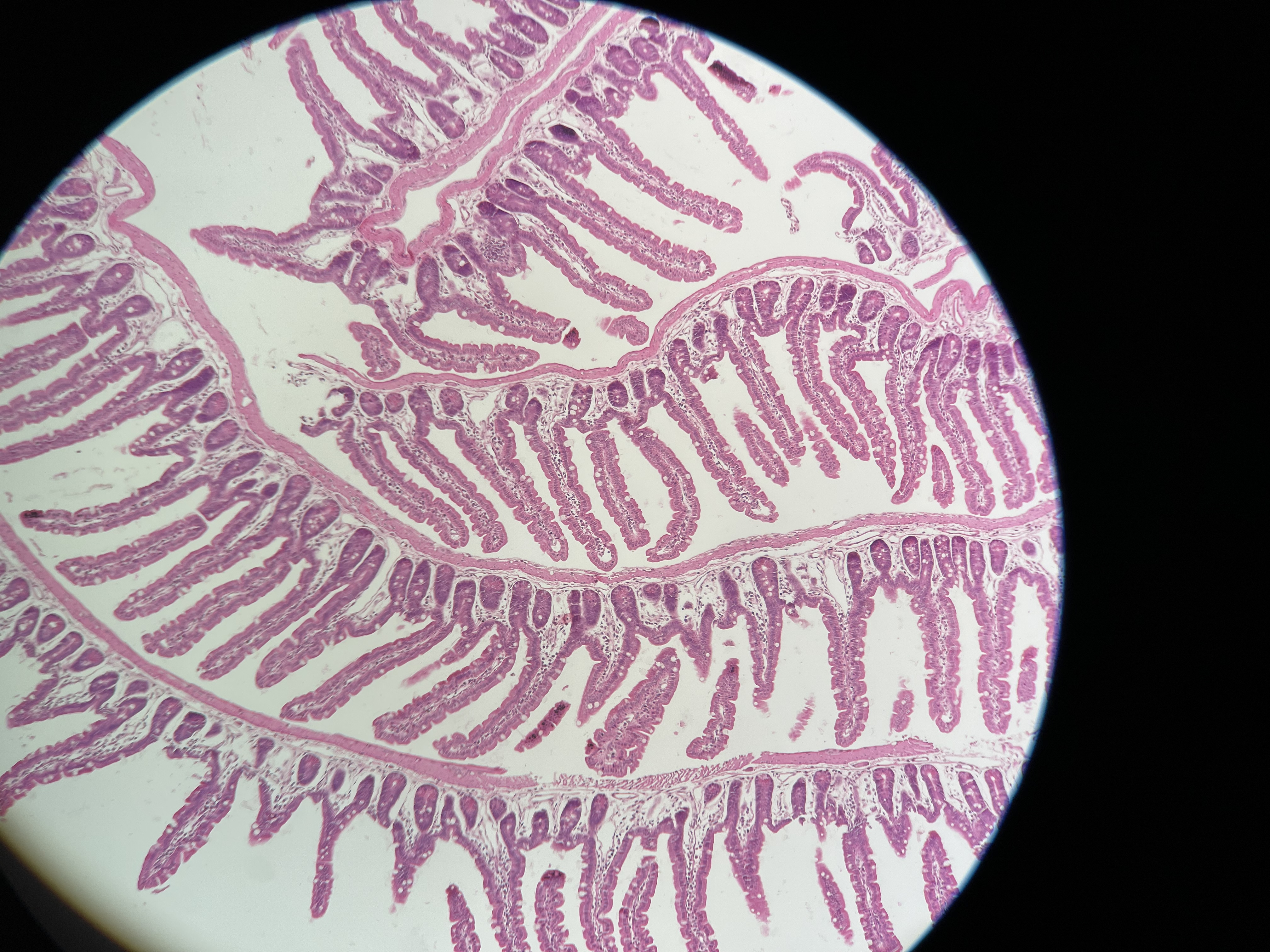

Supplement: Supplementary file 15 — Source Data for Figure 9 [file EMBR-24-e56030-s009.zip › Figure 9/Figure 9A-HE (EC-specific line)/4. EC-Foxc-DKO, RSPO3.jpeg]

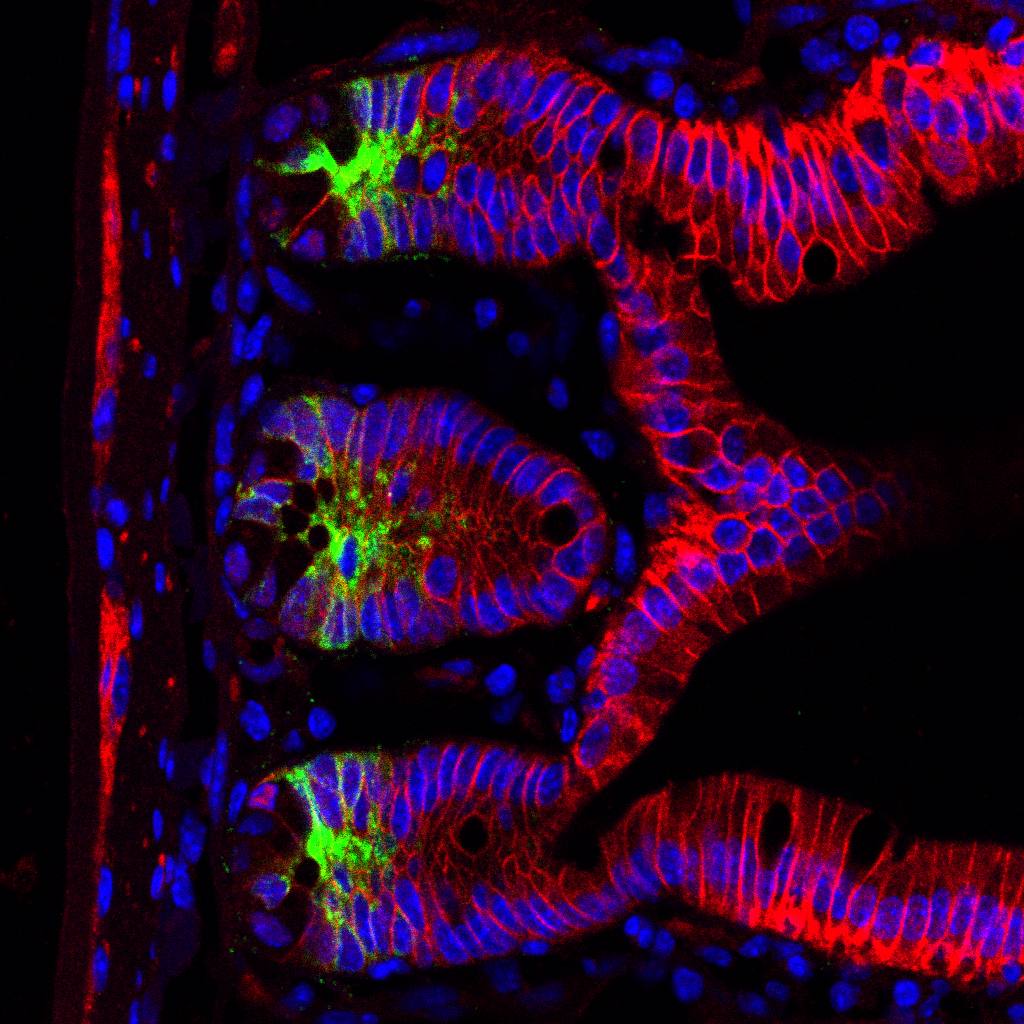

Supplement: Supplementary file 15 — Source Data for Figure 9 [file EMBR-24-e56030-s009.zip › Figure 9/Figure 9C -b-catenin OLFM4/1-1. PBS, 3 colors.tif]

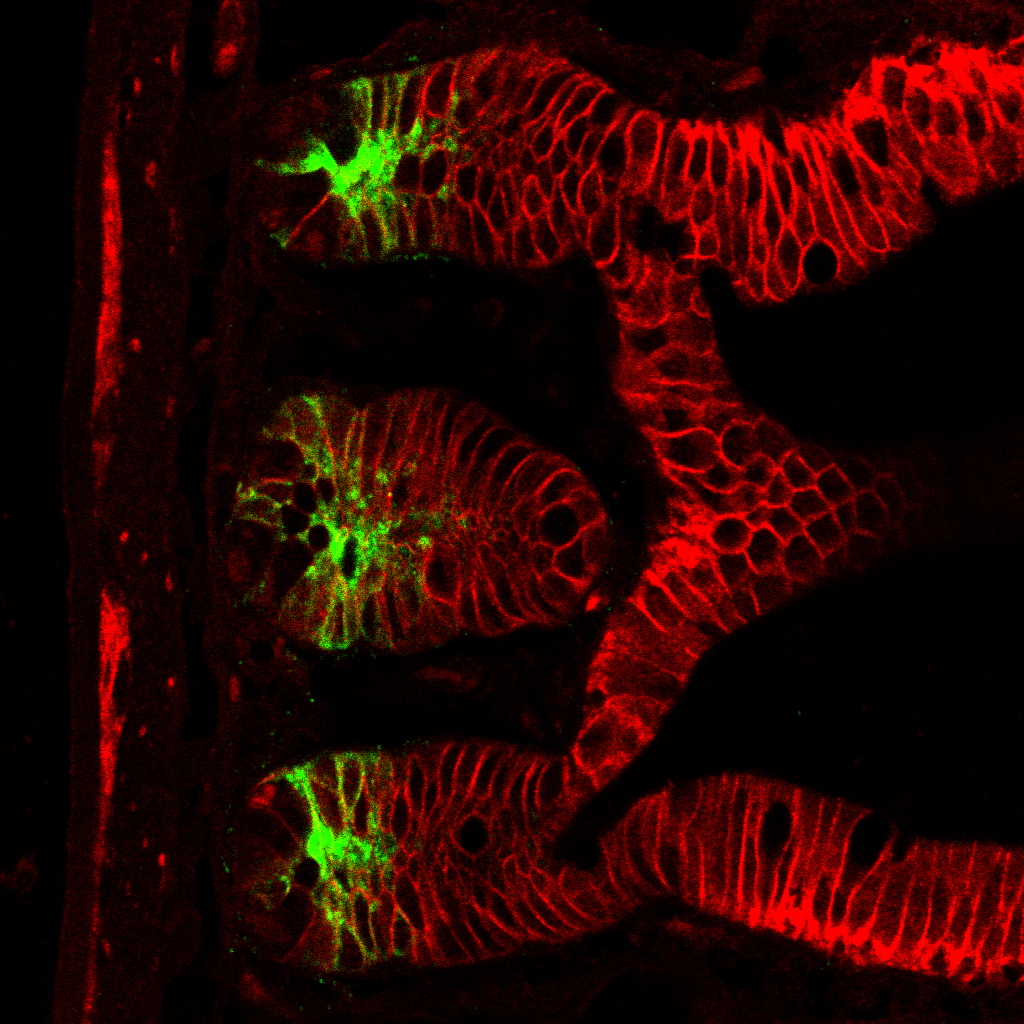

Supplement: Supplementary file 15 — Source Data for Figure 9 [file EMBR-24-e56030-s009.zip › Figure 9/Figure 9C -b-catenin OLFM4/1-2. PBS. 2 colors.tif]

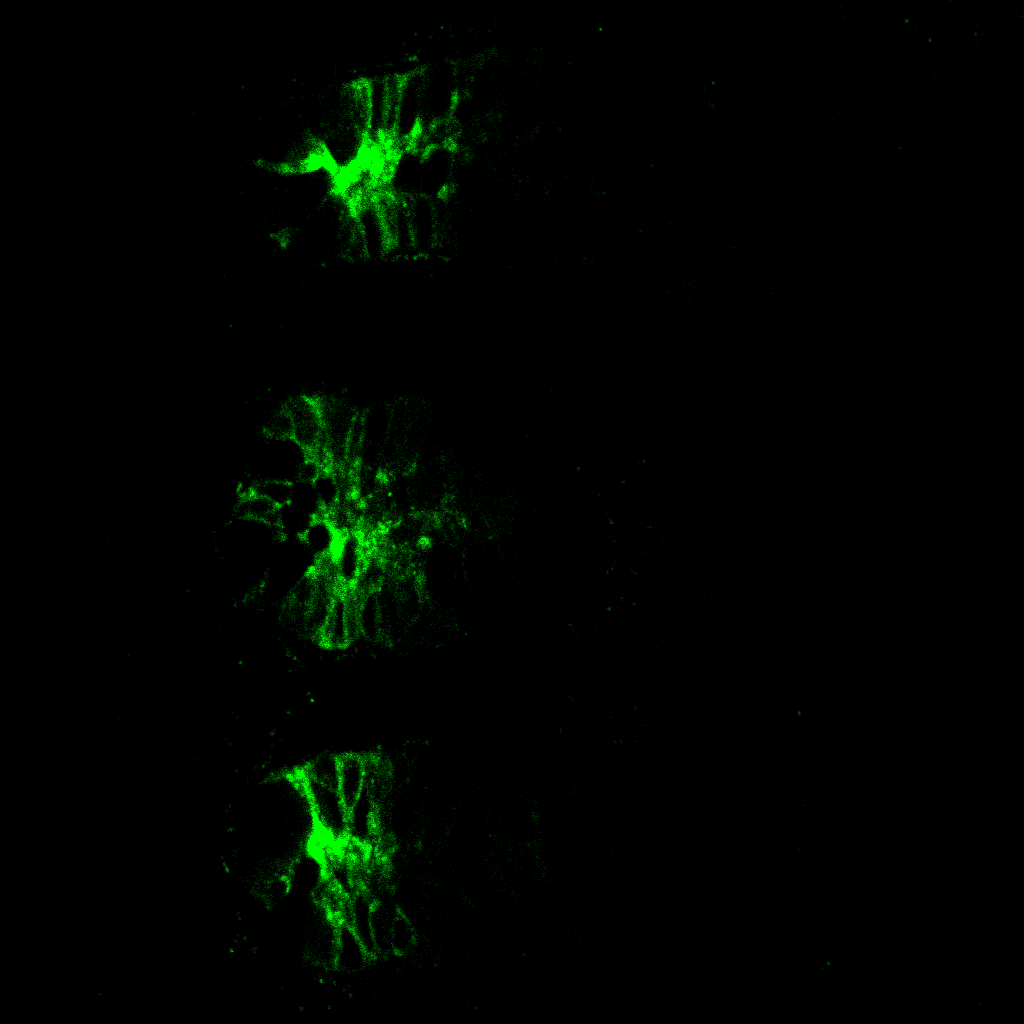

Supplement: Supplementary file 15 — Source Data for Figure 9 [file EMBR-24-e56030-s009.zip › Figure 9/Figure 9C -b-catenin OLFM4/1-3. PBS. OLFM4.tif]

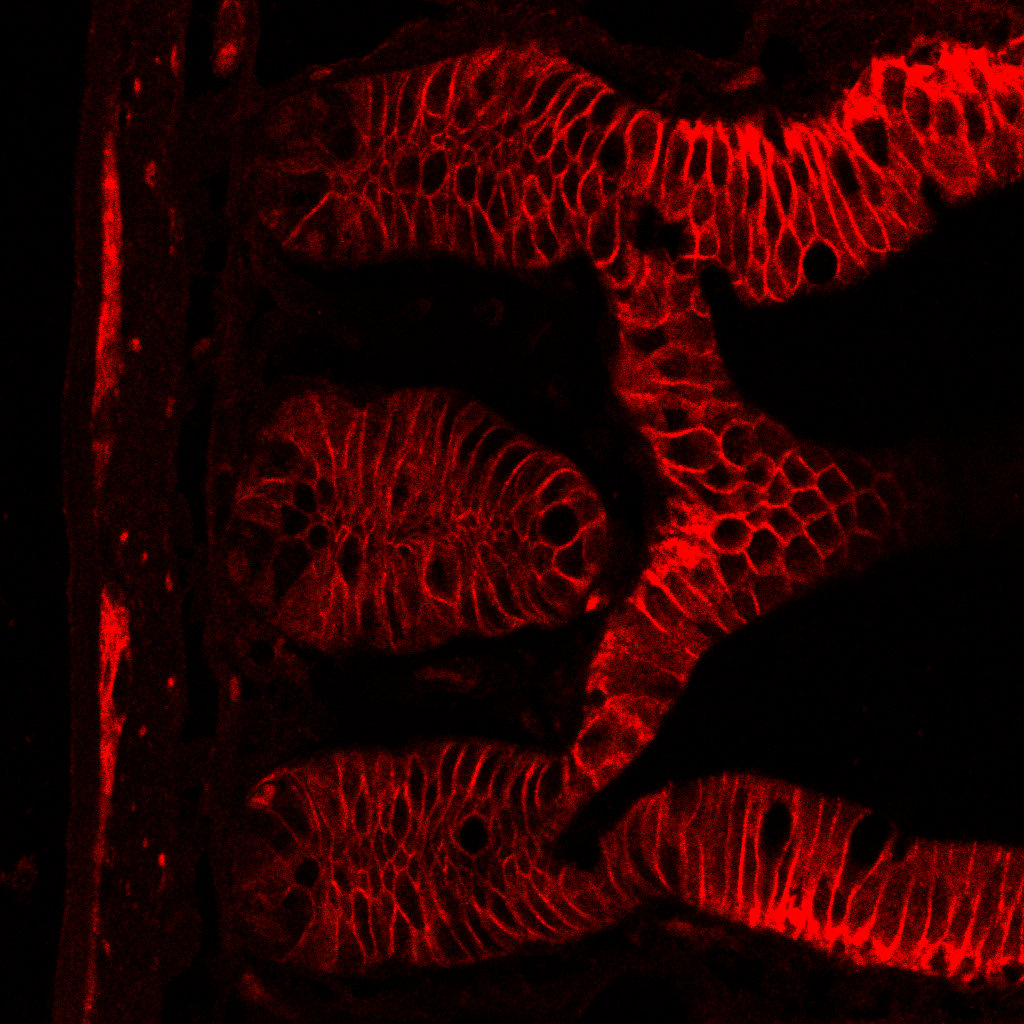

Supplement: Supplementary file 15 — Source Data for Figure 9 [file EMBR-24-e56030-s009.zip › Figure 9/Figure 9C -b-catenin OLFM4/1-4. RSPO3. b-catenin.tif]

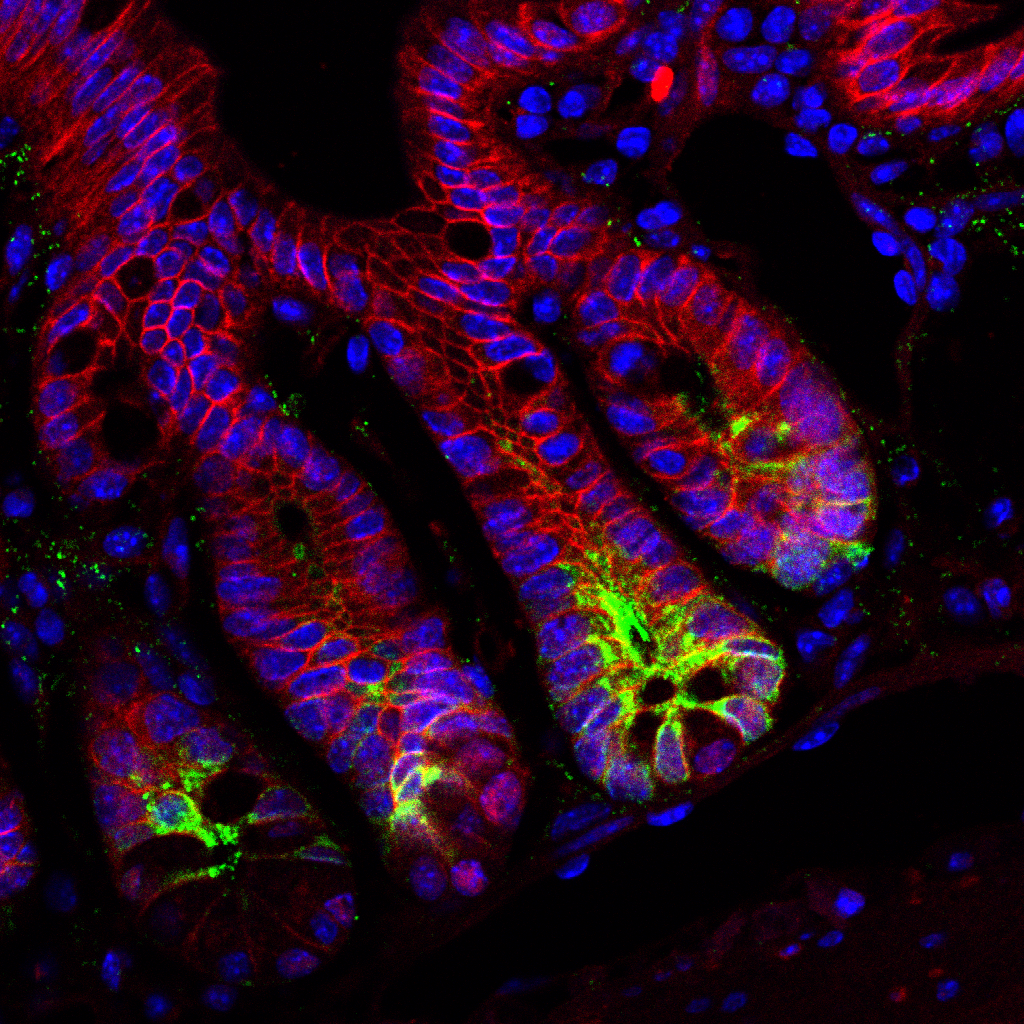

Supplement: Supplementary file 15 — Source Data for Figure 9 [file EMBR-24-e56030-s009.zip › Figure 9/Figure 9C -b-catenin OLFM4/2-1. RSPP3, 3 colors.tif]

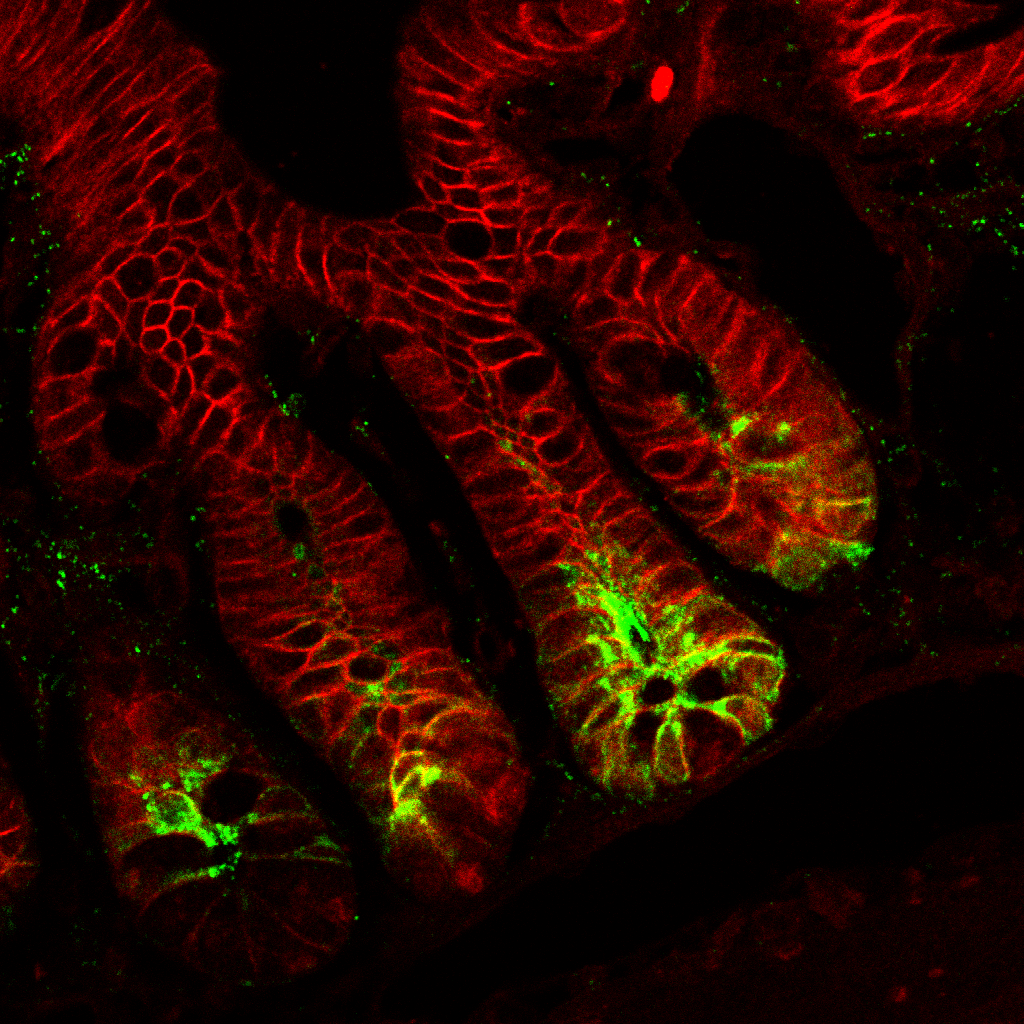

Supplement: Supplementary file 15 — Source Data for Figure 9 [file EMBR-24-e56030-s009.zip › Figure 9/Figure 9C -b-catenin OLFM4/2-2. RSPO3, 2 colors.tif]

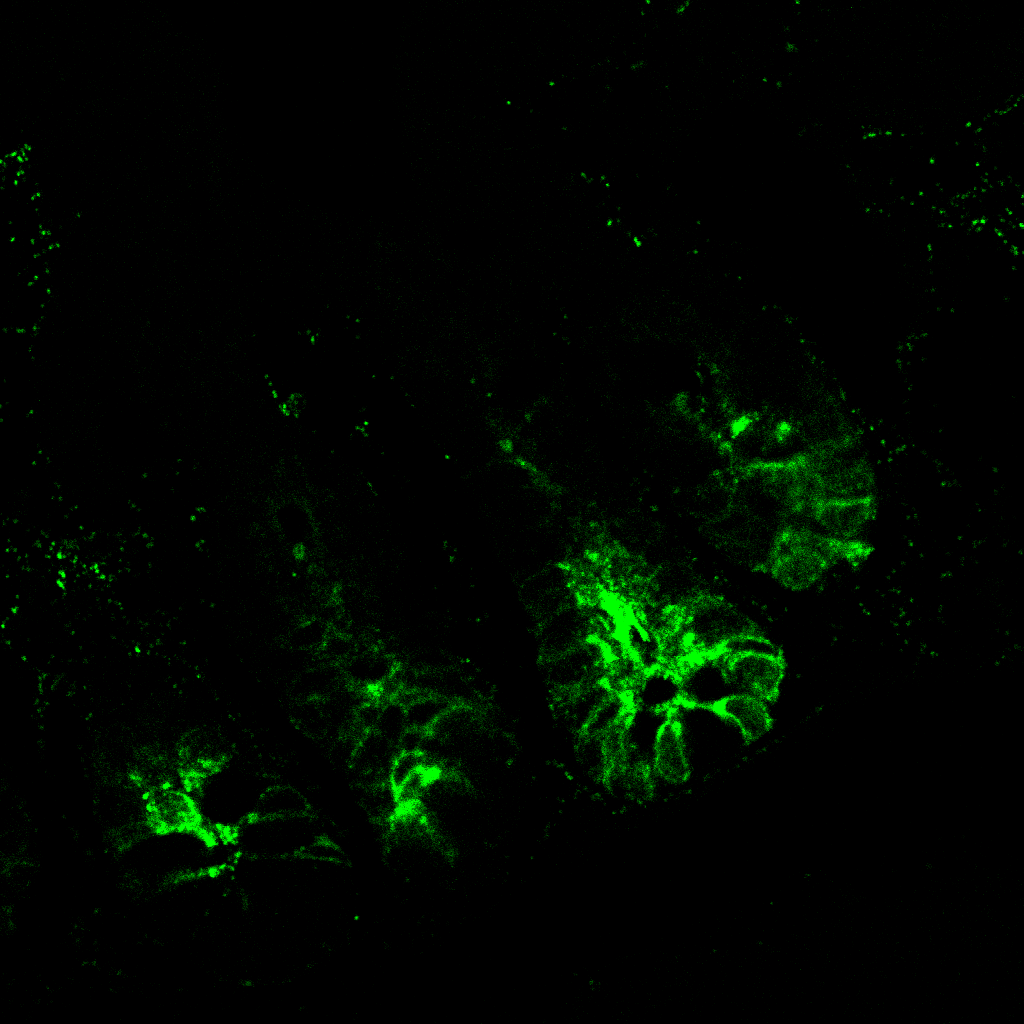

Supplement: Supplementary file 15 — Source Data for Figure 9 [file EMBR-24-e56030-s009.zip › Figure 9/Figure 9C -b-catenin OLFM4/2-3. RSPO3, OLFM4.tif]

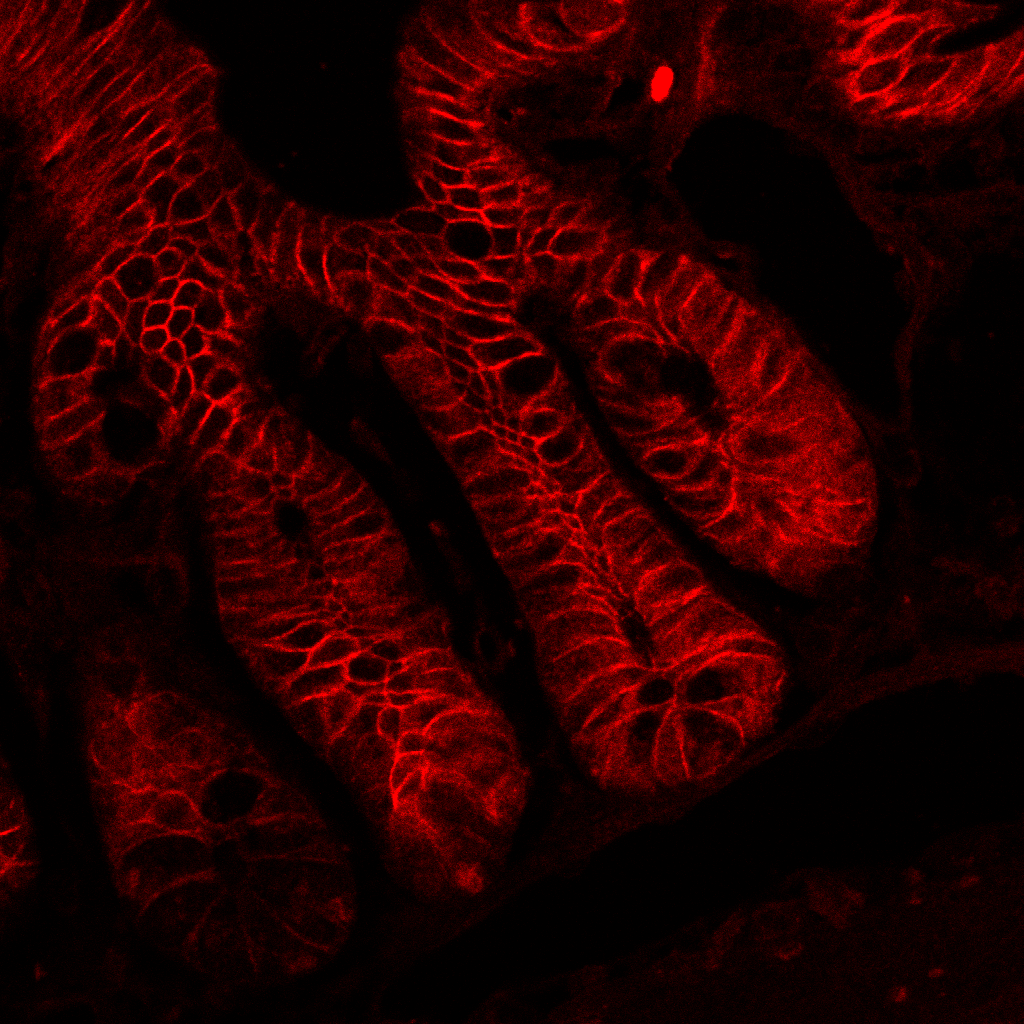

Supplement: Supplementary file 15 — Source Data for Figure 9 [file EMBR-24-e56030-s009.zip › Figure 9/Figure 9C -b-catenin OLFM4/2-4. RSPO3, b-catenin.tif]

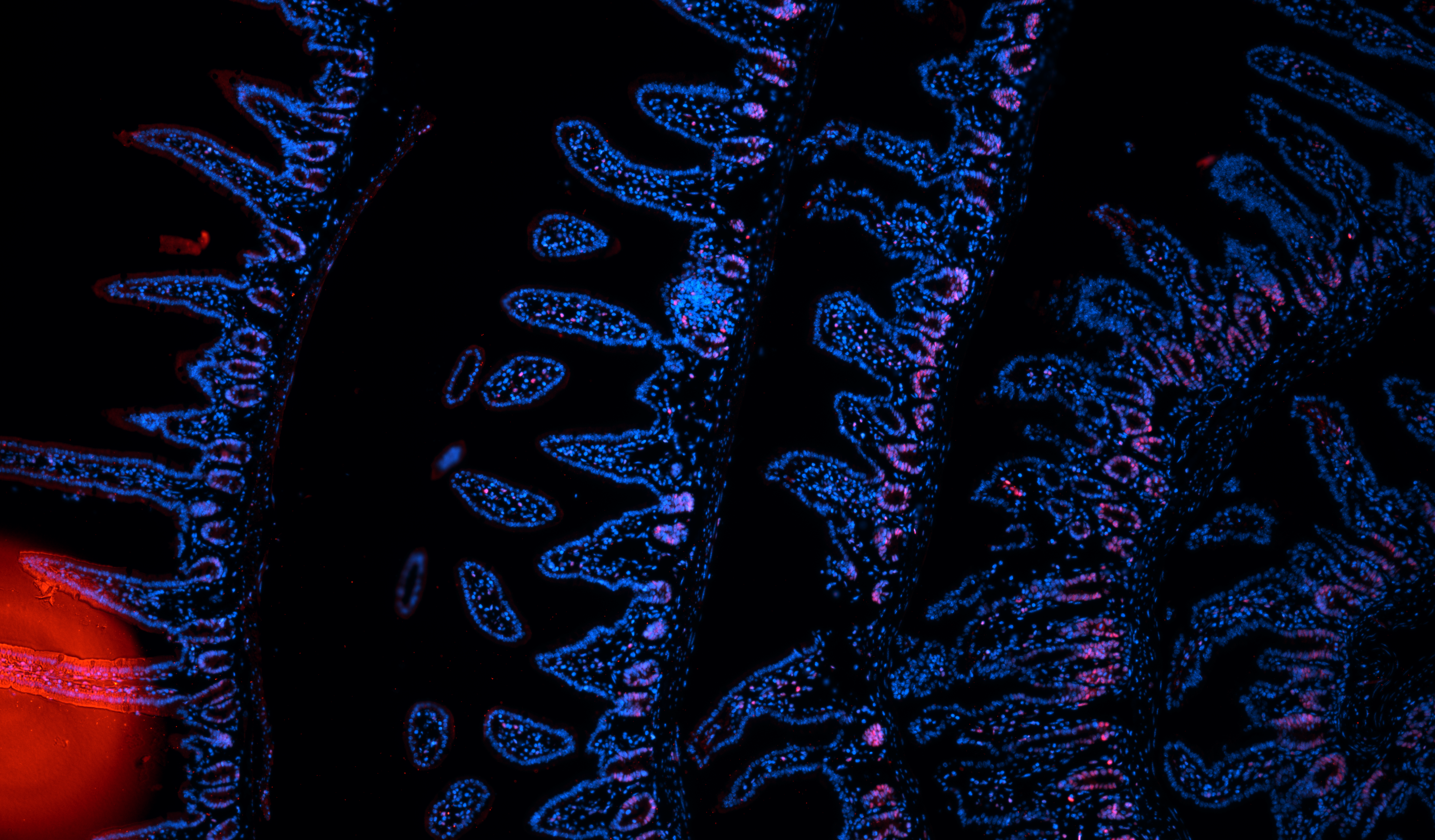

Supplement: Supplementary file 15 — Source Data for Figure 9 [file EMBR-24-e56030-s009.zip › Figure 9/Figure 9F-IHC-CCDN1/1.PBS.tif]

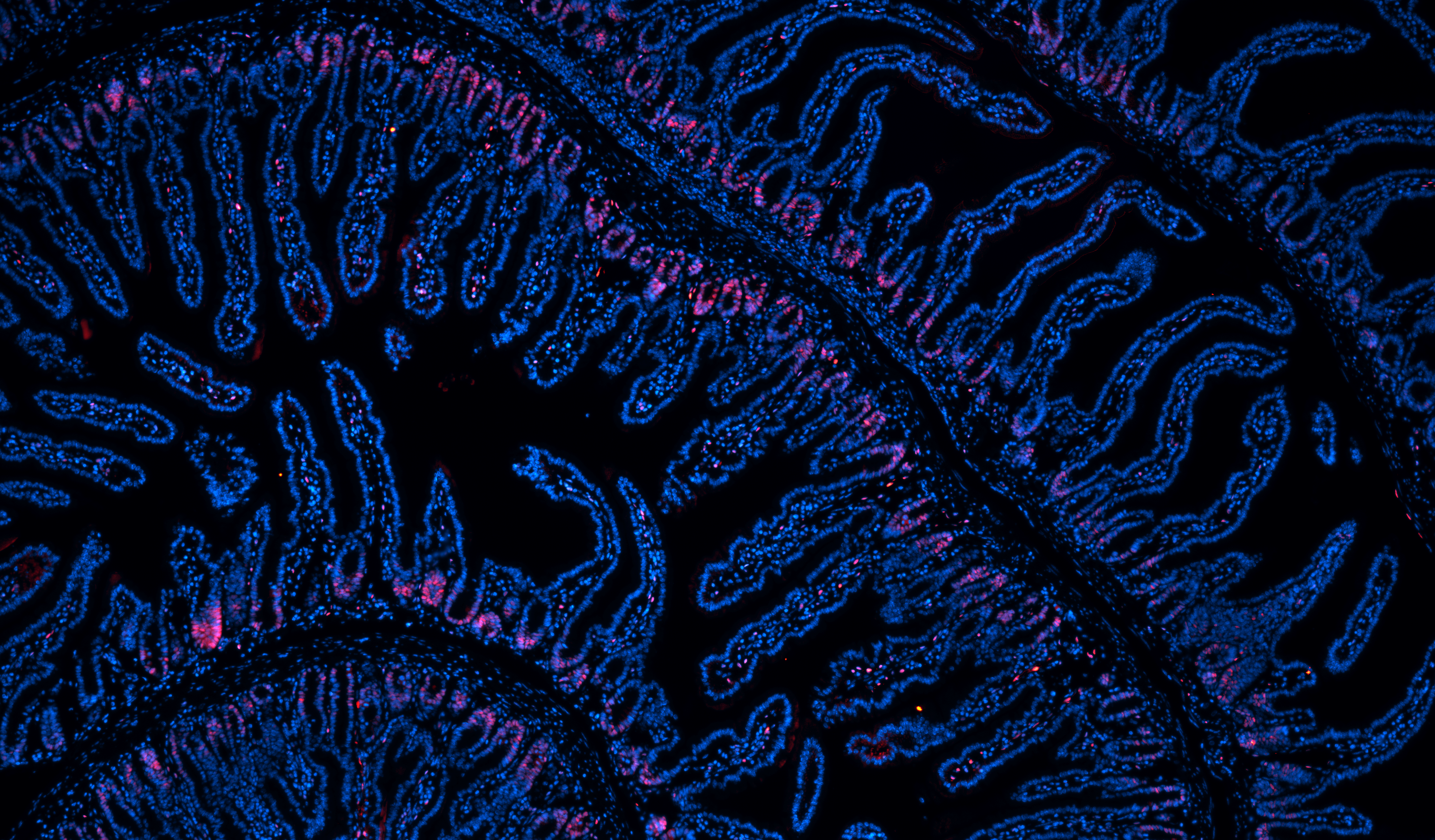

Supplement: Supplementary file 15 — Source Data for Figure 9 [file EMBR-24-e56030-s009.zip › Figure 9/Figure 9F-IHC-CCDN1/2. RSPO3.tif]

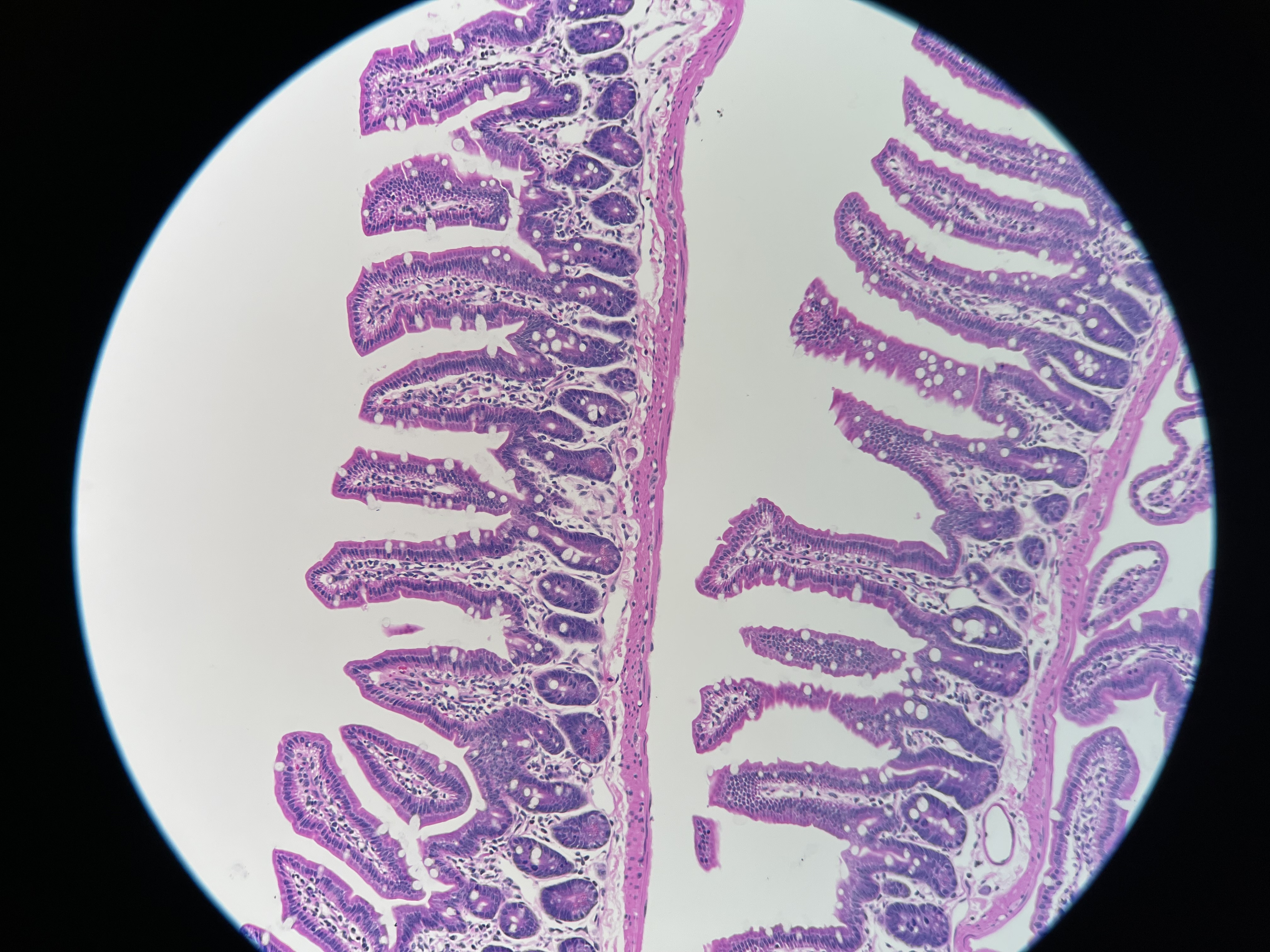

Supplement: Supplementary file 15 — Source Data for Figure 9 [file EMBR-24-e56030-s009.zip › Figure 9/Figure 9I-HE (LEC-specific line)/1. Control, PBS.jpeg]

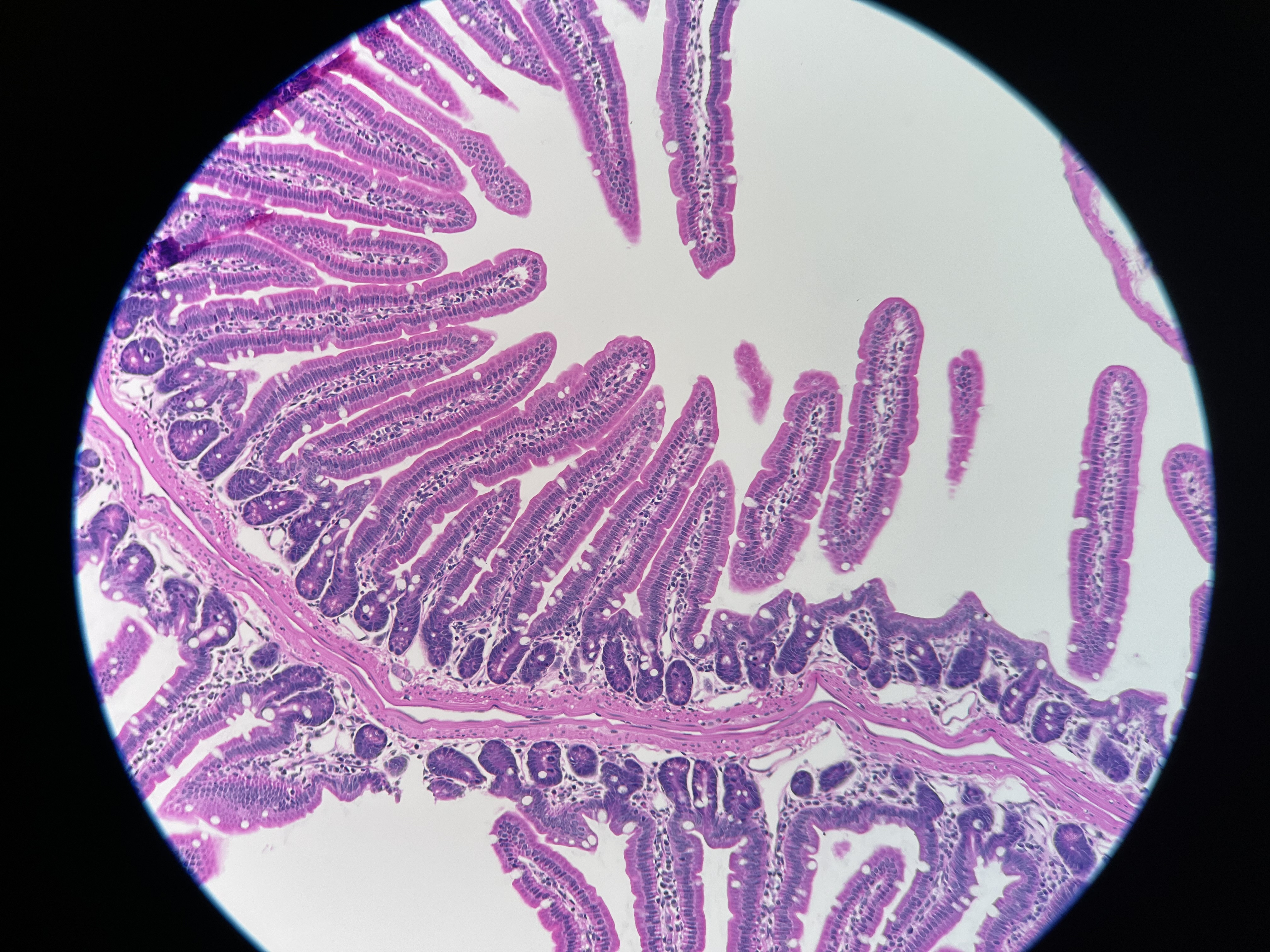

Supplement: Supplementary file 15 — Source Data for Figure 9 [file EMBR-24-e56030-s009.zip › Figure 9/Figure 9I-HE (LEC-specific line)/2. Control, RSPO3.jpeg]

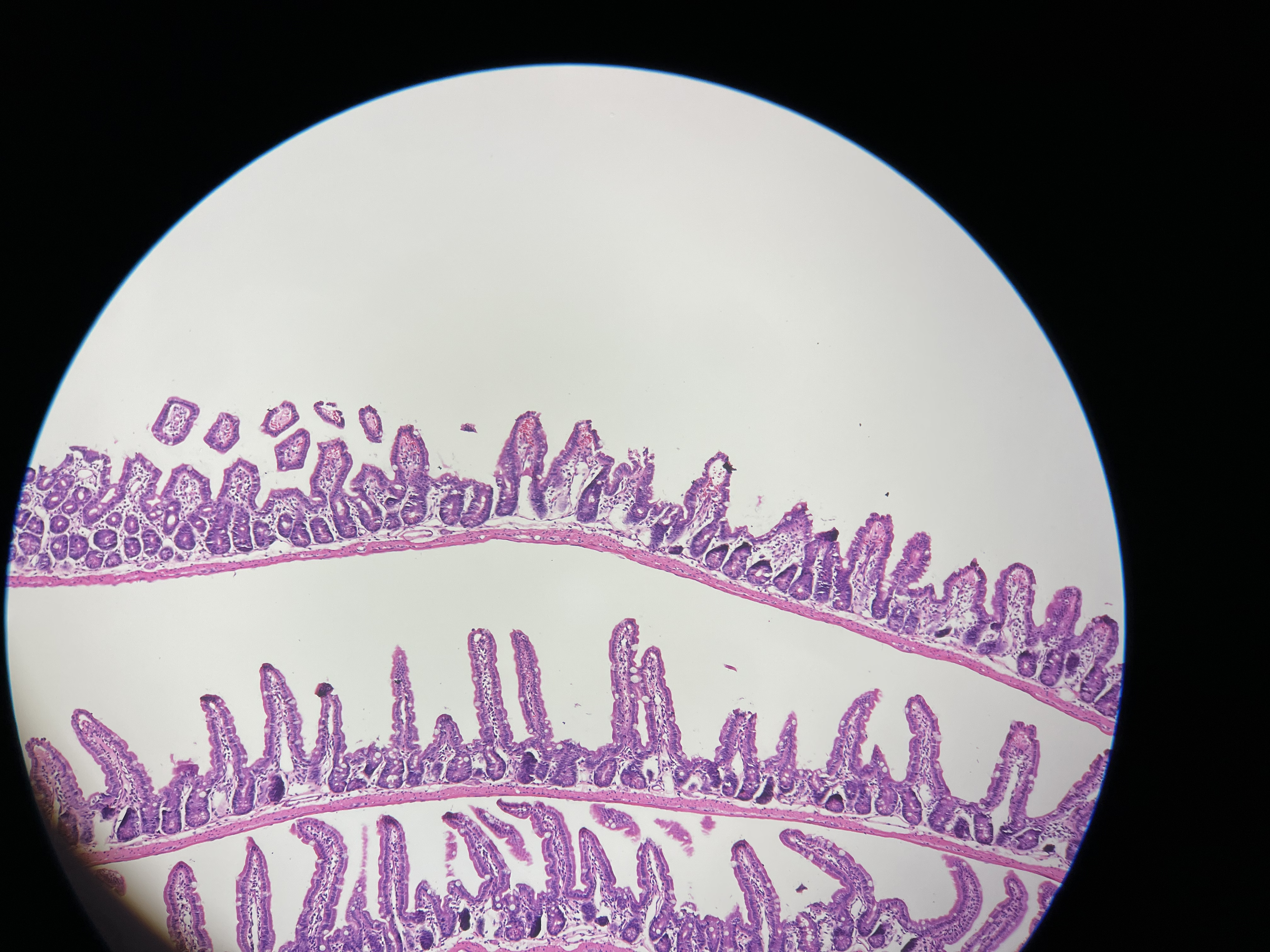

Supplement: Supplementary file 15 — Source Data for Figure 9 [file EMBR-24-e56030-s009.zip › Figure 9/Figure 9I-HE (LEC-specific line)/3. LEC-Foxc-DKO, PBS.jpeg]

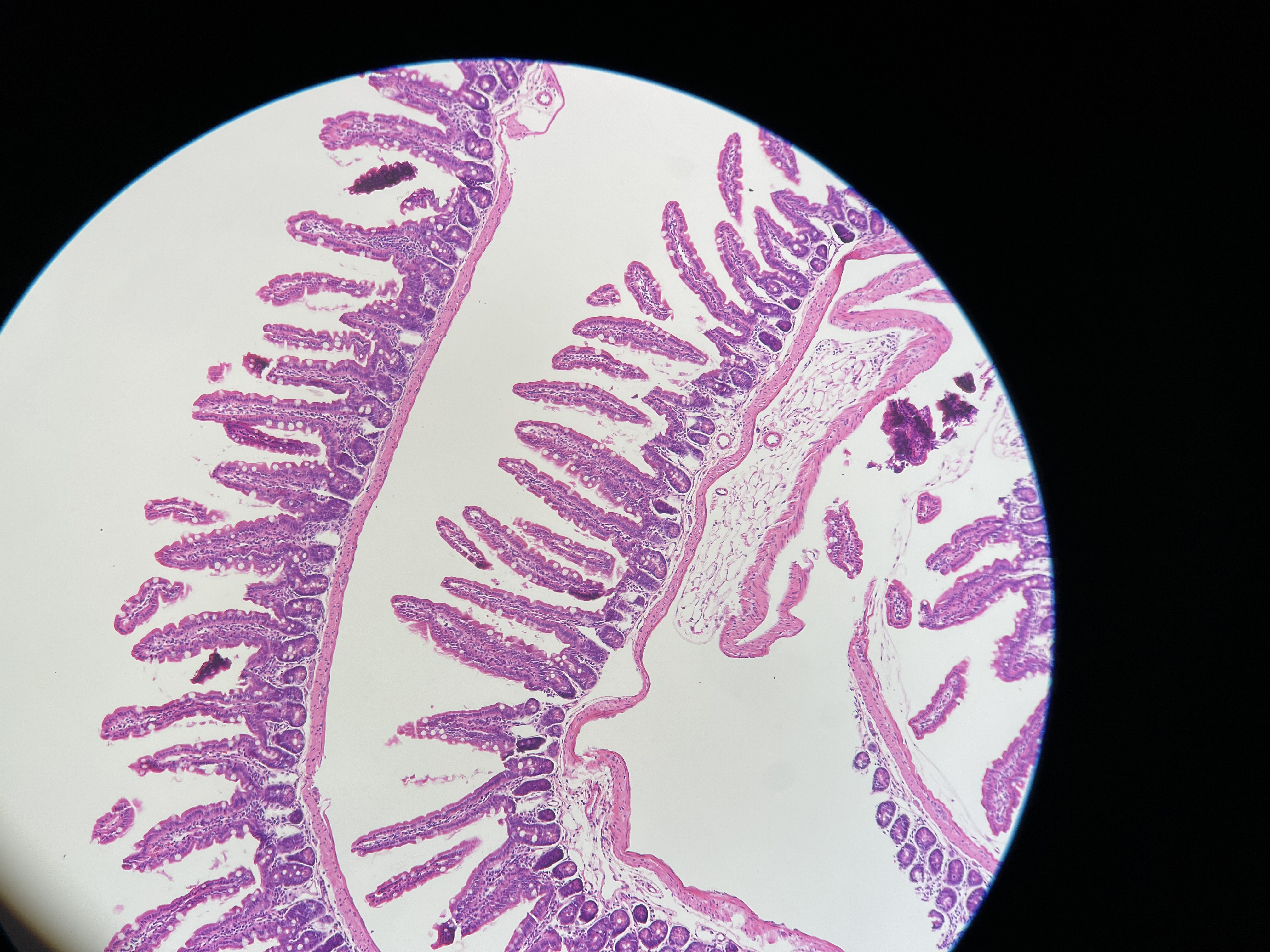

Supplement: Supplementary file 15 — Source Data for Figure 9 [file EMBR-24-e56030-s009.zip › Figure 9/Figure 9I-HE (LEC-specific line)/4. LEC-Foxc-DKO, RSPO3.jpeg]

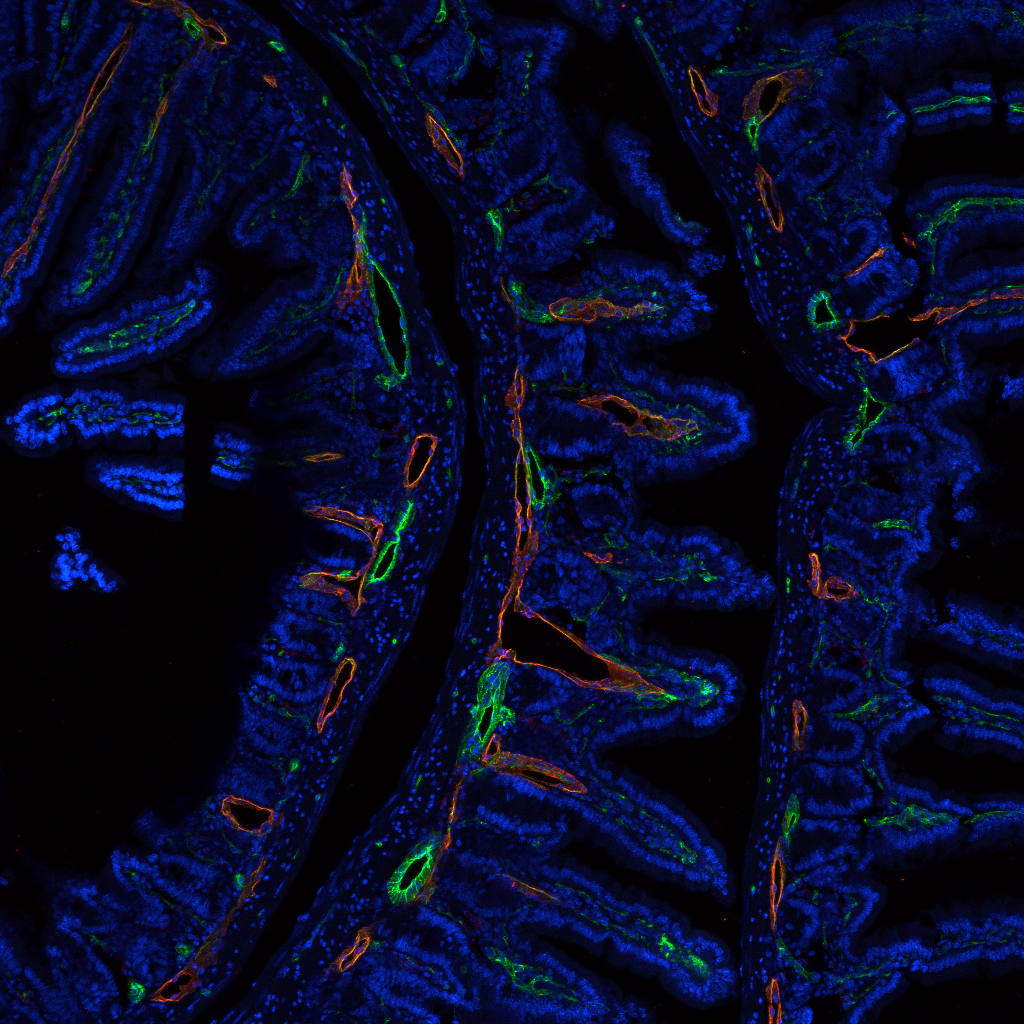

Supplement: Supplementary file 15 — Source Data for Figure 9 [file EMBR-24-e56030-s009.zip › Figure 9/Figure 9K-IHC-CD31 LYVE1/1. PBS.tif]

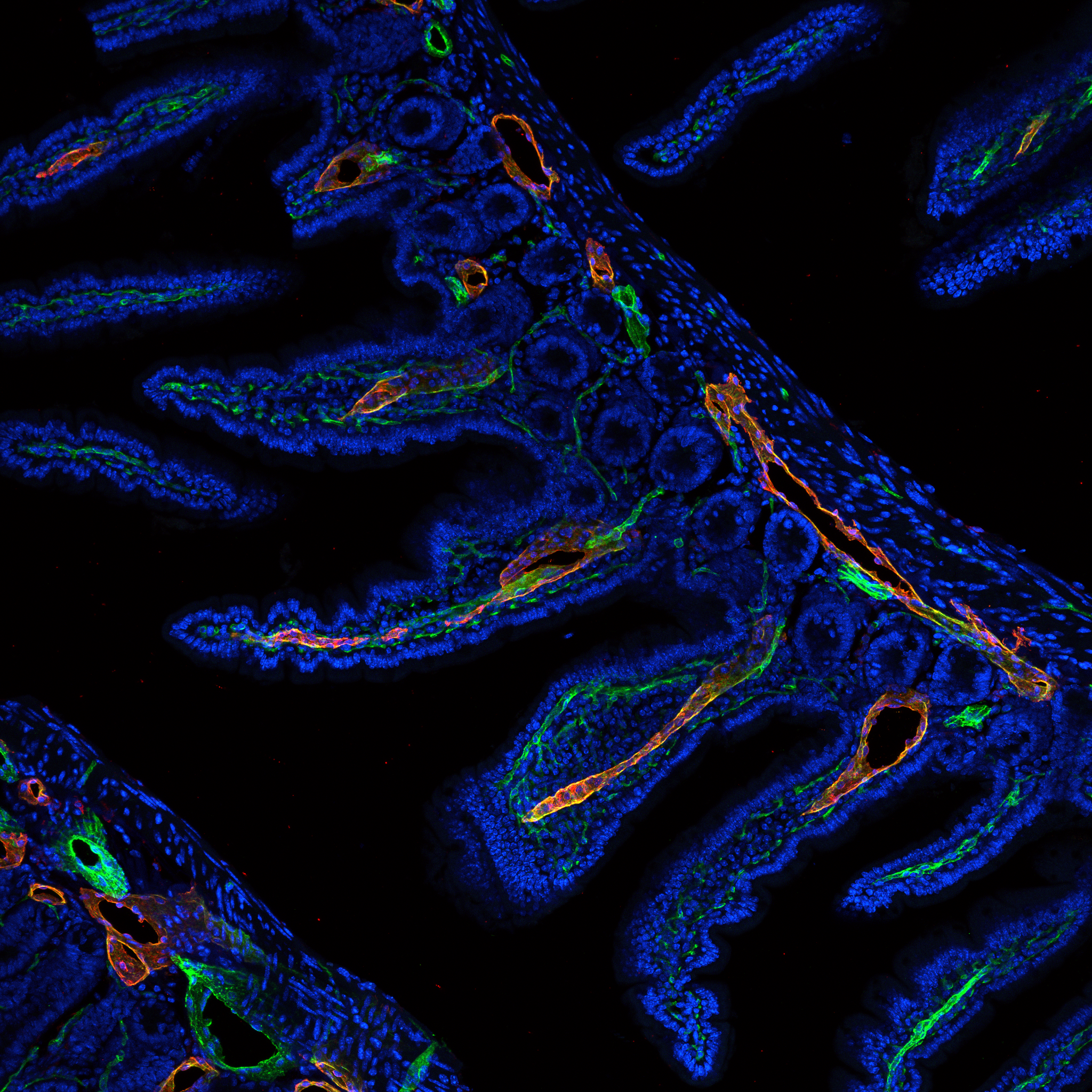

Supplement: Supplementary file 15 — Source Data for Figure 9 [file EMBR-24-e56030-s009.zip › Figure 9/Figure 9K-IHC-CD31 LYVE1/2. RSPO3.tif]

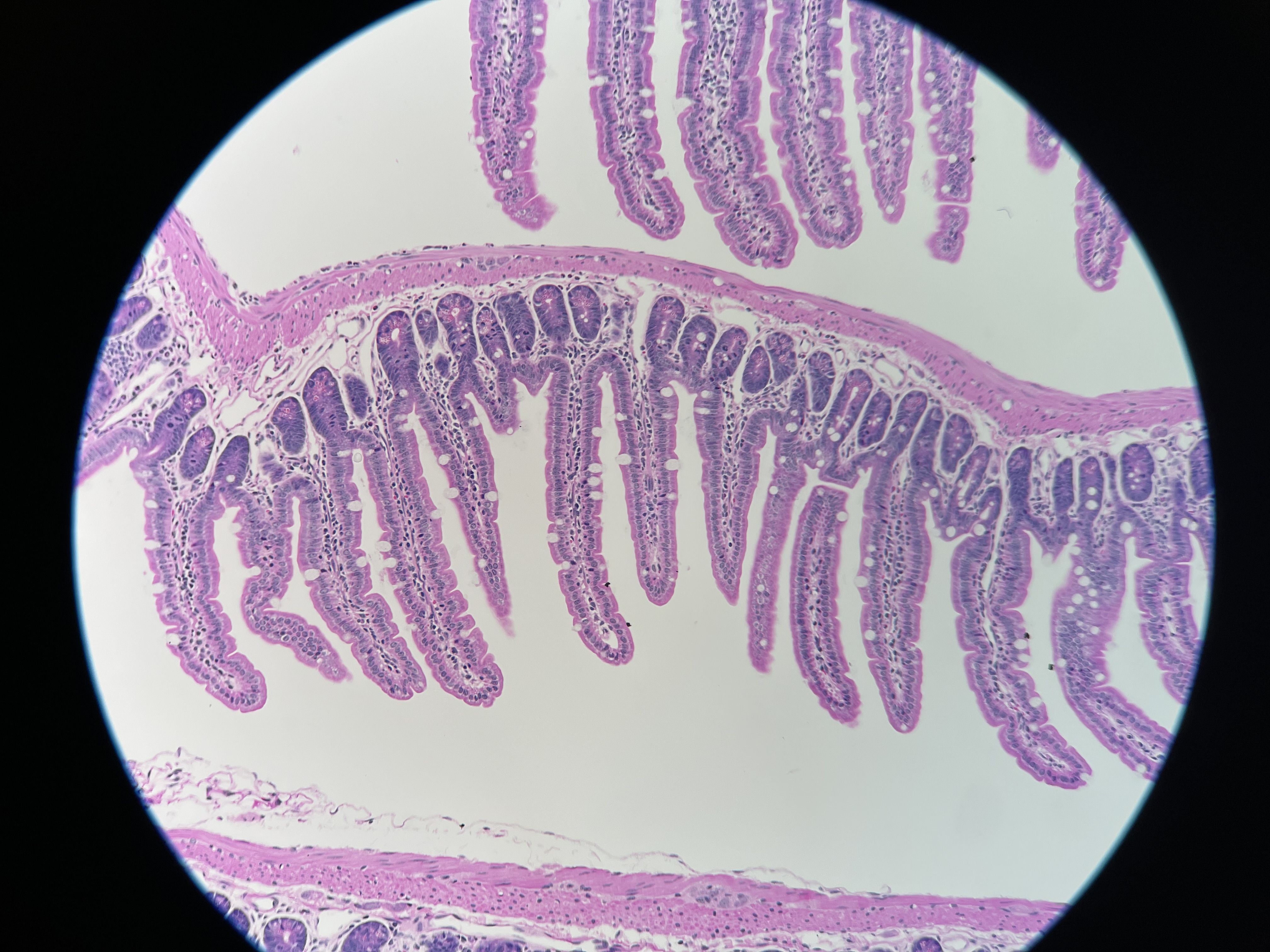

Supplement: Supplementary file 16 — Source Data for Figure 10 [file EMBR-24-e56030-s004.zip › Figure 10/Figure 10A-HE/1. PBS treated Control.jpeg]

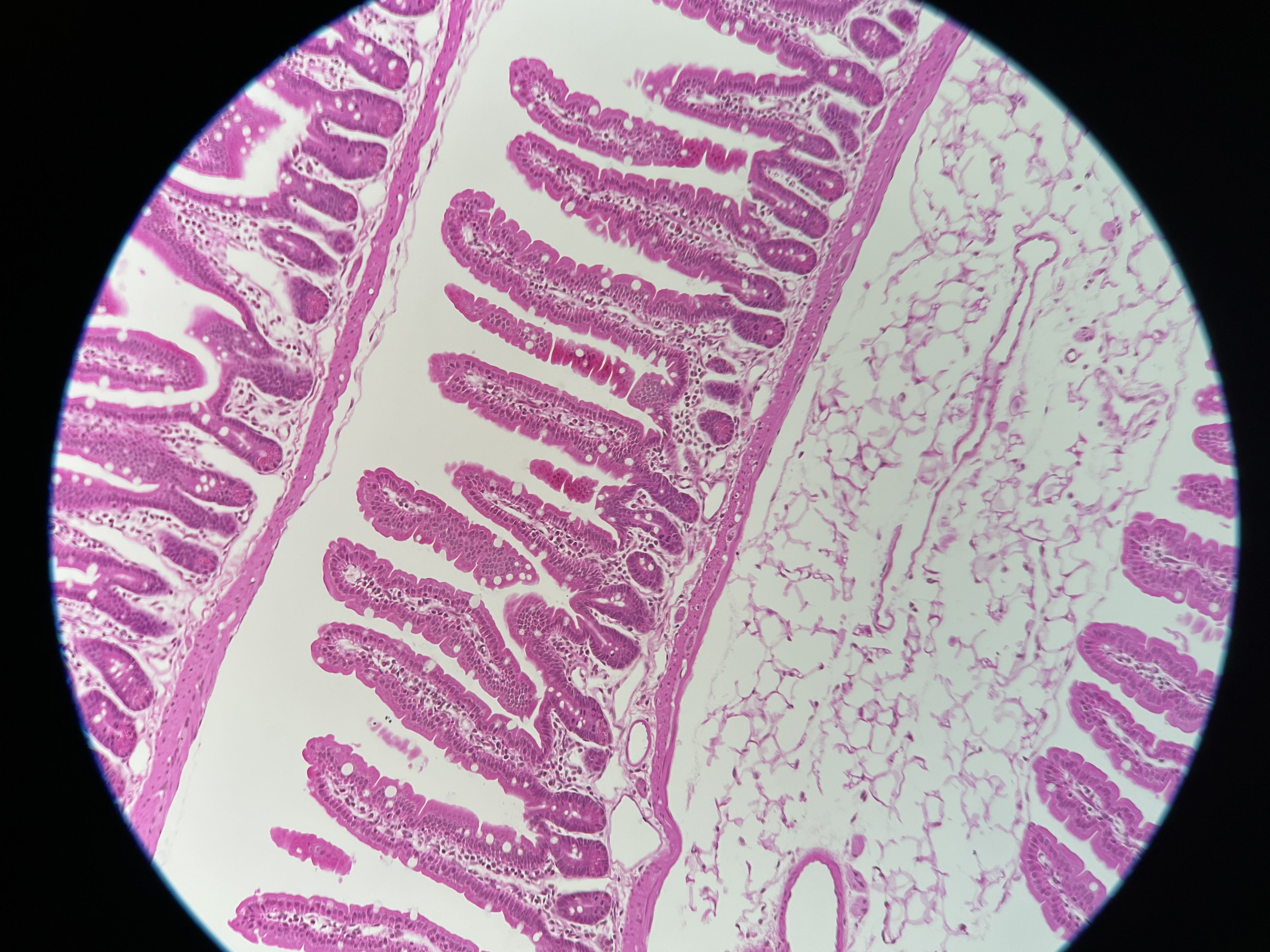

Supplement: Supplementary file 16 — Source Data for Figure 10 [file EMBR-24-e56030-s004.zip › Figure 10/Figure 10A-HE/2. CXCL12 treated Control.jpeg]

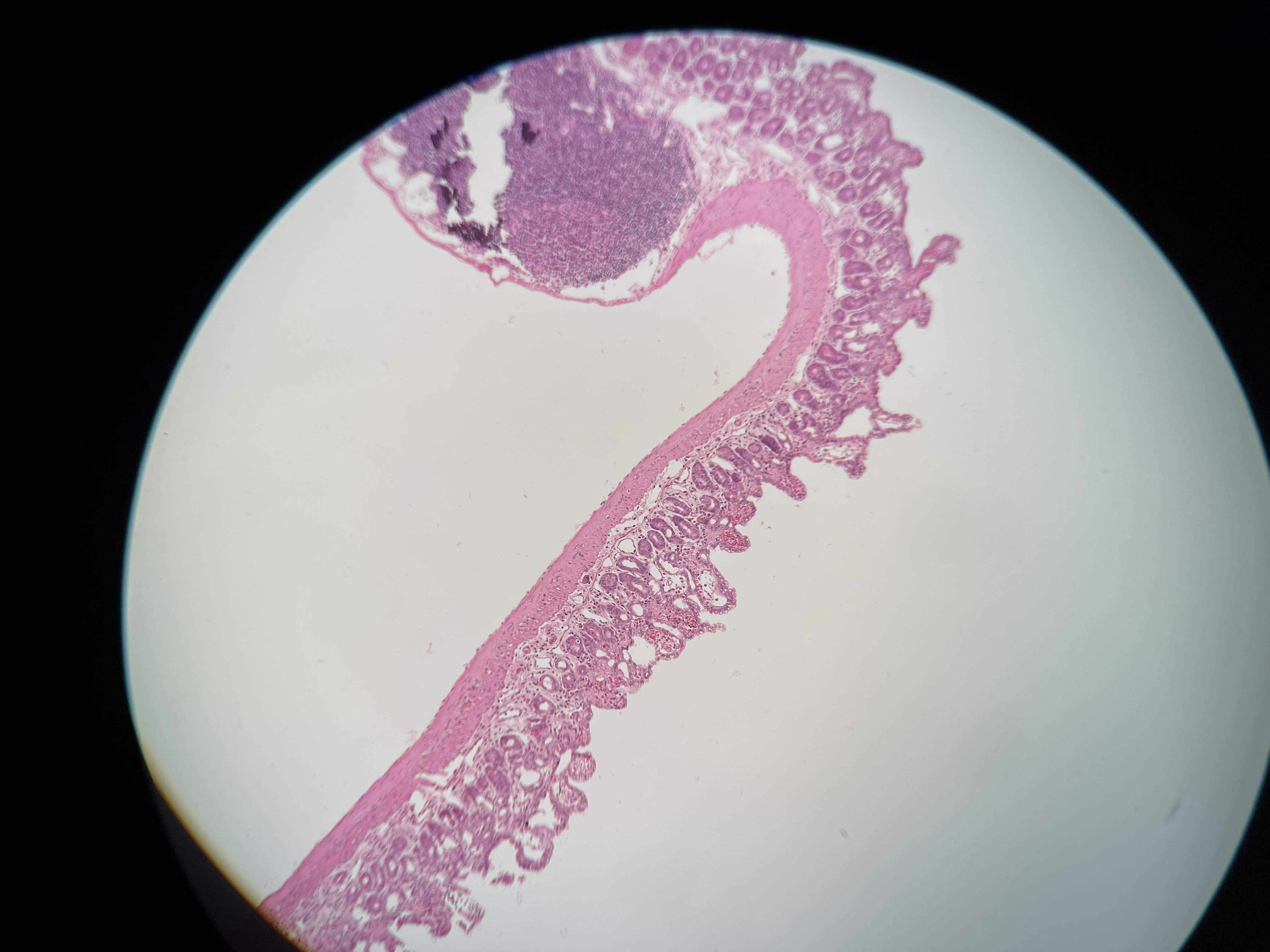

Supplement: Supplementary file 16 — Source Data for Figure 10 [file EMBR-24-e56030-s004.zip › Figure 10/Figure 10A-HE/3. PBS treated EC-Foxc-DKO.jpeg]

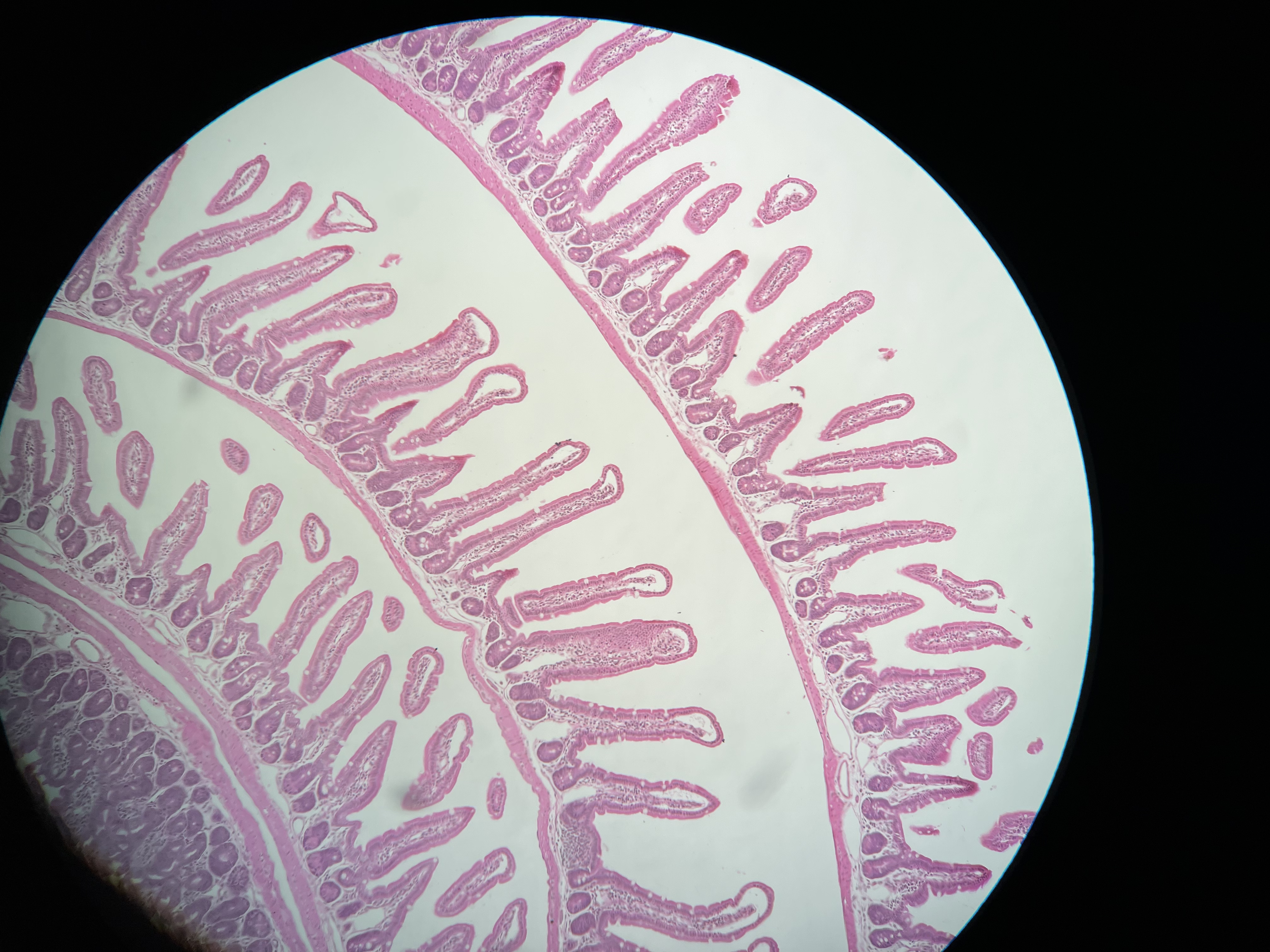

Supplement: Supplementary file 16 — Source Data for Figure 10 [file EMBR-24-e56030-s004.zip › Figure 10/Figure 10A-HE/4. CXCL12 treated EC-Foxc-DKO.jpeg]

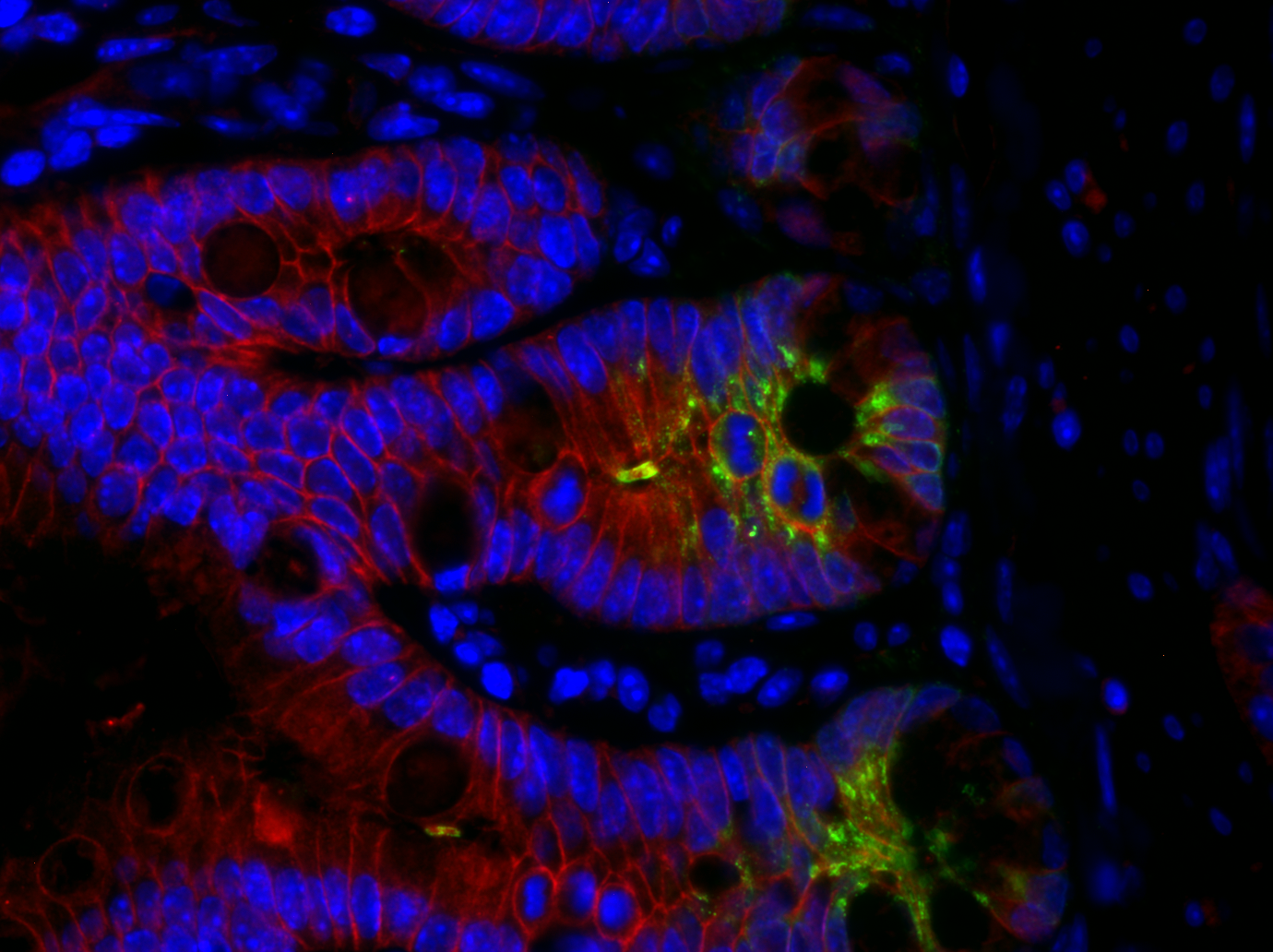

Supplement: Supplementary file 16 — Source Data for Figure 10 [file EMBR-24-e56030-s004.zip › Figure 10/Figure 10C-IHC-b-catenin, OLFM4/1-1. PBS treated, 3 colors.tif]

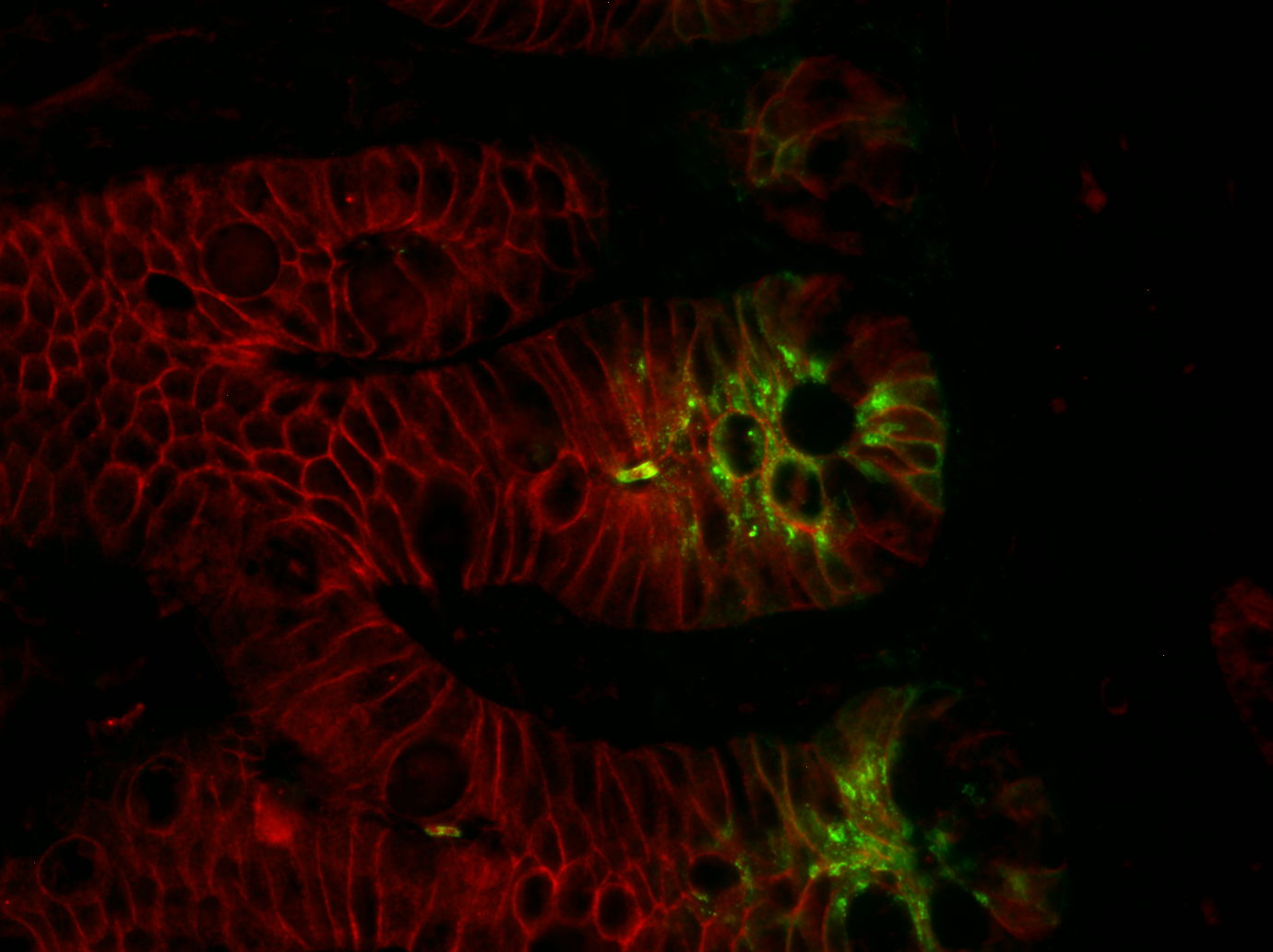

Supplement: Supplementary file 16 — Source Data for Figure 10 [file EMBR-24-e56030-s004.zip › Figure 10/Figure 10C-IHC-b-catenin, OLFM4/1-2. PBS treated, 2 colors.tif]

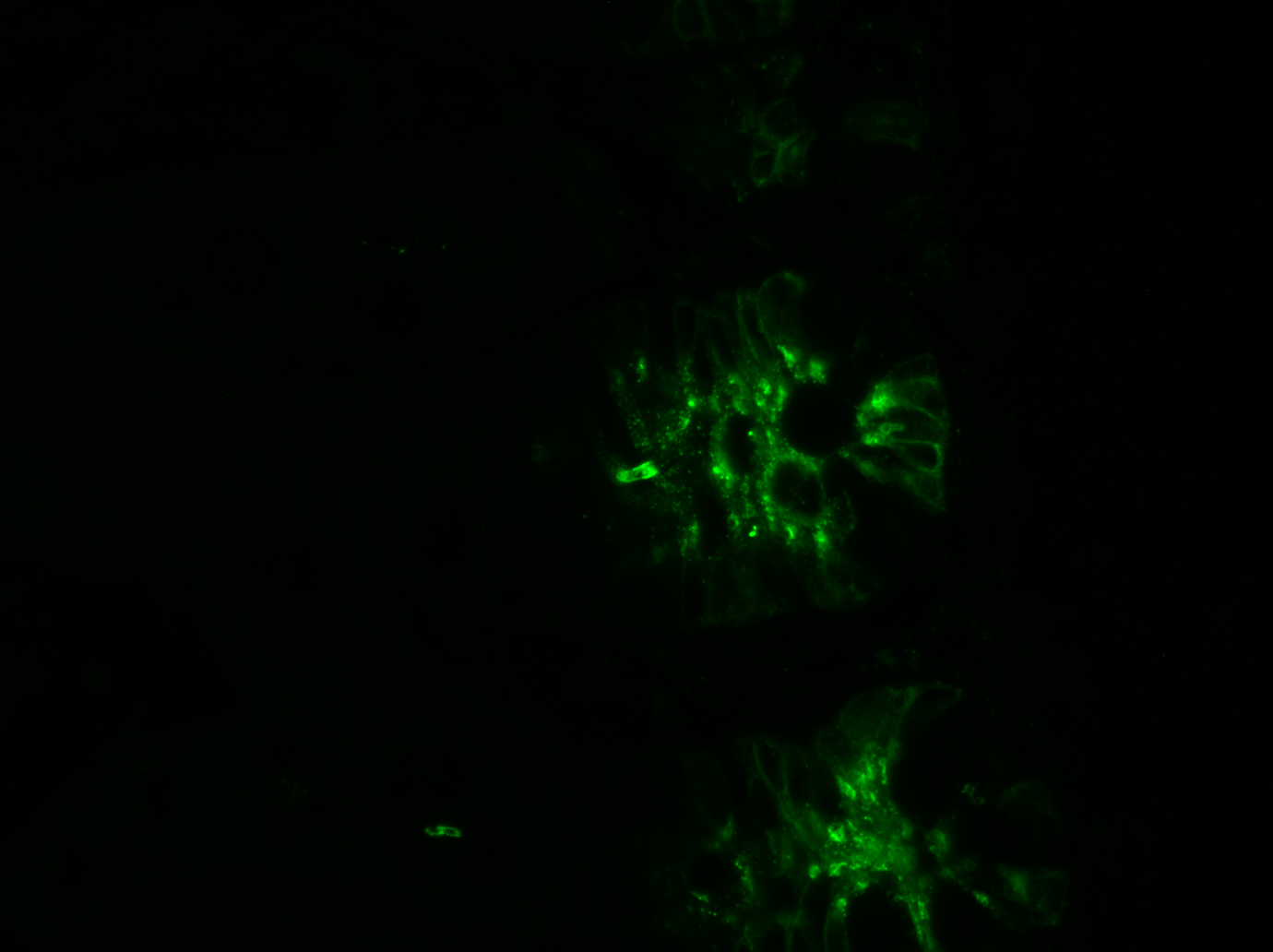

Supplement: Supplementary file 16 — Source Data for Figure 10 [file EMBR-24-e56030-s004.zip › Figure 10/Figure 10C-IHC-b-catenin, OLFM4/1-3. PBS treated, OLFM4.tif]

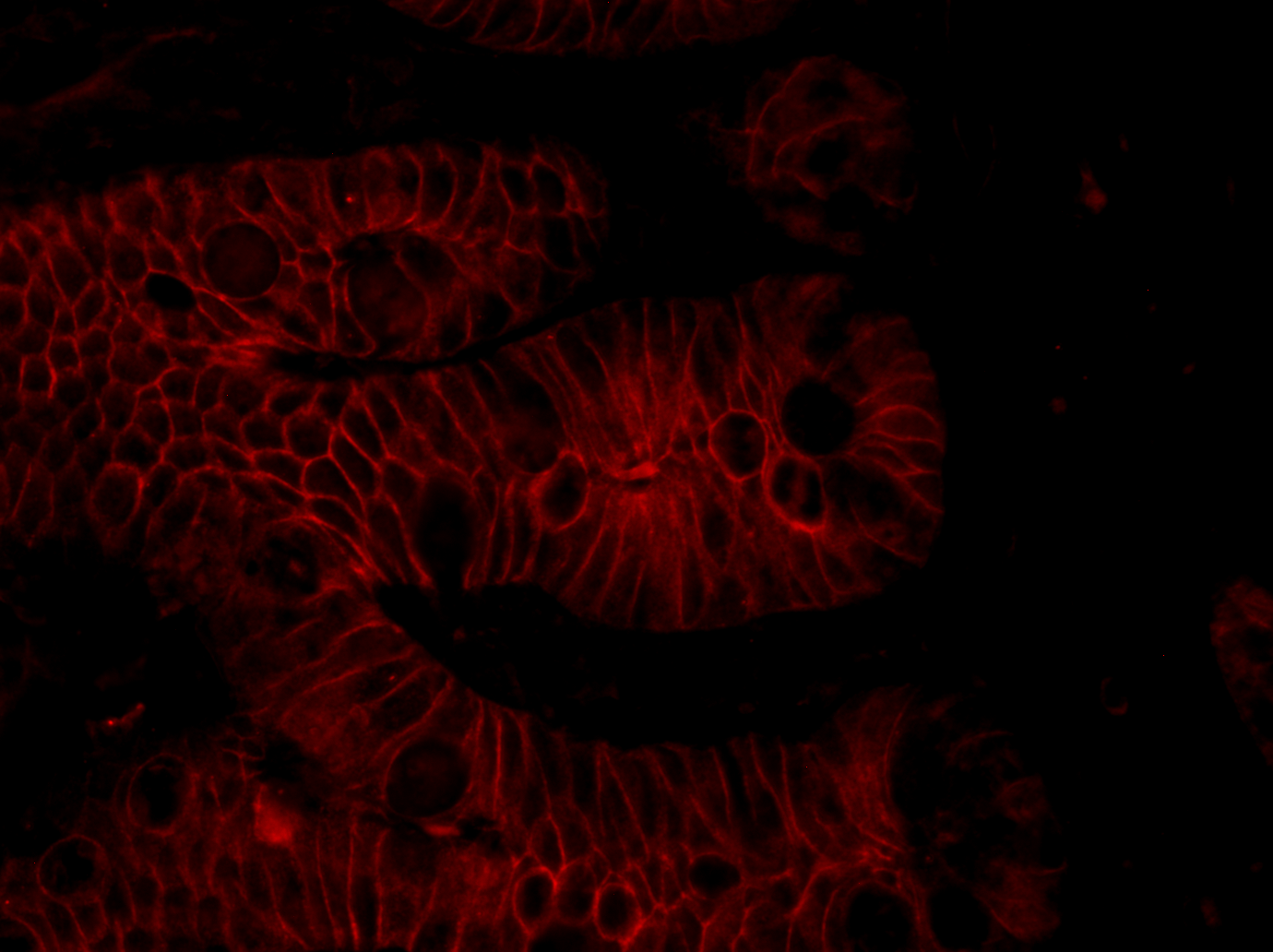

Supplement: Supplementary file 16 — Source Data for Figure 10 [file EMBR-24-e56030-s004.zip › Figure 10/Figure 10C-IHC-b-catenin, OLFM4/1-4. PBS treated, b-catenin.tif]

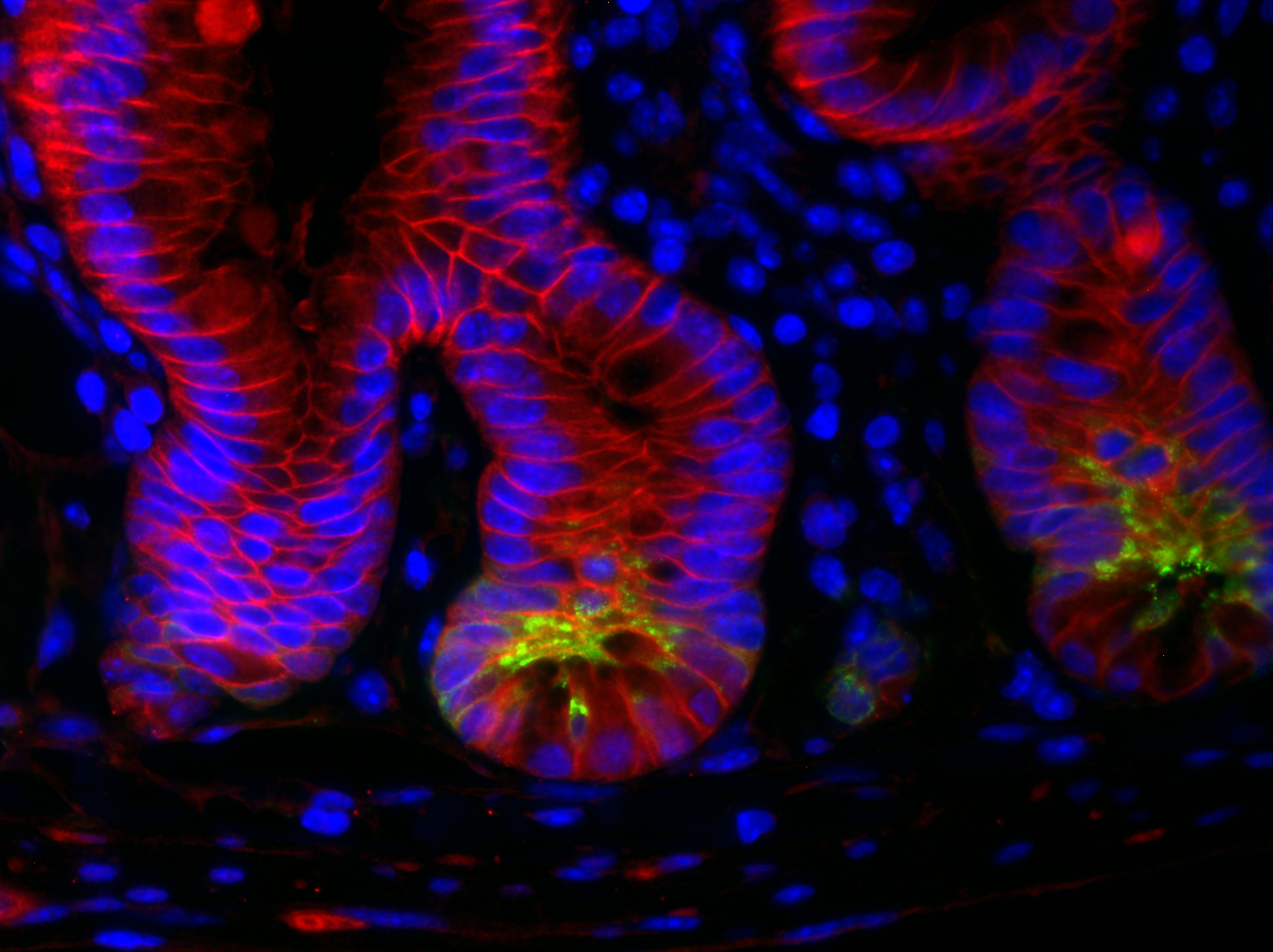

Supplement: Supplementary file 16 — Source Data for Figure 10 [file EMBR-24-e56030-s004.zip › Figure 10/Figure 10C-IHC-b-catenin, OLFM4/2-1. CXCL12 treated, 3 colors.tif]

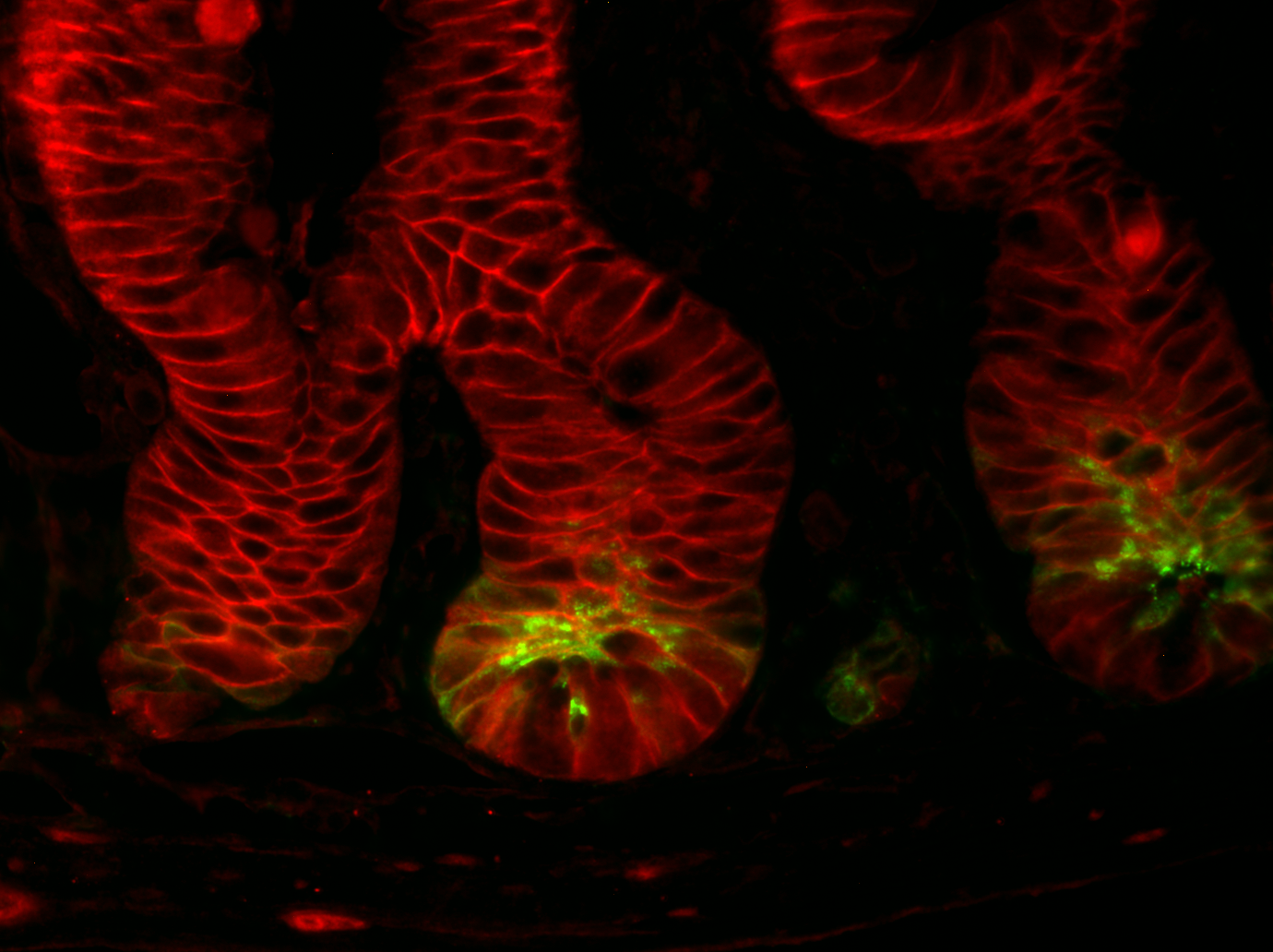

Supplement: Supplementary file 16 — Source Data for Figure 10 [file EMBR-24-e56030-s004.zip › Figure 10/Figure 10C-IHC-b-catenin, OLFM4/2-2. CXCL12 treated, 2 colors.tif]

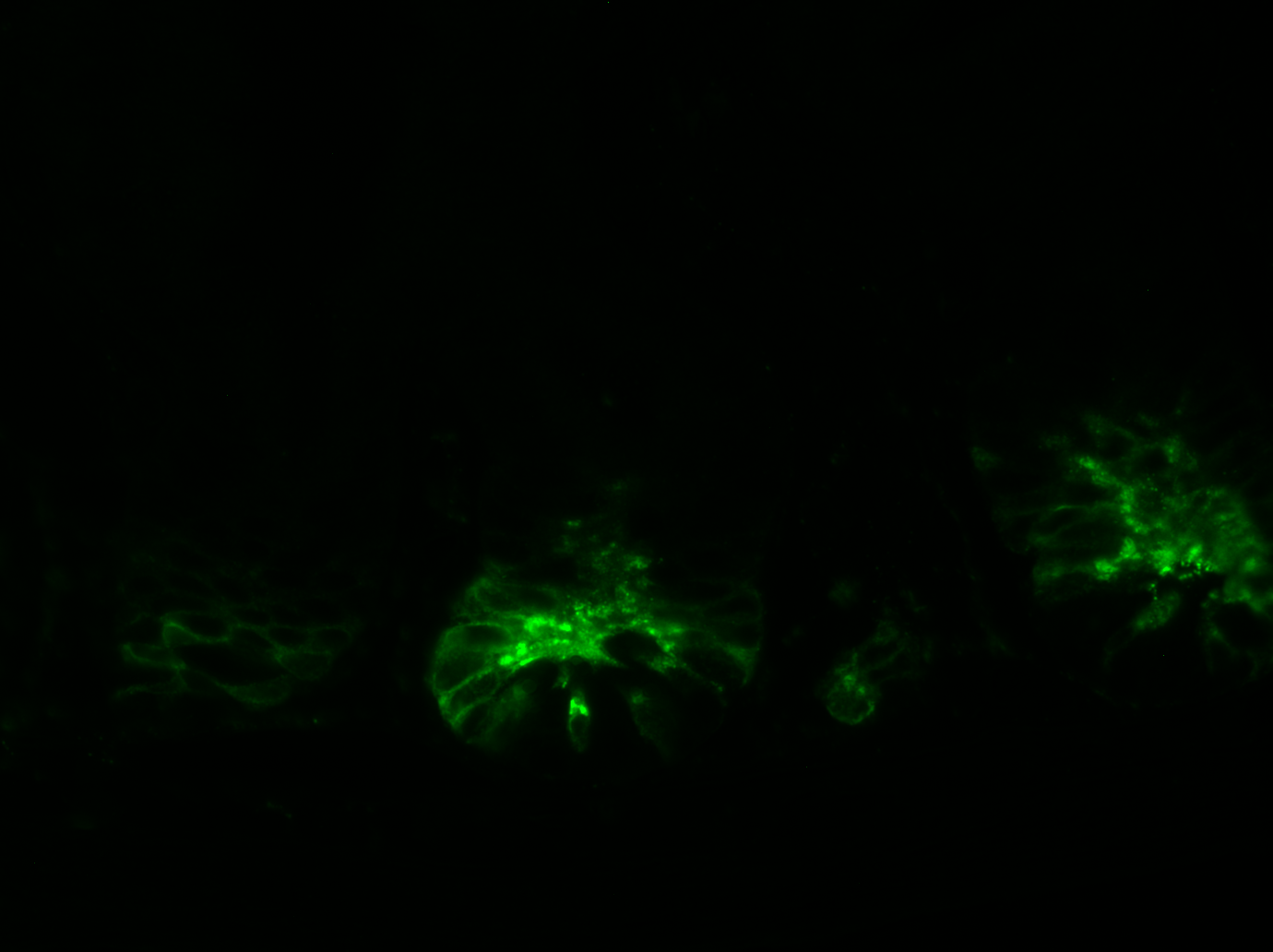

Supplement: Supplementary file 16 — Source Data for Figure 10 [file EMBR-24-e56030-s004.zip › Figure 10/Figure 10C-IHC-b-catenin, OLFM4/2-3. CXCL12 treated, OLFM4.tif]

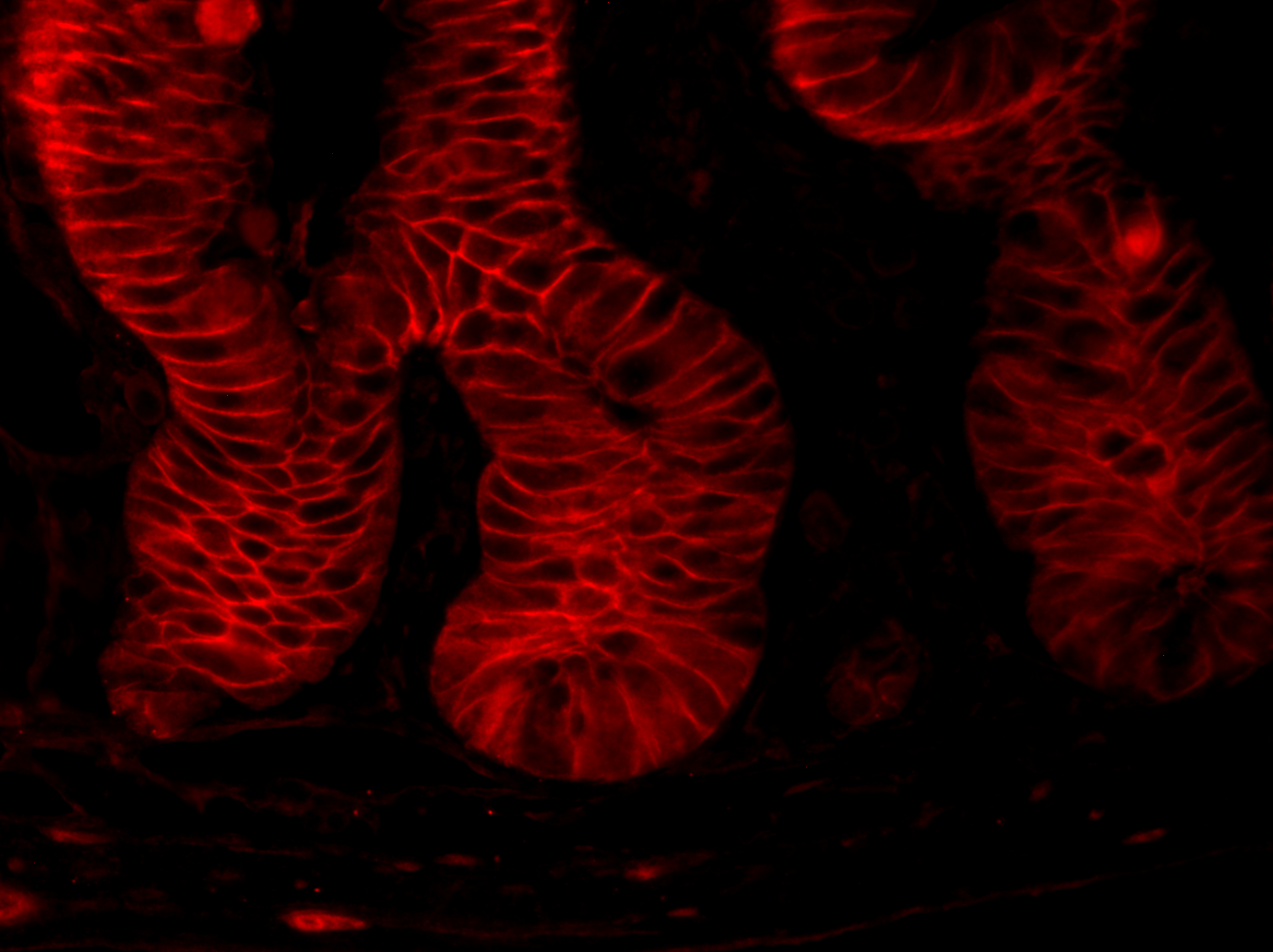

Supplement: Supplementary file 16 — Source Data for Figure 10 [file EMBR-24-e56030-s004.zip › Figure 10/Figure 10C-IHC-b-catenin, OLFM4/2-4. CXCL12 treated, b-catenin.tif]

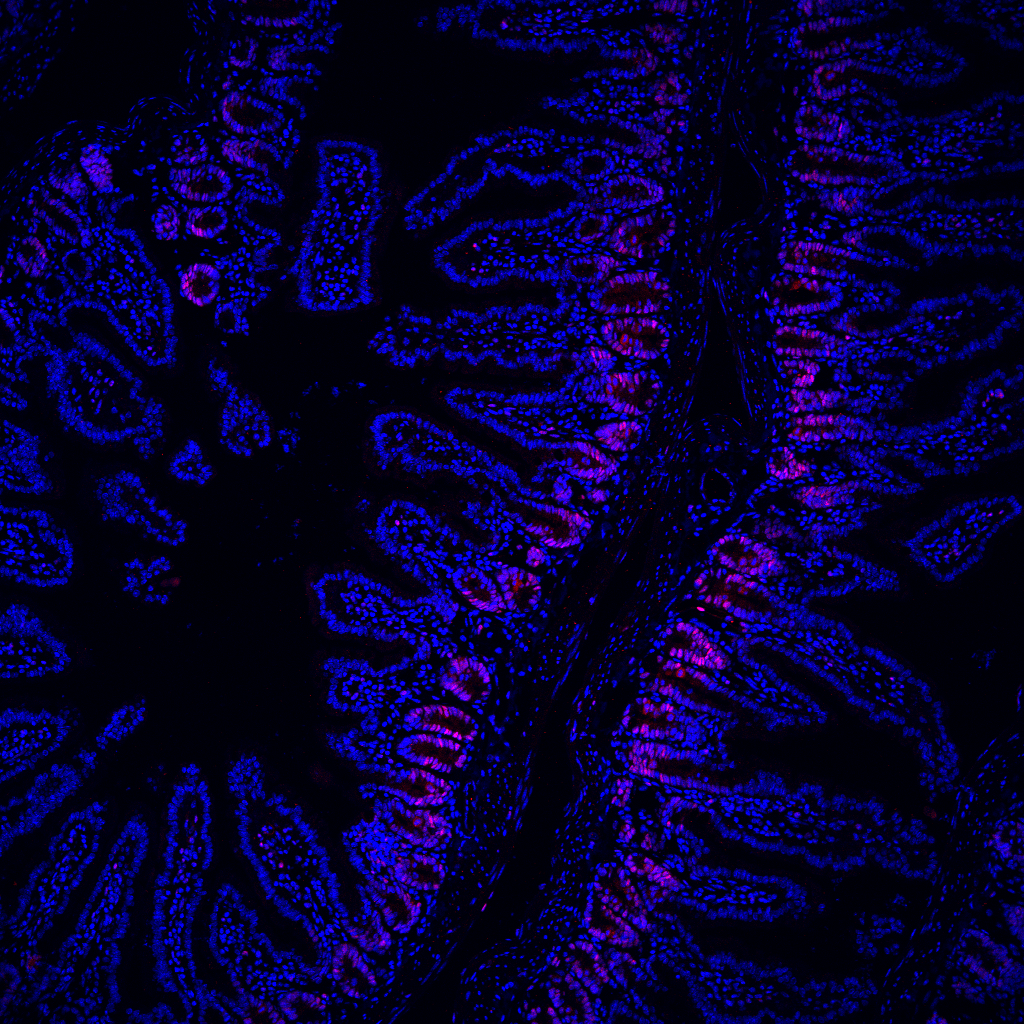

Supplement: Supplementary file 16 — Source Data for Figure 10 [file EMBR-24-e56030-s004.zip › Figure 10/Figure 10F-IHC-CCND1/1. PBS treated EC-Foxc-DKO.tif]

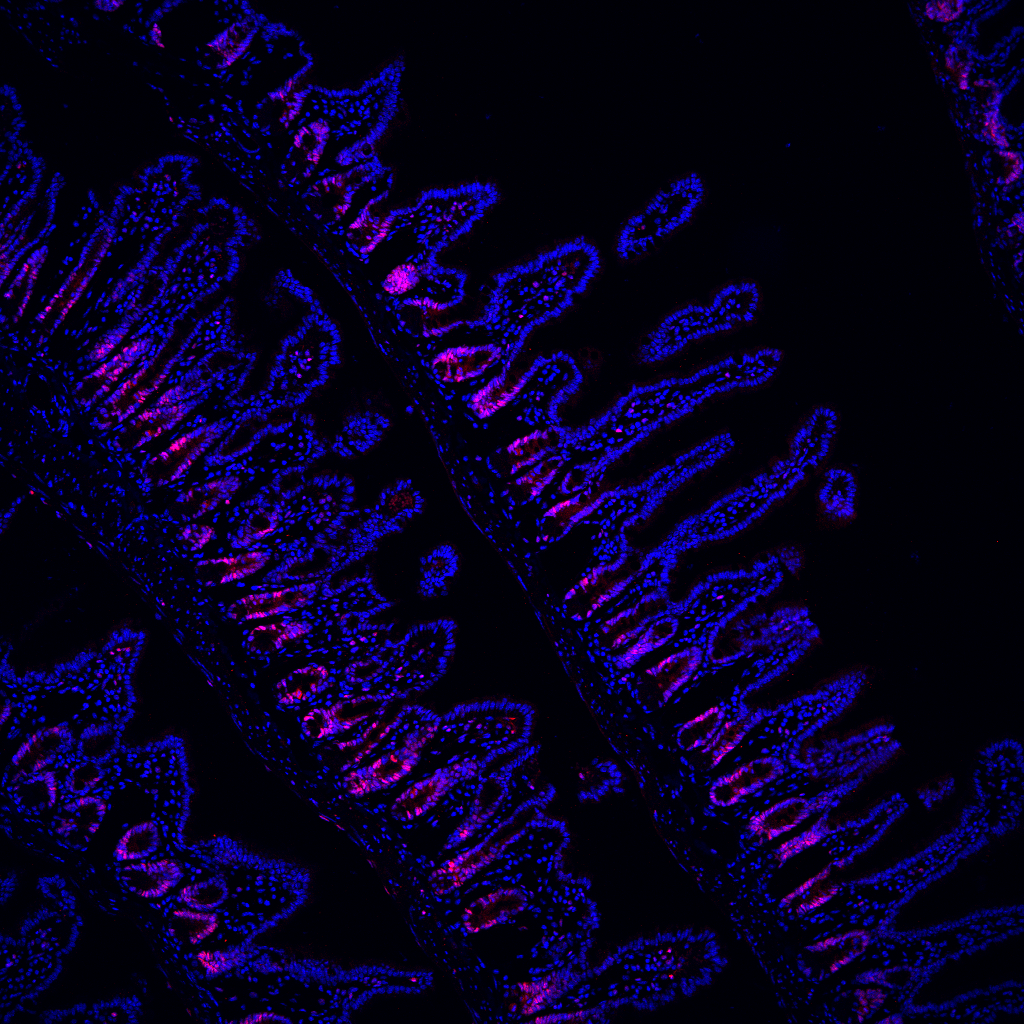

Supplement: Supplementary file 16 — Source Data for Figure 10 [file EMBR-24-e56030-s004.zip › Figure 10/Figure 10F-IHC-CCND1/2. CXCL12 treated EC-Foxc-DKO.tif]
