# Supplementary figures and images for: Endothelial FOXC1 and FOXC2 promote intestinal regeneration after ischemia–reperfusion injury (part 3 of 3)
Source: EMBO Rep. 2023 May 8;24(7):e56030. doi: 10.15252/embr.202256030 (PMC10328078; doi:10.15252/embr.202256030)

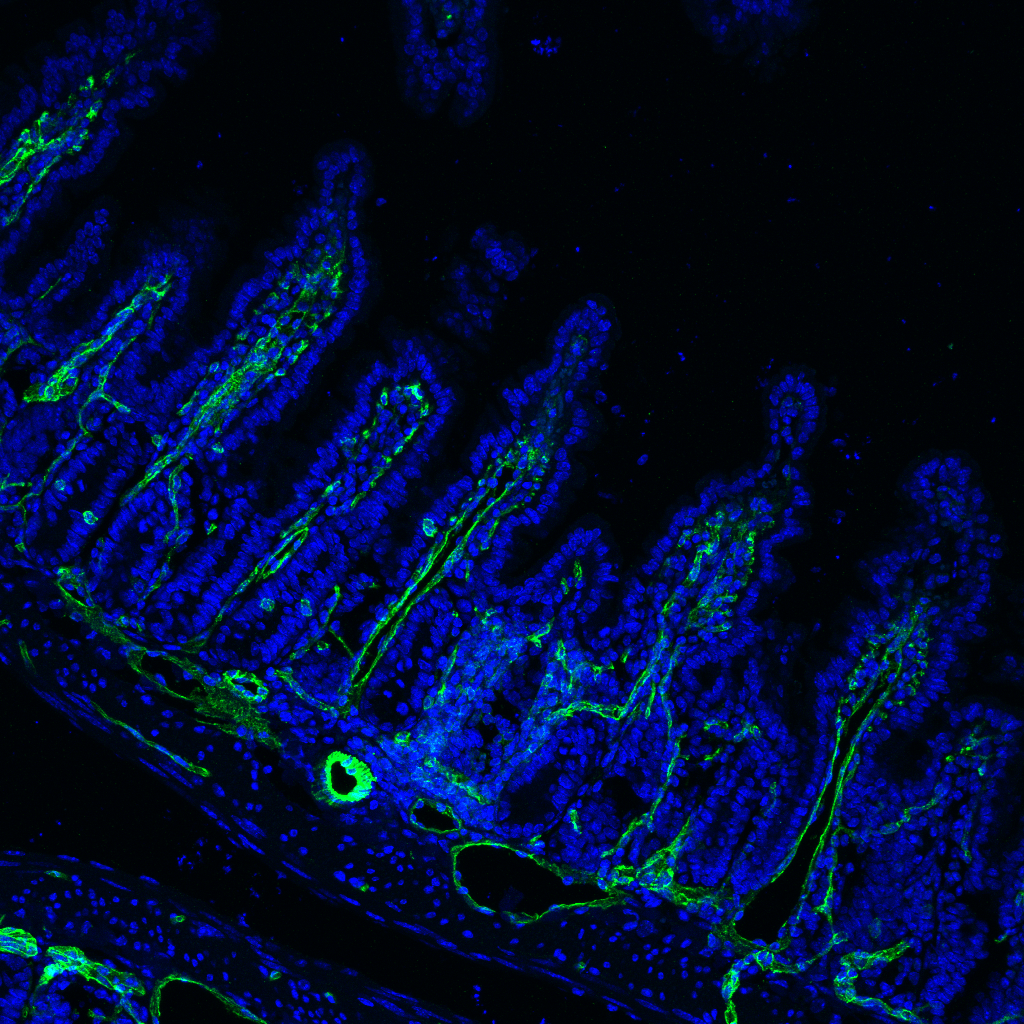

Supplement: Supplementary file 16 — Source Data for Figure 10 [file EMBR-24-e56030-s004.zip › Figure 10/Figure 10H-IHC-CD31/1. PBS treated EC-Foxc-DKO.tif]

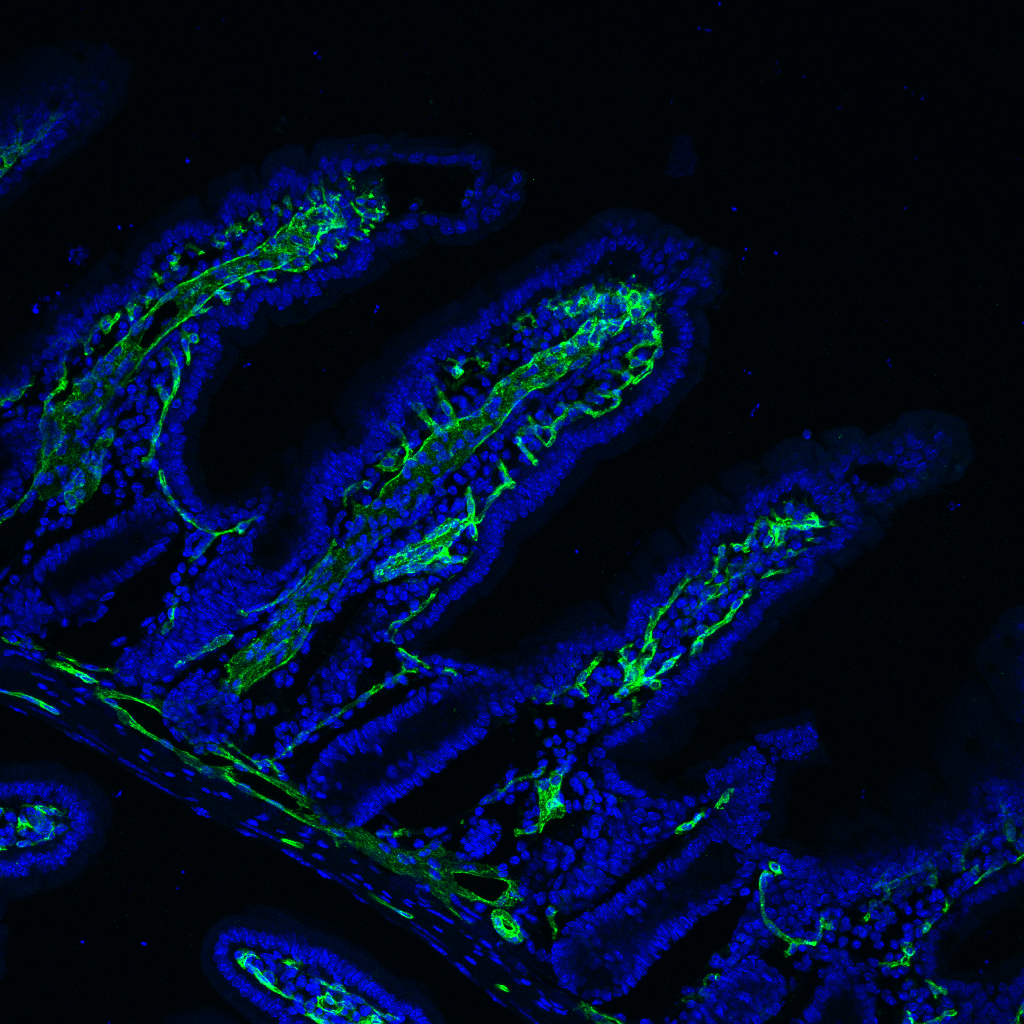

Supplement: Supplementary file 16 — Source Data for Figure 10 [file EMBR-24-e56030-s004.zip › Figure 10/Figure 10H-IHC-CD31/2. CXCL12 treated EC-Foxc-DKO.tif]
